# Controlling the helicity of π-conjugated oligomers by tuning the aromatic backbone twist

**DOI:** 10.1038/s41467-022-28072-7

**Published:** 2022-01-21

**Authors:** Anjan Bedi, Amit Manor Armon, Yael Diskin-Posner, Benny Bogosalvsky, Ori Gidron

**Affiliations:** 1 Institute of Chemistry and the Center for Nanoscience and Nanotechnology, The Hebrew University of Jerusalem, Edmond J. Safra Campus, 9190401 Jerusalem, Israel; 2 Chemical Research Support Unit, Weizmann Institute of Science, 7610001 Rehovot, Israel; 3 Present Address: Department of Chemistry, SRM Institute of Science and Technology, Kattankulathur, 603203 Tamil Nadu India

**Keywords:** Organic chemistry, Physical chemistry

## Abstract

The properties of π-conjugated oligomers and polymers are commonly controlled by side group engineering, main chain engineering, or conformational engineering. The last approach is typically limited to controlling the dihedral angle around the interring single bonds to prevent loss of π-conjugation. Here we propose a different approach to conformational engineering that involves controlling the twist of the aromatic units comprising the backbone by using a tether of varying lengths. We demonstrate this approach by synthesizing an inherently twisted building unit comprised of helically locked tethered acenes, bearing acetylene end-groups to enable backbone extension, which was applied in a series of nine helical oligomers with varying backbone length and twist. We find that the optical and electronic properties of π-conjugated systems may be determined by the additive, antagonistic, or independent effects of backbone length and twist angle. The twisted oligomers display chiral amplification, arising from the formation of secondary helical structures.

## Abstract (web summary)

One approach to altering the properties of π-conjugated oligomers is conformational engineering, in which the degree of rotation around the bonds linking monomers is restricted. Here the authors apply the conformational engineering approach on individual monomers using tethers of varying lengths to twist the aromatic units, and study the effects of varying the angles.

## Introduction

**Fig. 1:**
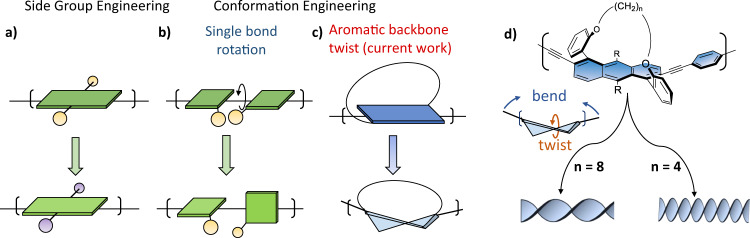
Approaches for tuning the properties of π-conjugated backbones. **a** Side-group engineering. Conformational engineering of the backbone by **b** single bond rotation, and **c** aromatic backbone twist, as introduced in this work. **d** Molecular representation of the tethered twistacene π-conjugated oligomers depicted in **c**. The length of the tether ((CH_2_)_n_, where *n* = 4, 6 or 8 methylene groups; left) determines the primary and secondary structures of the twistacenes by twisting and bending, respectively, and eventually long backbones (polymers) can adopt to a helical conformation with varying degree of helicity. R = CC(CH_3_)_3_.

The field of organic electronics relies on π-conjugated oligomers and polymers whose photophysical, chiroptical, and electronic properties can be tuned using synthetic methodologies^1–3^. One way to modify these properties is by side-group engineering, which involves the addition of electron-accepting or donating, solubilizing, or chiral units to the π-conjugated backbone to obtain desired properties (Fig. 1a)^4–9^. Another approach is main-chain engineering, which is commonly achieved by combining different units in the π-conjugated backbone^10,11^. For example, the inclusion of donor and acceptor systems can result in low bandgap oligomers and polymers, such as those commonly applied in organic solar cells^12^.

In addition to these two approaches, the properties of π-conjugated materials are also dependent on their conformation^13^. Conformational engineering commonly involves controlling the degree of rotation around the inter-unit single bond in the backbone (Fig. 1b) via non-covalent interactions (conformational locking) or by the addition of sterically bulky side groups^14,15^. Rotation around the single bond can result in interesting properties. For example, twisted donor–acceptor systems may have a lower singlet–triplet energy gap than their planar counterparts, which can enable thermally-activated delayed fluorescence (TADF) processes to occur^16,17^. Such rotation can also induce chirality, leading to chiral conducting polymers with a wide range of possible applications, from spin filters to magneto-optic materials^18^. However, as the potential energy surface for single bond rotation is relatively flat, it is hard to control the extent of rotation directly. As a result, conformational engineering depends on the nature of the different aggregation states obtained under different synthetic conditions and, consequently, the approach is of limited practical utility^19^. Moreover, most conformational engineering is applied to avoid rotation around the single bond, with a focus on obtaining a planar conformation^20^. This is because such rotation significantly decreases π-orbital overlap, which in turn increases the bandgap. Thus, an ideal approach to conformational engineering would avoid rotation around the single bonds in favor of controlled distortion of the planarity of the aromatic units themselves (Fig. 1c).

We have recently introduced diagonally tethered helically twisted acenes (twistacenes) in their enantiopure form^21,22^. The twist is induced by combining both molecular tethering^23,24^ and sterics^25^. The length of the tether controls the degree of backbone twisting, and this in turn affects their photophysical and electronic properties. For example, as twisting increases, absorption shifts bathochromically and the rate of intersystem crossing increases at the expense of fluorescence^26^. The chiroptical properties (molar circular dichroism and anisotropy) of twistacenes increase linearly with twisting^27^. We also found that, unlike rotation around a single bond, twisting hardly decreases conjugation^28^. Thus, a particularly attractive feature of our tethered helical twistacenes is that they offer the possibility of tuning backbone conformation in a highly controlled fashion without impairing π-conjugation. However, the extension of such twistacenes to enable their inclusion in longer conjugated systems posed a significant synthetic challenge.

In this work, we introduce a π-conjugated unit, characterized by tunable end-to-end twist and extendable acetylene end-groups (Fig. 1d). Using this family, we investigate the effect on material properties of the addition of repeating anthracene units with controlled twist, to synthesize a family of twisted π-conjugated oligomers up to the trimer (**1-Ant-Cn**; **2-Ant-Cn**; and **3-Ant-Cn**; where **Ant** refers to the anthracene backbone and **Cn** indicates the length of the methylene tether, *n* = 4,6,8). The tether length directly affects the both end-to-end twisting, as well as bending of the anthracene backbone. We map the influence of both variables, namely, backbone twist and length, on key optical and electronic properties. We find that backbone length and twist have synergistic effects on certain properties, such as absorption/emission maxima, while having antagonistic effects on other properties, such as extinction coefficient. By contrast, still other properties are affected primarily by only one of these factors, for example, quantum efficiency is primarily determined by backbone twist angle. Extending the length of the backbone while fully maintaining conjugation strongly amplifies the rate of increase of the molar circular dichroism because of the combined effect of a larger number of twisted units and the gradual formation of a secondary helical structure that exhibits increasing backbone pitch as the oligomer extends. The overall effect is marked chiral amplification with increasing oligomer length. Overall, the method described here constitutes an approach to tailoring the properties of π-conjugated materials: conformational engineering achieved by tuning the backbone twist via the constituent units, rather than via the single interring bond.

## Results

### Design and synthesis

**Fig. 2:**
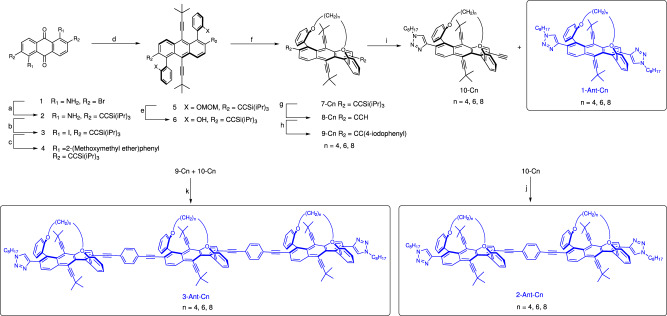
Synthesis of twistacene monomer (1-Ant-Cn), dimer (2-Ant-Cn), and trimer (3-Ant-Cn). **a** HCCSi(^*i*^Pr)_3_, Pd(PPh_3_)_2_Cl_2_ (0.1 equiv.), CuI (0.05 equiv.), THF/Et_3_N (2:1), reflux. **b** NaNO_2_, H_2_SO_4_, KI. **c** 2-(Methoxymethyl ether)phenylboronic acid, Pd(PPh_3_)_4_, dioxane/water (4/1), 94 °C, 5 d; **d** (1) (CH_3_)_3_CCH, n-BuLi, THF, 0 °C → rt, 10 h; (2) SnCl_2_, THF, 4 h; **e** dil. HCl, 40 °C, 16 h. **f** Br-(CH_2_)_n_-Br, K_2_CO_3_, DMF, 40–60 °C *n* = 4, 6, 8; **g** TBAF, THF, 0 °C, 0.5 h; **h** 1,4-diiodobenzene, Pd(PPh_3_)_4_, Et_3_N, rt→50 °C, 36 h; **i** 1-octylazide, CuSO_4_ ∙ 5H_2_O, sodium ascorbate, THF:water (4:1), reflux, 48 h; **j** 1,4-diiodobenzene, Pd(PPh_3_)_4_, THF/Et_3_N (2:1), 65 °C, 22 h; **k** Pd(PPh_3_)_4_, CuI, Et_3_N/THF, 65 °C, 22 h.

The main synthetic challenges in the introduction of extendable groups to tethered twistacenes (**Ant-Cn)** lay in protecting both the extendable groups and the ortho-phenyl groups prior to tethering. The synthetic method is shown in Fig. 2. We protected the extendable acetylene unit with triisopropylsilyl (TIPS) and protected the *ortho*-phenol groups prior to tethering by using 2-(methoxymethyl ether)phenylboronic acid. Both groups proved to be stable throughout the synthesis and did not undergo undesired deprotection. Starting from anthraquinone **1**, a Sonogashira coupling reaction was performed to replace the bromine groups at the 2,6 positions with TIPS-acetylene to yield **2**, followed by a Sandmeyer reaction to convert the amine group to iodine to yield **3**. This enabled Suzuki-Miyaura coupling using 2-(methoxymethyl ether)phenylboronic acid to produce **4**. In this reaction, a mixture of two atropisomers of **4** were obtained, with the methyoxymethyl ether groups in with either *syn* or *anti* conformation with respect to each other, of which only the *syn* isomer successfully reacted in the next steps. Accordingly, heating the mixture of **4** (110 °C in toluene) resulted in equilibration of the two conformers that were then separated using silica gel column chromatography. Compound **4** in the *syn* conformation was isolated in three such repetitive equilibration and column chromatography cycles.

The *syn*-**4** atropisomer was then reacted with 3,3-dimethyl-1-butyne and *n*-butyllithium, followed by SnCl_2_ aromatization to afford compound **5**. Deprotection with dilute HCl solution yielded molecule **6**, also as *syn* and *anti*-isomers. The tethering reaction was performed on **6** under moderate conditions using K_2_CO_3_ to avoid decomposition (with 60% yield). Removal of the protecting groups produced **8-C4**, **8-C6**, and **8-C8**, where **8** consists of an anthracene core helically locked with a diagonal **Cn** tether. Synthesis was not successful for **8-C3**, perhaps because its short tether meant that the strain energy was prohibitively high^29^. The racemic mixture of **8-Cn** was separated to the *M* and *P* enantiomers using preparative chiral HPLC (Chiralpak IB-N and IG columns) with dichloromethane/hexane mixture as eluent (the enantiomers were assigned based on the X-ray structures presented in the next section). Since acetylenes tend to undergo homo-coupling and polymerization, we utilized the “click” copper-catalyzed azide-alkyne cycloaddition (CuAAC) reaction and mild conditions for Sonogashira coupling to produce the monomer **1-Ant-Cn**, compound **10-Cn** and compound **9-Cn**, which were then used for oligomer assembly via another Sonogashira coupling, to obtain dimers and trimers with different twist angles (**2-Ant-Cn** and **3-Ant-Cn**, respectively). The coupling reactions were performed with enantiopure reactants to avoid obtaining a mixture of diastereomers.

### Structure

**Fig. 3:**
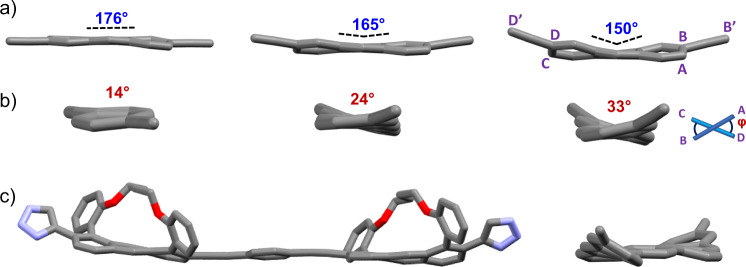
X-ray analysis of twistacenes. **a** Side view and **b** end view for the X-ray structures of the anthracene cores of the monomer precursors **8-C8** (top), **8-C6** (middle), and **8-C4** (bottom), with the substituents and **Cn** tether removed for clarity. The bend angles between B’B and D’D are depicted in blue, and the (calculated) dihedral angles between ABCD are depicted in red. **c** X-ray structure of the dimer, *M*,*M*-**2-Ant-C4**, displaying both its monomeric units in the *syn* conformation, with hydrogens, ^*t*^Bu-acetylene, and n-octyl groups removed for clarity. Right: side view with tether removed for clarity. Color scheme: gray, C; blue, N; red, O.

Figure 3 displays the X-ray structures for all the monomer precursors, **8-Cn**. The X-ray structures of **8-C4** to **8-C8** demonstrate the effect of the tether on the backbone twist of a single unit. All monomers were crystallized from their racemic mixtures to yield racemates. Whereas for **8-C8** the backbone is nearly planar, with an end-to-end twist of 4°, this twist increases to 20° for **8-C6** and further increases to 31° for **8-C4**. Calculated (DFT-B3LYP/6-31 G(d) structures of **8-Cn** display a similar trend, with end-to-end twists of 14°, 24°, and 33° for **8-C8**, **8-C6**, and **8-C4**, respectively. As expected, the difference between the solid state structure and the (calculated) gas phase structure is more pronounced for longer tethers, as these structures are more flexible^21^. For example, when tethering involves a short chain, deprotection of the acetylene backbone barely affects backbone twist angle, with only a 1° difference in twist angle between TIPS-protected **7-C4** (30°) and its deprotected counterpart **8-C4 (**31°). By contrast, with the longer **C8** tether, the difference in twist angle following removal of TIPS is greater, with a 3° difference in twist angle between TIPS-protected **7-C8** (1°) and deprotected **8-C8** (4°). In a similar manner, the addition of triazole does not significantly affect the twist angle, with a difference ranging from 1–3° for **1-Ant-Cn** compared with **8-Cn** (see ESI for the structures of **7-Cn** and **1-Ant-Cn**). In contrast to **8-Cn**, which was crystallized from their racemic mixtures, all **1-Ant-Cn** were crystallized as in their enantiopure form (*P* enantiomer, see ESI for structures), and the *P* and *M* absolute configurations was determined based on both X-ray structures, as well as comparison between experimental and computational ECD spectra (Supplementary Fig. 218, see ESI).

Since the acetylene substituents are positioned diagonally at the 2,6 positions, twisting also affects the relative orientations of the two ends of the tether, that is, the degree of bending, calculated by taking the angle between the vectors D’D and B’B (Fig. 3a). As the tether shortens, the bending increases from 176° for **1-Ant-C8** to 150° for **1-Ant-C4**, corresponding to out of plane bending of 2° for **1-Ant-C8** to 15° for **1-Ant-C4**. This bending can induce a secondary helical structure (discussed below), in addition to the primary helical structure induced by the end-to-end backbone twist.

The X-ray structure of the dimer, **2-Ant-C4** (Fig. 3c), displays nearly the same twist in each of its anthracene units (30 and 31°) as was found for the corresponding monomer **8-C4** (31°). This indicates that coupling (i.e., backbone extension) does not significantly affect backbone twist; rather, the twist angles are directly affected by the inherent twist of the monomer units, which in turn are affected by the length of the tether. The anthracene backbones are in-plane with the phenyl inter-unit linker, which results in retention of full π-conjugation and provides evidence that the twist stems from the anthracene core rather than from rotation around the single bond. The monomer units are in the *syn* conformation with respect to each other, and calculation show that both *syn* and *anti*-conformers have similar energies, whereas non-conjugated conformers are higher in energy.

### The effect of backbone length and twist on photophysical, chiroptical, and electronic properties

**Fig. 4:**
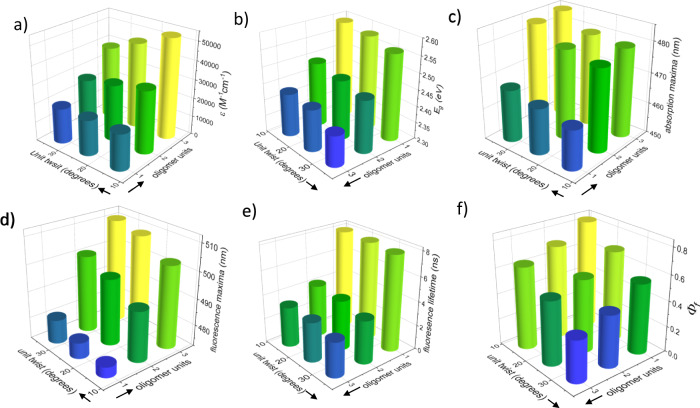
The effect of backbone twist and length on the photophysical properties. 3D graphs displaying the effect of backbone unit twist (*y*-axis) and backbone length (*x*-axis) on **a** extinction coefficients at maximal absorption; **b** optical bandgaps (*E*_g_); **c** absorbance maxima (from the onset of the lowest energy band); **d** fluorescence maxima; **e** fluorescence lifetime; and **f** fluorescence quantum yields (Φ_f_) of **Ant-Cn** monomers, dimers, and trimers. The colors portray the *z*-axis value, from lowest (blue) to highest (yellow).

Our synthesis of a family of π-conjugated oligomers possessing varying tether lengths, backbone lengths, and backbone twist angles provides a means of exploring how oligomer properties are controlled by the number and conformation (length and twist) of their constituent repeat units. Figure 4 shows color maps of various electronic and optical properties by backbone length and twist angle per repeat unit. All values were obtained experimentally. Figure 4a shows that the extinction coefficient decreases with increasing twist and increases with backbone length. This is a direct consequence of the decrease in conjugation arising from the deviation from planarity. However, the effect of twisting is relatively small for the monomer and dimer, as the deviation from planarity does not significantly decrease their conjugation. The extinction coefficient for the longer trimer is significantly more affected by twisting compared with the dimer. Indeed, as twisting increases from 14° to 33°, the extinction coefficient decreases by 37% for the trimer and by 22% for the dimer, which indicates a synergistic effect of backbone length and twist angle. As the extinction coefficient of a conjugated backbone is directly related to the degree of π-orbital overlap, the trimer’s high extinction coefficient indicates that π-conjugation remains strong even with a cumulative end-to-end backbone twist of over 90°.

Figure 4b shows that the optical bandgap, *E*_g(opt)_, is affected to a much greater extent by conjugation length than by twisting, although both have a marked effect. The bandgap decreases with increasing twist, which was previously explained by the LUMO energy-level stabilizing as mixing between π and σ orbitals increases. The bandgap decreases sharply with increasing backbone length, with the effect of length being cumulative, such that the trimer is more affected by twisting compared with the dimer and monomer. Upon further elongation, increased conjugation is expected to decrease the optical bandgap. In a similar manner, the absorption maxima increase with both twisting and conjugation length (Fig. 4c), reaching a maximal value for the most twisted trimer. Twisting has a greater effect on the fluorescence maxima (Fig. 4d) than on the absorption maxima (Fig. 4c). For example, twisting **3-Ant-Cn** shifts its fluorescence maximum by 39 meV whereas it shifts its absorption maximum by only 32 meV, which can be explained by the twist increasing the rigidity of the compound.

The fluorescence lifetime (Fig. 4e) decreases with twist, but this decrease is negligible compared with the strong decrease in fluorescence lifetime with increasing backbone length. In contrast, the fluorescence quantum yield is strongly affected by both backbone twisting and length, but the effect of twisting is greater (Fig. 4f). For a given oligomer length, the fluorescence quantum efficiency decreases 2-fold when moving from a nearly planar to a 31° twisted unit, but decreases only 1.5-fold for a doubling of backbone length from the monomer to the dimer. Different mechanisms produce these differential effects of twisting versus backbone length. The introduction of backbone twist induces a non-radiative intersystem crossing (ISC) pathway, which reduces fluorescence quantum efficiency both by decreasing the singlet–triplet energy gap and by increasing the extent of spin-orbit coupling (SOC)^26,30^. In the case of increasing backbone length, fluorescence quantum yield decreases because of an increase in the degrees of conformational freedom (due to an increase in the number of rotatable triple bonds), which opens non-radiative decay pathways (internal conversion). Consequently, although the fluorescence lifetime decreases with bakcbone length (Fig. 4e), the introduction of a non-radiative pathway means that fluorescent quantum yield also decreases (Fig. 4f). Overall, when compared with the monomer, extension of the conjugation length does not extinguish the effect of unit twist on the electronic and optical properties, and in some cases even amplifies the trends observed in monomers.

### Chiral amplification and secondary structure

**Fig. 5:**
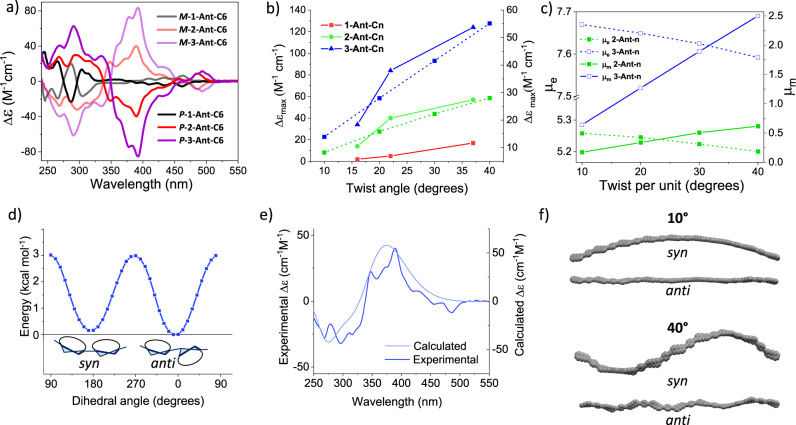
Chiroptical properties and secondary helical structures of twistacenes. **a** Electronic circular dichroism (ECD) spectra of enantiopure **n-Ant-C6** (*n* = 1–3), measured in chloroform. **b** Maximal molar circular dichroism (Δ*ε*_max_) for **n-Ant-C** measured experimentally (solid lines, left *y*-axis) and computationally (dashed lines, right *y*-axis). **c** Calculated electronic transition dipole moment (*μ*_e_, dashed lines) and magnetic transition dipole moment (*μ*_m_, solid lines) for **2-Ant-Cn** (green) and **3-Ant-Cn** (blue). The backbone twist angles were determined computationally. **d** Calculated rotation energy around the inter-unit acetylene bond of **2-Ant-C6**. Calculated at the B3LYP-D3/6-31G(d) level of theory. **e** Measured (dark blue) and calculated (light blue) ECD spectra, consisting of averaged *syn* and *anti* conformers. **f** Optimized (B3LYP/6-31G(d)) structures of anthracene-phenyl skeleton, in syn or anti conformations, with each anthracene unit twisted in 10° (top) or 40° (bottom).

As the tether induces a backbone twist, the twistacene adopts helical chirality and therefore interacts with circularly polarized light. We have previously demonstrated that, upon twisting a monomer, the molar circular dichroism increases linearly with increasing backbone twist^27^. Figure 5a presents the ECD spectra of the *M* and *P* enantiomers of **1-**, **2-**, and **3-Ant-C6** (see ESI for the ECD spectra of the complete **1-**, **2-**, and **3-Ant-Cn** series). The *M* enantiomers exhibit the expected mirror image of the *P* enantiomers, an indication that the observed spectrum is not an artifact, but rather the direct result of backbone twist. For oligomers with the same unit twist, the ECD intensity increases with backbone length, likely because of the increase in oscillator strength.

Within a series consisting of the same number of units, the maximal molar circular dichroism (Δ*ε*_max_) also increases with increasing twist, despite a decrease in the molar extinction coefficient (*ε*) that occurs as the magnetic transition dipole moment (*μ*_m_) increases. However, the increase in molar circular dichroism (Δ*ε*) with twist is more significant as length increases. This difference is expressed in the observed slope in Fig. 5b, with the steepest slope observed for the trimer, **3-Ant-Cn**. The ECD spectra for the backbone (with no substituents and tether) were calculated for the dimer and trimer with varying unit twists, from 10° to 40°, at the TD-DFT/CAM-B3LYP/6-31G(d) level of theory. The slope for the trimer is larger compared with the dimer (Fig. 5b, dashed lines), which is in good agreement with the experimental values (In order to verify that chiral amplification is pesistent for longer oligomers, we have calculated the ECD spectra up to the hexamer. The trend clearly shows that as the length extends, the molar circular dichroism is increasingly affected by the backbone twist, as depicted by increasing slope with backbone length. See Supplementary Fig. 219 in ESI).

The chiral amplification observed both computationally and experimentally cannot be explained as arising solely from a helical primary structure^31^. As oligomer length extends, the backbone is expected to adopt a secondary helical structure. Whereas in the vast majority of helical conjugated polymers, secondary helical structures stem from the effect of auxiliary side chains^32^, in the current case they arise from the intrinsic backbone chirality, inducing increasing twist and bend as the tether length decreases. The presence of a secondary structure is known to increase the molar circular dichroism in a non-linear fashion^31,33–35^. As the molar circular dichroism effect is proportional to the electric and magnetic transition dipole moments (denoted as *μ*_e_ and *μ*_m_, respectively), we followed the calculated values of *μ*_e_ and *μ*_m_ for the skeletons of **2-** and **3-Ant-Cn** (Fig. 5c). Although *μ*_e_ decreases and *μ*_m_ increases with twist in both the dimer and trimer, the extent of increase in *μ*_m_ with increasing twist is significantly greater for **3-Ant-Cn**, whose slope is almost five times larger compared with that of **2-Ant-Cn**. (Fig. 5c). Such an increase can only be explained by a contribution from a secondary helical structure.

The barrier of free rotation around inter-unit acetylene bond was calculated at the B3LYP-D3/6-31G(d) level of theory (Fig. 5d)^36^. The Boltzmann population of both *syn* and *anti* conformers is dominant, while conformers with rotation around the acetylenic bond is negligible. Since the ECD spectrum is very sensitive to conformation, we utilized the predicted spectra to elucidate the dominant conformation in solution, as was previously applied by Diederich^37,38^. We simulated the ECD spectrum for **2-Ant-C6** for different torsion angles around the alkyne bond (Supplementary Fig. 222). The weighted spectra according to the Boltzmann distribution, consisting of *syn* and *anti* conformers matches the experimental spectra of **2-Ant-C6** (Fig. 5e, for similar treatment of **2-Ant-C4** see Supplementary Fig. 223). We therefore consider both the *syn* and *anti* conformers when discussing a secondary helical structure.

To model the effect of different bridge lengths on the structure of long π-conjugated polymers, we extended the optimized structure of trimers of **Ant-Cn** to a 9-mer long chain, in either *syn* or *anti* conformation. Whereas backbones composed of **Ant-C8** are only slightly curved, backbones composed of **Ant-C4** display a helical structure, with a half turn after five repeat units (Fig. 5f). Such helical secondary structure observed for both *syn* and *anti* conformers. Therefore, twisting the π-conjugated backbone results both in twisting of the acene building block and the formation of a secondary helical structure. Consequently, the tether length controls both the extent of helicity of the primary structure (acene end-to-end twist) and the pitch of the helical secondary structure. Whereas for a 40° end-to-end twist a loop is completed after nine units, for a 10° twist it requires 38 units. Overall, the primary structure accounts for the linear increase in the molar circular dichroism for a given monomer twist, and the secondary structure accounts for the amplification of chiroptical properties with backbone length, resulting in the non-linear increase. While chiral amplification of π-conjugated materials is commonly observed for supramolecular assemblies^39–41^, this study demonstrates that chiral amplification can be induced by both primary and secondary structures of π-conjugated backbones.

## Discussion

We introduce a methodology to control the properties of π-conjugated backbones by conformational engineering of the aromatic units rather than of the interring single bond. This was achieved by the synthesis of enantiopure tethered twisted acenes with tunable twist in which acetylenes serve as extension groups. The twistacenes, which exhibit increasing backbone twisting and bending, were utilized as the building unit to construct oligomers with controllable backbone length and twist. We found that their electronic and optical properties can be tuned by controlling both backbone length and twist. Some properties, such as the optical bandgap and the fluorescence maxima, arise from the synergistic effect of increasing backbone length and twist, whereas for other properties, such as the extinction coefficient, the effects of increasing twist and backbone length are antagonistic. For fluorescence quantum efficiency, the effect of backbone twist is more significant than the effect of backbone length.

Oligomers consisting of twisted acene units display strong chiroptical properties, with the molar circular dichroism increasing as either twist or backbone length increase, and a synergistic effect observed when both increase. The non-linear increase in the molar circular dichroism effect suggests chiral amplification, which is explained by the combination of primary and secondary helical structures, both controlled by the tether length.

Overall, the extendable twistacenes with controllable twist presented here can serve as building units for π-conjugated oligomers and polymers whose electronic and optical properties can be tuned while maintaining π-conjugation, and that can be used to introduce chirality directly into the conjugated backbone. We are currently embedding our twistacene units in donor–acceptor systems in order to study the effect of backbone twisting on energy transfer and charge delocalization processes.

## Methods

“The full Methods for this manuscript can be found within the Supplementary Information file which accompanies this paper.”

## Supplementary information

Supplementary Information

## Data Availability

Crystallographic data for the structures reported in this article have been deposited at the Cambridge Crystallographic Data Centre, under deposition numbers CCDC 2099695 (**7-C8**), 2099696 (**7-C4**), 2099699 (**8-C8**), 2099700 (**8-C6**), 2099701 (**8-C4**), 2099698 (*P*-**1-Ant-C8**), 2099697 (*P*-**1-Ant-C6**), 2099197 (*P*-**1-Ant-C4**) and 2099196 (*M,M***-2-Ant-C4**). Copies of the data can be obtained free of charge via https://www.ccdc.cam.ac.uk/structures/. Synthetic and characterization data for all reported compounds as well as computational details and coordinates for optimized structures are included in the Supplementary Information.
