# Supplementary material for: Controlling the helicity of π-conjugated oligomers by tuning the aromatic backbone twist
Source: Nat Commun. 2022 Jan 21;13:451. doi: 10.1038/s41467-022-28072-7 (PMC8782941; doi:10.1038/s41467-022-28072-7)
Supplement: Supplementary file 1 — Supplementary Information [file 41467_2022_28072_MOESM1_ESM.pdf]

Supplementary Information

Controlling the Helicity of π-Conjugated Oligomers by Tuning the Aromatic Backbone Twist

Anjan Bedi[1,2], Amit Manor Armon[1], Yael Diskin Posner[3], Benny Bogosalvsky[1], and Ori Gidron[1]*

[1] Institute of Chemistry, The Hebrew University of Jerusalem, Edmond J. Safra Campus, Jerusalem, Israel. [2] Current address: Department of Chemistry, SRM Institute of Science and Technology, Kattankulathur 603203, Tamil Nadu, India. [3] Chemical Research Support Unit, Weizmann Institute of Science, Rehovot, Israel. *email: ori.gidron@mail.huji.ac.il

Contents

Supplementary Methods 4
S1 General 4
S2 Synthesis 5
S2.1 Synthesis of the twistacene building block 5
S2.1.1 Supplementary Scheme 1. Synthesis of 8-Cn 5
Synthesis of 2 6
Synthesis of 3 6
Synthesis of 4 7
Synthesis of 5 8
Synthesis of 6 10
Synthesis of 7-C8 11
Synthesis of 7-C6 12
Synthesis of 7-C4 13
Synthesis of 8-C8 14
Synthesis of 8-C6 15
Synthesis of 8-C4 15
S2.2 Chiral HPLC of racemic 8-Cn series 16
S2.3 Synthesis of the enantiopure oligomers 17
Supplementary Scheme 2.3.1 Synthesis of the enantiopure oligomers 1-Ant-Cns, 2-Ant-Cns and 3-Ant-Cns 17
Synthesis of P-9-C8 18
Synthesis of P-9-C6 19
Synthesis of M-9-C6 20
Synthesis of P-9-C4 21
Synthesis of P-10-C8 and P-1-Ant-C8 21
Synthesis of P-10-C6 and P-1-Ant-C6 23
Synthesis of M-10-C6 and M-1-Ant-C6 25
Synthesis of P-10-C4 and P-1-Ant-C4 27
S2.4 General method for synthesis of 2-Ant-Cns 28
Synthesis of P,P-2-Ant-C8 28
Synthesis of P,P-2-Ant-C6 30

Synthesis of M,M-2-Ant-C6 30
Synthesis of P,P-2-Ant-C4 31
S2.5 General method for synthesis of the 3-Ant-Cn series 33
Synthesis of P,P,P-3-Ant-C8 33
Synthesis of P,P,P-3-Ant-C6 34
Synthesis of M,M,M-3-Ant-C6 35
Synthesis of P,P,P-Ant-C4 35
S3 Characterization 37
S4 Photophysical properties 236
S5 Single crystal X-ray diffraction crystallography (SCXRD) 240
S6 Computational details 244
S6.1 Calculated structures of the analogs of the synthesised molecules 244
Supplementary Table 4. Optimized (DFT-B3LYP-6-31G(d)) structures of the analogs of the synthesized molecules 244
6.1.1 Optimized (DFT-B3LYP-6-31G(d)) geometries of 8-Cn 244
6.1.2 Optimized (DFT-B3LYP-6-31G(d)) geometries of 1-Ant-Cn 245
6.1.3 Optimized (DFT-B3LYP-6-31G(d)) geometries of 2-Ant-Cn 245
6.1.4 Optimized (DFT-B3LYP-6-31G(d)) geometries of 3-Ant-Cn 245
S6.2 Computational insights from the twisted parent anthracene oligomers 247
Supplementary Table 5. Optimized (DFT-B3LYP-6-31G(d)) structures of the M-Ant-Cn skeleton 247
6.2.1 Optimized (DFT-B3LYP-6-31G(d)) geometries of the M-Ant-Cn skeleton 247
6.2.2 Optimized (DFT-B3LYP-6-31G(d)) geometries of the syn-M,M-2-Ant-Cn skeleton 247
6.2.3 Optimized (DFT-B3LYP-6-31G(d)) geometries of the M,M,M-3-Ant-Cn skeleton 248
6.2.4 Transition dipole moment of the skeletons M,M-2-Ant-40 and M,M,M-3-Ant-40 251
Supplementary References 253

Supplementary Methods

S1 General

Commercially available reagents and chemicals were used without further purification unless otherwise stated. 2-(Methoxymethoxy)phenylboronic acid was received from Angene Chemicals. Compound **1** was synthesized according to a previous report[1] and the crude mixture was used for the next step.

Flash chromatography (FC) was performed using CombiFlash SiO_2 columns. Chiral HPLC separations were performed with a Chiralpak® IG semi-preparative column and CHIRALPAK® IB-N (250 × 4.6 mm / 5μm) preparative columns, with hexane/dichloromethane as eluent.

^{1}H and ^{13}C NMR spectra were recorded in solution on a Bruker-AVIII 400 MHz and 500 MHz spectrometers using tetramethylsilane (TMS) as the external standard. The spectra were recorded using chloroform-d and $C_2D_2Cl_4$ as the solvent. Chemical shifts are expressed in δ units.

UV-vis absorption spectra were recorded with an Agilent Cary-5000 spectrophotometer. The spectra were measured using a quartz cuvette (1 cm) at 25 °C. The absorption wavelengths are reported in nm with the extinction coefficient ε ($M^{-1}cm^{-1}$) in brackets.

Steady state fluorescence measurements were performed on a HORIBA JOBIN YVON Fluoromax-4 spectrofluorometer with the excitation/emission geometry at right angles. Fluorescence quantum yields (ϕ_f) were determined using a standard procedure under a HORIBA-Φ integrating sphere. The lifetimes of the excited species were measured using an NL-C2 Pulsed Diode Controller NanoLED light source with time-correlated single proton counting (TSCPC) Controller DeltaHub (HORIBA), referenced against colloidal Ludox solution (50 wt. % solution in water) obtained from Aldrich. Electronic Circular Dichroism (ECD) spectra were recorded on a MOS-500 spectrophotometer from BioLogic Science Instruments.

High resolution mass spectra were measured on a HR Q-TOF LCMS and Waters Micromass GCT_Premier Mass Spectrometer using ESI. MALDI-TOFMS spectra were acquired using an MALDI-TOF/TOF autoflex speed mass spectrometer (Bruker Daltonilk GmbH, Bremen, Germany) equipped with a smartbeam-II solid-state laser (modified Nd:YAG laser) λ = 355 nm. The instrument was operated in positive ion, reflectron mode. The accelerating voltage was 21.0 kV. The delay time was 130 ns. Laser fluence were optimized for each sample. The laser was fired

at a frequency of 2 kHz and spectra were accumulated in multiples of 500 laser shots to achieve 1500 shots in total. Sample preparation: 2-[(2E)-3-(4-tert-Butylphenyl)-2-methylprop2-enylidene]malononitrile (DCTB) matrix solutions were made to a concentration of 20 mg/mL in dichloromethane (DCM). Sample solutions were made to an approximate concentration of 5 mg/mL in DCM. Sample and matrix solutions were premixed in ratio of 1:10 or 1:40 (v/v). A volume of 0.5 µL of this mixture was disposed on a MALDI steel target plate. After evaporation of the solvent, the target was inserted into the mass spectrometer. IR spectra were recorded on a Perkin Elmer BX FT-IR spectrophotometer.

S2 Synthesis

S2.1 Synthesis of the twistacene building block

S2.1.1 Supplementary Scheme 1. Synthesis of 8-Cn

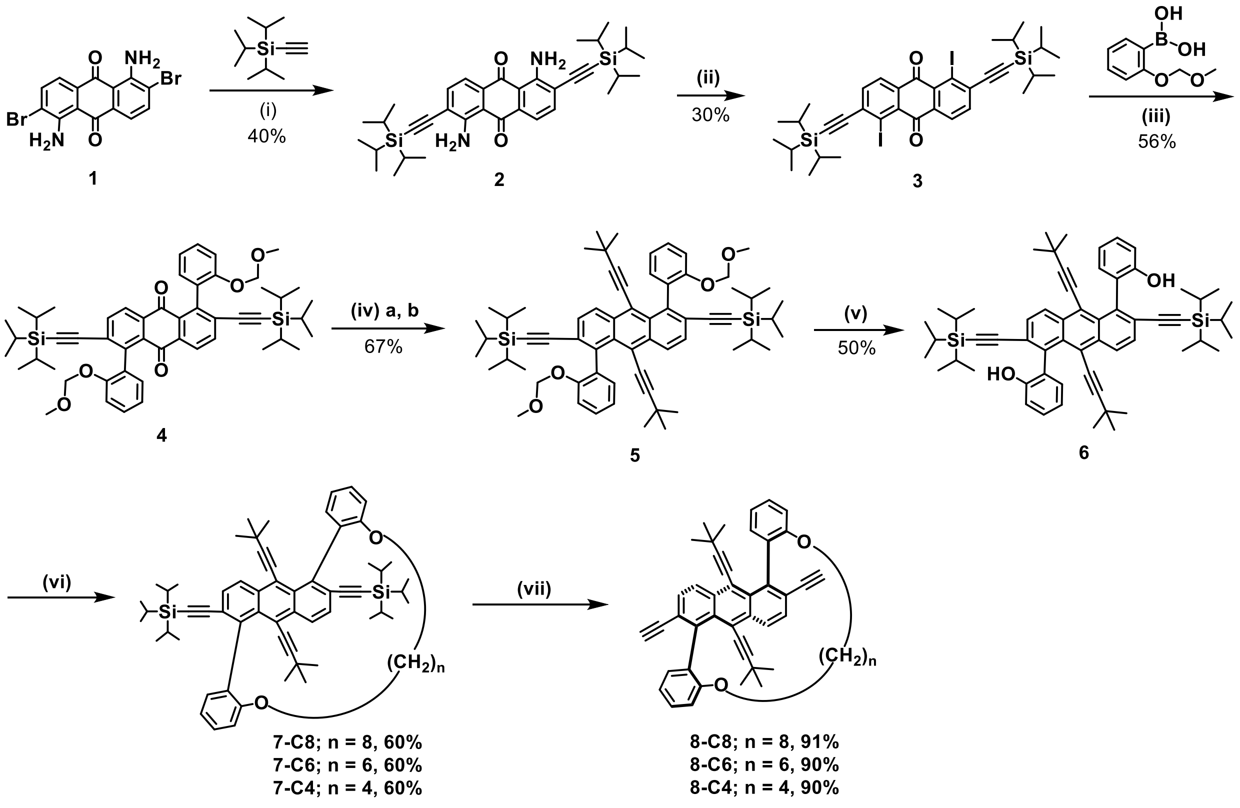

(i) $Pd(PPh_3)_2Cl_2$, CuI, THF/Et_3N (2:1), reflux; (ii) $NaNO_2$, H_2SO_4, KI; (iii) $Pd(PPh_3)_4$, dioxane/water (4:1), 94 °C, 5 d; (iv) (a) 3,3-Dimethyl-1-butyne, n-BuLi, THF, −5 °C→RT, 10 h; (b) $SnCl_2$, THF, 4 h; (v) dil. HCl, 40 °C, 16 h; (vi) Br-$(CH_2)_n$-Br, K_2CO_3, DMF, 40–60 °C; (vii) *N*,*N*,*N*,*N*-tetra-^{n}butylammonium fluoride, THF, 0 °C→RT, 0.5 h.

Synthesis of 2

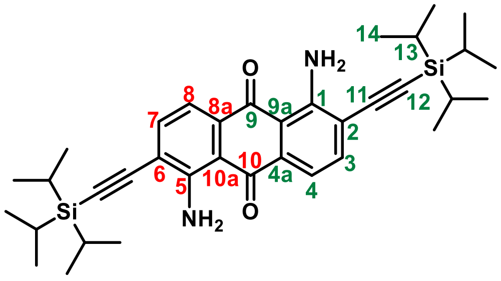

THF (40 mL) and triethylamine (20 mL) were mixed in an oven-dried two-necked round bottomed (RB) flask equipped with a magnetic stirrer and a condenser, and purged thoroughly with Ar for 20 min. Compound **1** (25 g, 0.15 mmol), triisopropylsilyl acetylene (11.2 mL, 0.22 mmol) and CuI (1.5 mg, 0.008 mmol) were added to the mixture under positive Ar flow followed by $Pd(PPh_3)_2Cl_2$ (262 mg, 0.015 mmol). The mixture was then heated to reflux for 4 h. The solvent was removed in a rotavapor, and the resulting semi-solid mixture was subjected to silica gel chromatography using dichloromethane/hexane (1:9) to afford a blood-red solid (yield = 21.5 g, 40%).

^{1}H NMR (400 MHz, Chloroform-d) δ 7.63 (d, J = 7.8 Hz, 2H, H-C(3)), 7.55 (d, J = 7.8 Hz, 2H, H-C(4)), 1.16 (br, 42H, H-C(13), H-C(14)).

^{13}C NMR (101 MHz, Chloroform-*d*) δ 184.87 (C9), 151.40(C1), 137.53 (C3), 135.09 (C4a), 115.41 (C4), 115.05 (C2), 113.23 (9a), 101.84 (C11), 101.75 (12), 18.72 (14), 11.24 (13).

HR-ESI-MS m/z (%): 598.3474 (100, $[M+H]^+$) calcd. for $C_{36}H_{51}N_2O_2Si_2^+$: 599.3484.

UV-vis ($CHCl_3$): λ_{max} (ε) = 265 (59229), 297 (10522), 315 (9279), 517 nm (17169 M^{-1} cm^{-1}).

Synthesis of 3

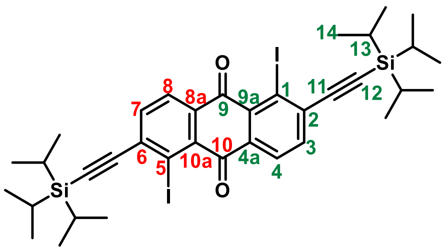

Conc. H_2SO_4 (25 mL) was added to a finely ground powder of **2** (7 g, 11.68 mmol), kept in a 1000 mL RB flask and stirred for 10 min. The flask was placed in an ice-bath and a solution of sodium nitrite (4.03 g, 58.40 mmol) in water (25 mL) was added in five portions, which caused the

formation of a brown gas. After stirring the reaction mixture for 3 h to produce a yellow reaction mixture, a solution of potassium iodide (9.69 g, 58.40 mmol) in water (25 mL) was added in three portions. A dark brown cake formed immediately, which slowly became a dispersed solution. The reaction mixture was allowed to reach RT and stirred for 9 h. The conversions were monitored by TLC. Then, water (200 mL) was added to the reaction mixture and stirred vigorously for 45 min followed by the addition of chloroform (200 mL) and another 45 min of stirring. The double diluted resulting mixture was filtered through a Bückner funnel and the organic layer was separated from the liquor via a separatory funnel. The clear organic layer was washed with satd. $NaHCO_3$ and satd. $Na_2S_2O_3$ solution, which produced a clear yellow solution that was dried over $MgSO_4$. Chloroform was evaporated off in a rotavapor and the resulting brownish-yellow crude product was subjected to silica gel column chromatography using DCM/hexane (3:17) to afford an orange solid. Finally, washing the solid with hexane (40 mL) furnished **3** as a canary yellow solid (4.4 g, yield = 30%; m.p. 210-212 °C).

^{1}H NMR (400 MHz, Chloroform-d) δ 8.27 (d, J = 8.1 Hz, 2H, H-C(4), 7.81 (d, J = 8.1 Hz, 2H, H-C(3)), 1.18 (d, J = 4.0 Hz, 42H, H-C(13), H-C(14)).

^{13}C NMR (101 MHz, Chloroform-d) δ 180.30 (C9), 139.45 (C2), 137.18 (C3), 134.70 (C4a), 133.16 (C9a), 127.86 (C4), 108.22 (C1), 102.86 (C12), 99.03 (11), 18.73 (C14), 11.31 (C13).

HR-ESI-MS m/z (%): 821.1172 (100, $[M+H]^+$) calcd. for $C_{36}H_{47}I_2O_2Si_2^+$: 821.1198.

UV-vis ($CHCl_3$): λ_{max} (ε) = 256 (28752), 301 (35588), 394 nm (9764 M^{-1} cm^{-1}).

Synthesis of 4

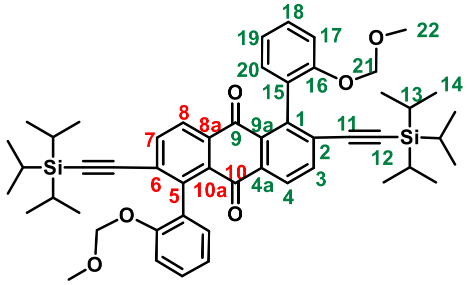

A two-necked RB flask fitted with a condenser was evacuated and refilled with Ar three times. 1, 4-Dioxane (80 mL) and water (20 mL) were added and the mixture was purged with Ar for 20 min before adding **3** (16 g, 19.02 mmol), 2-(methoxymethoxy)phenylboronic acid (7.61 g, 41.84

mmol), Na_2CO_3 (26.61 g, 251.04 mmol) and $Pd(PPh_3)_4$ (4.834 g, 4.184 mmol) at once. The reaction mixture was then kept at 94 °C for 5 days. Then, 1, 4-dioxane was evaporated off and the resulting mixture was extracted with chloroform (3 × 90 mL). The combined organic layers are dried over $MgSO_4$ and concentrated to obtain a brown-yellow crude. The purification of the crude by silica gel column chromatography using DCM/hexane (2:3) resulted in an orange solid. Washing the solid with hexane (50 mL) afforded **4** as a golden-yellow solid (10 g, yield = 60%) as a mixture of conformational isomers arising from different alignments (*syn*- or *anti*-periplanar) of the methoxymethyl groups. Interestingly, only *syn*-**4** reacted in the expected manner in the next synthetics steps. So, heating the mixture of conformational isomers in toluene above 110 °C resulted in an irreversible equilibrium concentration that underwent room temperature purification using silica gel column chromatography, as mentioned earlier. Finally, *syn*-**4** (8.89 g, 56%; m.p. 261-263 °C) was isolated in three such repetitive equilibration and column chromatography cycles.

^1H NMR (400 MHz, Chloroform-d) δ 8.11 (d, J = 8.1 Hz, 2H, H-C(4)), 7.86 (d, J = 8.1 Hz, 2H, H-C(3)), 7.36 (ddd, J = 8.4, 6.6, 2.5 Hz, 2H, H-C(18)), 7.22 (dd, J = 8.1, 0.9 Hz, 2H, H-C(17)), 7.14 – 7.07 (m, 4H, H-C(20), H-C(19)), 5.08 – 4.97 (m, 4H, H-C(21)), 3.26 (s, 6H, H-C(22)), 0.93 (s, 42H, H-C(13), H-C(14)).

^{13}C NMR (101 MHz, Chloroform-d) δ 182.67 (C9), 153.99 (C16), 142.12 (C1), 137.31 (C3), 134.50 (C4a), 131.10 (C2), 131.06 (9a), 130.10 (C15), 129.19 (C20), 128.85 (C18), 126.84 (C4), 121.97 (C19), 114.43 (C17), 104.39 (C11), 99.83 (C12), 94.62 (C21), 55.71 (C22), 18.47 (C14), 11.04 (C13).

HR-ESI-MS m/z (%): 863.4103 (100, $[M+Na]^+$) calcd. for $C_{52}H_{64}NaO_6Si_2^+$: 863.4134.

UV-vis ($CHCl_3$): λ_{max} (ε) = 283 (44251), 309 (37684), 363 nm (11680 M^{-1} cm^{-1}).

Synthesis of 5

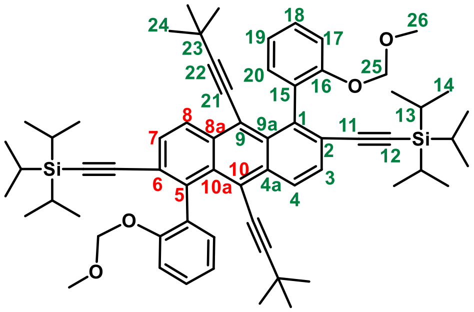

Step 1: An oven-dried two-necked RB flask was charged with anhydrous tetrahydrofuran and kept at −5 °C for 10 min and then 3,3-dimethyl-1-butyne (8.09 g, 98.6 mmol) was added to it. To the chilled solution, n-BuLi (1.6M in hexanes, 55.46 mL) was added slowly to maintain the temperature and the reaction mixture was kept at the same temperature for one hour to complete lithiation. The lithium-alkynide salt was quenched by dropwise addition of a solution of *syn*-**4** (8.3 gm, 9.86 mmol) in 20 mL of anhydrous tetrahydrofuran, and fading of the golden-yellow color of **4** was observed. The resulting reaction mixture was kept at -5 °C for 0.5 h before allowing it rise to RT, at which temperature it was then stirred for an additional hour. Then, the reaction mixture was quenched with water and the solvents were evaporated off in a rotavapor, producing an off-white solid. The solid was washed with water and hexane and dried under vacuum to afford a white solid (8.5 g) which was immediately used in the next step without further purification and characterization.

Step 2: The white solid produced at the end of Step 1 was reacted with $SnCl_2 \cdot 5H_2O$ for 4 h in THF under Ar to accomplish full conversion as monitored by TLC and the appearance of a bright yellow color and a bright green emission under a 365 nm lamp. The THF was distilled off and the resulting orange crude product was purified by silica gel column and eluted with DCM/hexane (5:95) to afford a bright yellow solid **5** (6.44 g, 67% of two steps; m.p. 210-212 °C).

^{1}H NMR (400 MHz, Chloroform-*d*) δ 8.63 (d, *J* = 9.1 Hz, 2H, H-C(4)), 7.57 (d, *J* = 9.1 Hz, 2H, H-C(3)), 7.39 – 7.29 (m, 4H, , H-C(18), , H-C(20)), 7.16 (dd, *J* = 8.3, 1.1 Hz, 2H, H-C(17)), 7.06 (td, *J* = 7.4, 1.1 Hz, 2H, H-C(19)), 5.00 (d, *J* = 6.6 Hz, 2H, H-C(25)), 4.95 (d, *J* = 6.6 Hz, 2H, H-C(25)), 3.20 (s, 6H, , H-C(26)), 1.15 (s, 18H, , H-C(24)), 0.97 (s, 42H, H-C(13) , H-C(14)).

^{13}C NMR (101 MHz, Chloroform-d) δ 155.64 C(16), 140.28 C(1), 134.59 (4a), 132.32 (C20), 131.80 (C15), 129.95 (C9a), 129.51 (C3), 129.07 (C18), 127.22 (C4), 123.46 (C2), 121.63 (C19), 119.41 (C9), 116.03 (C22), 114.62 (C17), 107.24 (C11), 96.12 (C21), 94.54 (C25), 78.42 (C12), 55.78 (C26), 30.84 (C24), 28.54 (C23), 18.61 (C14), 11.22 (C13).

HR-ESI-MS m/z (%): 971.5836 (100, $[M+H]^+$) calcd. for $C_{64}H_{83}O_4Si_2^+$: 971.5830.

UV-vis ($CHCl_3$): λ_{max} (ε) = 309 (70464), 321 (155845), 395 (3868), 418 (8315), 444 (14513), 472 nm (18431 M^{-1} cm^{-1}).

Synthesis of 6

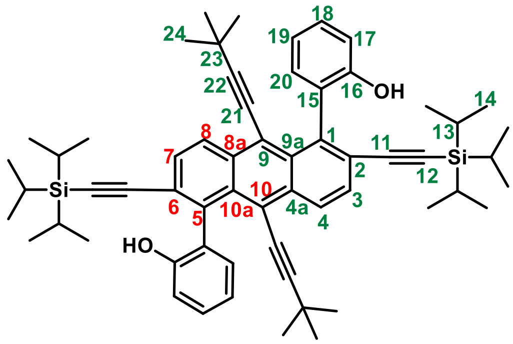

To a solution of **5** (6.44 g, 6.62 mmol) in 30 mL THF/MeOH (4:1) was added 0.55 mL of conc. HCl (35-40% in water) under Ar at RT and kept at 40 °C. The reaction was monitored by NMR spectroscopy to confirm complete consumption of **5** after 12 h. The reaction was diluted with water and then the organic layer was separated in a separatory funnel. The aqueous layer was extracted with ethyl acetate (3 × 50 mL). The organic extracts were combined and dried over $MgSO_4$ and concentrated in a rotavapor at < 40 °C to produce a dark green semi-solid mass. Further purification by silica gel column chromatography using DCM/hexane (1:3) afforded a greenish yellow gummy solid, which upon washing with hexane (10 mL) furnished **6** (2.92 g, 50%) as a yellow powder with a bright green emission under a 365 nm lamp. The final product was a combination of the epimers *syn*-**6** (2.60 g; m.p. 310-314 °C (decomp.)) and *anti*-**6** (0.32g).

^{1}H NMR (400 MHz, Chloroform-d) δ 8.64 (d, *J* = 9.1 Hz, 2H, H-C(4)), 7.61 (d, *J* = 9.1 Hz, 2H, H-C(3)), 7.30 (dd, J = 7.8, 1.8 Hz, 2H, H-C(18)), 7.17 – 7.12 (m, 2H, H-C(20)), 7.00 – 6.91 (m, 4H , H-C(19), H-C(17)), 4.68 (s, 2H, -O-H), 1.15 (s, 18H, H-C(24)), 0.99 (d, J = 2.0 Hz, 42H, H-C(13) , H-C(14)).

^{13}C NMR (101 MHz, Chloroform-d) δ 153.52 (C16), 137.23 (C1), 134.92 (C4a), 131.92 (C20), 130.30 (C9a), 129.85 (C3), 129.51 (C18), 128.32 (C4, C15), 124.95 (C2), 120.51 (C19), 119.47 (C9), 117.13 (C22), 115.78 (C17), 105.84 (C11), 98.24 (C21), 30.65 (C24), 28.57 (C23), 18.58 (C14), 11.19 (C13).

HR-ESI-MS m/z (%): 883.5270 (100, $[M+H]^+$) calcd. for $C_{60}H_{75}O_2Si_2^+$: 883.5306.

UV-vis ($CHCl_3$): λ_{max} (ε) = 323 (145409), 397 (4934), 421 (9388), 449 (14474), 478 nm (17732 M^{-1} cm^{-1}).

Synthesis of 7-C8

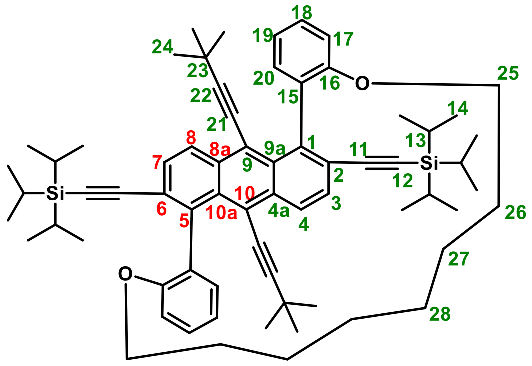

Anti-**6** (1.00 g, 1.13 mmol) and anhydrous K_2CO_3 (1.56 g, 11.3 mmol) were purged with Ar in a two-necked RB flask under positive Ar flow and the 20 mL of anhydrous *N*,*N*-DMF was added to the mixture. After stirring the solution for 4 h at 60 °C, formation of the dipotassio salt was confirmed by a change in color from greenish yellow to brown and complete quenching of the bright green emission. Then, 1,8-dibromooctane (0.216 g, 0.79 mmol) was added and the reaction was again continued at 60 °C for 48 h. Then, *N*,*N*-DMF was distilled off the reaction mixture in a rotavapor and the resulting dark semisolid was subjected to silica gel column chromatography using DCM/hexane (1:19), to afford a bright yellow solid **7-C8** (0.67 g, 60%; m.p. 273-276 °C) with green emission under a 365 nm lamp.

^{1}H NMR (400 MHz, $CDCl_3$) δ 8.63 (d, *J* = 9.2 Hz, 2H, H-C(4)), 7.56 (d, J = 9.2 Hz, 2H, H-C(3)), 7.37 – 7.31 (qd, J = 7.5, 1.7 Hz, 4H, H-C(20, H-C(18)), 7.01 (td, J = 7.4, 0.9 Hz, 2H, H-C(19)), 6.86 (d, J = 7.8 Hz, 2H, H-C(17)), 3.94 (dt, J = 8.8, 4.3 Hz, 2H, H-C(25)), 3.77 (td, J = 8.4, 4.4 Hz, 2H, H-C(25)), 1.31 (m, 4H, H-C(26)), 1.13 (s, 18H, H-C(24)), 0.96 (d, J = 3.3 Hz, 42H, H-C(13) , H-C(14)), 0.56 (t, J = 26.6 Hz, 8H, H-C(27) , H-C(28)).

^{13}C NMR (101 MHz, $CDCl_3$) δ 157.7 (C16), 140.8 (C16), 134.6 (4a), 132.1 (C20), 131.3 (C15), 129.8 (C9a), 129.2 (C3), 128.9 (C18), 127.3 (C4), 123.2 (C2), 120.2 (C19), 119.6 (C9), 115.5 (C22), 111.6 (C17), 107.5 (C11), 95.6 (C21), 78.7 (C12), 68.9 (C25), 30.9 (C24), 29.7 (C26), 29.3 (C27), 28.5 (C23), 27.1 (C28), 18.6 (C14), 11.2 (C13).

HR-ESI-MS m/z (%): 993.6413 (100, $[M+H]^+$) calcd. for $C_{68}H_{89}O_2Si_2^+$: 993.6401.

UV-vis ($CHCl_3$): λ_{max} (ε) = 309 (73265), 322 (159311), 395 (3965), 417 (8335), 443 (14769), 472 nm (18649 M^{-1} cm^{-1}).

Synthesis of 7-C6

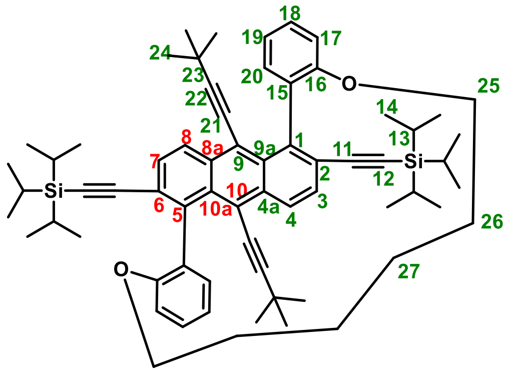

Compound **6** (1.00 g, 1.13 mmol) and K_2CO_3 (1.56 g, 11.3 mmol) were purged with Ar in a two-necked RB flask under positive Ar flow and 20 mL of anhydrous *N*,*N*-DMF was added to the mixture. After stirring the solution for 10 min at RT, formation of the dipotassio salt of the twistacene was confirmed by a change in color from greenish yellow to brown and complete quenching of the bright green emission. The dipotassio salt was quenched with 1,6-dibromohexane (0.193 g, 0.791 mmol) and the reaction was again continued at 60 °C for 48 h. Then, the reaction mixture was distilled to remove the *N*,*N*-DMF in a rotavapor and subjected to silica gel column chromatography using DCM/hexane (1:19) to afford bright yellow solid **7-C6** (0.65 g, 60%; m.p. 304-306 °C) with green emission under a 365 nm lamp.

^{1}H NMR (500 MHz, $CDCl_3$) δ 8.55 (d, *J* = 9.1 Hz, 1H, H-C(4)), 7.67 (dd, *J* = 7.4, 1.7 Hz, 1H, H-C(20)), 7.57 (d, *J* = 9.1 Hz, 1H, H-C(3)), 7.33 (m, 1H, H-C(18)), 7.04 (td, *J* = 7.4, 1.0 Hz, 1H, H-C(19)), 6.79 (d, *J* = 8.2 Hz, 1H, H-C(17)), 3.71 (ddd, *J* = 9.6, 6.1, 3.8 Hz, 1H, , H-C(25)), 3.59 (td, *J* = 8.7, 3.1 Hz, 1H, H-C(25)), 1.13 – 1.04 ((s, 20H, H-C(24), H-C(26)), 0.99 (br, 18H, H-C(14)), 0.87 – 0.76 (m, 2H H-C(26)), 0.52 – 0.35 (m, 4H, H-C(27)).

^{13}C NMR (126 MHz, $CDCl_3$) δ 157.4 (C16), 140.3 (C1), 133.9 (C4a), 132.6 (C20), 130.9 (C15), 130.3 (C9a), 129.6 (C3), 128.9 (C18), 126.8 (C4), 123.0 (C9), 120.3 (C19), 119.5 (C2), 114.8 (C22), 112.1 (C17), 107.7 (C11), 96.0 (C21), 78.2 (C12), 68.2 (C25), 30.8 (C24), 29.4 (C26), 28.4 (C23), 25.4 (C27), 18.6 (C14), 11.2 (C13).

HR-ESI-MS m/z (%): 965.6076 (100, $[M+H]^+$) calcd. for $C_{66}H_{85}O_2Si_2^+$: 965.6088.

UV-vis ($CHCl_3$): λ_{max} (ε) = 309 (70392), 322 (143859), 395 (3959), 418 (8156), 445 (13977), 473 nm (17387 M^{-1} cm^{-1}).

Synthesis of 7-C4

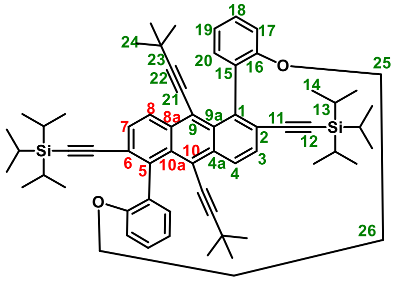

Syn-**6** (1.00 g, 1.13 mmol) and K_2CO_3 (1.56 g, 11.3 mmol) were purged with Ar in a two-necked RB flask under positive Ar flow and 20 mL of anhydrous *N*,*N*-DMF was added to the mixture. After stirring the solution for 10 min at RT, formation of the dipotassio salt of the twistacene was confirmed by a change in color from greenish yellow to brown and complete quenching of the bright green emission. The dipotassio salt was quenched with 1,4-dibromobutane (0.171 g, 0.791 mmol) and the reaction was continued at 60 °C for 48 h. Then, the reaction mixture was distilled to remove *N*,*N*-DMF a in rotavapor and subjected to silica gel column chromatography using DCM/hexane (1:19), to afford bright yellow solid **7-C4** (0.64 g, 60%; m.p. 302-304 °C) with green emission under a 365 nm lamp.

^{1}H NMR (500 MHz, $CDCl_3$) δ 8.30 (dd, *J* = 9.0, 1.1 Hz, 2H, H-C(4)), 7.94 (dt, *J* = 7.5, 1.4 Hz, 2H, H-C(20)), 7.54 (dd, *J* = 9.0, 1.1 Hz, 2H, H-C(3)), 7.25 (d, *J* = 4.9 Hz, 2H, H-C(18)), 7.02 (s, 1H, H-C(19)), 6.61 (s, 2H, H-C(17)), 3.60 (m, 2H, H-C(25)), 3.28 (d, *J* = 8.4 Hz, 2H, H-C(25)), 1.10 (d, *J* = 1.4 Hz, 18H, H-C(24)), 1.04 (d, *J* = 2.0 Hz, 42H, H-C(14) , H-C(13)), 0.79 (dd, *J* = 13.1, 2.2 Hz, 2H, H-C(26)), 0.62 – 0.52 (m, 2H, H-C(26)).

^{13}C NMR (126 MHz, $CDCl_3$) δ 155.9 (C16), 139.1 (C1), 132.8 (C4a), 132.1 (C20), 131.9 (C9a), 129.7 (C3), 129.5 (C15), 128.7 (C18), 125.8 (C4), 121.9 (C2), 119.7 (C19), 118.4 (C9), 113.4 (C22), 109.9 (C17), 107.6 (C11), 95.8 (C21), 77.2 (C22), 66.0 (C25), 30.7 (C24), 28.3 (C23), 26.1 (C26), 18.6 (C14), 11.3 (C13).

HR-ESI-MS m/z (%): 937.5795 (100, $[M+H]^+$) calcd. for $C_{64}H_{81}O_2Si_2^+$: 937.5775.

UV-vis ($CHCl_3$): λ_{max} (ε) = 313 (68888), 326 (138981), 401 (4010), 424 (8068), 451 (13919), 480 nm (17208 M^{-1} cm^{-1}).

Synthesis of 8-C8

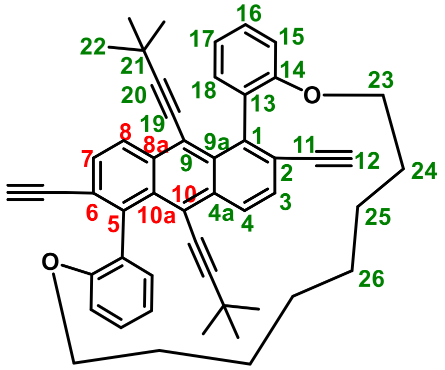

To a solution of **7-C8** (0.67 g, 0.678 mmol) in 20 mL THF, kept under Argon in a single-neck RB flask was added dropwise *N*,*N*,*N*,*N*-tetrabutylammonium fluoride (TBAF) solution (1 M in THF, 1.42 mL) at 0°C. Then, the reaction mixture was stirred at room temperature for an additional 0.5 h. The THF was evaporated off in a rotavapor and the resulting semisolid crude was directly subjected to silica gel column chromatography using DCM/hexane (1:4) to obtain a bright yellow solid **8-C8** (0.42 g, yield = 91%; m.p. 274-276 °C) with green emission under a 365 nm lamp.

^{1}H NMR (400 MHz, $CDCl_3$) δ 8.65 (d, *J* = 9.2 Hz, 2H, H-C(4)), 7.57 (d, *J* = 9.2 Hz, 2H, H-C(3)), 7.45 – 7.41 (m, 2H, H-C(16)), 7.38 (dd, *J* = 7.4, 1.7 Hz, 2H, H-C(18)), 7.09 (td, *J* = 7.4, 1.0 Hz, 2H, H-C(17)), 6.94 (dd, *J* = 8.2, 0.8 Hz, 2H, H-C(15)), 3.97 – 3.93 (dt, *J* = 8.9, 4.3 Hz, 2H, H-C(23)), 3.81 – 3.76 (m, 2H, H-C(23)), 2.98 (s, 2H, H-C(12)), 1.32 (s, 4H, H-C(24)), 1.13 (s, 18H, H-C(22)), 0.67 – 0.51 (m, 8H, H-C(25) , H-C(26)).

^{13}C NMR (101 MHz, $CDCl_3$) δ 157.7 (C14), 141.5 (C1), 134.7 (C4a), 132.1 (C18), 131.2 (C13), 129.8 (C9a), 129.1 (C16), 128.9 (C3), 127.5 (C4), 122.1 (C2), 120.1 (C17), 119.8 (C9), 116.0 (C20), 111.9 (C15), 83.9 (C11), 81.5 (C12),78.6 (C19), 69.2 (C23), 30.8 (C22), 29.5 (C25), 29.2 (C24), 28.5 (C21), 26.8 (C26).

HR-ESI-MS m/z (%): 680.3625 (100, $[M]^+$) calcd. for $C_{50}H_{48}O_2^+$: 680.3654.

UV-vis ($CHCl_3$): λ_{max} (ε) = 308 (121894), 390 (3131), 414 (7205), 440 (14966), 468 nm (18885 M^{-1} cm^{-1}).

Synthesis of 8-C6

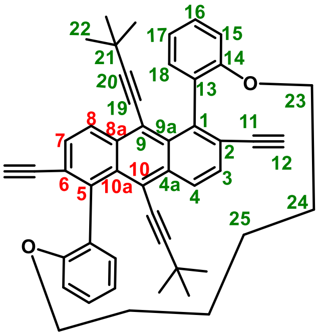

This was synthesized using same procedure as for **8-C8**, starting from **7-C6** (0.65g, 0.678 mmol) and 1.42 mL TBAF solution (mmol) to obtain a bright yellow solid, **8-C6** (0.40 g, yield = 90%; m.p. 253-255 °C) with green emission under a 365 nm lamp.

^{1}H NMR (400 MHz, $CDCl_3$) δ 8.57 (d, *J* = 9.1 Hz, 1H, H-C(4)), 7.69 (dd, *J* = 7.5, 1.7 Hz, 1H, H-C(18)), 7.58 (d, *J* = 9.1 Hz, 1H, H-C(3)), 7.43 (ddd, *J* = 8.1, 7.5, 1.7 Hz, 1H, H-C(16)), 7.11 (td, *J* = 7.5, 1.0 Hz, 1H, H-C(17)), 6.86 – 6.84 (m, 2H, H-C(15)), 3.73 (ddd, *J* = 9.6, 5.7, 3.8 Hz, 1H) , H-C(23), 3.63 (td, *J* = 8.7, 3.0 Hz, 1H, H-C(23)), 3.04 (s, 2H, H-C(12)), 1.13 (m, 2H, H-C(24)), 1.11 – 1.06 ((S, 18H, H-C(22), 0.84 – 0.77 (m, 2H, H-C(24)), 0.49 – 0.35 (m, 4H, H-C(25)).
^{13}C NMR (101 MHz, CDCl3) δ 157.3 (C14), 141.0 (C1), 134.0 (C4a), 132.6 (C18), 130.6 (C13), 130.3 (C9a), 129.3 (C3), 129.2 (C16), 127.1 (C4), 121.7 (C2), 119.9 (C17), 119.7 (C9), 115.2 (C20), 112.1 (C15), 84.23 (C11), 81.9 (C12), 78.1 (C19), 68.2 (C23), 30.8 (C22), 29.4 (C24), 28.4 (C21), 25.4 (C25).

HR-ESI-MS m/z (%): 652.3309 (100, $[M]^+$) calcd. for $C_{48}H_{44}O_2^+$: 652.3341.

UV-vis ($CHCl_3$): λ_{max} (ε) = 310 (114991), 390 (3288), 395 (3965), 415 (7279), 442 (14918), 470 nm (18786 M^{-1} cm^{-1}).

Synthesis of 8-C4

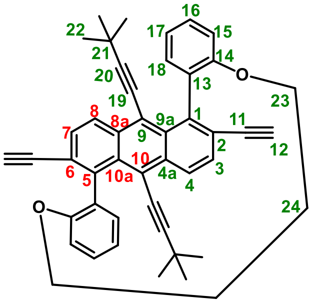

This was synthesized using same procedure as for **8-C8**, starting from **7-C4** (0.64 g, 0.678 mmol) and 1.42 mL TBAF solution to obtain a bright yellow solid, **8-C4** (0.38 g, yield = 90%; m.p. 230-232 °C) with green emission under a 365 nm lamp.

^{1}H NMR (500 MHz, $CDCl_3$) δ 8.32 (d, *J* = 9.0 Hz, 2H, H-C(4)), 7.95 (dd, *J* = 7.5, 1.7 Hz, 2H, H-C(18)), 7.53 (d, *J* = 9.0 Hz, 2H, H-C(3)), 7.34 (qd, *J* = 7.4, 1.7 Hz, 2H, H-C(16)), 7.11 (td, *J* = 7.5, 1.1 Hz, 2H, H-C(17)), 6.67 (dd, *J* = 8.2, 1.1 Hz, 2H, H-C(15)), 3.65 (dd, *J* = 8.4, 1.9 Hz, 2H, H-C(23)), 3.34 – 3.26 (m, 2H, H-C(23)), 3.10 (s, 2H, H-C(12)), 1.11 (s, 18H, H-C(22)), 0.79 (m, 2H, H-C(24)), 0.58 – 0.47 (m, 2H, H-C(24)).

^{13}C NMR (126 MHz, $CDCl_3$) δ 155.8 (C14), 139.8 (C1), 132.9 (C4a), 132.0 (C18), 131.8 (C9a), 129.3 (C3), 129.2 (C13), 129.1 (C16), 126.0 (C4), 120.6 (C2), 119.3 (C17), 118.6 (C9), 113.8 (C20), 110.0 (C15), 84.2 (C11), 81.7 (C12), 77.2 (C19), 65.8 (C23), 30.7 (C22), 28.3 (C21), 25.9 (C24).

HR-ESI-MS m/z (%): 624.2996 (100, $[M]^+$) calcd. for $C_{46}H_{40}O_2^+$: 624.3028.

UV-vis ($CHCl_3$): λ_{max} (ε) = 314 (109131), 396 (3718), 421 (7784), 417 (8335), 447 (15170), 476 nm (18619 M^{-1} cm^{-1}).

S2.2 Chiral HPLC of racemic 8-Cn series

Racemic **8-C8** and **8-C6** were resolved using semi-preparative CHIRALPAK-IG column using 35% DCM/hexane as the eluent. By contrast, **8-C4** was resolved using CHIRALPAK® IB-N (250 × 4.6mm/5μm) preparative columns using dichloromethane/hexane (1:4) as the eluent. In both methods, the *M*-enantiomer eluted first followed by the *P*-enantiomer with baseline separation between them.

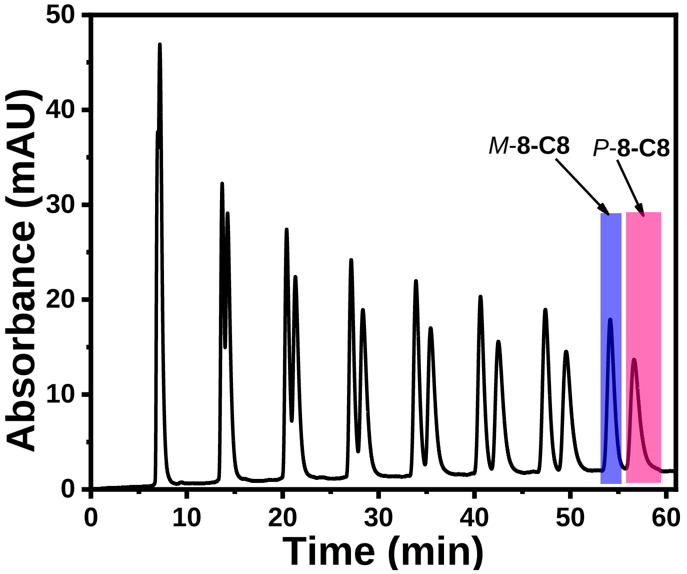

Supplementary Figure 1. Chiral HPLC of racemic-**8-C8**

S2.3 Synthesis of the enantiopure oligomers

Supplementary Scheme 2.3.1 Synthesis of the enantiopure oligomers 1-Ant-Cns, 2-Ant-Cns and 3-Ant-Cns

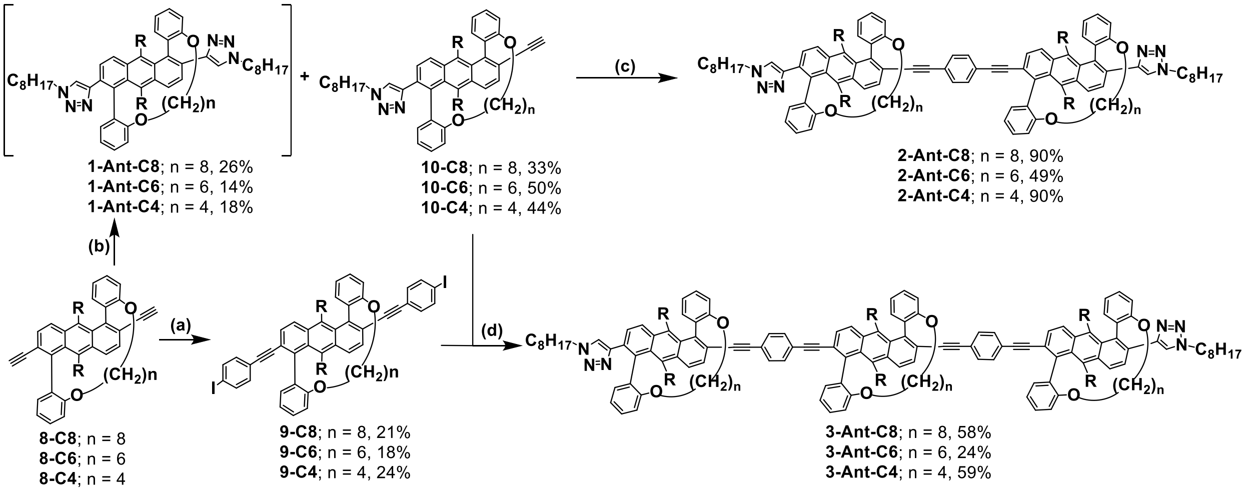

Reagents and conditions: (a) 1,4-diiodobenzene, $Pd(PPh_3)_4$, Et_3N, RT-50°C, 36 h; (b) 1-octylazide, $CuSO_4·5H_2O$, sodium ascorbate, THF/water (4:1), reflux, 48 h; (c) 1,4-diiodobenzene, $Pd(PPh_3)_4$, THF/Et_3N (2:1), 65°C, 22 h; (d) $Pd(PPh_3)_4$, CuI, Et_3N/THF, 65°C, 22 h.

Synthesis of P-9-C8

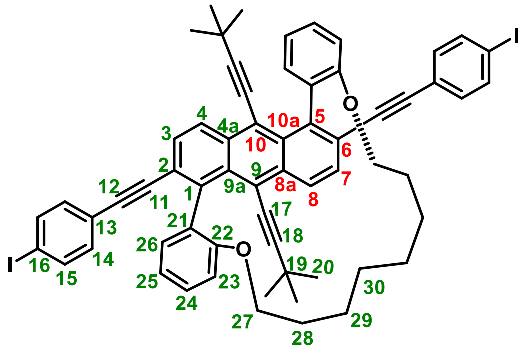

To a two necked RB flask kept under positive Ar flow were added *P*-**8-C8** (30 mg, 0.044 mmol), 1,4-diiodobenzene (31.97 mg, 0.097 mmol), $Pd(PPh_3)_4$ (1.12 mg, 0.00097 mmol), and CuI (0.37 mg, 0.001894 mmol). Triethylamine (2 mL) was purged with Ar for 5 min and then added to the previously mixed reagents and the reaction mixture was stirred for 28 h at room temperature until complete consumption of *P*-**8-C8** was observed by TLC. The solvent was evaporated and the reaction mixture was directly loaded on a silica gel column and eluted slowly with DCM/hexane (1:9) to afford *P*-**8-C8** as a deep yellow solid (10 mg, yield = 21%; m.p. 215-217 °C) with weak green emission under a 365 nm lamp.

^{1}H NMR (400 MHz, $CDCl_3$) δ 8.70 (d, *J* = 9.2 Hz, 2H, H-C(4)), 7.61 (d, *J* = 9.2 Hz, 2H, H-C(3)), 7.58 – 7.57 (m, 4H, H-C(15)), 7.47 – 7.42 (m, 4H, H-C(24) , H-C(26)), 7.11 (td, *J* = 7.4, 1.0 Hz, 2H, , H-C(25)), 6.96 (d, *J* = 8.1 Hz, 2H, H-C(23)), 6.84 – 6.81 (m, 4H, H-C(14)), 3.95 (dt, *J* = 8.9, 4.3 Hz, 2H, H-C(27)), 3.83 (dt, *J* = 8.8, 6.1 Hz, 2H, H-C(27)), 1.31 (M, 4H, H-C(28)), 1.15 (s, 18H, H-C(20)), 0.68 – 0.53 (m, 8H, H-C(29) , H-C(30)).

^{13}C NMR (101 MHz, $CDCl_3$) δ 158.1 (C22), 141.2 (C1), 137.2 (C15), 134.6 (C4a), 133.0 (C14, C16), 132.5 (C26), 131.7 (C21), 129.8 (C9a), 128.9 (C24), 128.1 (C3), 127.6 (C4), 123.1 (C13), 122.9 (C2), 120.0 (C25), 119.7 (C9), 115.9 (C18), 111.9 (C9), 93.64 (C12), 92.1 (C11), 78.8 (C17), 69.3 (C27), 30.8 (C20), 29.5 (C30), 29.2 (C28), 28.5 (C19), 26.9 (C29).
HR-ESI-MS m/z (%): 1084.2243 (100, $[M]^+$) calcd. for $C_{62}H_{54}I_2O_2^+$: 1084.2213.

UV-vis ($CHCl_3$): λ_{max} (ε) = 338 (91556), 353 (210790), 405 (7429), 428 (12274), 453 (17128), 483 nm (21153 M^{-1} cm^{-1}).

Synthesis of P-9-C6

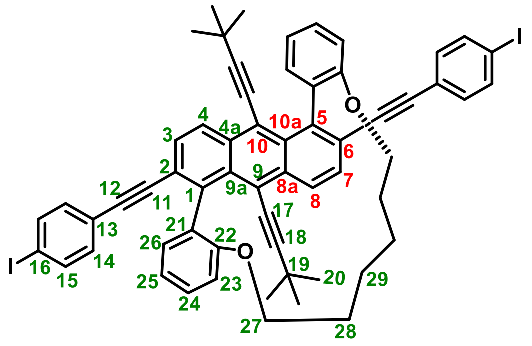

P-**9-C6** was synthesized according to the same procedure as was used for *P*-**9-C8**, starting from *P*-**8-C6** (30 mg, 0.046 mmol), 1,4-diiodobenzene (33.38 mg, 0.10 mmol), $Pd(PPh_3)_4$ (1.15 mg, 0.0010 mmol), CuI (0.38 mg, 0.0020 mmol), triethylamine (3 mL). *P*-**9-C6** was isolated as a deep yellow solid (9 mg, yield = 18%; m.p. 208-210 °C) with weak green emission under a 365 nm lamp.

^{1}H NMR (400 MHz, $CDCl_3$) δ 8.62 (d, *J* = 9.1 Hz, 2H, H-C(4)), 7.74 (dd, *J* = 7.5, 1.7 Hz, 2H, H-C(26)), 7.62 (m, 2H, H-C(3), 7.59 (m, 2H, H-C(15)), 7.46 – 7.40 (m, 2H, H-C(24)), 7.14 (dd, *J* = 7.5, 1.0 Hz, 2H, H-C(25)), 6.92 (m, 2H, H-C(14)), 6.87 (d, *J* = 7.5 Hz, 2H,H-C(23)), 3.74 (ddd, *J* = 9.0, 5.5, 3.9 Hz, 2H, H-C(27)), 3.64 (td, *J* = 8.8, 2.8 Hz, 2H, H-C(27), 1.16 (br, 2H, H-C(28), 1.12 (s, 18H), 0.82 (m, 2H, H-C(28)), 0.55 – 0.31 (m, 2H, H-C(29)).

^{13}C NMR (400 MHz, $CDCl_3$) δ 157.5 (C22), 140.6 (C1), 137.2 (C15), 134.0 (C4a), 132.9 ((C14), (C16)), 132.8 (C26), 131.1 (C21), 130.3 (C9a), 129.1 (C24), 128.5 (C3), 127.2 (C4), 123.2 (C13), 122.6 (C2), 119.8 (C25), 119.6 (C9), 115.1 (C18), 112.2 (C23), 93.7 (C12), 92.4 (C11), 78.2(C17), 68.2 (C27), 30.8 (C20), 29.5 (C28), 28.4 (C19), 25.4 (C29).

HR-ESI-MS m/z (%): 1056.1925 (100, $[M]^+$) calcd. for $C_{60}H_{50}I_2O_2^+$: 1056.1900.

UV-vis ($CHCl_3$): λ_{max} (ε) = 338 (84168), 354 (181387), 406 (7296), 430 (11659), 455 (15042), 485 nm (18309 M^{-1} cm^{-1}).

Synthesis of M-9-C6

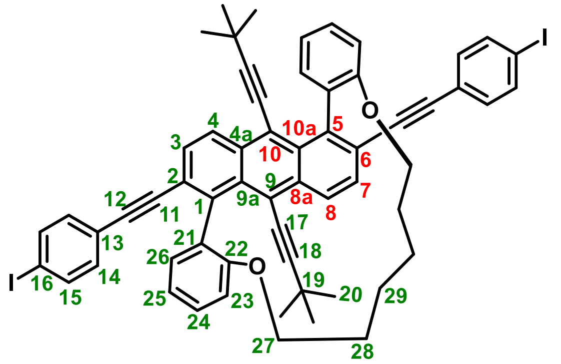

M-**9-C6** was synthesized according to the same procedure as was used for *P*-**9-C8**, starting from *M*-**8-C6** (30 mg, 0.046 mmol), 1,4-diiodobenzene (33.38 mg, 0.10 mmol), $Pd(PPh_3)_4$ (1.15 mg, 0.0010 mmol), CuI (0.38 mg, 0.0020 mmol) and triethylamine (3 mL). *M*-**9-C6** was isolated as a deep yellow solid (9 mg, yield = 18% m.p. 225-227 °C) with weak green emission under a 365 nm lamp;.

^{1}H NMR (400 MHz, $CDCl_3$) δ 8.62 (d, *J* = 9.1 Hz, 2H, H-C(4)), 7.74 (dd, *J* = 7.5, 1.7 Hz, 2H, H-C(26)), 7.62 (m, 2H, H-C(3), 7.59 (m, 2H, H-C(15)), 7.46 – 7.40 (m, 2H, H-C(24)), 7.14 (dd, *J* = 7.5, 1.0 Hz, 2H, H-C(25)), 6.92 (m, 2H, H-C(14)), 6.87 (d, *J* = 7.5 Hz, 2H,H-C(23)), 3.74 (ddd, *J* = 9.0, 5.5, 3.9 Hz, 2H, H-C(27)), 3.64 (td, *J* = 8.8, 2.8 Hz, 2H, H-C(27), 1.16 (br, 2H, H-C(28), 1.12 (s, 18H), 0.82 (m, 2H, H-C(28)), 0.55 – 0.31 (m, 2H, H-C(29)).

^{13}C NMR (400 MHz, $CDCl_3$) δ 157.5 (C22), 140.6 (C1), 137.2 (C15), 134.0 (C4a), 132.9 ((C14), (C16)), 132.8 (C26), 131.1 (C21), 130.3 (C9a), 129.1 (C24), 128.5 (C3), 127.2 (C4), 123.2 (C13), 122.6 (C2), 119.8 (C25), 119.6 (C9), 115.1 (C18), 112.2 (C23), 93.7 (C12), 92.4 (C11), 78.2(C17), 68.2 (C27), 30.8 (C20), 29.5 (C28), 28.4 (C19), 25.4 (C29).

HR-ESI-MS m/z (%): 1056.1925 (100, $[M]^+$) calcd. for $C_{60}H_{50}I_2O_2^+$: 1056.1900.

UV-vis ($CHCl_3$): λ_{max} (ε) = 338 (84168), 354 (181387), 406 (7296), 430 (11659), 455 (15042), 485 nm (18309 M^{-1} cm^{-1}).

Synthesis of P-9-C4

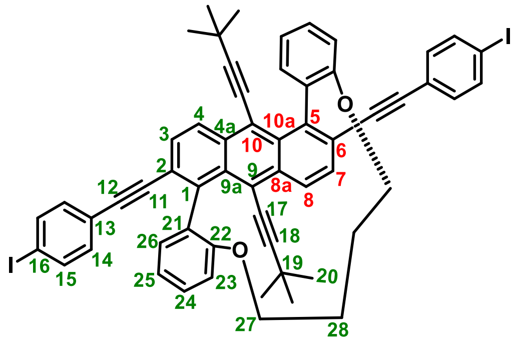

P-**9-C4** was synthesized according to the same procedure as was used for used for *P*-**9-C8**, starting from *P*-**8-C4** (30 mg, 0.048 mmol), 1,4-diiodobenzene (36.29 mg, 0.11 mmol), $Pd(PPh_3)_4$ (1.27 mg, 0.00011 mmol), CuI (0.42 mg, 0.0022 mmol) and triethylamine (4 mL). *P*-**9-C4** was isolated as a deep yellow solid (12 mg, yield = 24%; m.p. 201-203 °C) with weak green emission under a 365 nm lamp.

^{1}H NMR (500 MHz, $CDCl_3$) δ 8.36 (dd, *J* = 9.0, 1.0 Hz, 2H, H-C(4)), 8.02 (dd, *J* = 7.4, 1.5 Hz, 2H, H-C(26)), 7.63 (m, 4H, H-C(15)), 7.57 (dd, *J* = 9.0, 1.0 Hz, 2H, H-C(3)), 7.37 (m, 2H, H-C(24)), 7.16 (dd, *J* = 7.9, 7.0 Hz, 2H, H-C(25)), 7.05 (m, 4H, H-C(14)), 6.69 (d, *J* = 7.7 Hz, 2H, H-C(23)), 3.67 (s, 2H, H-C(27)), 3.33 (m, 2H, H-C(27)), 1.12 (S, 18H, H-C(20)), 0.83 (m, 2H, H-C(28)), 0.57 (m, 2H, H-C(28)).

^{13}C NMR (126 MHz, $CDCl_3$) δ 155.9 (C22), 139.4 (C1), 137.3 (C15), 132.9 (C4a), 132.8 (C14, C16), 132.1 (C26), 132.0 (C9a), 129.6 (C21), 129.0 (C24), 128.6 (C3), 126.1 (C4), 123.2 (C13), 121.4 (C2), 119.1 (C25), 118.5 (C9), 113.8 (C18), 110.1 (C23), 93.7 (C12), 93.0 (C17), 92.4 (C11), 65.9 (C27), 30.7 (C20), 28.4 (C19), 25.9 (C28).

HR-ESI-MS m/z (%): 1028.1600 (100, $[M]^+$) calcd. for $C_{58}H_{46}I_2O_2^+$: 1028.1587.

UV-vis ($CHCl_3$): λ_{max} (ε) = 341 (80099), 35 (161071), 410 (6672), 434 (10796), 461 (14233), 491 nm (16866 M^{-1} cm^{-1}).

Synthesis of P-10-C8 and P-1-Ant-C8

$CuSO_4{\cdot}5H_20$ (12.84 mg, 0.051 mmol) and sodium ascorbate (13.60 mg, 0.068 mmol) were added to *P*-**8-C8** (100.09 mg, 0.147 mmol) and 1-octylazide (22.85 mg, 0.147 mmol) in 5 mL THF/water (3:2) and kept at 60 °C 36 hours. The reaction mixture was distilled to remove THF and then

diluted with water. The aqueous mixture was extracted with dichloromethane (2 × 10 mL) and concentrated to produce an orange semisolid mass. Three fractions were recovered during further purification using silica gel column chromatography. The first fraction eluted at EtOAc/hexane (1/95) was *P*-**8-C8** (10.00 mg). Second fraction eluted at EtOAc/hexane (3:17) was *P*-**10-C8** (40 mg, yield = 33%; m.p. 303-305 °C) and the final fraction eluted at EtOAc/hexane (1:4) was *P*-**1-Ant-C8** (38 mg, yield = 26%; m.p. 198-200 °C) as a yellow solid with sea-green emission under a 365 nm lamp.

P-10-C8:

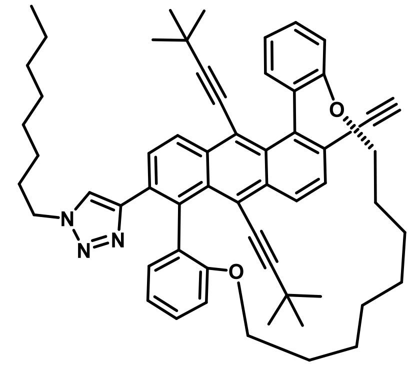

^{1}H NMR (500 MHz, Chloroform-d) δ 8.81 (d, *J* = 9.4 Hz, 1H), 8.66 (d, *J* = 9.2 Hz, 1H), 8.47 (d, *J* = 9.4 Hz, 1H), 7.55 (d, *J* = 9.2 Hz, 1H), 7.52 – 7.40 (m, 3H), 7.21 (dd, *J* = 7.4, 1.7 Hz, 1H), 7.08 (td, *J* = 7.4, 1.0 Hz, 1H), 7.02 (td, *J* = 7.4, 1.0 Hz, 1H), 6.94 (d, *J* = 8.3 Hz, 2H), 5.74 (s, 1H, -N-C=C-H), 4.11 (q, *J* = 6.9 Hz, 2H), 4.01 – 3.93 (m, 1H), 3.88 – 3.73 (m, 3H), 2.98 (s, 1H), 1.67 (p, *J* = 7.1 Hz, 2H)), 1.29 (br, 12H), 1.15 (br, 20H), 0.89 (t, *J* = 7.0 Hz, 3H), 0.74 – 0.35 (m, 8H).

^{13}C NMR (126 MHz, Chloroform-*d*) δ 158.4, 157.7, 146.6, 141.5, 134.8, 134.8, 133.5, 132.7, 132.2, 131.6, 131.3, 130.1, 129.8, 129.4, 129.3, 129.1, 128.7, 128.0, 127.4, 126.7, 121.9, 121.6, 121.1, 120.1, 119.9, 119.4, 116.0, 115.9, 112.4, 112.0, 84.1, 81.4, 79.2, 69.4, 69.2, 49.8, 31.7, 30.8, 29.9, 29.7, 29.4, 29.3, 29.09, 29.07, 28.9, 28.59, 28.57, 27.1, 26.6, 26.3, 22.6, 14.0.

HR-ESI-MS m/z (%): 835.5080 (100, $[M]^{+}$) calcd. for $C_{58}H_{65}N_3O_2^{+}$: 835.5076.

UV-vis ($CHCl_3$): λ_{max} (ε) = 312 (104197), 390 (3912), 412 (8826), 438 (17233), 465 nm (21027 M^{-1} cm^{-1}).

P-1-Ant-C8:

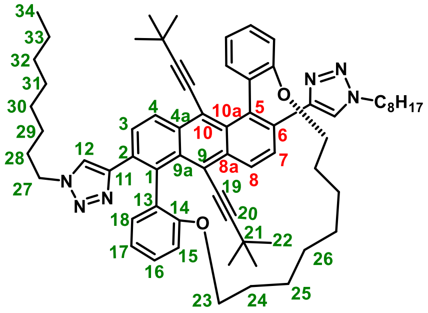

^{1}H NMR (400 MHz, $CDCl_3$) δ 8.83 (d, *J* = 9.4 Hz, 2H, H-C(3)), 8.47 (d, *J* = 9.4 Hz, 2H, H-C(4)), 7.50 (m, 2H, H-C(16)), 7.25 (dd, *J* = 7.4, 1.7 Hz, 2H, H-C(18)), 7.04 (m, 2H, H-C(17)), 6.97 (d, *J* = 7.8 Hz, 2H) , H-C(15), 5.75 (s, 2H, H-C(12)), 4.17 – 4.04 (m, 4H, H-C(27)), 3.90 (dt, *J* = 8.5, 4.2 Hz, 2H, H-C(23)), 3.79 (td, *J* = 8.7, 3.8 Hz, 2H, H-C(23)), 1.71 (m, 4H, H-C(28)), 1.31-1.25 (br, 20H, H-C(29) , H-C(30) , H-C(31) , H-C(32) , H-C(33)), 1.19 (m, 22H, H-C(22), H-C(24)), 0.91 (t, *J* = 6.9 Hz, 6H) , H-C(34), 0.67 – 0.44 (m, 8H, H-C(25) , H-C(26)).

^{13}C NMR (400 MHz, $CDCl_3$) δ 158.6 (C14), 146.7 (C2), 134.9 (C4a), 133.6 (C9a), 132.7 (C18), 131.7 (C13), 129.7 (C9), 129.39 (C16), 129.36 (C1), 127.9 (C3), 126.5 (C4), 121.9 (C12), 121.1 (C17), 119.5 (C11), 116.0 (C20), 112.6 (C15), 79.4 (C19), 69.4 (C23), 49.9 (C27), 31.7 (C29), 30.9 (C22), 29.9 (C28), 29.5 (C24), 29.3 (C26), 29.0 (C30), 28.9 (C31), 28.6 (C21), 26.8 (C25), 26.3 (C32), 22.6 (C33), 14.0 (C34).

HR-ESI-MS m/z (%): 990.6485 (100, $[M]^+$) calcd. for $C_{66}H_{82}N_6O_2^+$: 990.6499.

UV-vis ($CHCl_3$): λ_{max} (ε) = 318 (95418), 388 (3183), 410 (8866), 435 (16151), 463 nm (19416 M^{-1} cm^{-1}). $[\alpha]^{20}_D$ (CH_2Cl_2, *c* = 0.004) +310.

Synthesis of P-10-C6 and P-1-Ant-C6

Reaction of $CuSO_4{\cdot}5H_20$ (12.84 mg, 0.051 mmol) and of sodium ascorbate (13.60 mg, 0.068 mmol), ***P*-8-C6** (96 mg, 0.147 mmol), and 1-octylazide (22.85 mg, 0.147 mmol) furnished ***P*-8-C6** (12 mg), ***P*-10-C6** (59 mg, yield = 50%; m.p. 283-285 °C), and ***P*-1-Ant-C6** (20 mg, yield = 14%; m.p. 215-217 °C).

P-10-C6:

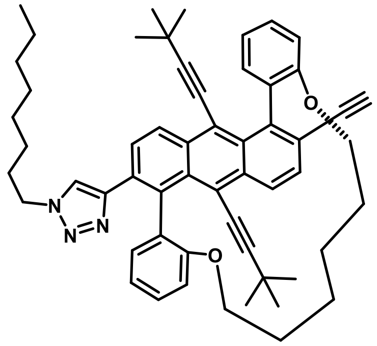

^{1}H NMR (400 MHz, Chloroform-*d*) δ 8.73 (d, *J* = 9.4 Hz, 1H), 8.57 (d, *J* = 9.1 Hz, 1H), 8.42 (d, *J* = 9.2 Hz, 1H), 7.73 (dd, *J* = 7.5, 1.7 Hz, 1H), 7.56 (d, *J* = 9.1 Hz, 1H), 7.43 (m, 2H), 7.32 (dd, *J* = 7.5, 1.7 Hz, 1H), 7.11 (td, *J* = 7.5, 1.1 Hz, 1H), 7.02 – 6.94 (m, 2H), 6.84 (dd, *J* = 8.2, 1.0 Hz, 1H), 5.75 (s, 1H, -N-C=C-H), 4.17 (dt, *J* = 14.1, 7.2 Hz, 1H), 4.04 (dt, *J* = 13.8, 7.0 Hz, 1H), 3.81 – 3.56 (m, 4H), 3.04 (s, 1H, -C≡C-H), 1.74 – 1.62 (m, 2H), 1.28 (br, 8H), 1.19 – 1.04 (br, 22H), 0.90 (t, *J* = 6.8 Hz, 3H), 0.80 – 0.67 (m, 1H), 0.54 – 0.21 (m, 4H).

^{13}C NMR (101 MHz, Chloroform-*d*) δ 158.5, 157.2, 146.6, 140.9, 134.2, 134.1, 133.4, 132.8, 132.6, 131.5, 130.6, 130.4, 130.0, 129.7, 129.3, 129.2, 129.1, 127.6, 127.0, 126.9, 122.3, 121.4, 121.2, 119.9, 119.1, 115.6, 115.3, 113.6, 112.0, 84.3, 81.6, 78.7, 78.1, 69.0, 68.2, 49.9, 31.7, 30.8, 29.9, 29.6, 29.3, 29.0, 28.9, 28.5, 28.4, 26.3, 25.5, 25.1, 22.6, 14.0.

HR-ESI-MS m/z (%): 807.4781 (100, $[M]^{+}$) calcd. for $C_{56}H_{61}N_3O_2^{+}$: 807.4764.

UV-vis ($CHCl_3$): λ_{max} (ε) = 313 (84741), 391 (3769), 413 (8171), 439 (15698), 467 nm (19132 M^{-1} cm^{-1}).

P-1-Ant-C6:

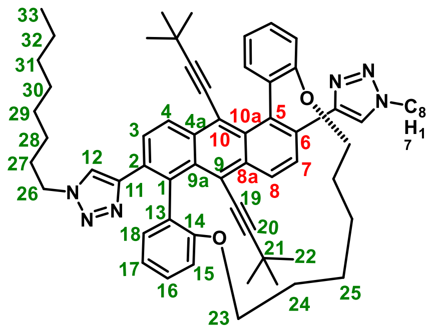

^{1}H NMR (400 MHz, $CDCl_3$) δ 8.75 (d, *J* = 9.4 Hz, 2H, H-C(3)), 8.41 (d, *J* = 9.4 Hz, 2H, H-C(4)), 7.47 (td, *J* = 7.8, 1.7 Hz, 2H, H-C(16)), 7.36 (dd, *J* = 7.5, 1.7 Hz, 2H, H-C(18)), 7.01 (m, 2H, H-C(17)), 6.96 (dd, *J* = 8.3, 1.1 Hz, 2H, H-C(15)), 5.7 (s, 2H, H-C(12)) 4.20 (dt, *J* = 14.1, 7.2 Hz, 2H, H-C(26)), 4.07 (dt, *J* = 13.7, 6.9 Hz, 2H, H-C(26)), 3.77 (m, 2H, H-C(23)), 3.68 (m, 2H, H-C(23)), 1.76 (m, 4H, H-C(26)), 1.27 (br, 20H, H-C(28), H-C(29) , H-C(30) , H-C(31) , H-C(32)), 1.14 – 1.06 (br, 22H, H-C(22) , H-C(24)), 0.90 (t, *J* = 6.9 Hz, 6H, H-C(33)), 0.344 – 0.29 (m, 4H, H-C(25)). $[\alpha]^{20}_{D}$ (CH_2Cl_2, c = 0.003) +750.

^{13}C NMR (101 MHz, $CDCl_3$) δ 158.5 (C14), 146.7 (C2), 134.3 (C4a), 133.4 (C9a), 132.8 (C18), 131.5 (C9), 130.2 (C13), 129.28 (C16), 129.25 (C1), 127.4 (C3), 126.8 (C4), 122.3 (C12), 121.3 (C17), 119.3 (C11), 115.7 (C20), 113.7 (C15), 78.7 (C19), 69.0 (C23), 49.2 (C26), 31.7 (C28), 30.8 (C22), 29.9 (C27), 29.5 (C24), 29.0 (C29), 28.9 (C30), 28.5 (C21), 26.3 (C31), 25.3 (C25), 22.6 (C32), 14.0 (C33).

HR-ESI-MS m/z (%): 962.6192 (100, $[M]^+$) calcd. for $C_{64}H_{78}N_6O_2^+$: 962.6186.

UV-vis ($CHCl_3$): λ_{max} (ε) = 313 (87589), 389 (3929), 411 (8882), 436 (15970), 464 nm (19069 M^{-1} cm^{-1}). $[\alpha]^{20}_{D}$ (CH_2Cl_2, c = 0.003) +470.

Synthesis of M-10-C6 and M-1-Ant-C6

Reaction of $CuSO_4{\cdot}5H_20$ (12.84 mg, 0.051 mmol) and of sodium ascorbate (13.60 mg, 0.068 mmol) with *M*-**8-C6** (96 mg, 0.147 mmol) and 1-octylazide (22.85 mg, 0.147mmol) furnished *M*-**8-C6** (12 mg), *M*-**10-C6** (59 mg, yield = 50%), and *M*-**1-Ant-C6** (20 mg, yield = 14%).

M-10-C6:

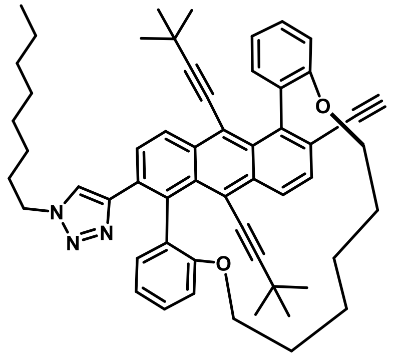

^{1}H NMR (400 MHz, Chloroform-*d*) δ 8.73 (d, *J* = 9.4 Hz, 1H), 8.57 (d, *J* = 9.1 Hz, 1H), 8.42 (d, *J* = 9.2 Hz, 1H), 7.73 (dd, *J* = 7.5, 1.7 Hz, 1H), 7.56 (d, *J* = 9.1 Hz, 1H), 7.43 (m, 2H), 7.32 (dd, *J* =

7.5, 1.7 Hz, 1H), 7.11 (td, *J* = 7.5, 1.1 Hz, 1H), 7.02 – 6.94 (m, 2H), 6.84 (dd, *J* = 8.2, 1.0 Hz, 1H), 5.75 (s, 1H, -N-C=C-H), 4.17 (dt, *J* = 14.1, 7.2 Hz, 1H), 4.04 (dt, *J* = 13.8, 7.0 Hz, 1H), 3.81 – 3.56 (m, 4H), 3.04 (s, 1H, -C≡C-H), 1.74 – 1.62 (m, 2H), 1.28 (br, 8H), 1.19 – 1.04 (br, 22H), 0.90 (t, *J* = 6.8 Hz, 3H), 0.80 – 0.67 (m, 1H), 0.54 – 0.21 (m, 4H).

^{13}C NMR (101 MHz, Chloroform-*d*) δ 158.5, 157.2, 146.6, 140.9, 134.2, 134.1, 133.4, 132.8, 132.6, 131.5, 130.6, 130.4, 130.0, 129.7, 129.3, 129.2, 129.1, 127.6, 127.0, 126.9, 122.3, 121.4, 121.2, 119.9, 119.1, 115.6, 115.3, 113.6, 112.0, 84.3, 81.6, 78.7, 78.1, 69.0, 68.2, 49.9, 31.7, 30.8, 29.9, 29.6, 29.3, 29.0, 28.9, 28.5, 28.4, 26.3, 25.5, 25.1, 22.6, 14.0.

HR-ESI-MS m/z (%): 807.4781 (100, $[M]^+$) calcd. for $C_{56}H_{61}N_3O_2^+$: 807.4764.

UV-vis ($CHCl_3$): λ_{max} (ε) = 313 (84741), 391 (3769), 413 (8171), 439 (15698), 467 nm (19132 M^{-1} cm^{-1}).

M-1-Ant-C6:

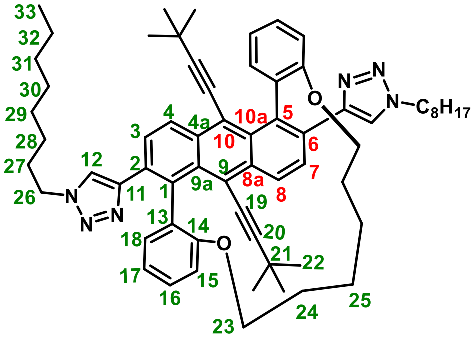

^{1}H NMR (400 MHz, $CDCl_3$) δ 8.75 (d, *J* = 9.4 Hz, 2H, H-C(3)), 8.41 (d, *J* = 9.4 Hz, 2H, H-C(4)), 7.47 (td, *J* = 7.8, 1.7 Hz, 2H, H-C(16)), 7.36 (dd, *J* = 7.5, 1.7 Hz, 2H, H-C(18)), 7.01 (m, 2H, H-C(17)), 6.96 (dd, *J* = 8.3, 1.1 Hz, 2H, H-C(15)), 5.7 (s, 2H, H-C(12)) 4.20 (dt, *J* = 14.1, 7.2 Hz, 2H, H-C(26)), 4.07 (dt, *J* = 13.7, 6.9 Hz, 2H, H-C(26)), 3.77 (m, 2H, H-C(23)), 3.68 (m, 2H, H-C(23)), 1.76 (m, 4H, H-C(26)), 1.27 (br, 20H, H-C(28), H-C(29) , H-C(30) , H-C(31) , H-C(32)), 1.14 – 1.06 (br, 22H, H-C(22) , H-C(24)), 0.90 (t, *J* = 6.9 Hz, 6H, H-C(33)), 0.344 – 0.29 (m, 4H, H-C(25)).

^{13}C NMR (101 MHz, $CDCl_3$) δ 158.5 (C14), 146.7 (C2), 134.3 (C4a), 133.4 (C9a), 132.8 (C18), 131.5 (C9), 130.2 (C13), 129.28 (C16), 129.25 (C1), 127.4 (C3), 126.8 (C4), 122.3 (C12), 121.3

(C17), 119.3 (C11), 115.7 (C20), 113.7 (C15), 78.7 (C19), 69.0 (C23), 49.2 (C26), 31.7 (C28), 30.8 (C22), 29.9 (C27), 29.5 (C24), 29.0 (C29), 28.9 (C30), 28.5 (C21), 26.3 (C31), 25.3 (C25), 22.6 (C32), 14.0 (C33).

HR-ESI-MS m/z (%): 962.6192 (100, $[M]^+$) calcd. for $C_{64}H_{78}N_6O_2^+$: 962.6186.

UV-vis ($CHCl_3$): λ_{max} (ε) = 313 (87589), 389 (3929), 411 (8882), 436 (15970), 464 nm (19069 M^{-1} cm^{-1}). $[\alpha]^{20}_D$ (CH_2Cl_2, c = 0.003) -430.

Synthesis of P-10-C4 and P-1-Ant-C4

Reaction of $CuSO_4 \cdot 5H_20$ (12.84 mg, 0.051 mmol) and of sodium ascorbate (13.60 mg, 0.068 mmol) with ***P*-8-C4** (92 mg, 0.147mmol) and 1-octylazide (22.85 mg, 0.147 mmol) furnished ***P*-8-C4** (15 mg; m.p. 231-233 °C), ***P*-10-C4** (50 mg, yield = 44%; m.p. 291-293 °C), and ***P*-1-Ant-C4** (25 mg, yield = 18%; m.p. 197-199 °C).

P-10-C4:

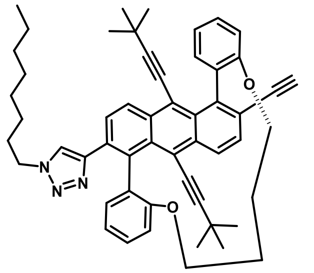

^{1}H NMR (400 MHz, Chloroform-*d*) δ 8.46 (d, *J* = 9.2 Hz, 1H), 8.34 (d, *J* = 9.0 Hz, 1H), 8.24 (d, *J* = 9.2 Hz, 1H), 7.98 (dd, *J* = 7.5, 1.7 Hz, 1H), 7.53 (d, *J* = 9.0 Hz, 1H), 7.48 (dd, *J* = 7.5, 1.7 Hz, 1H), 7.41 – 7.30 (m, 2H), 7.14 – 7.08 (m, 1H), 6.95 (td, *J* = 7.5, 1.0 Hz, 1H), 6.80 – 6.75 (m, 1H), 6.70 – 6.63 (m, 1H), 5.94 (s, 1H, -N-C=C-H), 4.26 – 4.14 (m, 1H), 4.11 – 4.00 (m, 1H), 3.63 (t, *J* = 11.5 Hz, 2H), 3.35 (dt, *J* = 32.8, 9.7 Hz, 2H), 3.09 (s, 1H, -C≡C-H), 1.76 – 1.66 (m, 2H), 1.30 (d, *J* = 12.6 Hz, 10H), 1.12 (d, *J* = 2.3 Hz, 18H), 0.94 – 0.86 (m, 3H), 0.82 – 0.69 (m, 2H), 0.57 (t, *J* = 13.4 Hz, 2H).

^{13}C NMR (101 MHz, Chloroform-*d*) δ 156.9, 155.8, 146.38, 139.8, 133.3, 133.0, 132.4, 132.2, 132.1, 131.5, 129.7, 129.3, 129.1, 129.0, 128.7, 127.3, 126.4, 125.9, 124.1, 123.8, 122.4, 120.2, 119.4, 118.7, 118.0, 114.3, 113.8, 111.3, 110.1, 84.2, 81.4, 66.6, 66.0, 49.9, 31.7, 30.77, 30.75, 30.0, 29.0, 28.9, 28.4, 28.3, 26.3, 26.1, 25.9, 22.6, 14.1.

HR-ESI-MS m/z (%): 779.4456 (100, [M]$^+$) calcd. for $C_{54}H_{57}N_3O_2^+$: 779.4451.

UV-vis ($CHCl_3$): λ_{max} (ε) = 316 (86658), 395 (3944), 418 (8562), 443 (15917), 472 nm (18831 M^{-1} cm^{-1}).

P-1-Ant-C4:

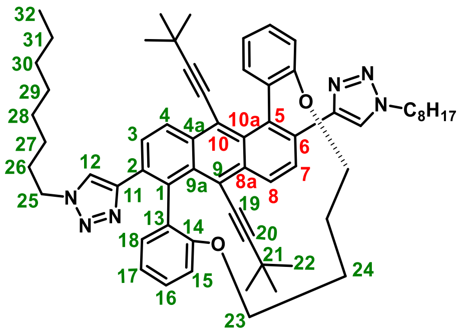

^{1}H NMR (400 MHz, $CDCl_3$) δ 8.48 (d, *J* = 9.2 Hz, 2H, H-C(3)), 8.24 (d, *J* = 9.2 Hz, 2H, H-C(4)), 7.51 (dd, *J* = 7.5, 1.7 Hz, 2H, H-C(18)), 7.36 (m, 2H, H-C(16)), 6.98 (td, *J* = 7.5, 0.9 Hz, 2H, H-C(17)), 6.78 (d, *J* = 7.9 Hz, 2H, H-C(15)), 4.95 (s, 2H, H-C(12)), 4.23 (dt, *J* = 14.0, 7.2 Hz, 2H, H-C(25)), 4.08 (dt, *J* = 13.8, 6.9 Hz, 2H, H-C(25)), 3.61 (m, 2H, H-C(23)), 3.43(m, 2H, H-C(23)), 1.75 (tt, *J* = 13.4, 6.8 Hz, 4H, H-C(26)), 1.33 – 1.17 (m, 20H, H-C(27), H-C(28) H-C(29) H-C(30) H-C(31)), 1.13 (m, 18H, H-C(22)), 0.90 (t, *J* = 6.9 Hz, 6H, H-C(32)), 0.74 – 0.54 (m, 4H, H-C(24)).

^{13}C NMR (101 MHz, $CDCl_3$) δ 156.9 (C14), 146.4 (C2), 133.5 (C4a), 132.9 (C9a), 132.5 (C18), 131.9 (C9), 129.9 (C13), 129.0 (C16), 28.4 (C1), 127.0 (C3), 126.3 (C4), 122.3 (C12), 120.3 (C17), 118.1 (C11), 114.2 (C20), 111.5 (C15), 77.3 (C19), 66.8 (C23), 49.9 (C25), 31.7 (C27), 30.7 (C22), 30.0 (C26), 29.0 (C28), 28.9 (C29), 28.4 (C21), 26.3 (C30), 26.0 (C24), 22.6 (C31), 14.0 (C32).

HR-ESI-MS m/z (%): 934.5874 (100, [M]$^+$) calcd. for $C_{62}H_{74}N_6O_2^+$: 934.5873.

UV-vis ($CHCl_3$): λ_{max} (ε) = 320 (82529), 394 (4168), 414 (9260), 439 (16261), 467 nm (18867 M^{-1} cm^{-1}). $[\alpha]^{20}_D$ (CH_2Cl_2, c = 0.005) +550.

S2.4 General method for synthesis of 2-Ant-Cns

Synthesis of P,P-2-Ant-C8

To a two necked RB flask kept under positive Ar flow were added *P*-**10-C8** (16 mg, 0.019 mmol), 1,4-diiodobenzene (3 mg, 0.009 mmol), $Pd(PPh_3)_4$ (0.10 mg, 0.00009 mmol) and CuI (0.03mg, 0.00018 mmol). Triethylamine/THF (2 mL / 1 mL) was purged with Ar for 5 min and then added to the previously mixed reagents and the reaction mixture was stirred at 65 °C for 22 h until complete consumption of *P*-**10-C8** was observed by TLC. The solvent was evaporated and the reaction mixture was directly loaded on a silica gel column and eluted with EtOAc/hexane (1:5) to afford *P*,*P*-**2-Ant-C8** as a yellow solid (14 mg, yield = 90%; m.p. 216-218 °C) with green emission under a 365 nm lamp.

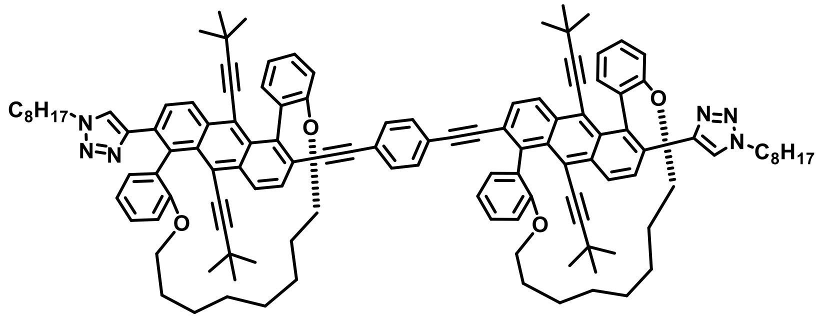

^1H NMR (500 MHz, $CDCl_3$) δ 8.82 (d, *J* = 9.4 Hz, 2H), 8.68 (d, *J* = 9.2 Hz, 2H), 8.47 (d, *J* = 9.4 Hz, 2H), 7.59 (d, *J* = 9.2 Hz, 2H), 7.52 – 7.44 (m, 6H), 7.22 (dd, *J* = 7.4, 1.6 Hz, 2H), 7.12 (t, *J* = 7.4 Hz, 2H), 7.03 (dd, *J* = 12.2, 4.7 Hz, 2H), 6.99 (s, 4H), 6.96 (t, *J* = 8.2 Hz, 4H), 5.75 (s, 2H), 4.11 (dq, *J* = 13.6, 6.8 Hz, 4H), 3.96 (dt, *J* = 8.6, 4.2 Hz, 2H), 3.87 – 3.75 (m, 6H), 1.72 – 1.64 (m, 4H), 1.32 1.16 (br, m, *J* = 1.4 Hz, 20H), 0.90 (t, *J* = 7.0 Hz, 6H), 0.77 – 0.33 (m, 10H).

^{13}C NMR (126 MHz, $CDCl_3$) δ 158.5, 158.0, 146.7, 141.0, 134.8, 134.7, 133.6, 132.7, 132.5, 131.7, 131.6, 131.1, 130.0, 129.9, 129.8, 129.7, 129.6, 129.3, 129.0, 128.09, 128.04, 127.4, 126.7, 123.0, 122.6, 121.9, 121.1, 120.0, 119.8, 119.4, 115.9, 112.4, 112.0, 94.3, 92.6, 79.3, 78.9, 69.5, 69.2, 49.9, 31.7, 30.9, 29.9, 29.8, 29.77, 29.70, 29.6, 29.59, 29.51, 29.36, 29.33, 29.31, 29.1, 29.0, 28.9, 28.6, 28.59, 27.2, 27.1, 26.6, 26.3, 22.69, 22.63, 14.11, 14.0.

MS (MALDI-TOF, m/z): calcd for $C_{122}H_{132}N_6O_4$, 1746.034; found 1746.037.

UV-vis ($CHCl_3$): λ_{max} (ε) = 288 (50593), 347 (84146), 369 (63519), 387 (68300), 424 (24677), 448 (31196), 477 nm (33413 M^{-1} cm^{-1}). $[\alpha]^{20}_D$ (CH_2Cl_2, c = 0.003) +750.

Synthesis of P,P-2-Ant-C6

Reaction of ***P*-10-C6** (21 mg, 0.026 mmol), 1,4-diiodobenzene (3.95 mg, 0.012 mmol), $Pd(PPh_3)_4$ (0.14 mg, 0.00012 mmol), and CuI (0.046mg, 0.00024 mmol) in triethylamine/THF (2 mL:1 mL) afforded ***P*,*P*-2-Ant-C6** as a yellow solid (10 mg, yield = 49%; m.p. 211-213 °C) with green emission under a 365 nm lamp.

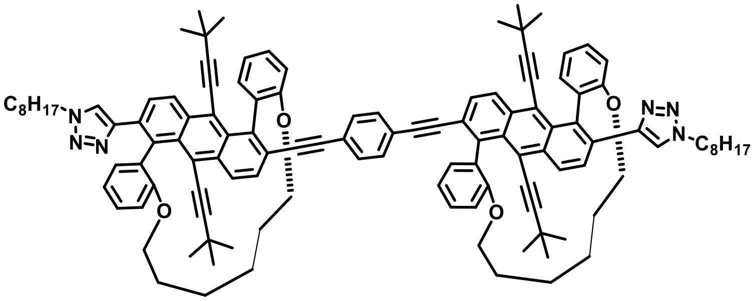

^{1}H NMR (500 MHz, $C_2D_2Cl_4$) δ 8.74 (d, *J* = 9.4 Hz, 2H), 8.61 (d, *J* = 9.3 Hz, 2H), 8.40 (d, *J* = 9.3 Hz, 2H), 7.81 (d, *J* = 7.4 Hz, 2H), 7.65 (d, *J* = 9.1 Hz, 2H), 7.51 (t, *J* = 7.8 Hz, 4H), 7.32 (d, *J* = 7.3 Hz, 2H), 7.21 (t, *J* = 7.5 Hz, 2H), 7.15 (s, 4H), 7.02 (dd, *J* = 17.8, 7.8 Hz, 4H), 6.89 (d, *J* = 8.1 Hz, 2H), 5.75 (s, 2H, -N-C=C-H), 4.20 (dt, *J* = 14.0, 7.1 Hz, 2H), 4.07 (dt, *J* = 14.0, 7.2 Hz, 2H), 3.79 – 3.65 (m, 8H), 1.71 (m, 4H), 1.38 – 1.27 (br, 20H), 1.18 – 1.12 (br, 44H), 0.92 (t, *J* = 6.9 Hz, 6H), 0.49 (m, 4H), 0.4 – 0.20 (m, 4H).

^{13}C NMR (126 MHz, $C_2D_2Cl_4$) δ 158.8, 157.8, 146.9, 141.0, 134.6, 134.4, 134.3, 133.2, 133.0, 131.7, 131.6, 131.1, 130.0, 129.7, 129.0, 129.0, 128.0, 127.6, 127.3, 123.5, 123.0, 122.5, 121.8, 120.3, 120.2 (s), 119.7, 116.5, 116.0, 114.2 (s), 112.7, 94.6, 93.8, 79.0, 78.6, 74.6, 69.4, 68.7, 50.4, 32.4, 32.2, 31.32, 31.30, 30.3, 30.2, 30.1, 39.9, 29.8, 29.5, 29.4, 28.94, 28.91, 26.8, 26.1, 25.7, 23.2, 23.1, 14.78, 14.74.

MS (MALDI-TOF, m/z): calcd for $C_{118}H_{124}N_6O_4$, 1689.971; found 1689.984.

UV-vis ($CHCl_3$): λ_{max} (ε) = 290 (41690), 347 (78842), 370 (60201), 388 (60114), 426 (23843), 449 (28385, 479 nm (29915 M^{-1} cm^{-1}). $[\alpha]^{20}_D$ (CH_2Cl_2, c = 0.004) +1150.

Synthesis of M,M-2-Ant-C6

Reaction of *M*-**10-C6** (21 mg, 0.026 mmol), 1,4-diiodobenzene (3.95 mg, 0.012 mmol), $Pd(PPh_3)_4$ (0.14 mg, 0.00012 mmol), and CuI (0.046mg, 0.00024 mmol) in triethylamine/THF (2 mL:1 mL) afforded *M*,*M*-**2-Ant-C6** as a yellow solid (10 mg, yield = 49%) with green emission under 365 nm lamp.

^{1}H NMR (500 MHz, $C_2D_2Cl_4$) δ 8.74 (d, *J* = 9.4 Hz, 2H), 8.61 (d, *J* = 9.3 Hz, 2H), 8.40 (d, *J* = 9.3 Hz, 2H), 7.81 (d, *J* = 7.4 Hz, 2H), 7.65 (d, *J* = 9.1 Hz, 2H), 7.51 (t, *J* = 7.8 Hz, 4H), 7.32 (d, *J* = 7.3 Hz, 2H), 7.21 (t, *J* = 7.5 Hz, 2H), 7.15 (s, 4H), 7.02 (dd, *J* = 17.8, 7.8 Hz, 4H), 6.89 (d, *J* = 8.1 Hz, 2H), 5.75 (s, 2H, -N-C=C-H), 4.20 (dt, *J* = 14.0, 7.1 Hz, 2H), 4.07 (dt, *J* = 14.0, 7.2 Hz, 2H), 3.79 – 3.65 (m, 8H), 1.71 (m, 4H), 1.38 – 1.27 (br, 20H), 1.18 – 1.12 (br, 44H), 0.92 (t, *J* = 6.9 Hz, 6H), 0.49 (m, 4H), 0.4 – 0.20 (m, 4H).

^{13}C NMR (126 MHz, $C_2D_2Cl_4$) δ 158.8, 157.8, 146.9, 141.0, 134.6, 134.4, 134.3, 133.2, 133.0, 131.7, 131.6, 131.1, 130.0, 129.7, 129.0, 129.0, 128.0, 127.6, 127.3, 123.5, 123.0, 122.5, 121.8, 120.3, 120.2 (s), 119.7, 116.5, 116.0, 114.2 (s), 112.7, 94.6, 93.8, 79.0, 78.6, 74.6, 69.4, 68.7, 50.4, 32.4, 32.2, 31.32, 31.30, 30.3, 30.2, 30.1, 39.9, 29.8, 29.5, 29.4, 28.94, 28.91, 26.8, 26.1, 25.7, 23.2, 23.1, 14.78, 14.74.

MS (MALDI-TOF, m/z): calcd for $C_{118}H_{124}N_6O_4$, 1689.971; found 1689.984.

UV-vis ($CHCl_3$): λ_{max} (ε) = 290 (41690), 347 (78842), 370 (60201), 388 (60114), 426 (23843), 449 (28385, 479 nm (29915 M^{-1} cm^{-1}). $[\alpha]^{20}_D$ (CH_2Cl_2, c = 0.004) -1250.

Synthesis of P,P-2-Ant-C4

Reaction of *P*-**10-C4** (16 mg, 0.020 mmol), 1,4-diiodobenzene (3.13 mg, 0.0095 mmol), $Pd(PPh_3)_4$ (0.11 mg, 0.000095 mmol), and CuI (0.036mg, 0.00019 mmol) in triethylamine/THF (2 mL:2 mL) afforded *P*,*P*-**2-Ant-C4** as a yellow solid (14 mg, yield = 90%; m.p. 206-208 °C) with green emission under a 365 nm lamp.

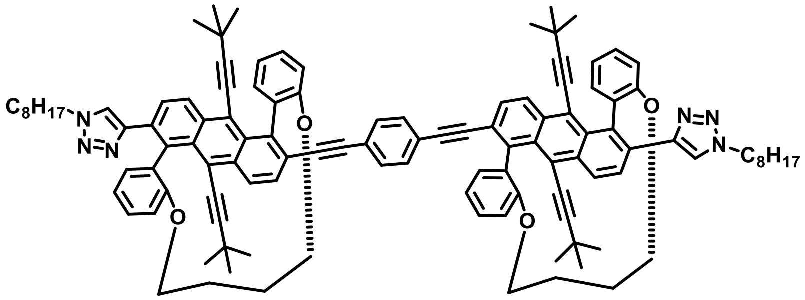

^{1}H NMR (500 MHz, $CDCl_3$) δ 8.48 (d, *J* = 9.0 Hz, 2H), 8.37 (d, *J* = 8.8 Hz, 2H), 8.25 (d, *J* = 9.1 Hz, 2H), 8.06 (d, *J* = 7.5 Hz, 2H), 7.58 (d, *J* = 8.9 Hz, 2H), 7.49 (d, *J* = 7.4 Hz, 2H), 7.40 (m, 4H), 7.24 (s, 4H), 7.21 (t, *J* = 7.5 Hz, 2H), 6.97 (t, *J* = 7.5 Hz, 2H), 6.79 (d, *J* = 8.2 Hz, 2H), 6.70 (d, *J* = 8.2 Hz, 2H), 5.94 (s, 2H), 4.24 (dt, *J* = 13.8, 6.9 Hz, 2H), 4.08 (dt, *J* = 13.9, 6.9 Hz, 2H), 3.67 (br, 4H), 3.42 – 3.30 (m, 4H), 1.75 – 1.65 (m, 4H), 1.32 – 1.30 (br, 20H), 1.14 – 1.13 (br, 36H), 0.92 (t, *J* = 6.6 Hz, 6H), 0.77 – 0.57 (m, 8H).

^{13}C NMR (126 MHz, $CDCl_3$) δ 156.9, 156.0, 146.4, 139.2, 133.3, 133.1, 133.0, 132.4, 132.3, 132.2, 131.7, 131.1, 129.7, 129.07, 129.0, 128.7, 128.5, 127.3, 126.4, 125.9, 123.2, 122.4, 121.2, 120.2, 119.2, 118.6, 118.0, 114.2, 113.8, 111.3, 110.2, 93.7, 92.9, 77.3, 66.6, 66.0, 49.9, 31.7, 30.7, 30.0, 29.6, 29.0, 28.9, 28.43, 28.40, 26.3, 26.1, 25.9, 22.6, 14.1, 14.0.

MS (MALDI-TOF, m/z): calcd for $C_{114}H_{116}N_6O_4$, 1633.909; found 1633.899.

UV-vis ($CHCl_3$): λ_{max} (ε) 349 (76144), 372 (55165), 390 (57391), 431 (21885), 454 (26421), 484 nm (27245 M^{-1} cm^{-1}). $[\alpha]^{20}_D$ (CH_2Cl_2, c = 0.005) +1420.

S2.5 General method for synthesis of the 3-Ant-Cn series

Synthesis of P,P,P-3-Ant-C8

To a two necked RB flask kept under positive Ar flow were added *P*-**10-C8** (16mg, 0.019 mmol), *P*-**9-C8** (9.81 mg, 0.009 mmol), $Pd(PPh_3)_4$ (0.10 mg, 0.00009 mmol), and CuI (0.03 mg, 0.00018 mmol). Triethylamine/THF (3 mL:1 mL) was purged with Ar for 5 min and then added to the previously mixed reagents and the reaction mixture was stirred at 65 °C for 22 h until complete consumption of *P*-**10-C8** was observed by TLC. The solvent was evaporated and the reaction mixture was directly loaded on a silica gel column and eluted with EtOAc/hexane (1:5) to afford *P,P,P*-**3-Ant-C8** as a deep orange solid (13 mg, yield = 58%; m.p. 261-263 °C) with green emission under a 365 nm lamp.

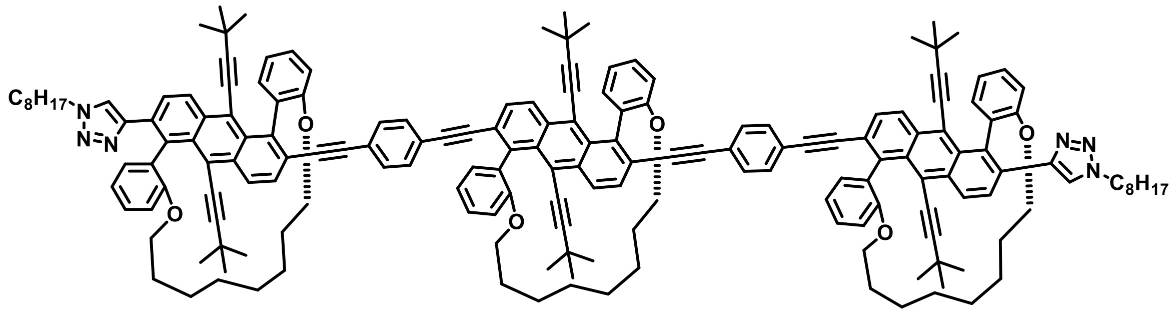

^{1}H NMR (500 MHz, $CDCl_3$) δ 8.83 (d, *J* = 9.3 Hz, 2H), 8.70 (dd, *J* = 9.2, 1.2 Hz, 4H), 8.48 (d, *J* = 9.4 Hz, 2H), 7.61 (dd, *J* = 9.1, 5.8 Hz, 4H), 7.52 – 7.42 (m, 10H), 7.23 (dd, *J* = 7.4, 1.7 Hz, 2H), 7.14 (tdd, *J* = 7.4, 6.3, 1.1 Hz, 4H), 7.03 (td, *J* = 7.4, 1.0 Hz, 2H), 6.99 – 6.94 (m, 12H), 5.75 (s, 2H), 4.16 – 4.07 (hept, *J* = 6.9 Hz, 4H), 3.97 – 3.92 (tt, *J* = 9.0, 4.4 Hz, 4H), 3.85 – 3.76 (m, 8H), 1.71 (p, *J* = 7.1 Hz, 5H), 1.32 – 1.26 (br, 20H), 1.17 – 1.03 (m, 66H), 0.89 (d, *J* = 7.1 Hz, 6H), 0.76 – 0.39 (m, 24H).

^{13}C NMR (126 MHz, $CDCl_3$) δ 158.5, 158.0, 146.7, 141.06, 141.04, 134.8, 134.7, 134.6, 133.6, 132.7, 132.57, 132.51, 131.76, 131.72, 131.6, 131.2, 131.1, 130.0, 129.8, 129.7, 129.6, 129.3, 129.0, 128.3, 128.0, 127.58, 127.50, 126.7, 123.1, 123.0, 122.6, 121.9, 121.1, 120.05, 120.02, 119.8, 119.6, 119.4, 115.9, 115.8, 112.4, 112.0, 111.9, 94.5, 94.3, 92.6, 92.5, 79.3, 78.9, 78.8, 69.5, 69.3, 69.2, 49.9, 31.7, 30.9, 30.8, 29.9, 29.8, 29.7, 29.59, 29.51, 29.36, 29.33, 29.2, 29.1, 29.0, 28.9, 28.6, 28.5, 27.1, 26.9, 26.6, 26.3, 22.69, 22.63, 14.1, 14.0.

MS (MALDI-TOF, m/z): calcd for $C_{178}H_{182}N_6O_4$, 2500.415; found 2500.357.

UV-vis ($CHCl_3$): λ_{max} (ε) = 282 (70912), 317 (67777), 354 (141017), 373 (128981), 391 (142402), 449 (52494), 479 nm (53469 M^{-1} cm^{-1}). $[\alpha]^{20}_D$ (CH_2Cl_2, c = 0.002) +2310.

Synthesis of P,P,P-3-Ant-C6

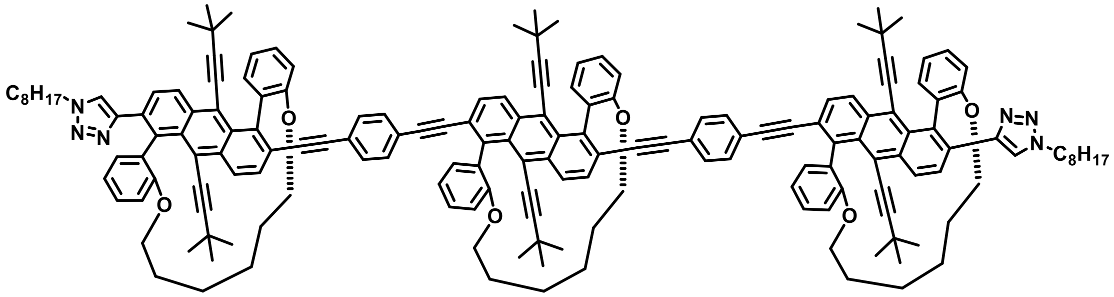

Reaction of *P*-**10-C6** (21 mg, 0.026 mmol), *P*-**9-C6** (12.68 mg, 0.012 mmol), $Pd(PPh_3)_4$ (0.14 mg, 0.00012 mmol), CuI (0.046mg, 0.00024 mmol), and triethylamine/THF (3 mL:1.5 mL) furnished *P,P,P*-**3-Ant-C6** as a deep orange solid (7 mg, yield = 24%; m.p. 299-301 °C) with green emission under a 365 nm lamp.

^{1}H NMR (500 MHz, Chloroform-*d*) δ 8.75 (d, *J* = 9.4 Hz, 2H), 8.62 (dd, *J* = 9.1, 2.2 Hz, 4H), 8.44 (d, *J* = 9.3 Hz, 2H), 7.80 (ddd, *J* = 19.3, 7.4, 1.7 Hz, 4H), 7.6 (dd, *J* = 9.1, 7.4 Hz, 4H), 7.48 – 7.43 (m, 8H), 7.34 (dd, *J* = 7.6, 1.7 Hz, 2H), 7.18 – 7.14 (m, 4H), 7.09 (s, 8H), 7.01 (dd, *J* = 17.9, 8.0 Hz, 4H), 6.89 (t, *J* = 7.9 Hz, 4H), 5.76 (s, 2H), 4.20 (dt, *J* = 14.1, 7.1 Hz, 2H), 4.08 (dd, *J* = 13.8, 6.9 Hz, 2H), 3.78 – 3.62 (m, 12H), 1.73 (m, 4H), 1.28 (br, 20H), 1.14 (br, m, 66H), 0.92 (s, 6H), 0.51 – 0.39 (m, 12H).

^{13}C NMR (126 MHz, Chloroform-d) δ 158.5, 157.58, 157.51, 146.7, 140.4, 140.3, 134.2, 134.1, 133.9, 133.5, 132.89, 132.83, 131.6, 131.19, 131.15, 131.11, 131.0, 130.4, 130.2, 129.3, 129.1, 128.7, 128.5, 127.6, 127.0, 123.2, 123.1, 122.7, 122.3, 121.4, 119.86, 119.83, 119.5, 119.1, 115.5, 115.2, 115.0, 113.7, 112.2, 112.1, 94.5, 94.2, 92.9, 92.2, 78.3, 69.0, 68.1, 49.9, 30.8, 30.4, 30.0, 29.9, 29.6, 29.5, 29.4, 29.3, 29.0, 28.9, 28.54, 28.50, 27.0, 26.3, 25.5, 25.4, 25.2, 22.64, 14.0.

MS (MALDI-TOF, m/z): calcd for $C_{178}H_{182}N_6O_4$, 2417.329; found 2417.356.

UV-vis ($CHCl_3$): λ_{max} (ε) = 285 (64532), 320 (70746), 354 (132601), 373 (124249), 391 (118082), 450 (45674), 480 nm (45802 M^{-1} cm^{-1}). $[\alpha]^{20}_D$ (CH_2Cl_2, c = 0.001) +3630.

Synthesis of M,M,M-3-Ant-C6

Reaction of *M*-**10-C6** (21 mg, 0.026 mmol), *M*-**9-C6** (12.68 mg, 0.012 mmol), $Pd(PPh_3)_4$ (0.14 mg, 0.00012 mmol), CuI (0.046mg, 0.00024 mmol), and triethylamine/THF (3 mL:1.5 mL) furnished *M,M,M*-**3-Ant-C6** as a deep orange solid (7 mg, yield = 24%) with green emission under a 365 nm lamp.

^{1}H NMR (500 MHz, Chloroform-*d*) δ 8.75 (d, *J* = 9.4 Hz, 2H), 8.62 (dd, *J* = 9.1, 2.2 Hz, 4H), 8.44 (d, *J* = 9.3 Hz, 2H), 7.80 (ddd, *J* = 19.3, 7.4, 1.7 Hz, 4H), 7.6 (dd, *J* = 9.1, 7.4 Hz, 4H), 7.48 – 7.43 (m, 8H), 7.34 (dd, *J* = 7.6, 1.7 Hz, 2H), 7.18 – 7.14 (m, 4H), 7.09 (s, 8H), 7.01 (dd, *J* = 17.9, 8.0 Hz, 4H), 6.89 (t, *J* = 7.9 Hz, 4H), 5.76 (s, 2H), 4.20 (dt, *J* = 14.1, 7.1 Hz, 2H), 4.08 (dd, *J* = 13.8, 6.9 Hz, 2H), 3.78 – 3.62 (m, 12H), 1.73 (m, 4H), 1.28 (br, 20H), 1.14 (br, m, 66H), 0.92 (s, 6H), 0.51 – 0.39 (m, 12H).
^{13}C NMR (126 MHz, Chloroform-d) δ 158.5, 157.58, 157.51, 146.7, 140.4, 140.3, 134.2, 134.1, 133.9, 133.5, 132.89, 132.83, 131.6, 131.19, 131.15, 131.11, 131.0, 130.4, 130.2, 129.3, 129.1, 128.7, 128.5, 127.6, 127.0, 123.2, 123.1, 122.7, 122.3, 121.4, 119.86, 119.83, 119.5, 119.1, 115.5, 115.2, 115.0, 113.7, 112.2, 112.1, 94.5, 94.2, 92.9, 92.2, 78.3, 69.0, 68.1, 49.9, 30.8, 30.4, 30.0, 29.9, 29.6, 29.5, 29.4, 29.3, 29.0, 28.9, 28.54, 28.50, 27.0, 26.3, 25.5, 25.4, 25.2, 22.64, 14.0.

UV-vis ($CHCl_3$): λ_{max} (ε) = 285 (64532), 320 (70746), 354 (132601), 373 (124249), 391 (118082), 450 (45674), 480 nm (45802 M^{-1} cm^{-1}). $[\alpha]^{20}_D$ (CH_2Cl_2, c = 0.001) +3410.

Synthesis of P,P,P-Ant-C4

Reaction of *P*-**10-C4** (16 mg, 0.020 mmol), *P*-**9-C4** (3.13 mg, 0.0095 mmol), $Pd(PPh_3)_4$ (0.11 mg, 0.000095 mmol), CuI (0.036mg, 0.00019 mmol), and triethylamine/THF (2 mL:2 mL) furnished *P,P,P*-**3-Ant-C4** as a deep orange solid (13 mg, yield = 59%; m.p. 285-287 °C) with green emission under a 365 nm lamp.

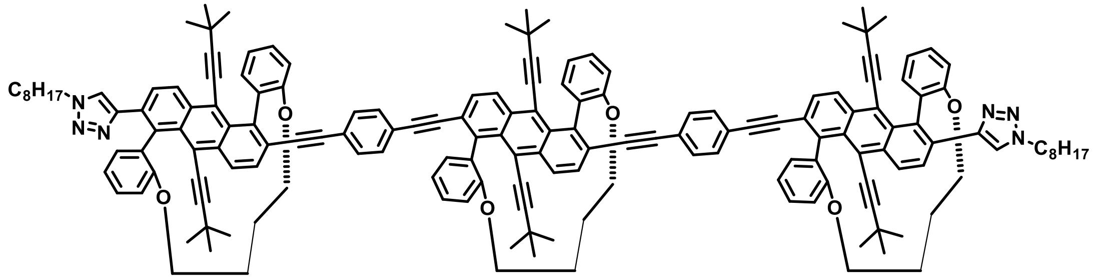

^{1}H NMR (500 MHz, $CDCl_3$) δ 8.49 (d, *J* = 9.2 Hz, 2H), 8.37 (d, *J* = 9.0 Hz, 4H), 8.25 (d, *J* = 9.2 Hz, 2H), 8.06 (ddd, *J* = 7.5, 3.7, 1.7 Hz, 4H), 7.59 (dd, *J* = 8.9, 8.2 Hz, 4H), 7.49 (dd, *J* = 7.5, 1.7 Hz, 2H), 7.40 – 7.36 (m, 6H), 7.25 (s, 8H), 7.20 (dd, *J* = 7.9, 7.0 Hz, 4H), 6.96 (td, *J* = 7.5, 0.9 Hz, 2H), 6.79 (d, *J* = 7.8 Hz, 2H), 6.71 (dd, *J* = 7.8, 4.3 Hz, 4H), 5.95 (s, 2H), 4.24 – 4.17 (m, 2H), 4.08 (dt, *J* = 13.7, 7.0 Hz, 2H), 3.68 (m, 6H), 3.42 (m, 6H), 1.75 (m, 4H), 1.33 – 1.28 (m, 20H), 1.13 (m, 54H), 0.91 (t, *J* = 7.0 Hz, 6H), 0.77 – 0.60 (m, 12H).

^{13}C NMR (126 MHz, $CDCl_3$) δ 156.9, 156.0, 155.9, 146.4, 139.2, 133.3, 133.1, 133.0, 132.9, 132.4, 132.3, 132.25, 132.21, 132.0, 131.7, 131.1, 129.7, 129.6, 129.07, 129.0, 128.7, 128.5, 127.3, 126.4, 126.1, 125.9, 123.3, 123.2, 122.4, 121.5, 121.2, 120.2, 119.28, 119.21, 118.6, 118.5, 118.0, 114.2, 113.8, 113.7, 111.3, 110.2, 110.1, 93.9, 93.7, 92.9, 92.8, 77.3, 66.6, 66.0, 65.9, 49.9, 31.7, 30.7, 30.0, 29.73, 29.73, 29.0, 28.9, 28.43, 28.40, 26.3, 26.1, 25.9, 22.69, 22.64, 14.1, 14.0.

MS (MALDI-TOF, m/z): calcd for $C_{166}H_{158}N_6O_4$, 2332.296; found 2332.247.

UV-vis ($CHCl_3$): λ_{max} (ε) = 285 (57310), 324 (63035), 356 (118870), 375 (103268), 394 (106266), 455 (39549), 485 nm (38946 M^{-1} cm^{-1}). $[\alpha]^{20}_{D}$ (CH_2Cl_2, c = 0.002) +4810.

S3 Characterization

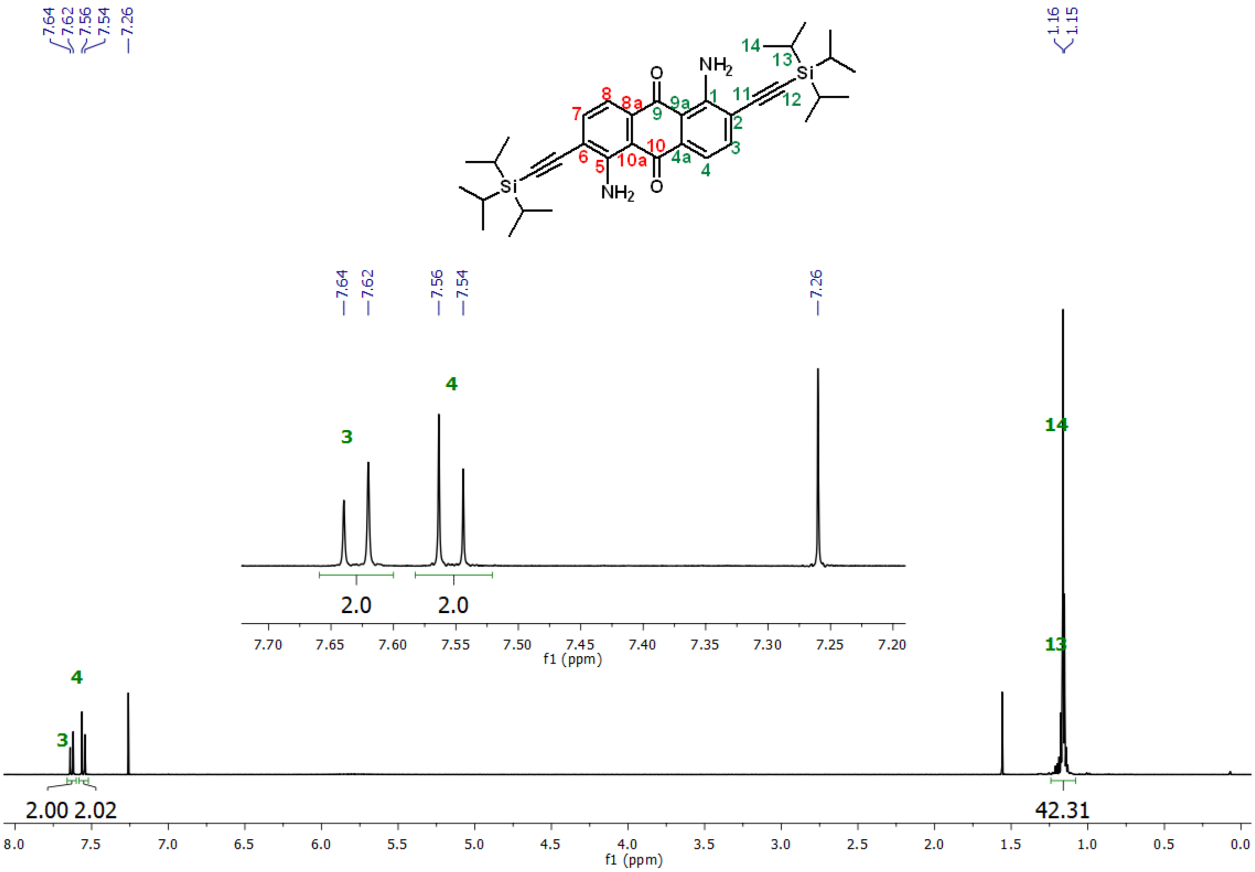

Supplementary Figure 2. ^{1}H NMR (400 MHz) of **2** in $CDCl_3$, measured at 298 K.

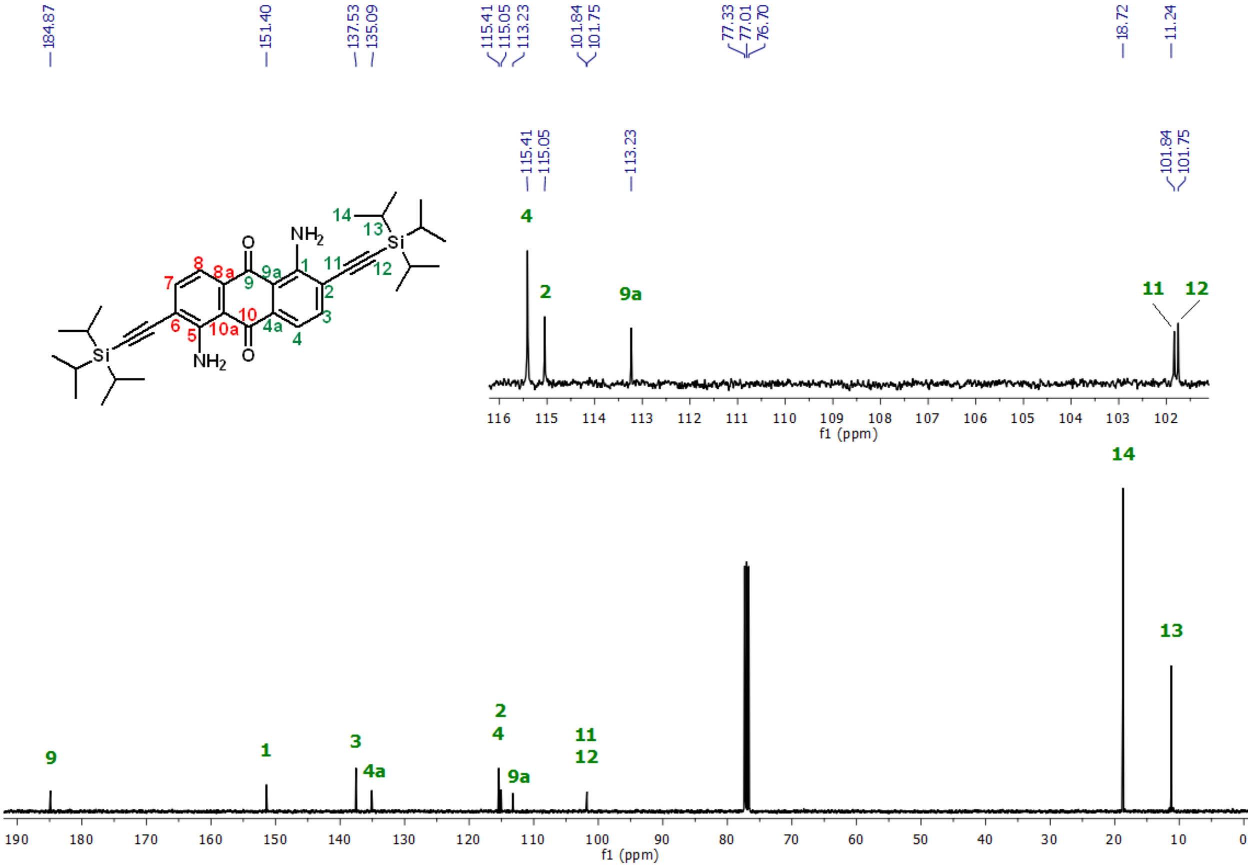

Supplementary Figure 3. ^{13}C NMR (101 MHz) of **2** in $CDCl_3$, measured at 298 K.

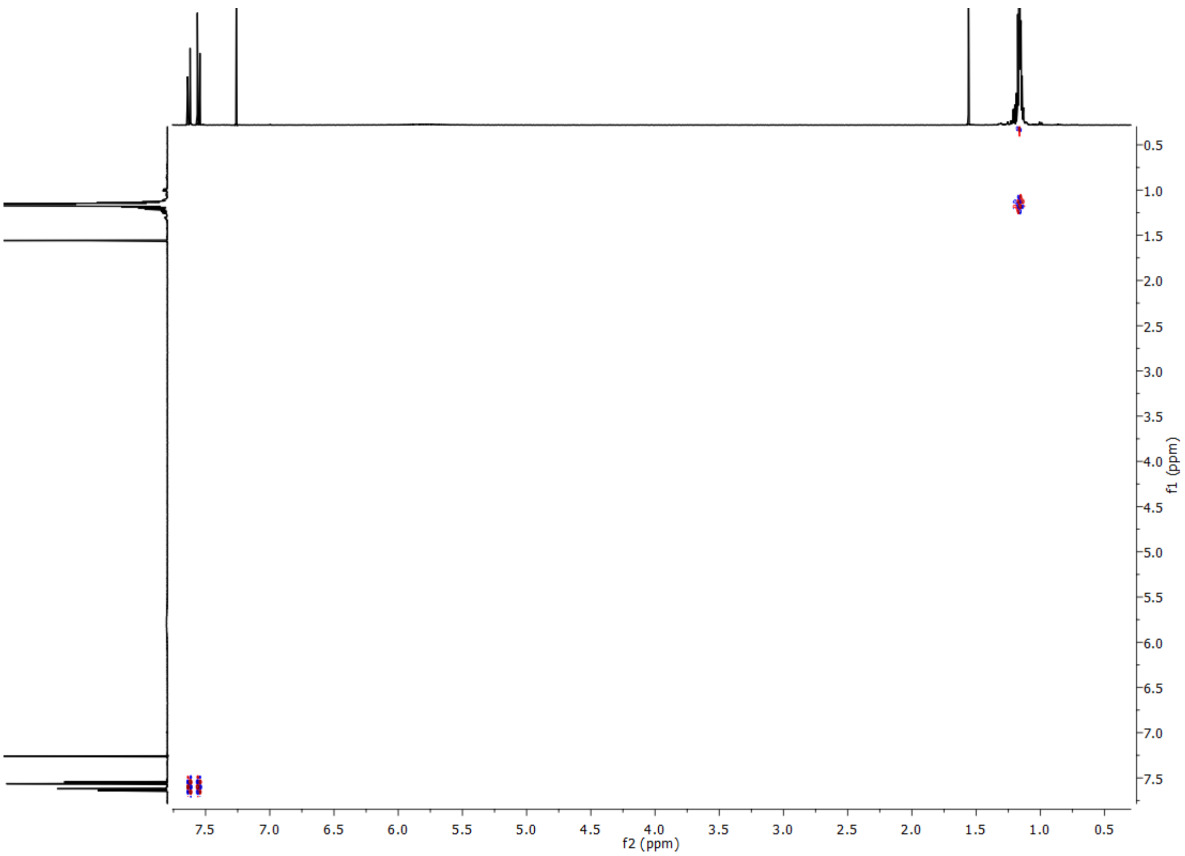

Supplementary Figure 4. COSY NMR (400 MHz) of **2** in $CDCl_3$, measured at 298 K.

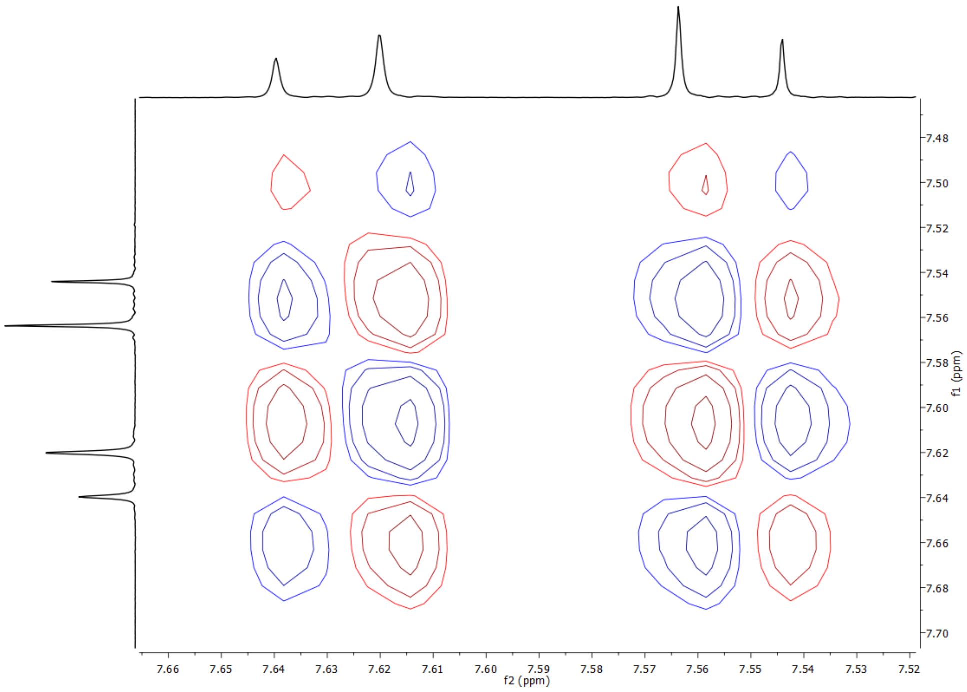

Supplementary Figure 5. COSY NMR (400 MHz) of **2** in $CDCl_3$, measured at 298 K (expansion in aromatic region).

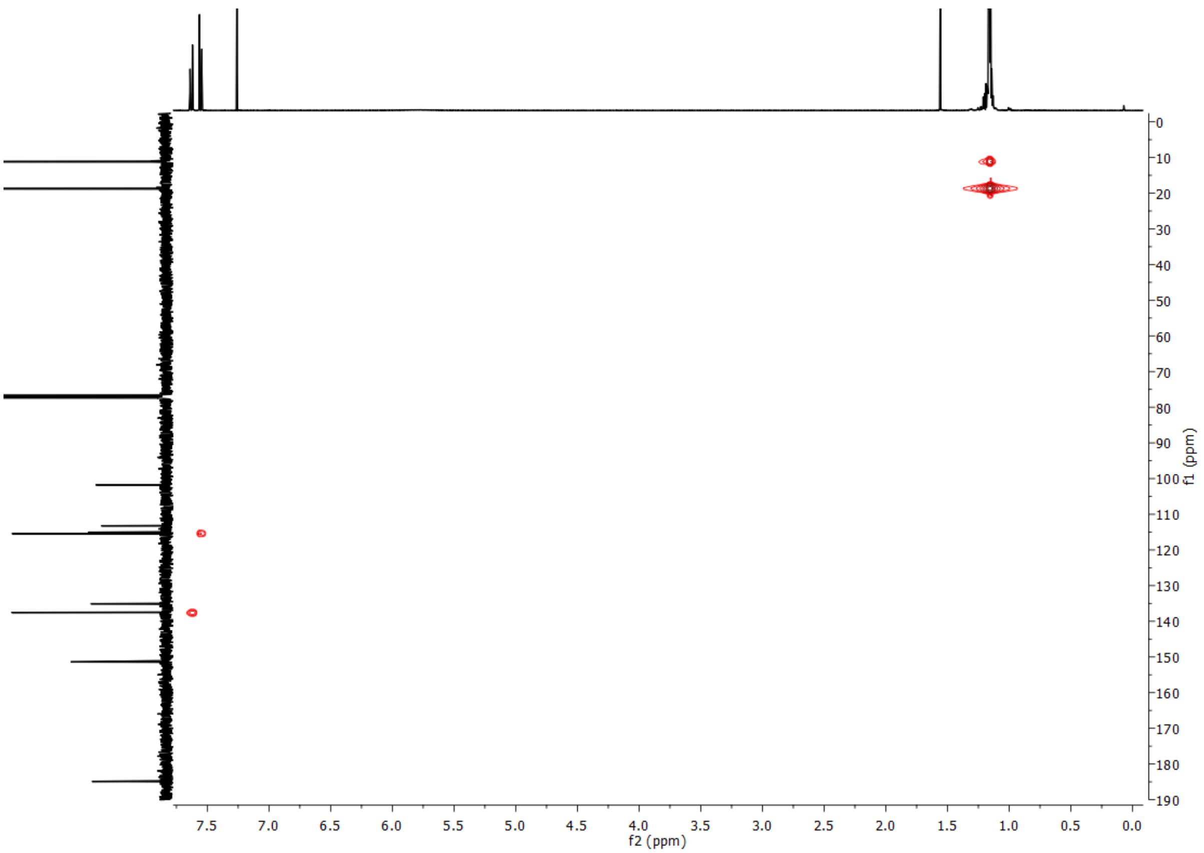

Supplementary Figure 6. HSQC NMR (400 MHz) of **2** in $CDCl_3$, measured at 298 K.

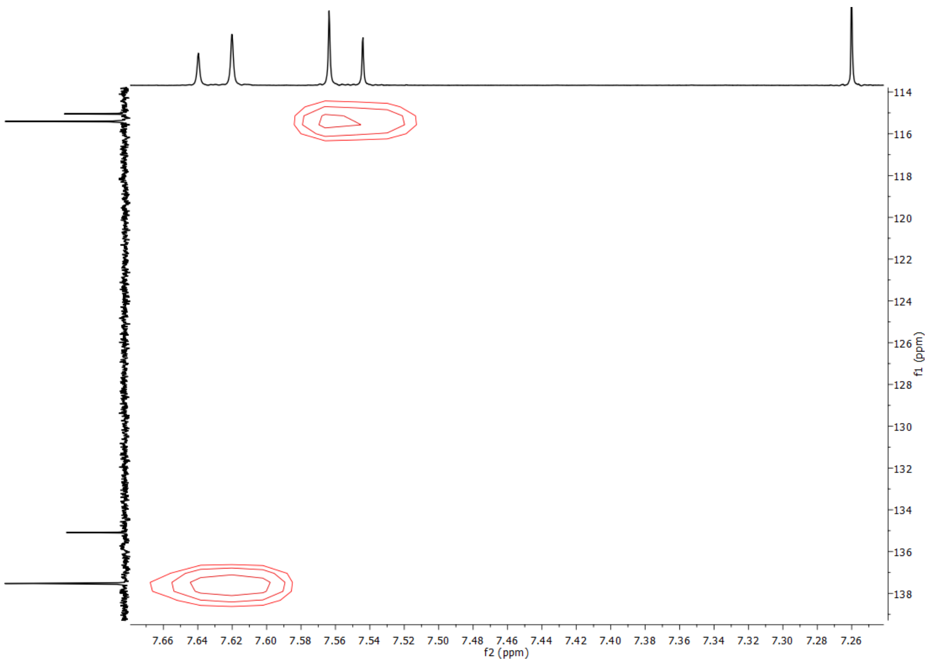

Supplementary Figure 7. HSQC NMR (400 MHz) of **2** in $CDCl_3$, measured at 298 K (expansion in aromatic region).

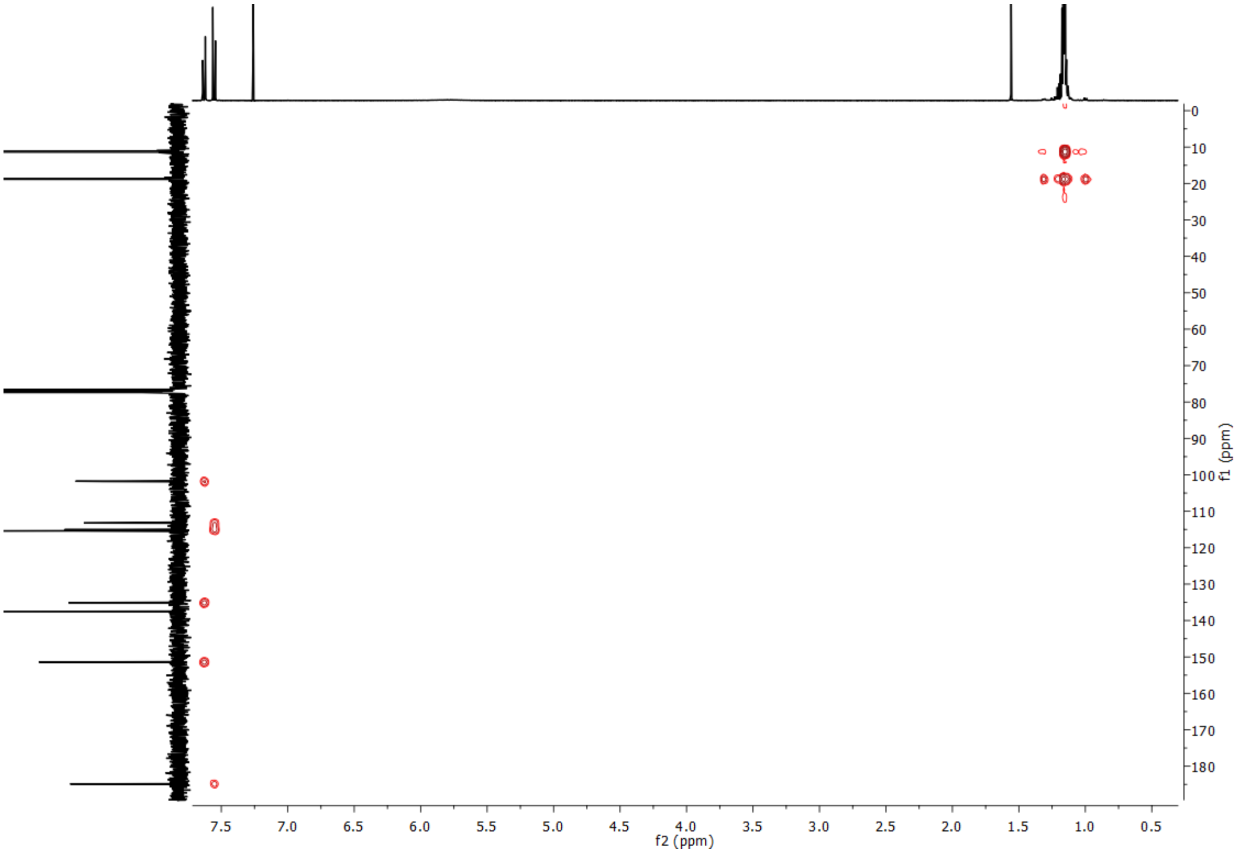

Supplementary Figure 8. HMBC NMR (400 MHz) of **2** in $CDCl_3$, measured at 298 K.

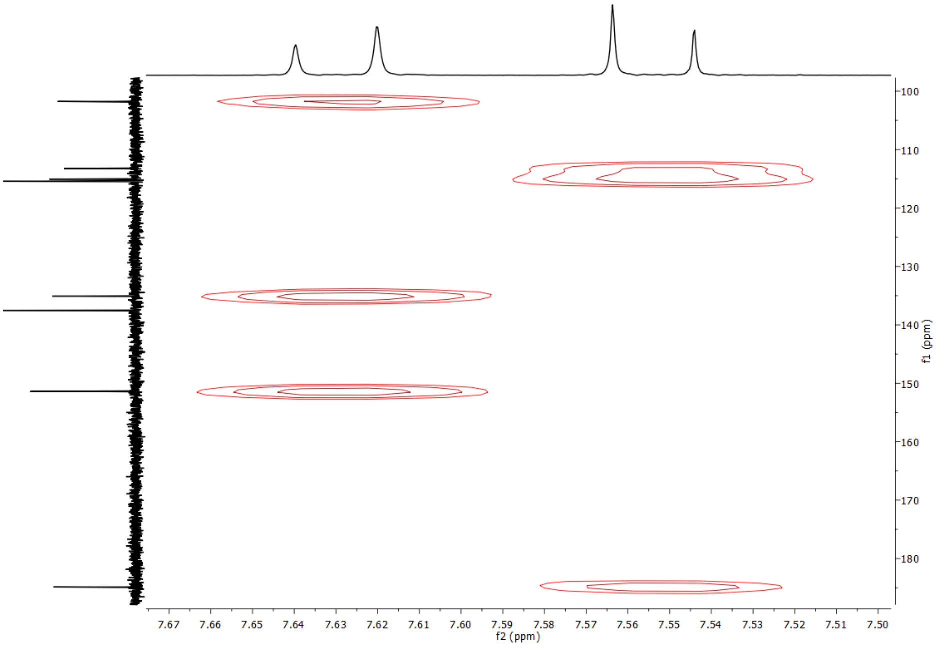

Supplementary Figure 9. HMBC NMR (500 MHz) of **3** in $CDCl_3$, measured at 298 K (expansion in aromatic region).

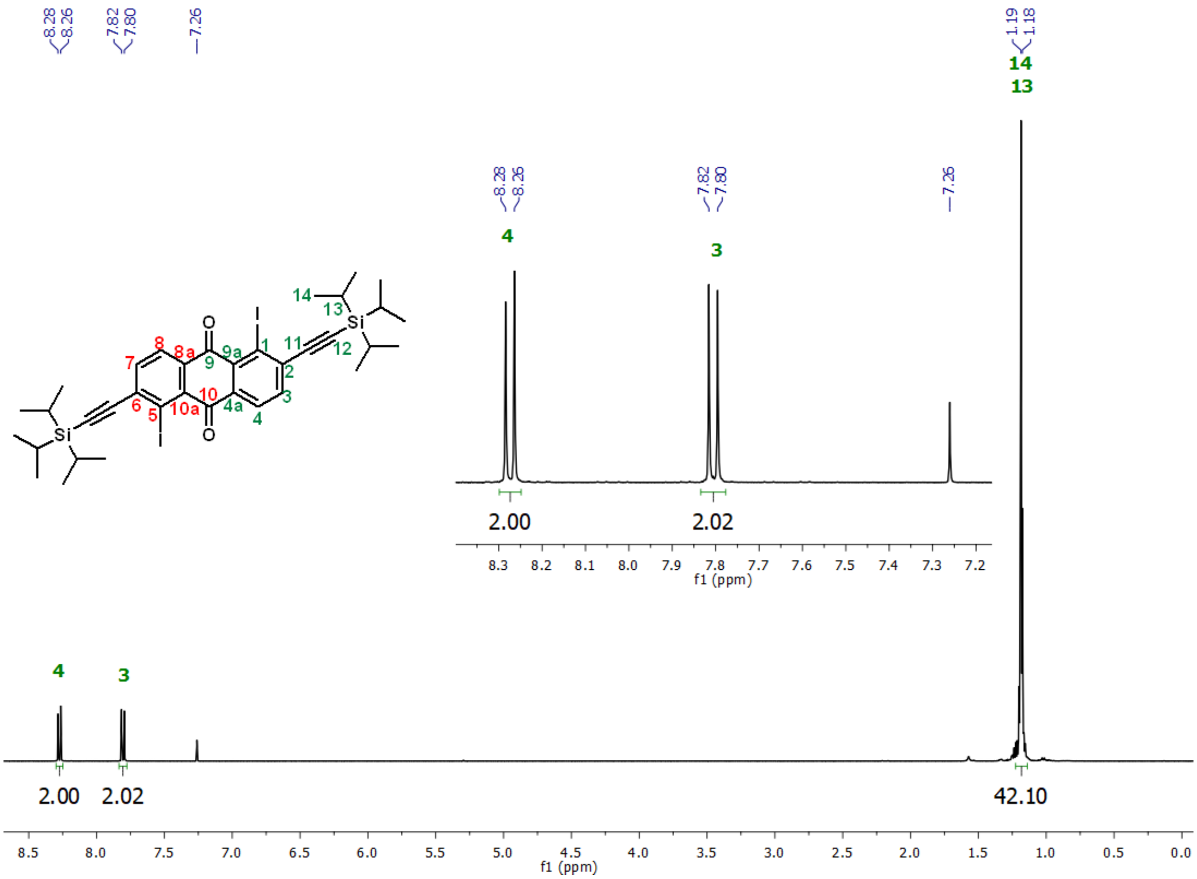

Supplementary Figure 10. ^{1}H NMR (400 MHz) of **3** in $CDCl_3$, measured at 298 K.

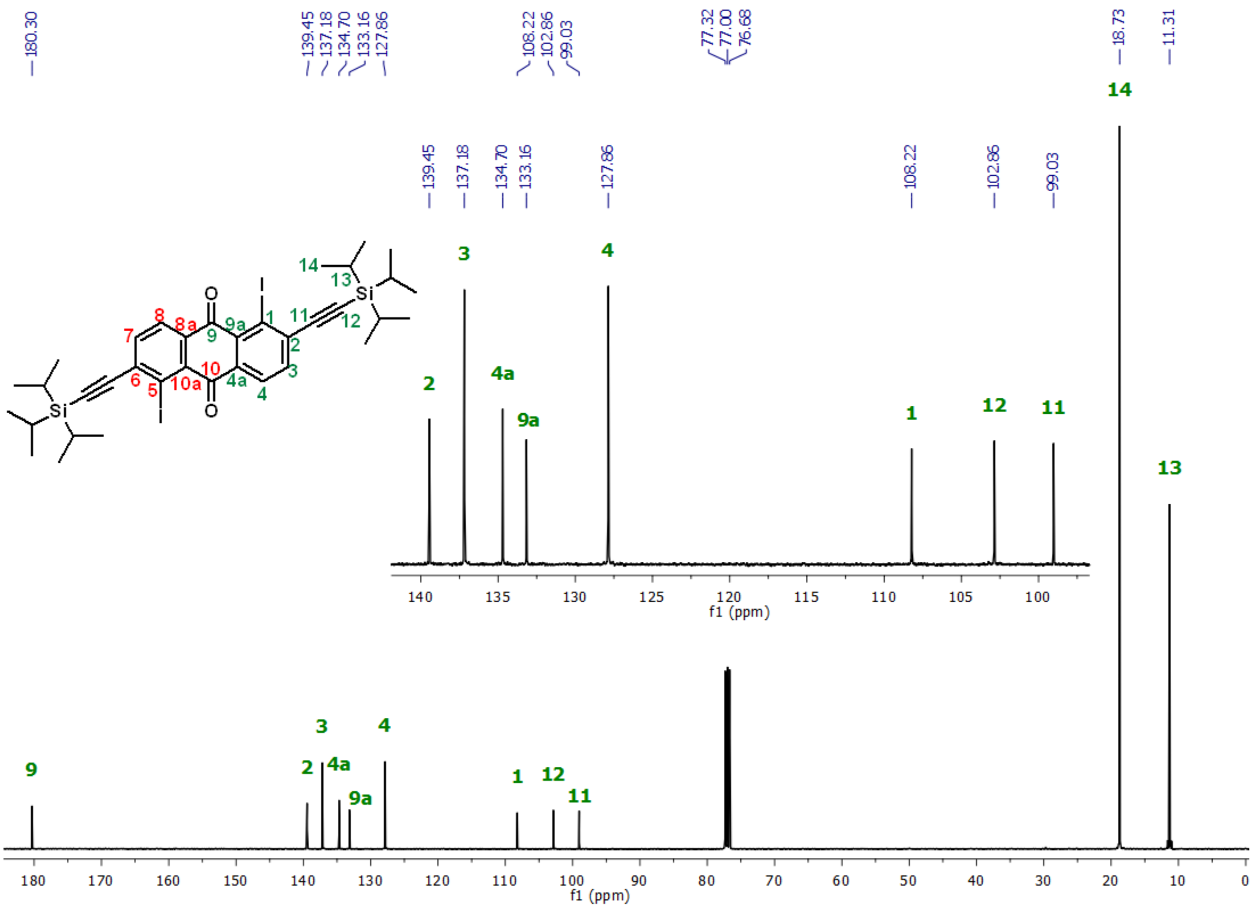

Supplementary Figure 11. ^{13}C NMR (101 MHz) of **3** in $CDCl_3$, measured at 298 K.

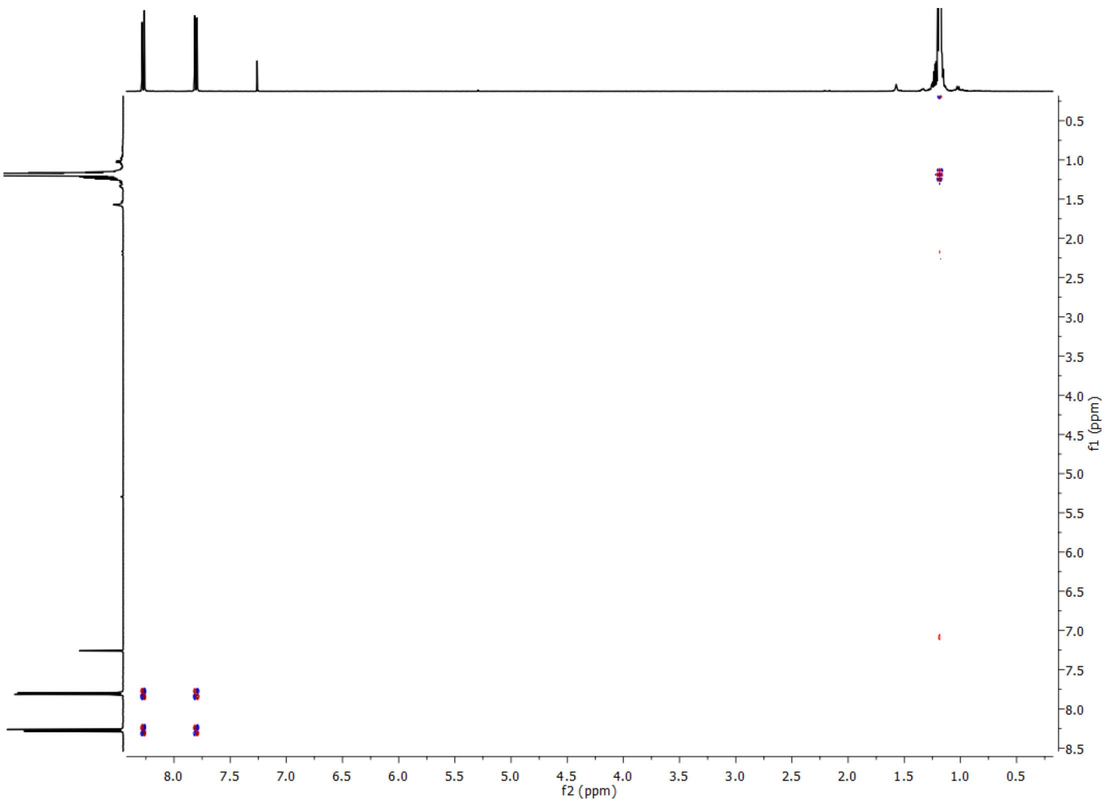

Supplementary Figure 12. COSY NMR (400 MHz) of **3** in $CDCl_3$, measured at 298 K.

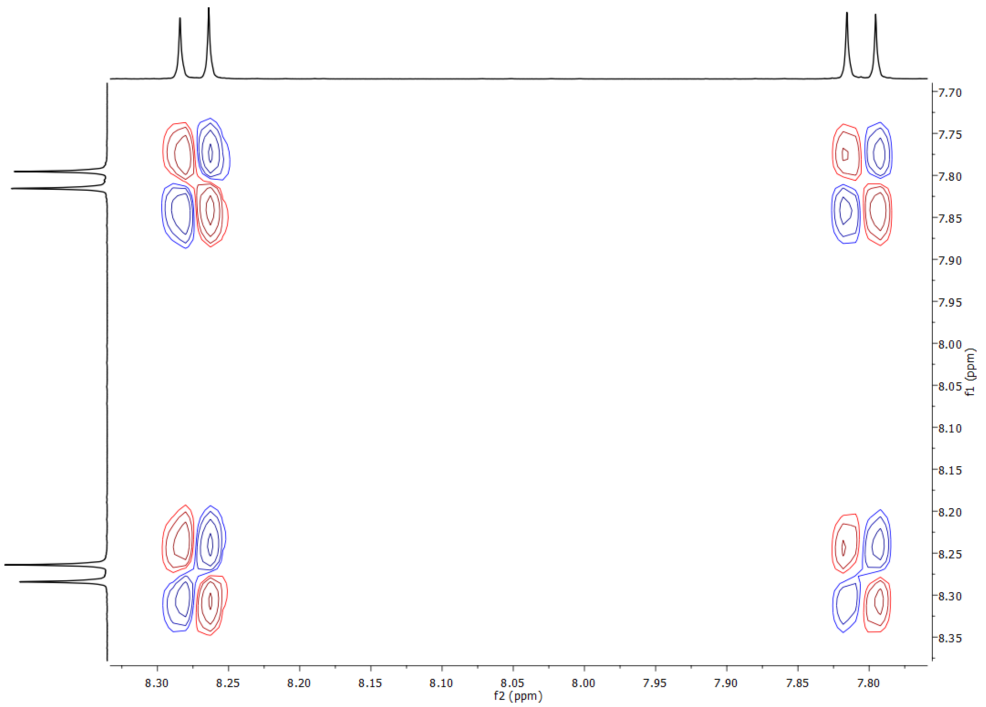

Supplementary Figure 13. COSY NMR (400 MHz) of **3** in $CDCl_3$, measured at 298 K (expansion in aromatic region).

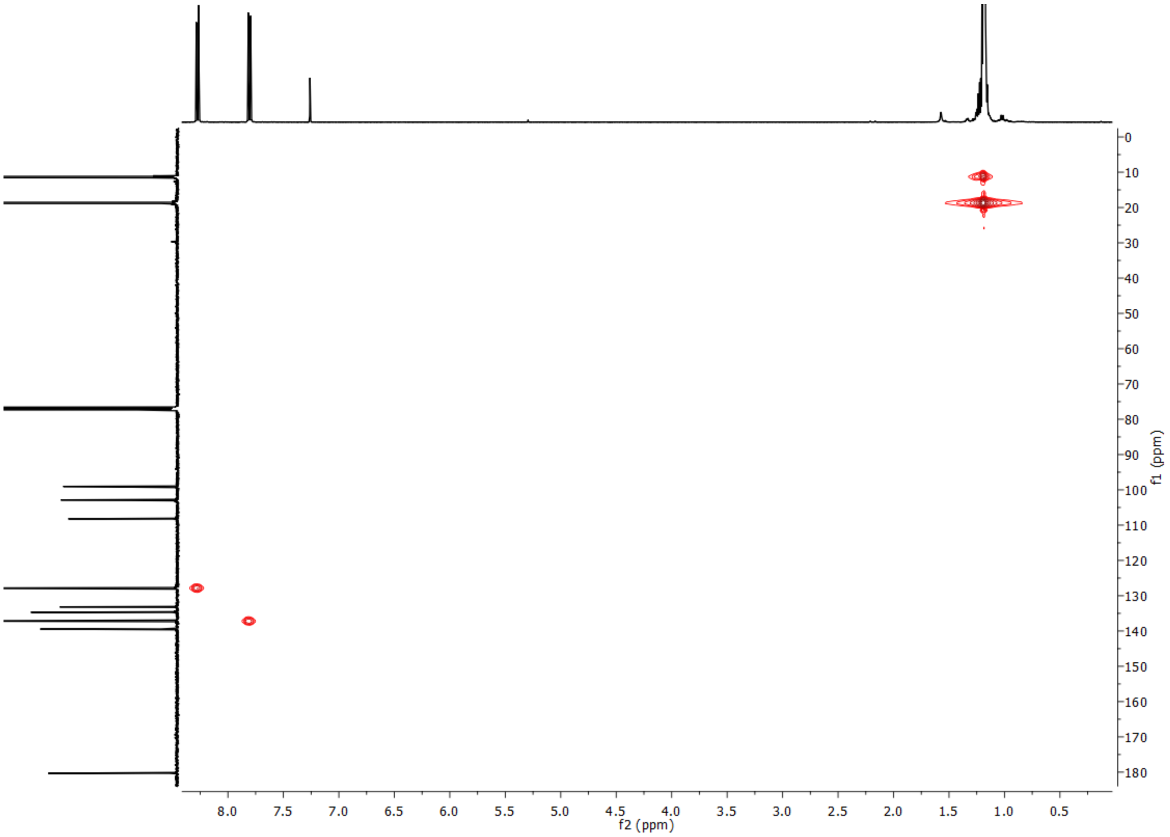

Supplementary Figure 14. HSQC NMR (500 MHz) of **3** in $CDCl_3$, measured at 298 K.

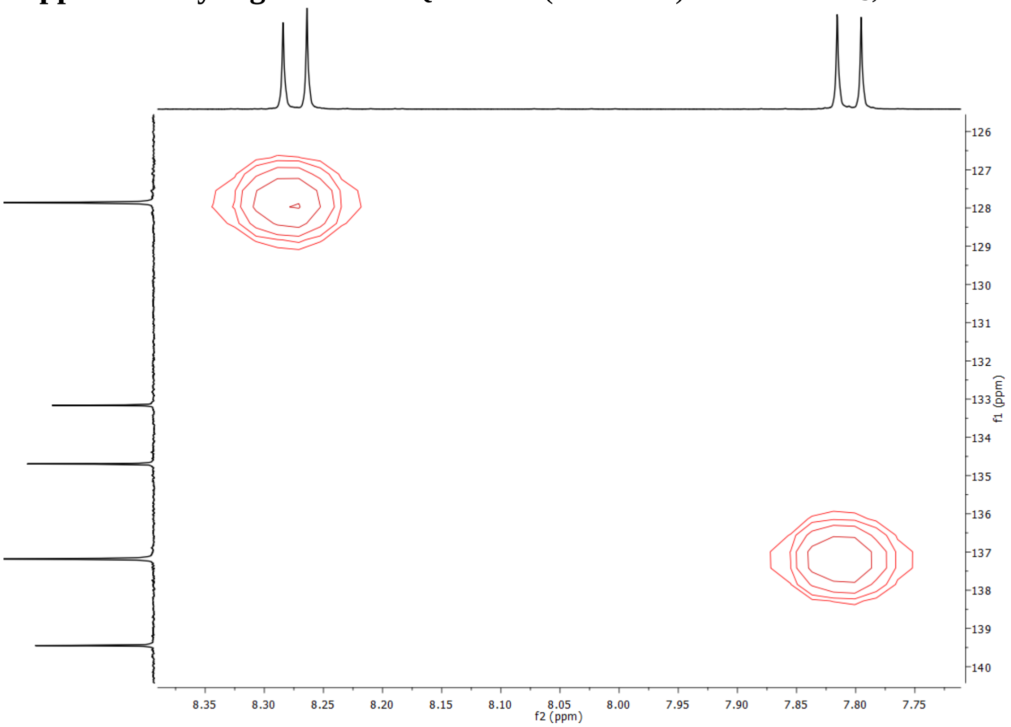

Supplementary Figure 15. HSQC NMR (500 MHz) of **3** in $CDCl_3$, measured at 298 K (expansion in aromatic region).

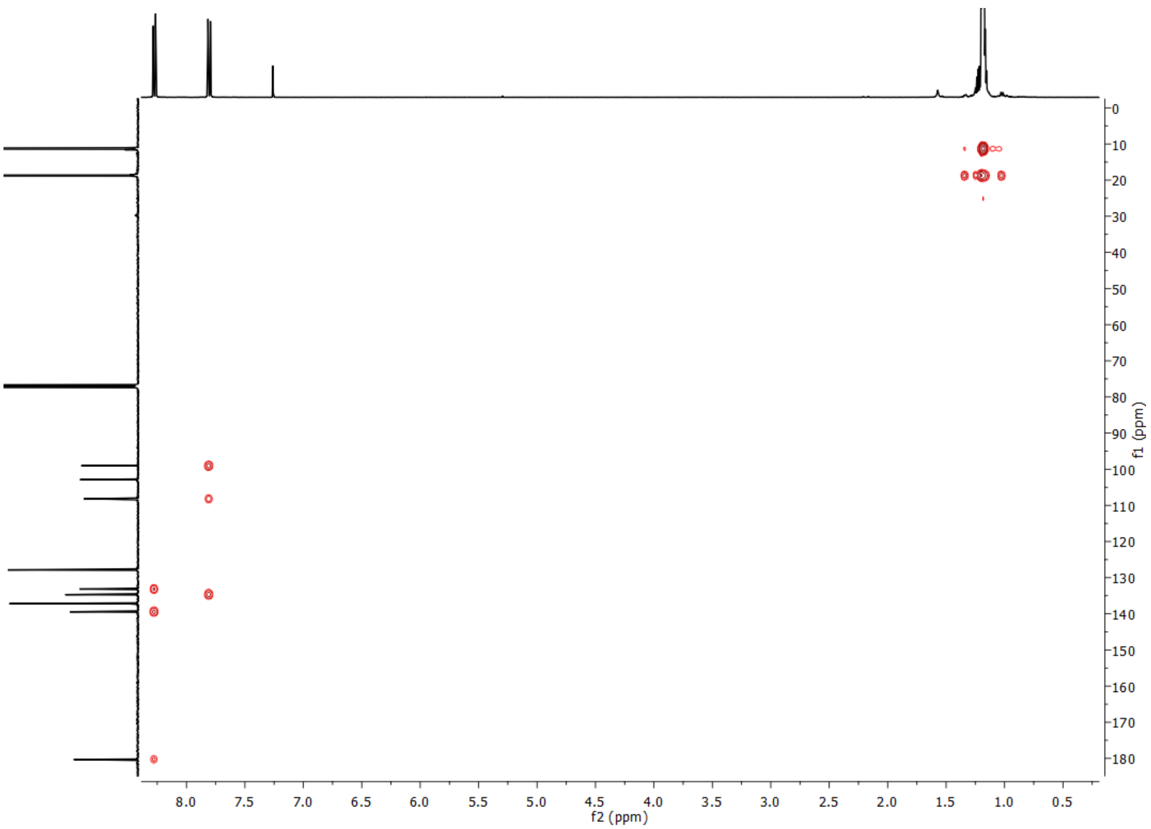

Supplementary Figure 16. HMBC NMR (400 MHz) of **3** in $CDCl_3$, measured at 298 K.

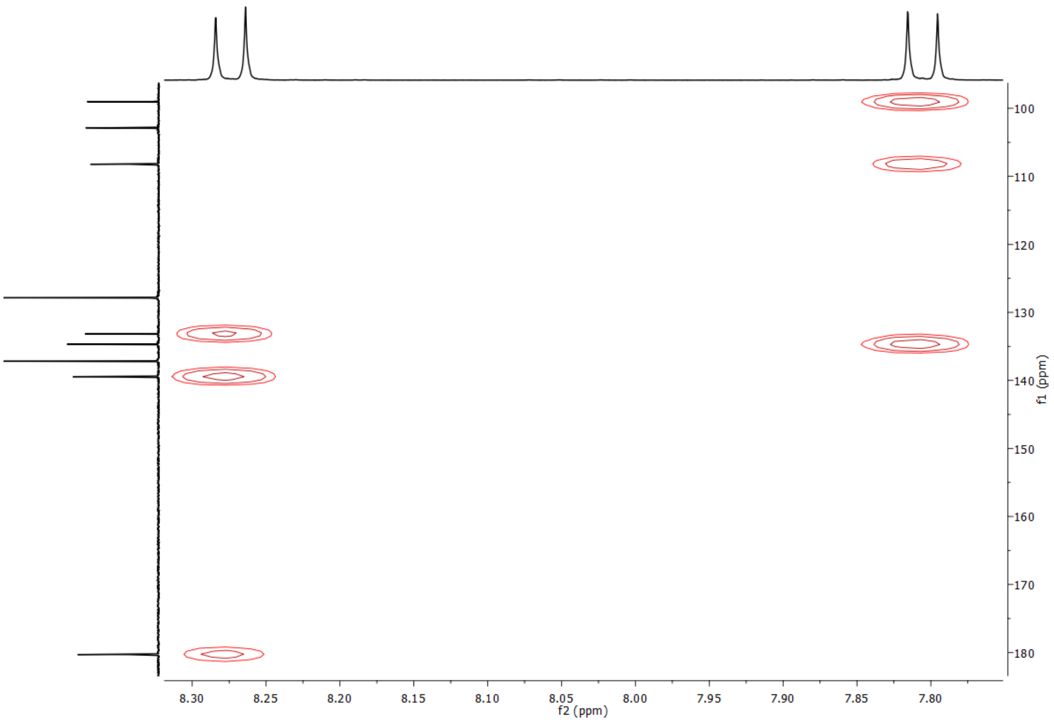

Supplementary Figure 17. HMBC NMR (500 MHz) of **3** in $CDCl_3$, measured at 298 K (expansion in aromatic region).

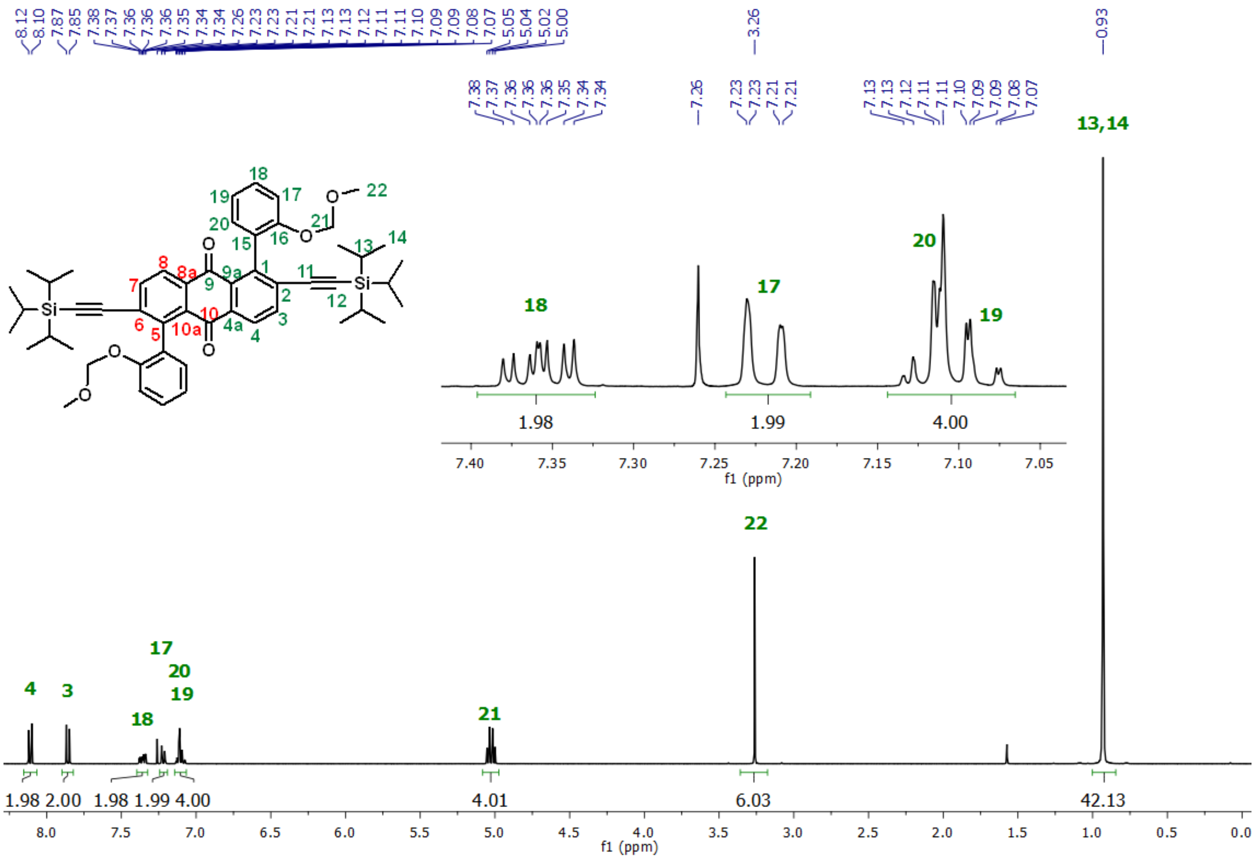

Supplementary Figure 18. ^{1}H NMR (400 MHz) of **4** in $CDCl_3$, measured at 298 K.

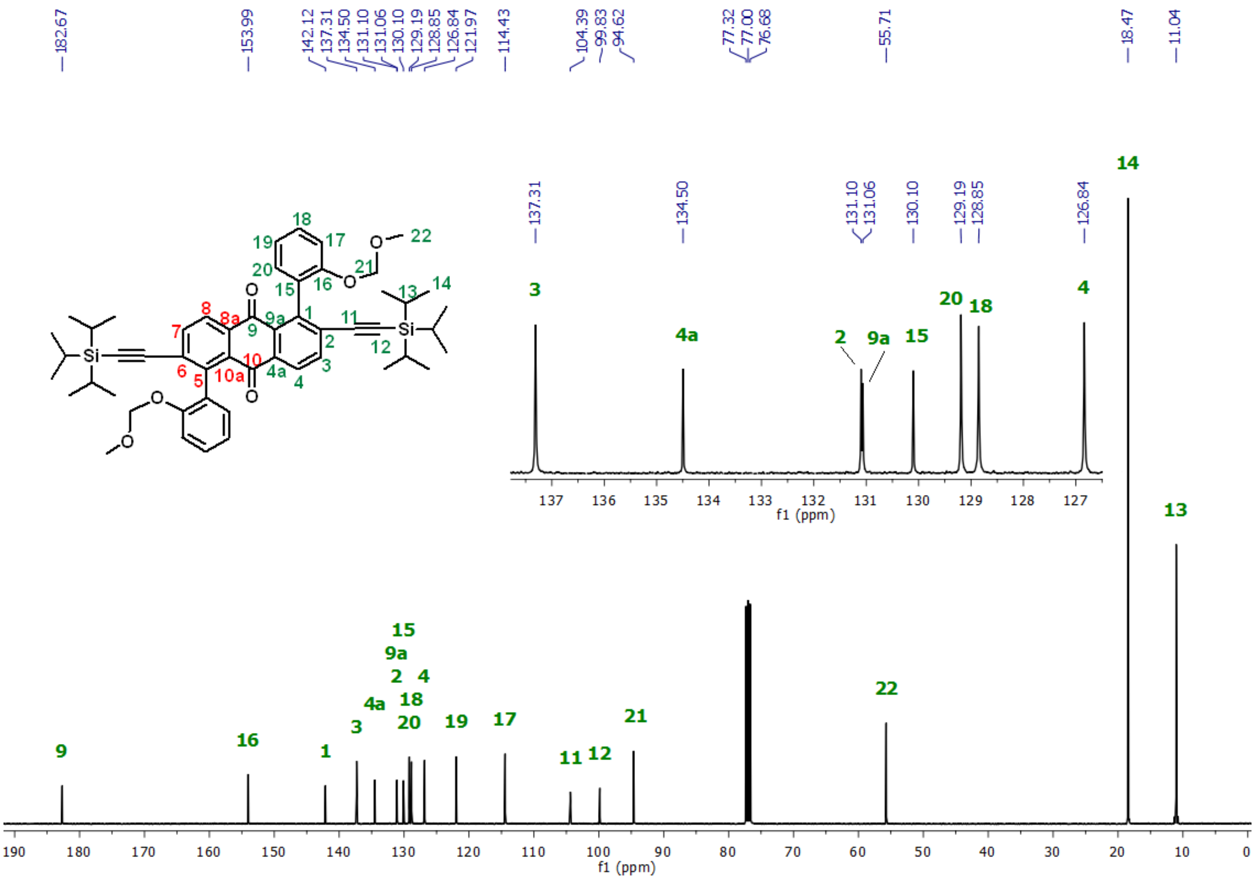

Supplementary Figure 19. ^{13}C NMR (101 MHz) of **5** in $CDCl_3$, measured at 298 K.

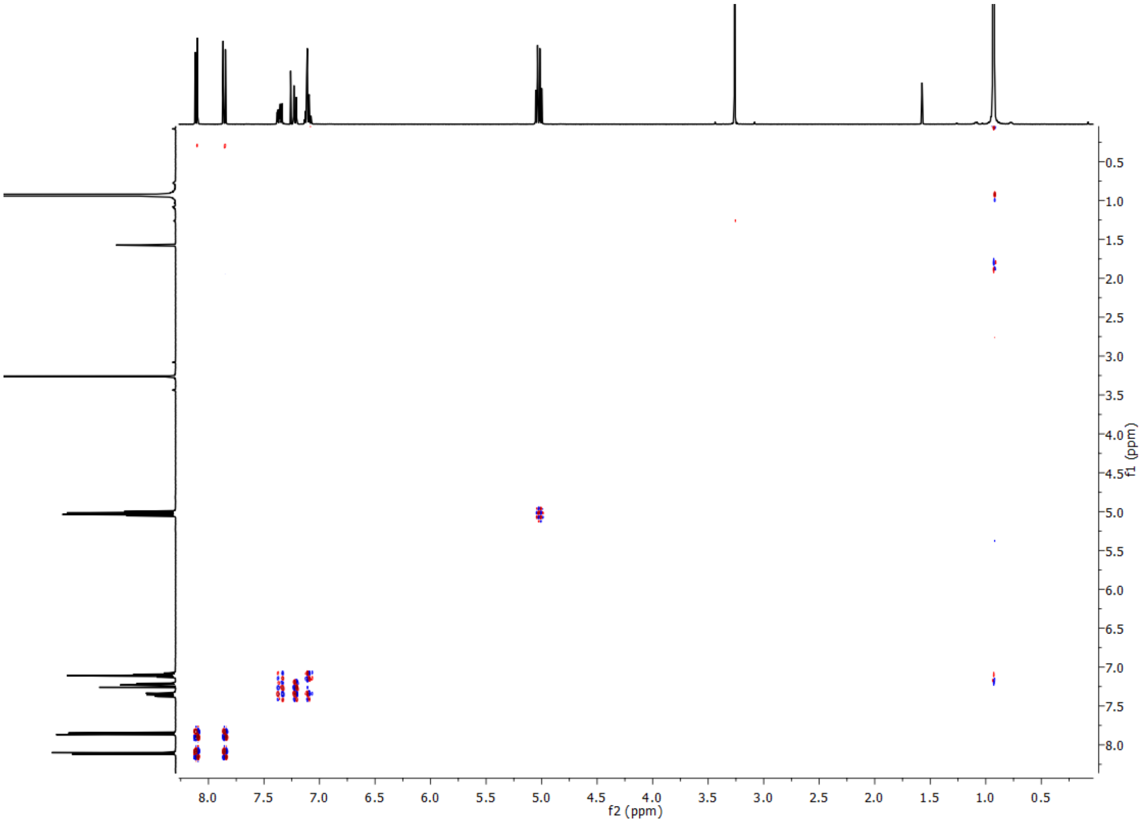

Supplementary Figure 20. COSY NMR (400 MHz) of **4** in $CDCl_3$, measured at 298 K.

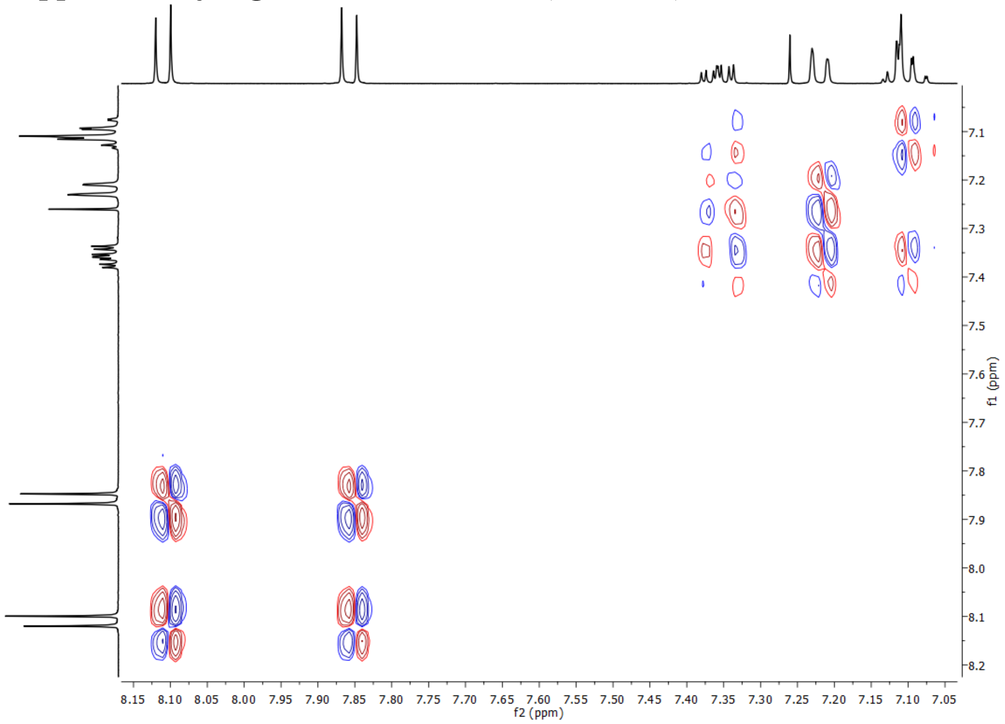

Supplementary Figure 21. COSY NMR (400 MHz) of **4** in $CDCl_3$, measured at 298 K (expansion in aromatic region).

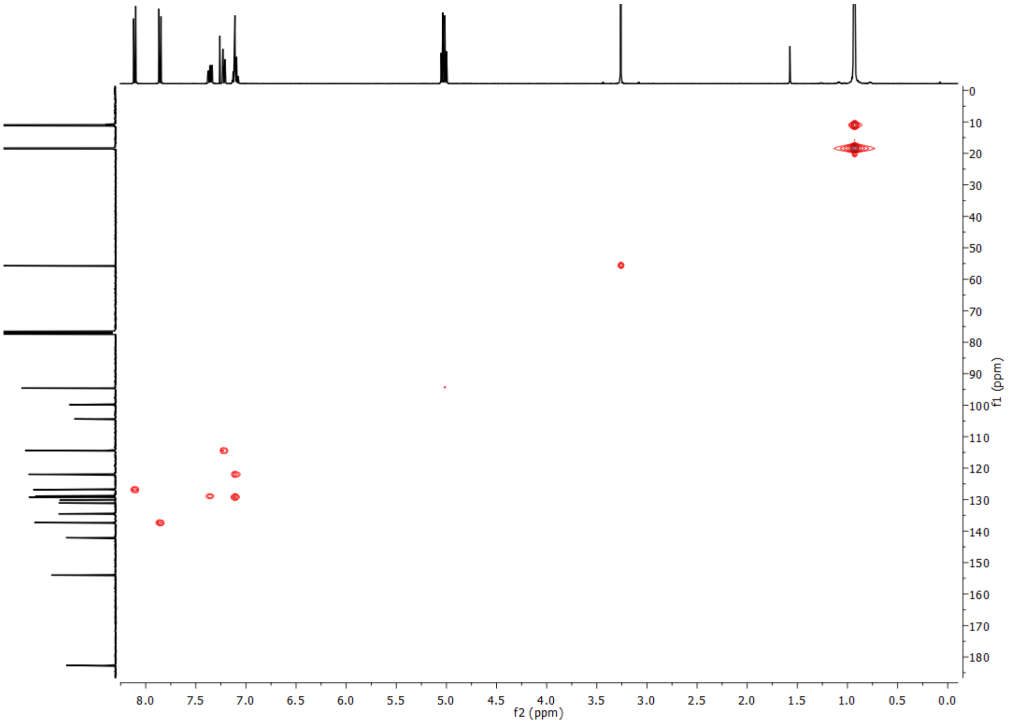

Supplementary Figure 22. HSQC NMR (400 MHz) of **4** in $CDCl_3$, measured at 298 K.

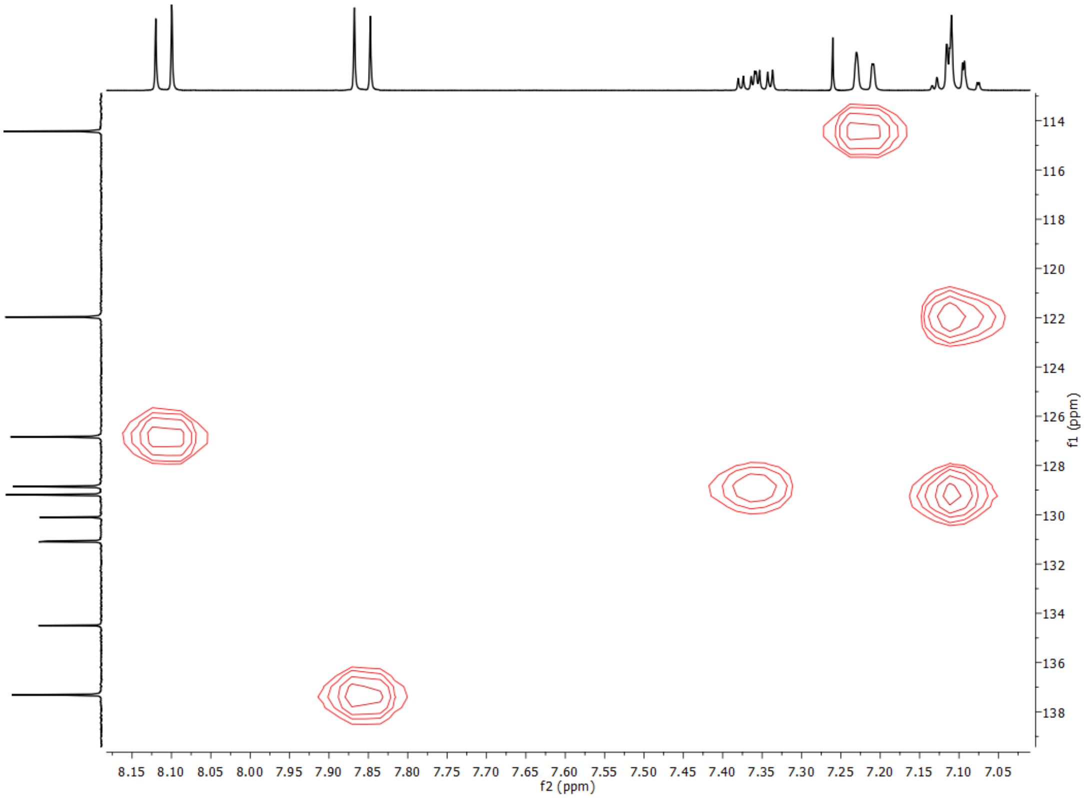

Supplementary Figure 23. HSQC NMR (400 MHz) of **4** in $CDCl_3$, measured at 298 K (expansion in aromatic region).

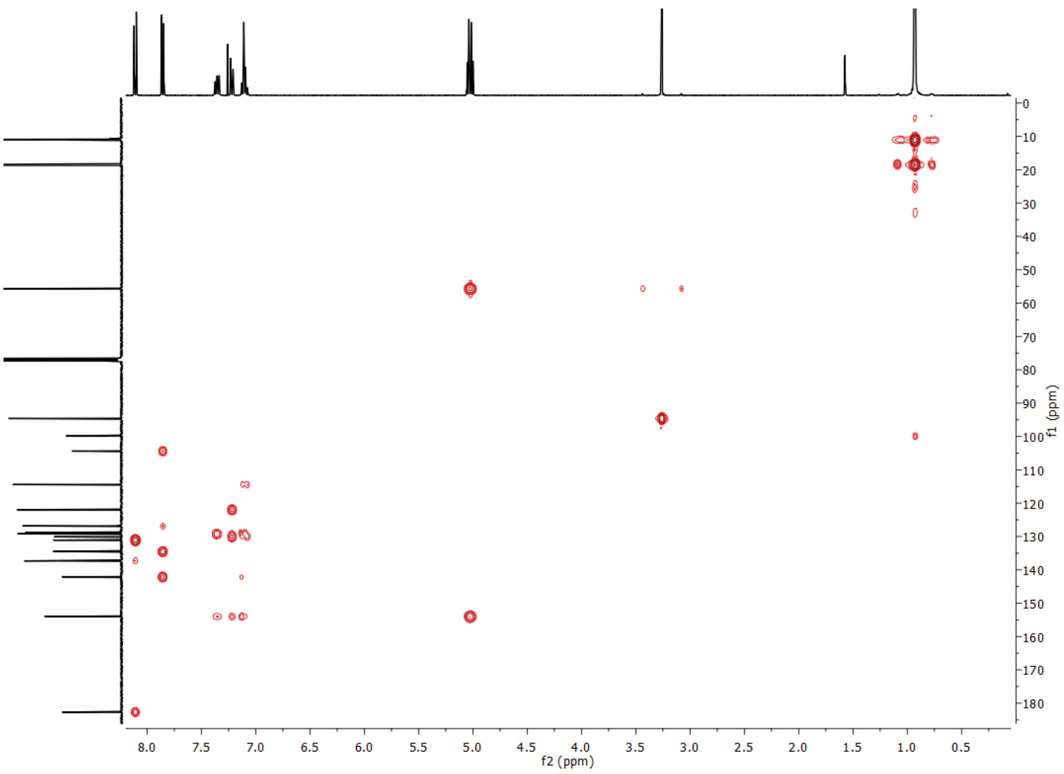

Supplementary Figure 24. HMBC NMR (400 MHz) of **4** in $CDCl_3$, measured at 298 K.

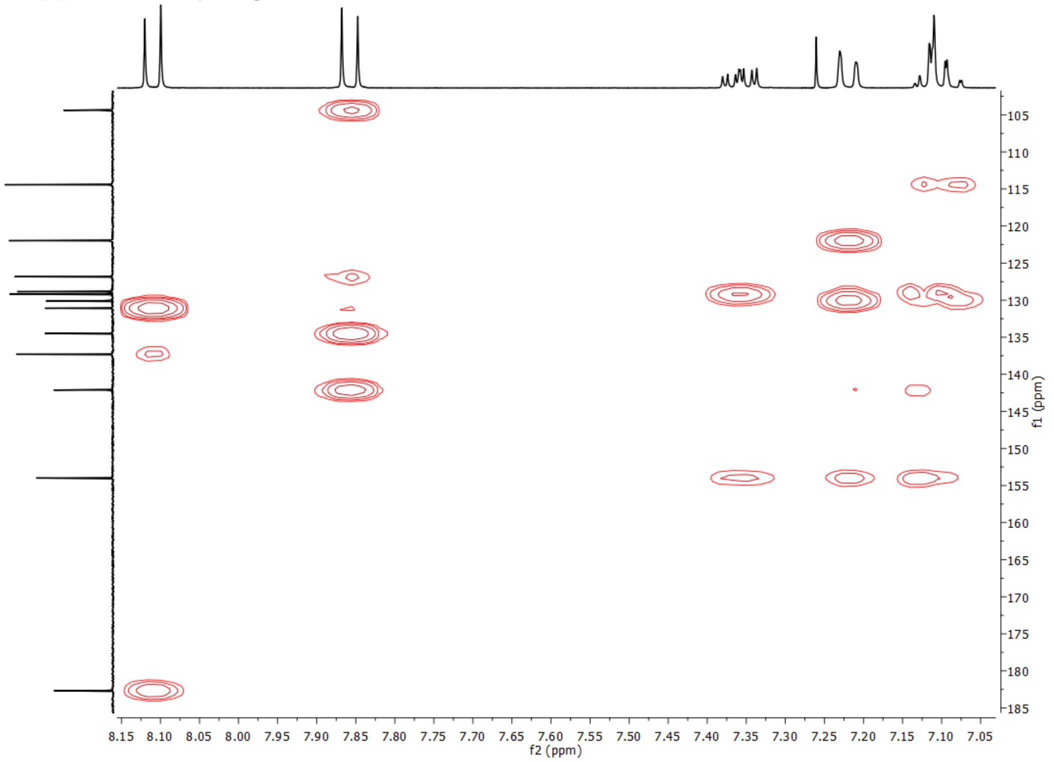

Supplementary Figure 25. HMBC NMR (400 MHz) of **4** in $CDCl_3$, measured at 298 K (expansion in aromatic region).

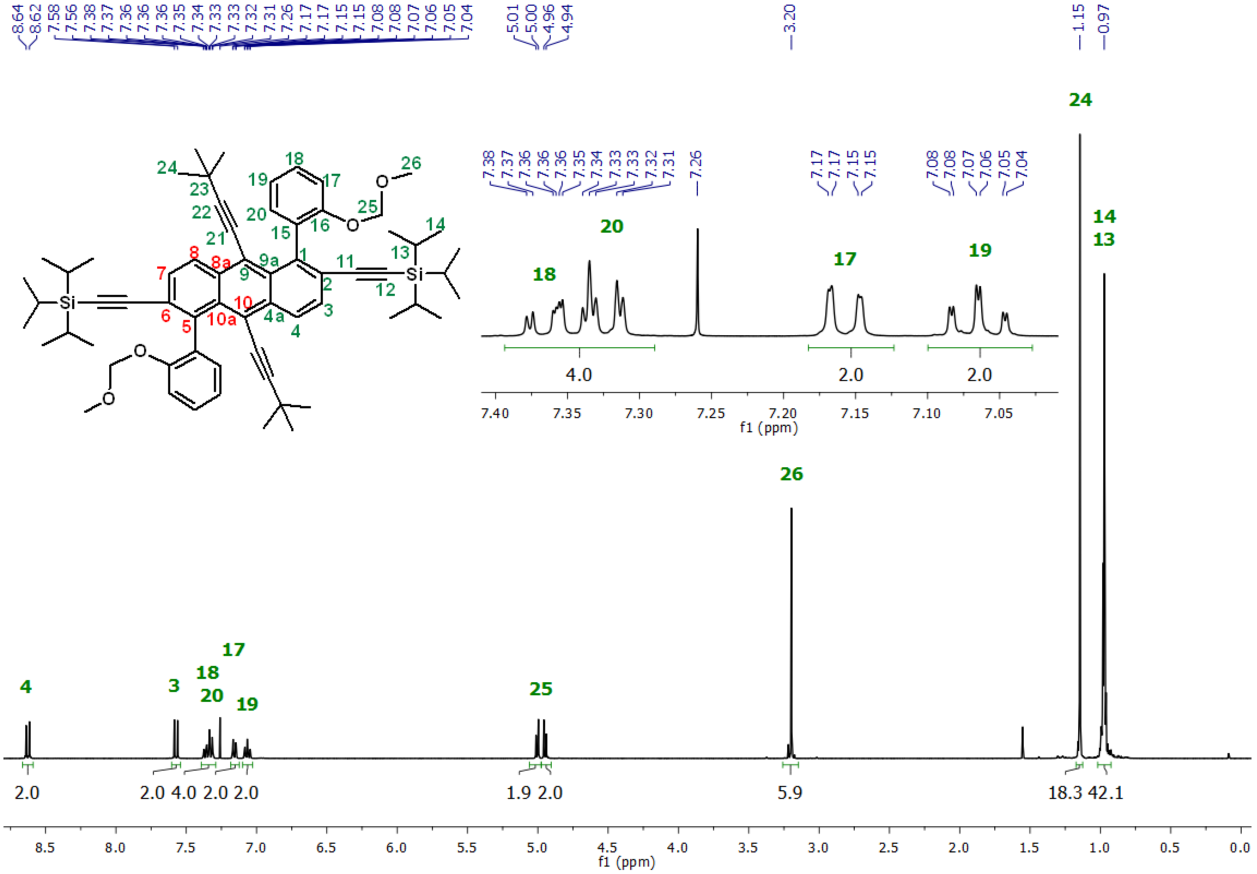

Supplementary Figure 26. ^{1}H NMR (400 MHz) of **5** in $CDCl_3$, measured at 298 K.

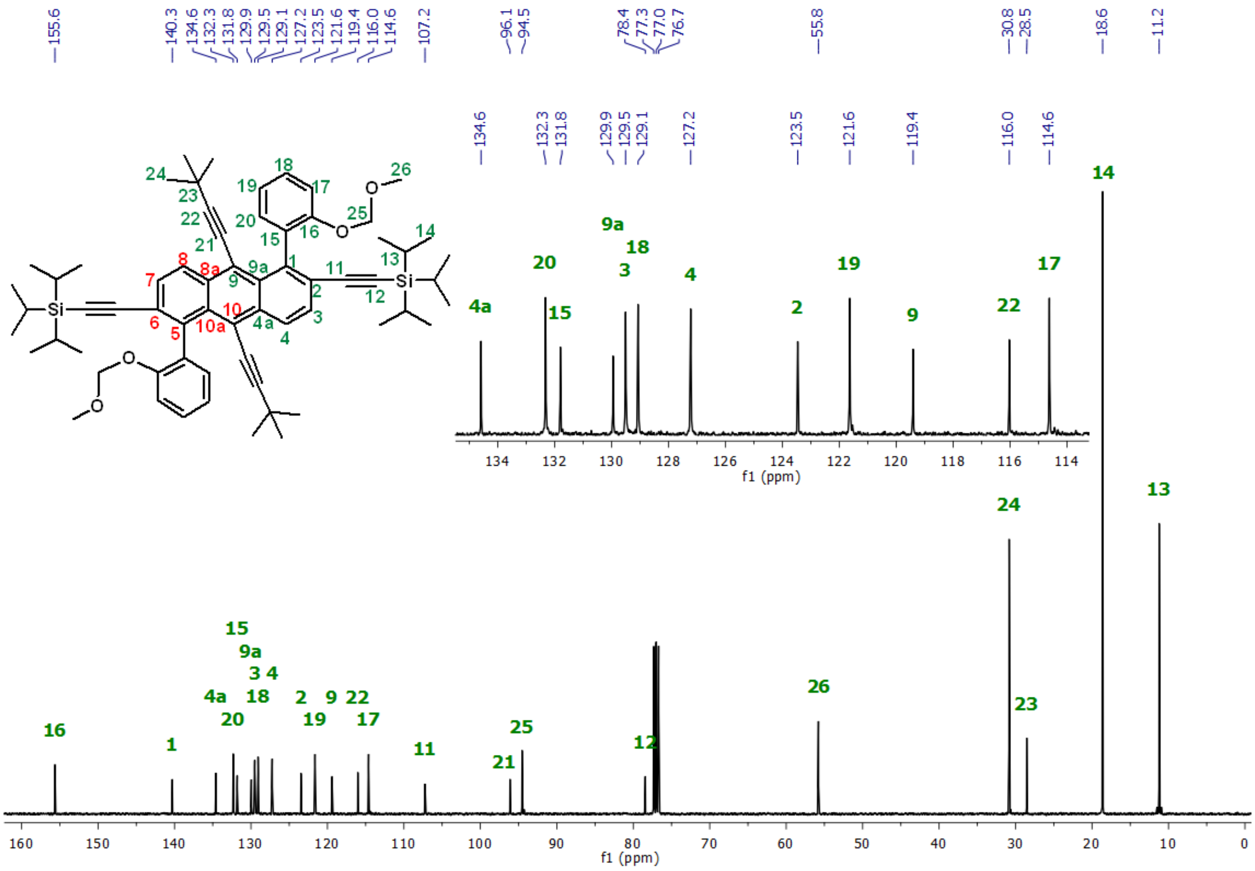

Supplementary Figure 27. ^{13}C NMR (101 MHz) of **5** in $CDCl_3$, measured at 298 K.

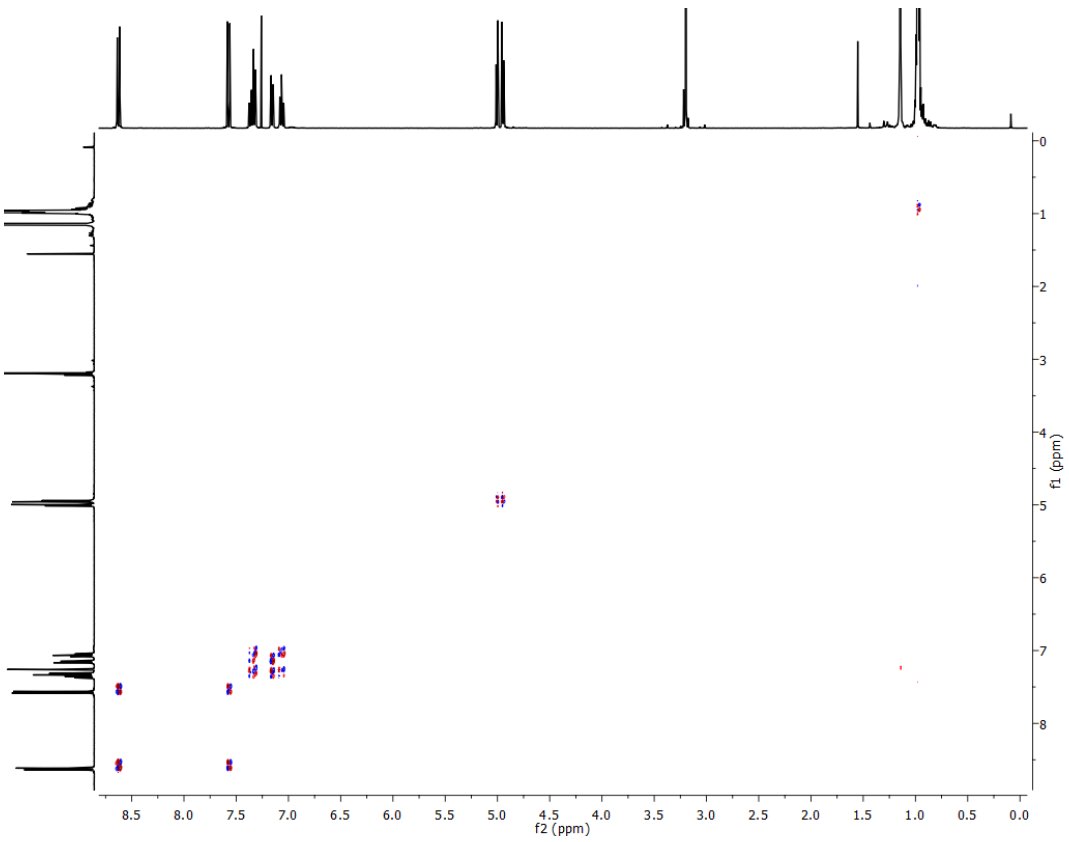

Supplementary Figure 28. COSY NMR (400 MHz) of **5** in $CDCl_3$, measured at 298 K.

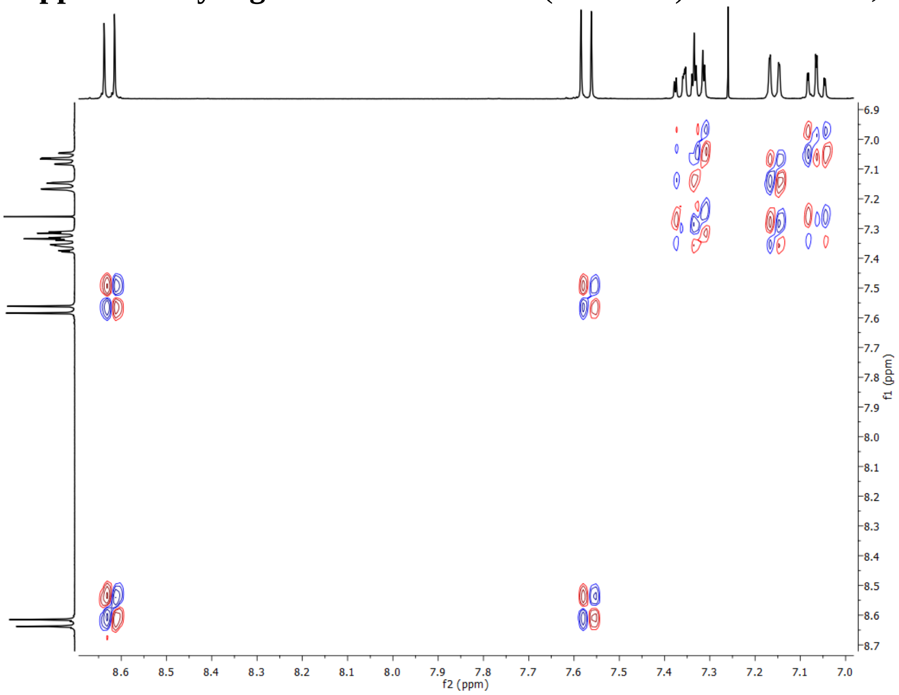

Supplementary Figure 29. COSY NMR (400 MHz) of **5** in $CDCl_3$, measured at 298 K (expansion in aromatic region).

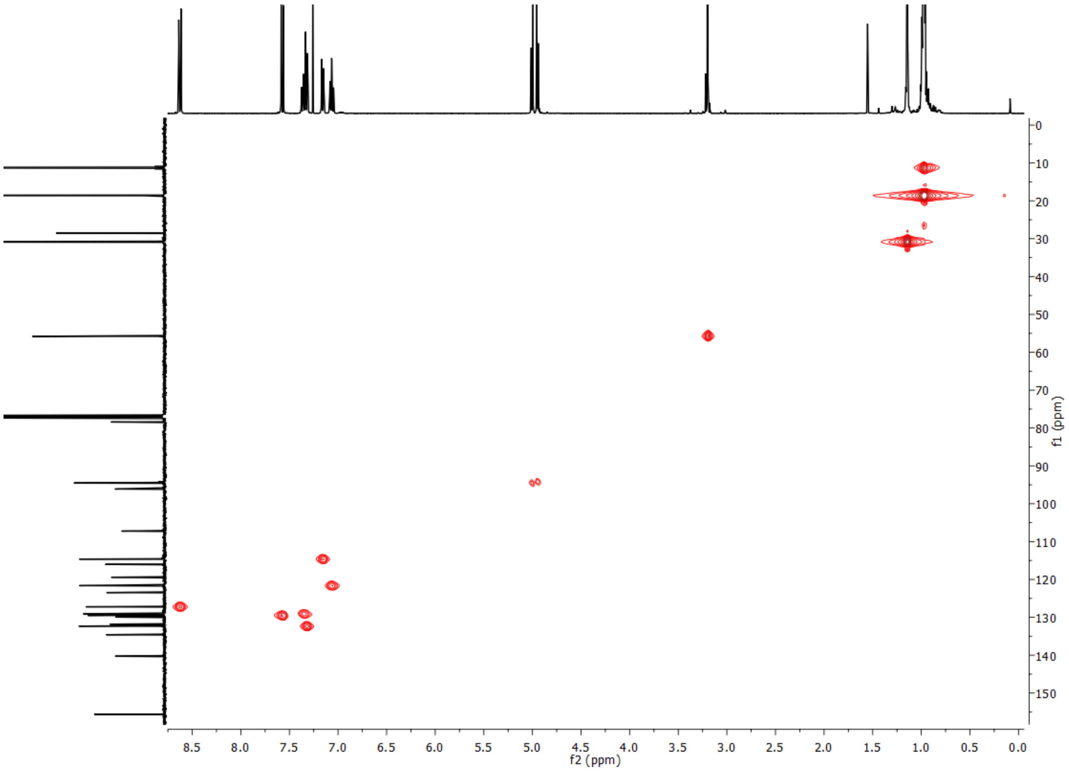

Supplementary Figure 30. HSQC NMR (400 MHz) of **5** in $CDCl_3$, measured at 298 K.

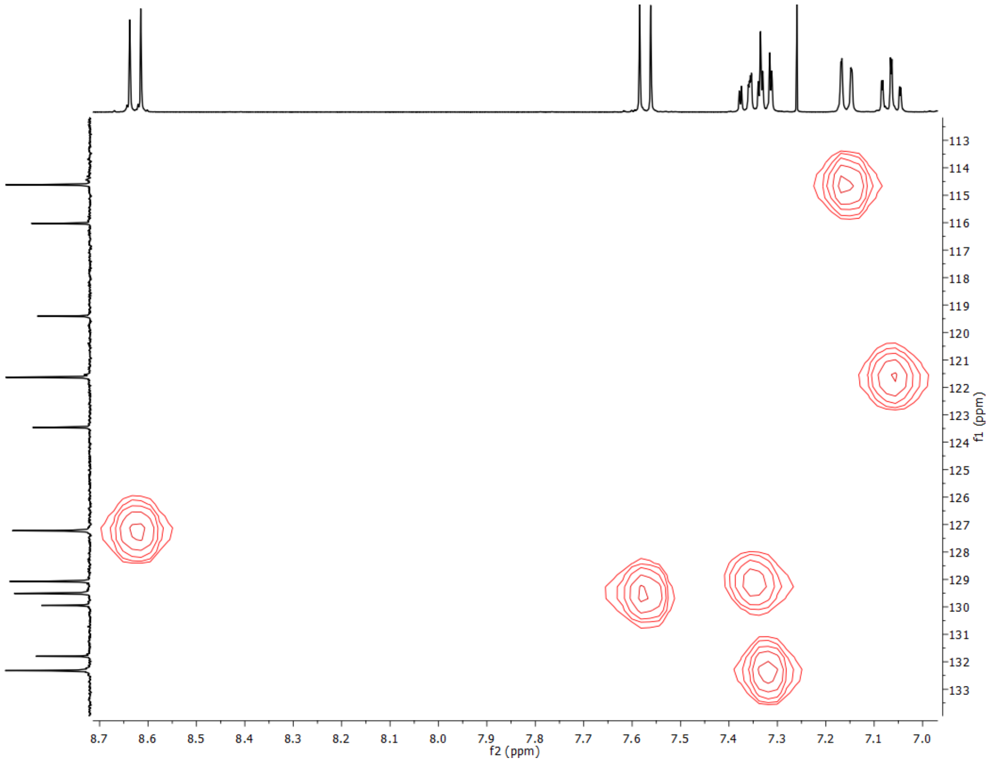

Supplementary Figure 31. HSQC NMR (400 MHz) of **5** in $CDCl_3$, measured at 298 K (expansion in aromatic region).

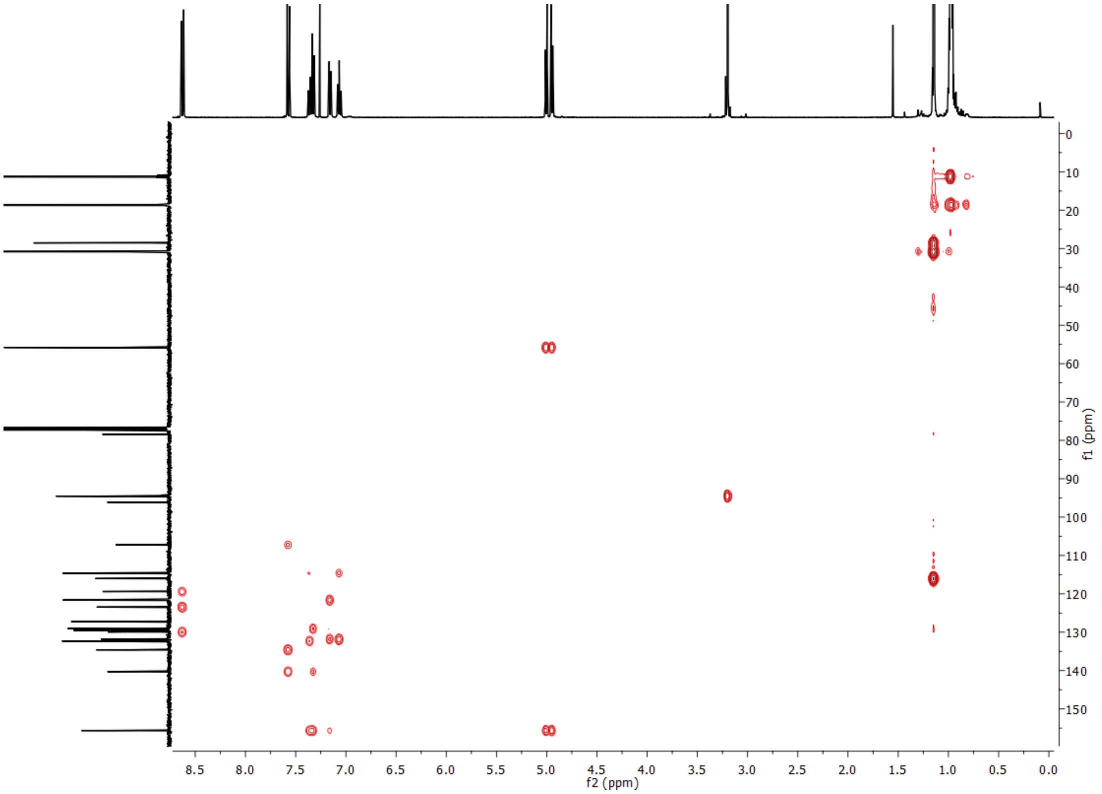

Supplementary Figure 32. HMBC NMR (400 MHz) of **5** in $CDCl_3$, measured at 298 K.

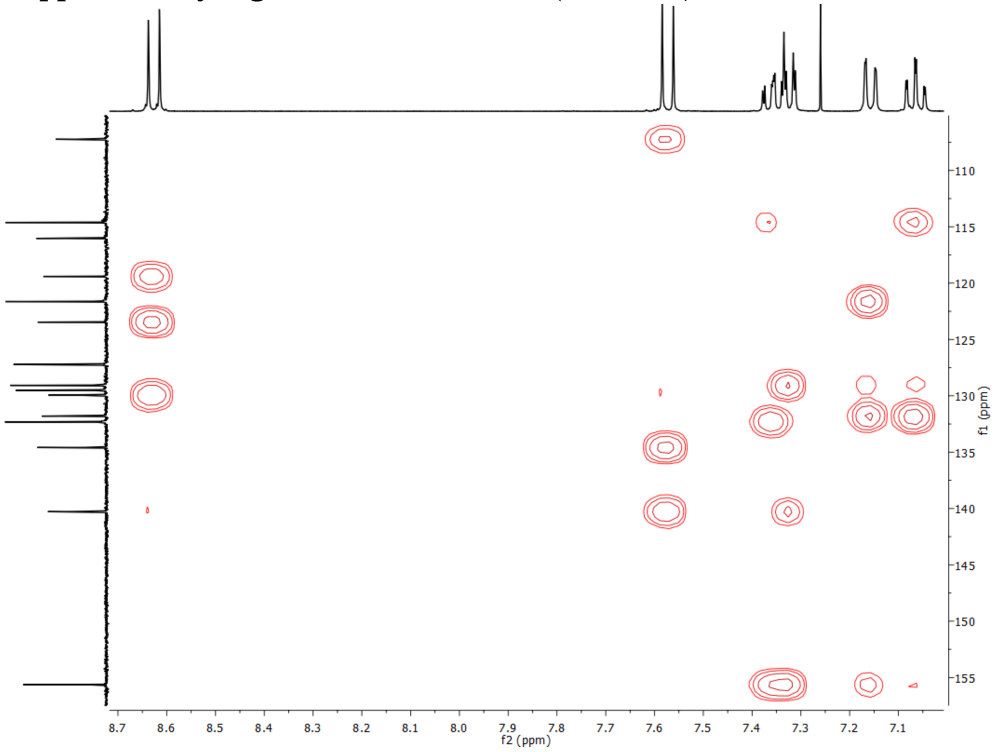

Supplementary Figure 33. HMBC NMR (400 MHz) of **5** in $CDCl_3$, measured at 298 K (expansion in aromatic region).

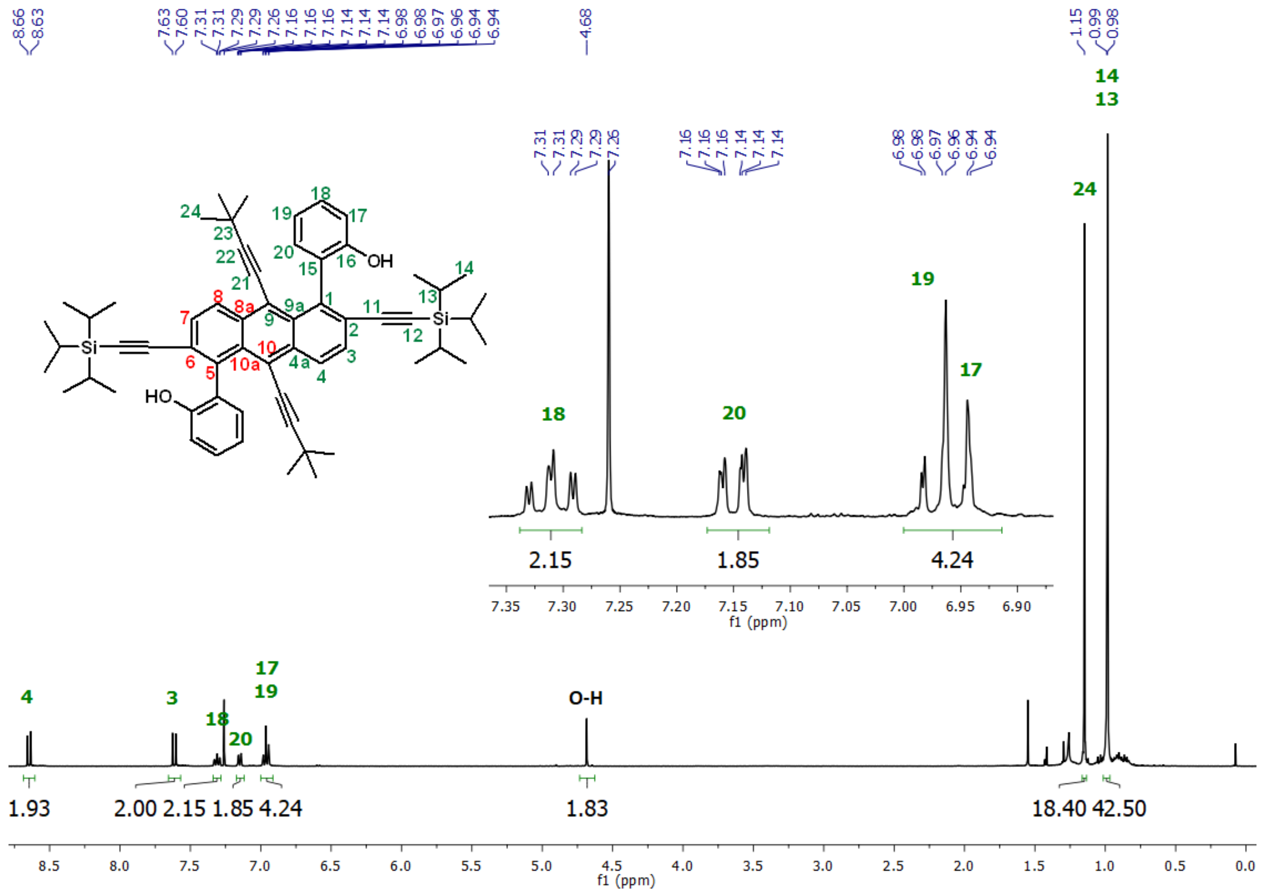

Supplementary Figure 34. ^{1}H NMR (400 MHz) of **6** in $CDCl_3$, measured at 298 K.

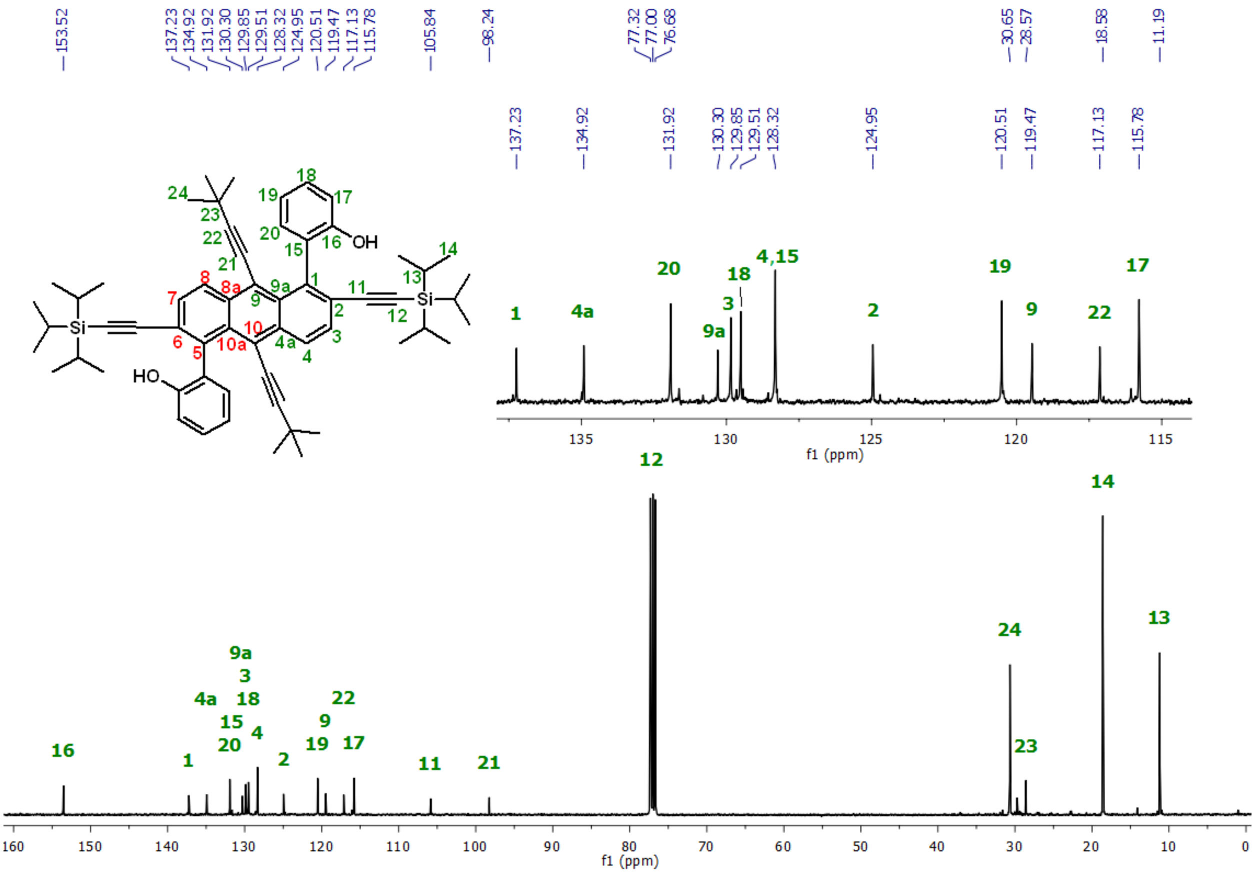

Supplementary Figure 35. ^{13}C NMR (101 MHz) of **6** in $CDCl_3$, measured at 298 K.

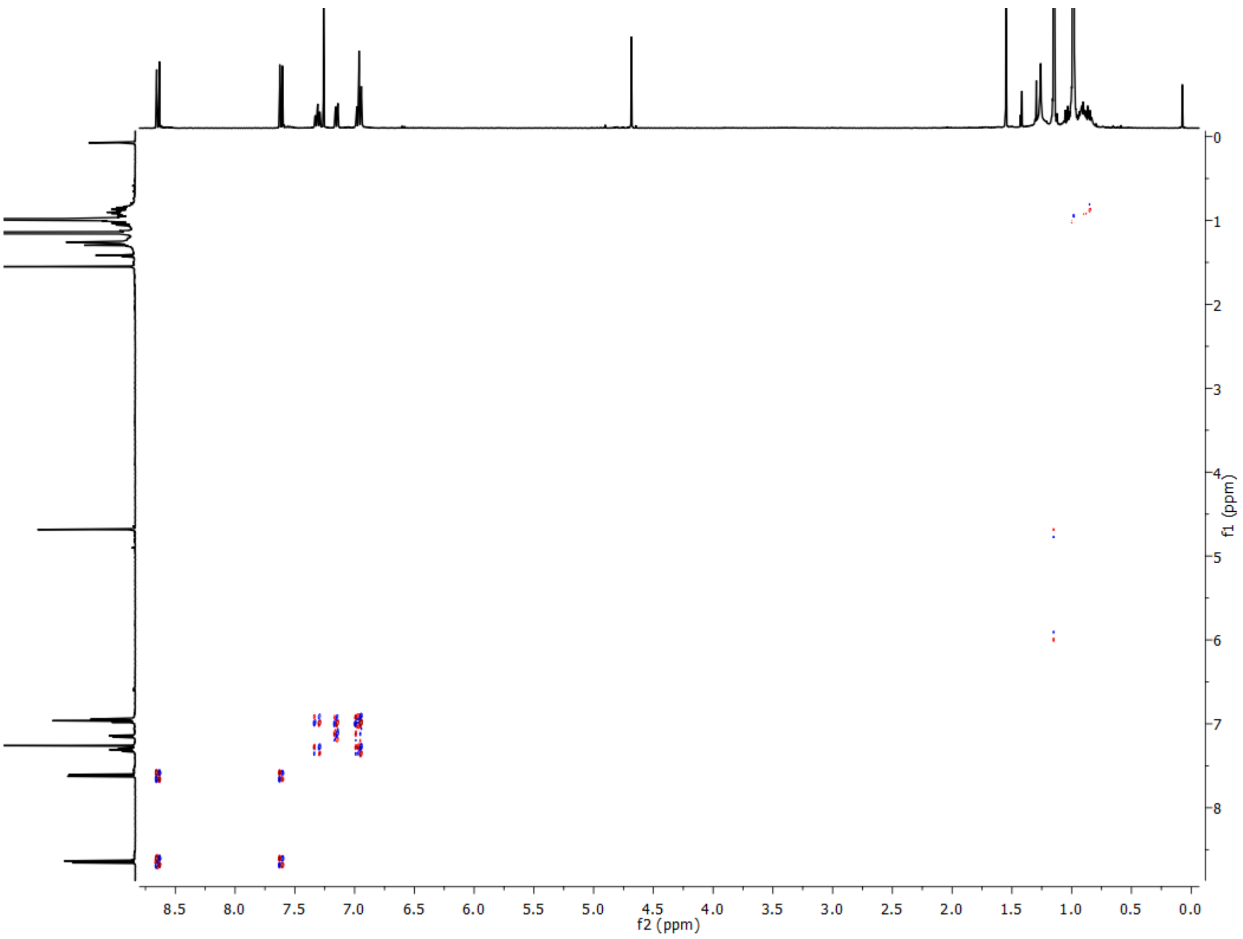

Supplementary Figure 36. COSY NMR (400 MHz) of **6** in $CDCl_3$, measured at 298 K.

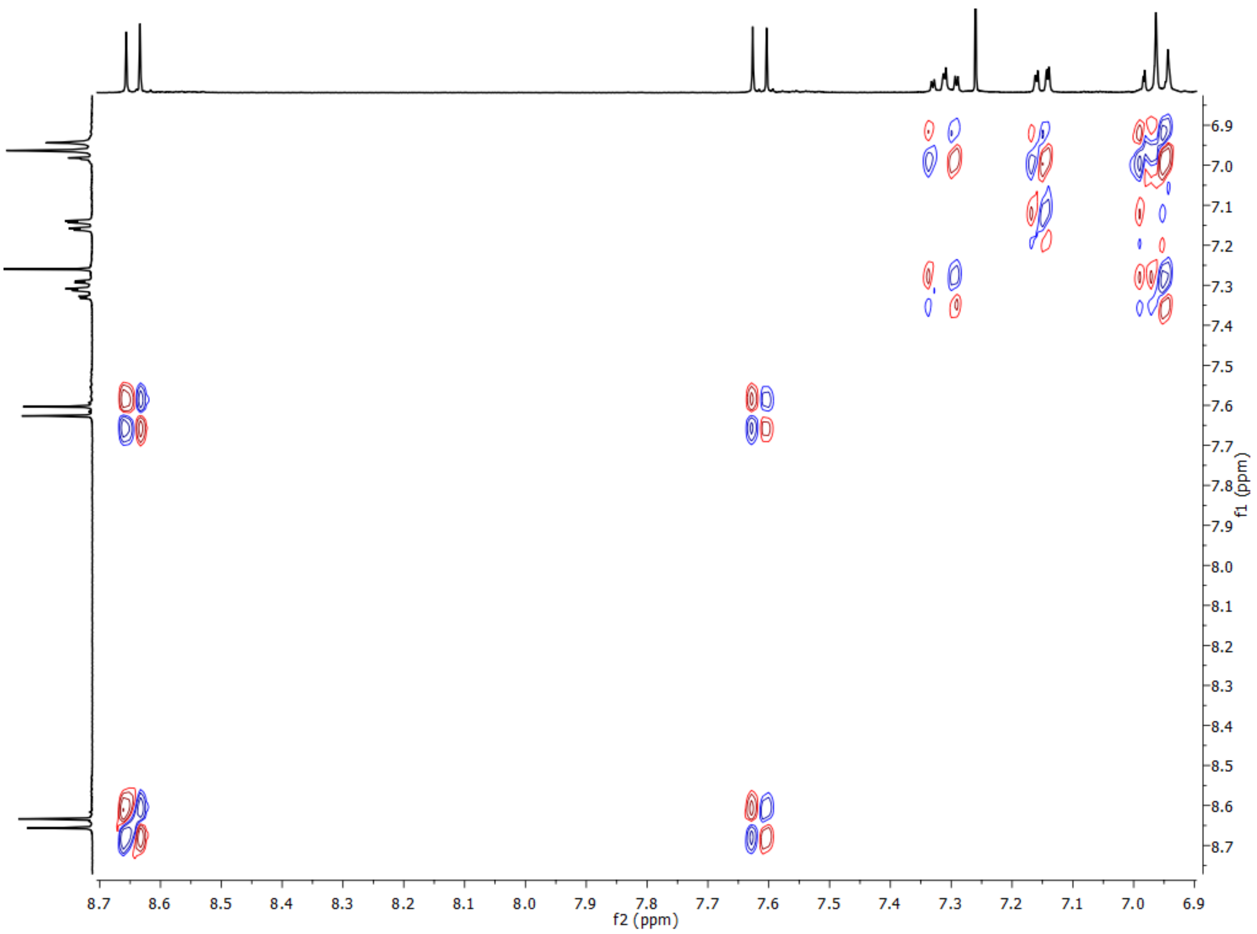

Figure 37. COSY NMR (400 MHz) of **6** in $CDCl_3$, measured at 298 K (expansion in aromatic region).

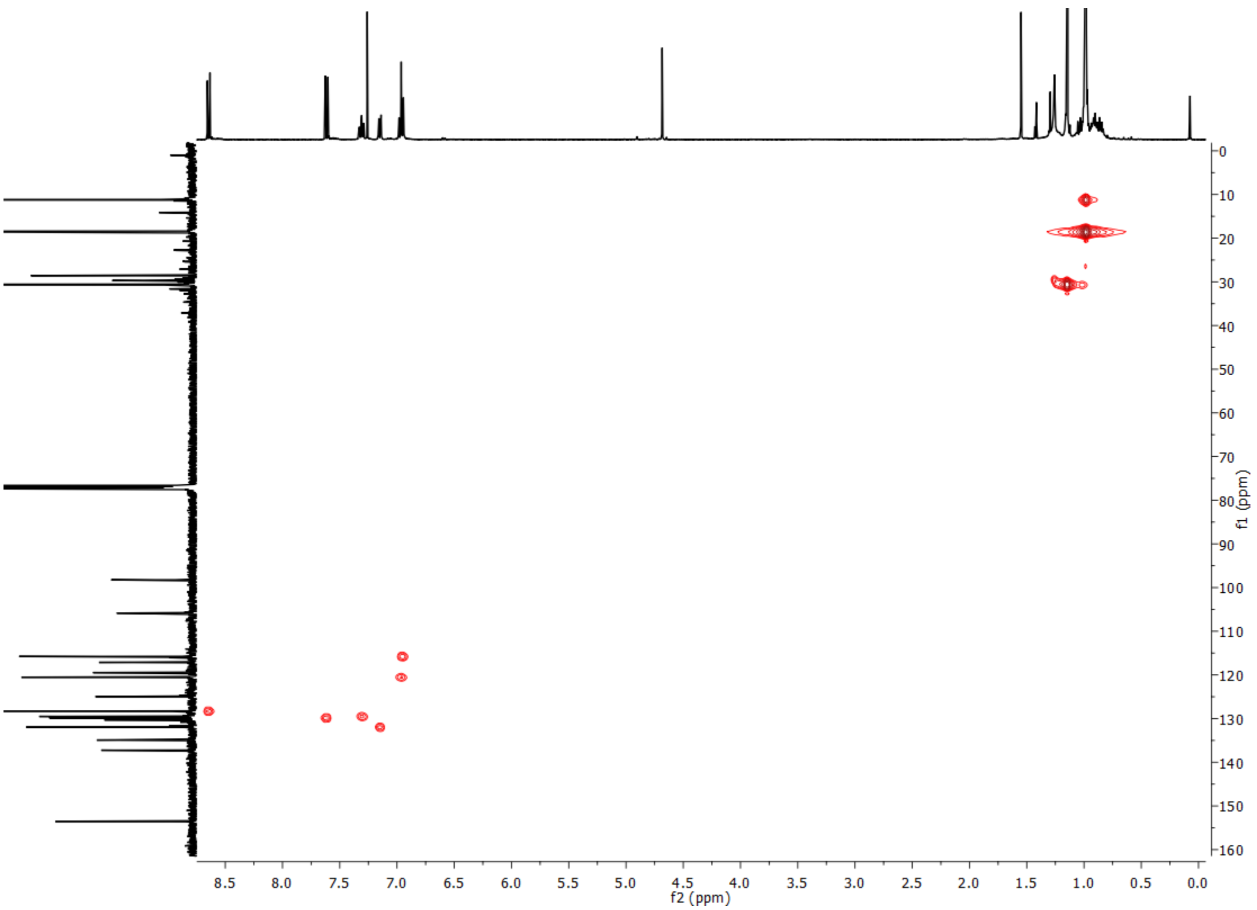

Supplementary Figure 38. HSQC NMR (400 MHz) of **6** in $CDCl_3$, measured at 298 K.

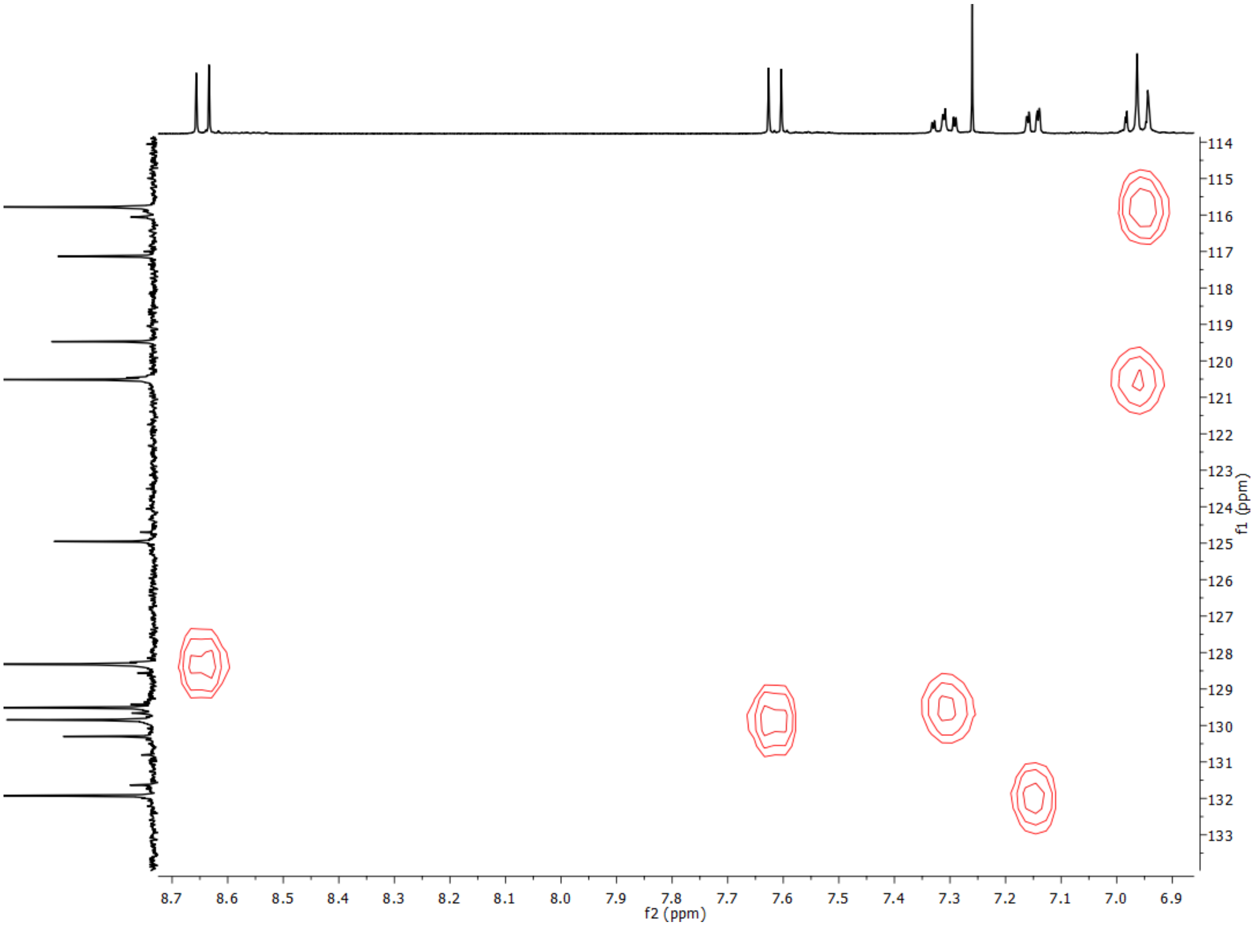

Supplementary Figure 39. HSQC NMR (500 MHz) of **6** in $CDCl_3$, measured at 298 K (expansion in aromatic region).

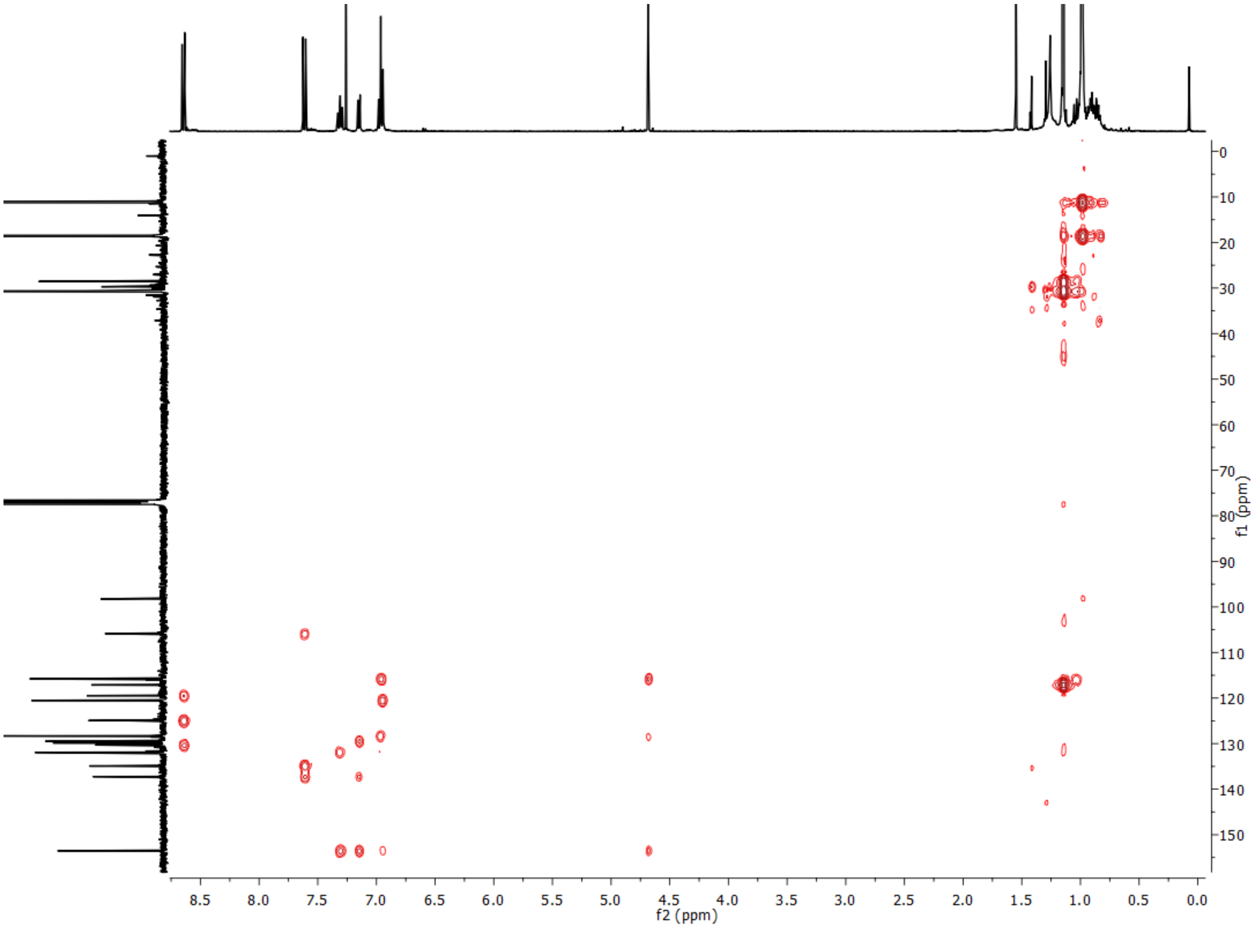

Supplementary Figure 40. HMBC NMR (500 MHz) of **6** in $CDCl_3$, measured at 298 K.

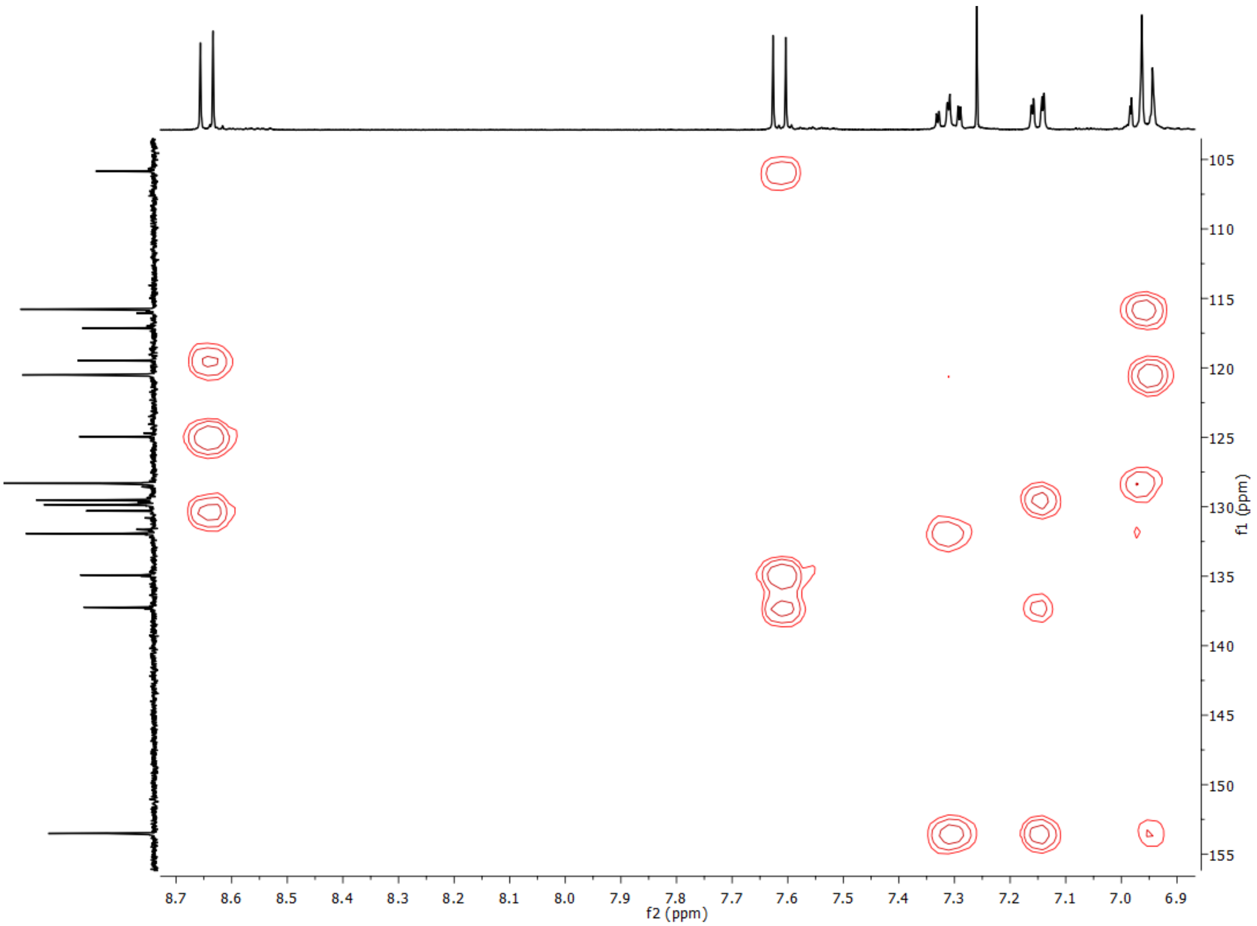

Supplementary Figure 41. HMBC NMR (500 MHz) of **6** in $CDCl_3$, measured at 298 K (expansion in aromatic region).

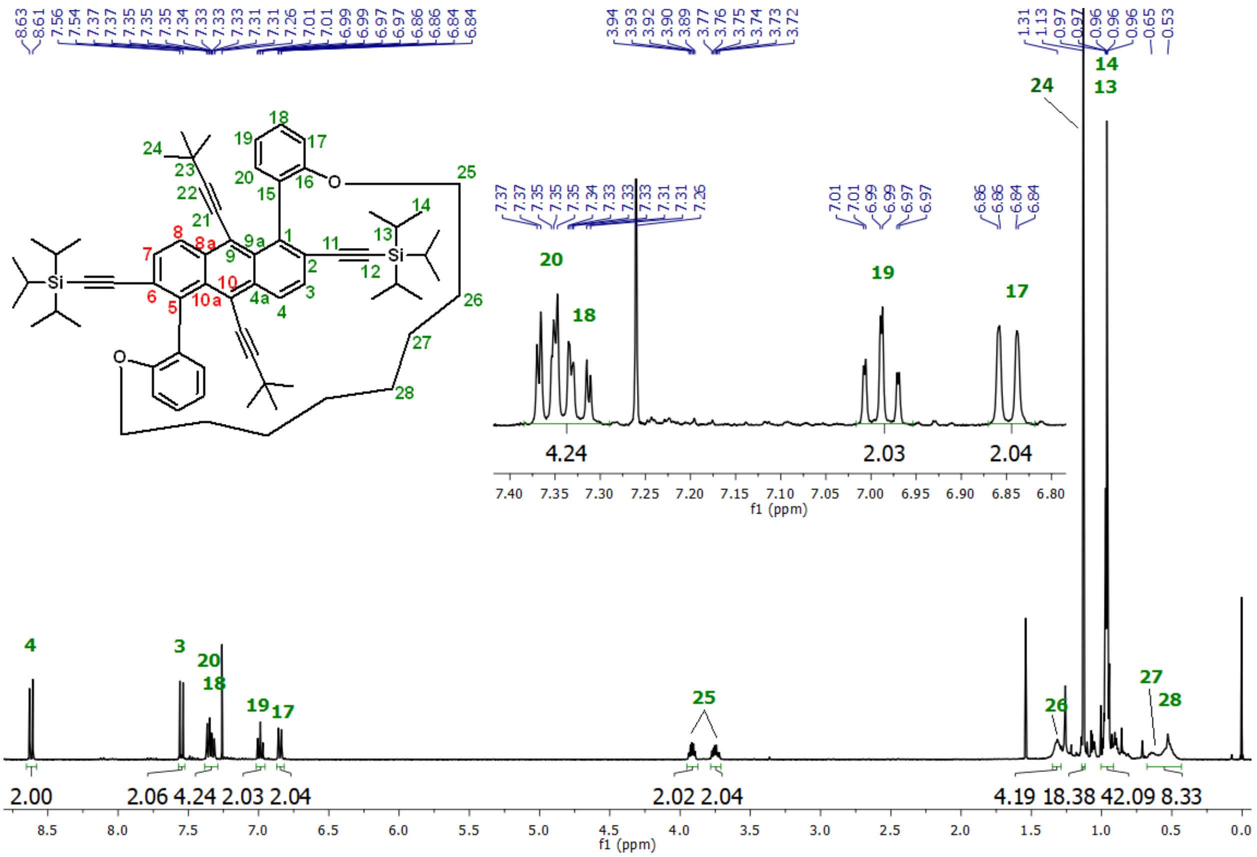

Supplementary Figure 42. ^{1}H NMR (400 MHz) of **7-C8** in $CDCl_3$, measured at 298 K.

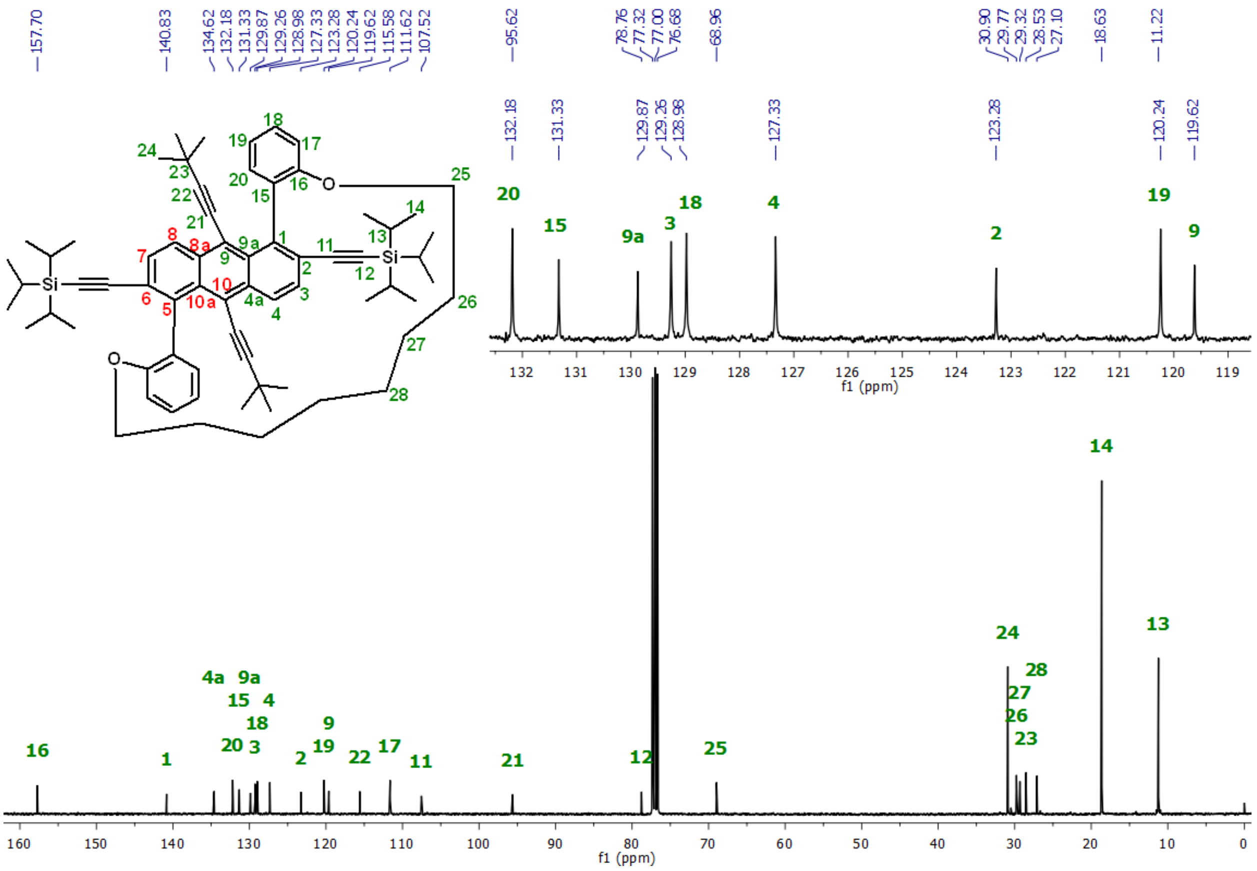

Supplementary Figure 43. ^{13}C NMR (101 MHz) of **7-C8** in $CDCl_3$, measured at 298 K.

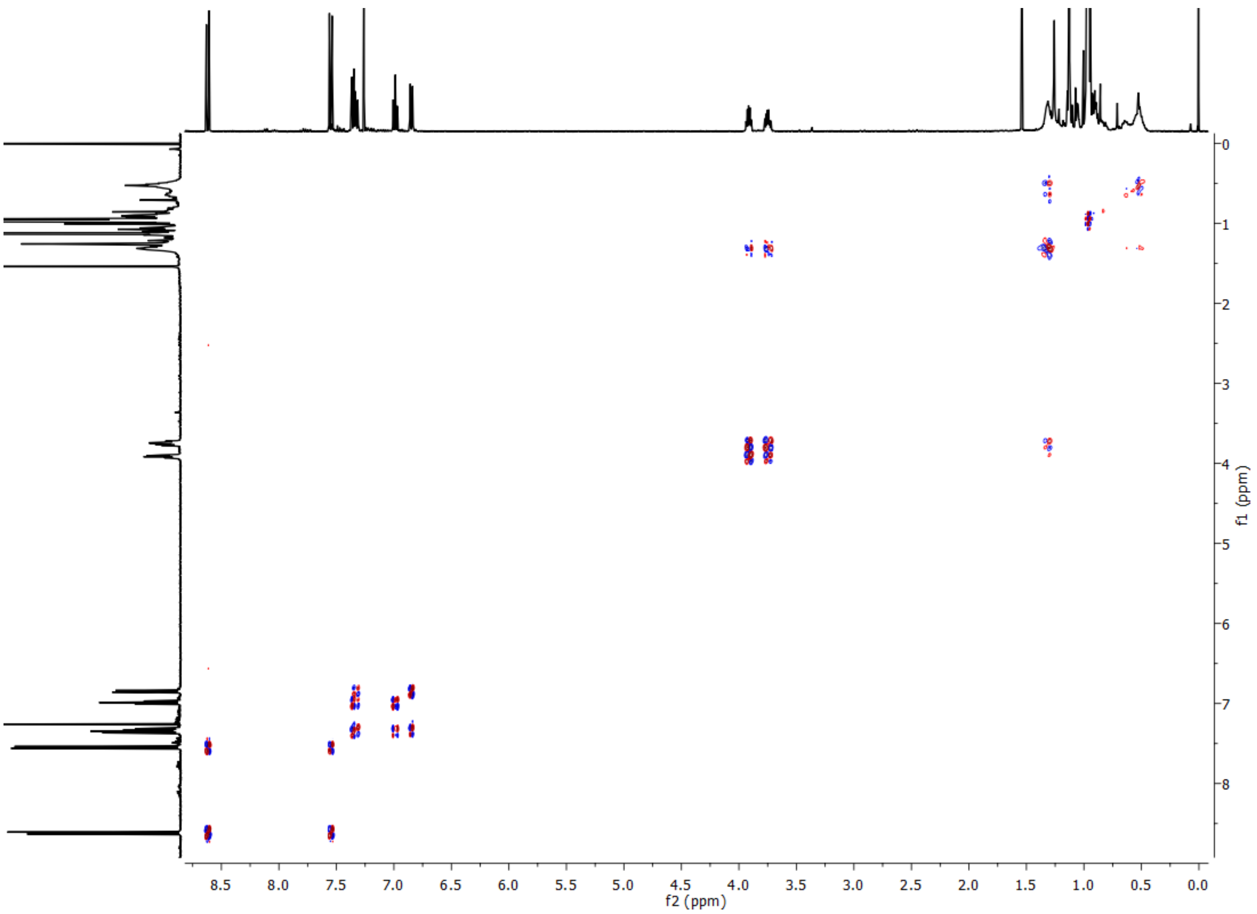

Supplementary Figure 44. COSY NMR (400 MHz) of **7-C8** in $CDCl_3$, measured at 298 K.

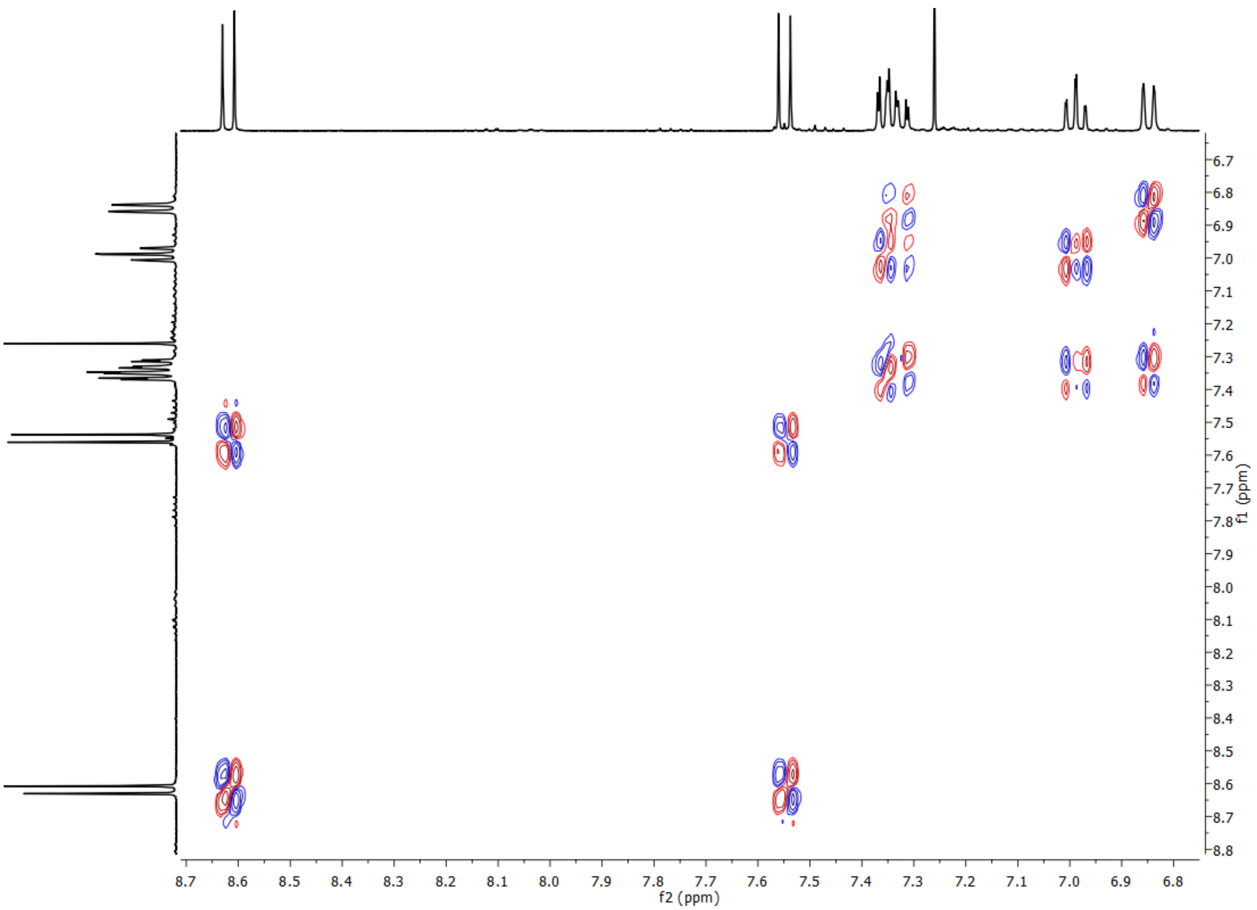

Supplementary Figure 45. COSY NMR (400 MHz) of **7-C8** in $CDCl_3$, measured at 298 K (expansion in aromatic region).

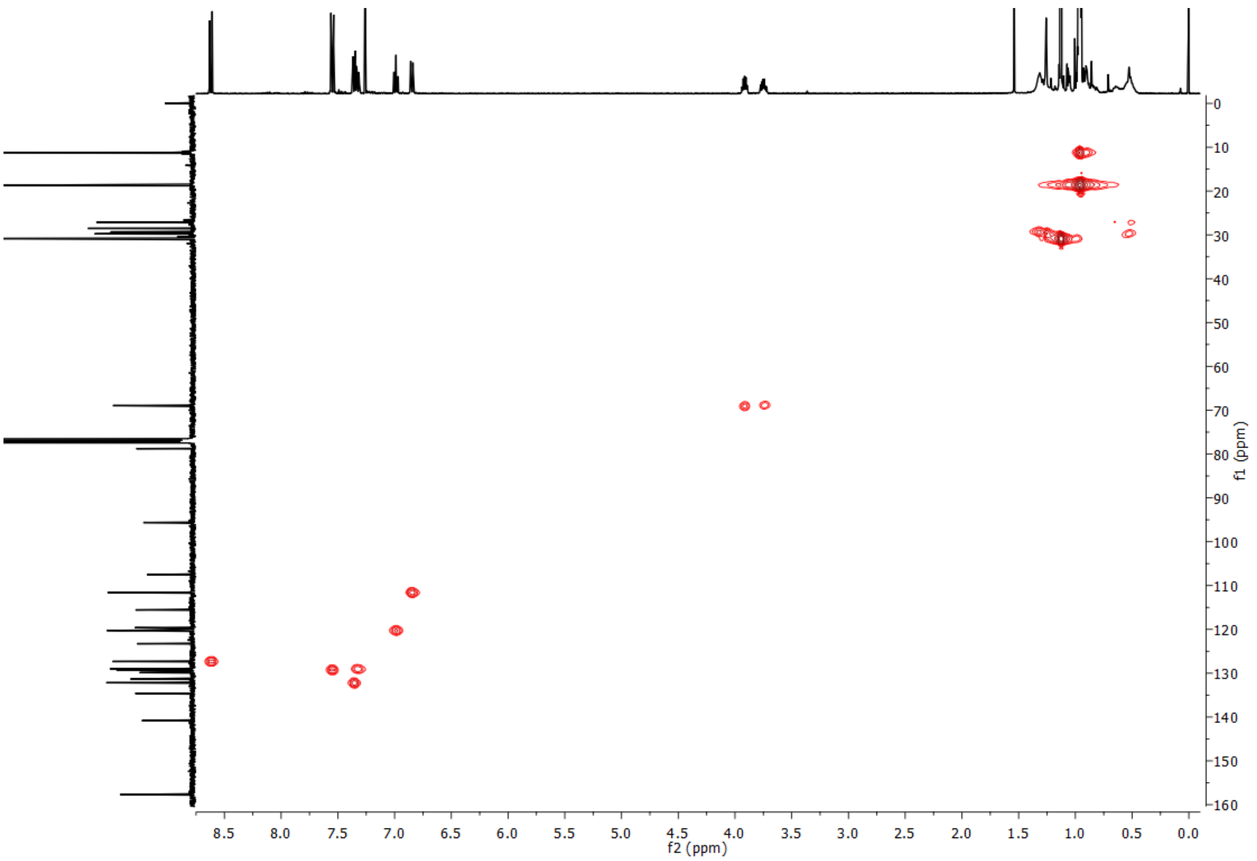

Supplementary Figure 46. HSQC NMR (400 MHz) of **7-C8** in $CDCl_3$, measured at 298 K.

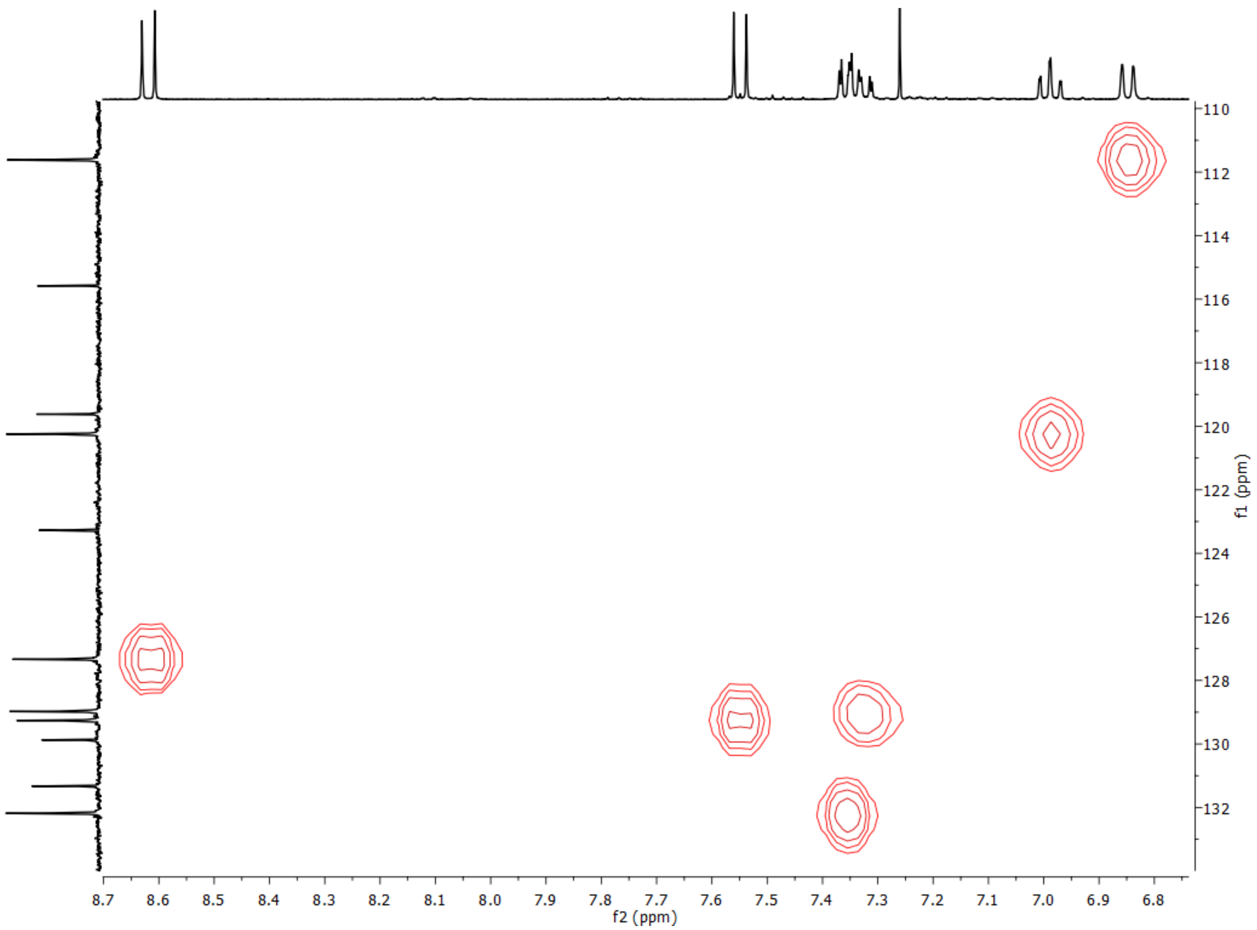

Supplementary Figure 47. HSQC NMR (400 MHz) of **7-C8** in $CDCl_3$, measured at 298 K (expansion in aromatic region).

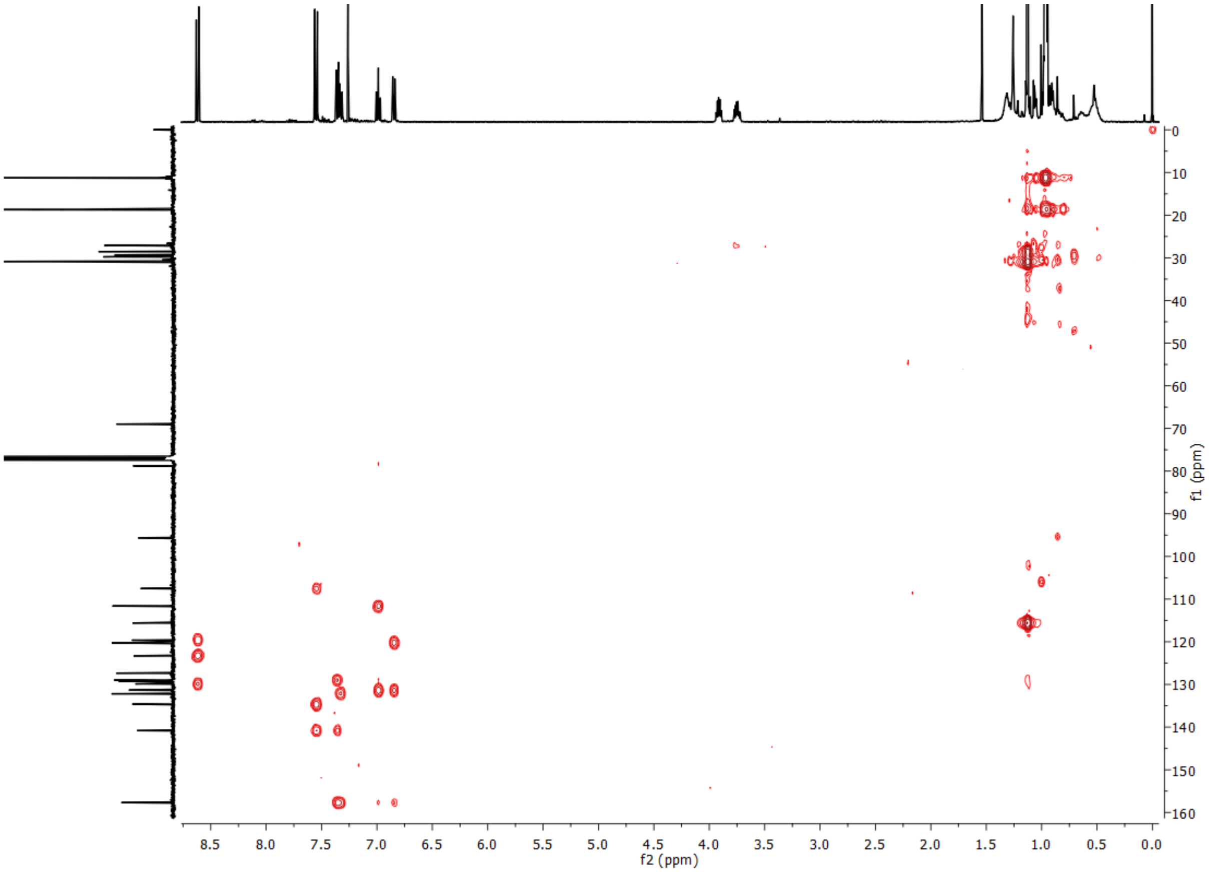

Supplementary Figure 48. HMBC NMR (400 MHz) of **7-C8** in $CDCl_3$, measured at 298 K.

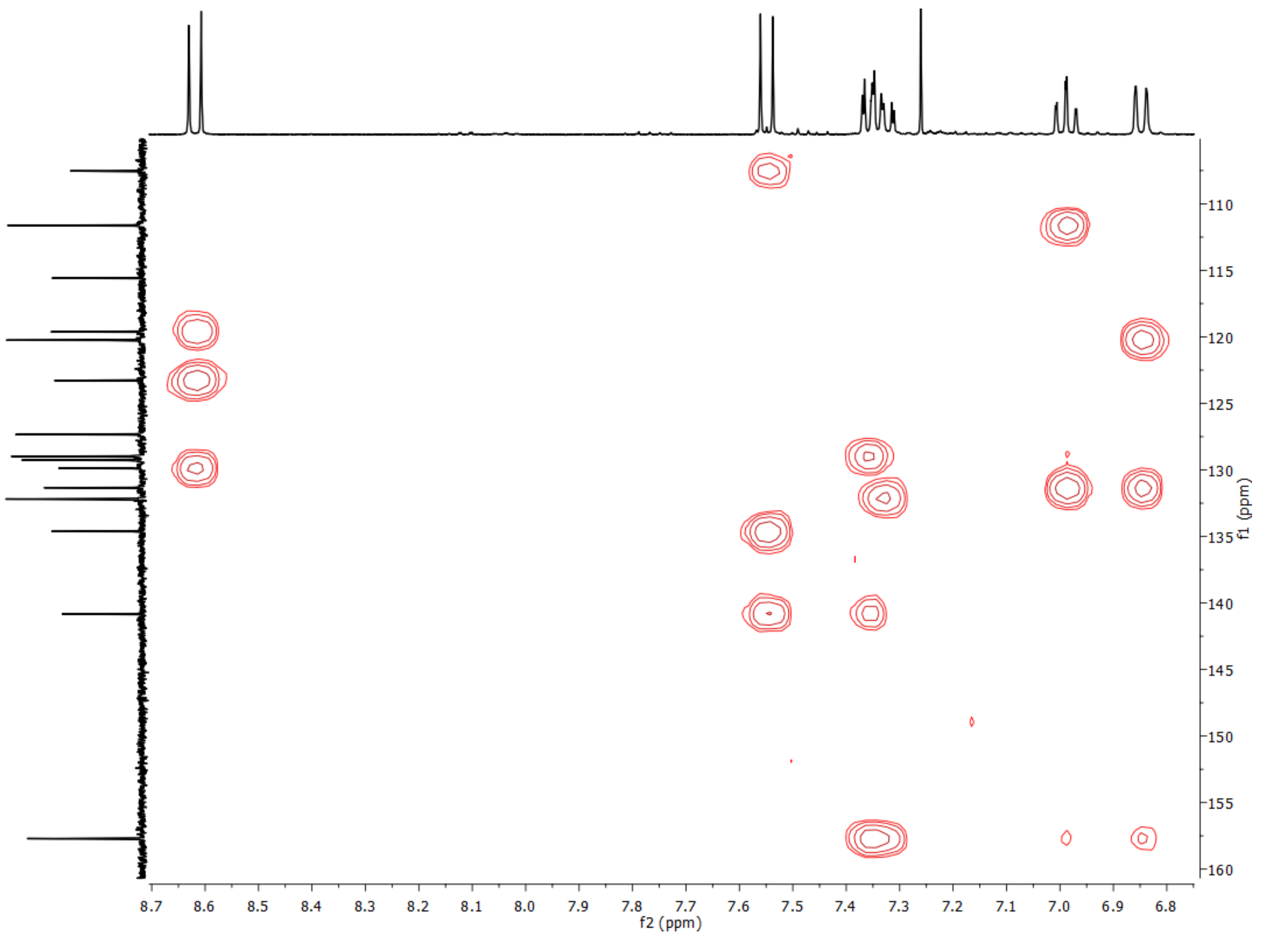

Supplementary Figure 49. HMBC NMR (400 MHz) of **7-C8** in $CDCl_3$, measured at 298 K (expansion in aromatic region).

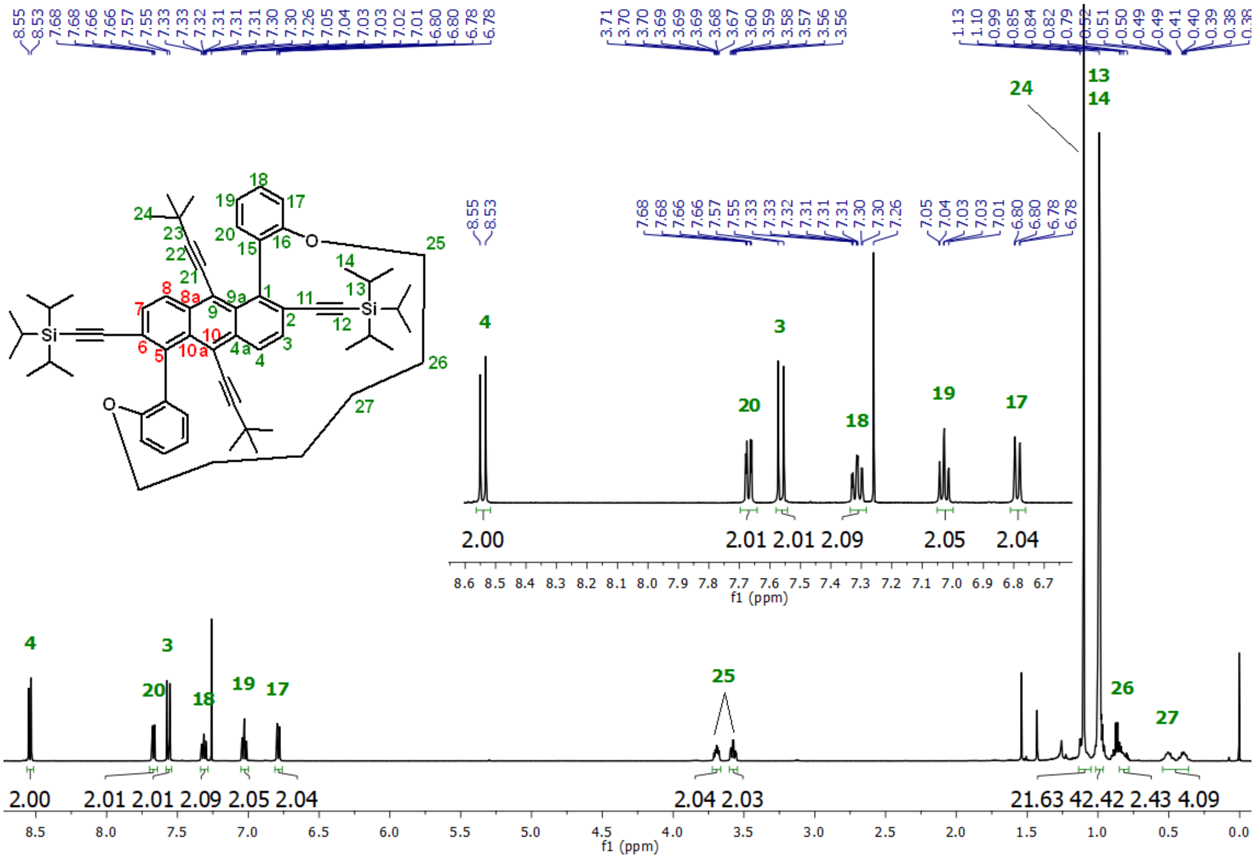

Supplementary Figure 50. ^{1}H NMR (500 MHz) of **7-C6** in $CDCl_3$, measured at 298 K.

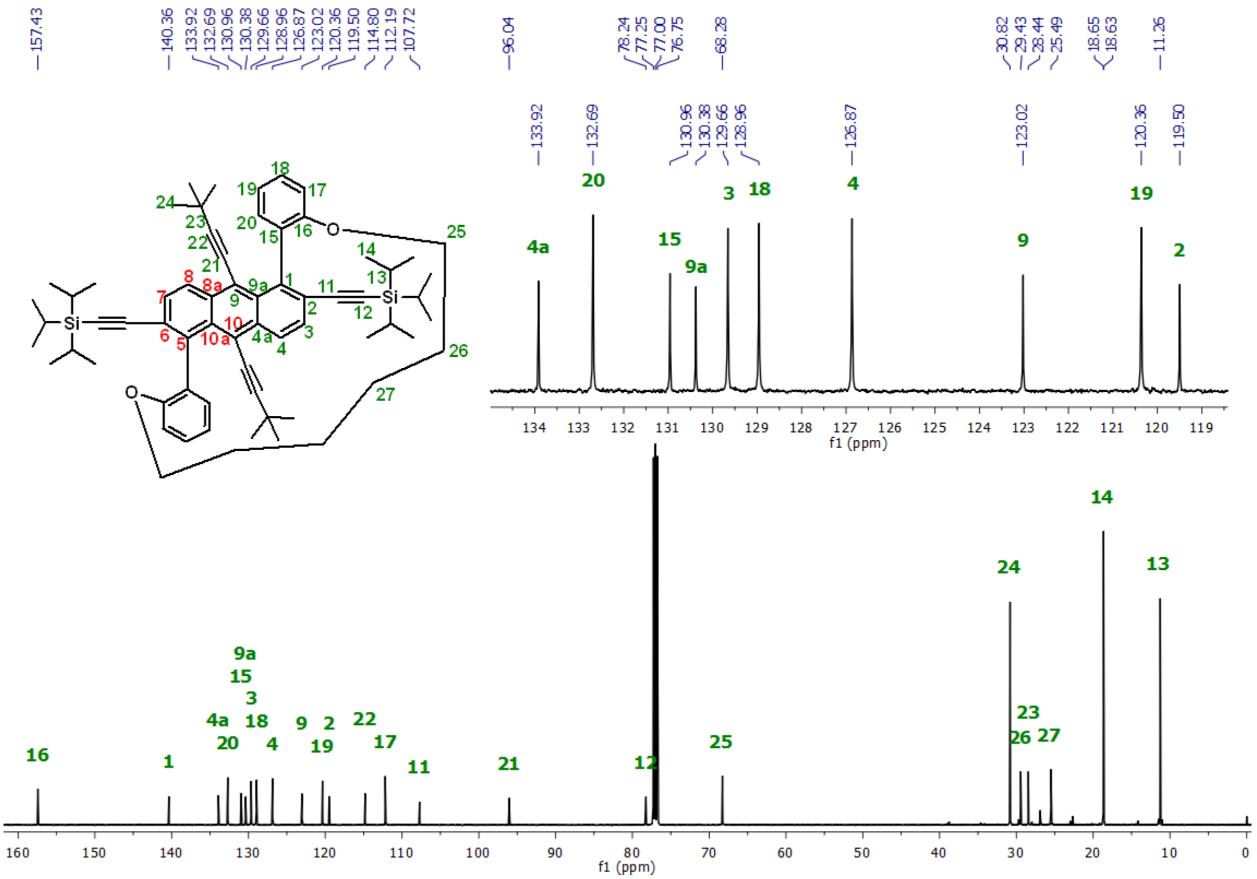

Supplementary Figure 51. ^{13}C NMR (126 MHz) of **7-C6** in $CDCl_3$, measured at 298 K.

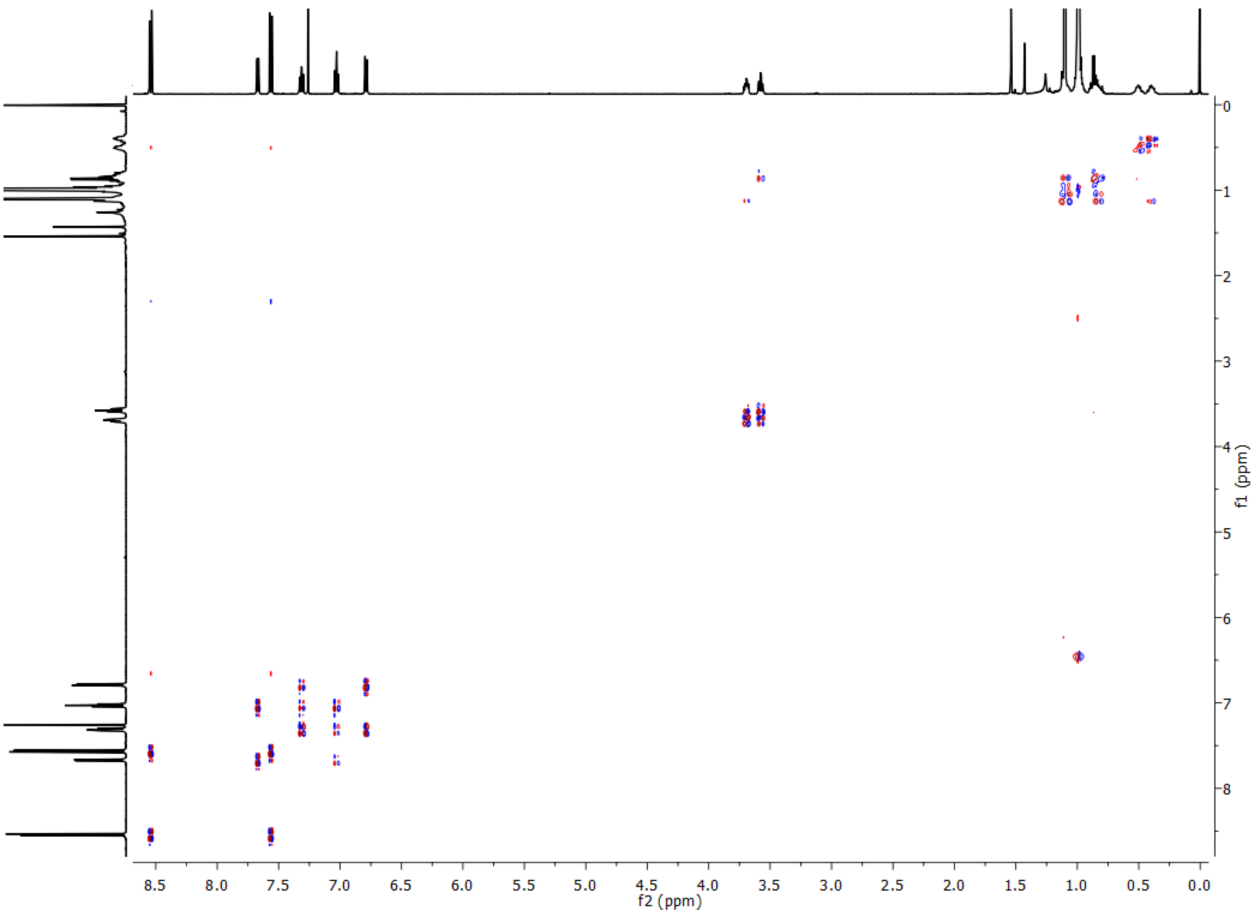

Supplementary Figure 52. COSY NMR (500 MHz) of **7-C6** in $CDCl_3$, measured at 298 K.

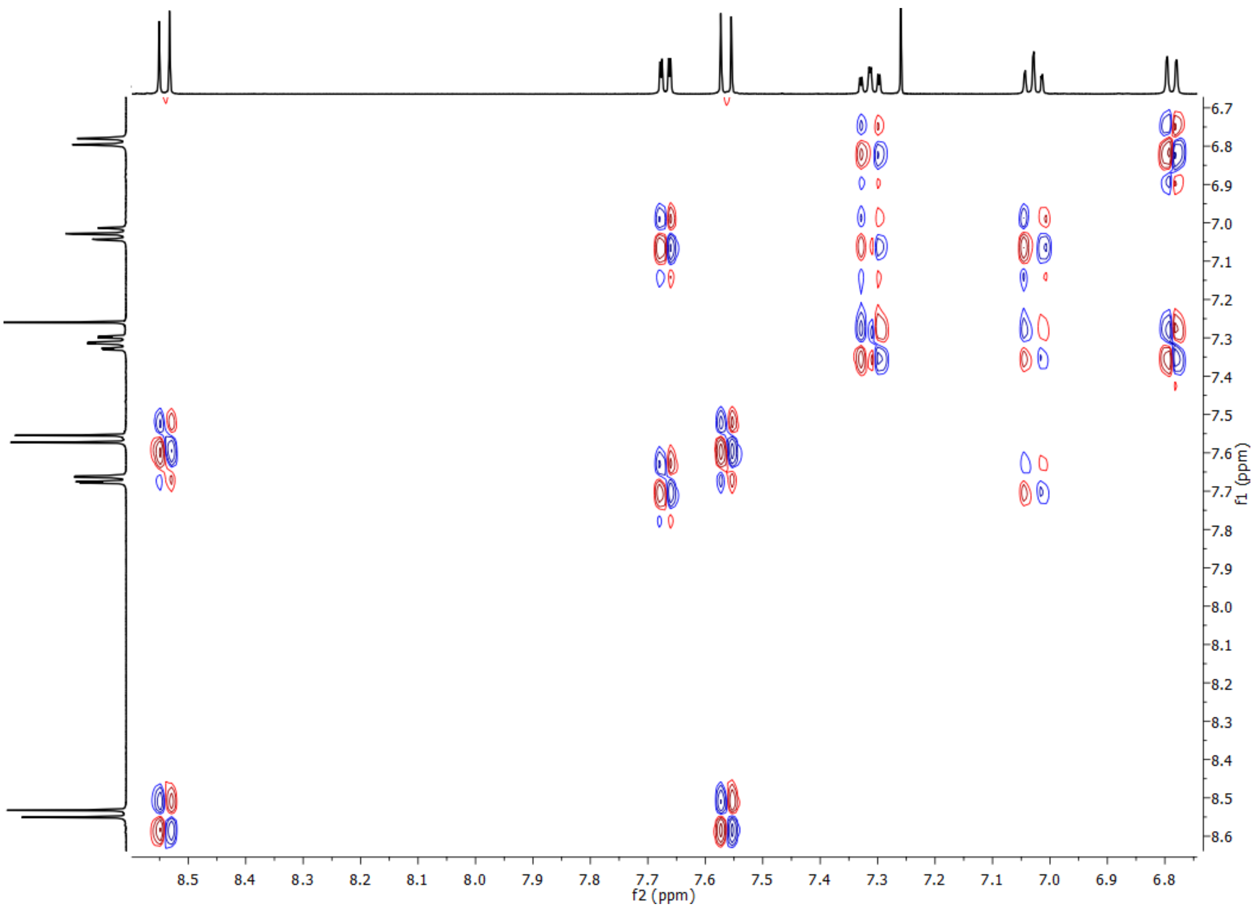

Supplementary Figure 53. COSY NMR (500 MHz) of **7-C6** in $CDCl_3$, measured at 298 K (expansion in aromatic region).

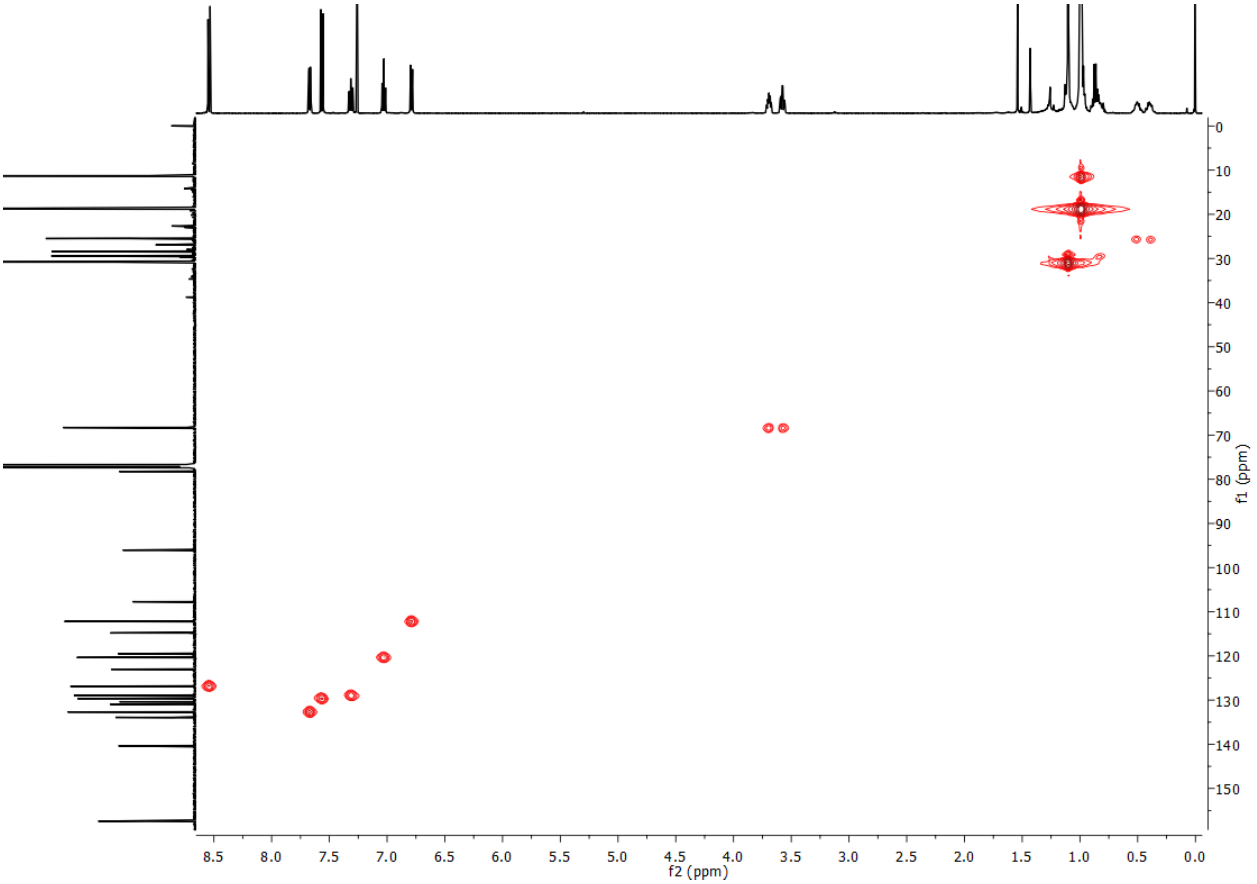

Supplementary Figure 54. HSQC NMR (500 MHz) of **7-C6** in $CDCl_3$, measured at 298 K.

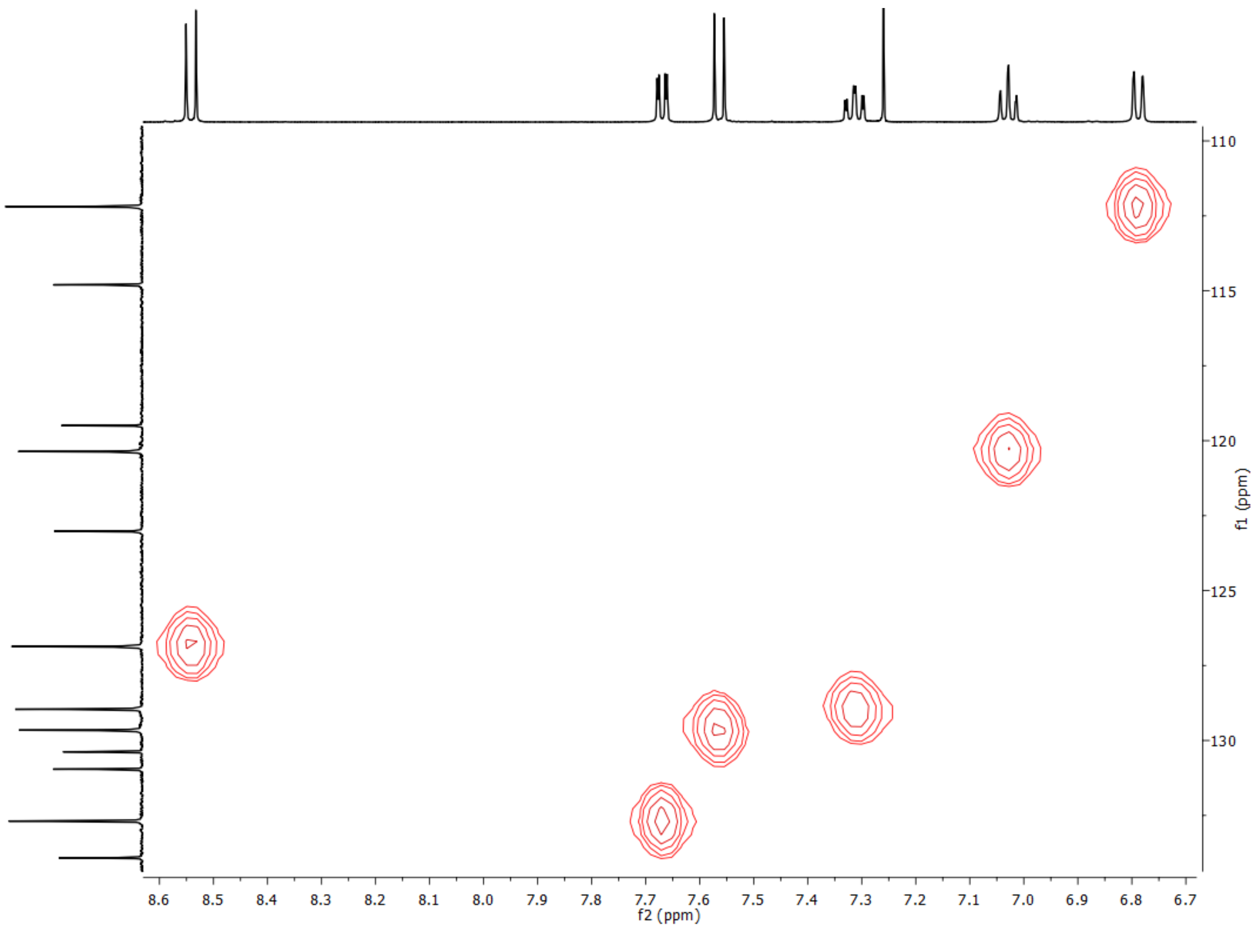

Supplementary Figure 55. HSQC NMR (500 MHz) of **7-C6** in $CDCl_3$, measured at 298 K (expansion in aromatic region).

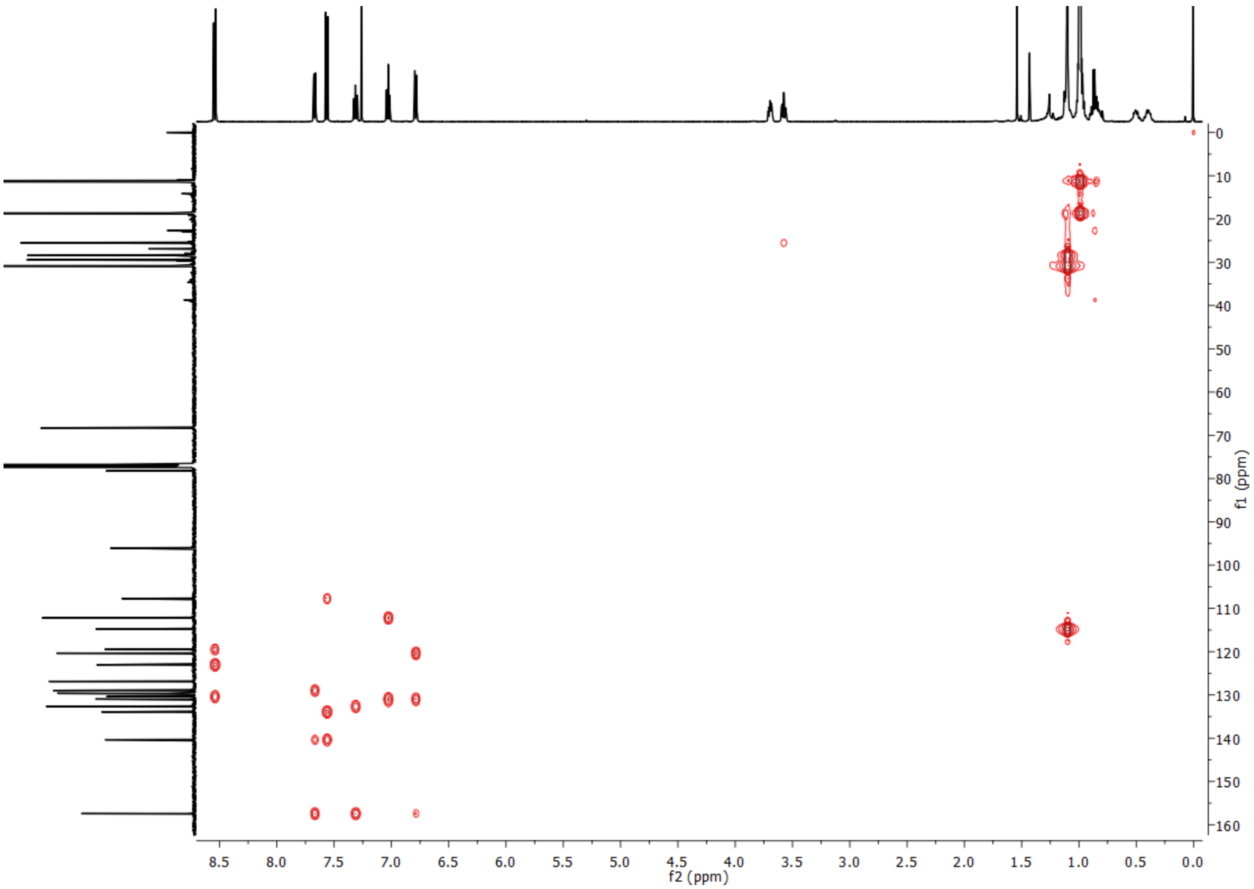

Supplementary Figure 56. HMBC NMR (500 MHz) of **7-C6** in $CDCl_3$, measured at 298 K.

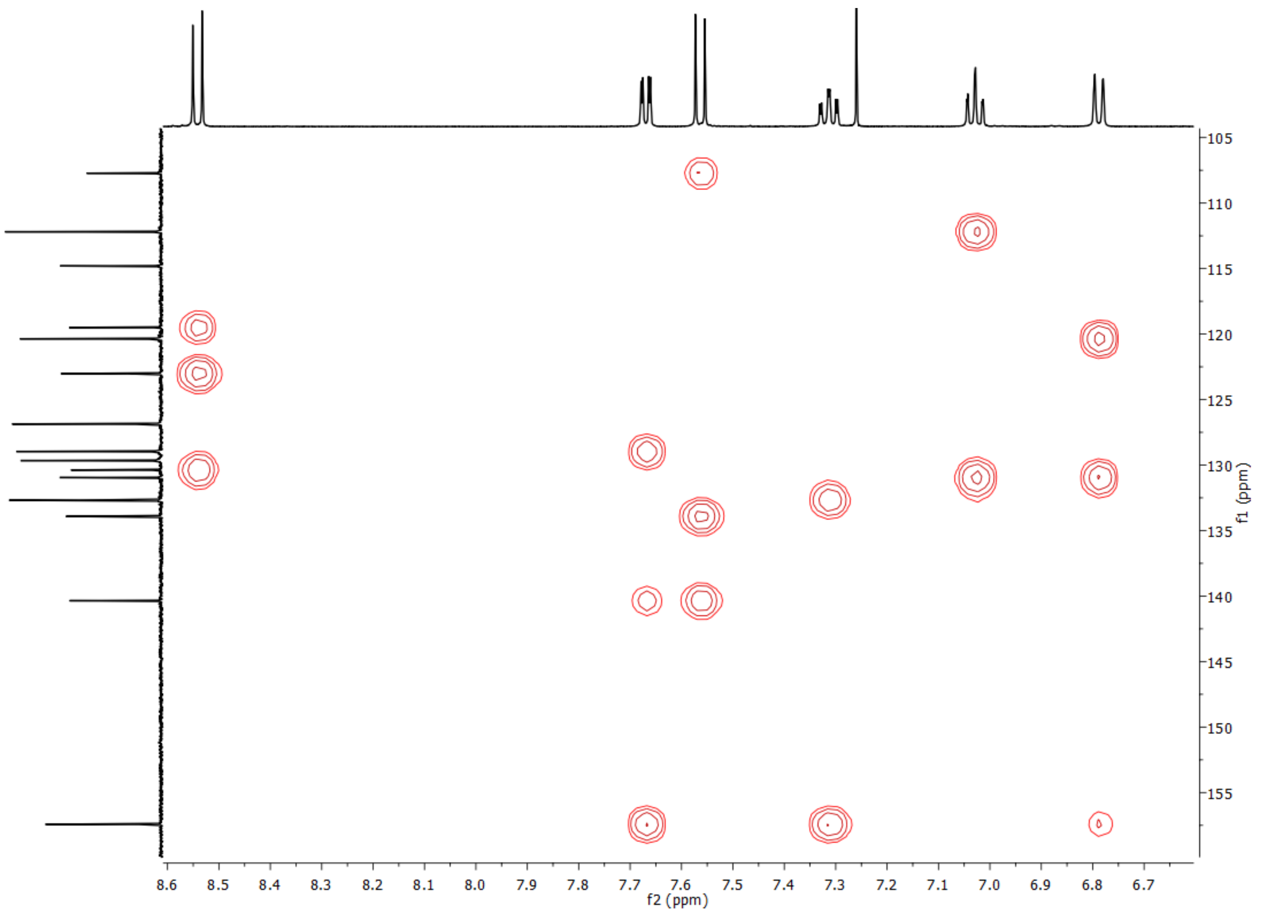

Supplementary Figure 57. HMBC NMR (500 MHz) of **7-C6** in $CDCl_3$, measured at 298 K (expansion in aromatic region).

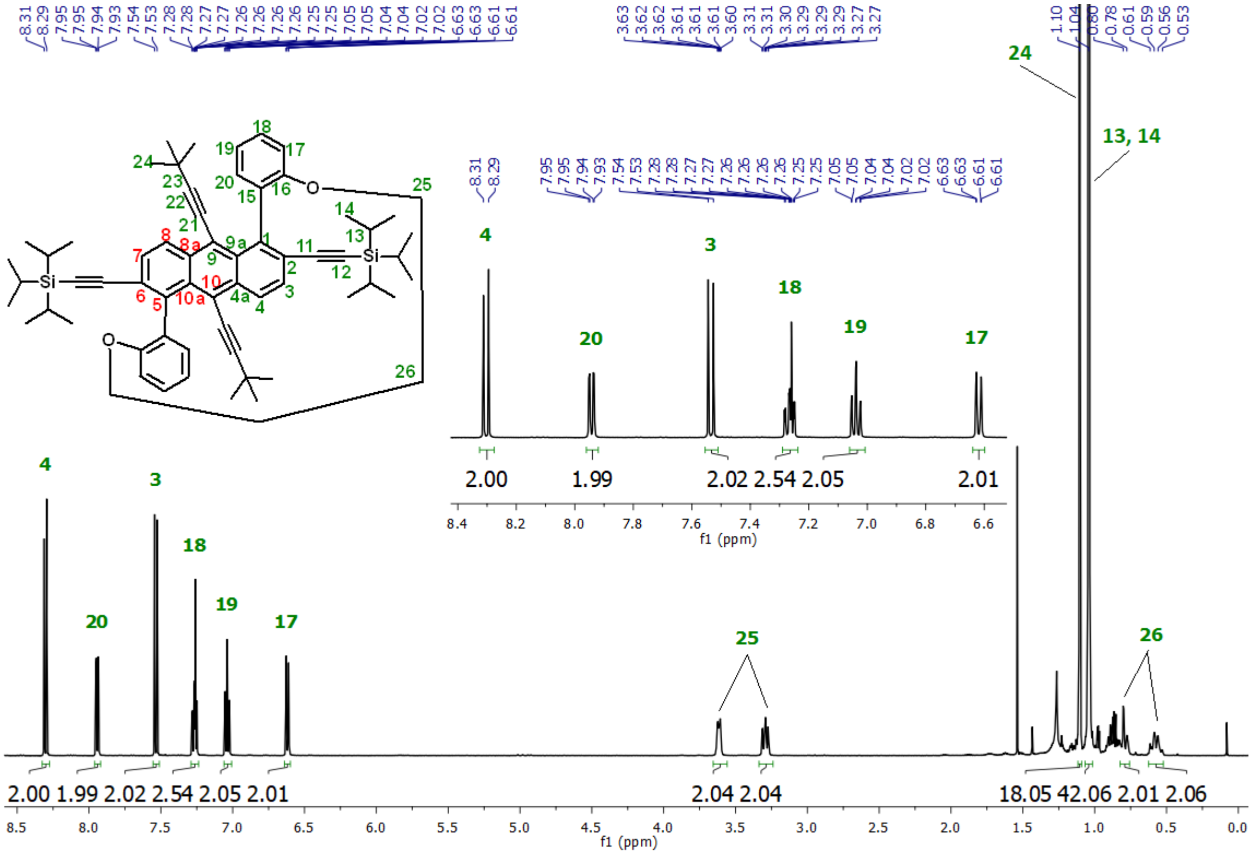

Supplementary Figure 58. ^{1}H NMR (500 MHz) of **7-C4** in $CDCl_3$, measured at 298 K.

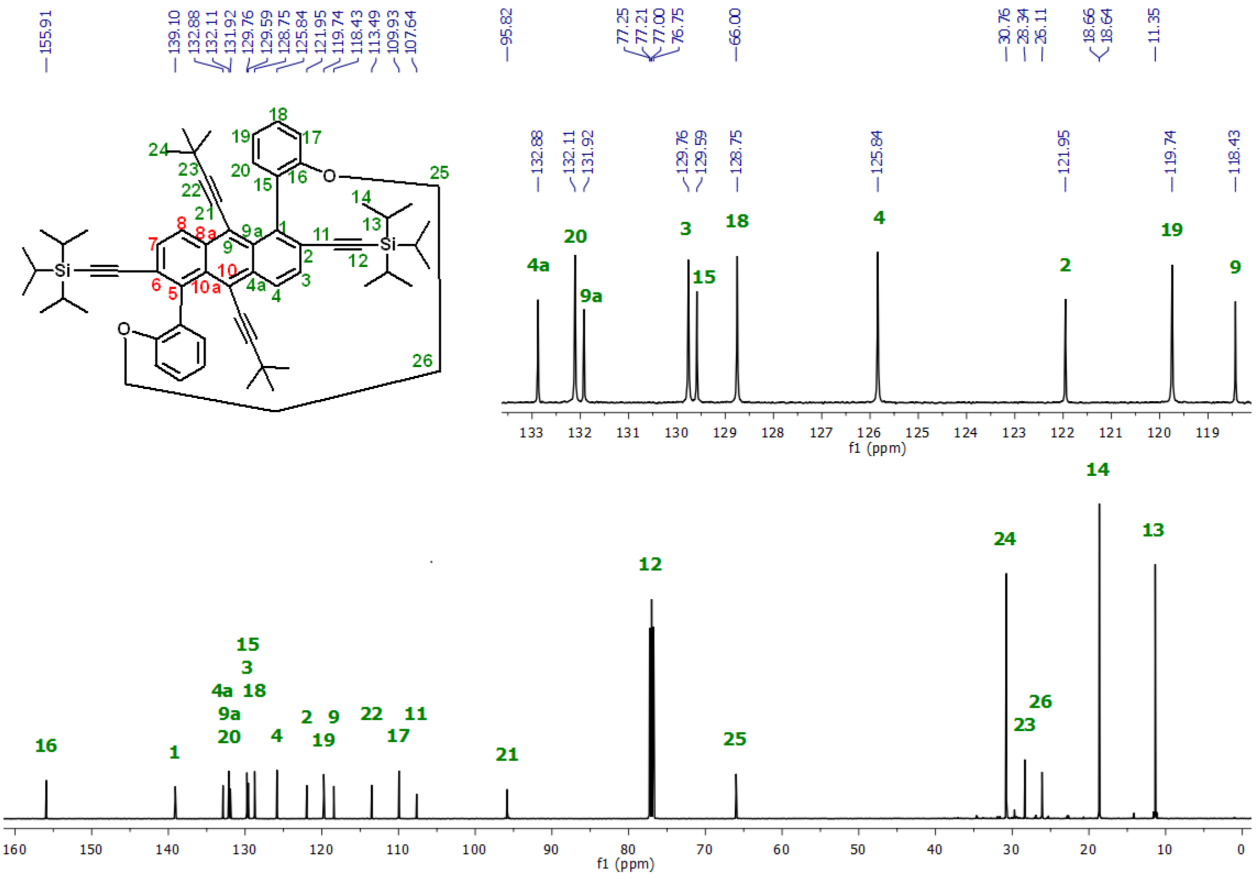

Supplementary Figure 59. ^{13}C NMR (126 MHz) of **7-C4** in $CDCl_3$, measured at 298 K.

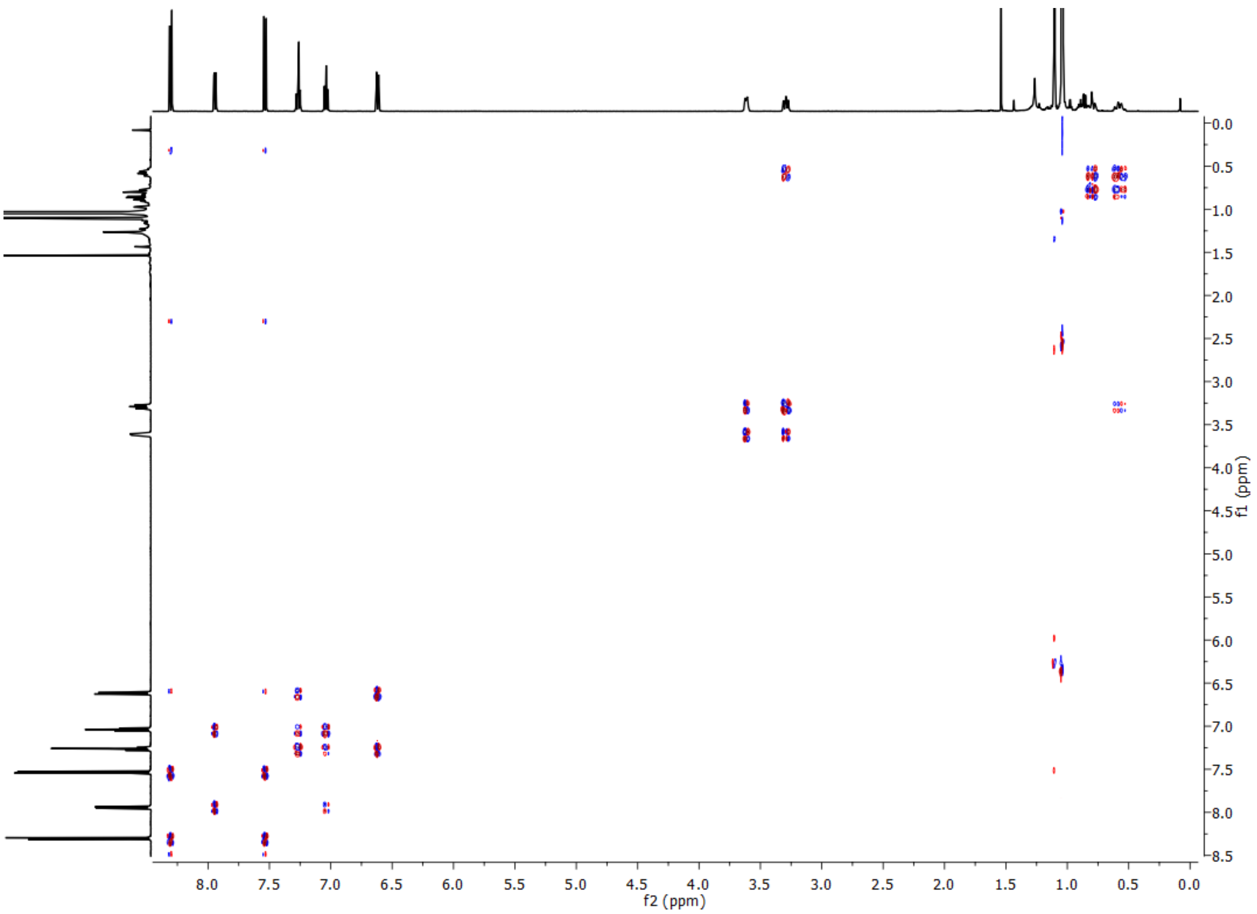

Supplementary Figure 60. COSY NMR (500 MHz) of **7-C4** in $CDCl_3$, measured at 298 K.

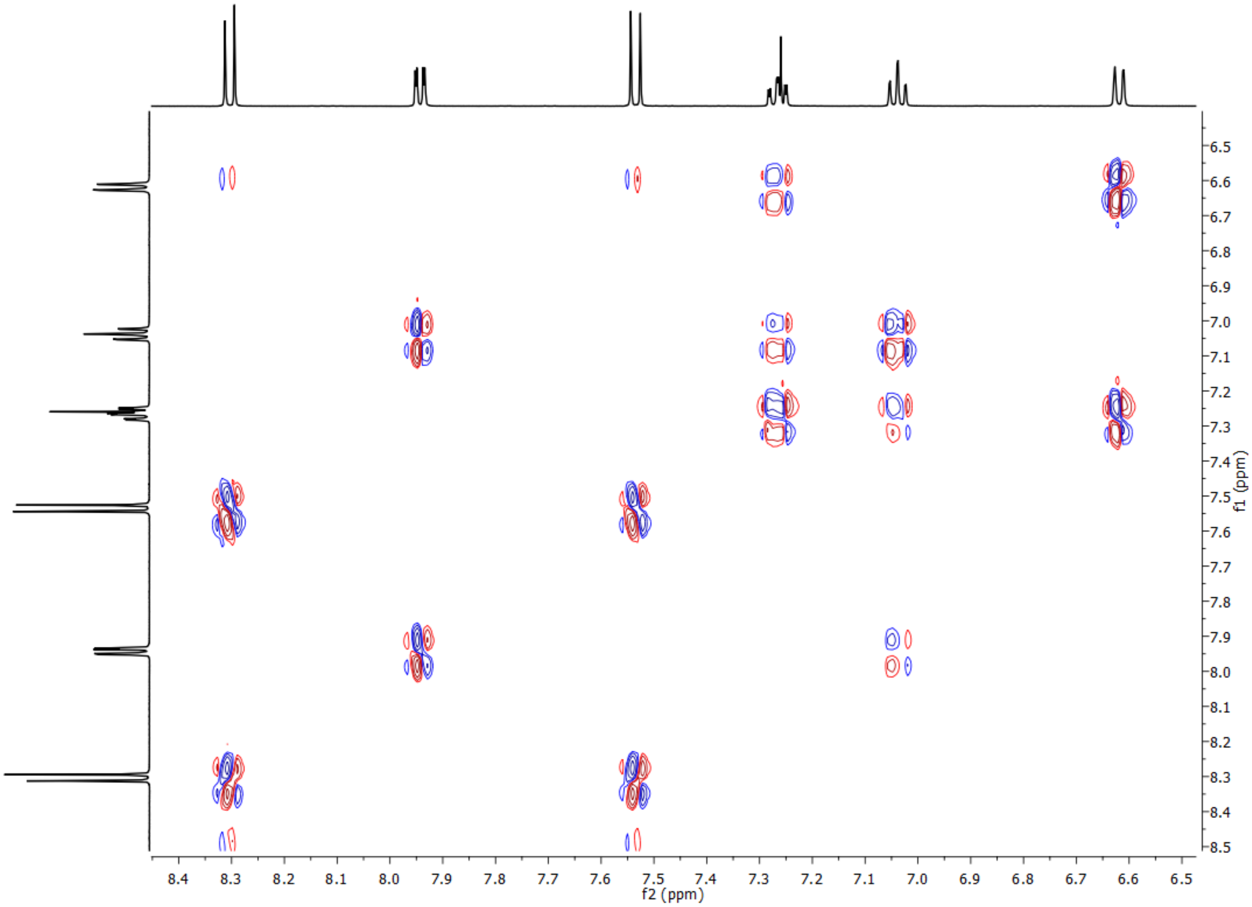

Supplementary Figure 61. COSY NMR (500 MHz) of **7-C4** in $CDCl_3$, measured at 298 K (expansion in aromatic region).

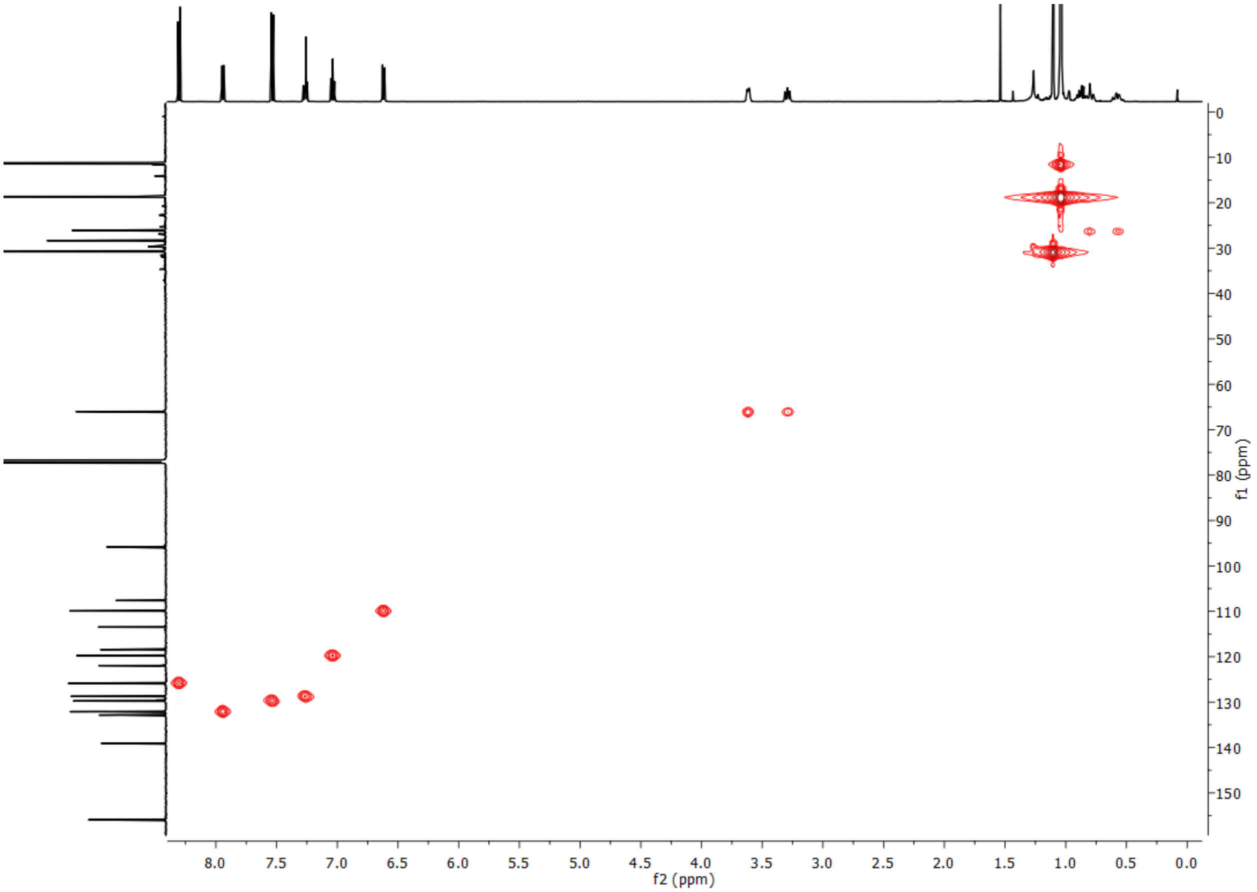

Supplementary Figure 62. HSQC NMR (500 MHz) of **7-C4** in $CDCl_3$, measured at 298 K

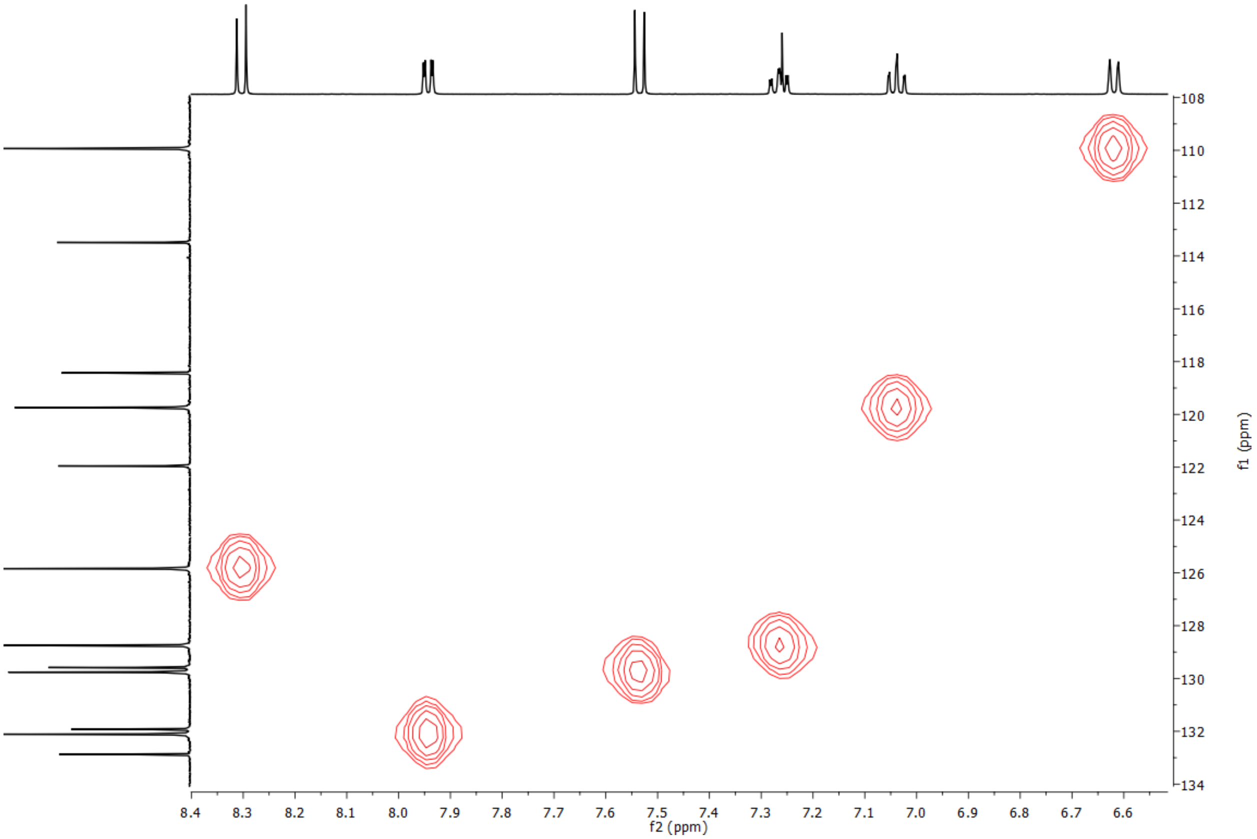

Supplementary Figure 63. HSQC NMR (500 MHz) of **7-C4** in $CDCl_3$, measured at 298 K (expansion in aromatic region).

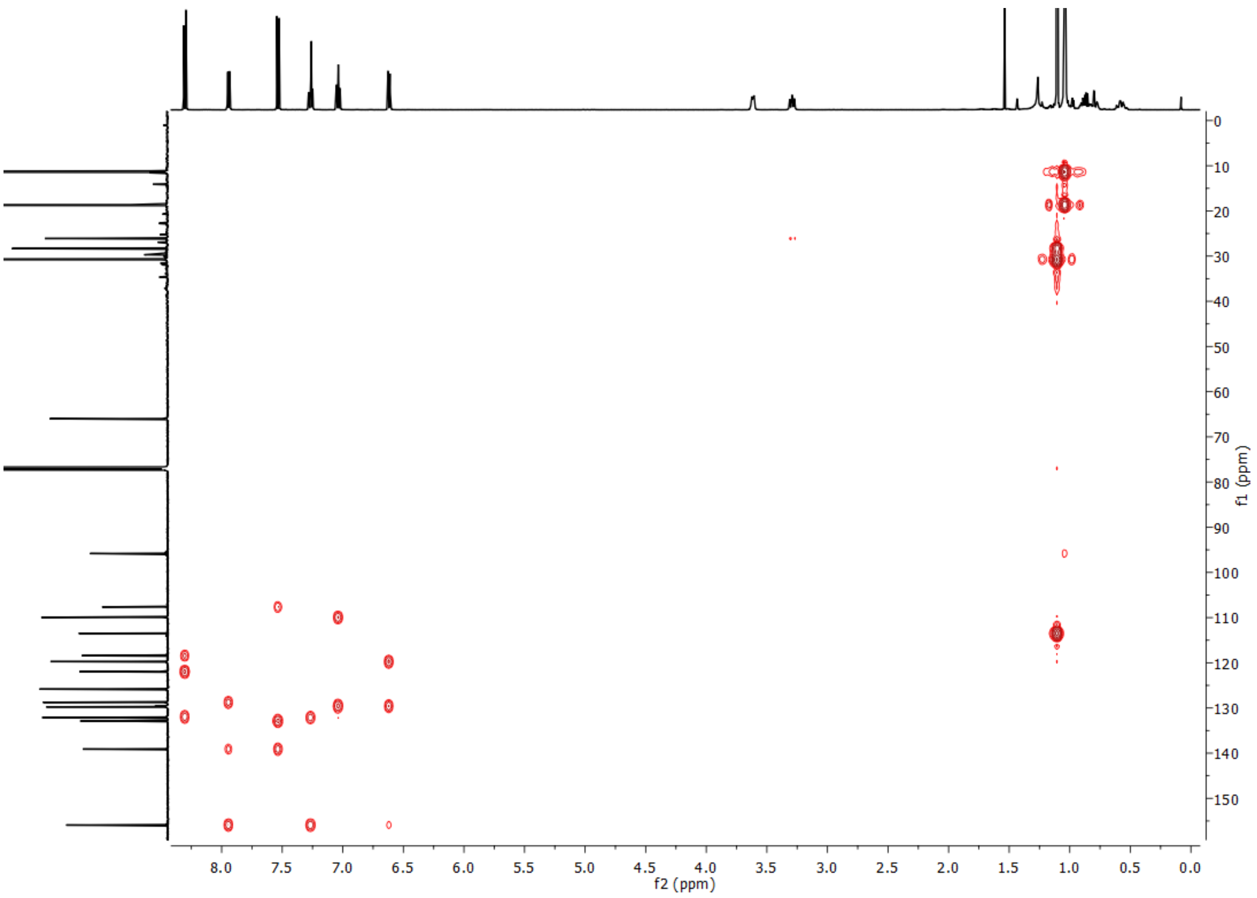

Supplementary Figure 64. HMBC NMR (500 MHz) of **7-C4** in $CDCl_3$, measured at 298 K.

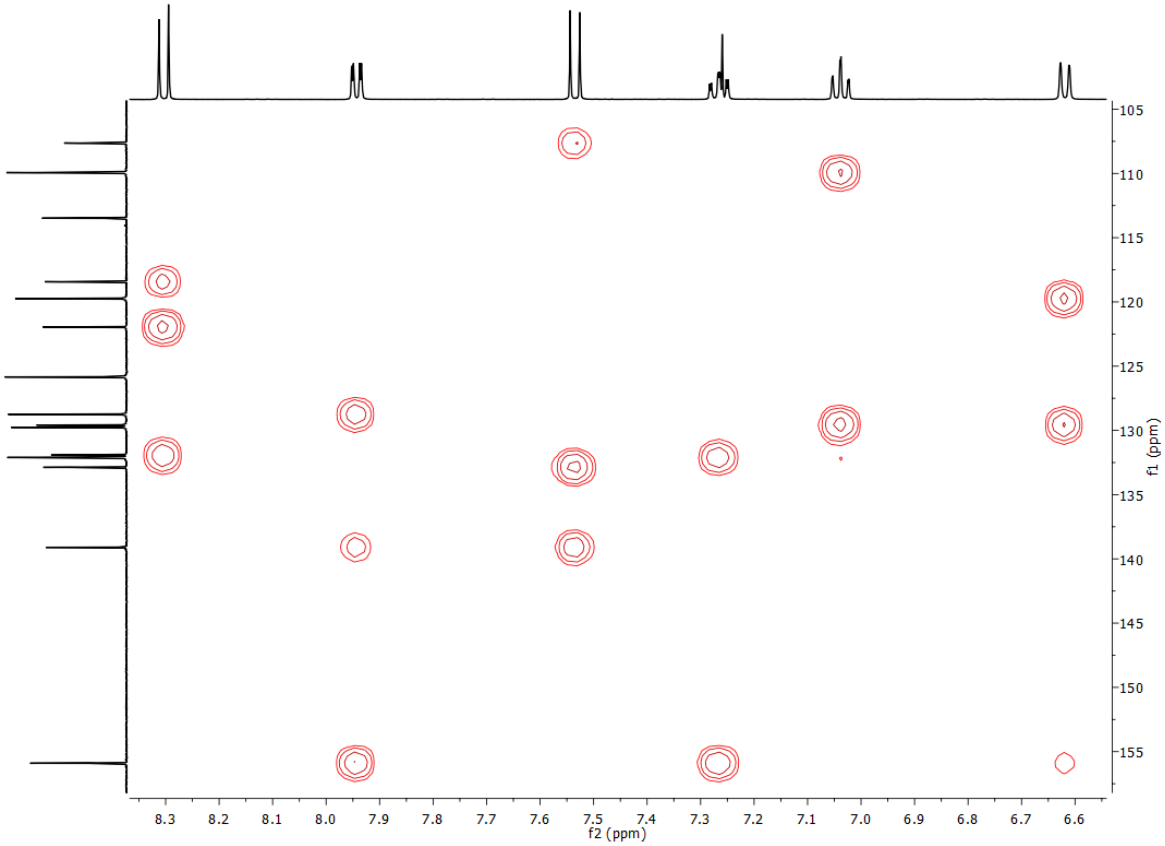

Supplementary Figure 65. HMBC NMR (500 MHz) of **7-C4** in $CDCl_3$, measured at 298 K (expansion in aromatic region).

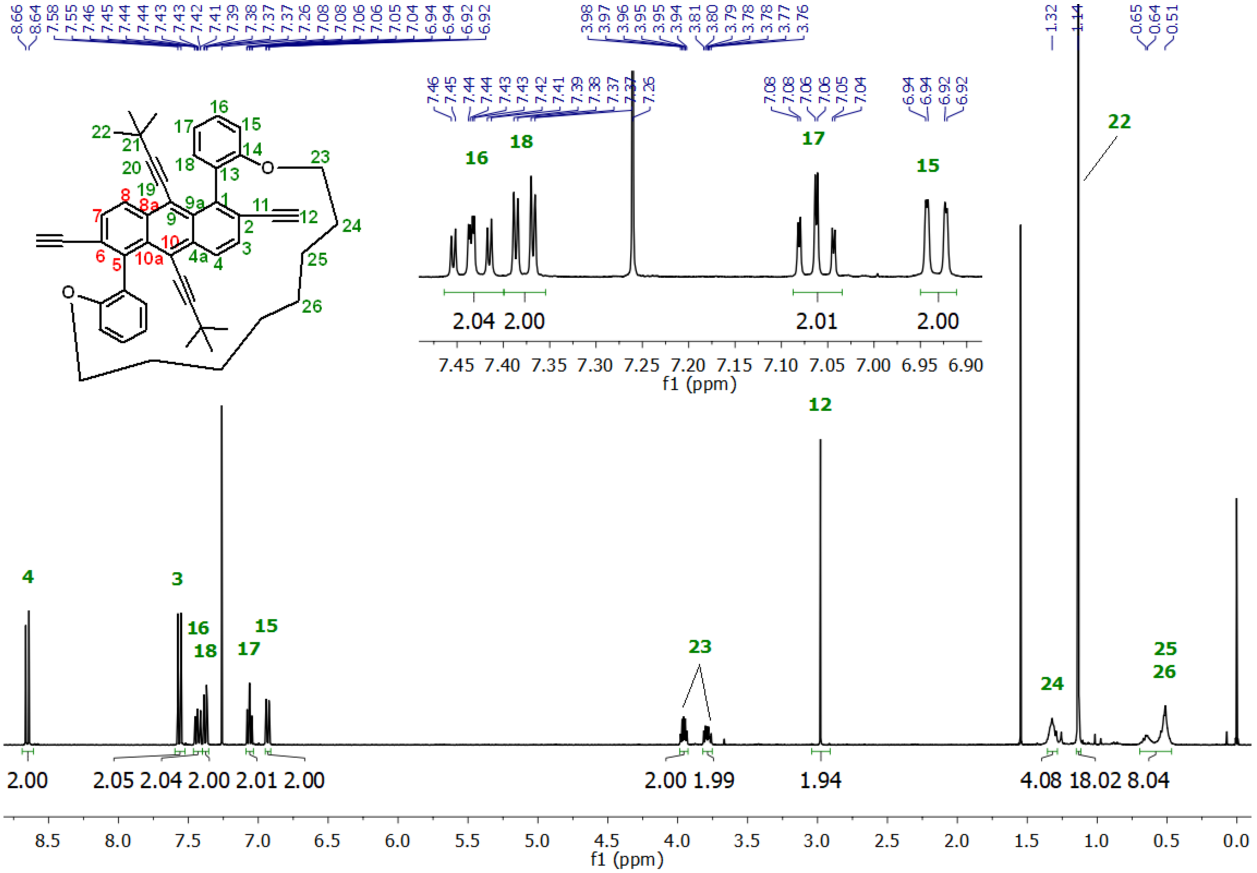

Supplementary Figure 66. ^{1}H NMR (400 MHz) of **8-C8** in $CDCl_3$, measured at 298 K.

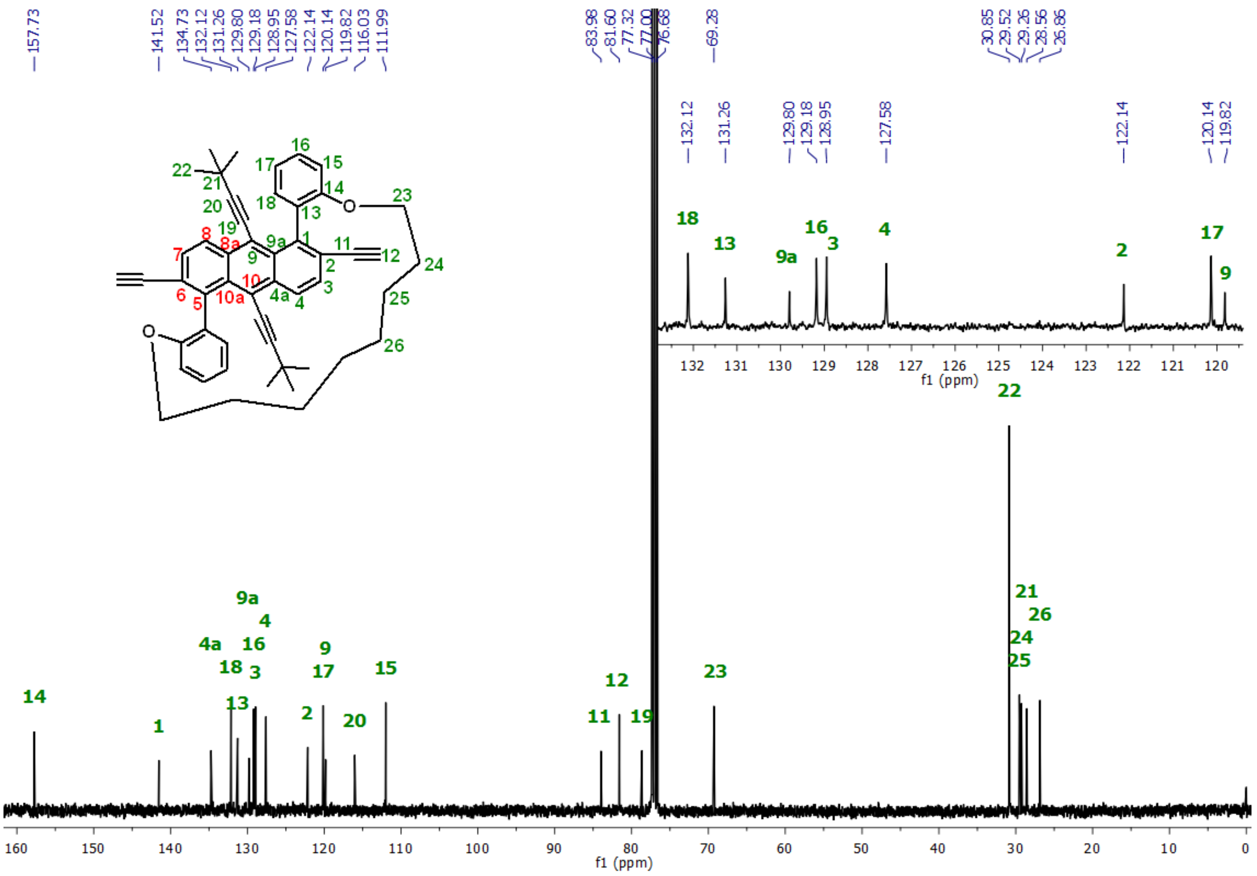

Supplementary Figure 67. ^{13}C NMR (101 MHz) of **8-C8** in $CDCl_3$, measured at 298 K.

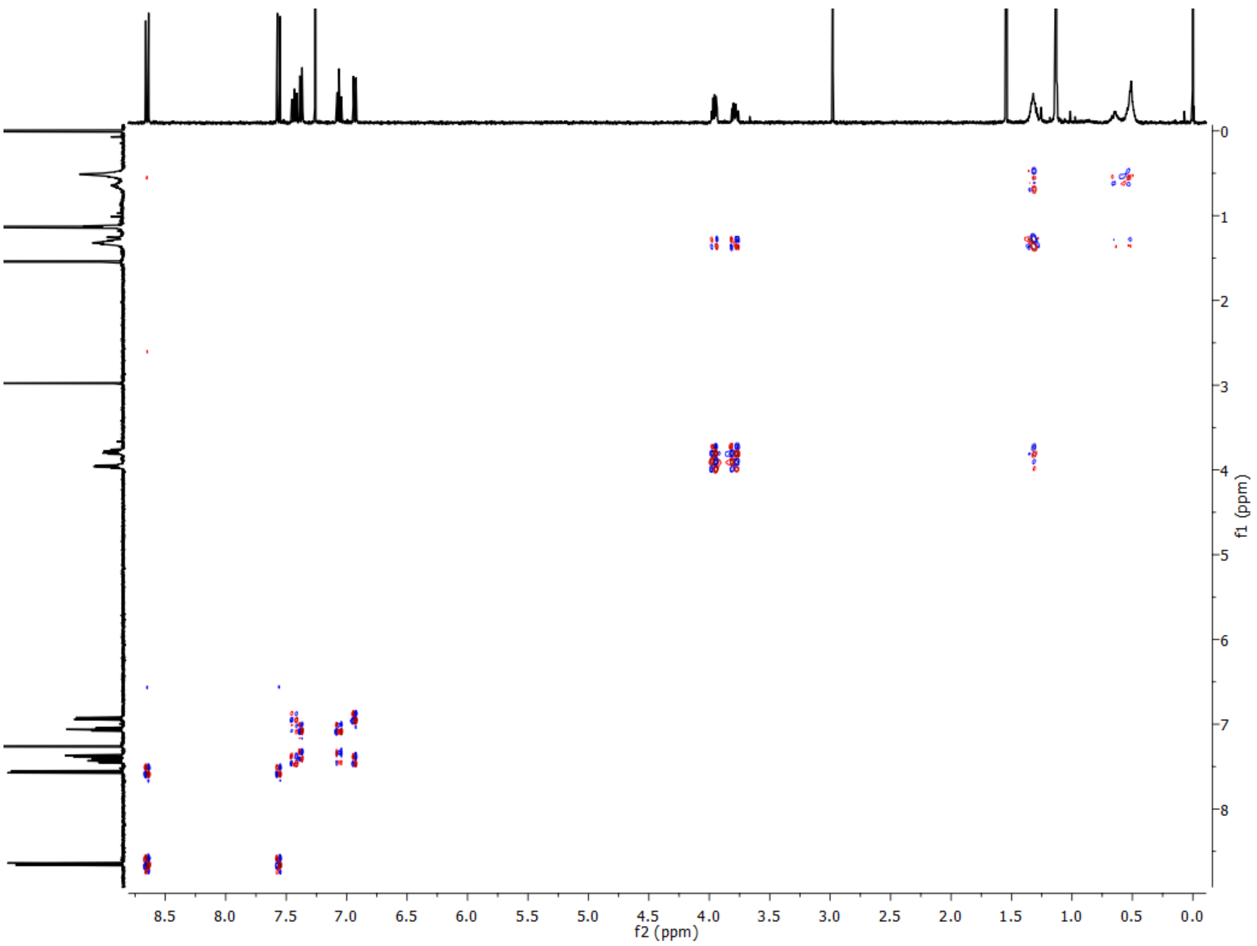

Supplementary Figure 68. COSY NMR (400 MHz) of **8-C8** in $CDCl_3$, measured at 298 K.

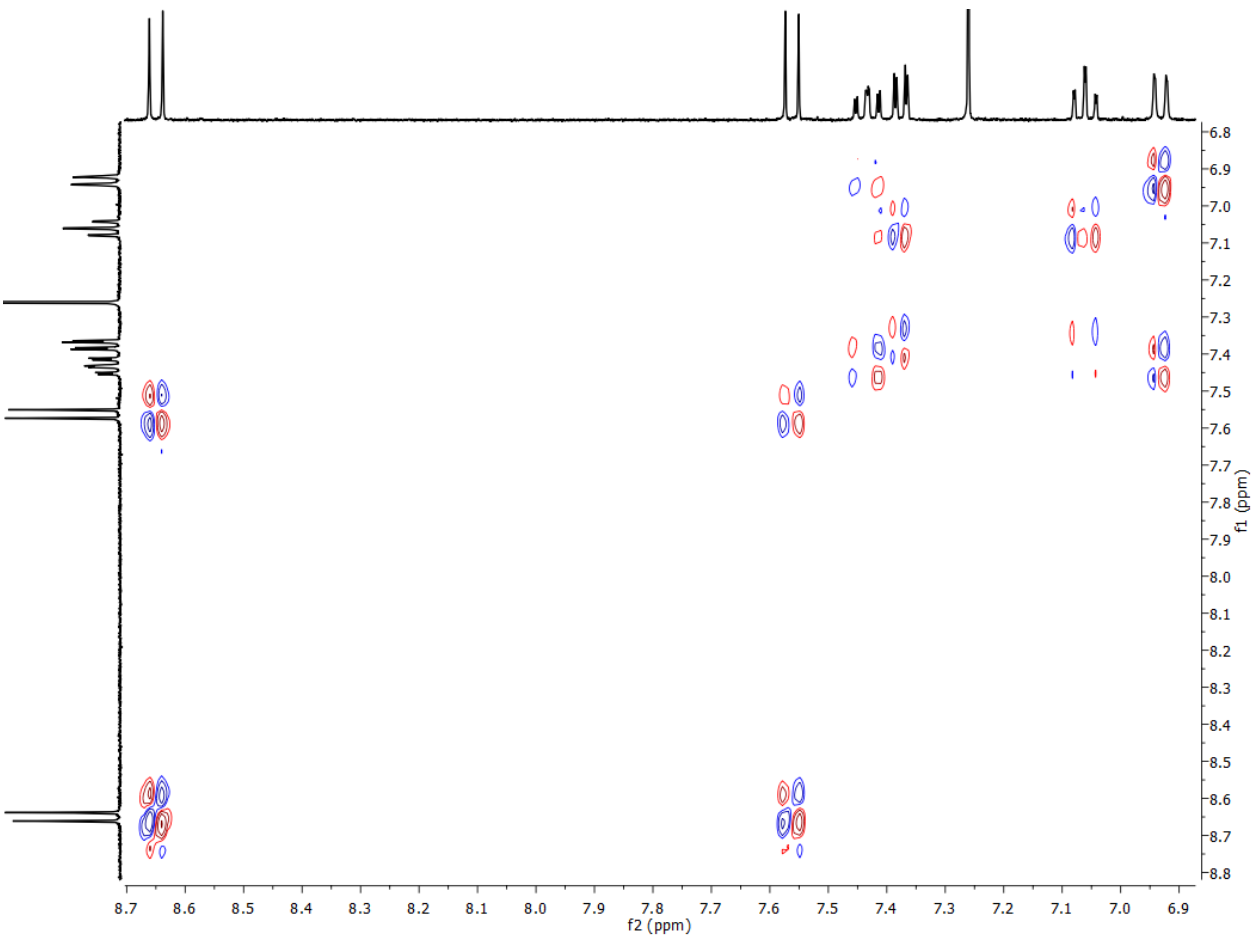

Supplementary Figure 69. COSY NMR (400 MHz) of **8-C8** in $CDCl_3$, measured at 298 K (expansion in aromatic region).

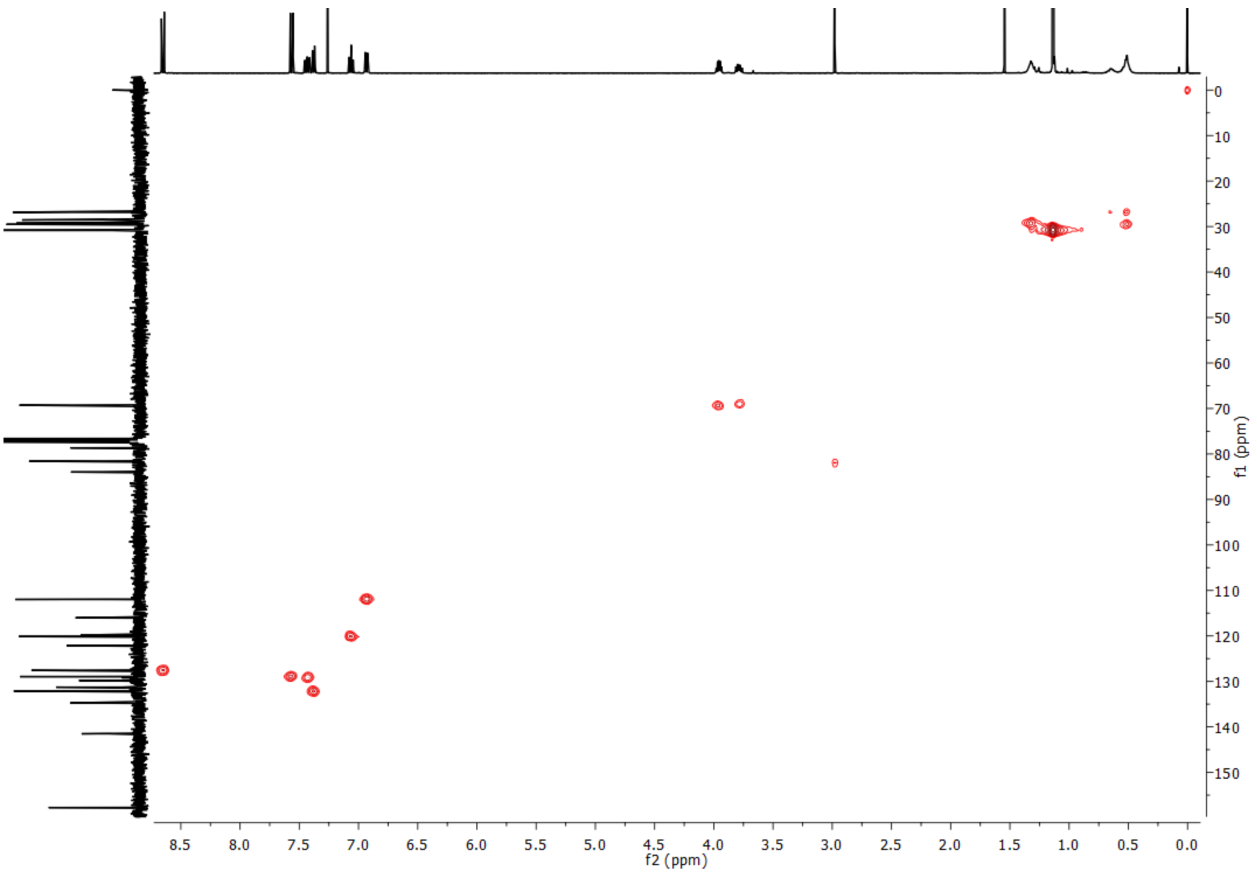

Supplementary Figure 70. HSQC NMR (400 MHz) of **8-C8** in $CDCl_3$, measured at 298 K.

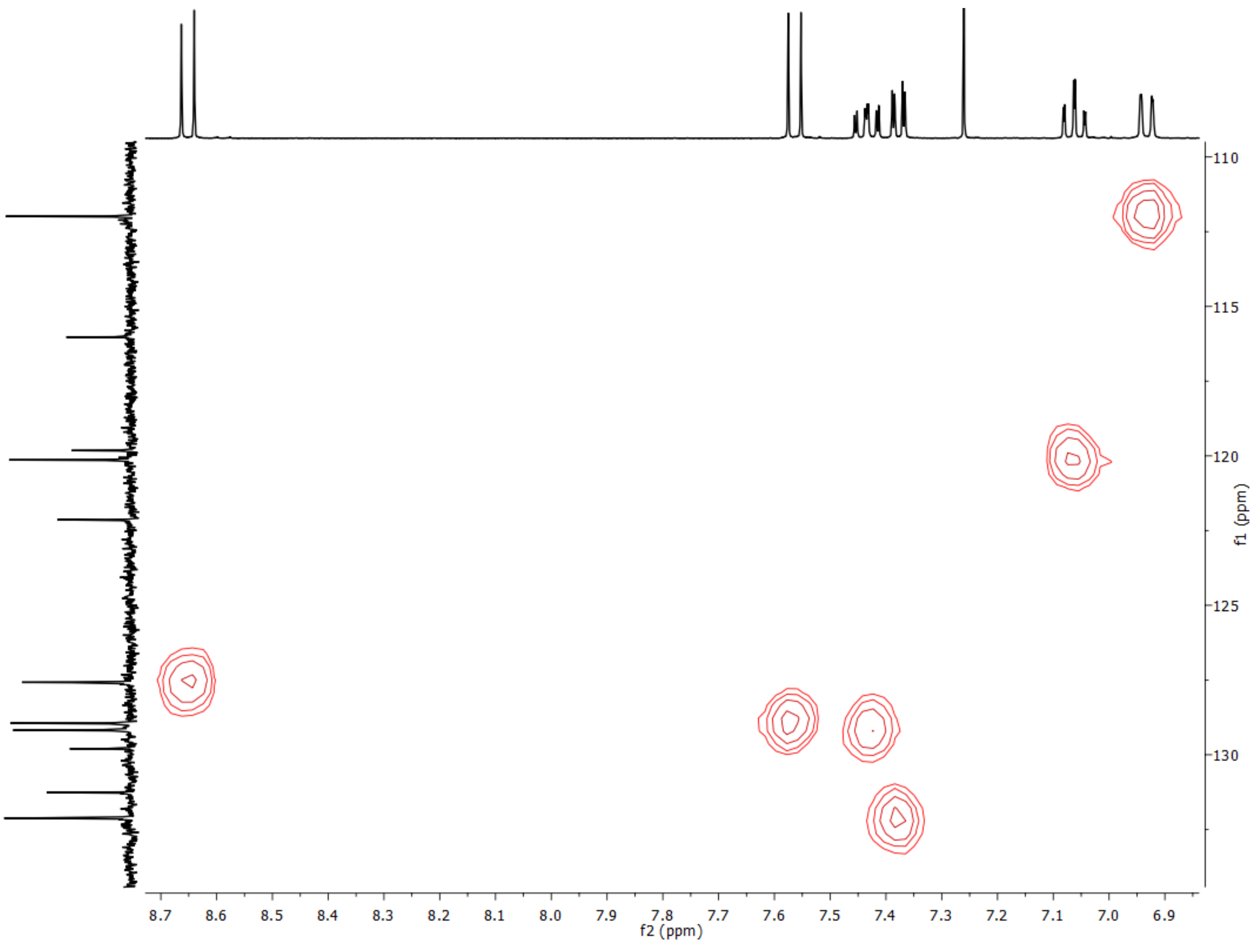

Supplementary Figure 71. HSQC NMR (400 MHz) of **8-C8** in $CDCl_3$, measured at 298 K (expansion in aromatic region).

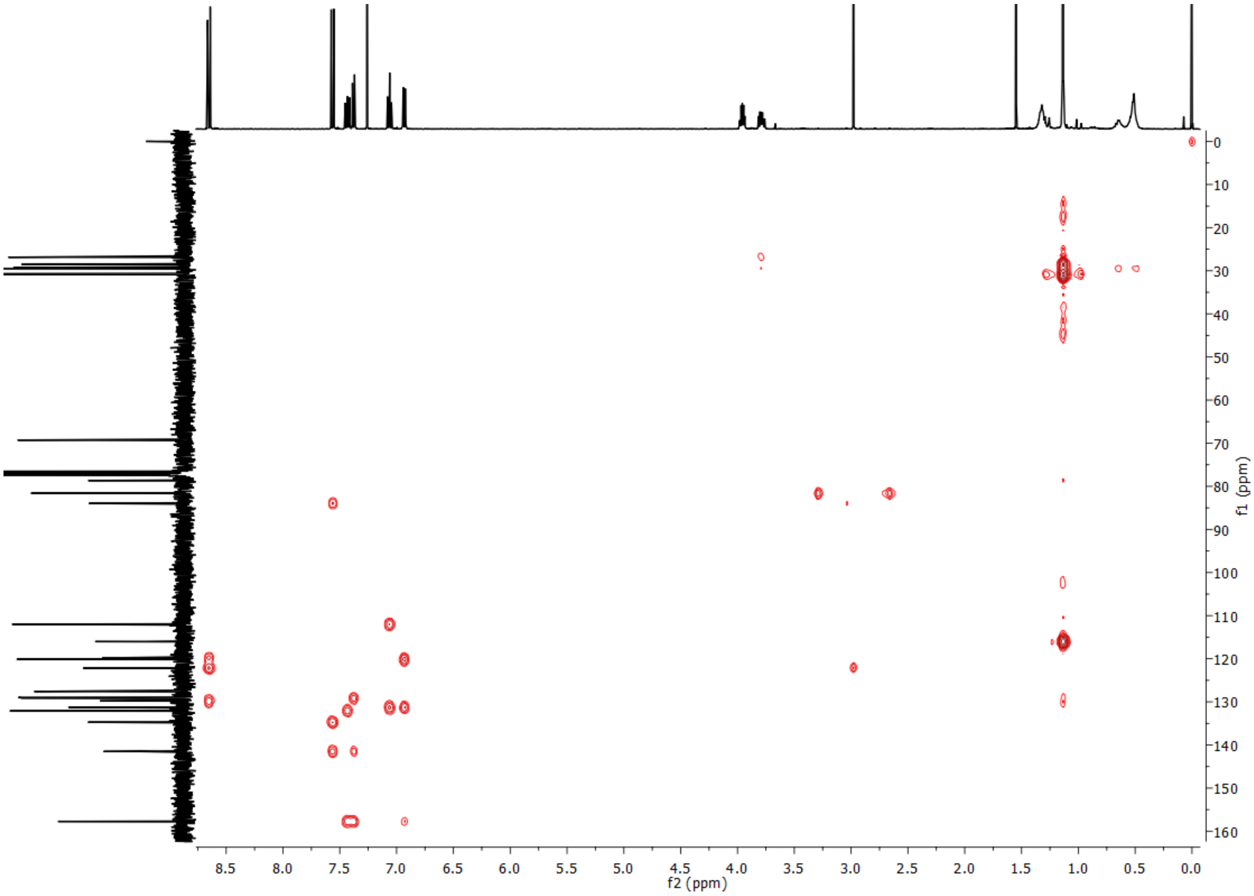

Supplementary Figure 72. HMBC NMR (400 MHz) of **8-C8** in $CDCl_3$, measured at 298 K.

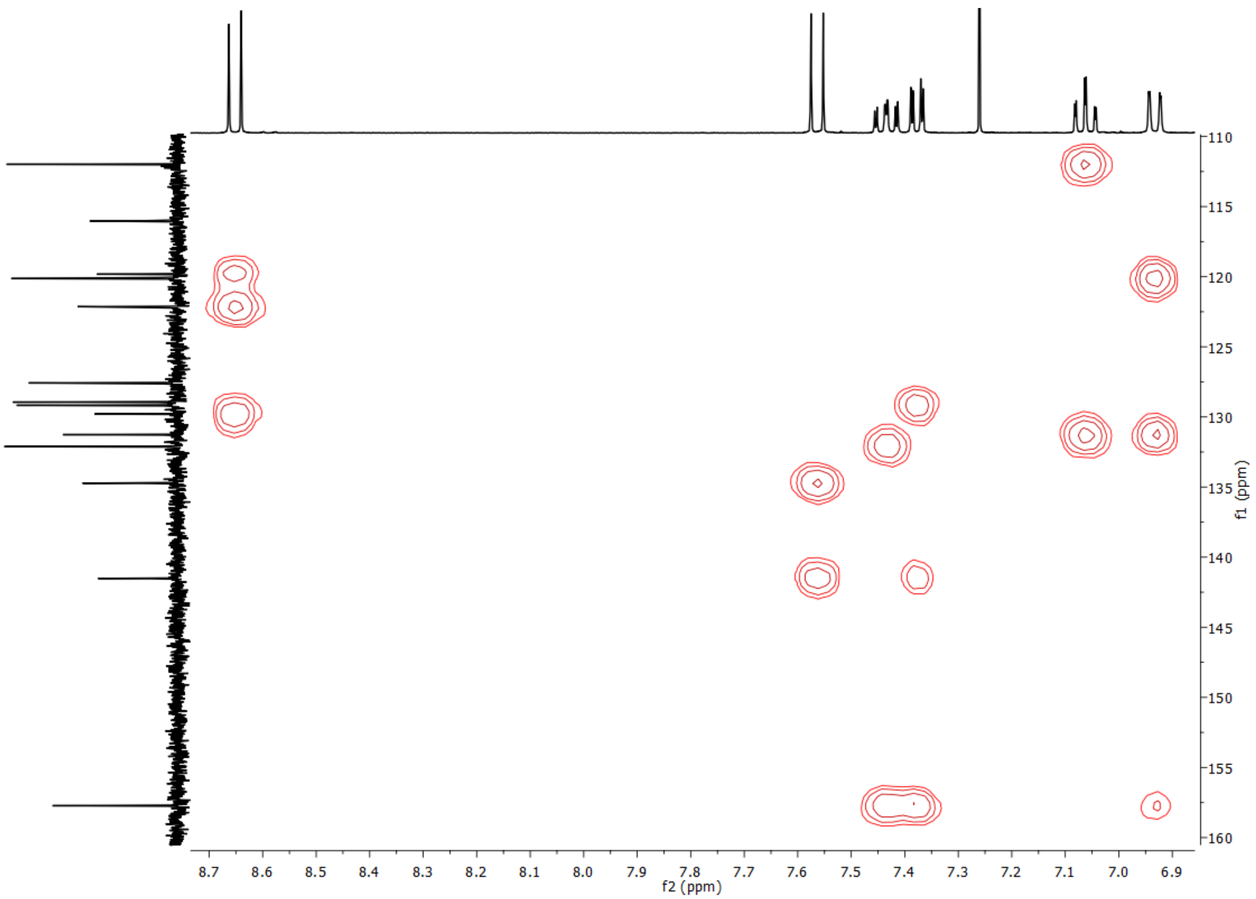

Supplementary Figure 73. HMBC NMR (400 MHz) of **8-C8** in $CDCl_3$, measured at 298 K (expansion in aromatic region).

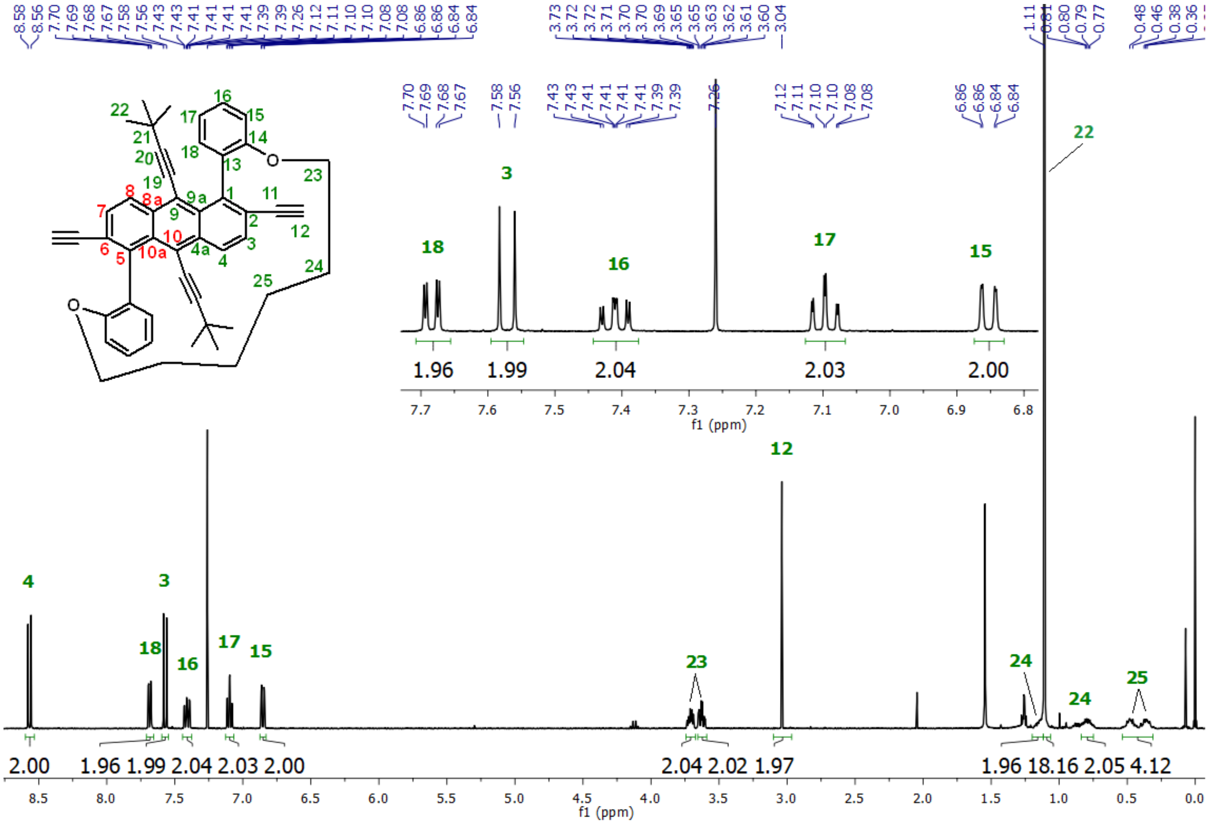

Supplementary Figure 74. ^{1}H NMR (400 MHz) of **8-C6** in $CDCl_3$, measured at 298 K.

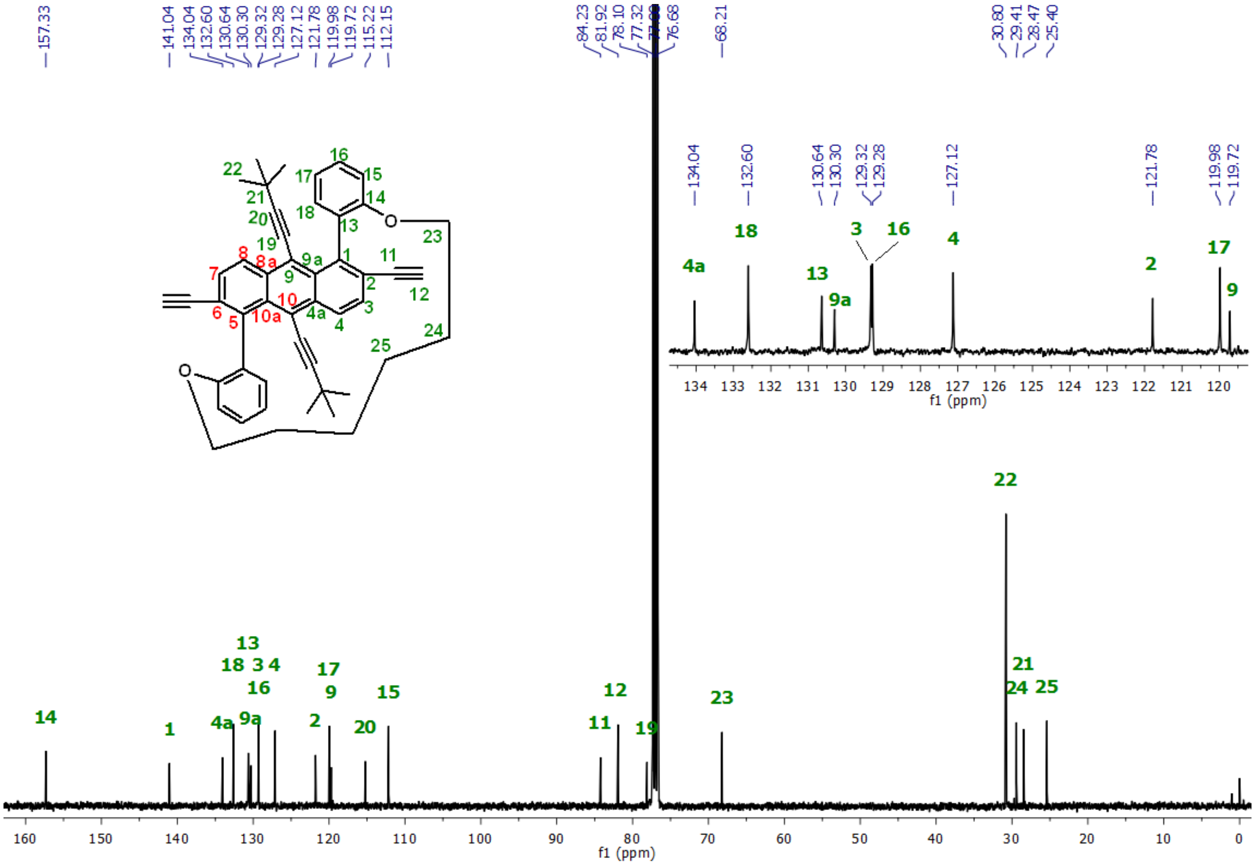

Supplementary Figure 75. ^{13}C NMR (101 MHz) of **8-C6** in $CDCl_3$, measured at 298 K.

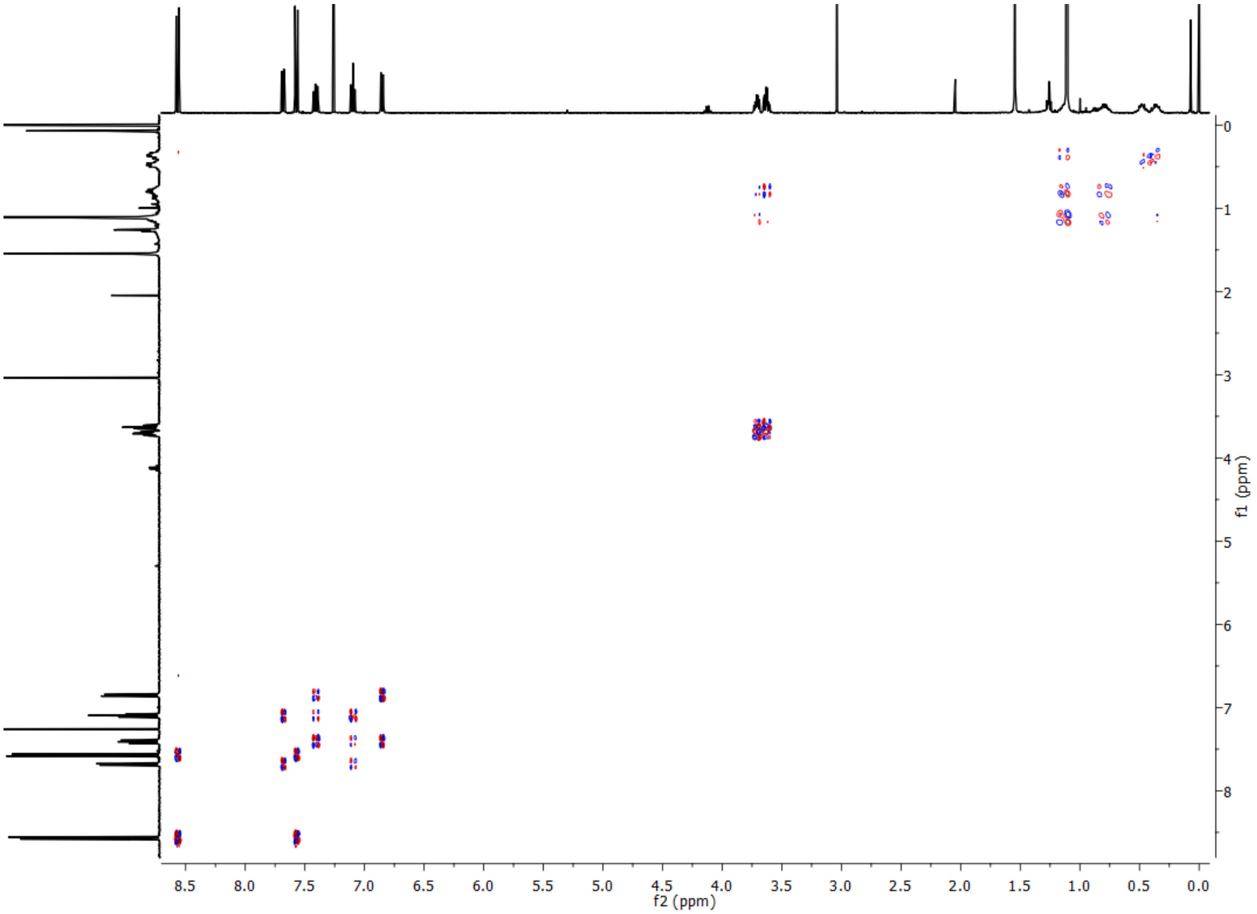

Supplementary Figure 76. COSY NMR (400 MHz) of **8-C6** in $CDCl_3$, measured at 298 K.

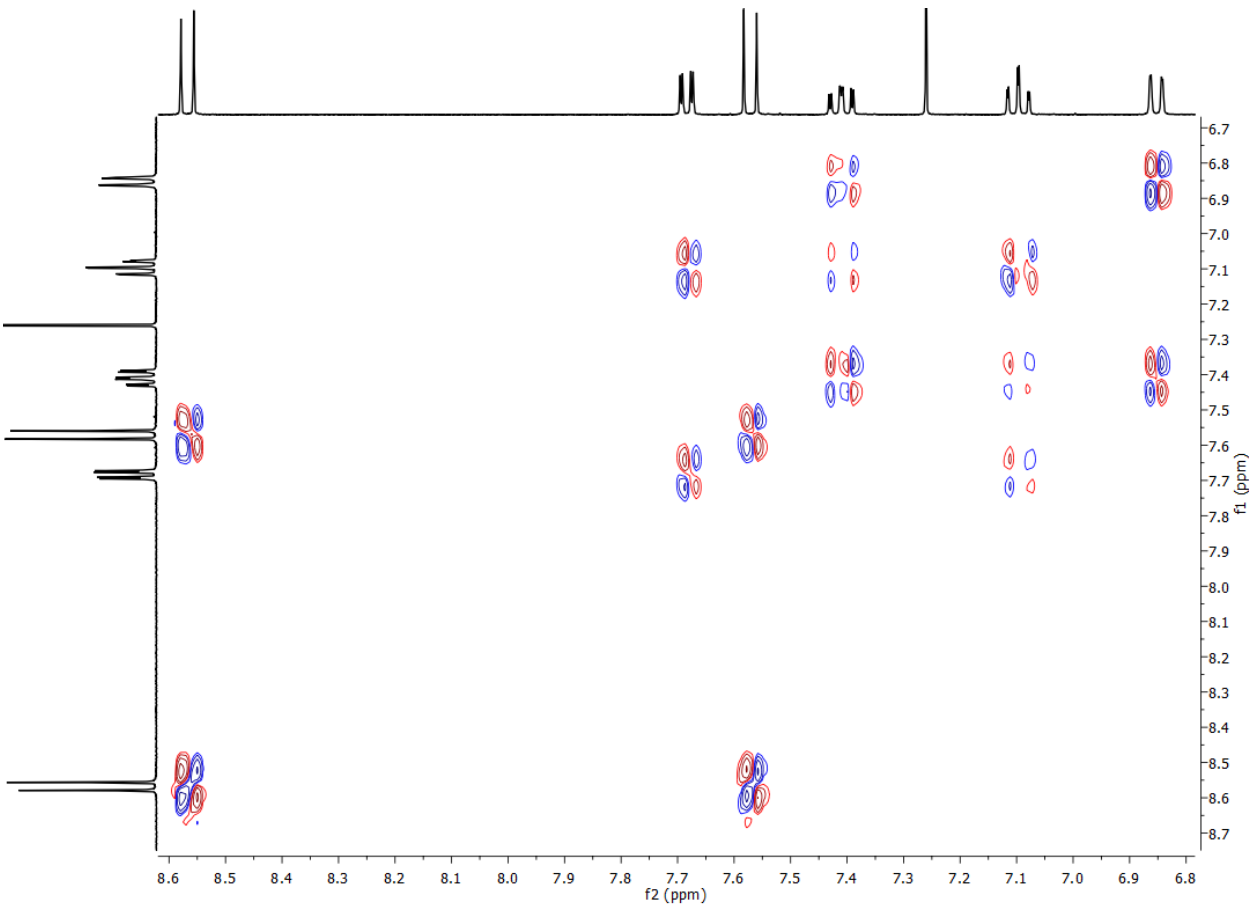

Supplementary Figure 77. COSY NMR (400 MHz) of **8-C6** in $CDCl_3$, measured at 298 K (expansion in aromatic region).

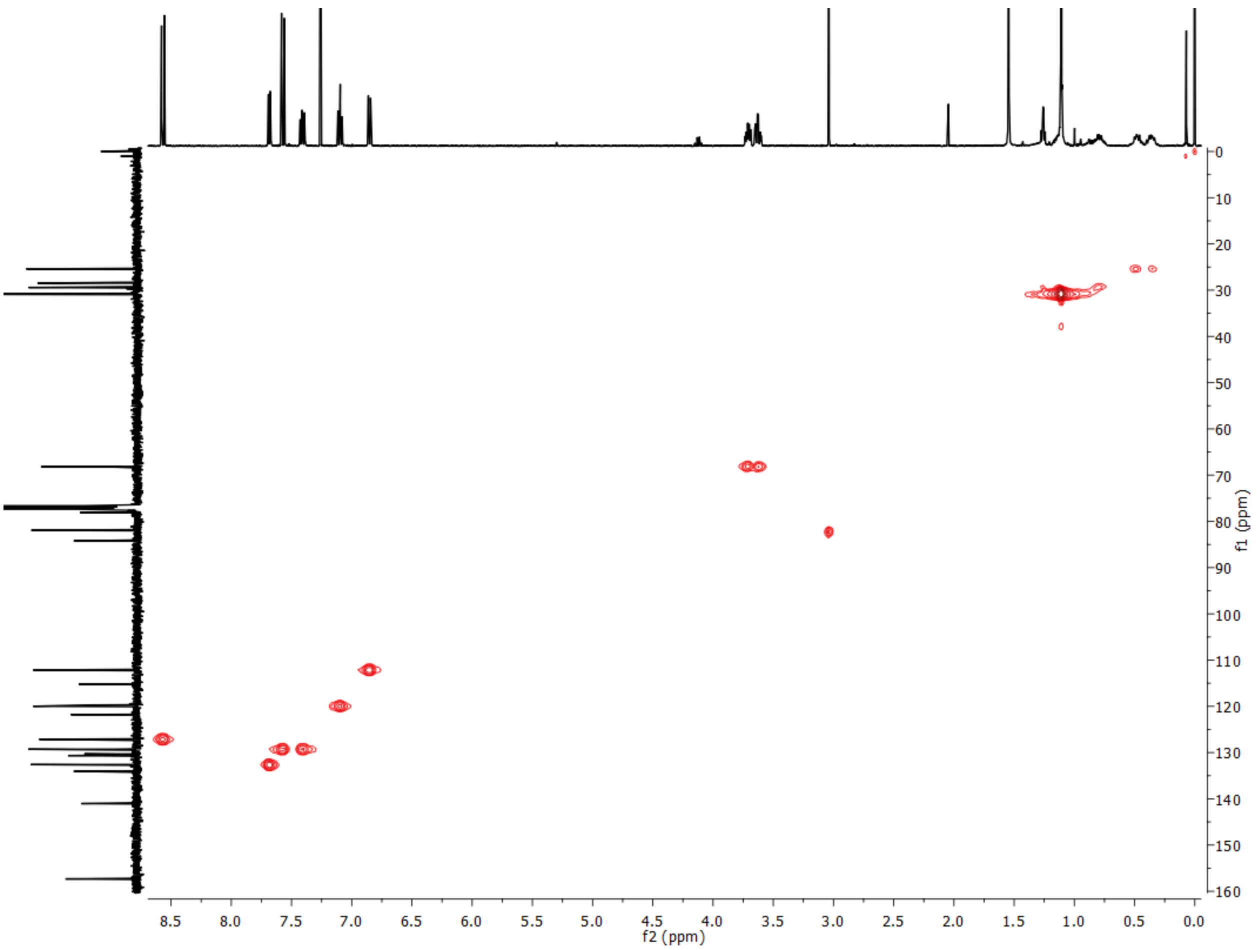

Supplementary Figure 78. HSQC NMR (400 MHz) of **8-C6** in $CDCl_3$, measured at 298 K.

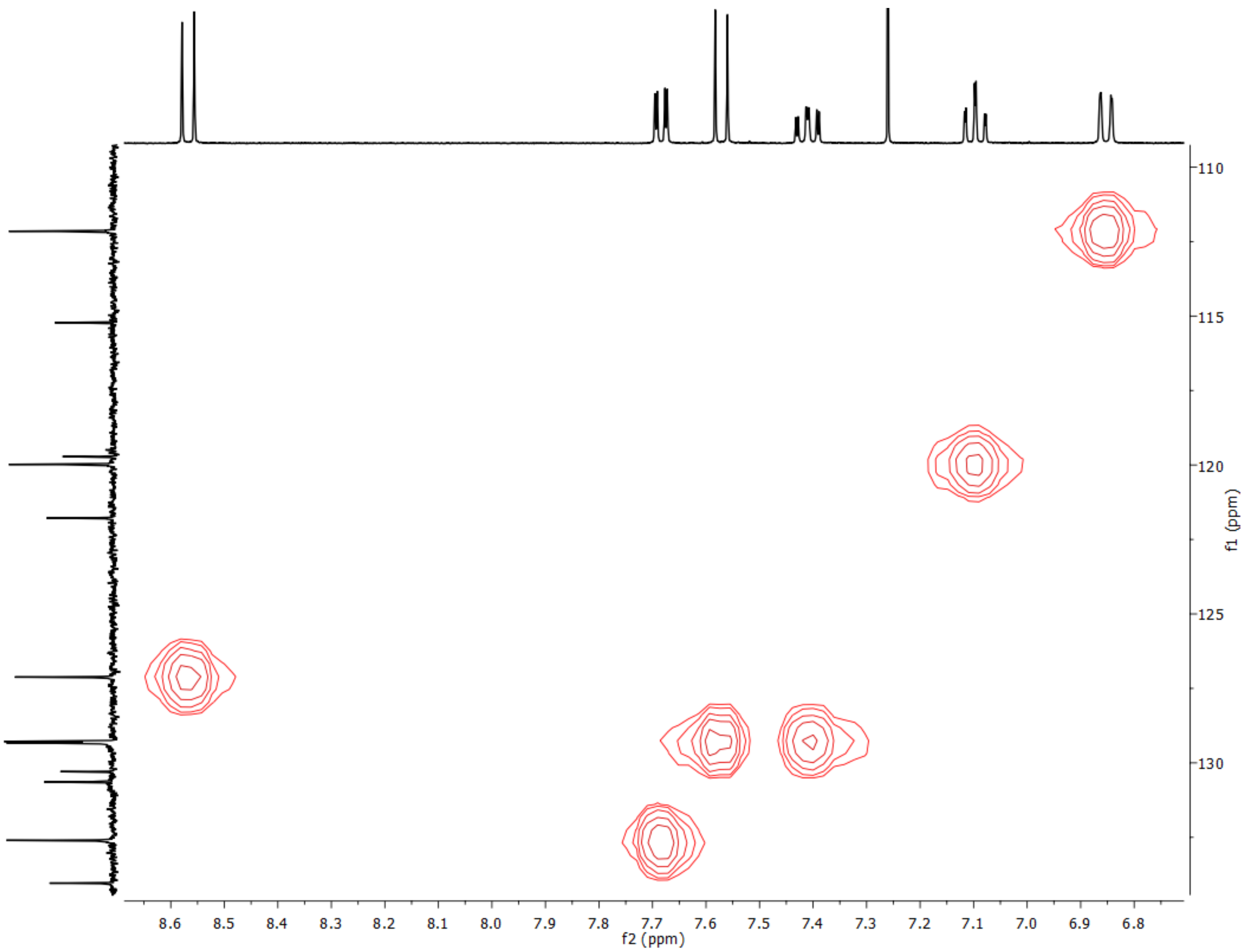

Supplementary Figure 79. HSQC NMR (400 MHz) of **8-C6** in $CDCl_3$, measured at 298 K (expansion in aromatic region).

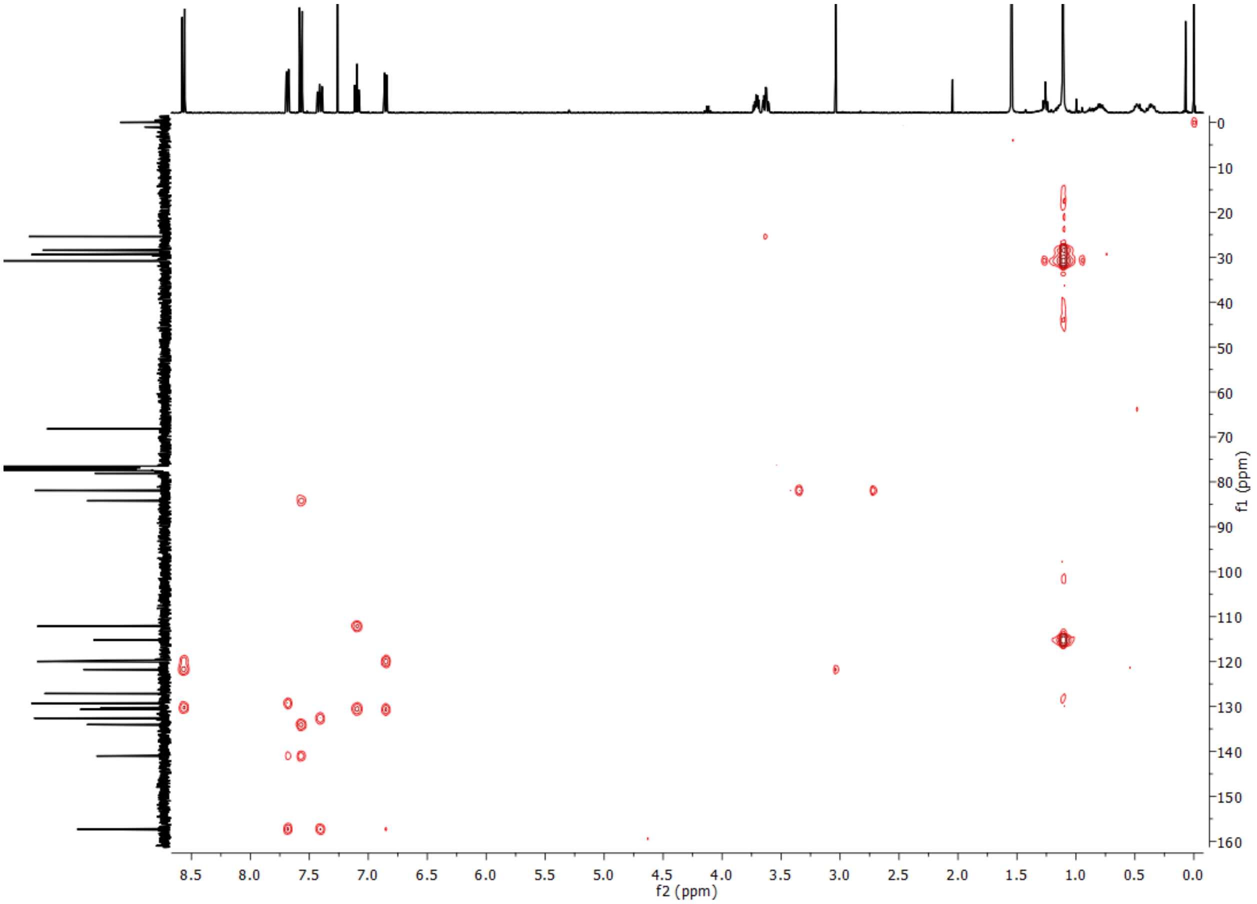

Supplementary Figure 80. HMBC NMR (400 MHz) of **8-C6** in $CDCl_3$, measured at 298 K.

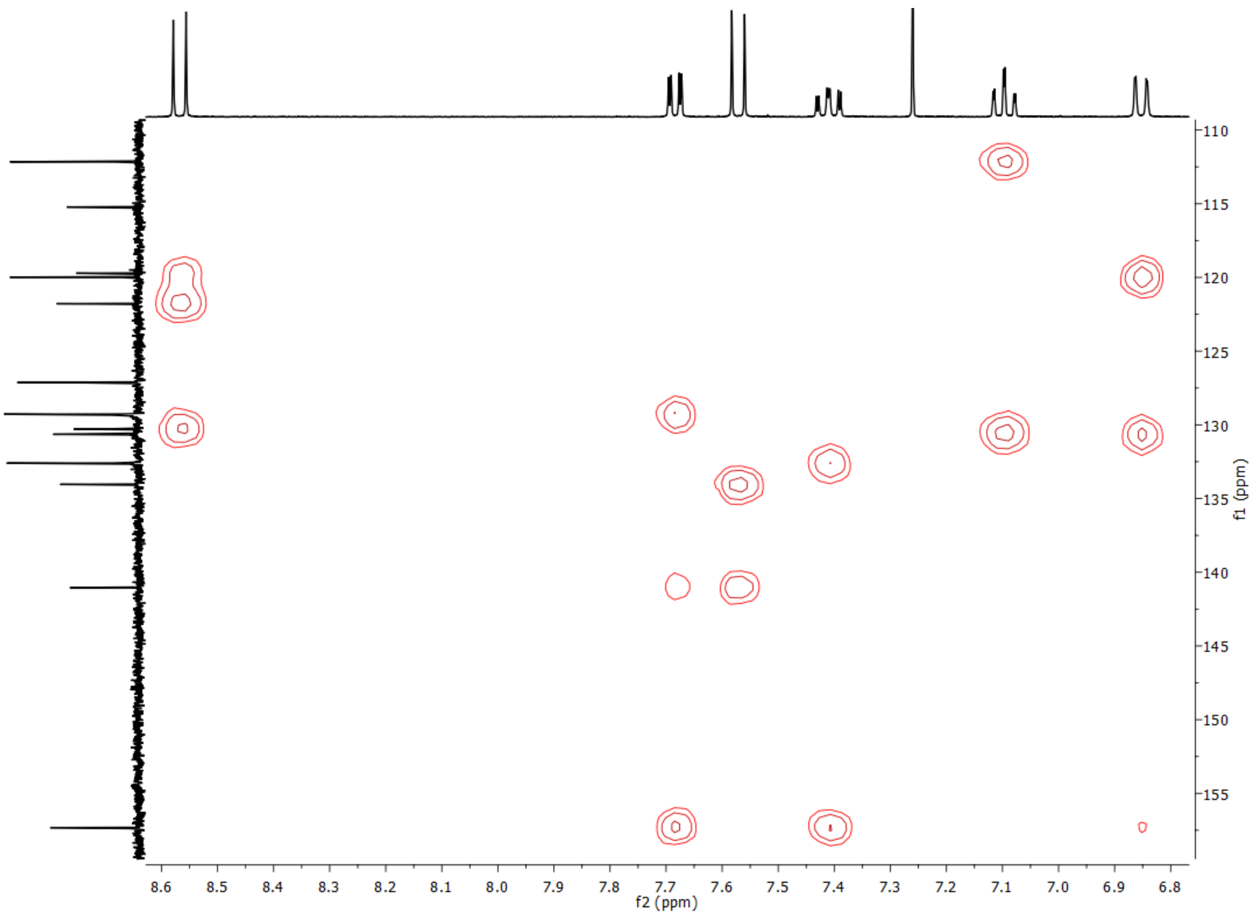

Supplementary Figure 81. HMBC NMR (400 MHz) of **8-C6** in $CDCl_3$, measured at 298 K (expansion in aromatic region).

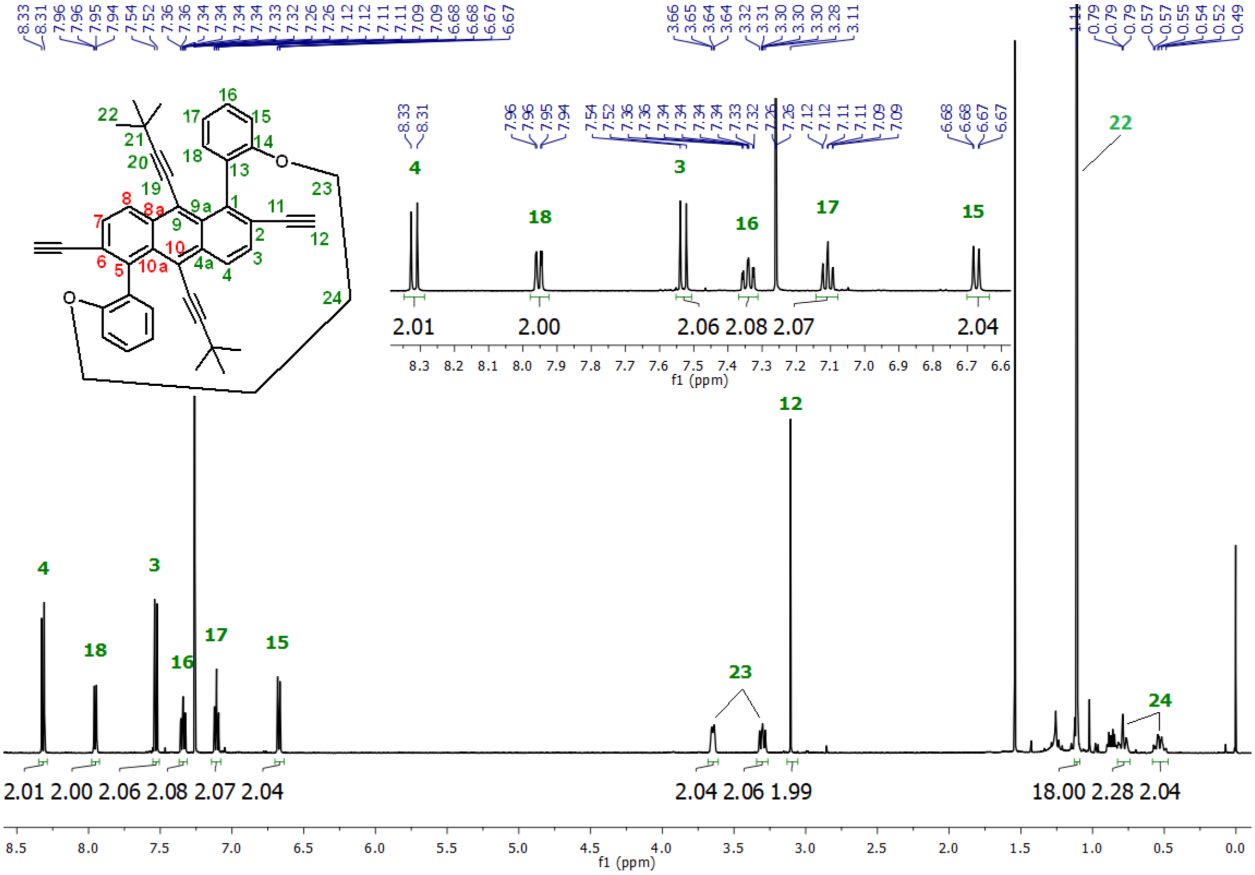

Supplementary Figure 82. ^{1}H NMR (500 MHz) of **8-C4** in $CDCl_3$, measured at 298 K.

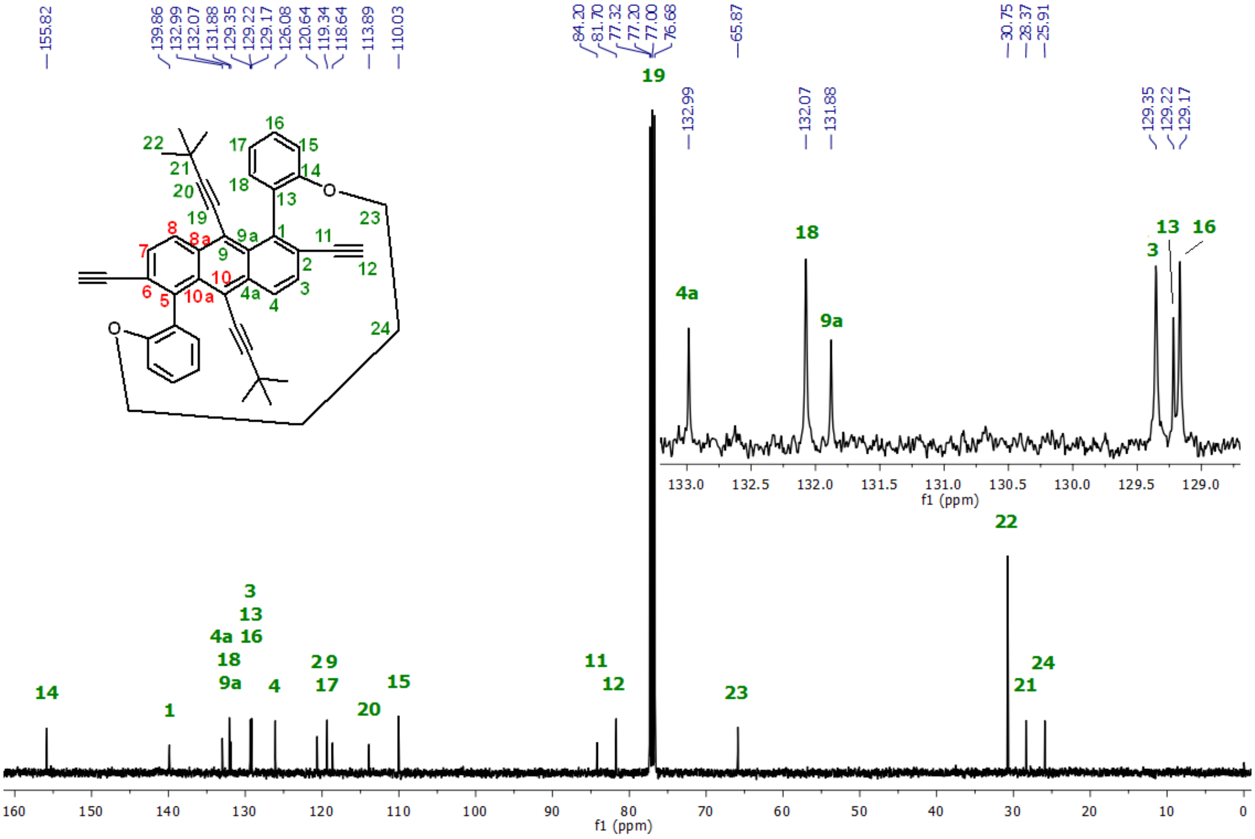

Supplementary Figure 83. ^{13}C NMR (126 MHz) of **8-C4** in $CDCl_3$, measured at 298 K.

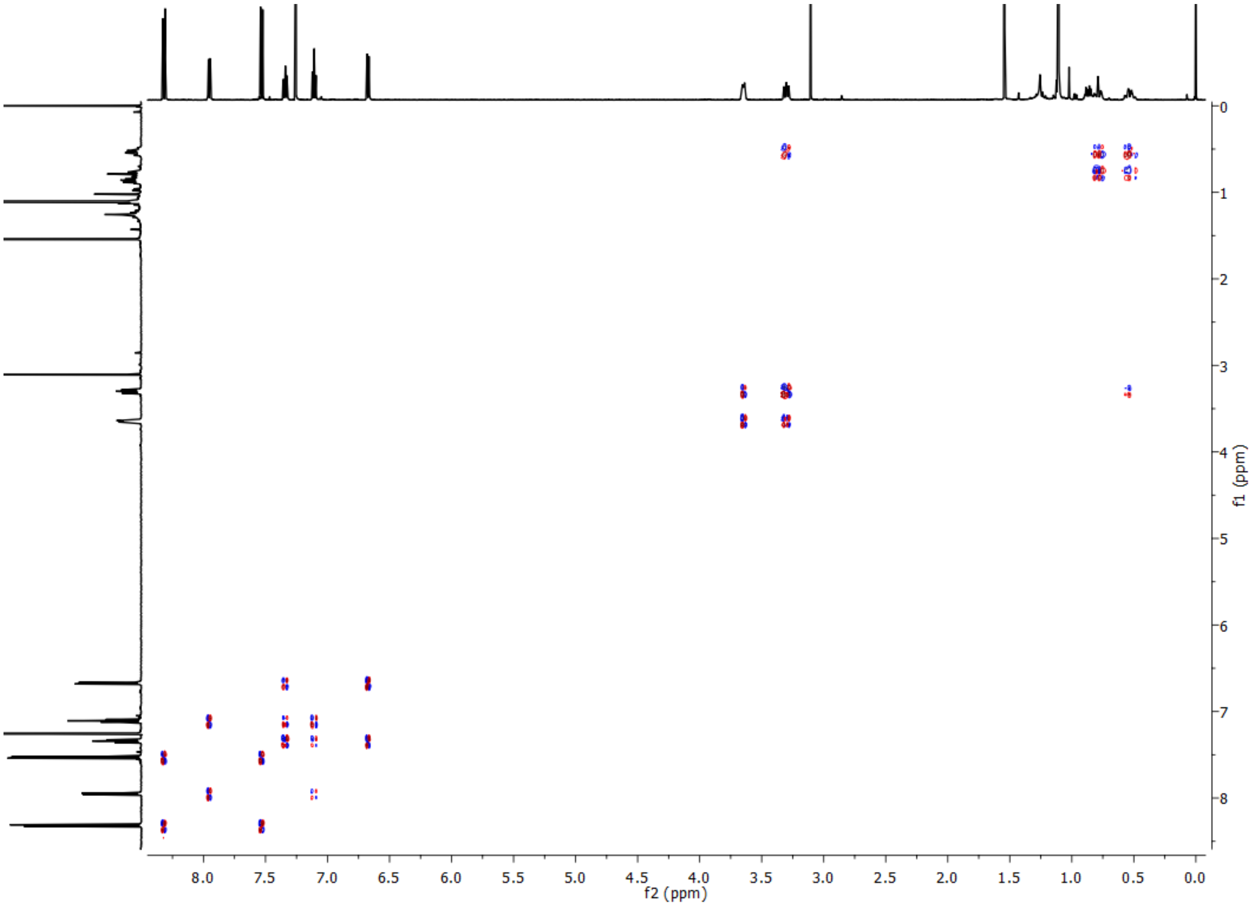

Supplementary Figure 84. COSY NMR (500 MHz) of **8-C4** in $CDCl_3$, measured at 298 K.

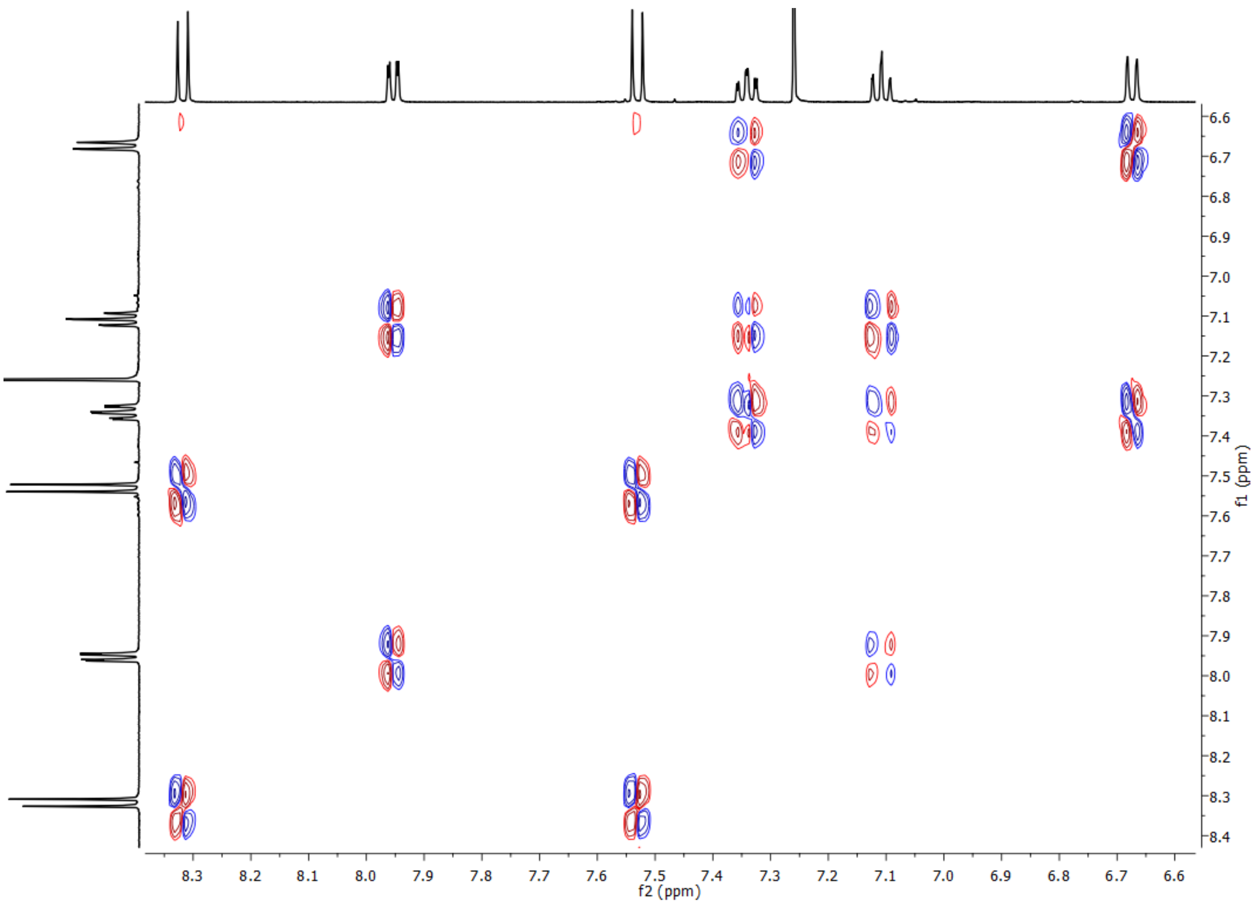

Supplementary Figure 85. COSY NMR (500 MHz) of **8-C4** in $CDCl_3$, measured at 298 K (expansion in aromatic region).

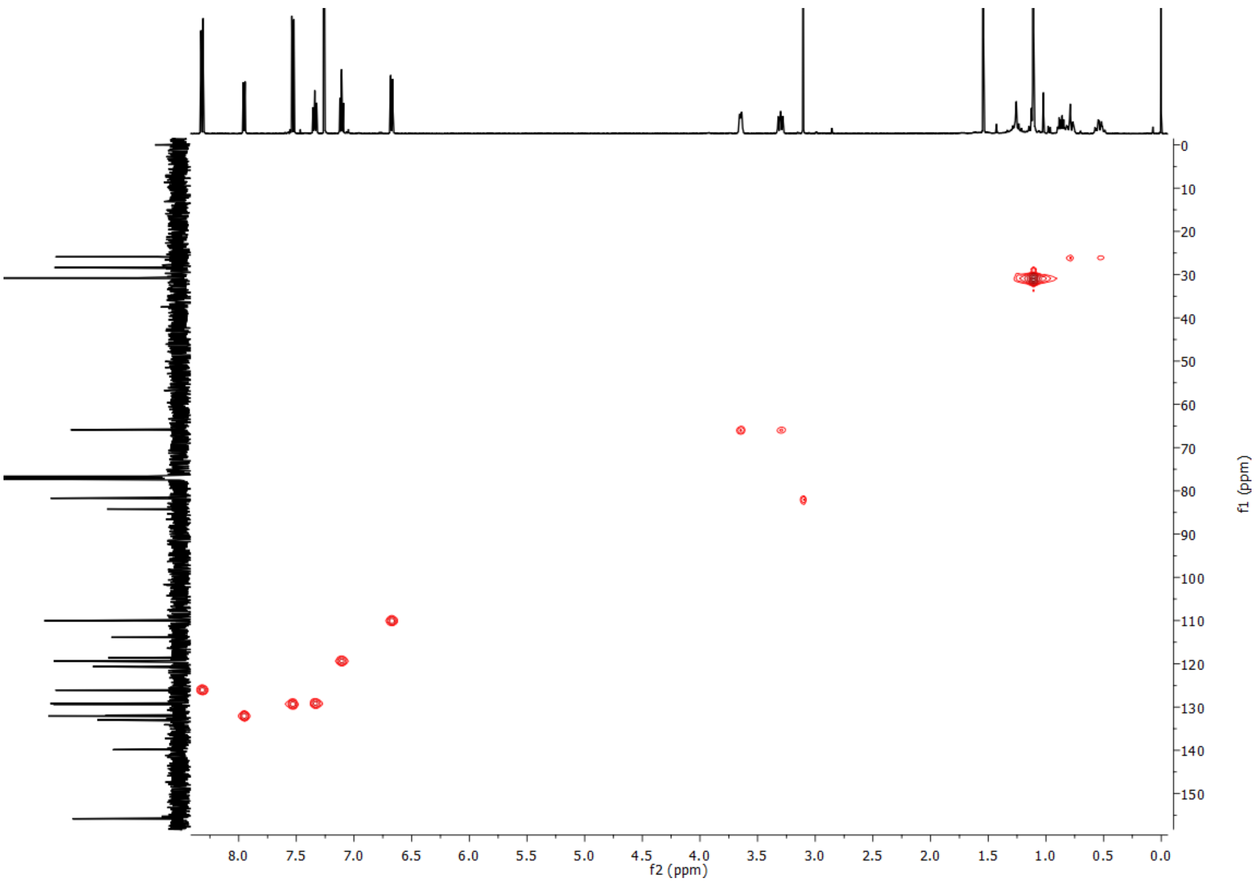

Supplementary Figure 86. HSQC NMR (500 MHz) of **8-C4** in $CDCl_3$, measured at 298 K.

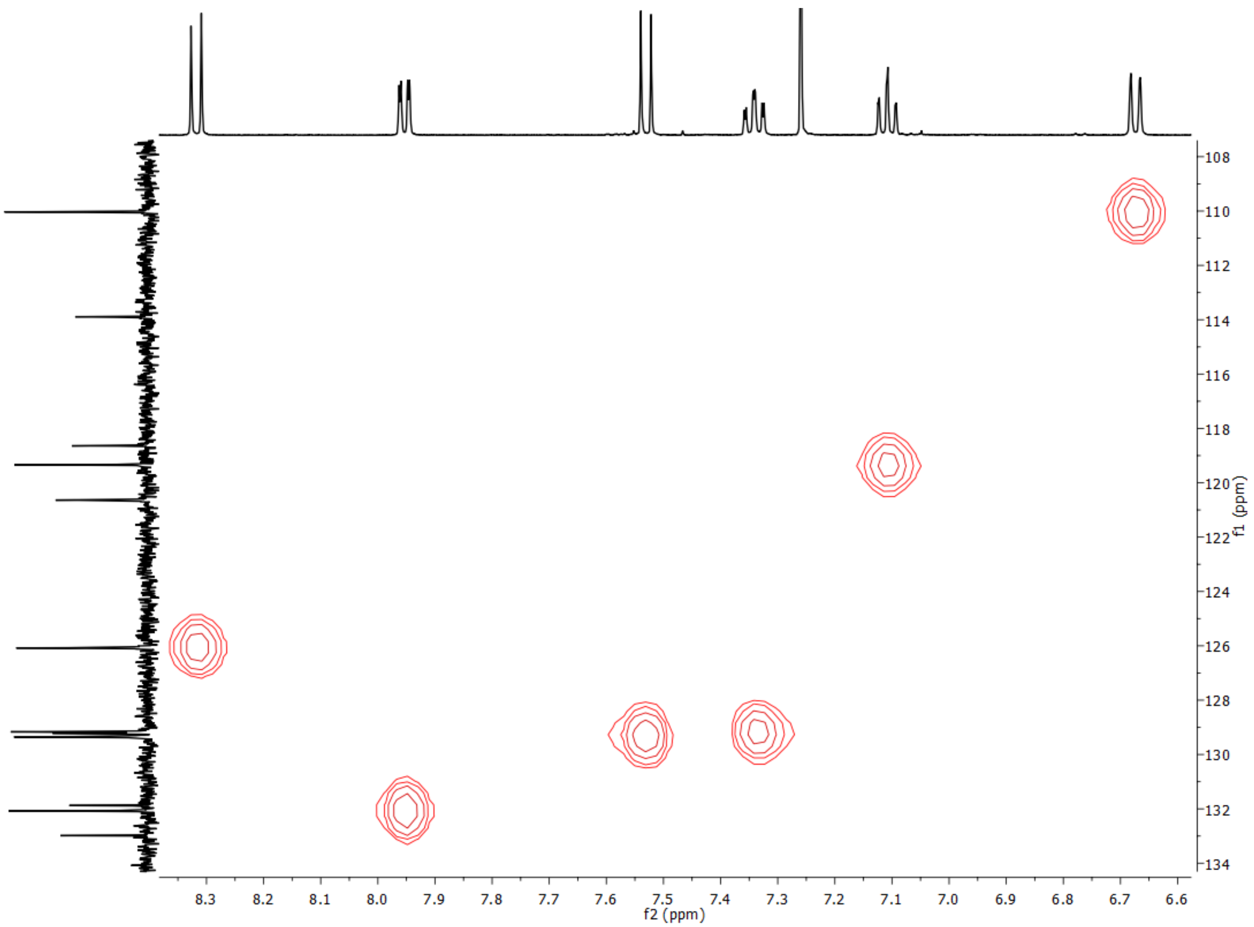

Supplementary Figure 87. HSQC NMR (500 MHz) of **8-C4** in $CDCl_3$, measured at 298 K (expansion in aromatic region).

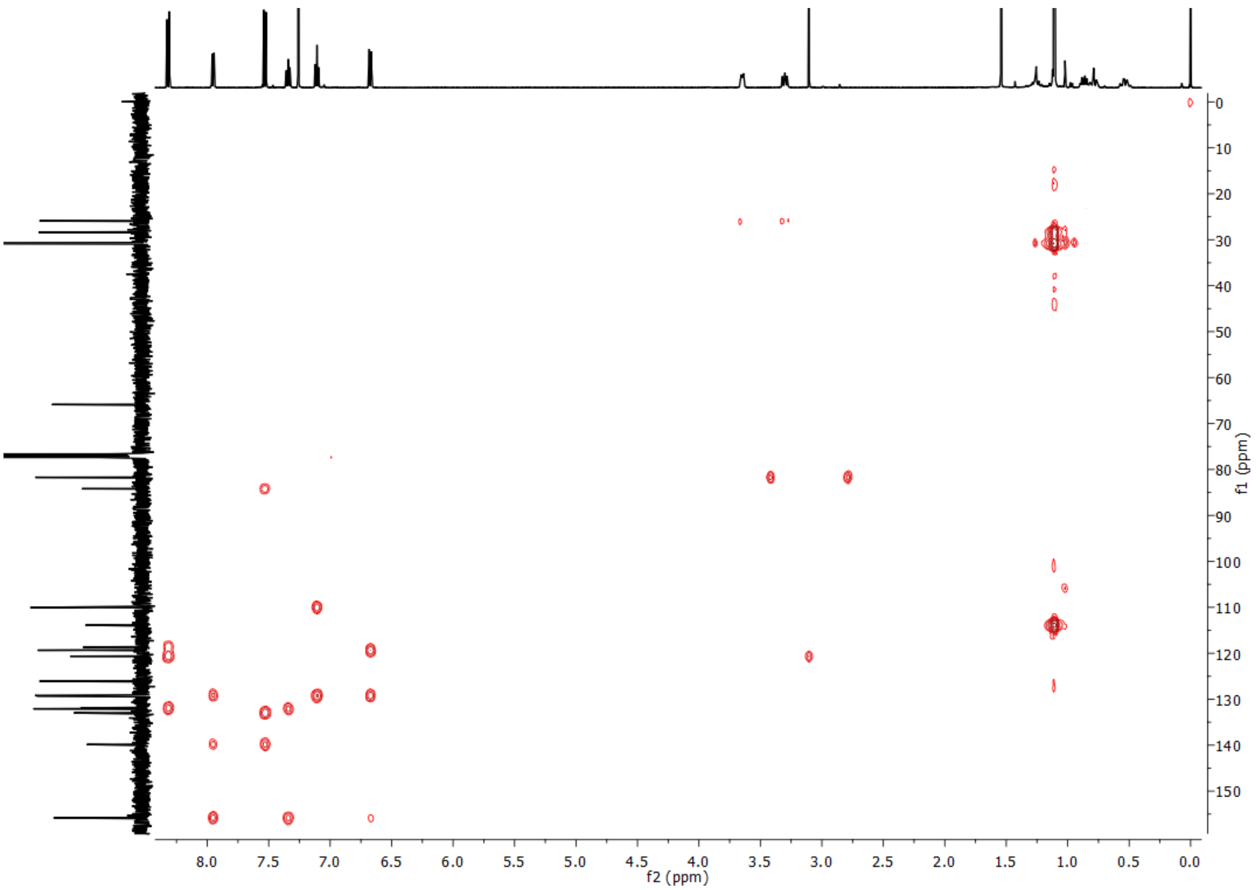

Supplementary Figure 88. HMBC NMR (500 MHz) of **8-C4** in $CDCl_3$, measured at 298 K.

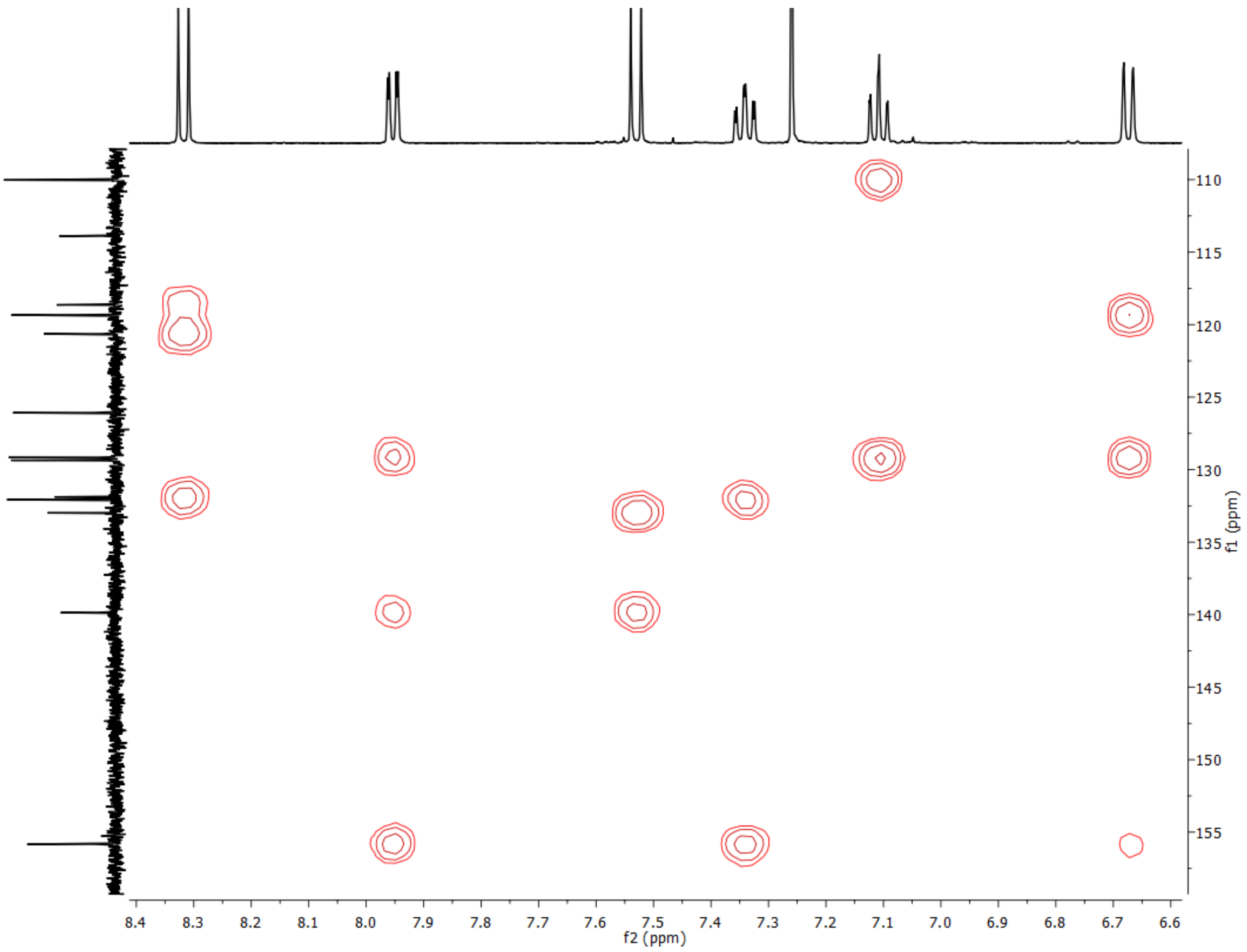

Supplementary Figure 89. HMBC NMR (500 MHz) of **8-C4** in $CDCl_3$, measured at 298 K (expansion in aromatic region).

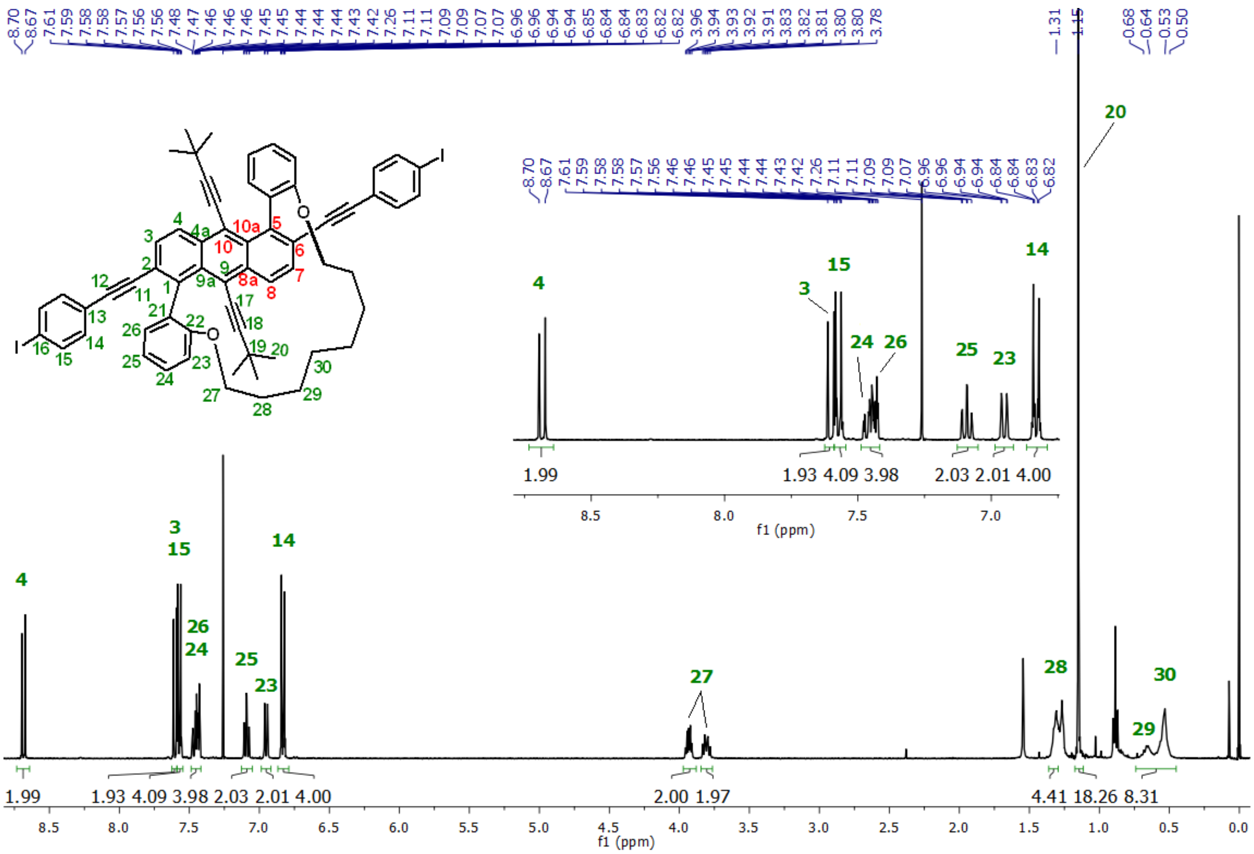

Supplementary Figure 90. ^{1}H NMR (400 MHz) of *P*-**9-C8** in $CDCl_3$, measured at 298 K.

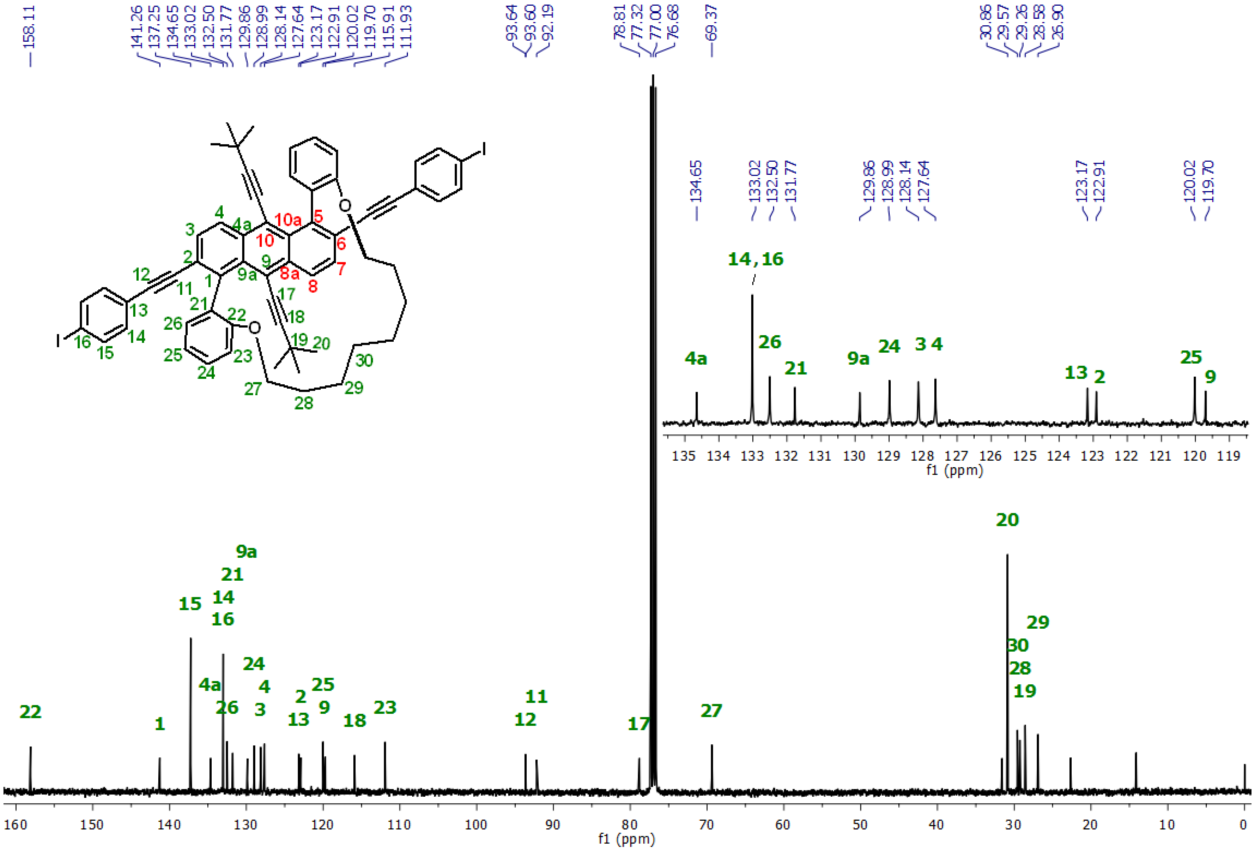

Supplementary Figure 91. ^{13}C NMR (101 MHz) of *P*-**9-C8** in $CDCl_3$, measured at 298 K.

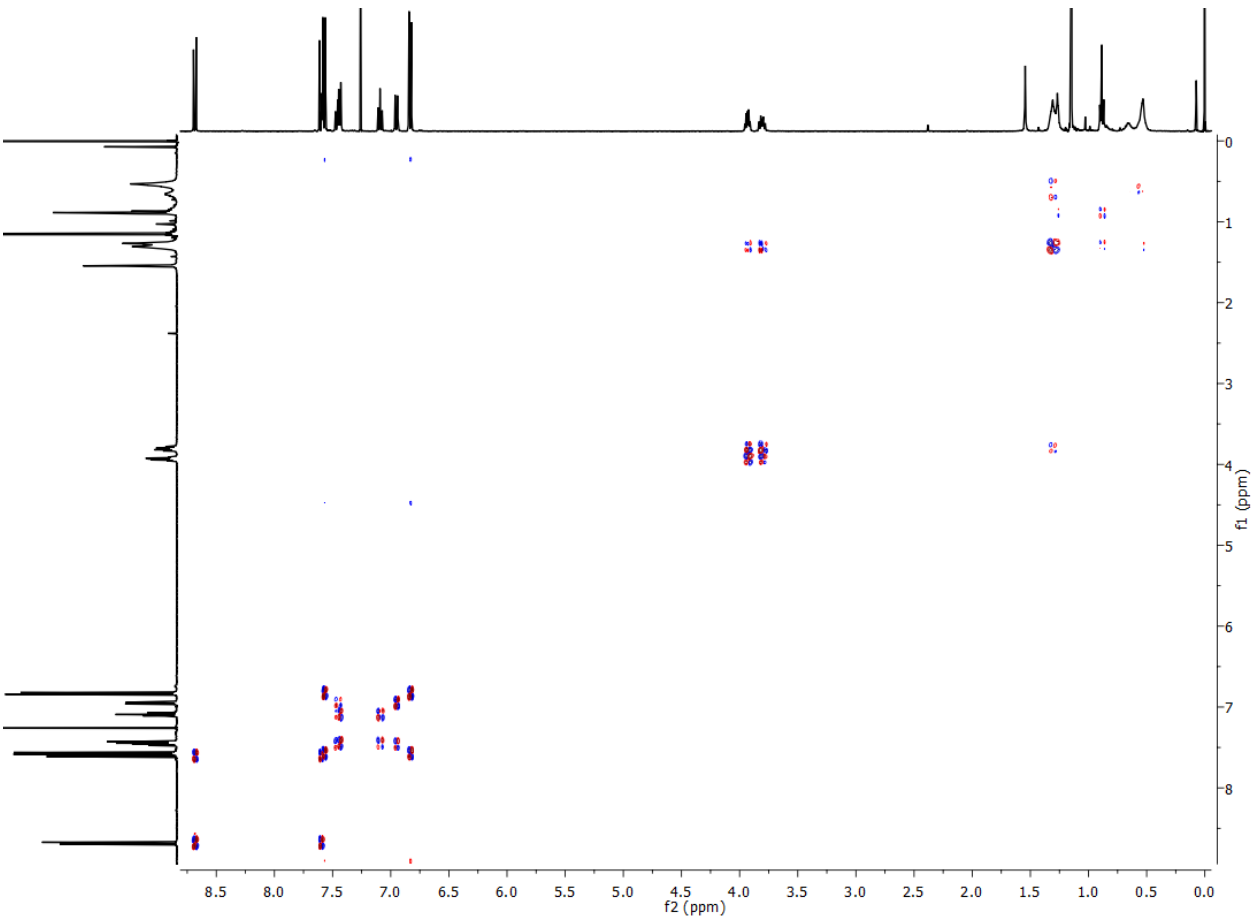

Supplementary Figure 92. COSY NMR (400 MHz) of *P*-**9-C8** in $CDCl_3$, measured at 298 K.

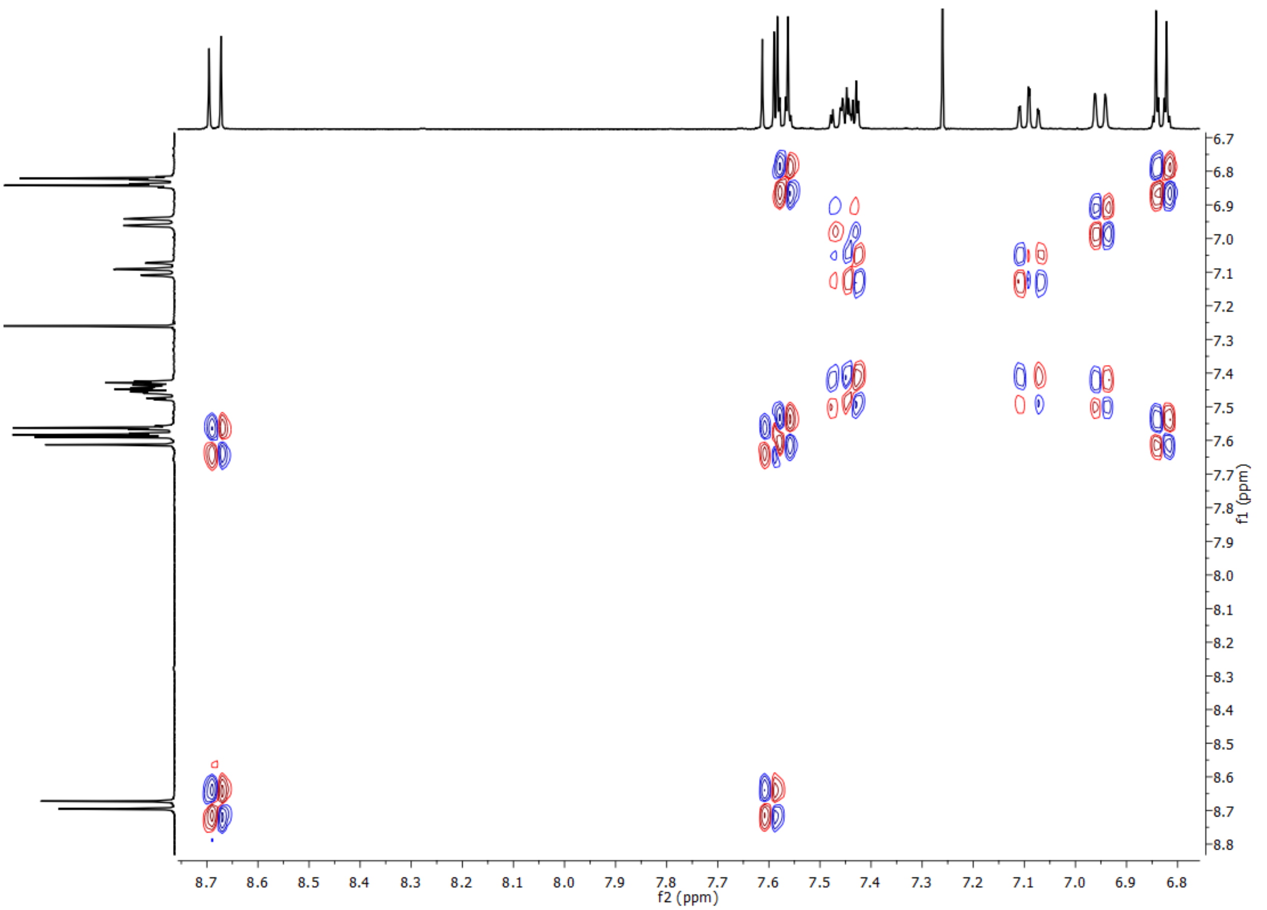

Supplementary Figure 93. COSY NMR (400 MHz) of ***P*-9-C8** in $CDCl_3$, measured at 298 K (expansion in aromatic region).

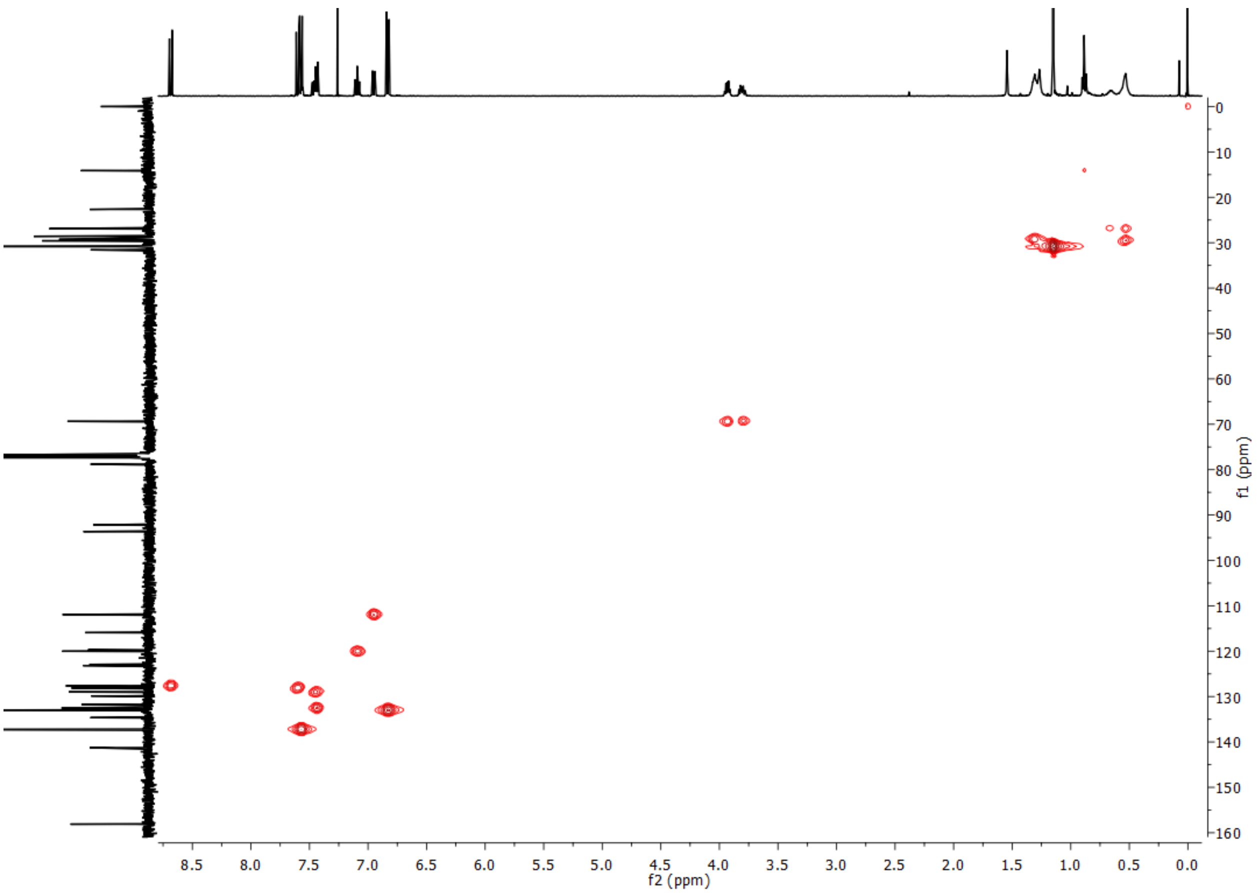

Supplementary Figure 94. HSQC NMR (400 MHz) of *P*-**9-C8** in $CDCl_3$, measured at 298 K.

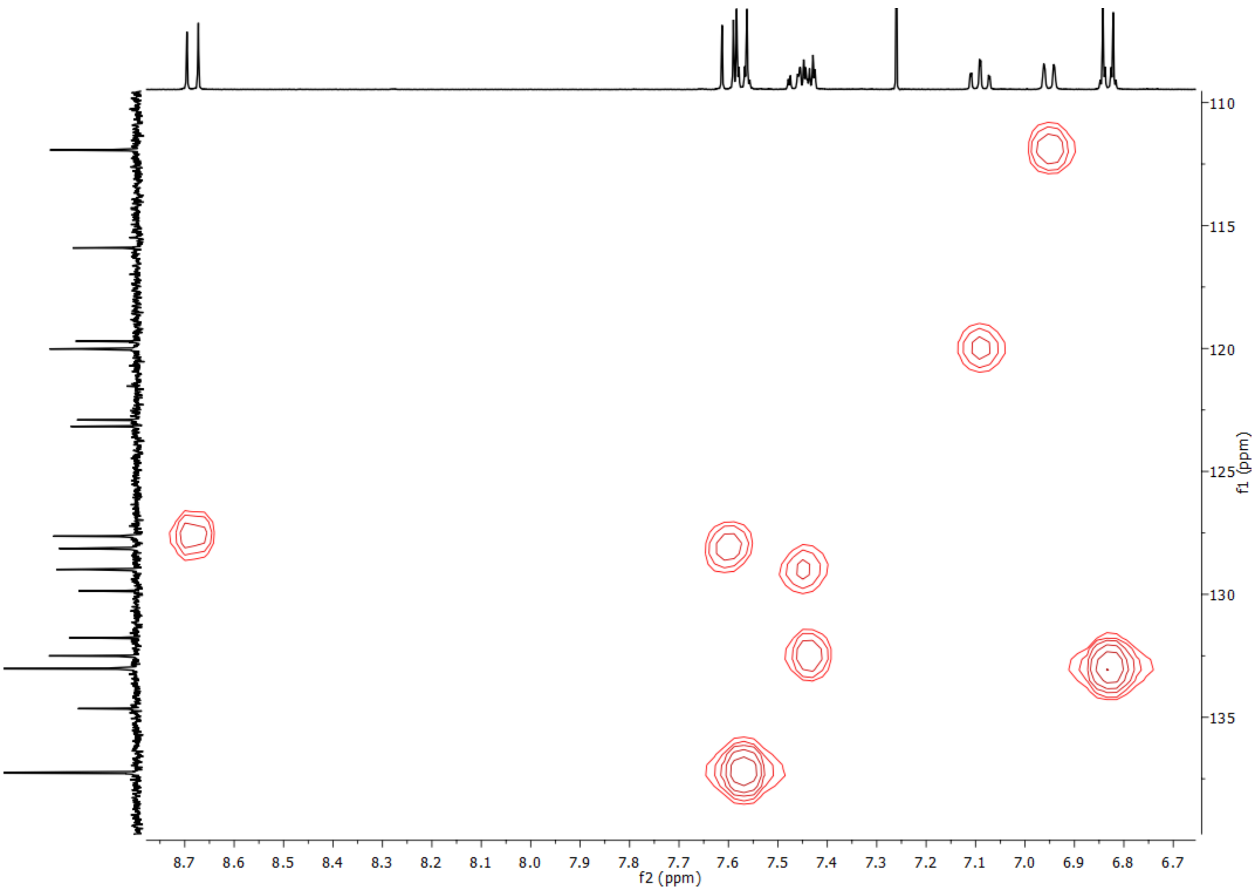

Supplementary Figure 95. HSQC NMR (400 MHz) of *P*-**9-C8** in $CDCl_3$, measured at 298 K (expansion in aromatic region).

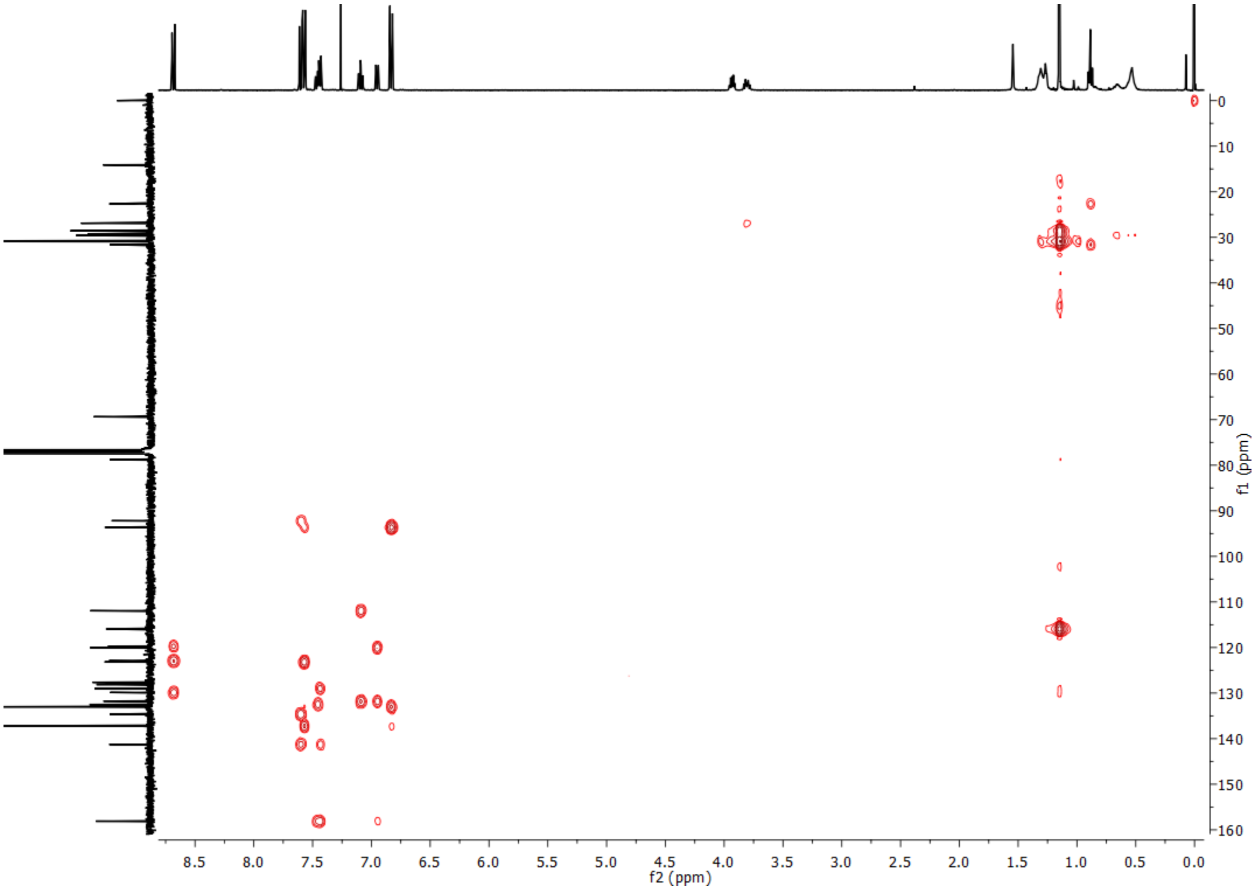

Supplementary Figure 96. HMBC NMR (400 MHz) of *P*-**9-C8** in $CDCl_3$, measured at 298 K.

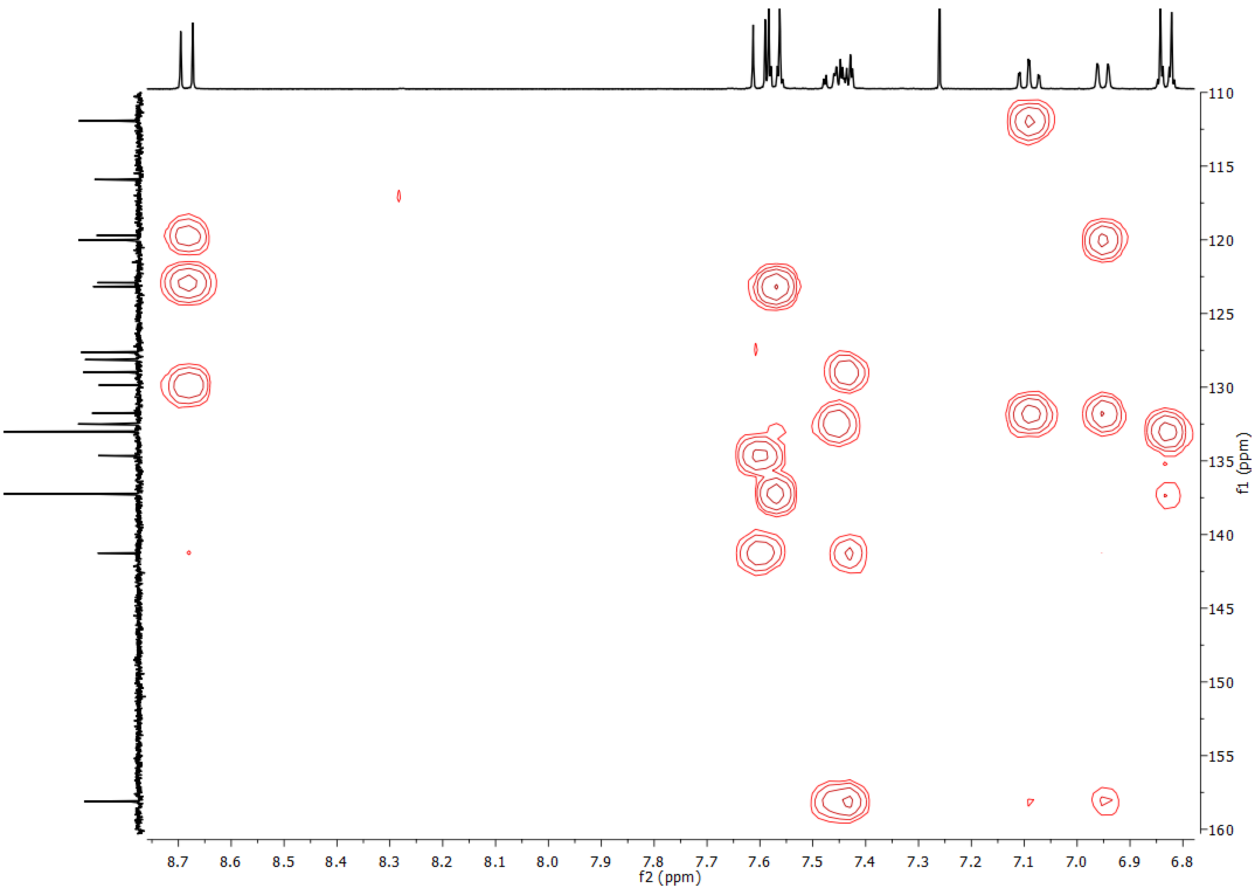

Supplementary Figure 97. HMBC NMR (400 MHz) of ***P*-9-C8** in $CDCl_3$, measured at 298 K (expansion in aromatic region).

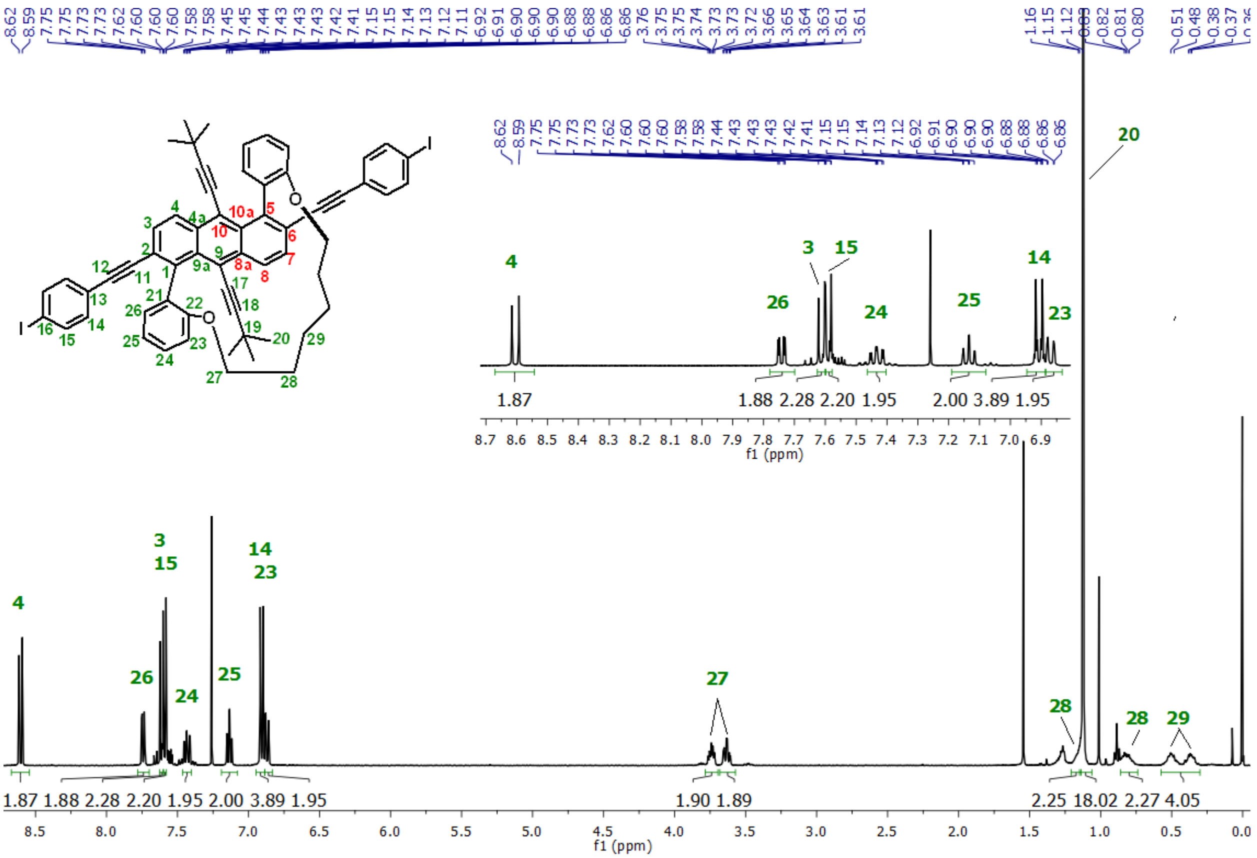

Supplementary Figure 98. ^{1}H NMR (400 MHz) of *P*-**9-C6** in $CDCl_3$, measured at 298 K.

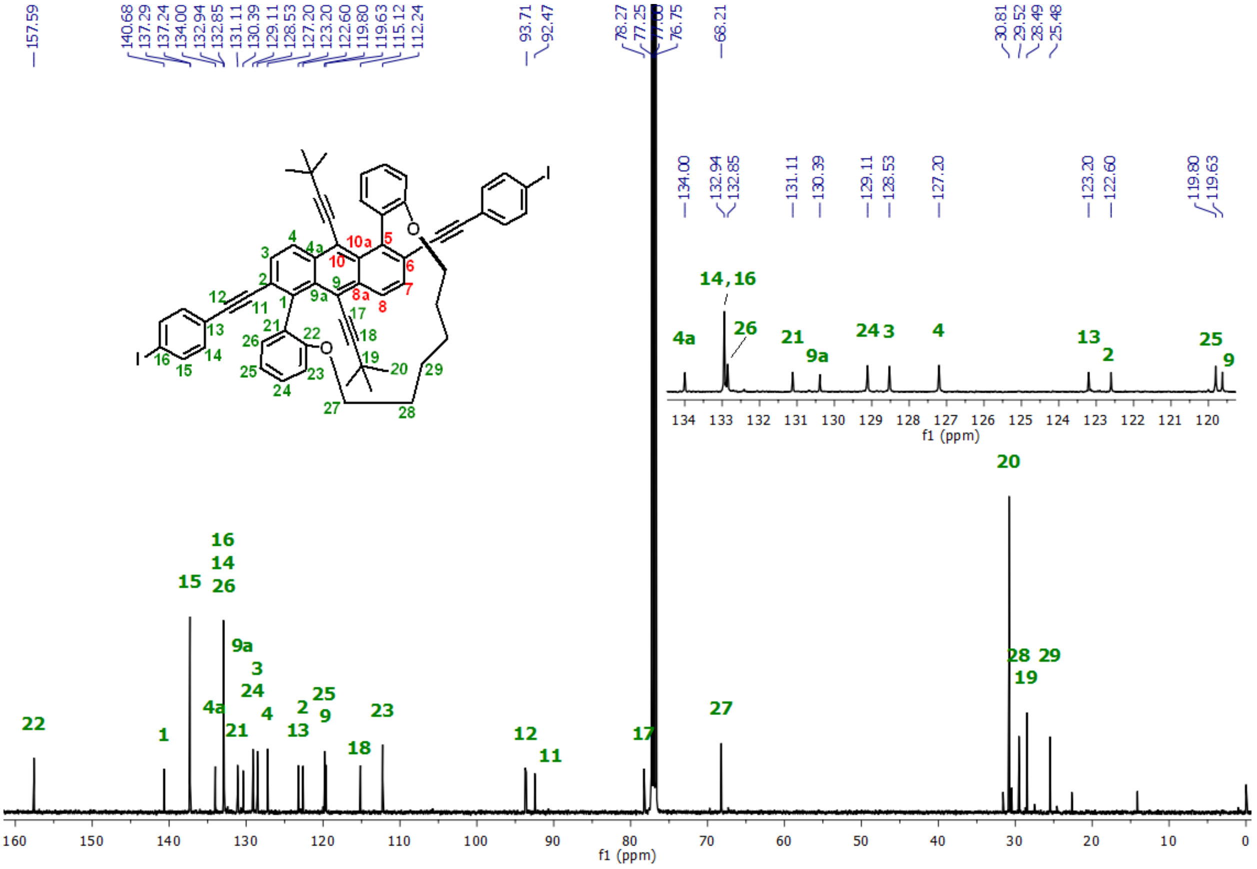

Supplementary Figure 99. ^{3}C NMR (101 MHz) of *P*-**9-C6** in $CDCl_3$, measured at 298 K.

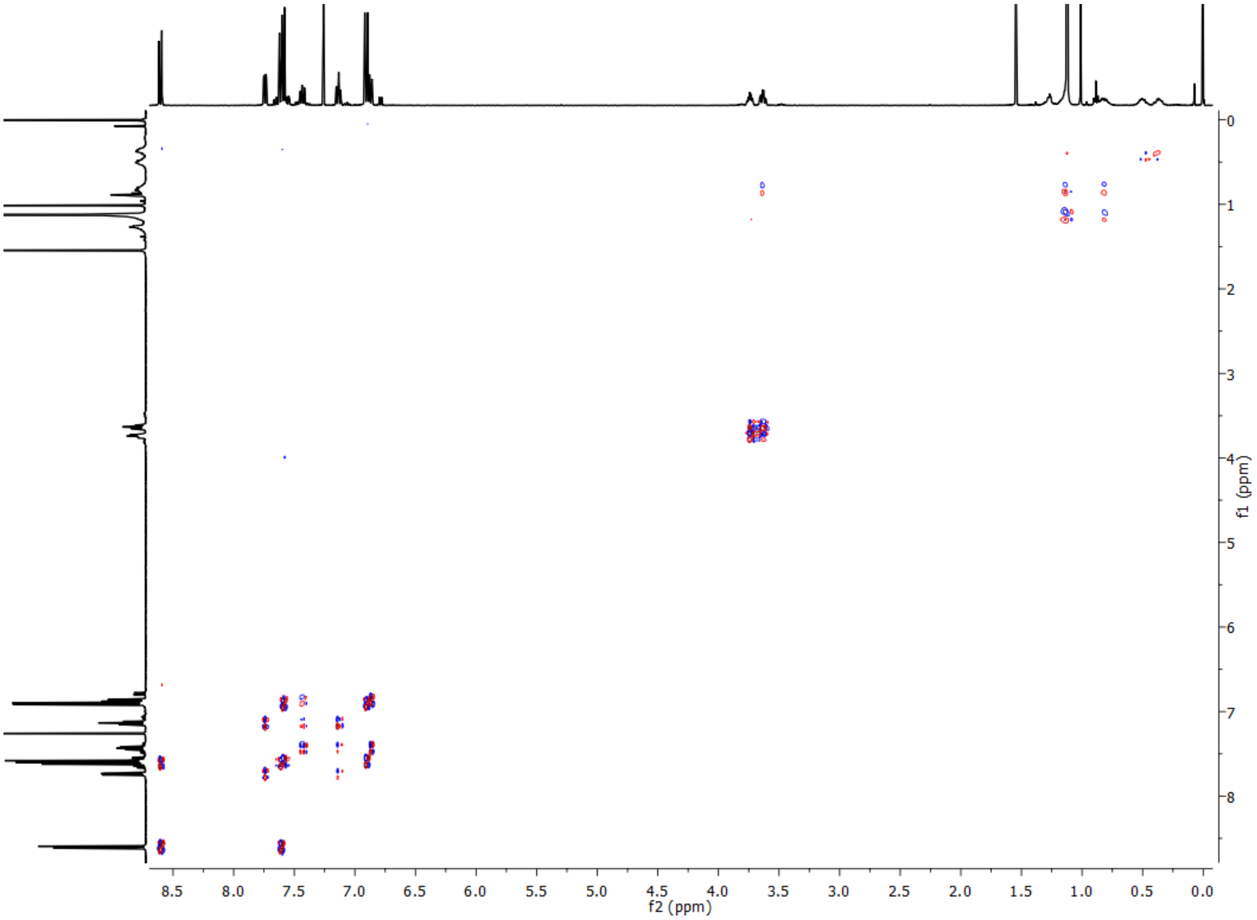

Supplementary Figure 100. COSY NMR (400 MHz) of ***P*-9-C6** in $CDCl_3$, measured at 298 K.

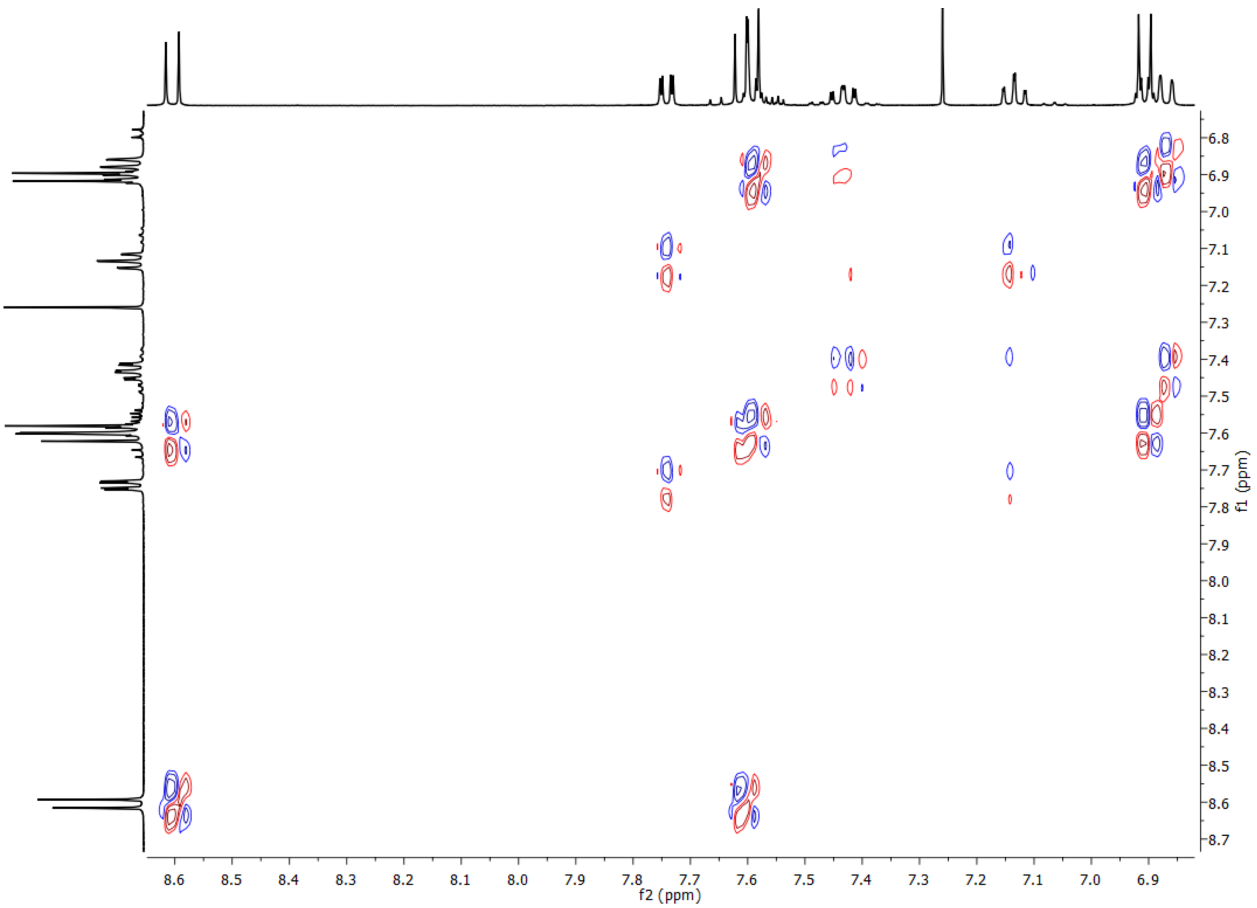

Supplementary Figure 101. COSY NMR (400 MHz) of *P*-**9-C6** in $CDCl_3$, measured at 298 K (expansion in aromatic region).

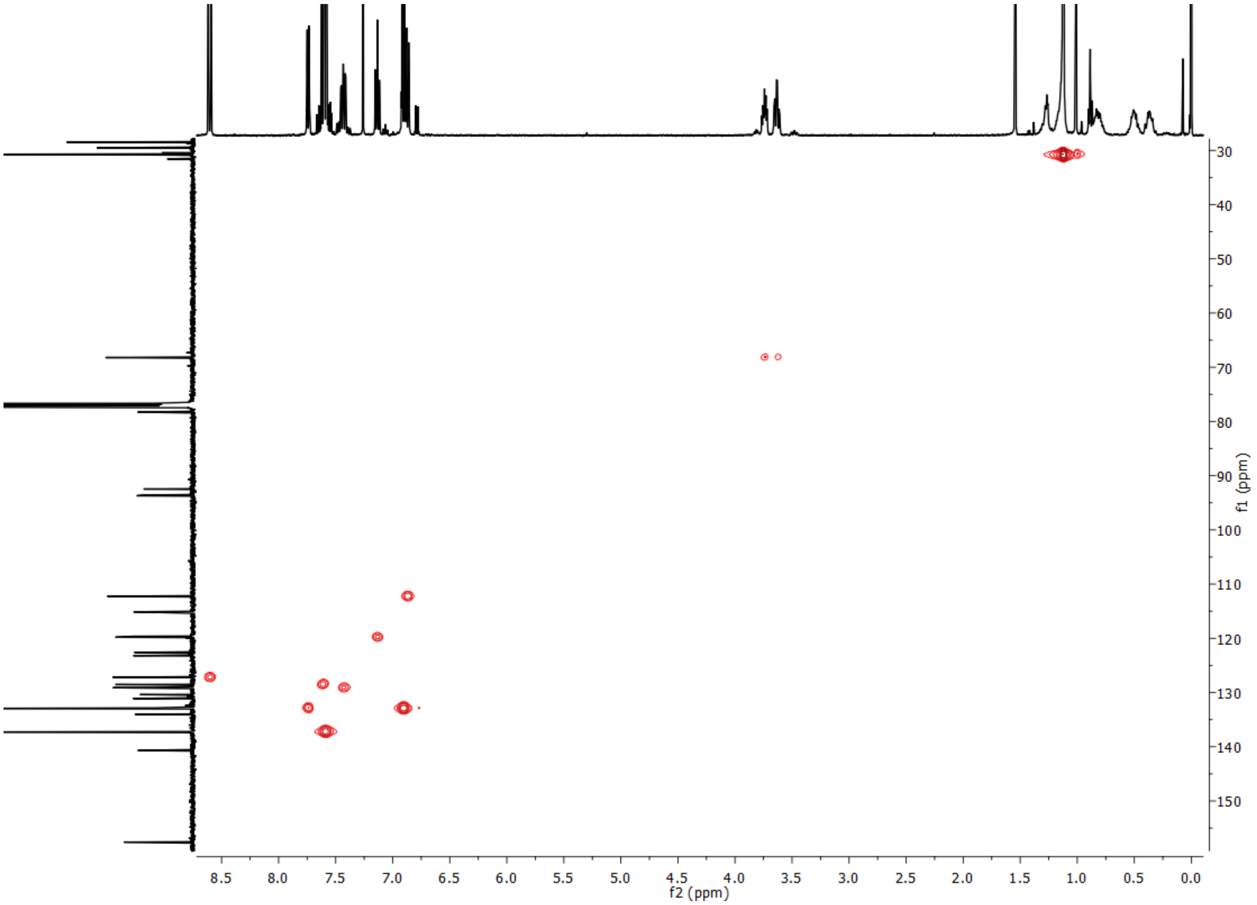

Supplementary Figure 102. HSQC NMR (400 MHz) of *P*-**9-C6** in $CDCl_3$, measured at 298 K.

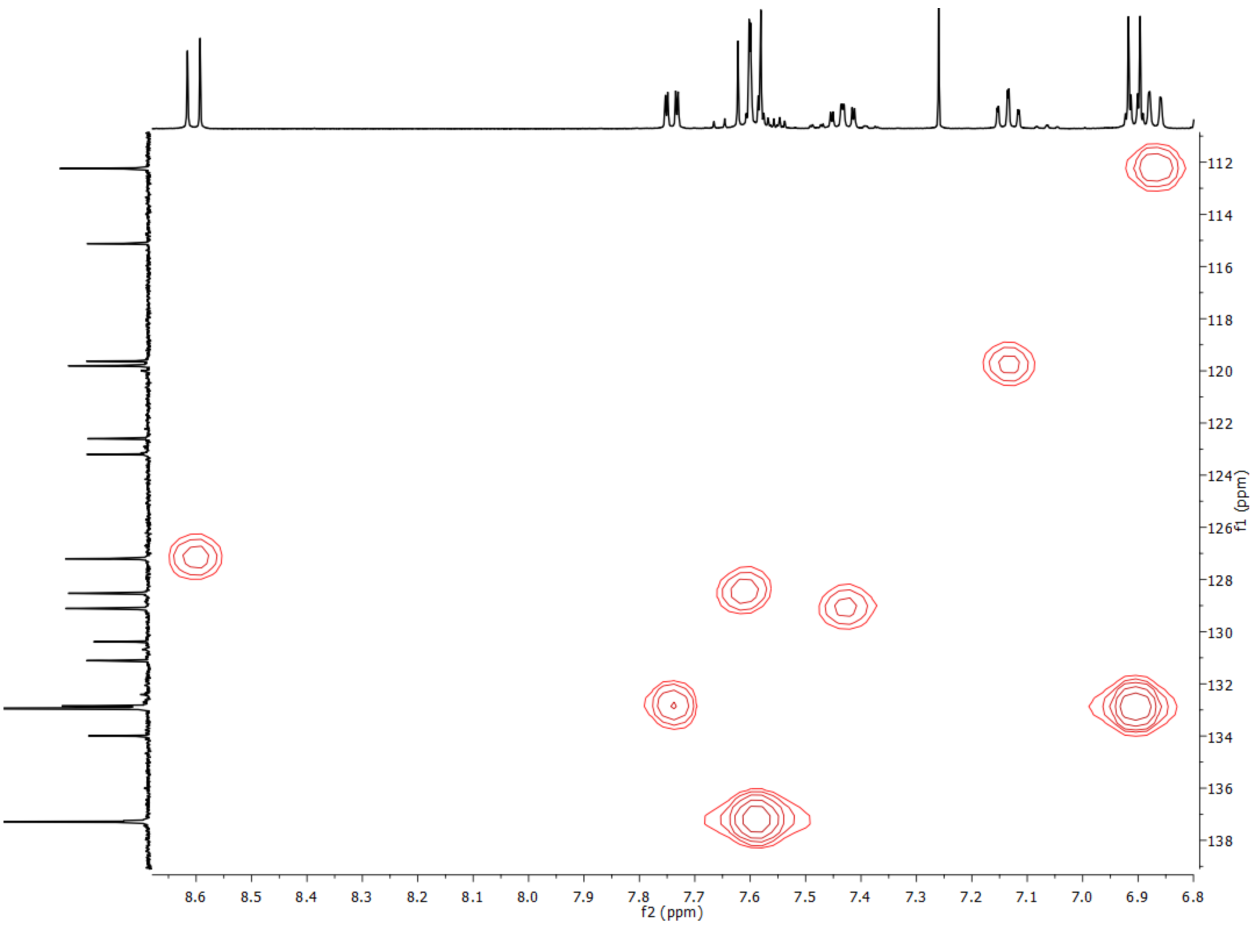

Supplementary Figure 103. HSQC NMR (400 MHz) of *P*-**9-C6** in $CDCl_3$, measured at 298 K (expansion in aromatic region).

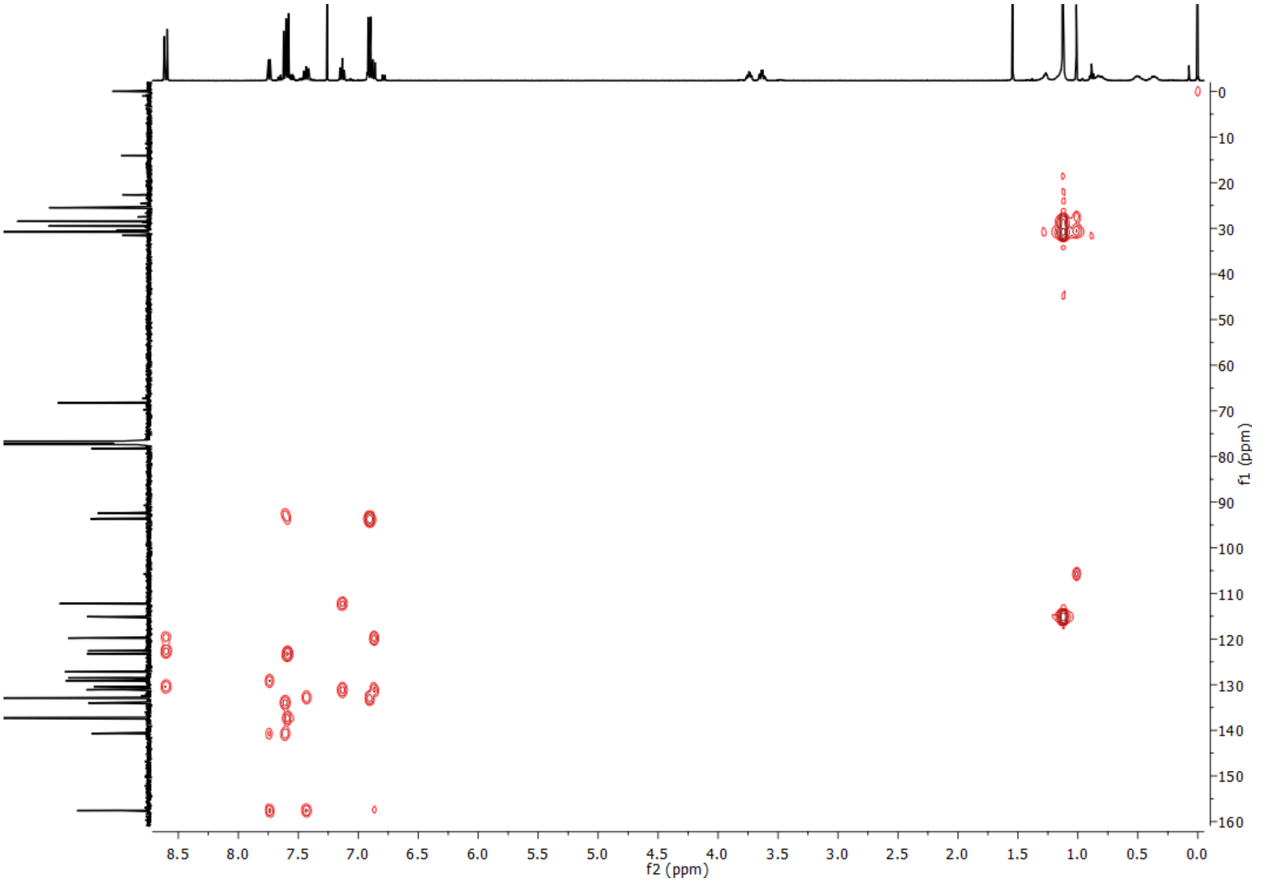

Supplementary Figure 104. HMBC NMR (400 MHz) of *P*-**9-C6** in $CDCl_3$, measured at 298 K.

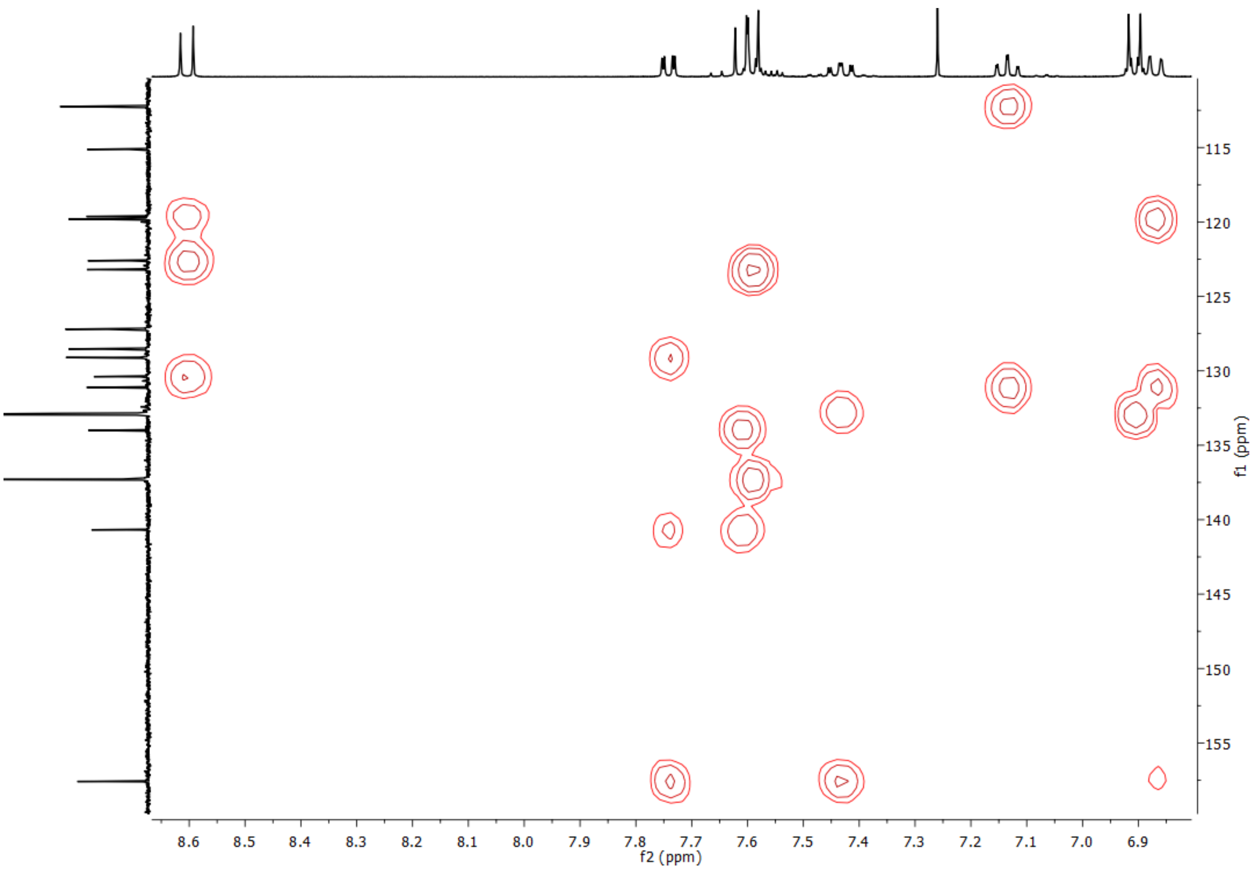

Supplementary Figure 105. HMBC NMR (400 MHz) of *P*-**9-C6** in $CDCl_3$, measured at 298 K (expansion in aromatic region).

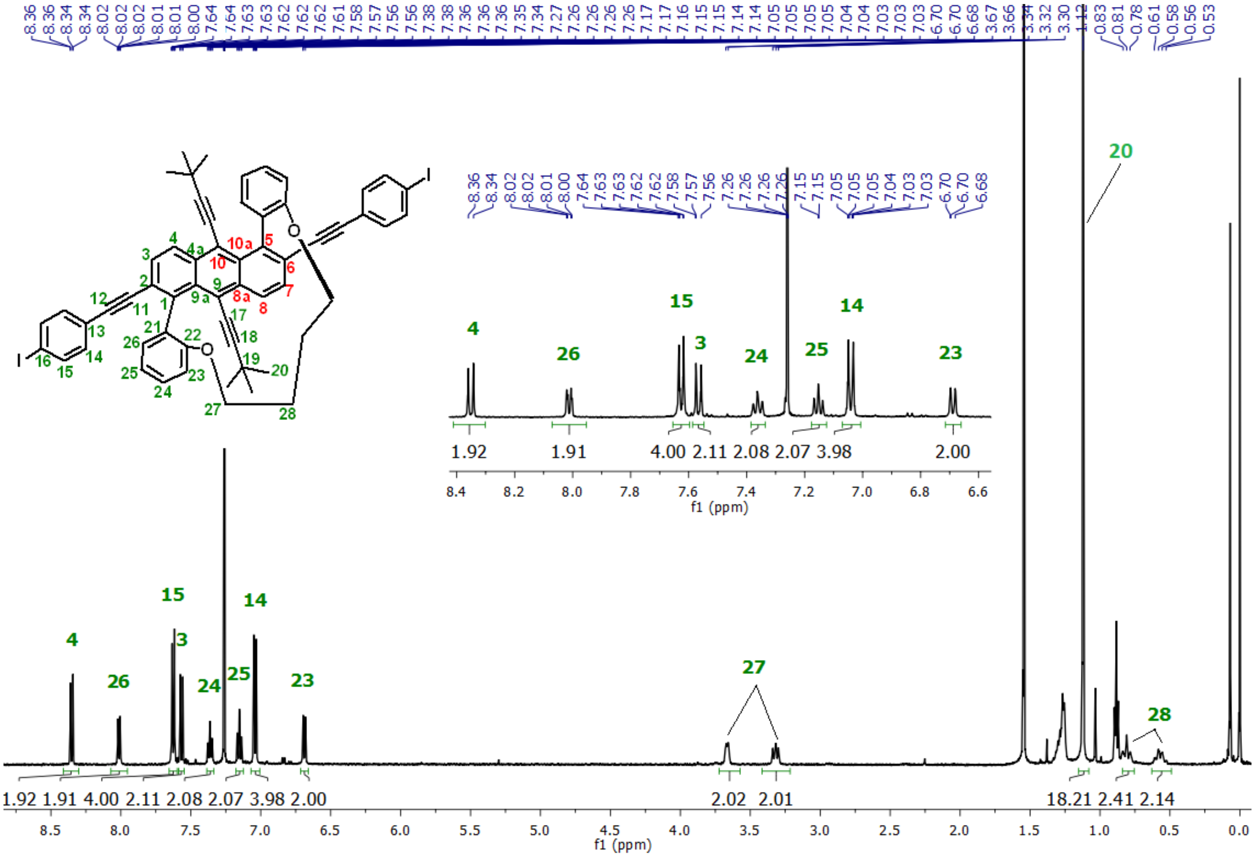

Supplementary Figure 106. ^{1}H NMR (500 MHz) of *P*-**9-C4** in $CDCl_3$, measured at 298 K.

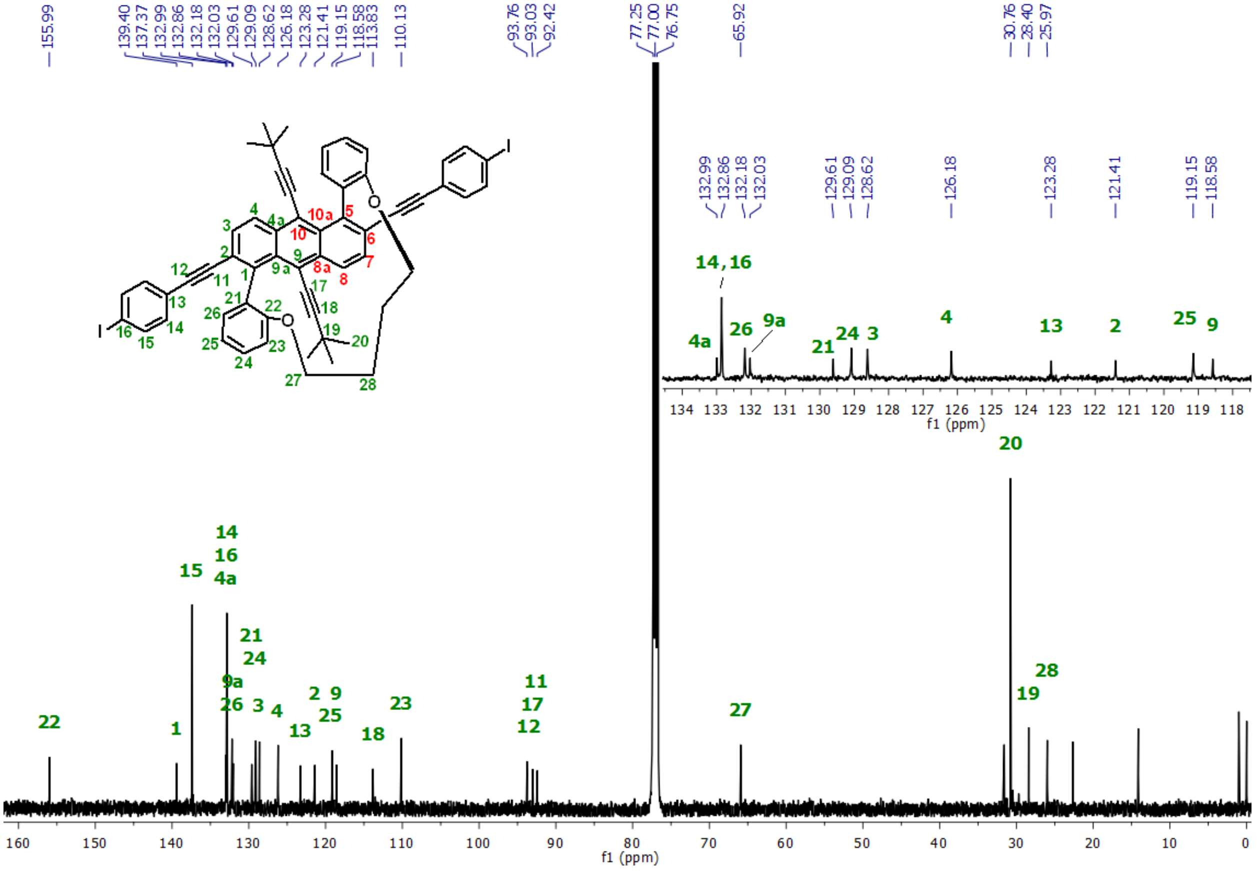

Supplementary Figure 107. ^{13}C NMR (126 MHz) of *P*-**9-C4** in $CDCl_3$, measured at 298 K.

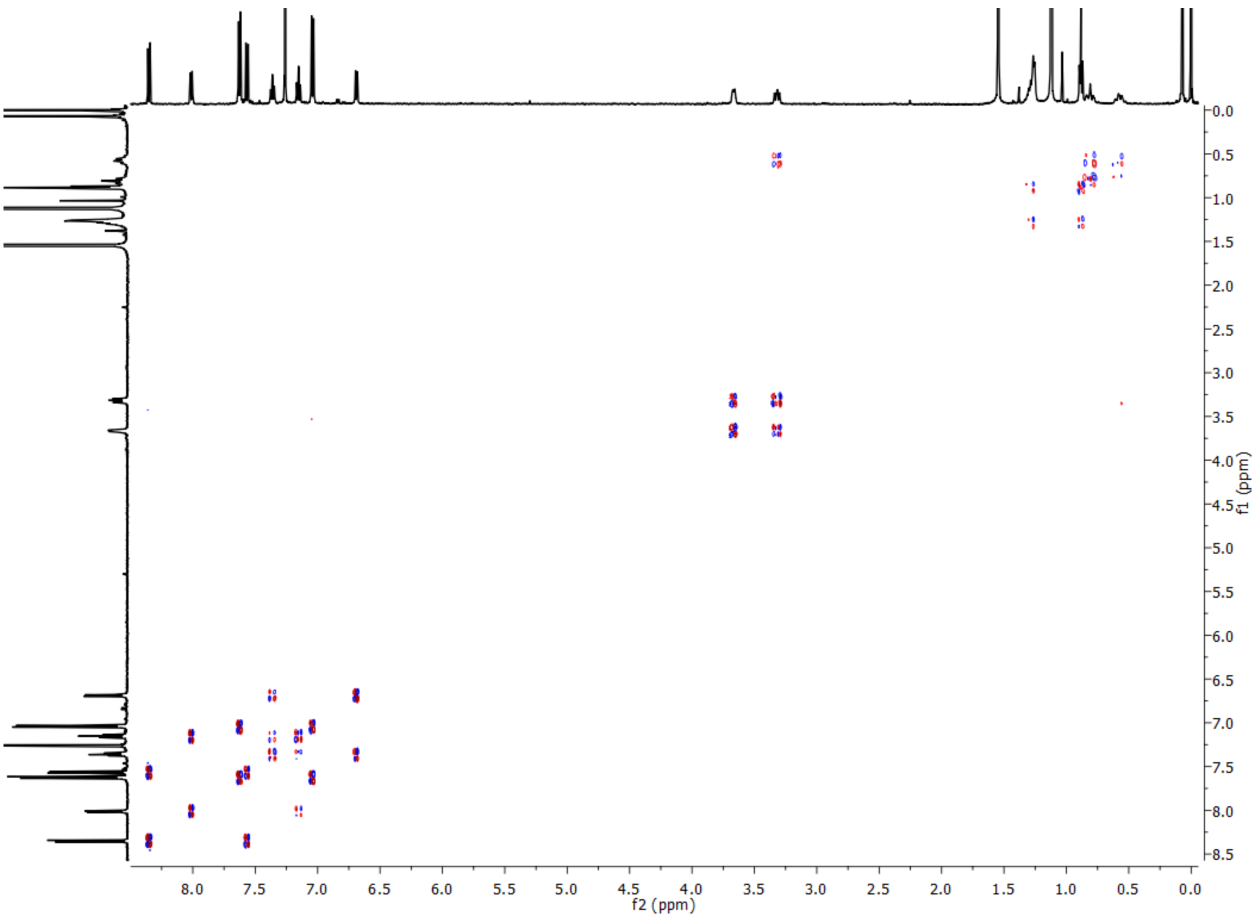

Supplementary Figure 108. COSY NMR (500 MHz) of ***P*-9-C4** in $CDCl_3$, measured at 298 K.

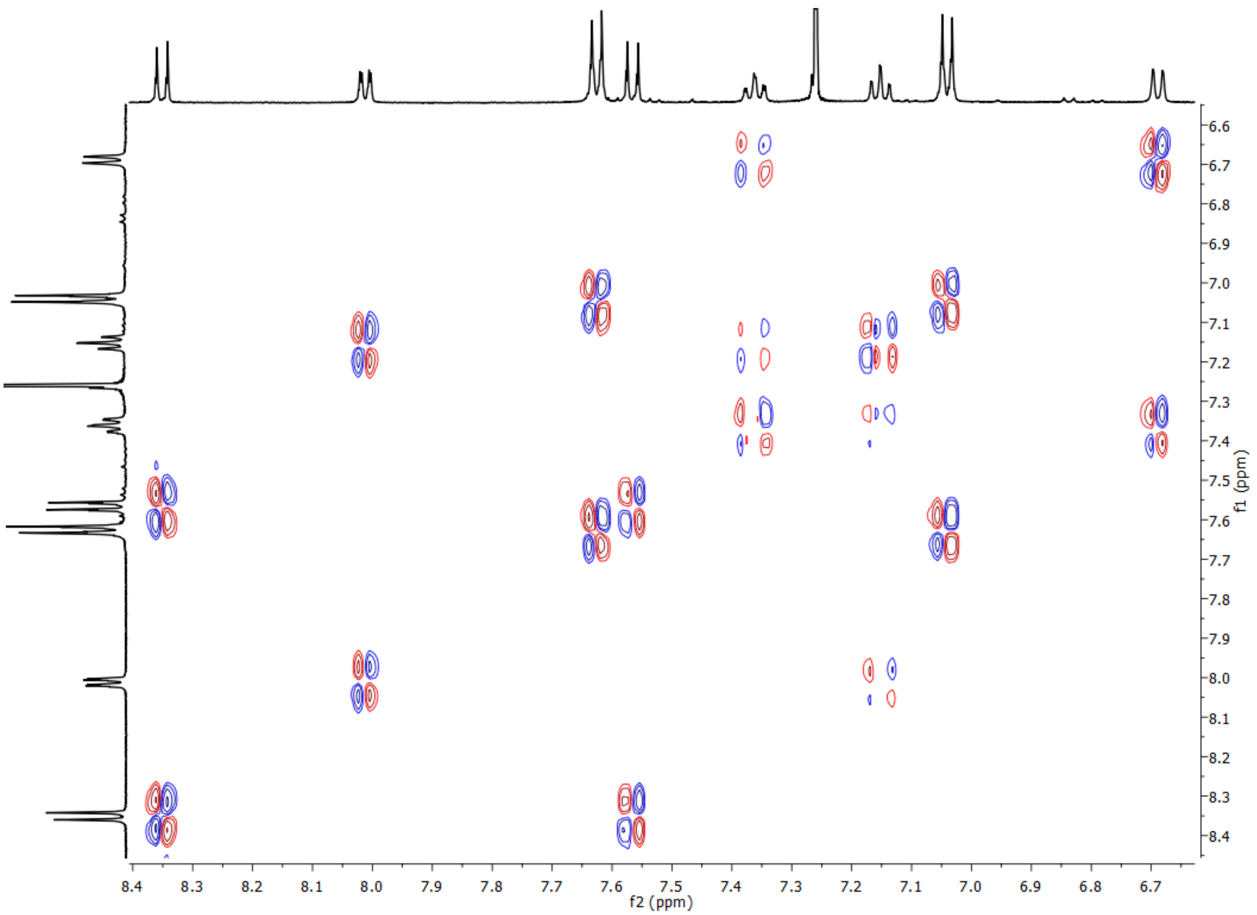

Supplementary Figure 109. COSY NMR (500 MHz) of ***P*-9-C4** in $CDCl_3$, measured at 298 K (expansion in aromatic region).

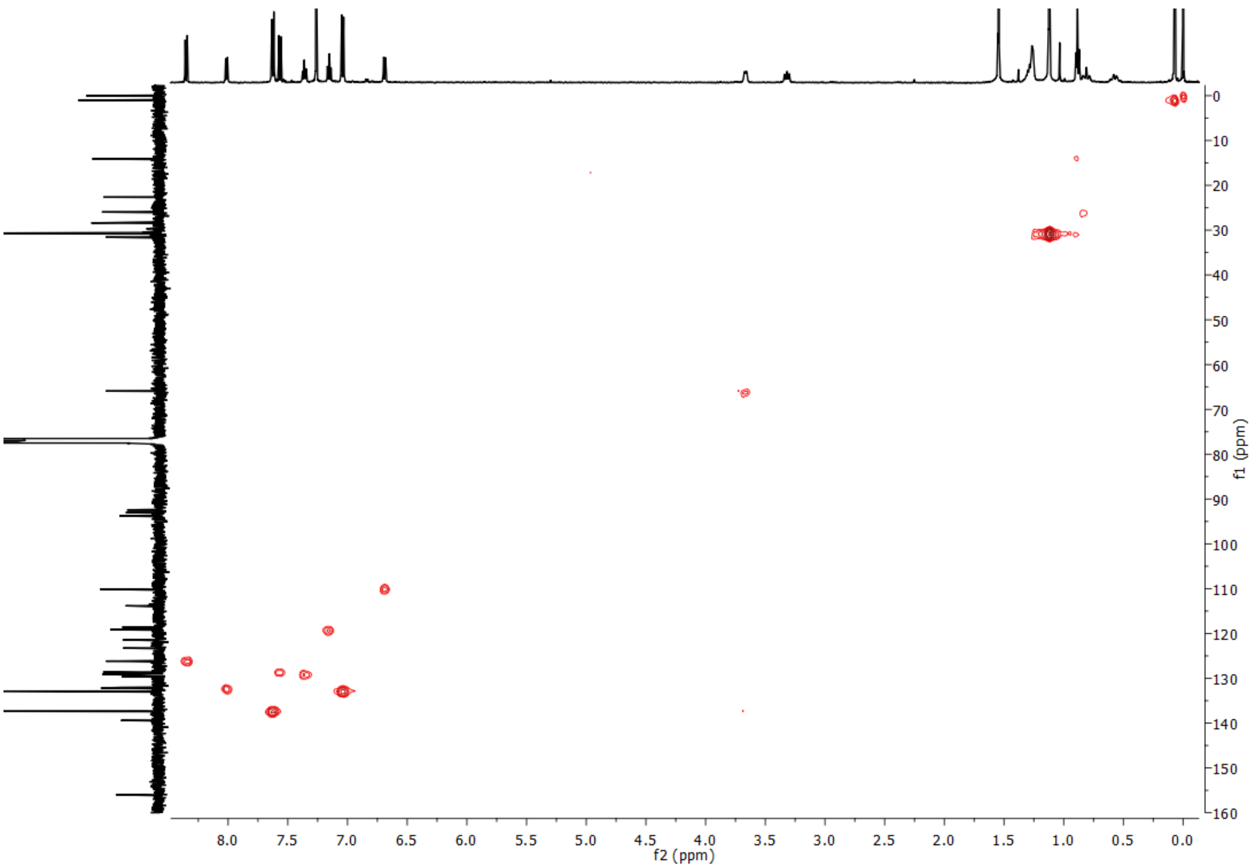

Supplementary Figure 110. HSQC NMR (500 MHz) of ***P*-9-C4** in $CDCl_3$, measured at 298 K.

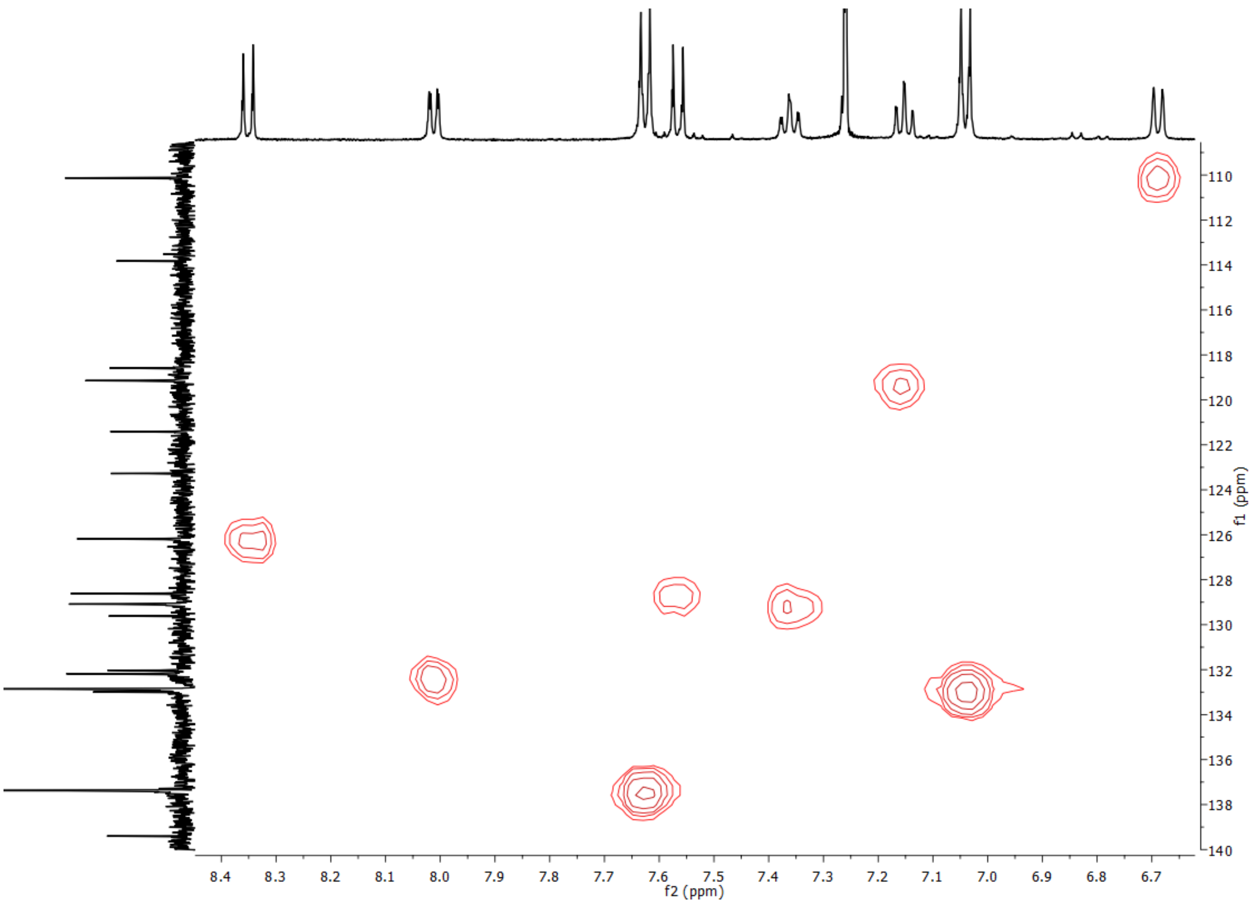

Figure 111. HSQC NMR (500 MHz) of *P*-**9-C4** in $CDCl_3$, measured at 298 K (expansion in aromatic region).

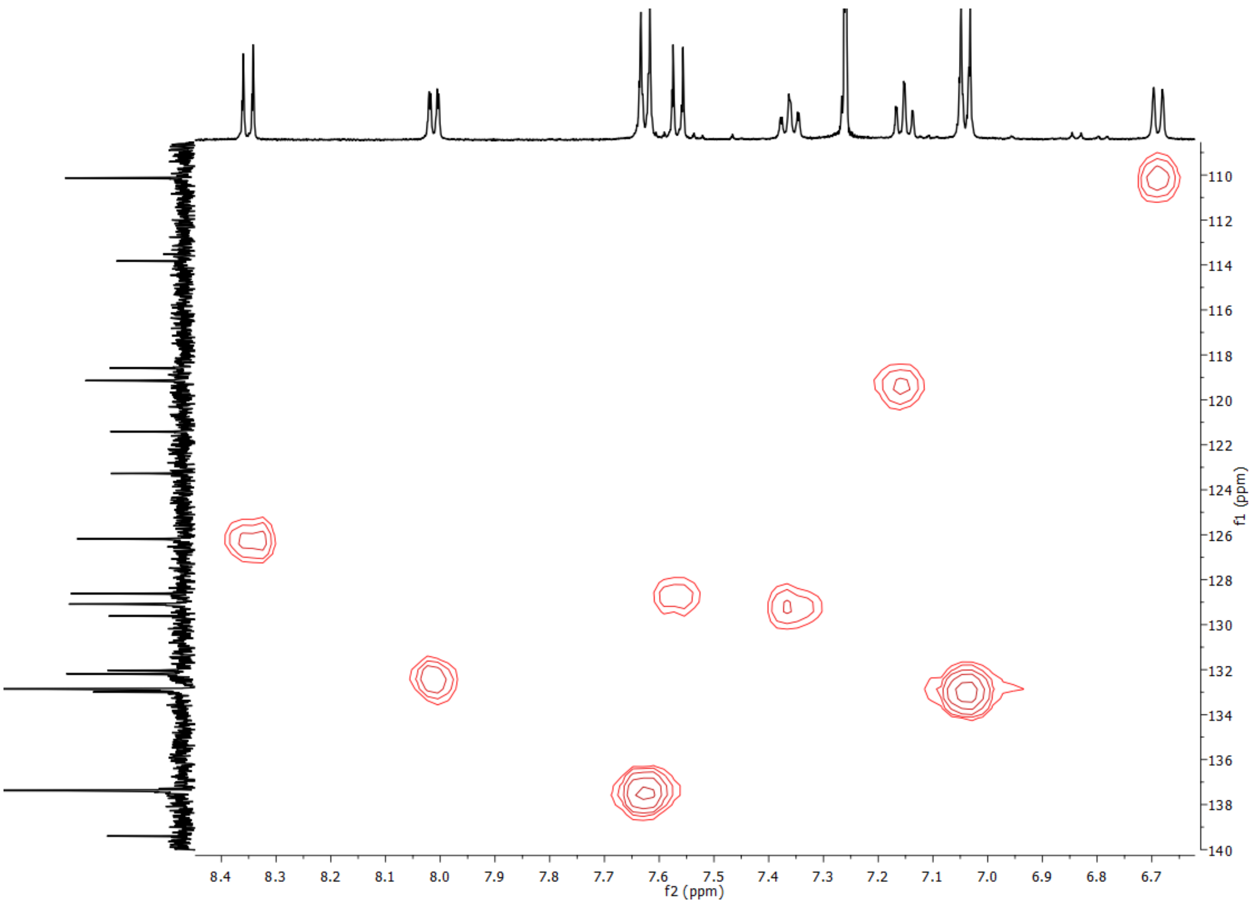

Supplementary Figure 112. HMBC NMR (500 MHz) of *P*-**9-C4** in $CDCl_3$, measured at 298 K.

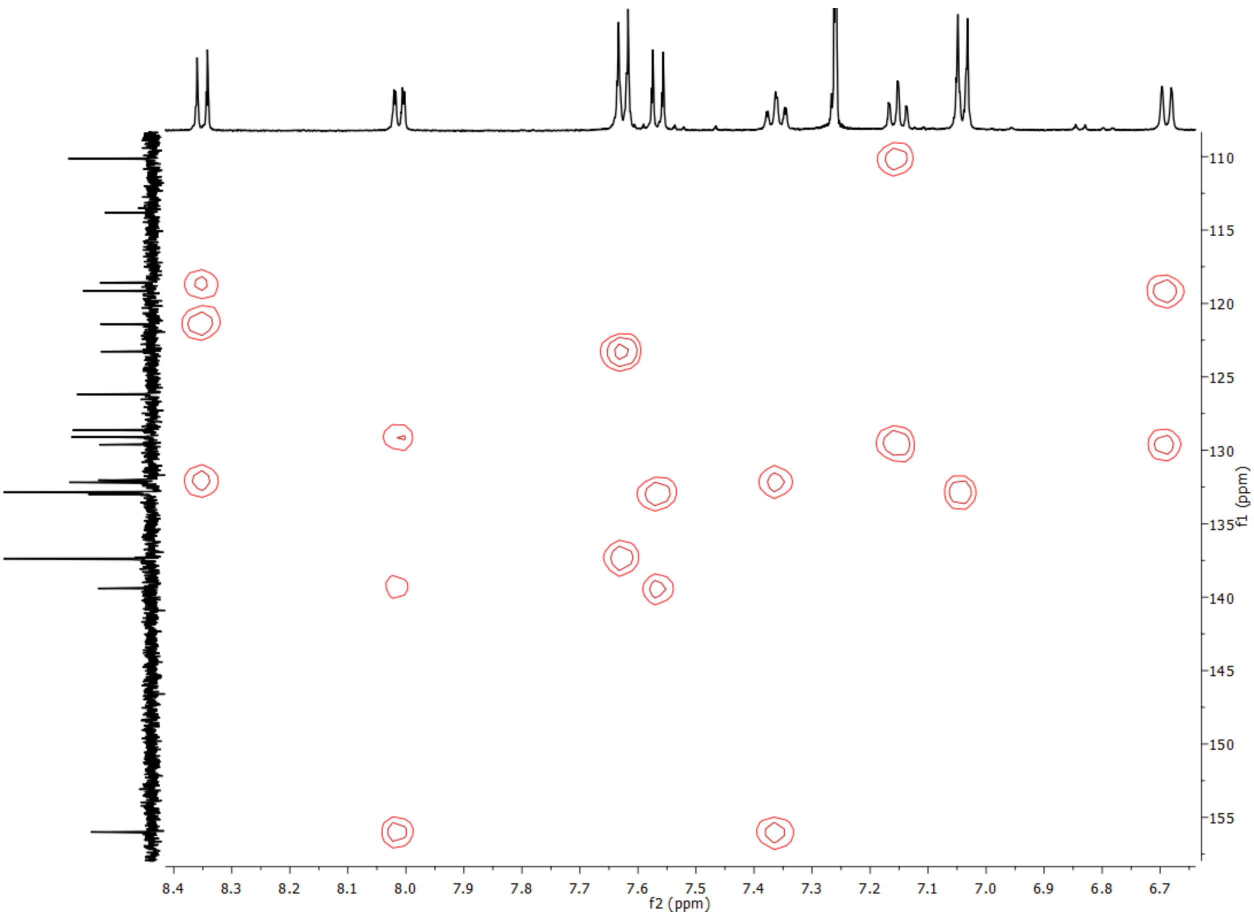

Supplementary Figure 113. HMBC NMR (500 MHz) of ***P*-9-C4** in $CDCl_3$, measured at 298 K (expansion in aromatic region).

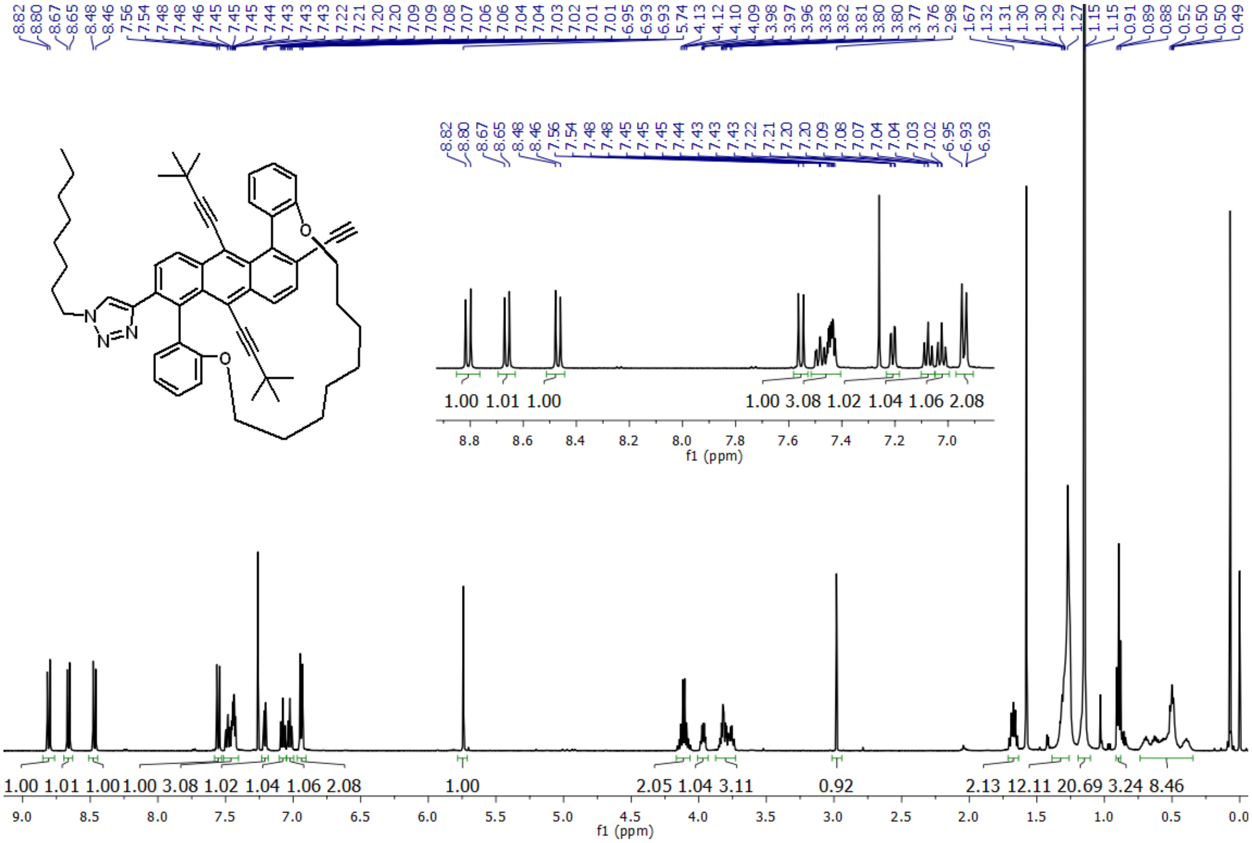

Supplementary Figure 114. ^{1}H NMR (500 MHz) of *P*-**10-C8** in $CDCl_3$, measured at 298 K.

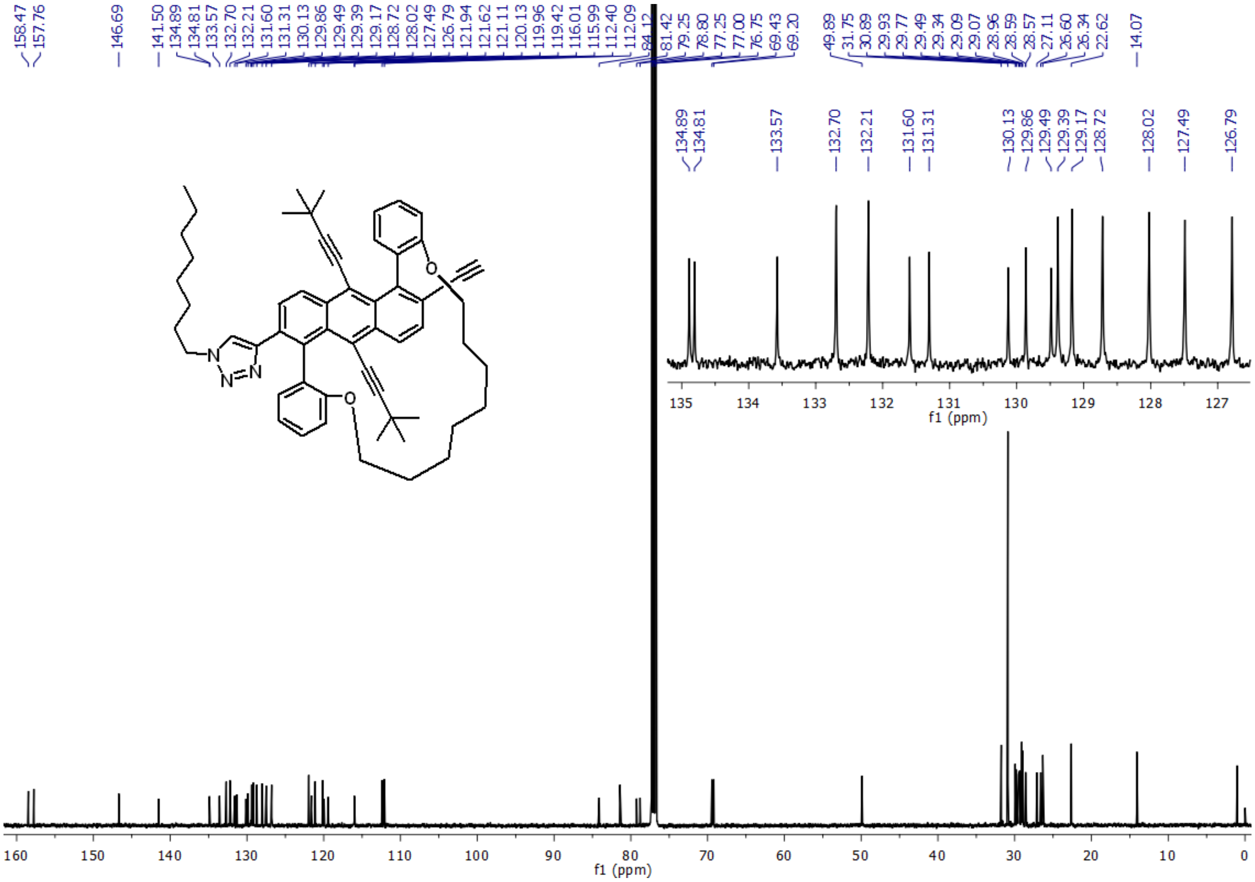

Supplementary Figure 115. ^{13}C NMR (126 MHz) of ***P*-10-C8** in $CDCl_3$, measured at 298 K.

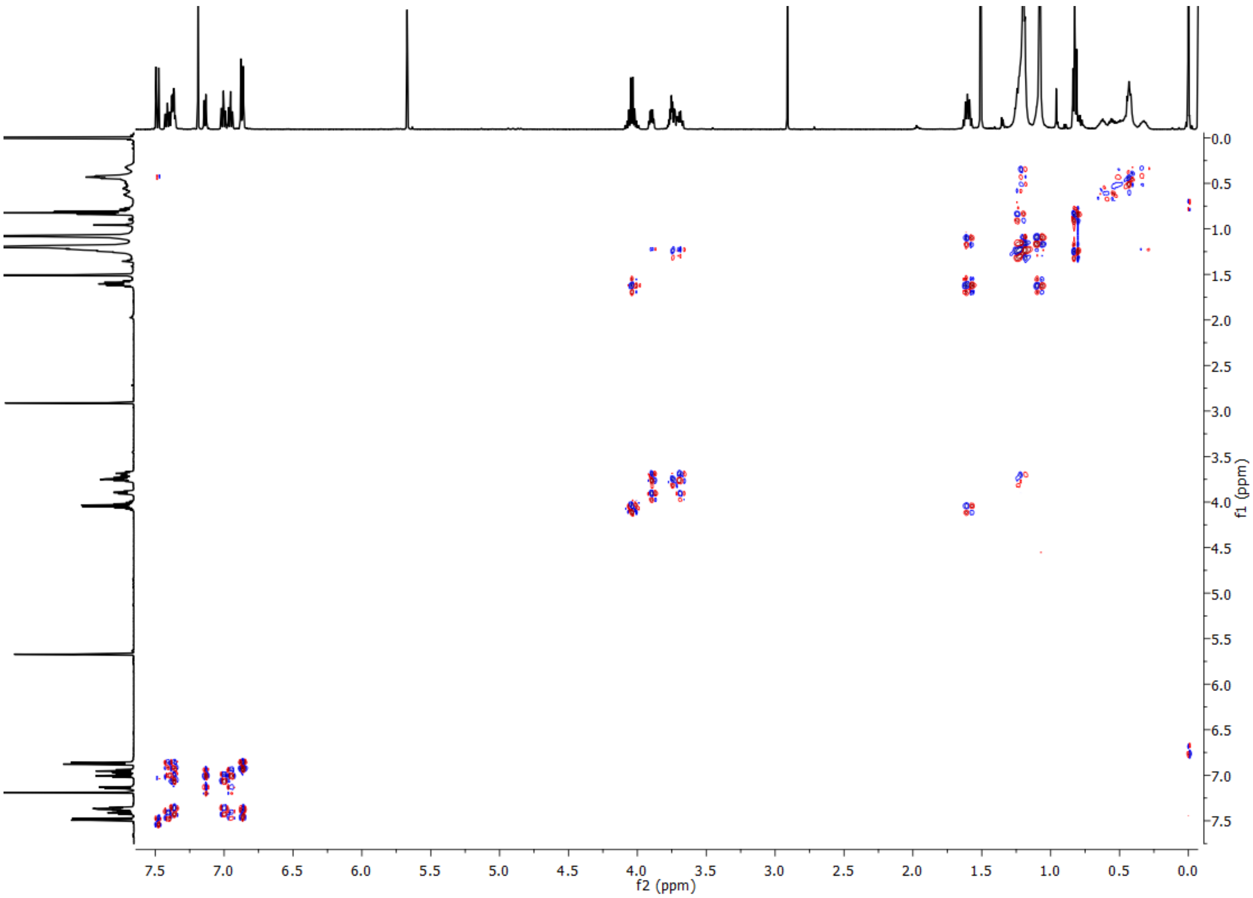

Supplementary Figure 116. COSY NMR (500 MHz) of ***P*-10-C8** in $CDCl_3$, measured at 298 K.

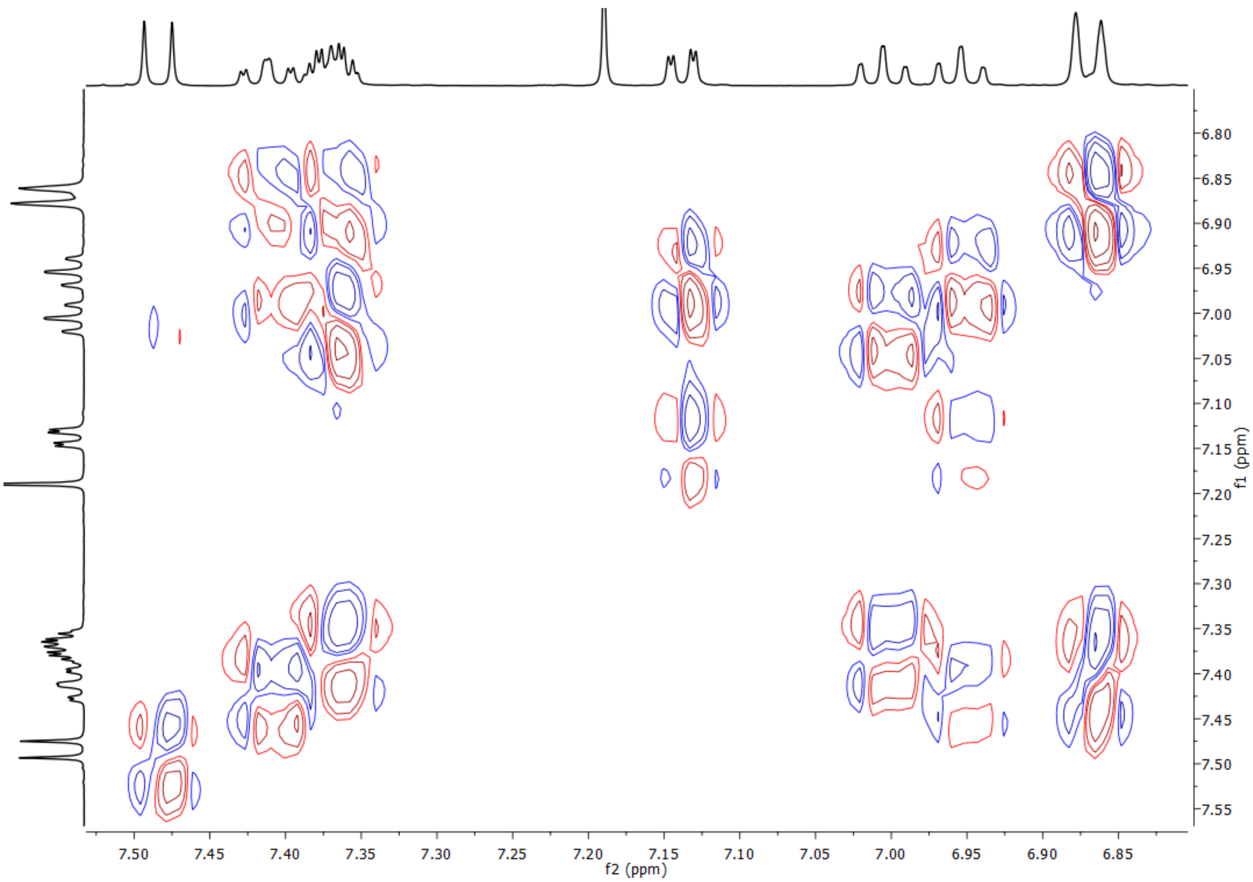

Supplementary Figure 117. COSY NMR (500 MHz) of ***P*-10-C8** in $CDCl_3$, measured at 298 K (expansion in aromatic region).

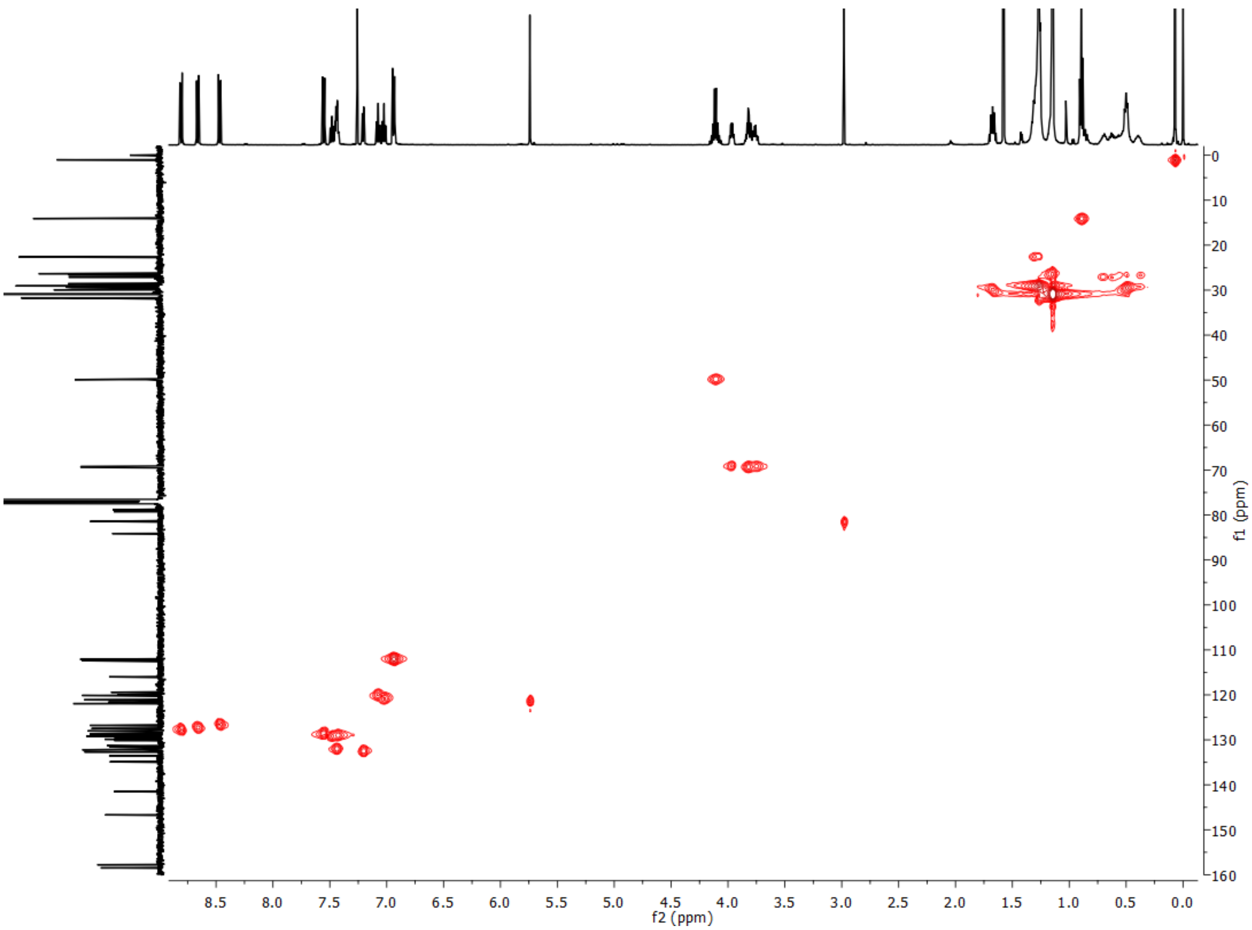

Supplementary Figure 118. HSQC NMR (500 MHz) of ***P*-10-C8** in $CDCl_3$, measured at 298 K.

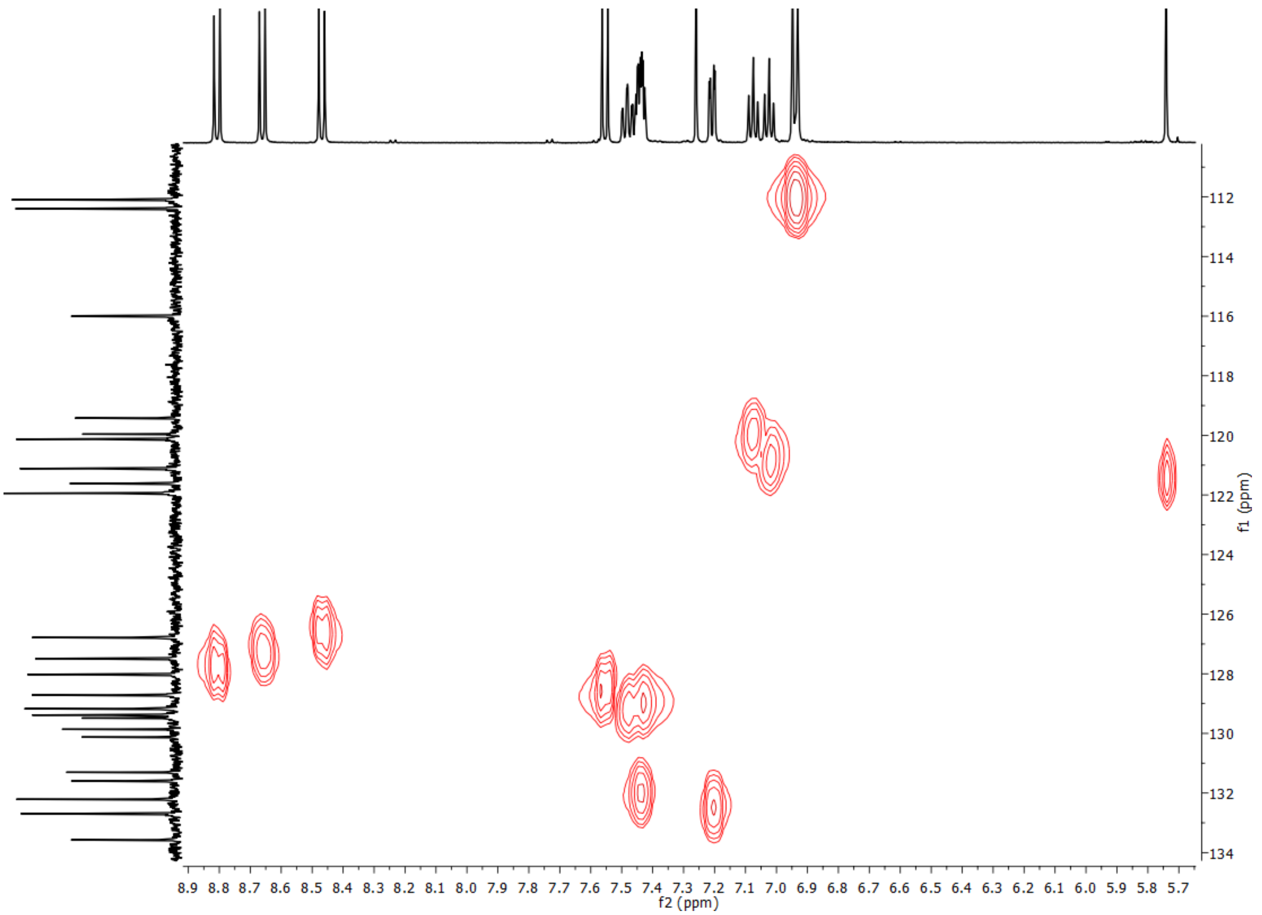

Figure 119. HSQC NMR (500 MHz) of ***P*-10-C8** in $CDCl_3$, measured at 298 K (expansion in aromatic region).

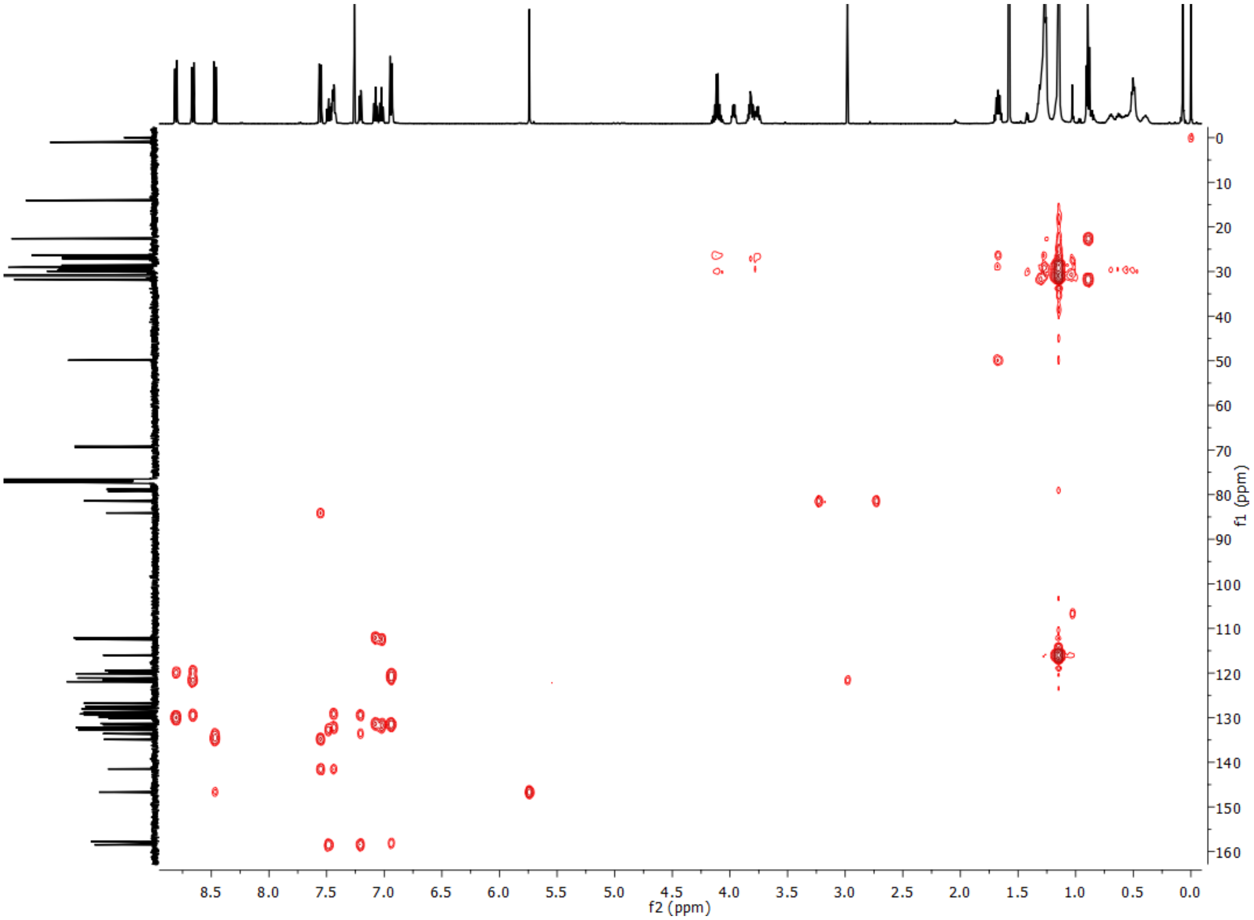

Supplementary Figure 120. HMBC NMR (500 MHz) of ***P*-10-C8** in $CDCl_3$, measured at 298 K.

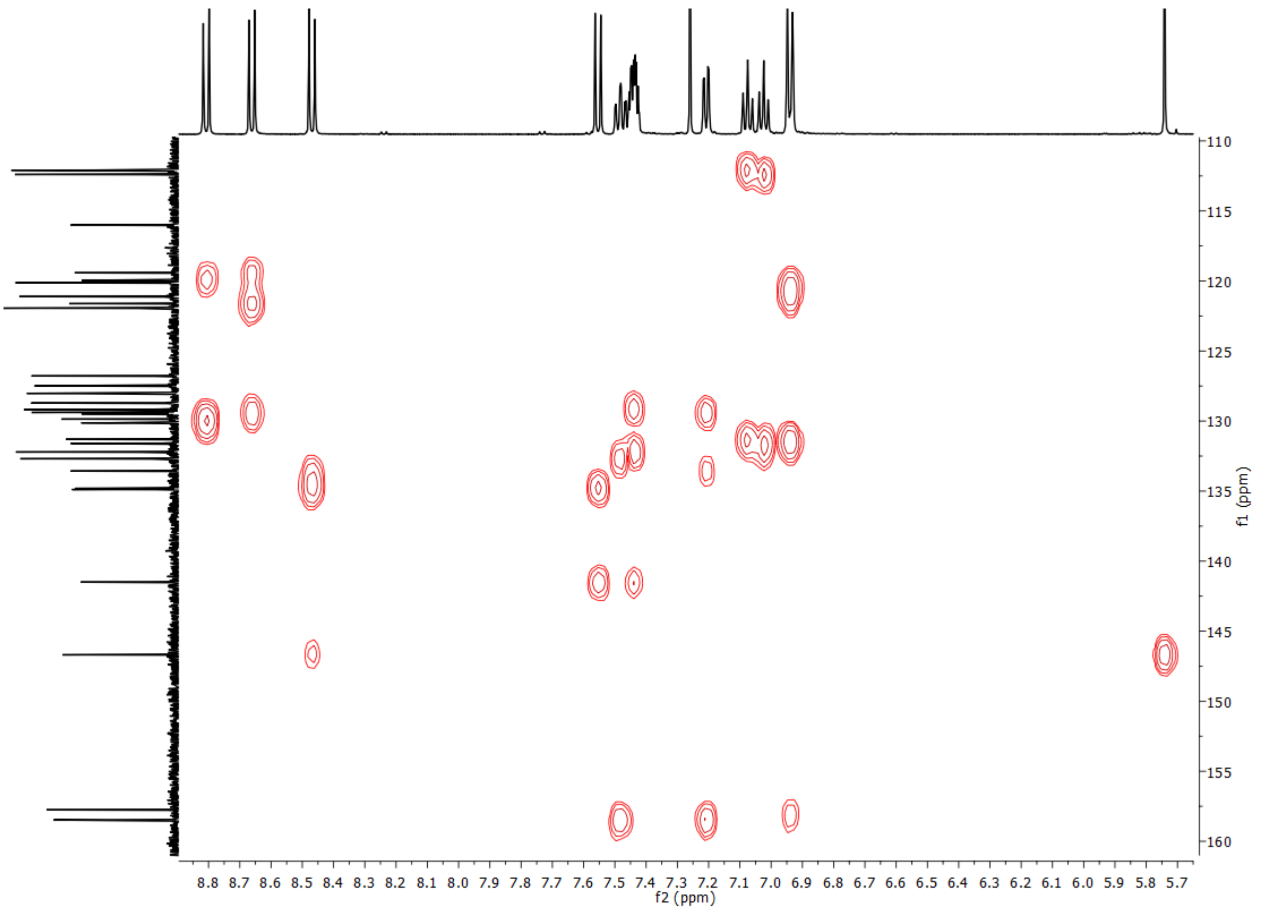

Supplementary Figure 121. HMBC NMR (500 MHz) of ***P*-10-C8** in $CDCl_3$, measured at 298 K (expansion in aromatic region).

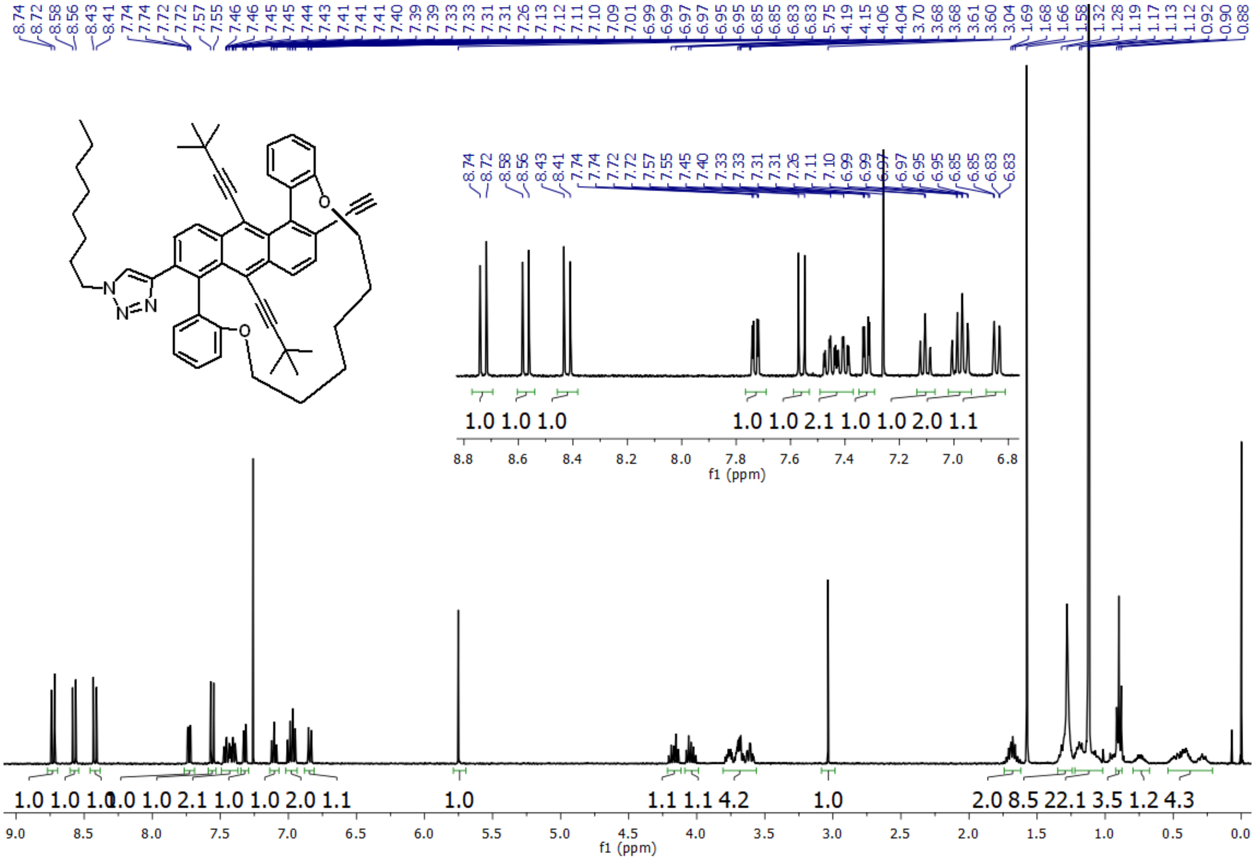

Supplementary Figure 122. ^{1}H NMR (400 MHz) of ***P*-10-C6** in $CDCl_3$, measured at 298 K.

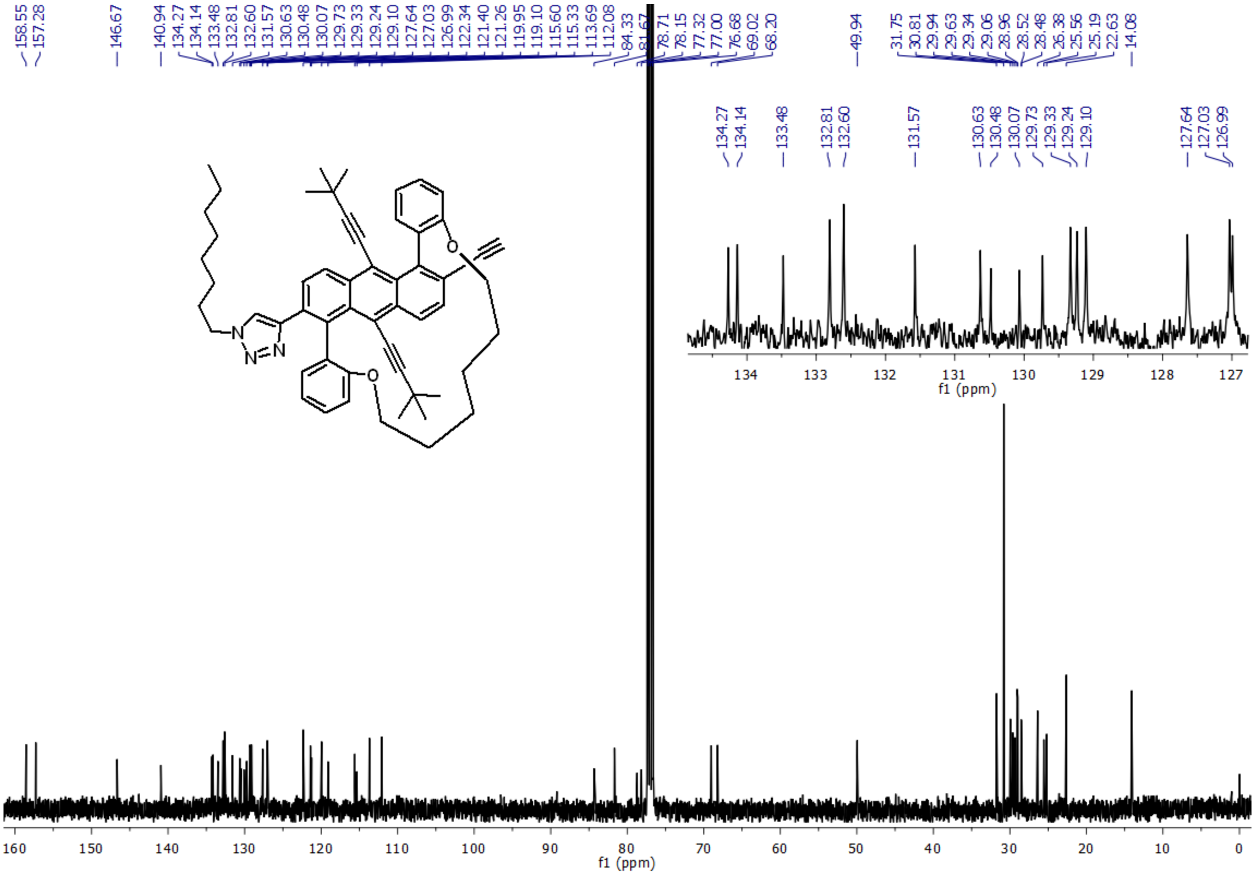

Supplementary Figure 123. ^{13}C NMR (101 MHz) of *P*-**10-C6** in $CDCl_3$, measured at 298 K.

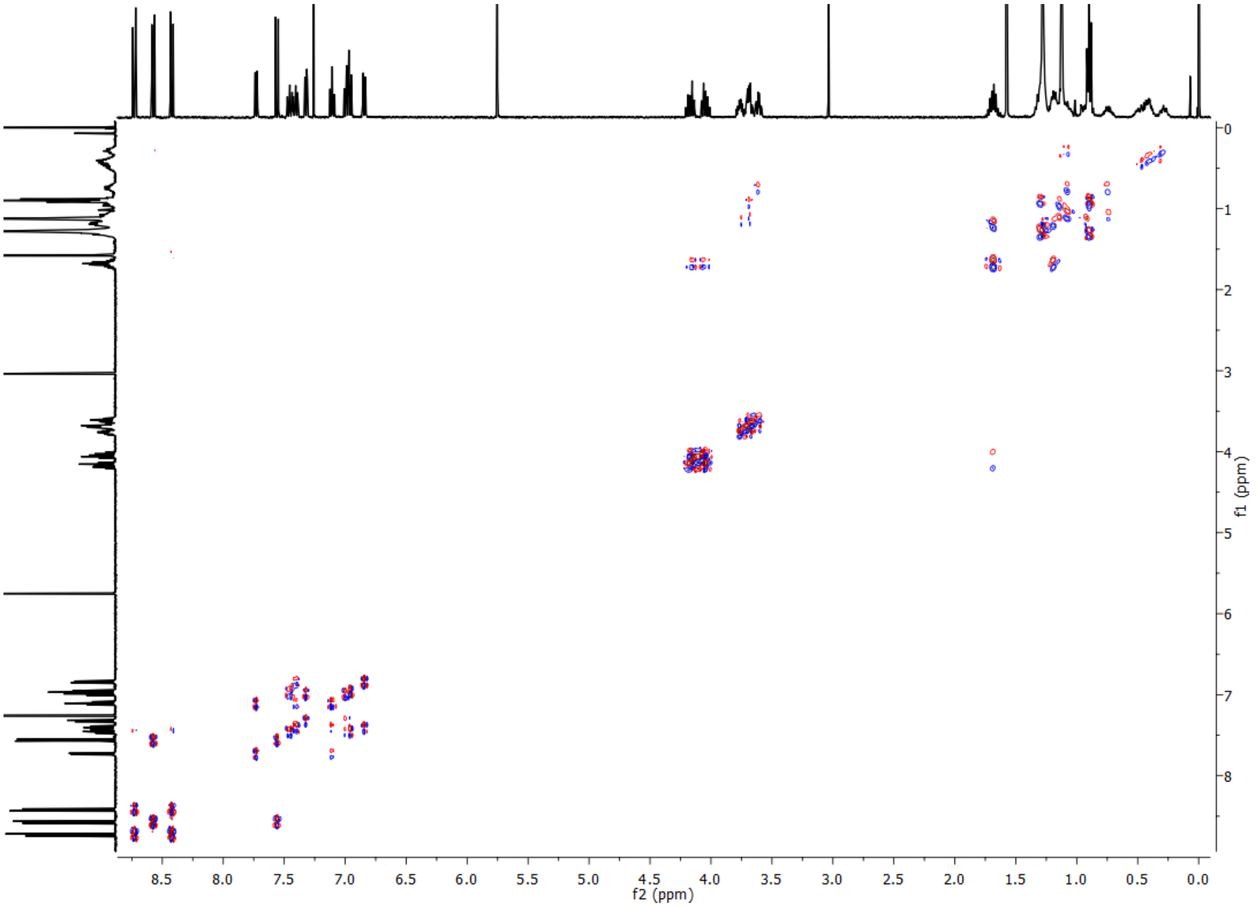

Supplementary Figure 124. COSY NMR (400 MHz) of ***P*-10-C6** in $CDCl_3$, measured at 298 K.

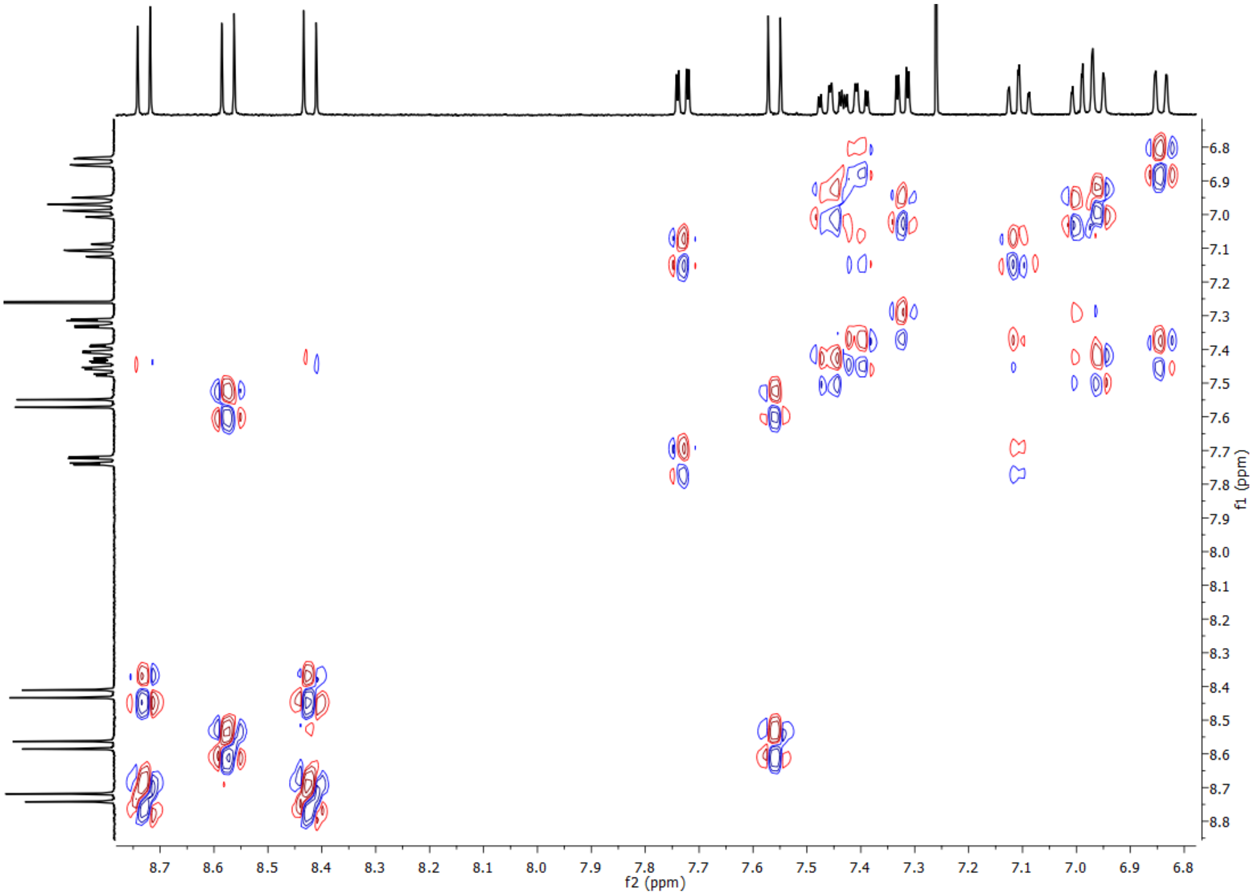

Supplementary Figure 125. COSY NMR (400 MHz) of *P*-**10-C6** in $CDCl_3$, measured at 298 K (expansion in aromatic region).

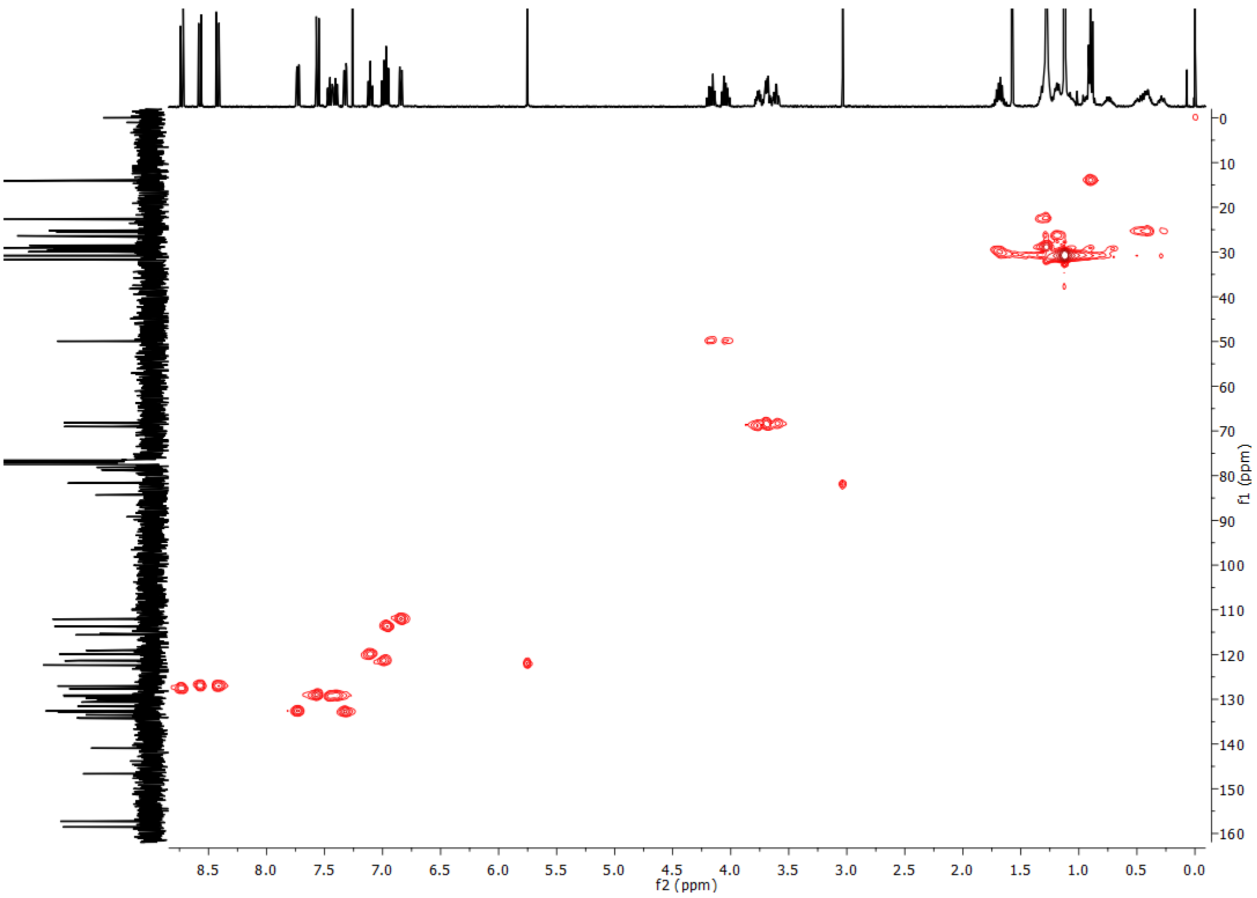

Supplementary Figure 126. HSQC NMR (400 MHz) of ***P*-10-C6** in $CDCl_3$, measured at 298 K.

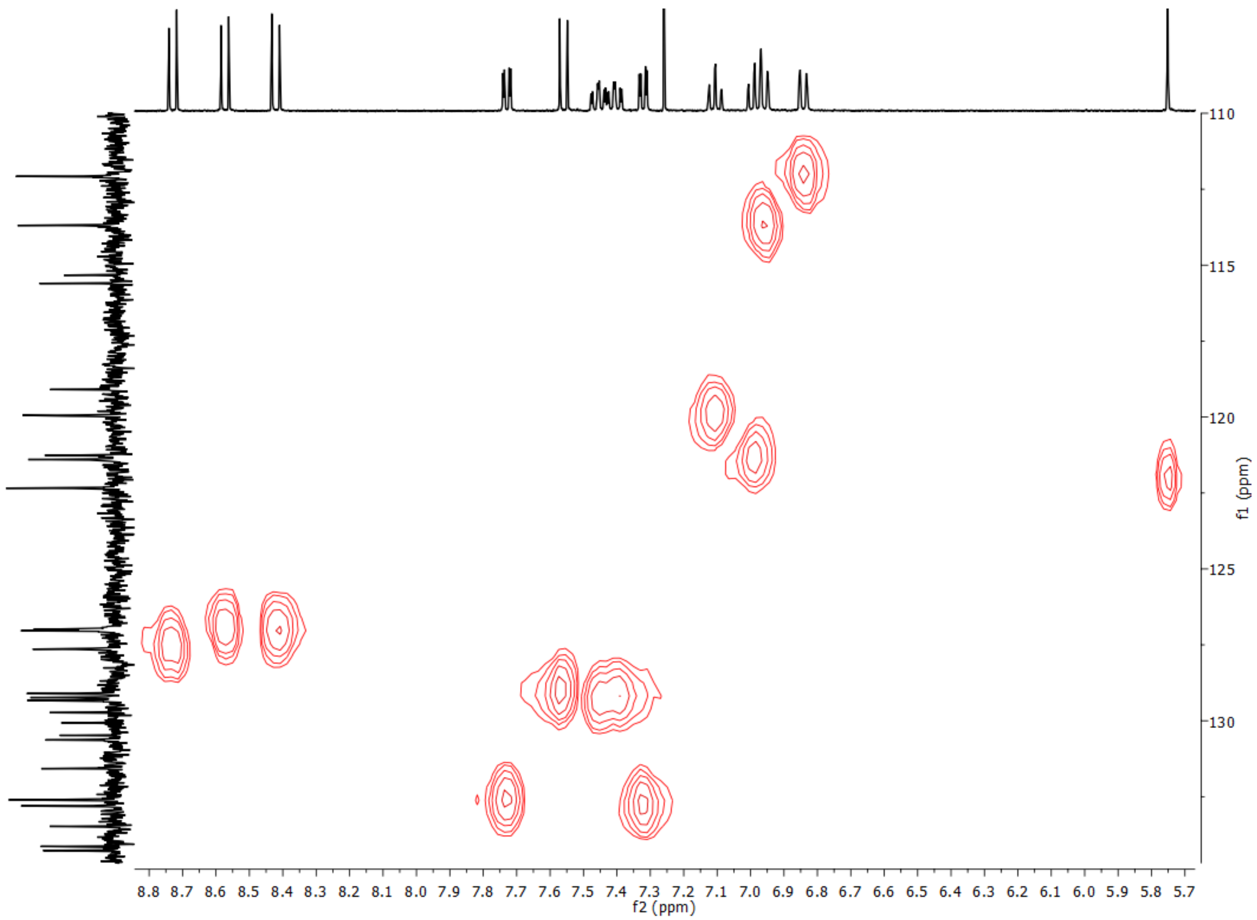

Supplementary Figure 127. HSQC NMR (400 MHz) of *P*-**10-C6** in $CDCl_3$, measured at 298 K (expansion in aromatic region).

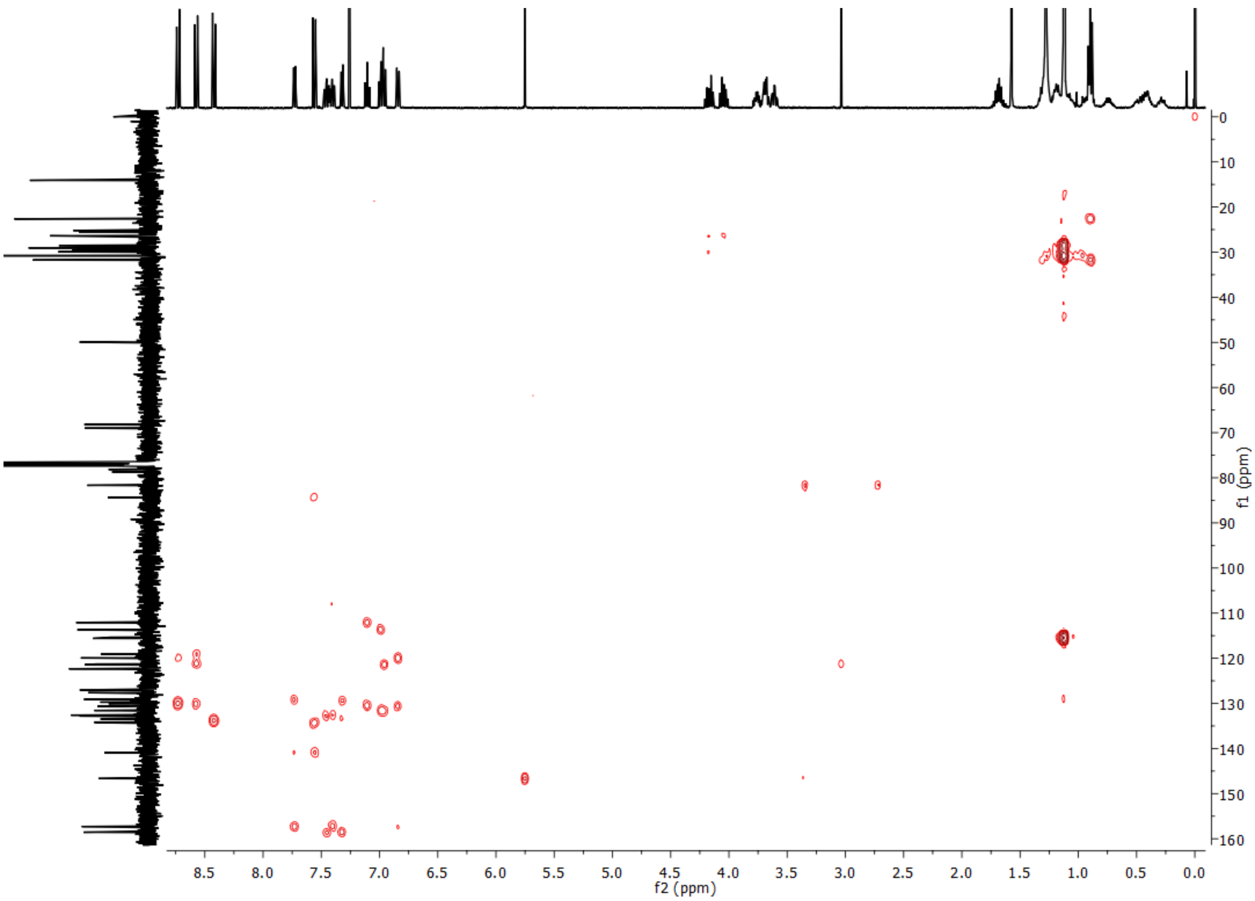

Supplementary Figure 128. HMBC NMR (400 MHz) of ***P*-10-C6** in $CDCl_3$, measured at 298 K.

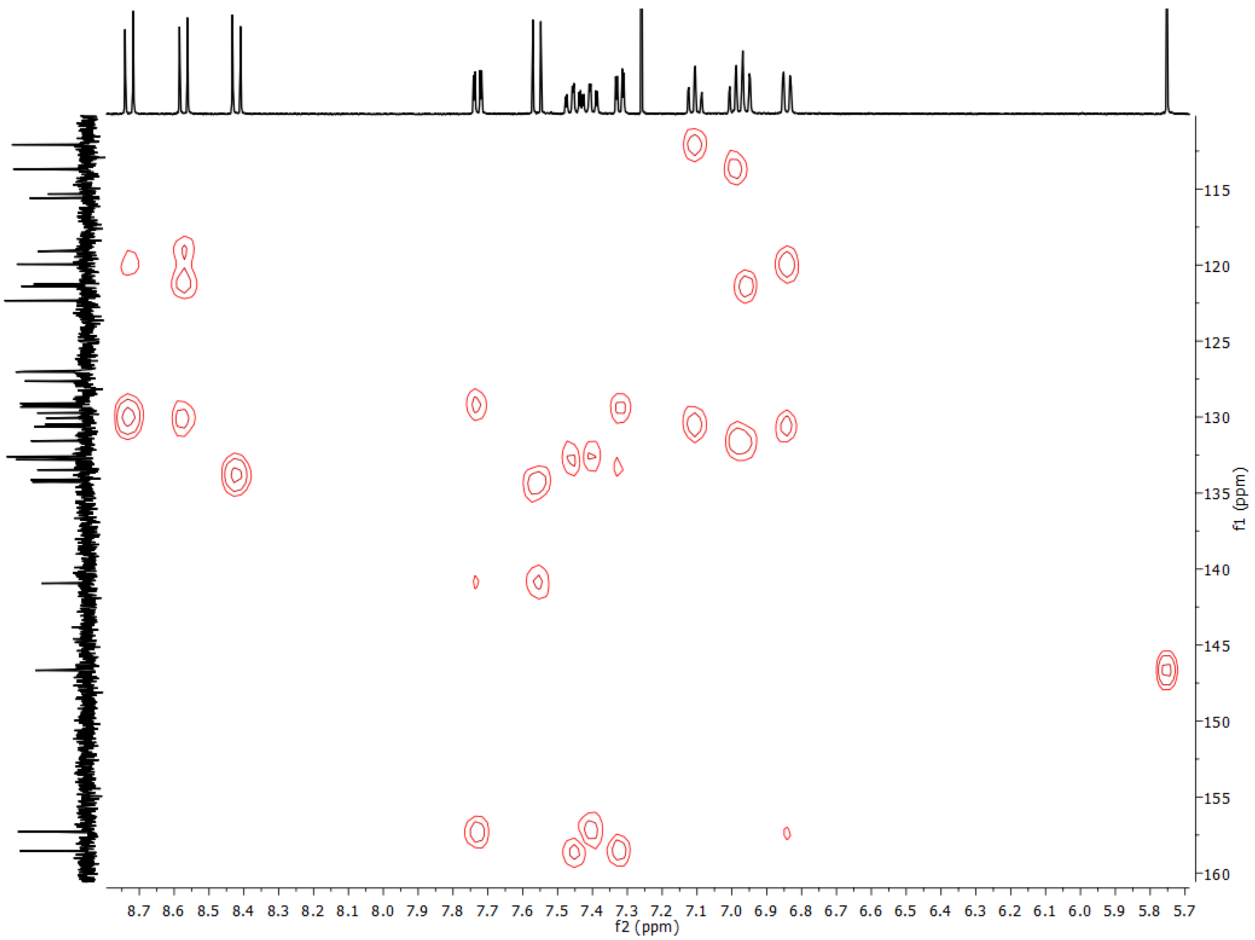

Supplementary Figure 129. HMBC NMR (400 MHz) of ***P*-10-C6**in $CDCl_3$, measured at 298 K (expansion in aromatic region).

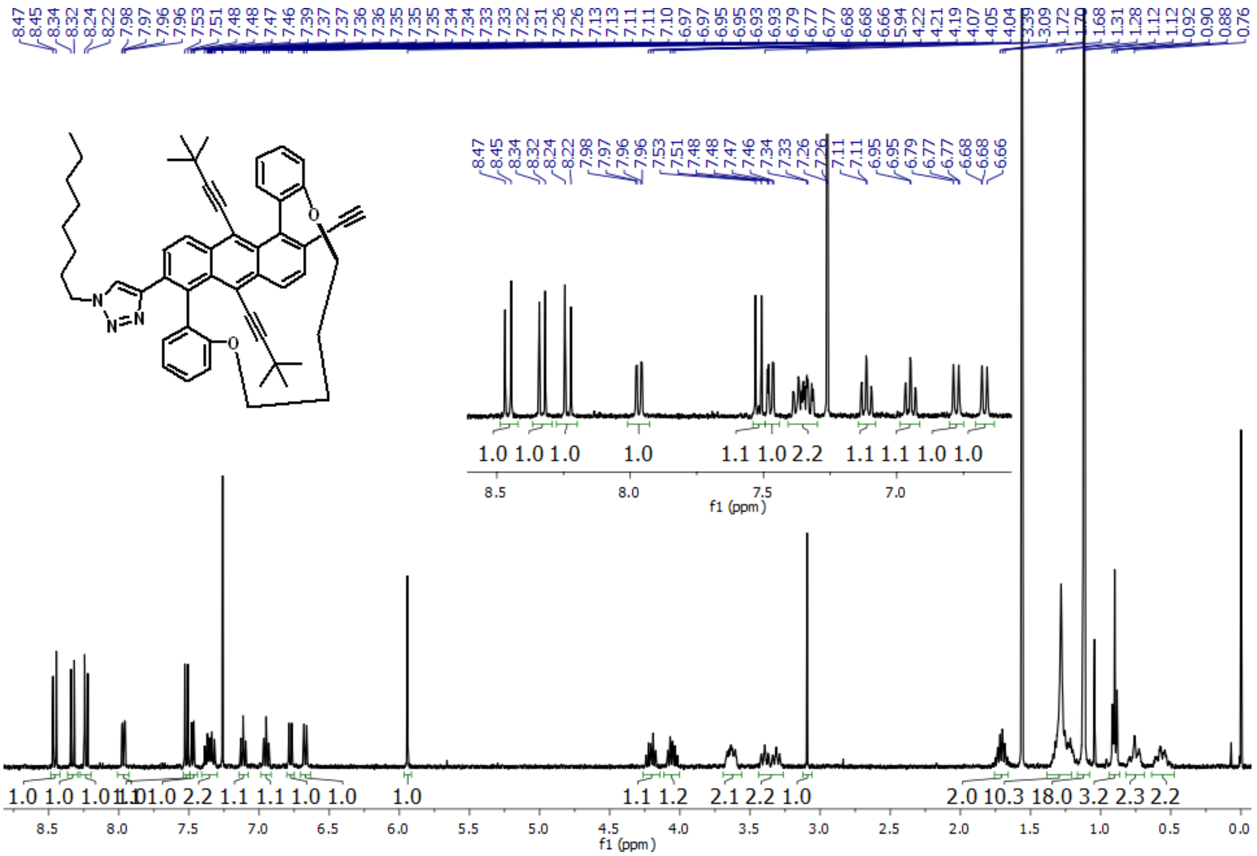

Supplementary Figure 130. ^{1}H NMR (400 MHz) of ***P*-10-C4** in $CDCl_3$, measured at 298 K.

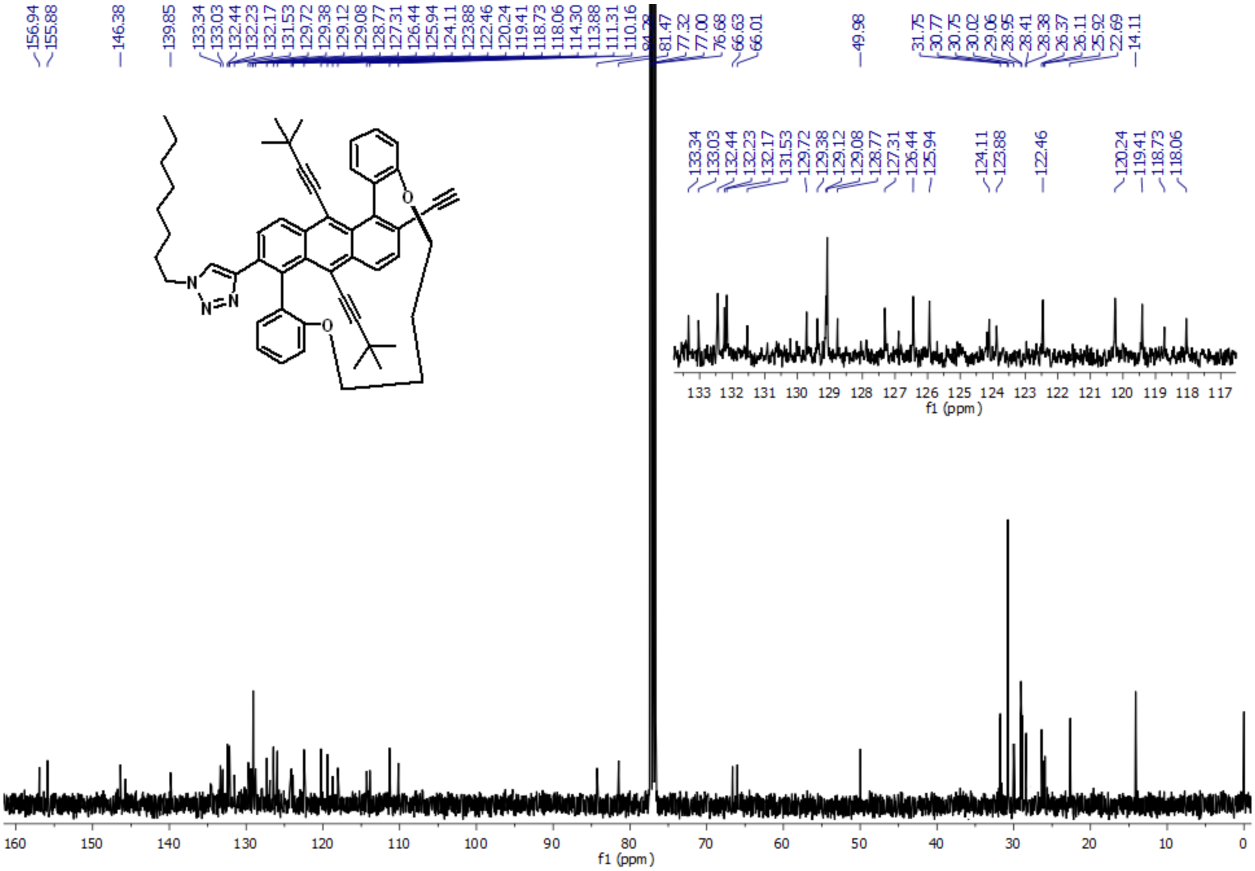

Supplementary Figure 131. ^{13}C NMR (101 MHz) of ***P*-10-C4** in $CDCl_3$, measured at 298 K.

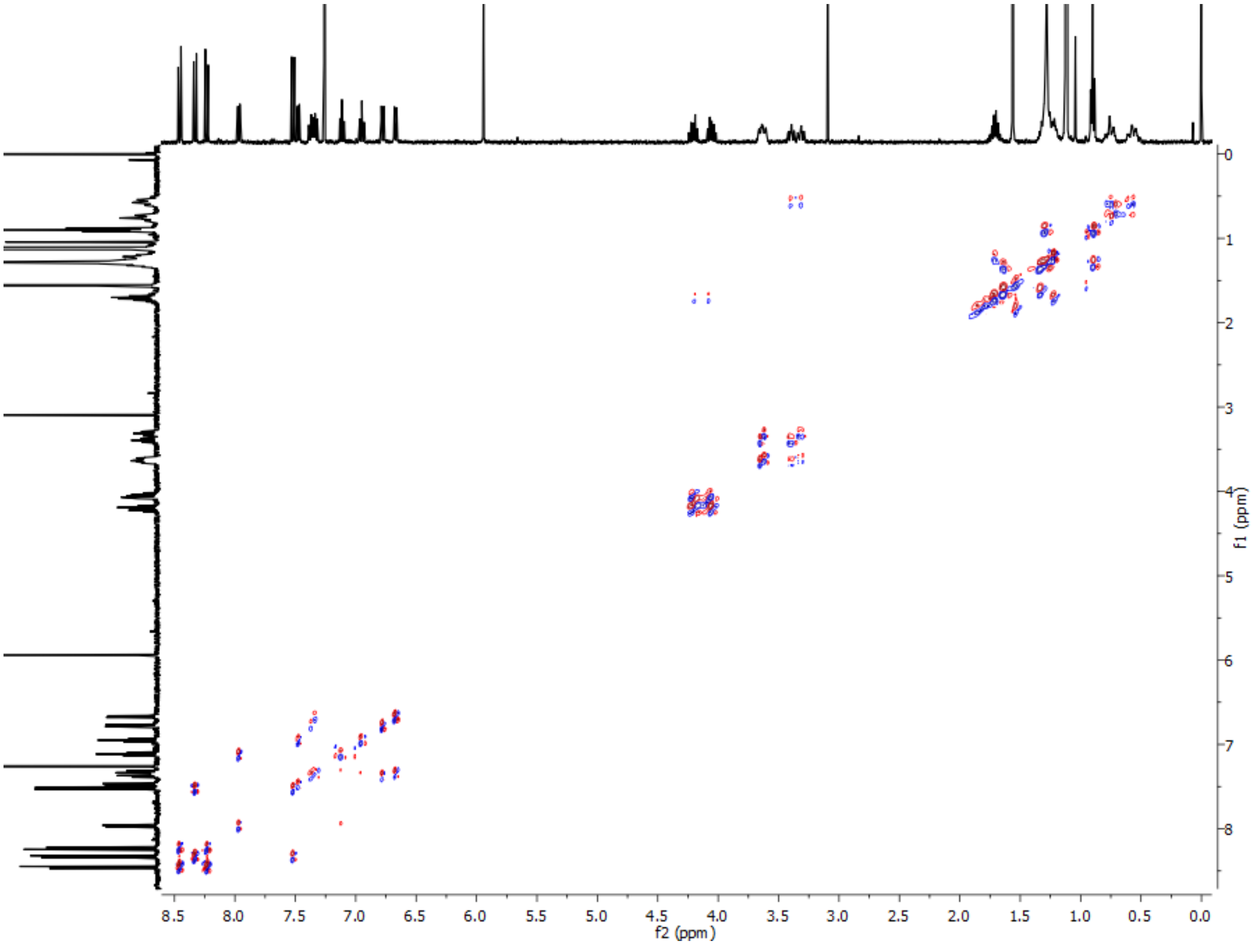

Supplementary Figure 132. COSY NMR (400 MHz) of ***P*-10-C4** in $CDCl_3$, measured at 298 K.

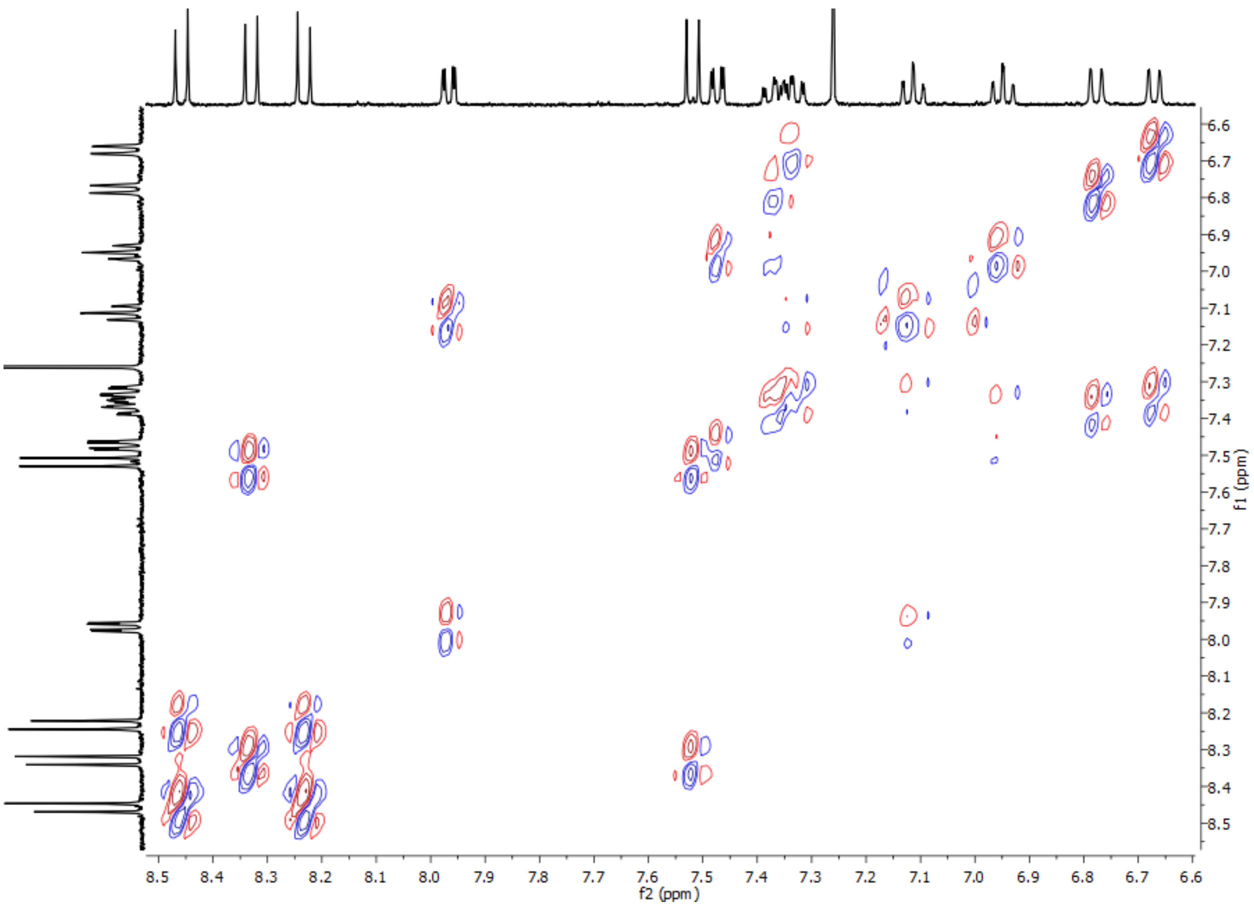

Supplementary Figure 133. COSY NMR (400 MHz) of *P*-**10-C4** in $CDCl_3$, measured at 298 K (expansion in aromatic region).

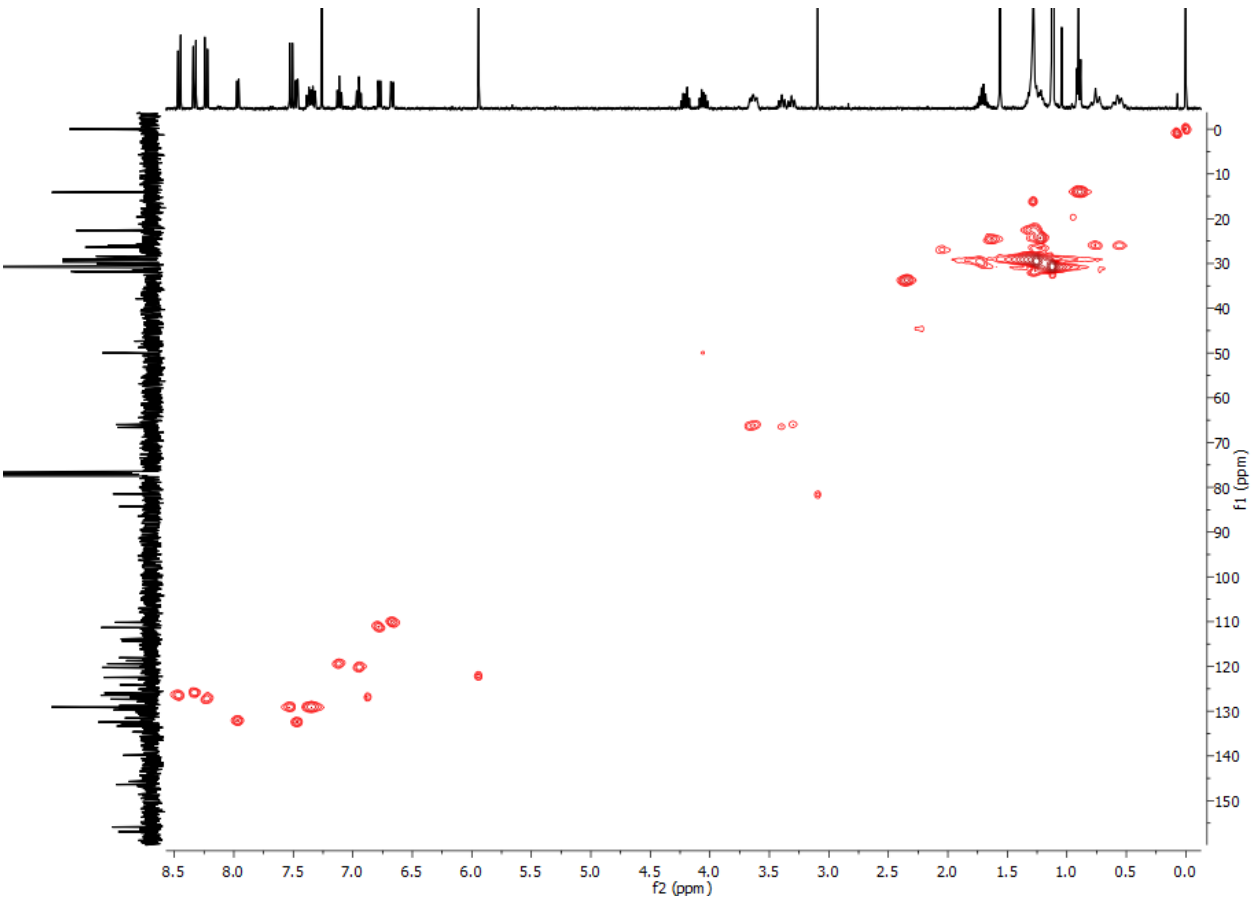

Supplementary Figure 134. HSQC NMR (400 MHz) of ***P*-10-C4** in $CDCl_3$, measured at 298 K.

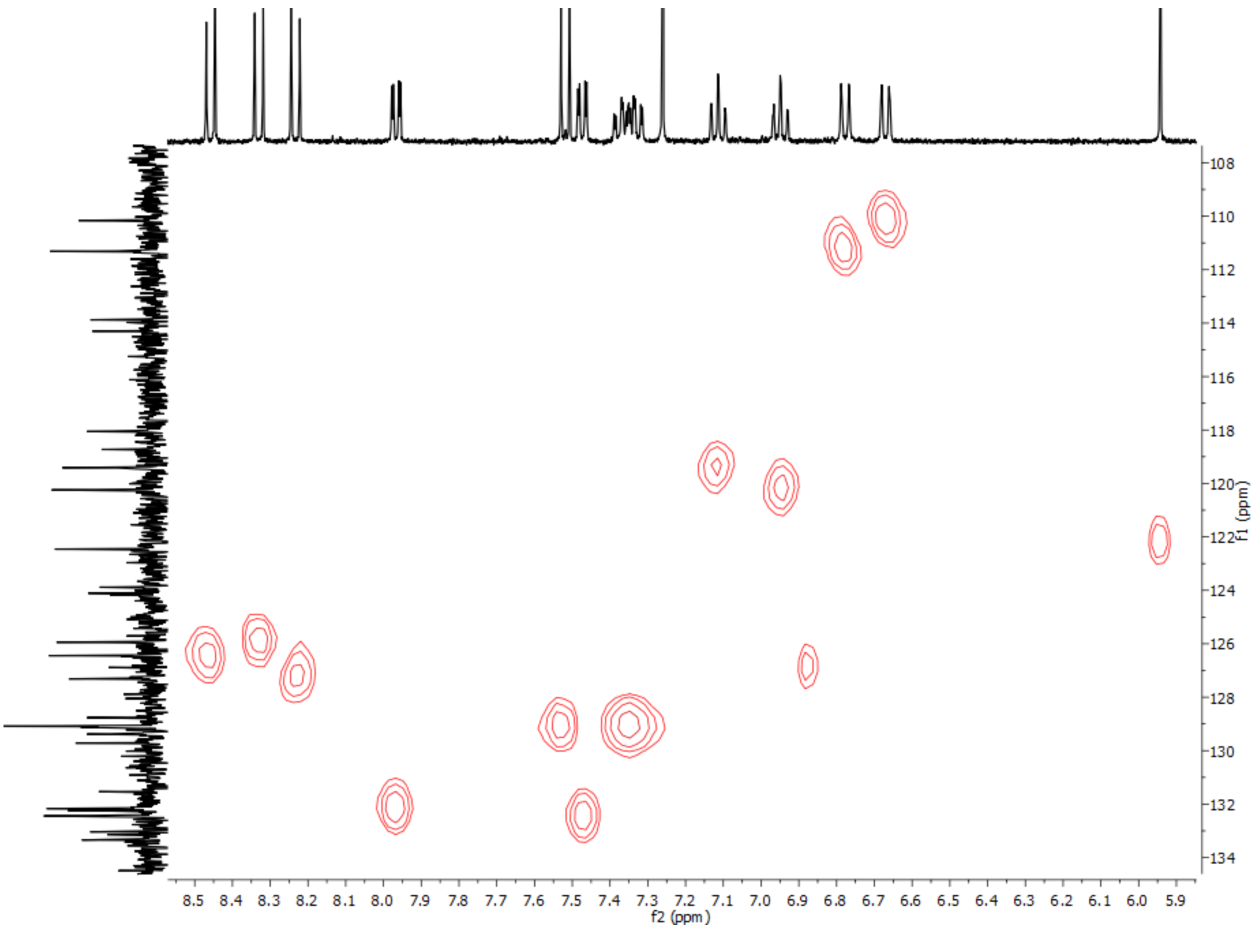

Supplementary Figure 135. HSQC NMR (400 MHz) of ***P*-10-C4** in $CDCl_3$, measured at 298 K (expansion in aromatic region).

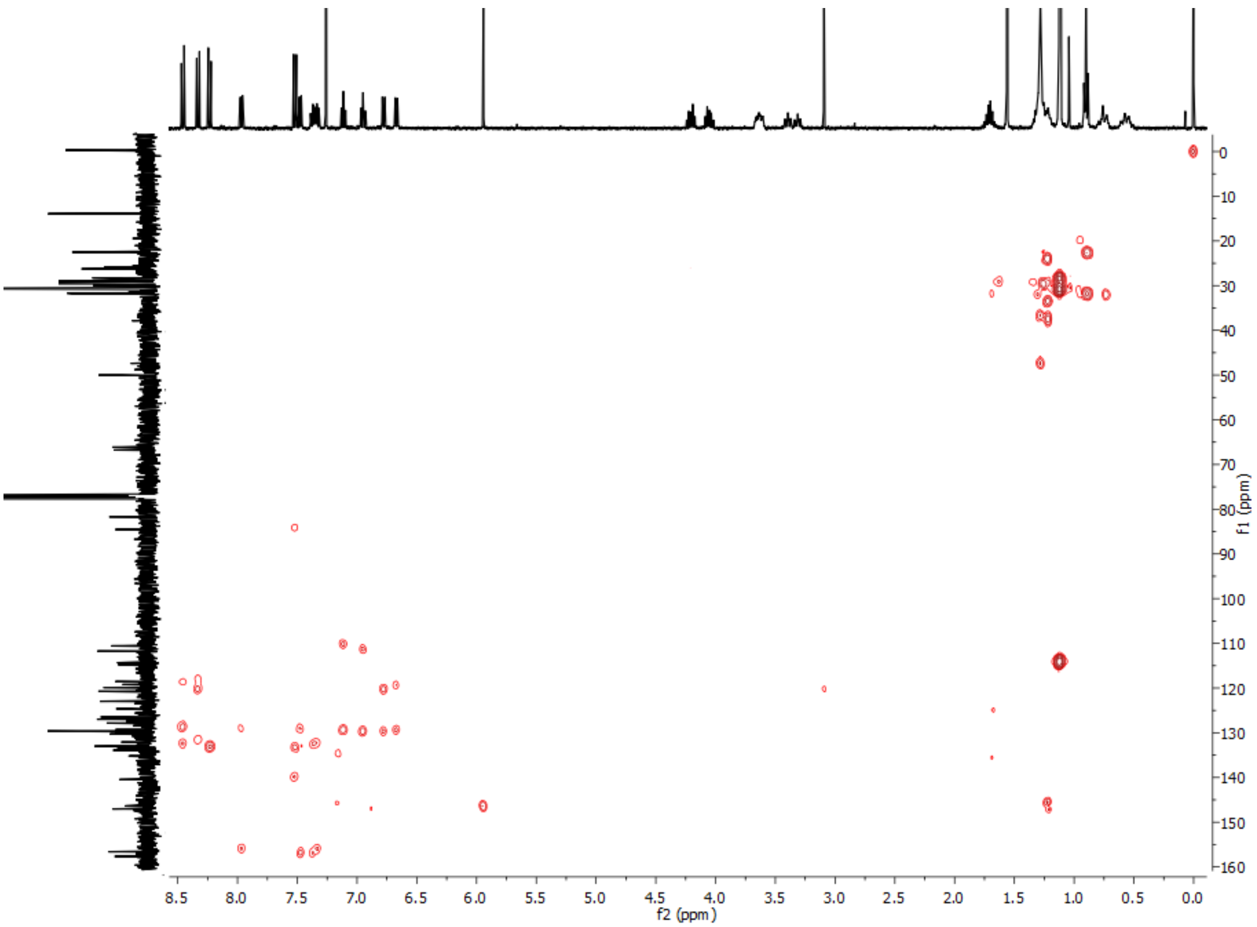

Figure 136. HMBC NMR (400 MHz) of *P*-**10-C4** in $CDCl_3$, measured at 298 K.

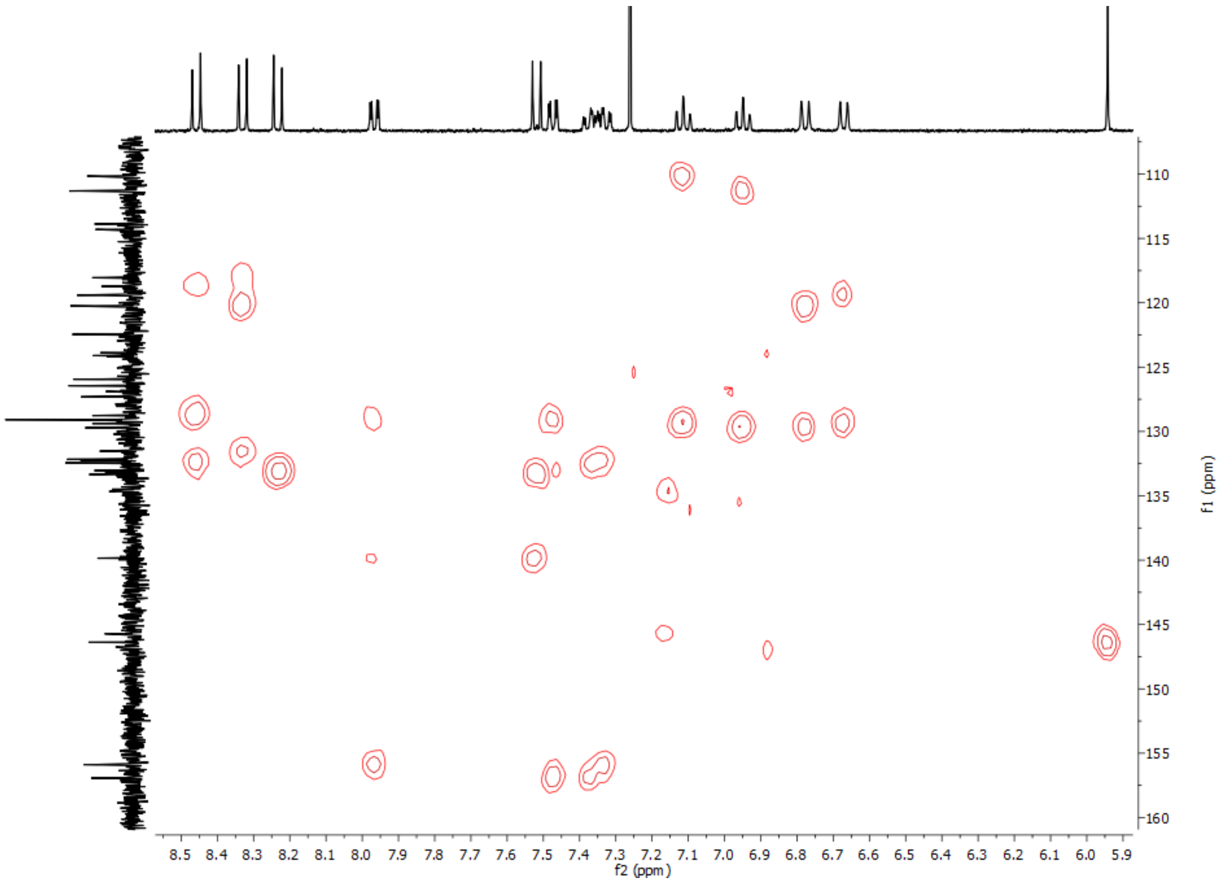

Supplementary Figure 137. HMBC NMR (400 MHz) of ***P*-10-C4** in $CDCl_3$, measured at 298 K (expansion in aromatic region).

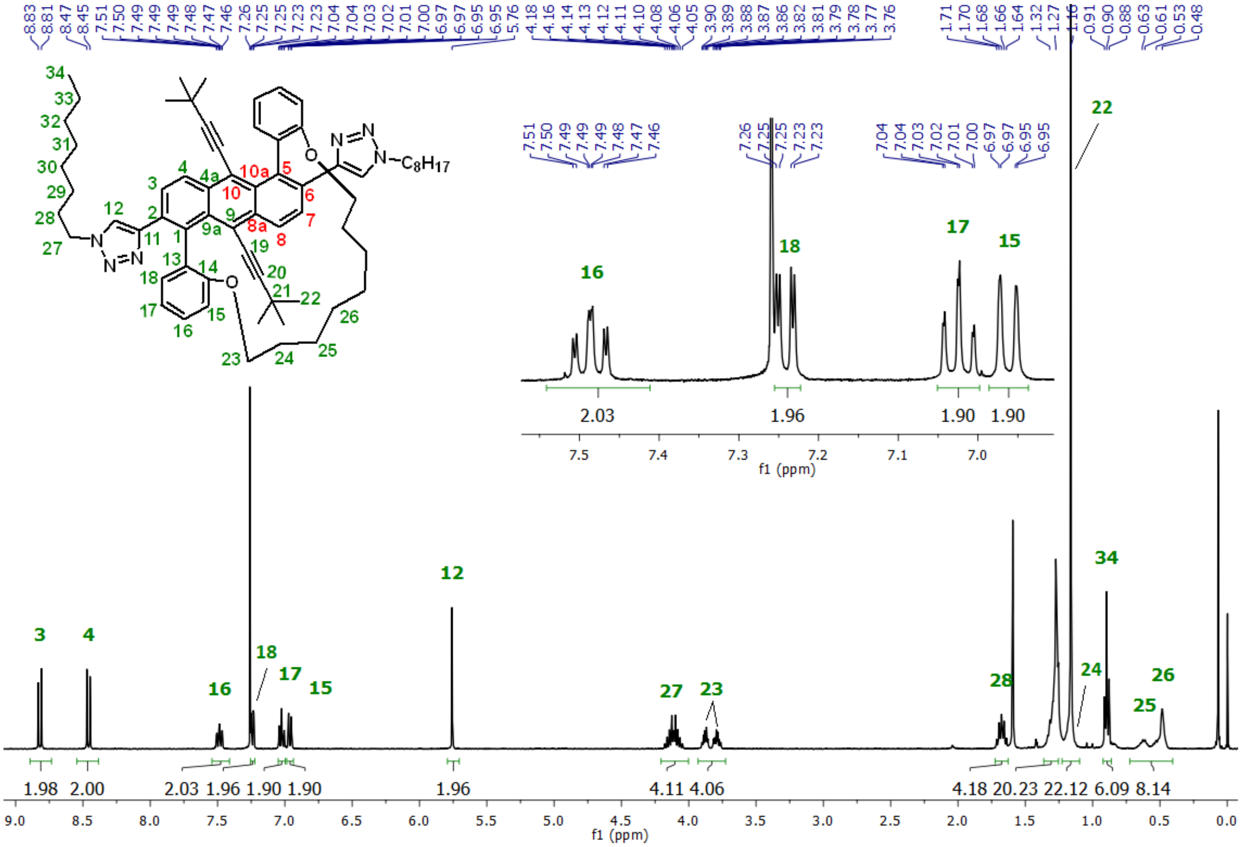

Supplementary Figure 138. ^{1}H NMR (400 MHz) of *P*-**1-Ant-C8** in $CDCl_3$, measured at 298 K.

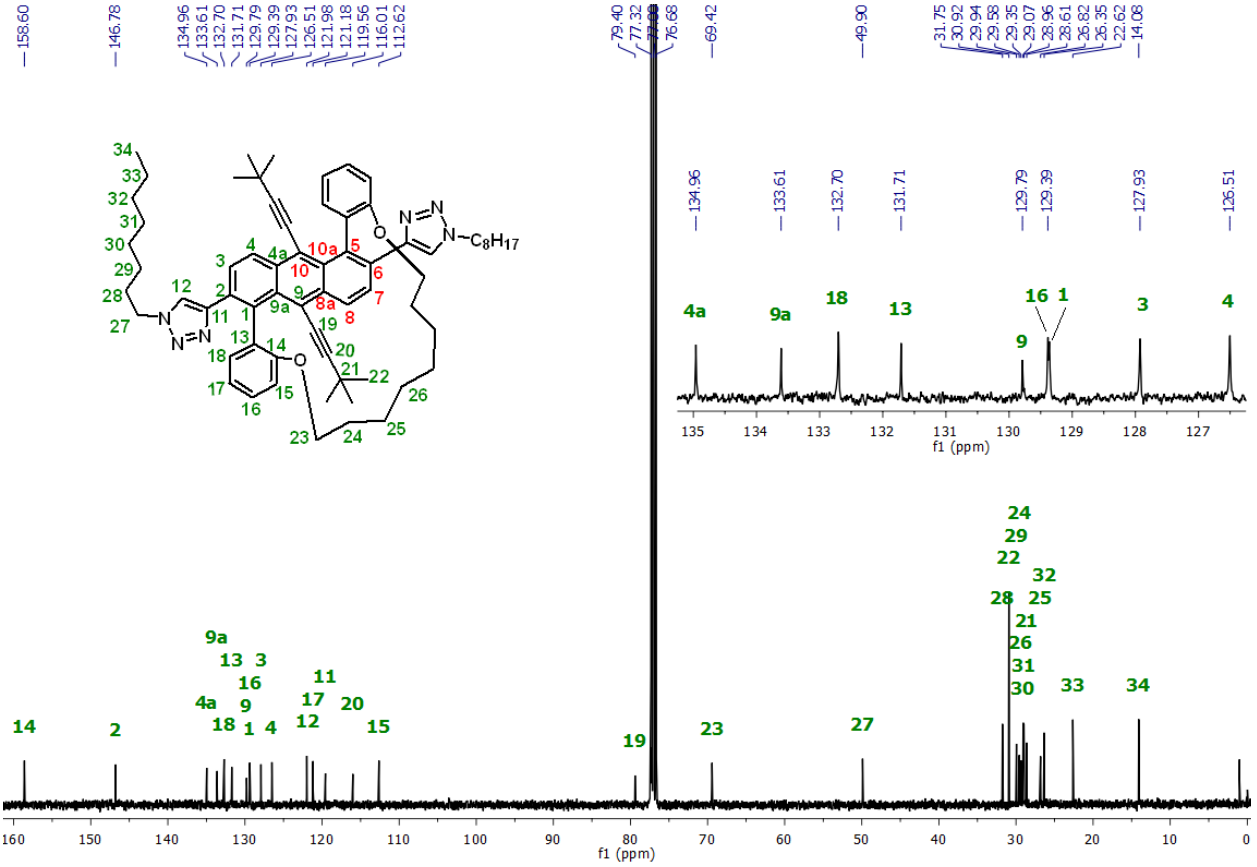

Supplementary Figure 139. ^{13}C NMR (101 MHz) of *P*-**1-Ant-C8** in $CDCl_3$, measured at 298 K.

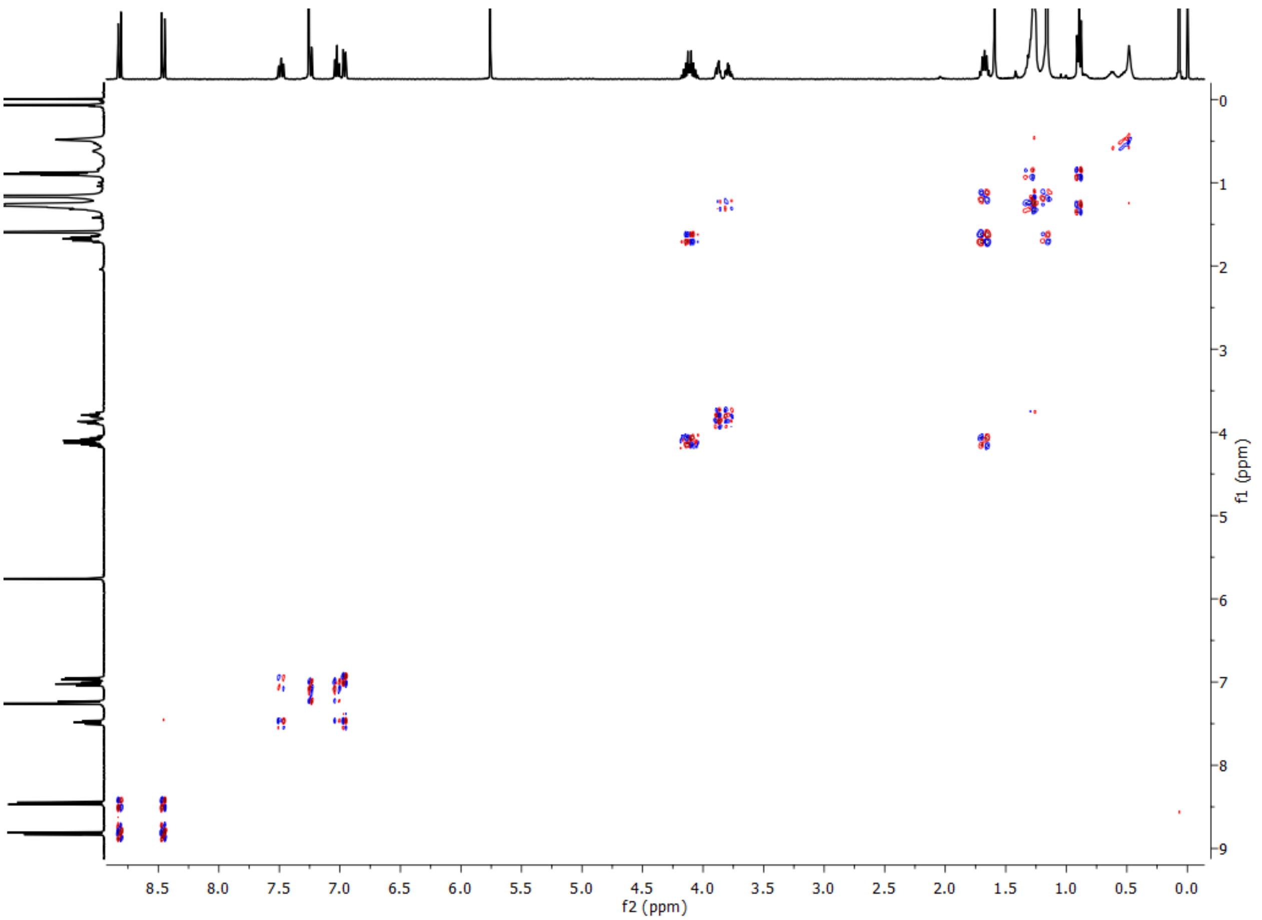

Supplementary Figure 140. COSY NMR (400 MHz) of *P*-**1-Ant-C8** in $CDCl_3$, measured at 298 K.

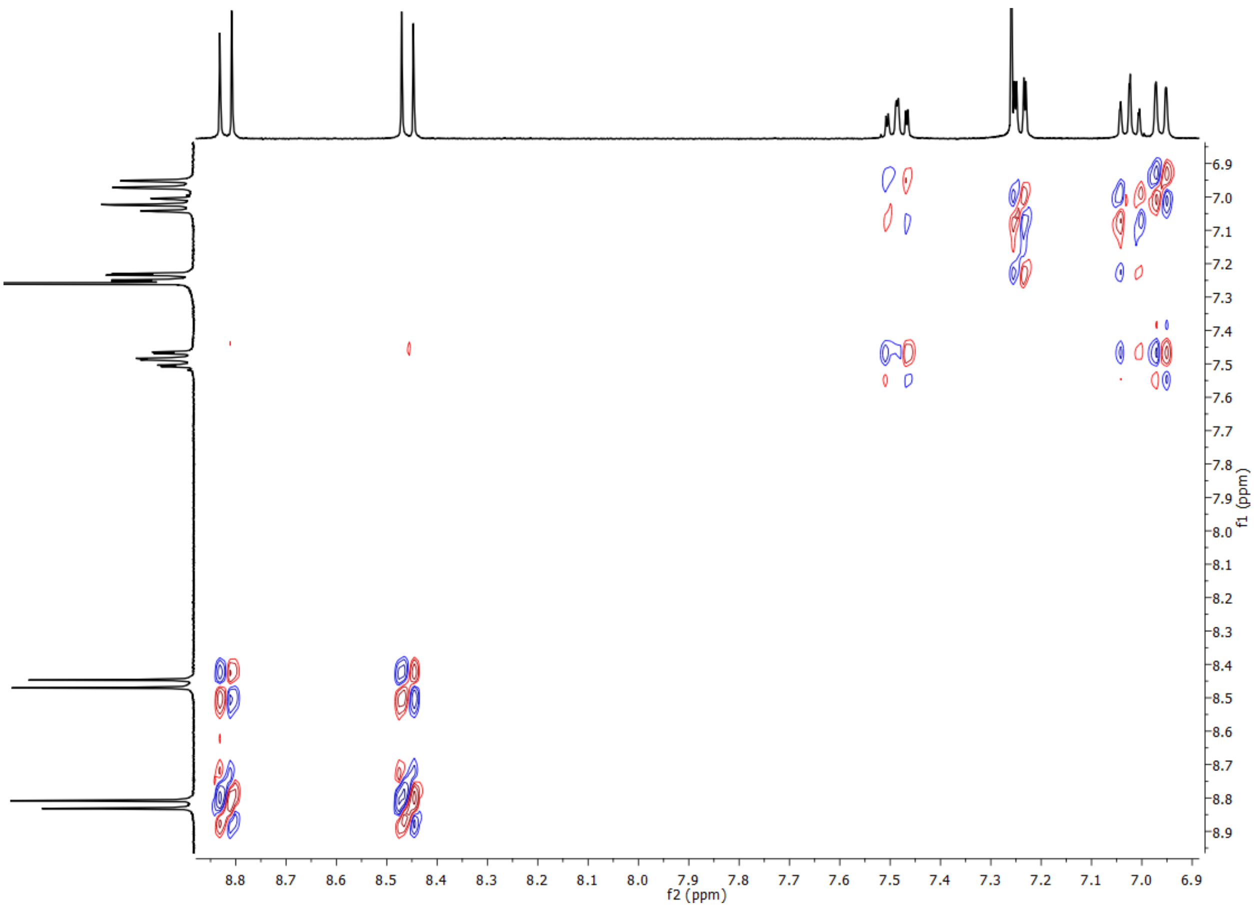

Supplementary Figure 141. COSY NMR (400 MHz) of *P*-**1-Ant-C8** in $CDCl_3$, measured at 298 K (expansion in aromatic region).

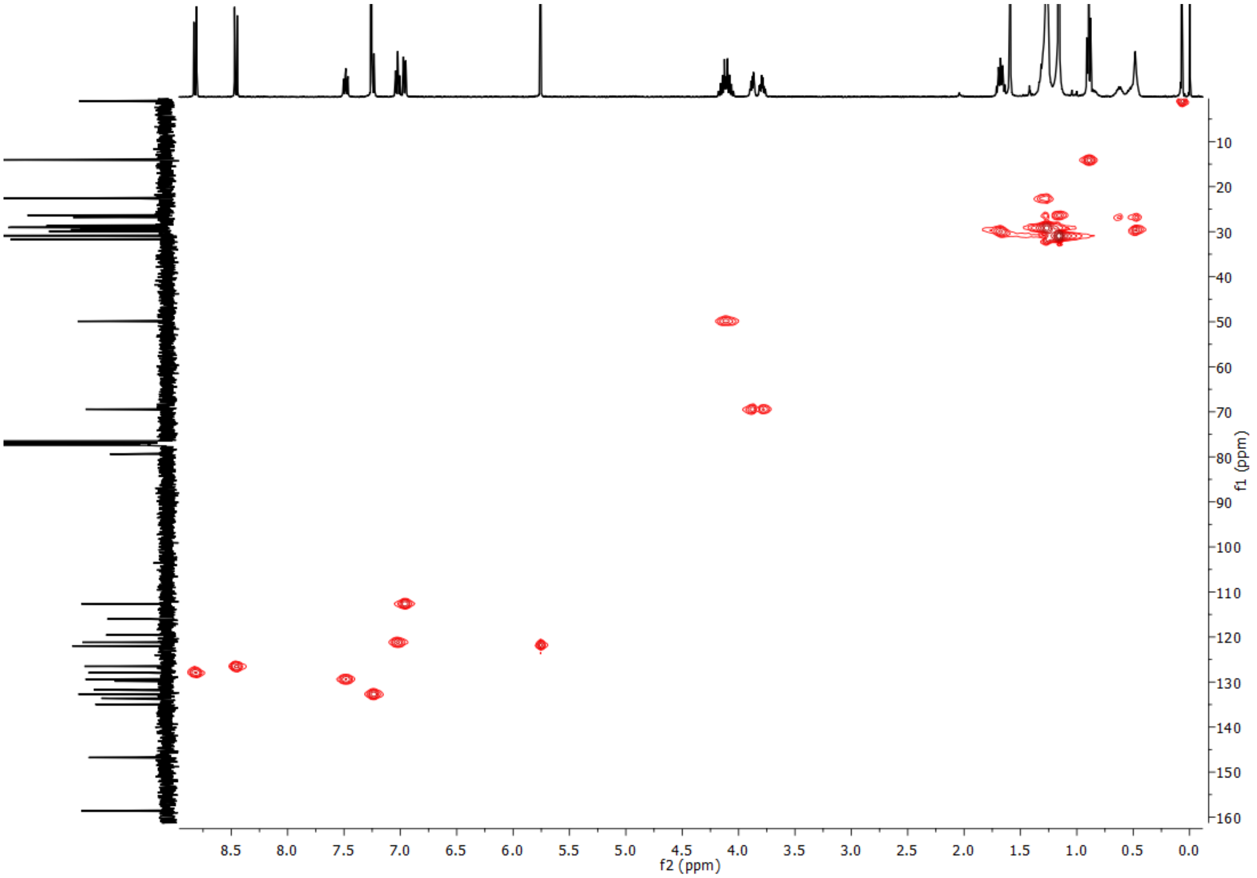

Supplementary Figure 142. COSY NMR (top) and HSQC NMR (bottom, 400 MHz) of ***P*-1-Ant-C8** in $CDCl_3$, measured at 298 K.

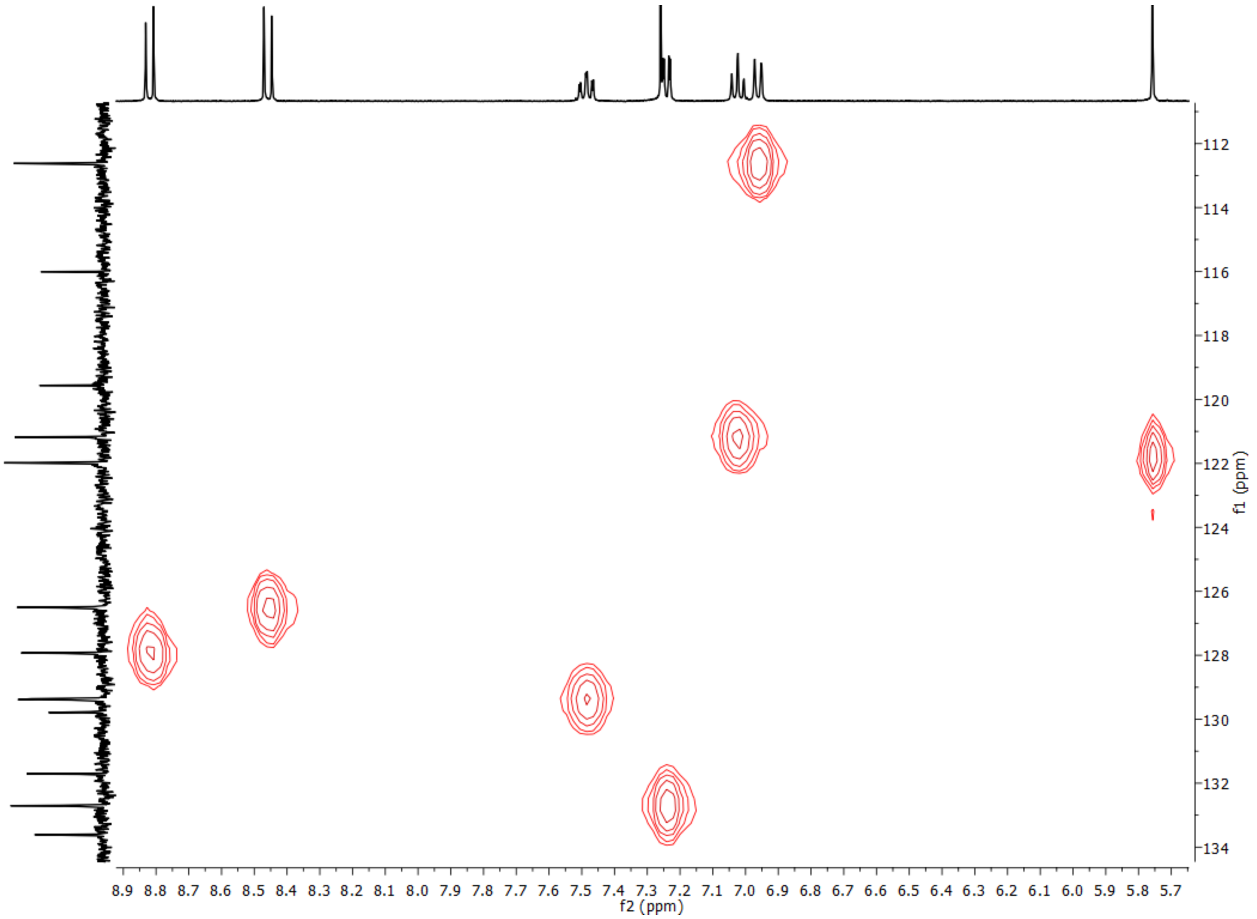

Supplementary Figure 143. HSQC NMR (400 MHz) of *P*-**1-Ant-C8** in $CDCl_3$, measured at 298 K (expansion in aromatic region).

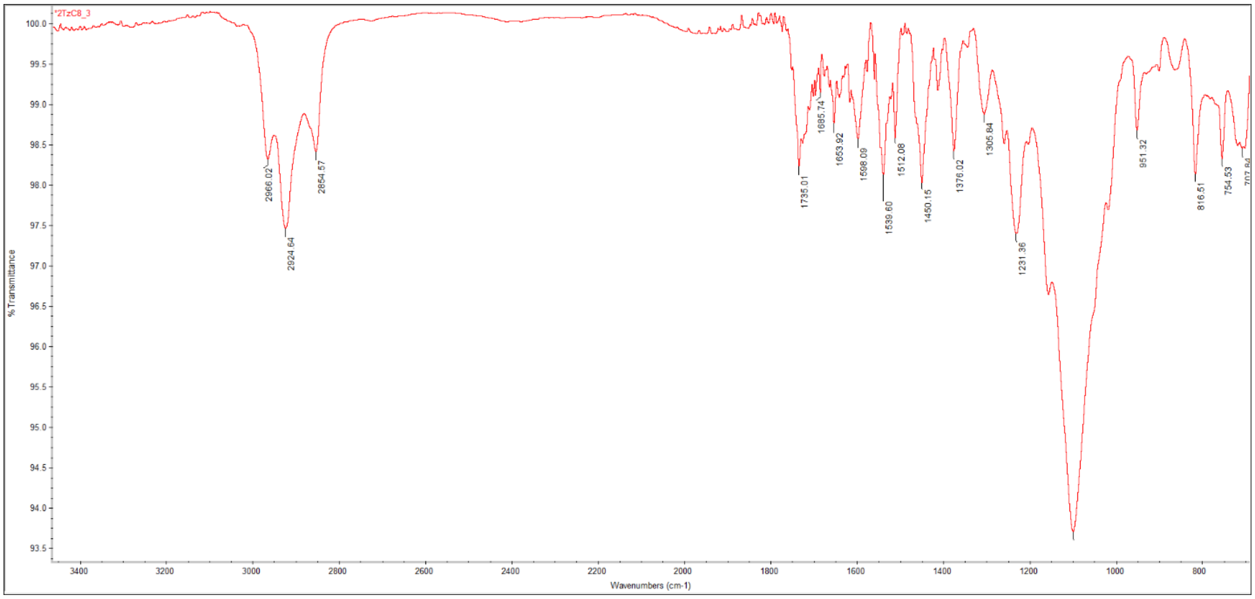

Supplementary Figure 144. ATR-FTIR of *P*-**1-Ant-C8.**

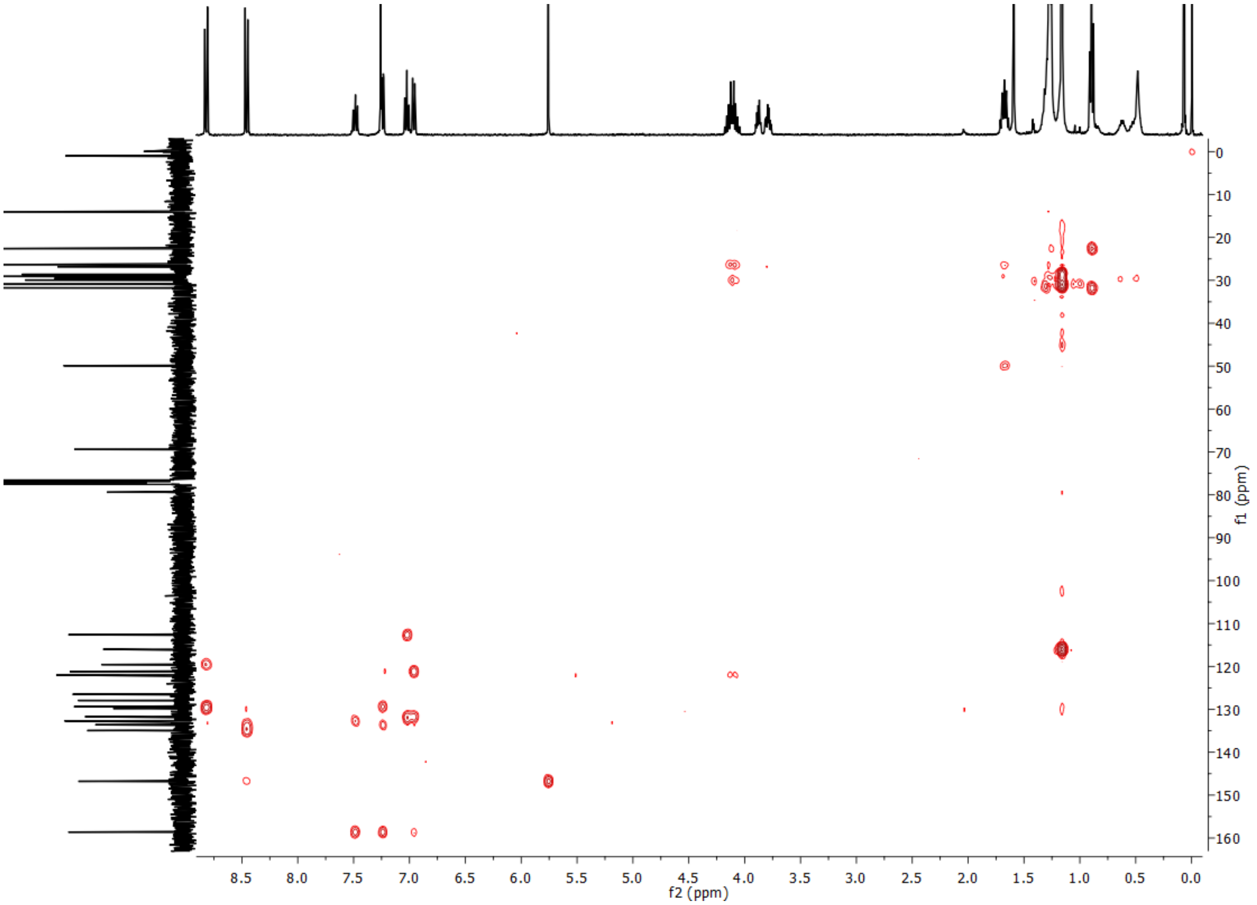

Supplementary Figure 145. HMBC NMR (400 MHz) of *P*-**1-Ant-C8** in $CDCl_3$, measured at 298 K.

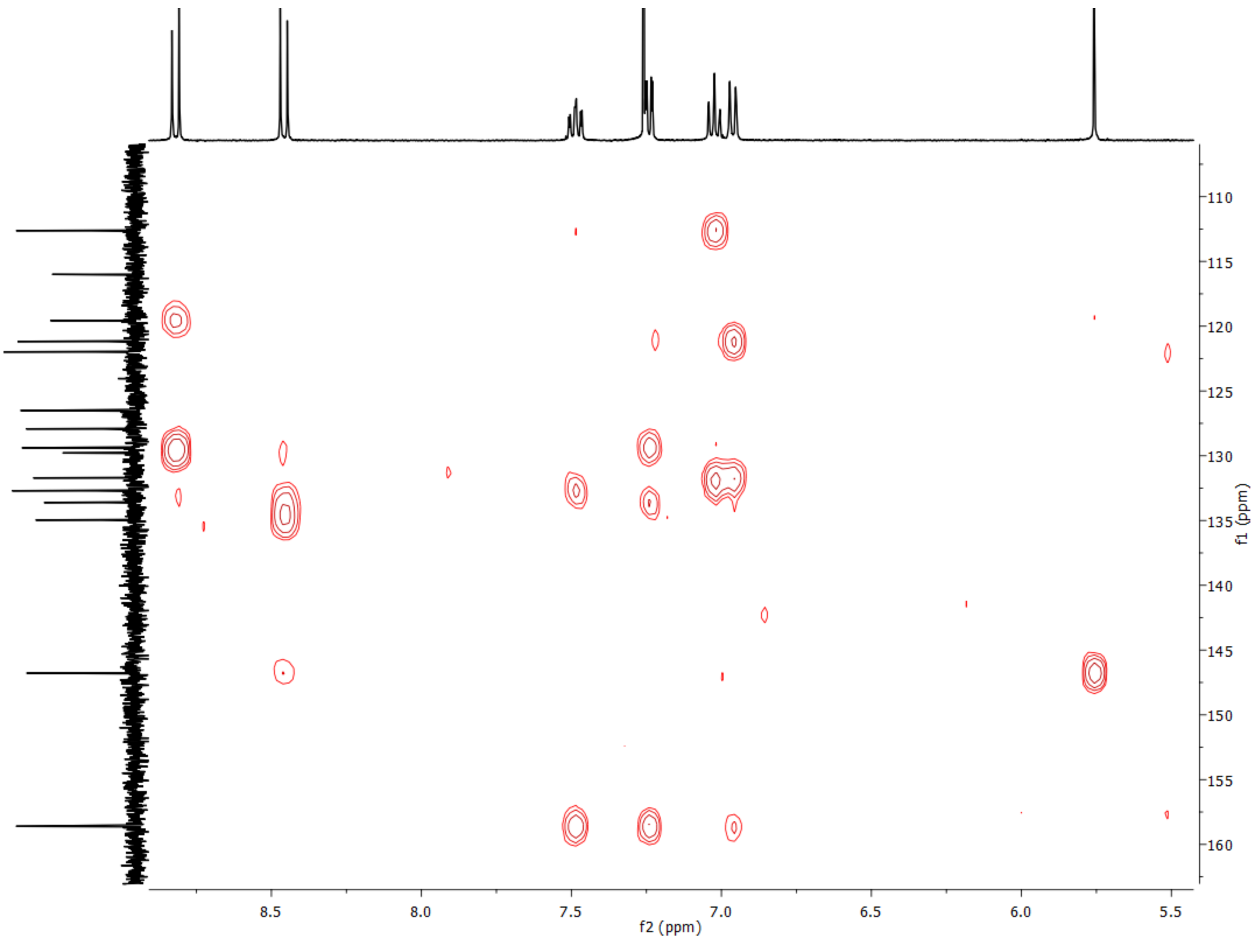

Supplementary Figure 146. HMBC NMR (400 MHz) of ***P*-1-Ant-C8** in $CDCl_3$, measured at 298 K (expansion in aromatic region).

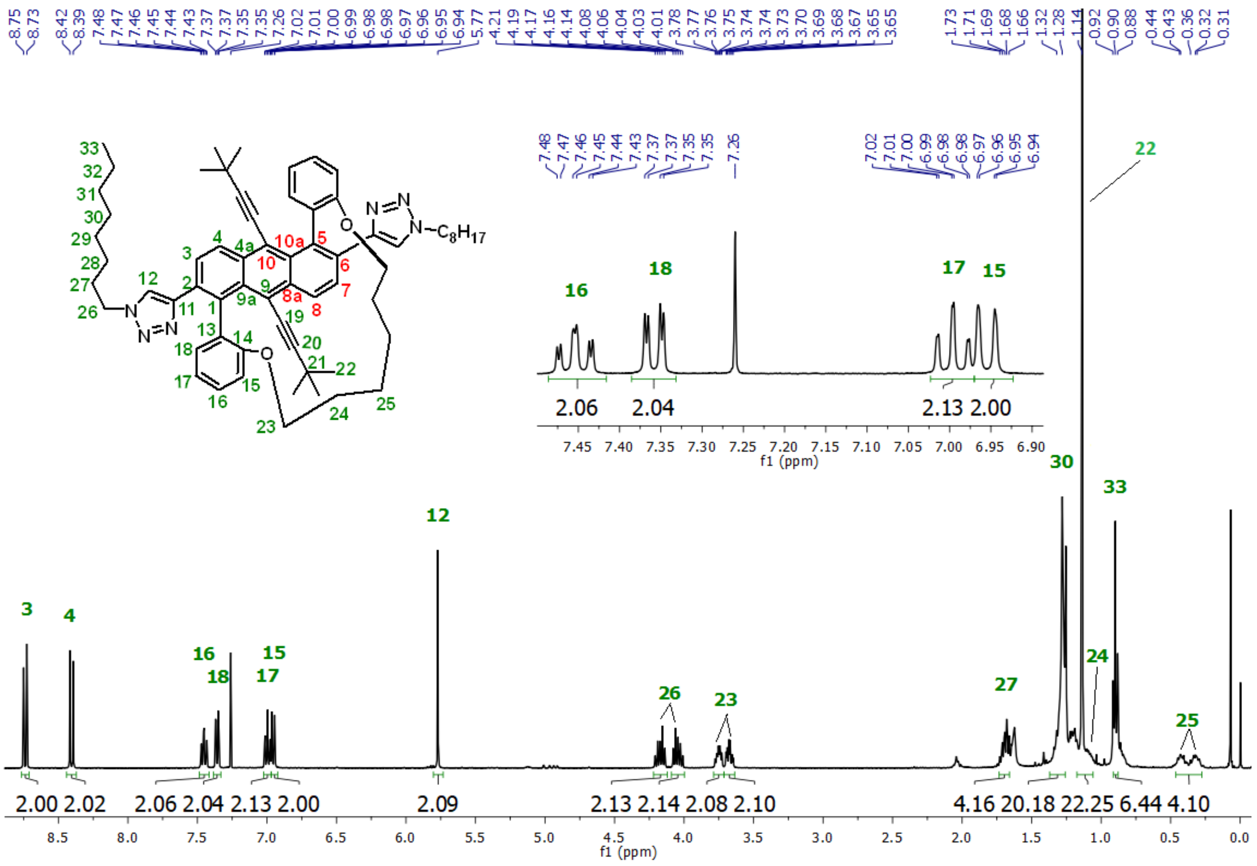

Supplementary Figure 147. ^{1}H NMR (400 MHz) of *P*-**1-Ant-C6** in $CDCl_3$, measured at 298 K.

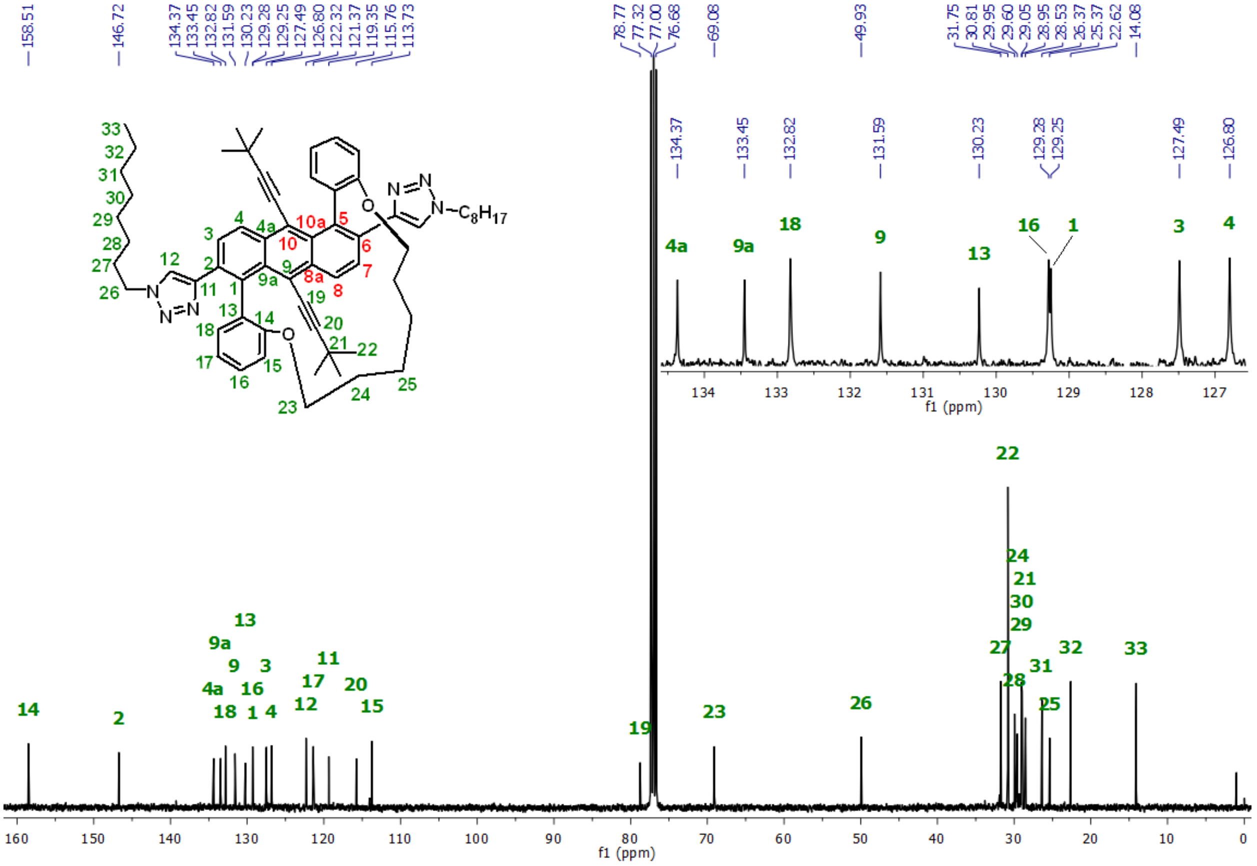

Supplementary Figure 148. ^{13}C NMR (101 MHz) of *P*-**1-Ant-C6** in $CDCl_3$, measured at 298 K.

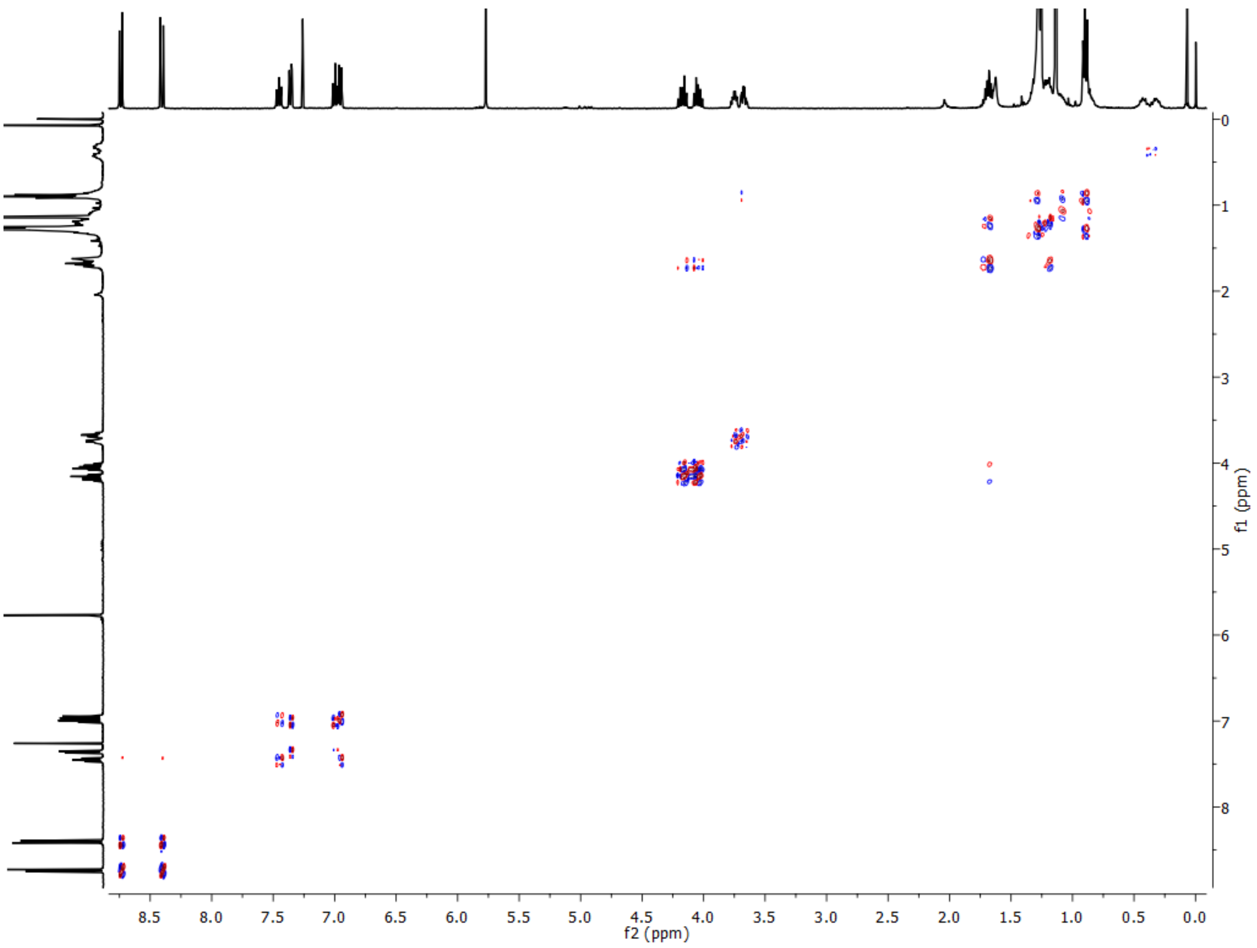

Supplementary Figure 149. COSY NMR (400 MHz) of *P*-**1-Ant-C6** in $CDCl_3$, measured at 298 K.

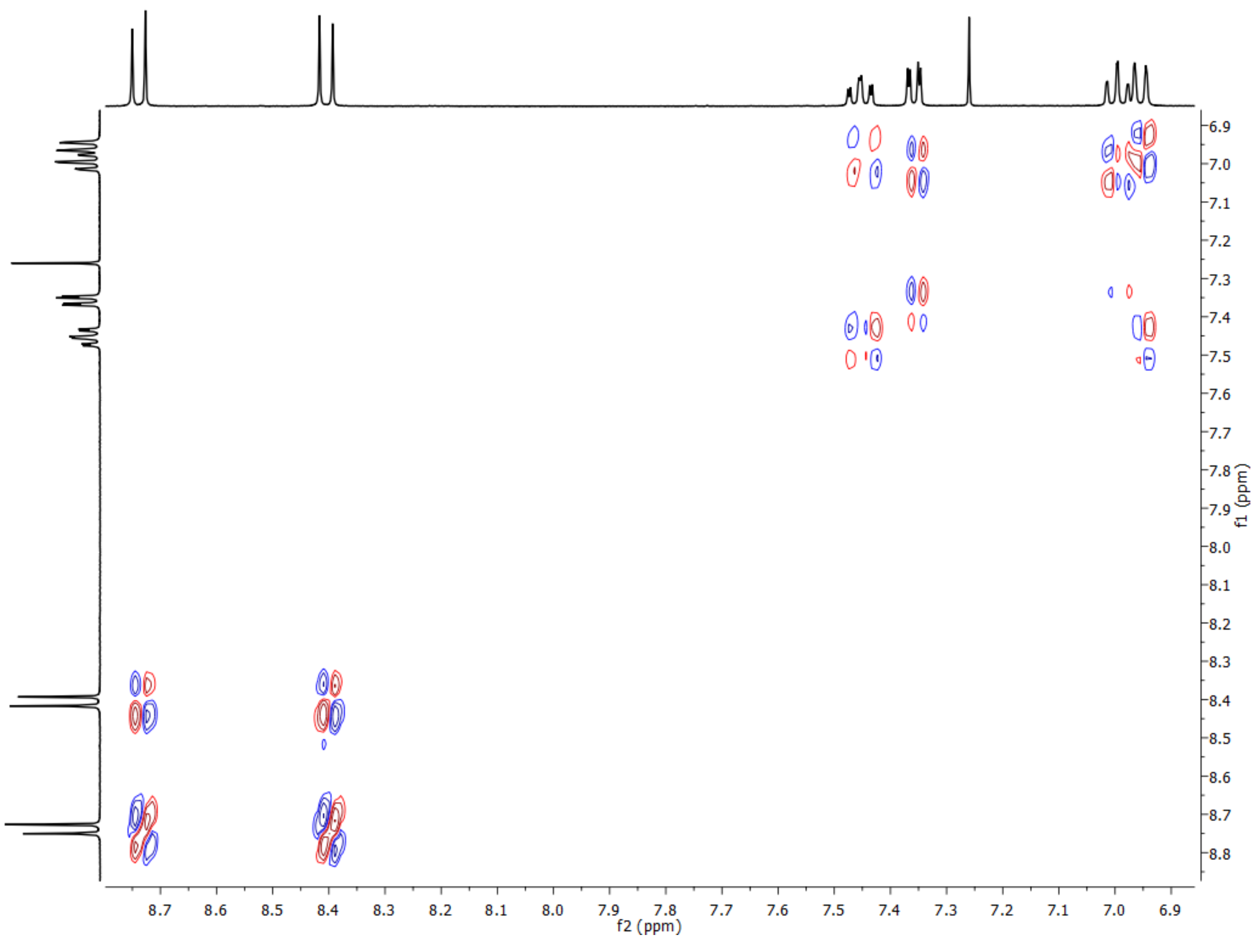

Supplementary Figure 150. COSY NMR (400 MHz) of ***P*-1-Ant-C6** in $CDCl_3$, measured at 298 K (expansion in aromatic region).

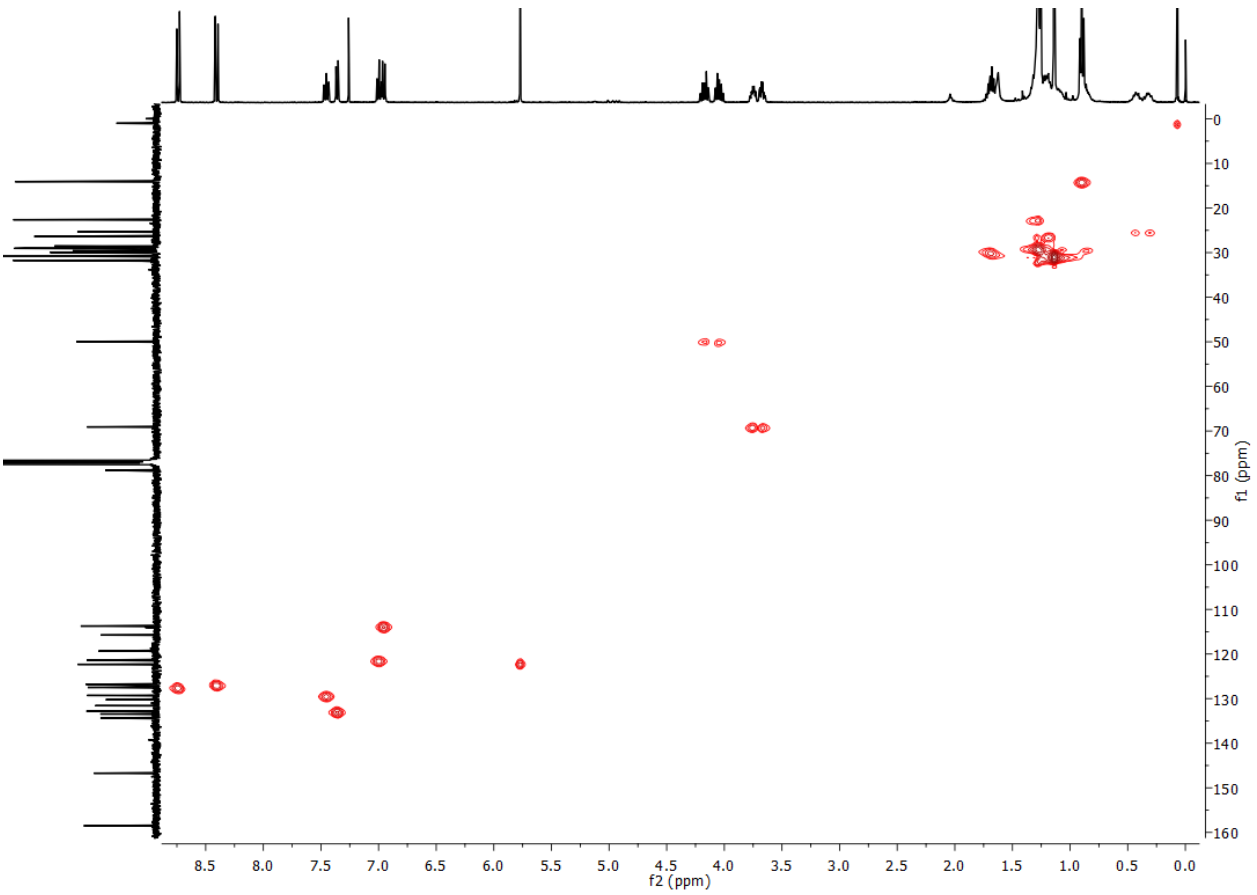

Supplementary Figure 151. HSQC NMR (400 MHz) of *P*-**1-Ant-C6** in $CDCl_3$, measured at 298 K.

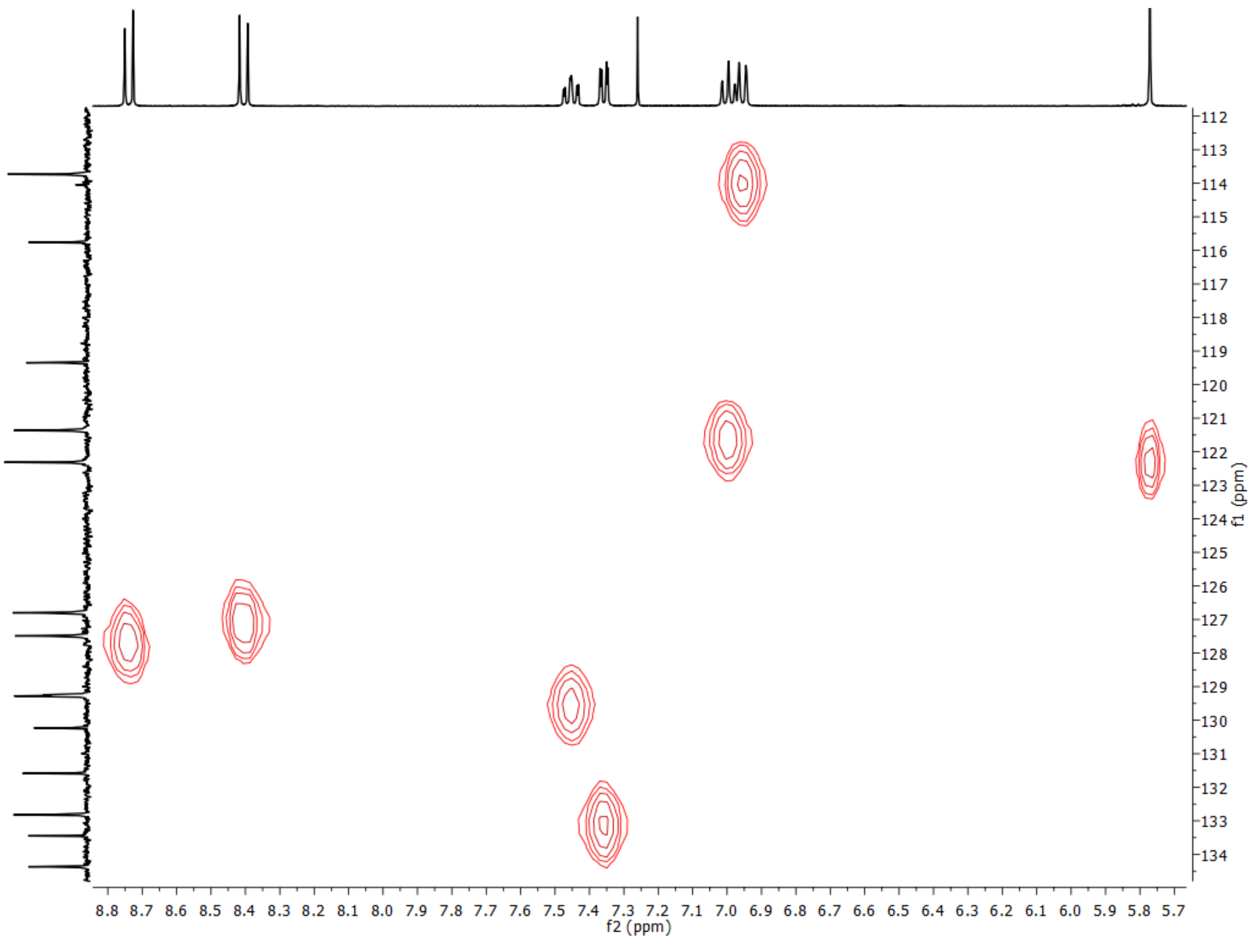

Supplementary Figure 152. HSQC NMR (400 MHz) of ***P*-1-Ant-C6** in $CDCl_3$, measured at 298 K (expansion in aromatic region).

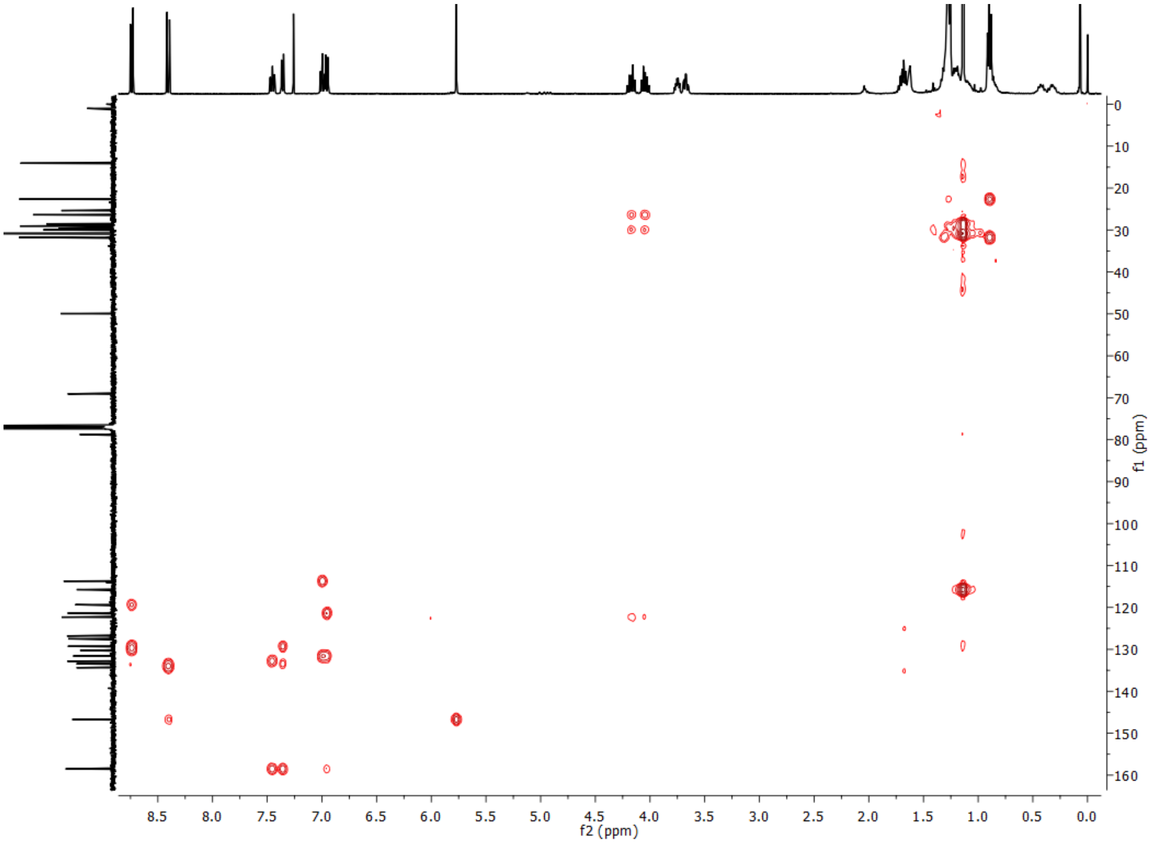

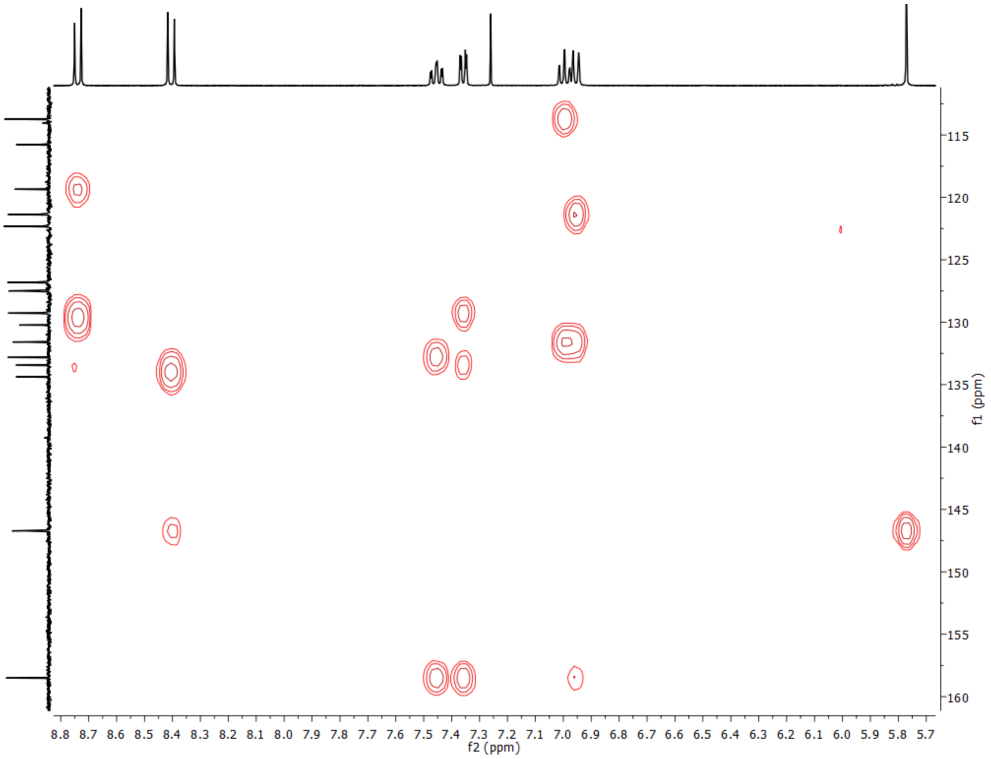

Supplementary Figure 153. HMBC NMR (400 MHz) of *P*-**1-Ant-C6** in $CDCl_3$, measured at 298 K (bottom: expansion in aromatic region).

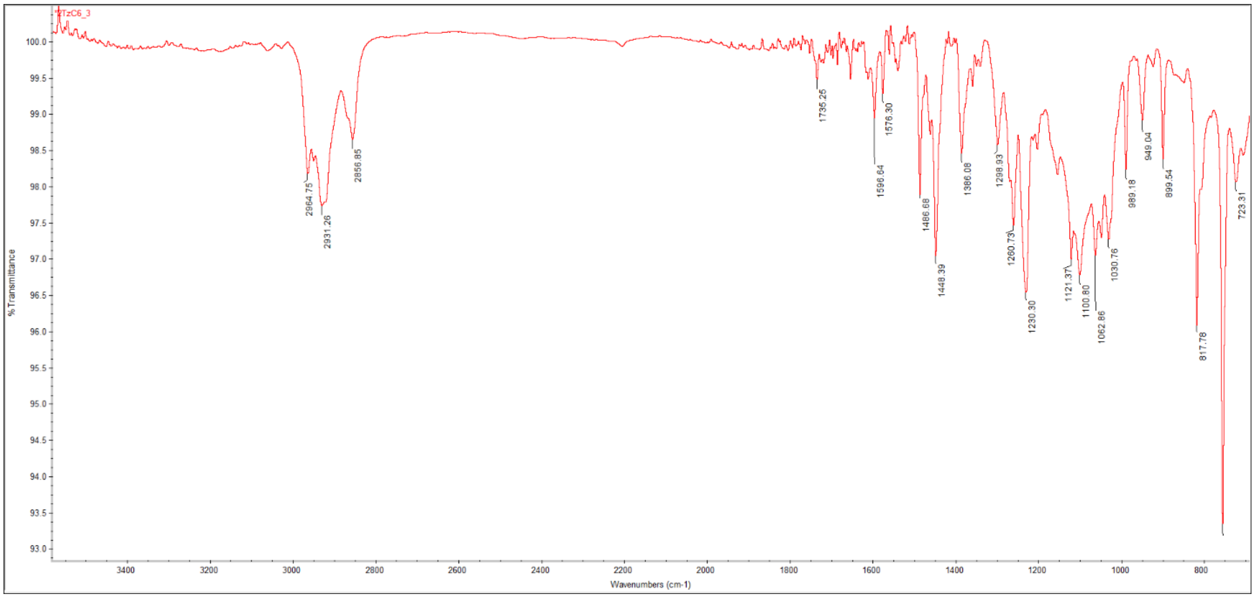

Supplementary Figure 154. ATR-FTIR of *P*-**1-Ant-C6**.

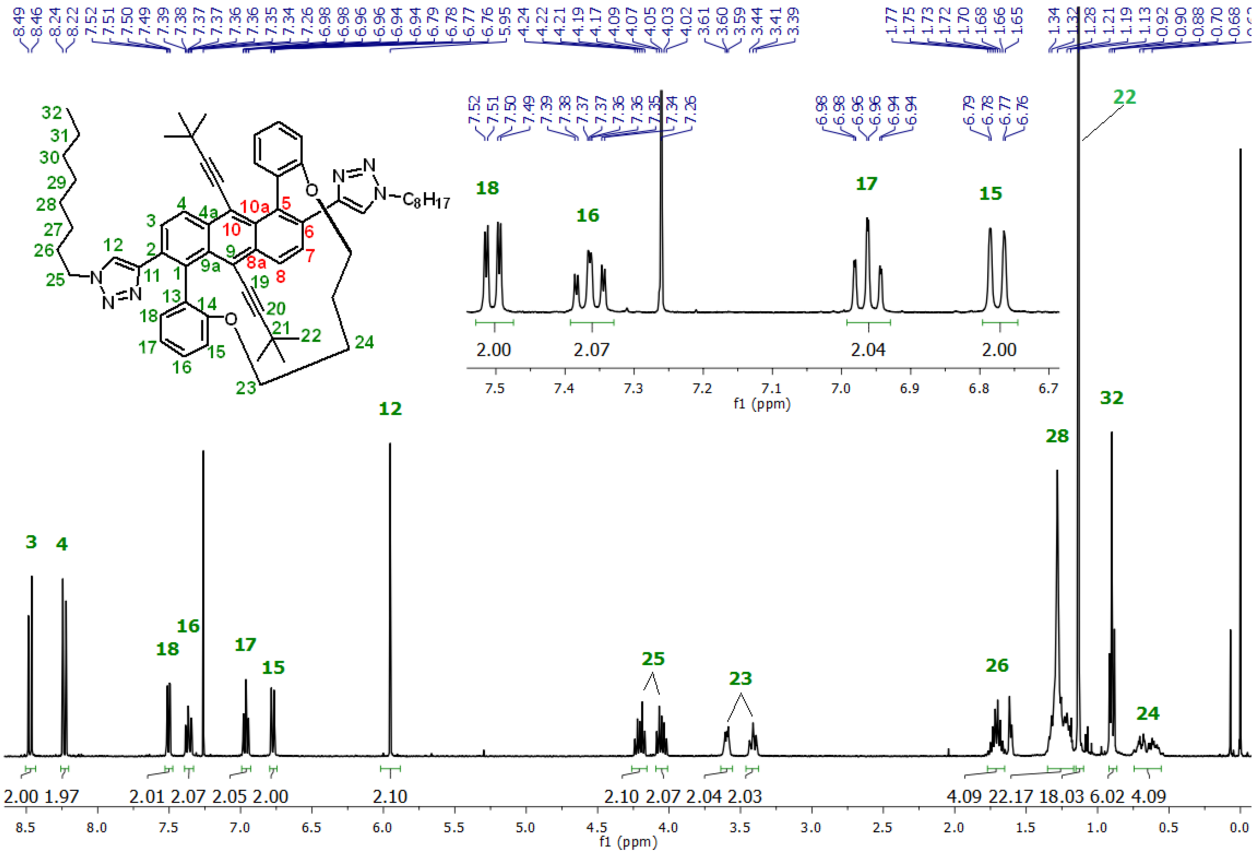

Supplementary Figure 155. ^{1}H NMR (400 MHz) of *P*-**1-Ant-C4** in $CDCl_3$, measured at 298 K.

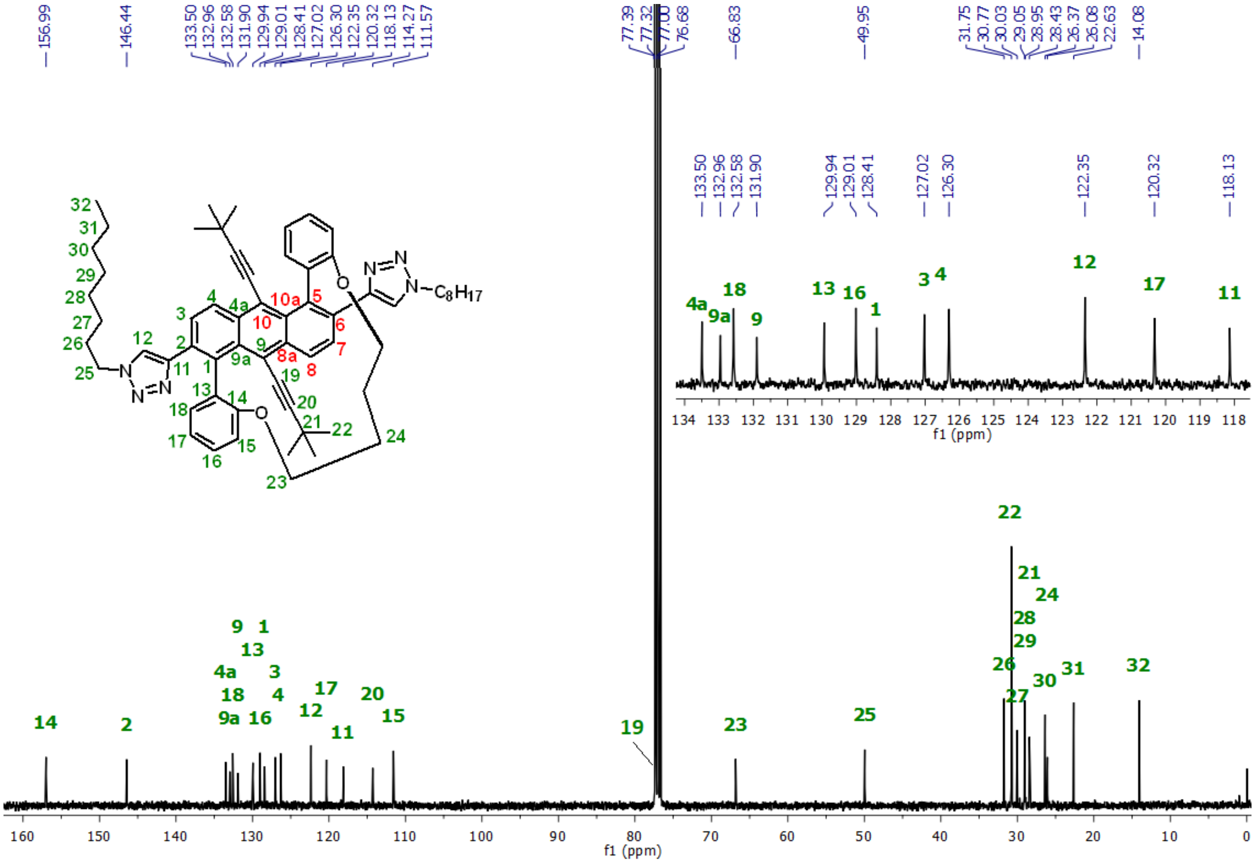

Supplementary Figure 156. ^{13}C NMR (101 MHz) of *P*-**1-Ant-C4** in $CDCl_3$, measured at 298 K.

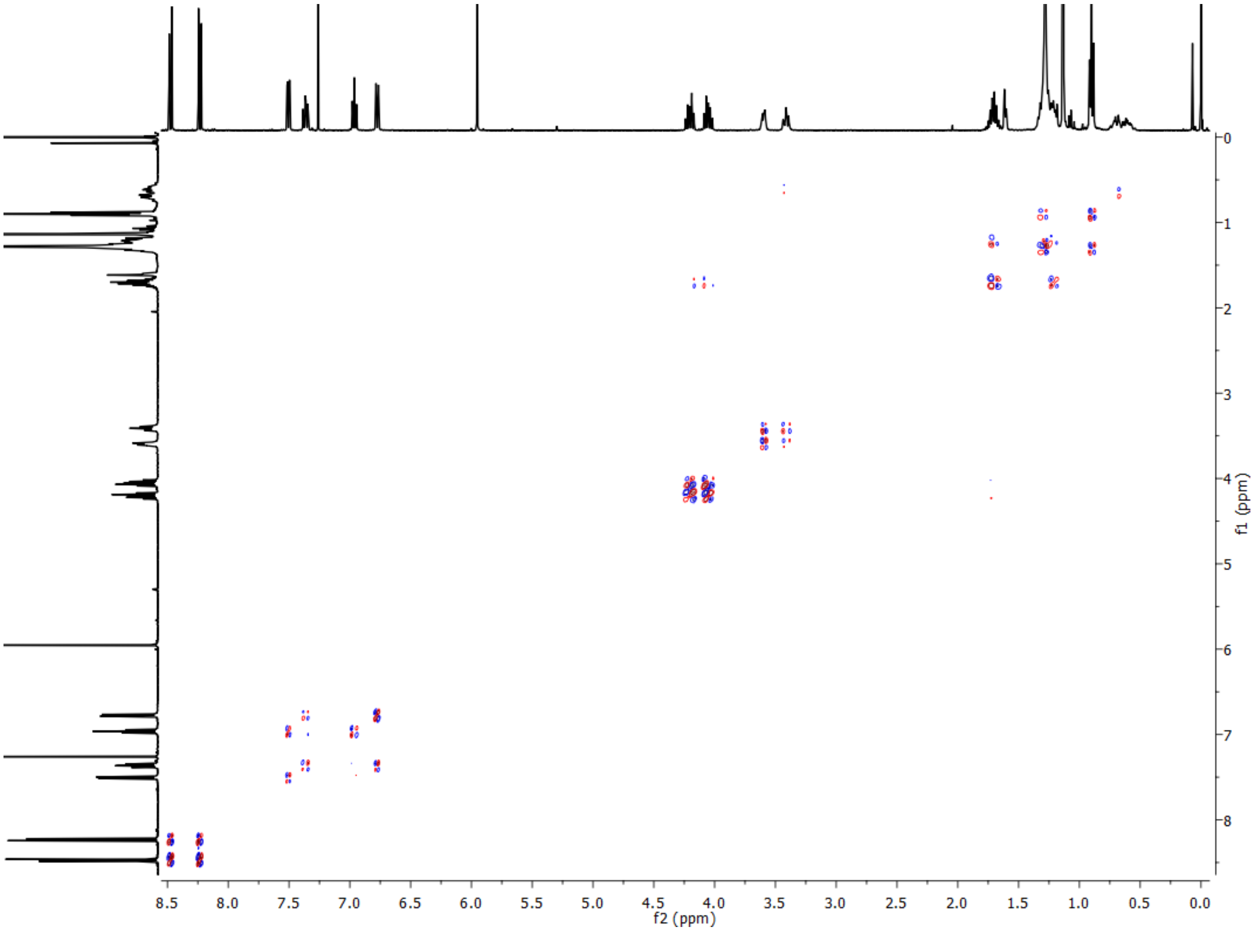

Supplementary Figure 157. COSY NMR (400 MHz) of *P*-**1-Ant-C4** in $CDCl_3$, measured at 298 K.

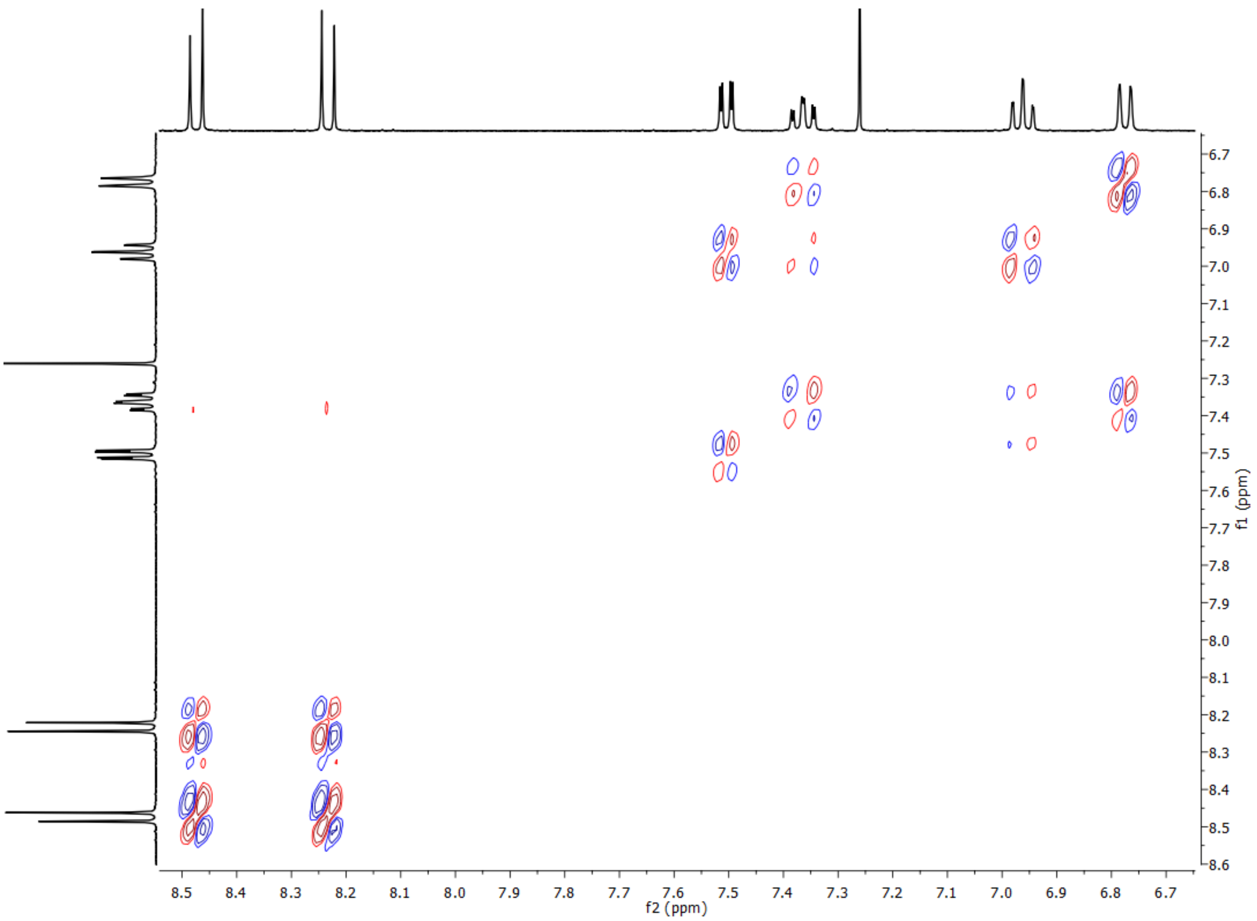

Supplementary Figure 158. COSY NMR (400 MHz) of *P*-**1-Ant-C4** in $CDCl_3$, measured at 298 K (expansion in aromatic region).

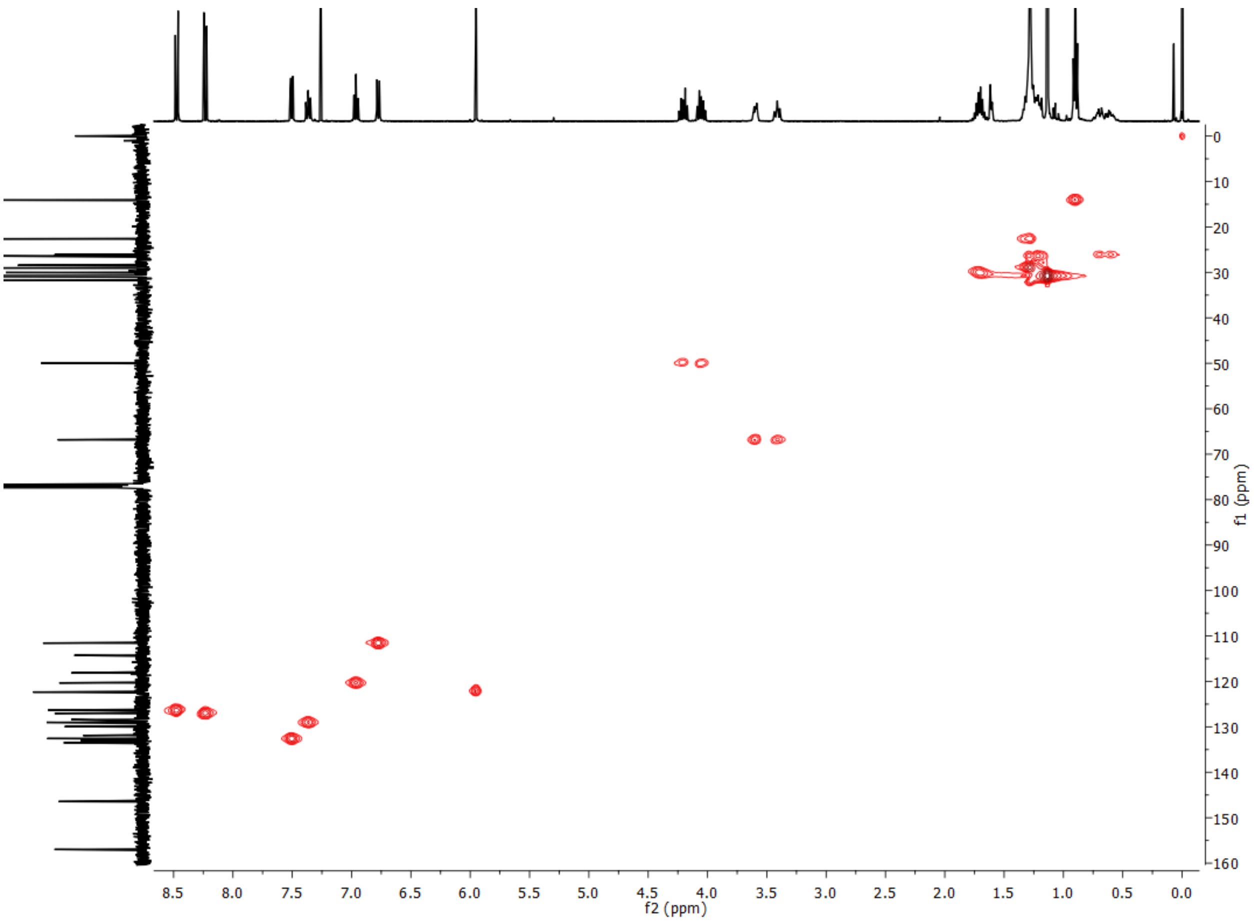

Supplementary Figure 159. HSQC NMR (400 MHz) of ***P*****-1-Ant-C4** in $CDCl_3$, measured at 298 K.

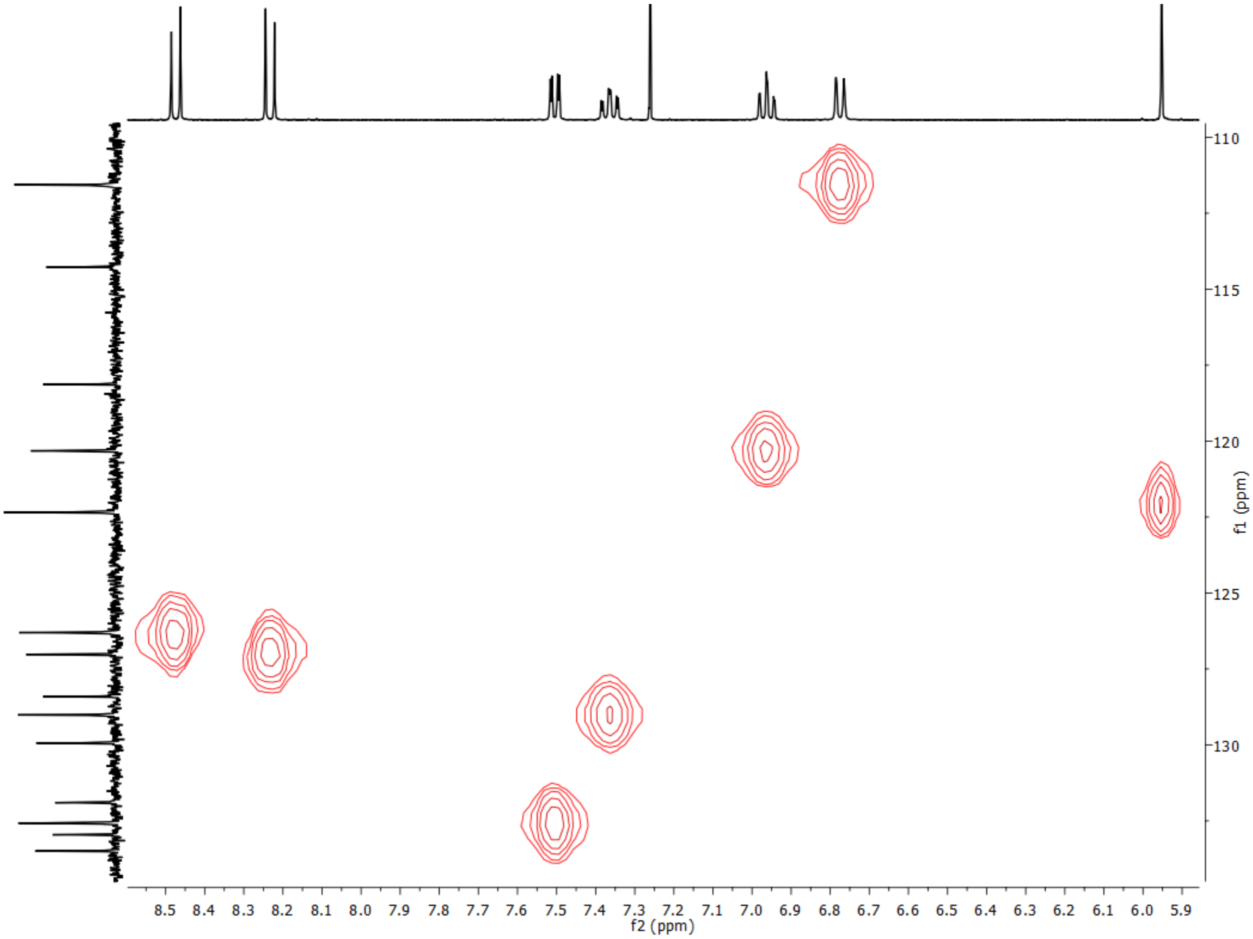

Supplementary Figure 160. HSQC NMR (400 MHz) of *P*-**1-Ant-C4** in $CDCl_3$, measured at 298 K (expansion in aromatic region).

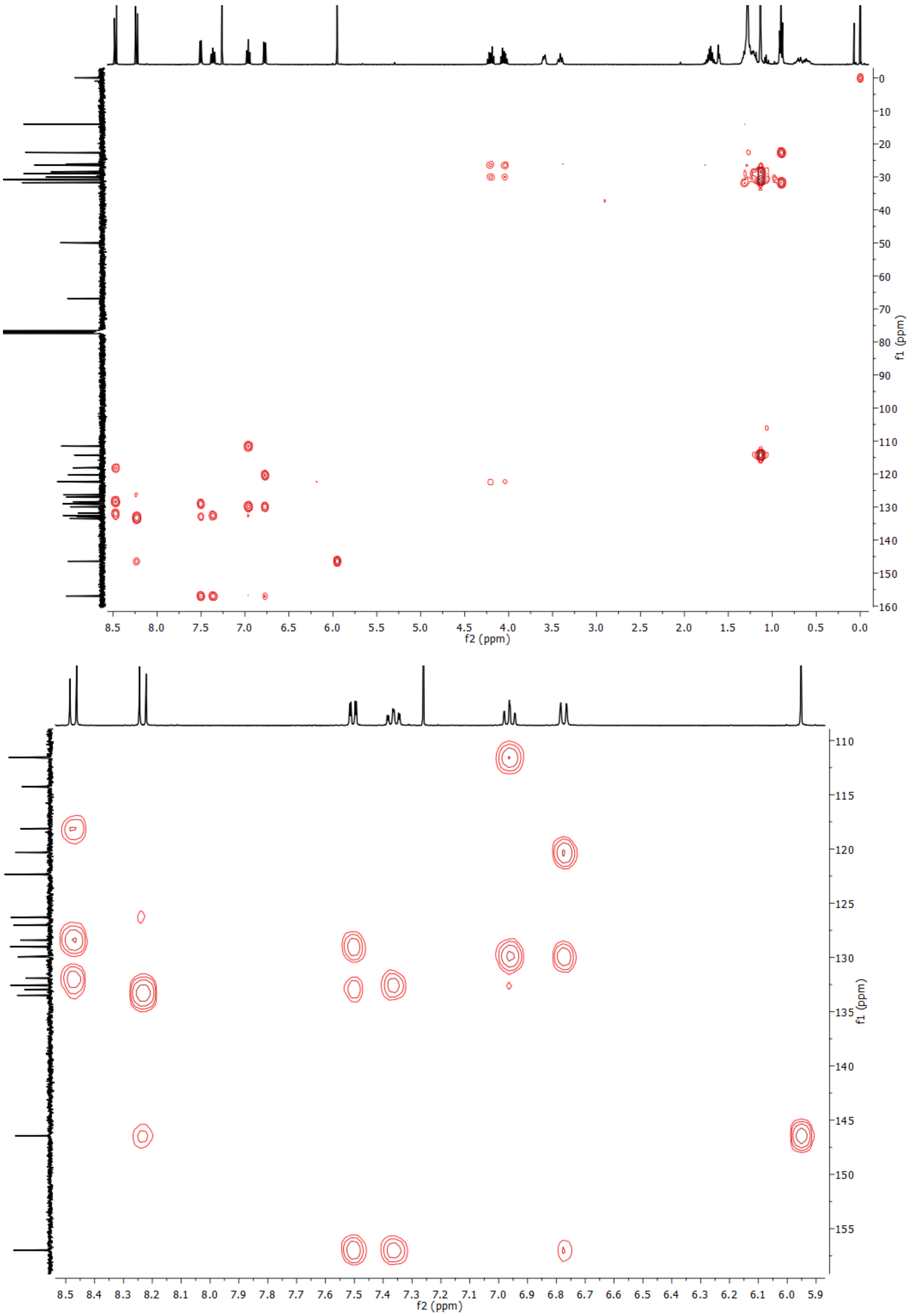

Supplementary Figure 161. HMBC NMR (400 MHz) of ***P*-1-Ant-C4** in $CDCl_3$, measured at 298 K (bottom: expansion in aromatic region).

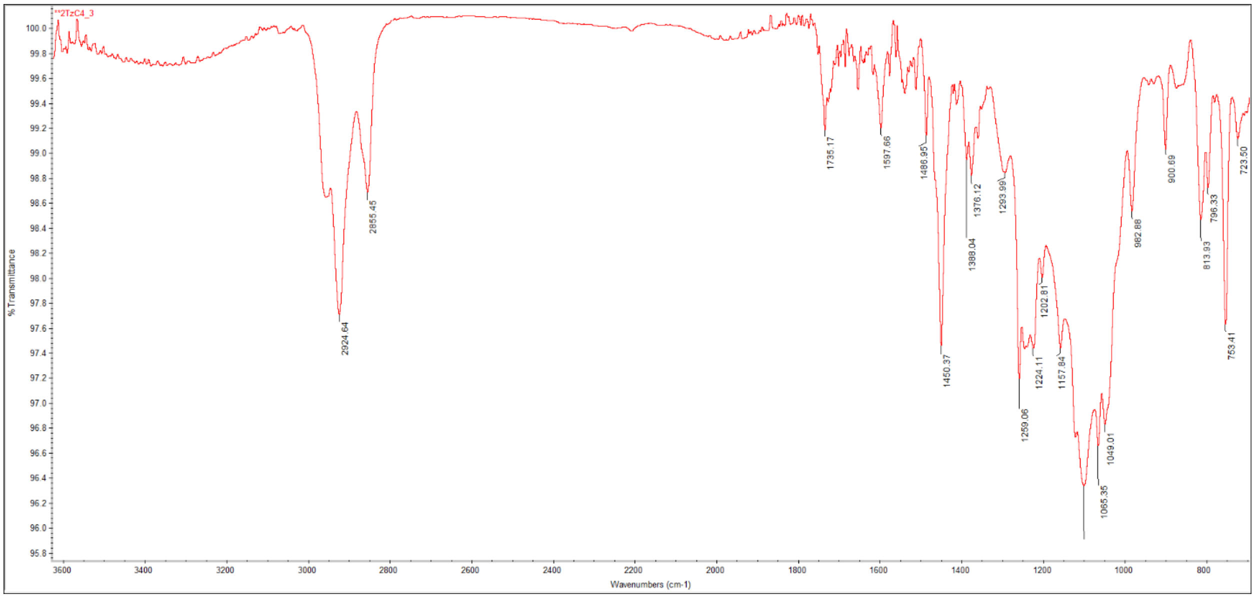

Supplementary Figure 162. ATR-FTIR of *P*-**1-Ant-C4**.

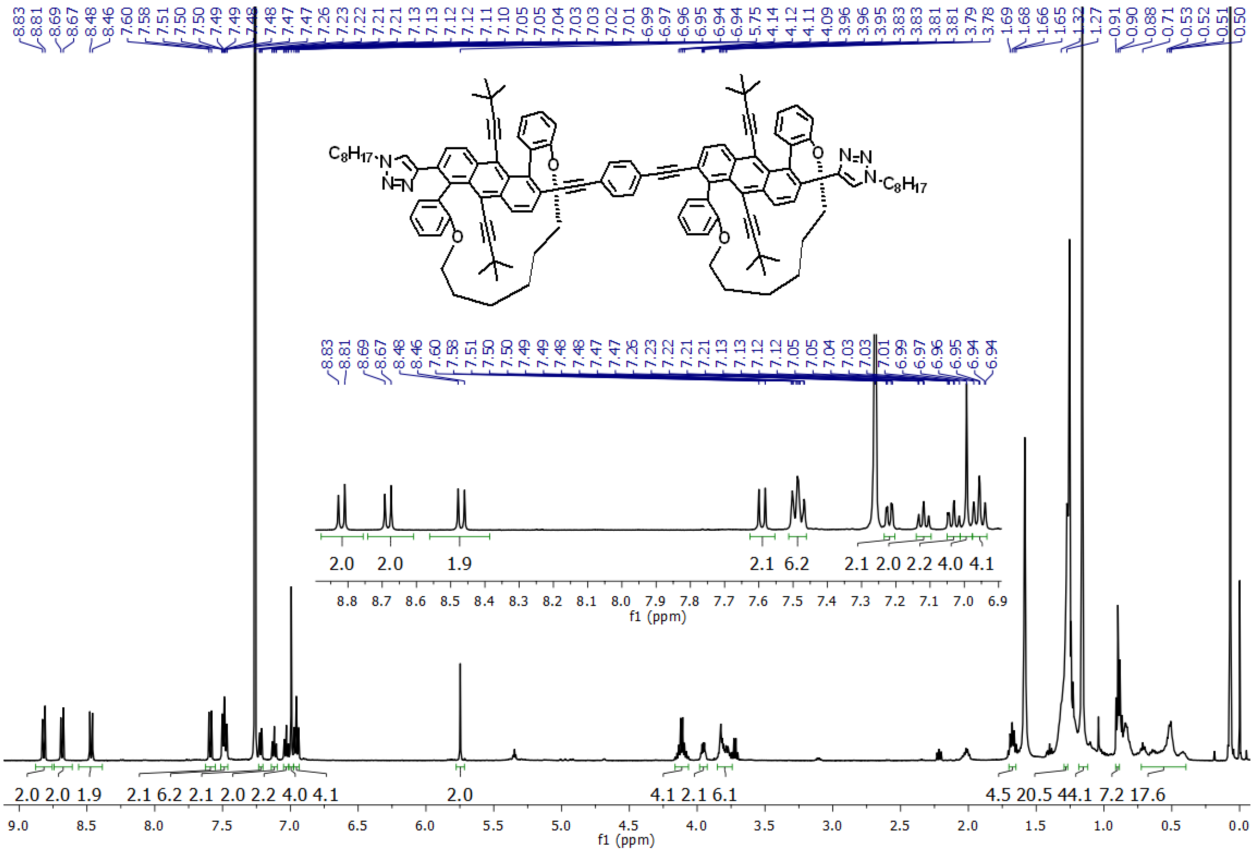

Supplementary Figure 163. H NMR (500 MHz) of *P*,*P*-**2-Ant-C8** in $CDCl_3$, measured at 298 K.

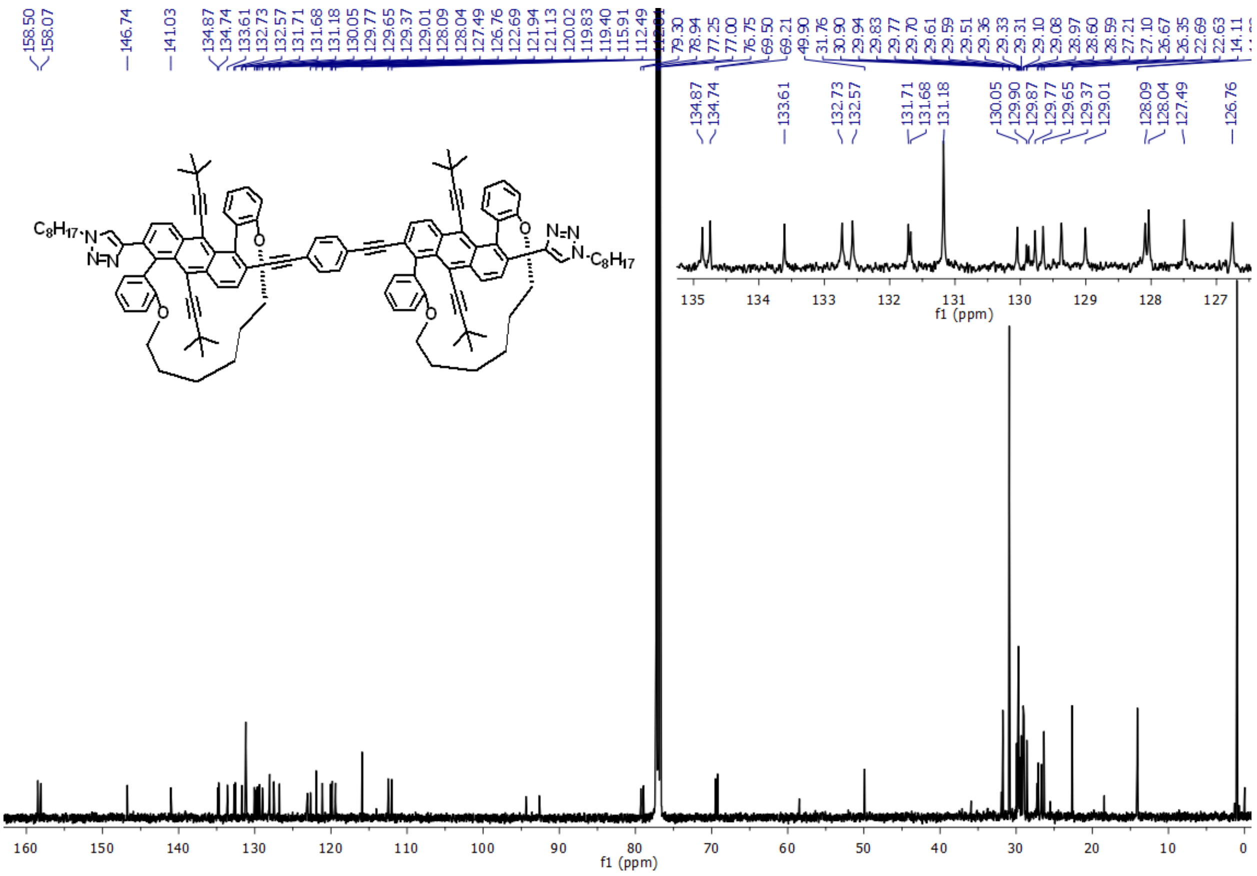

Supplementary Figure 164. ^{13}C NMR (126 MHz) of *P*,*P*-**2-Ant-C8** in $CDCl_3$, measured at 298 K.

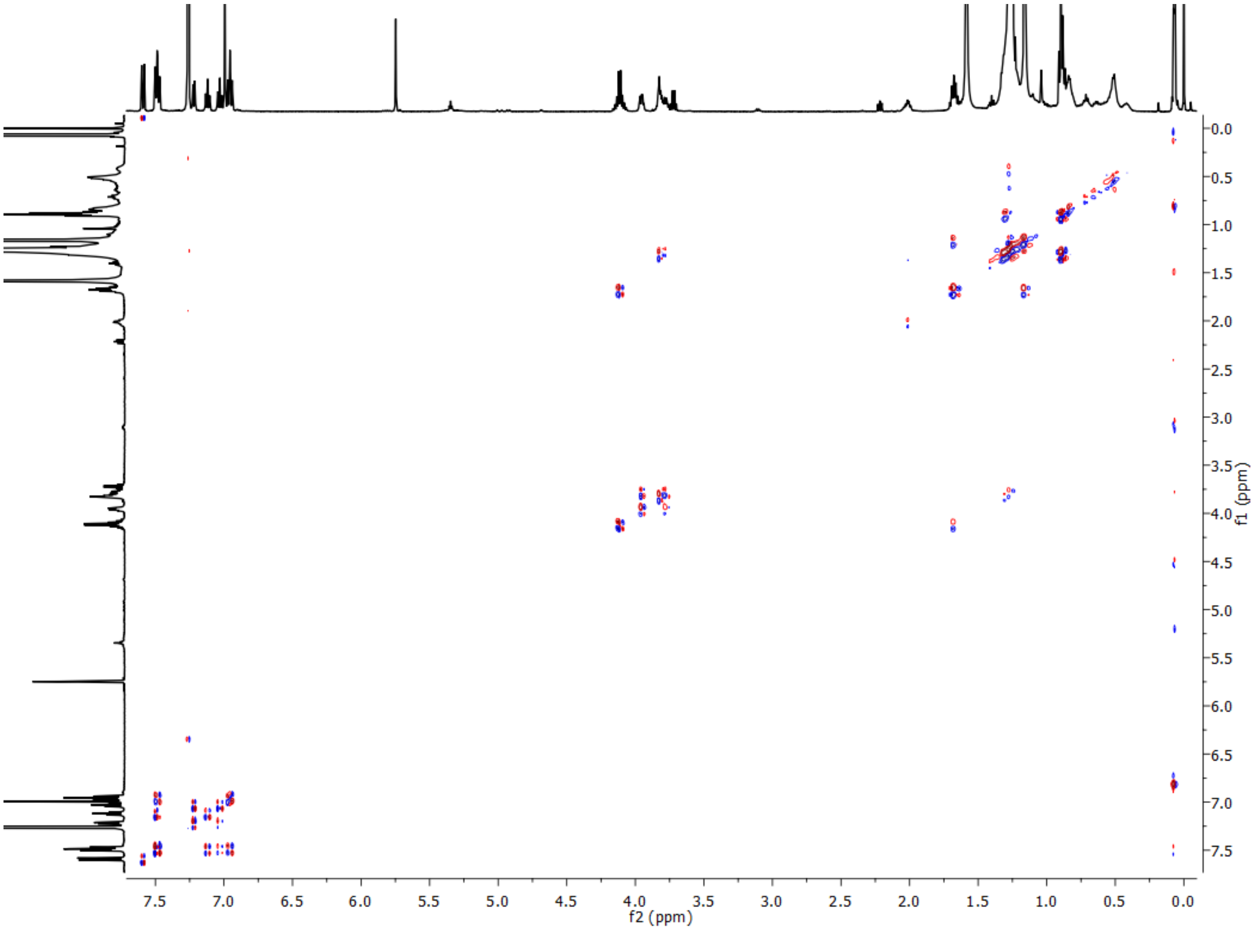

Supplementary Figure 165. COSY NMR (500 MHz) of *P*,*P*-**2-Ant-C8** in $CDCl_3$, measured at 298 K.

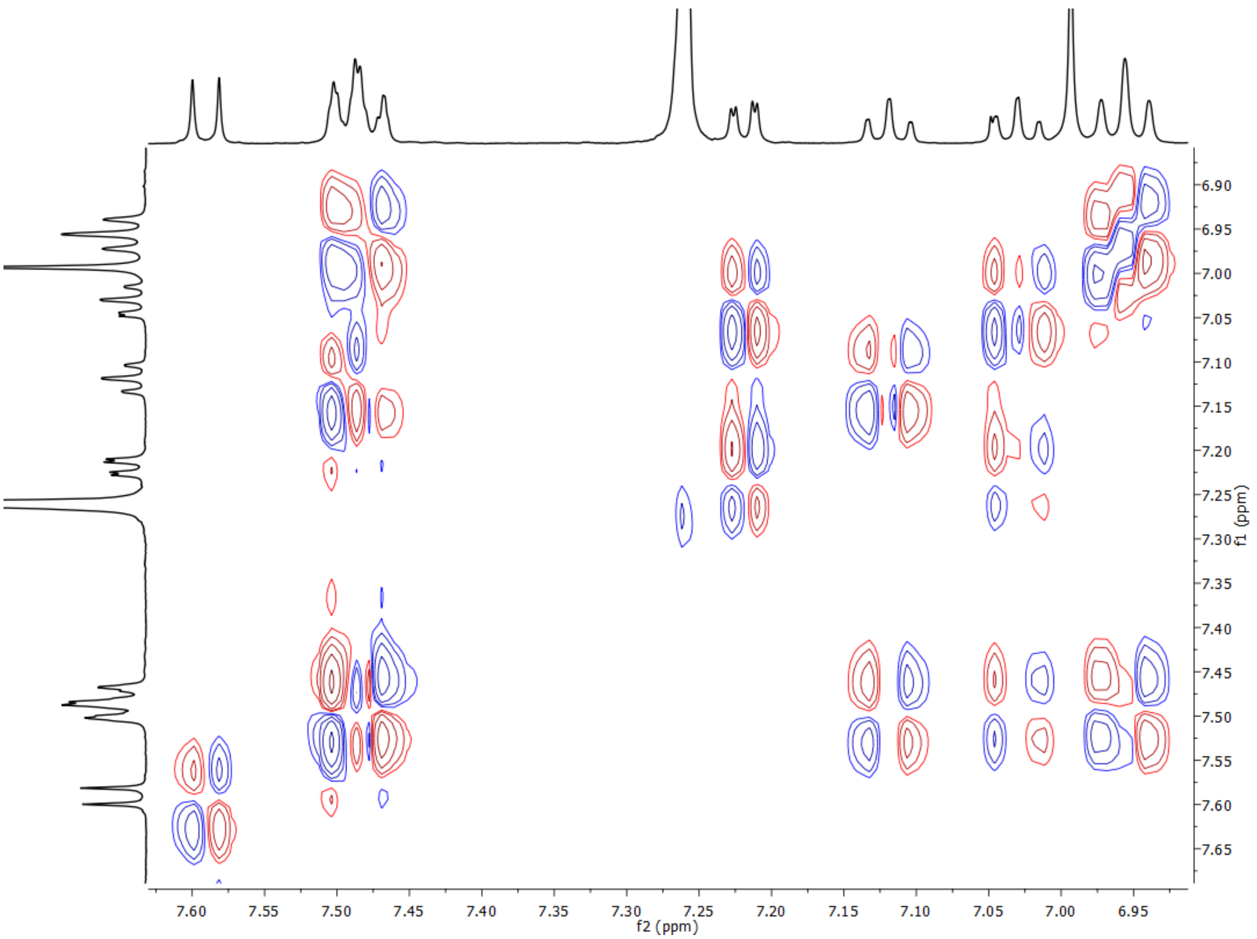

Supplementary Figure 166. COSY NMR (500 MHz) of *P*,*P*-**2-Ant-C8** in $CDCl_3$, measured at 298 K (expansion in aromatic region).

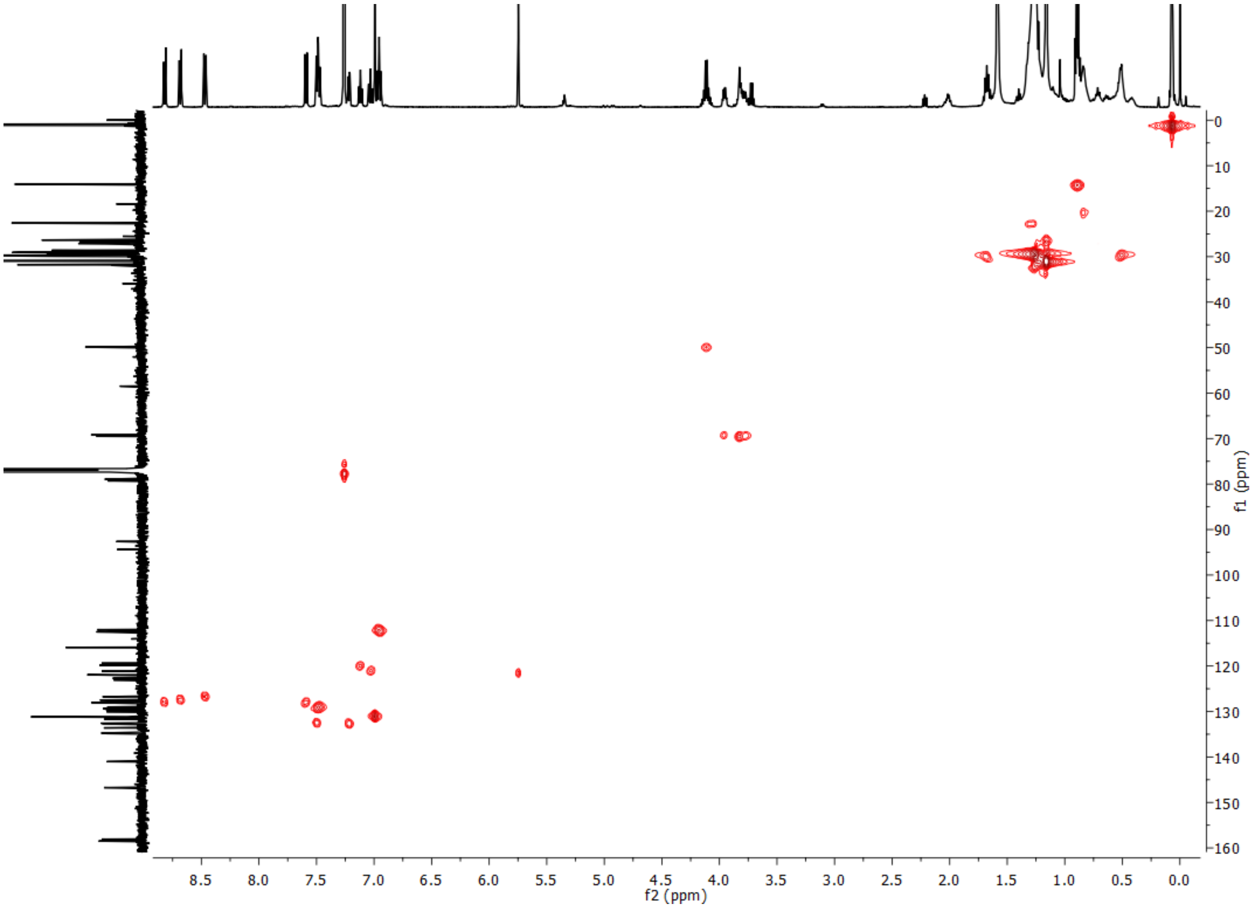

Supplementary Figure 167. HSQC NMR (500 MHz) of *P*,*P*-**2-Ant-C8** in $CDCl_3$, measured at 298 K.

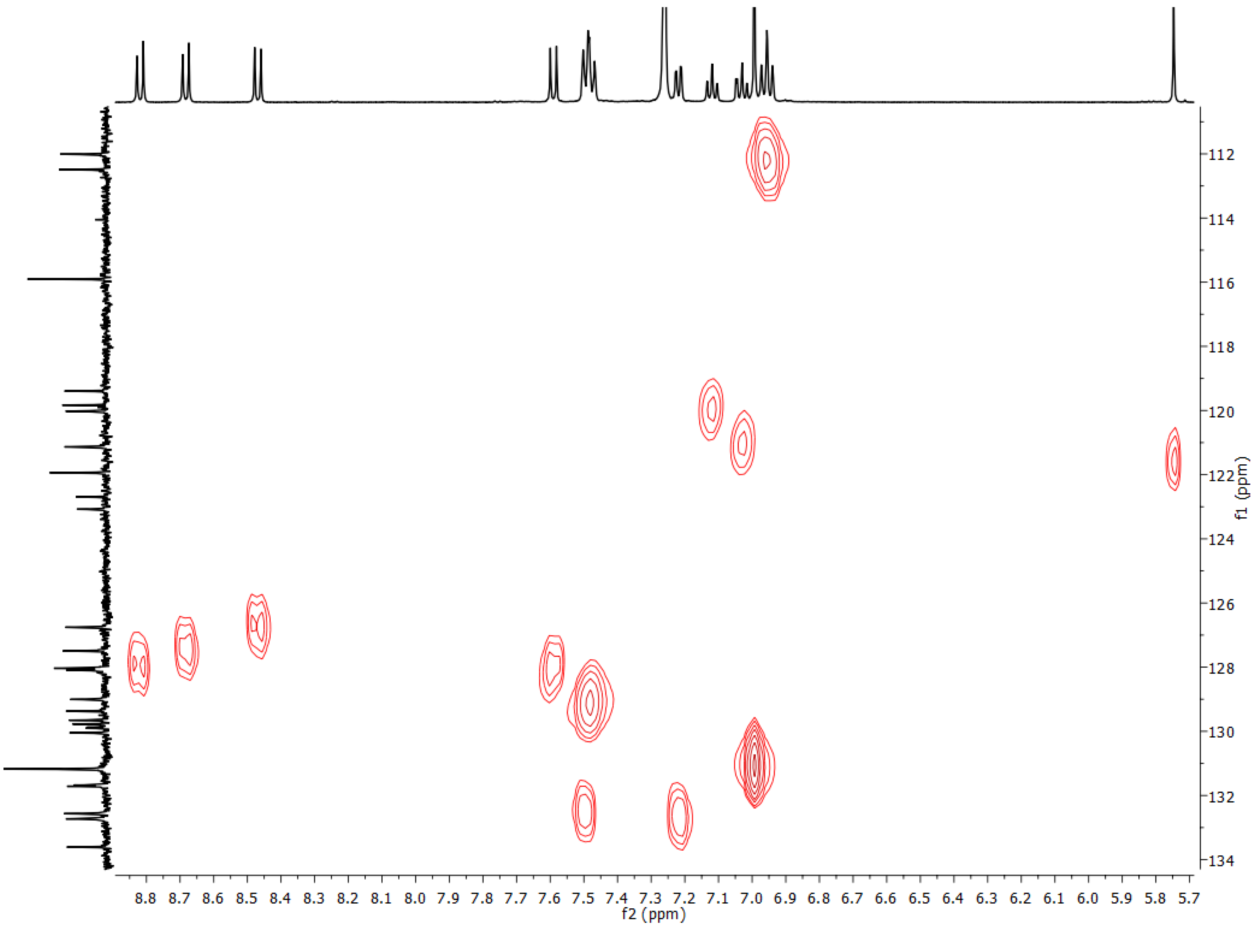

Supplementary Figure 168. HSQC NMR (500 MHz) of *P*,*P*-**2-Ant-C8** in $CDCl_3$, measured at 298 K (expansion in aromatic region).

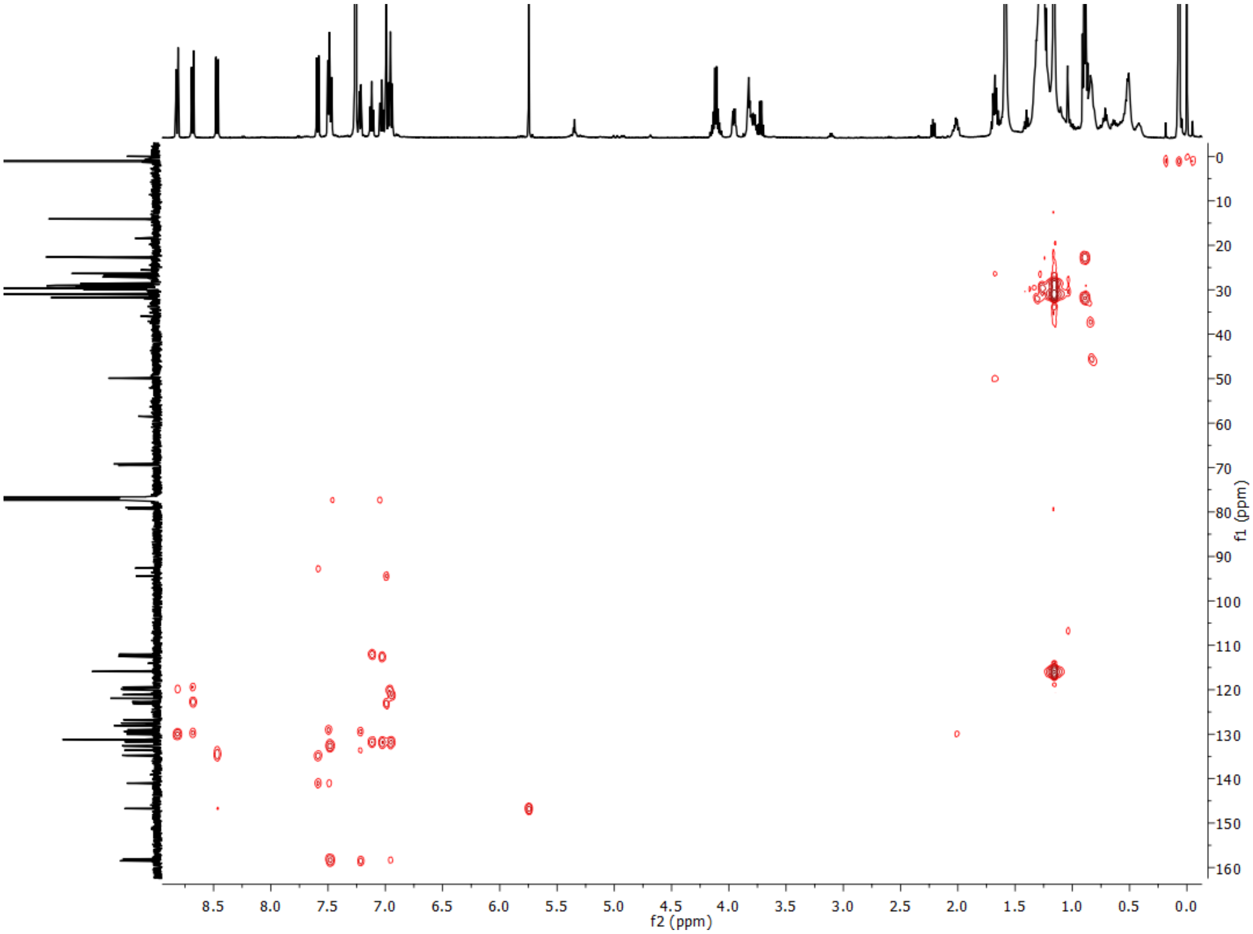

Supplementary Figure 169. HMBC NMR (500 MHz) of *P*,*P*-**2-Ant-C8**in $CDCl_3$, measured at 298 K.

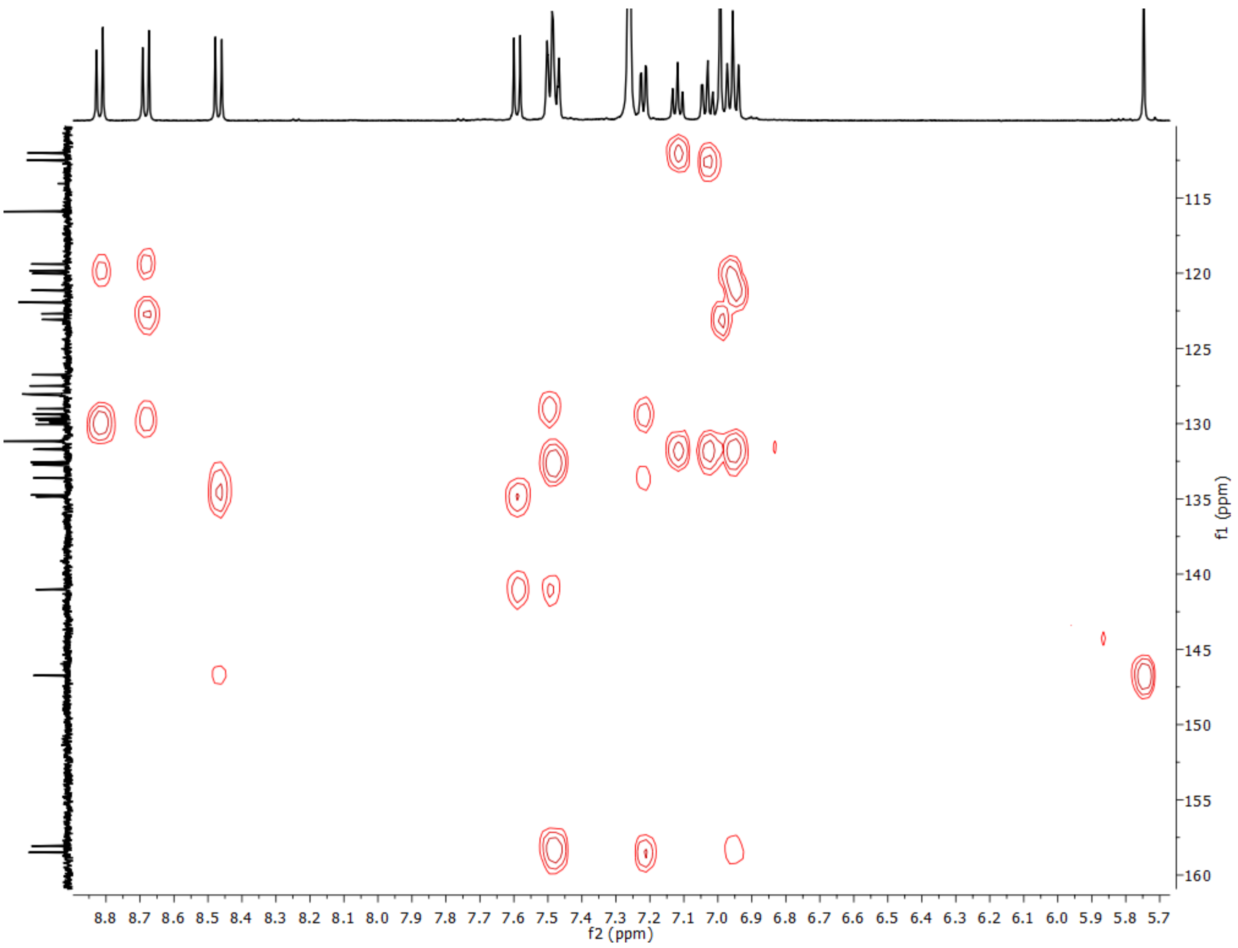

Supplementary Figure 170. HMBC NMR (500 MHz) of *P*,*P*-**2-Ant-C8** in $CDCl_3$, measured at 298 K (expansion in aromatic region).

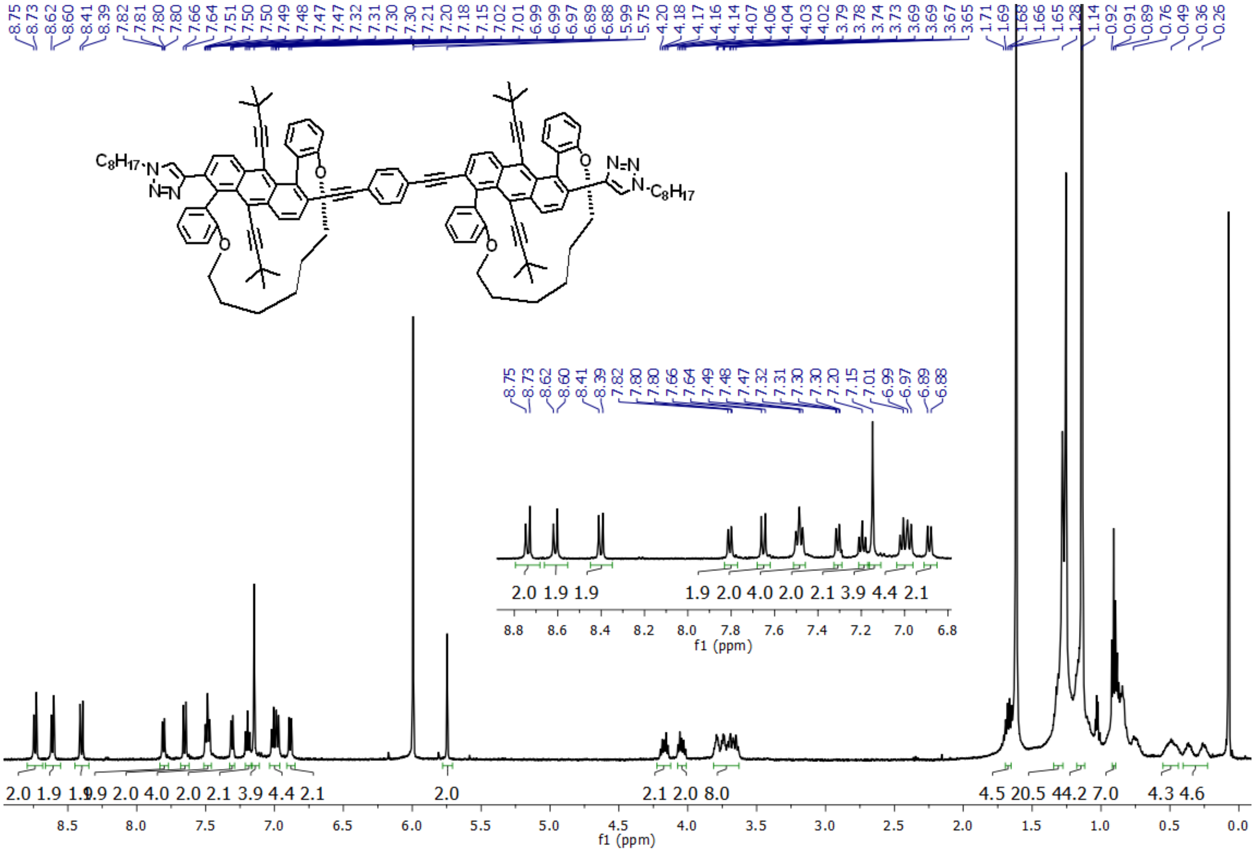

Supplementary Figure 171. ^{1}H NMR (500 MHz) of *P*,*P*-**2-Ant-C6** in C_2D_2Cl4, measured at 298 K.

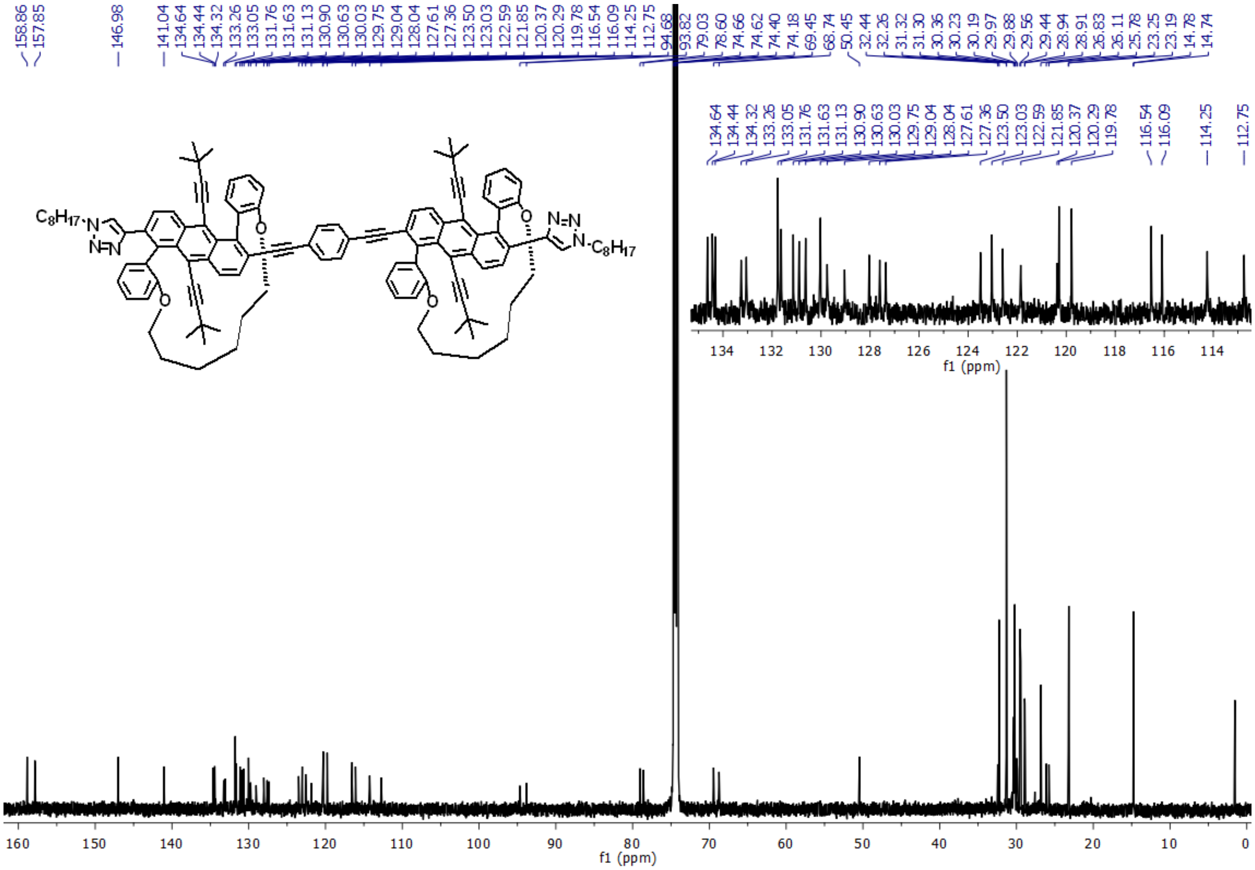

Supplementary Figure 172. ^{13}C NMR (126 MHz) of *P*,*P*-**2-Ant-C6** in C_2D_2Cl4, measured at 298 K.

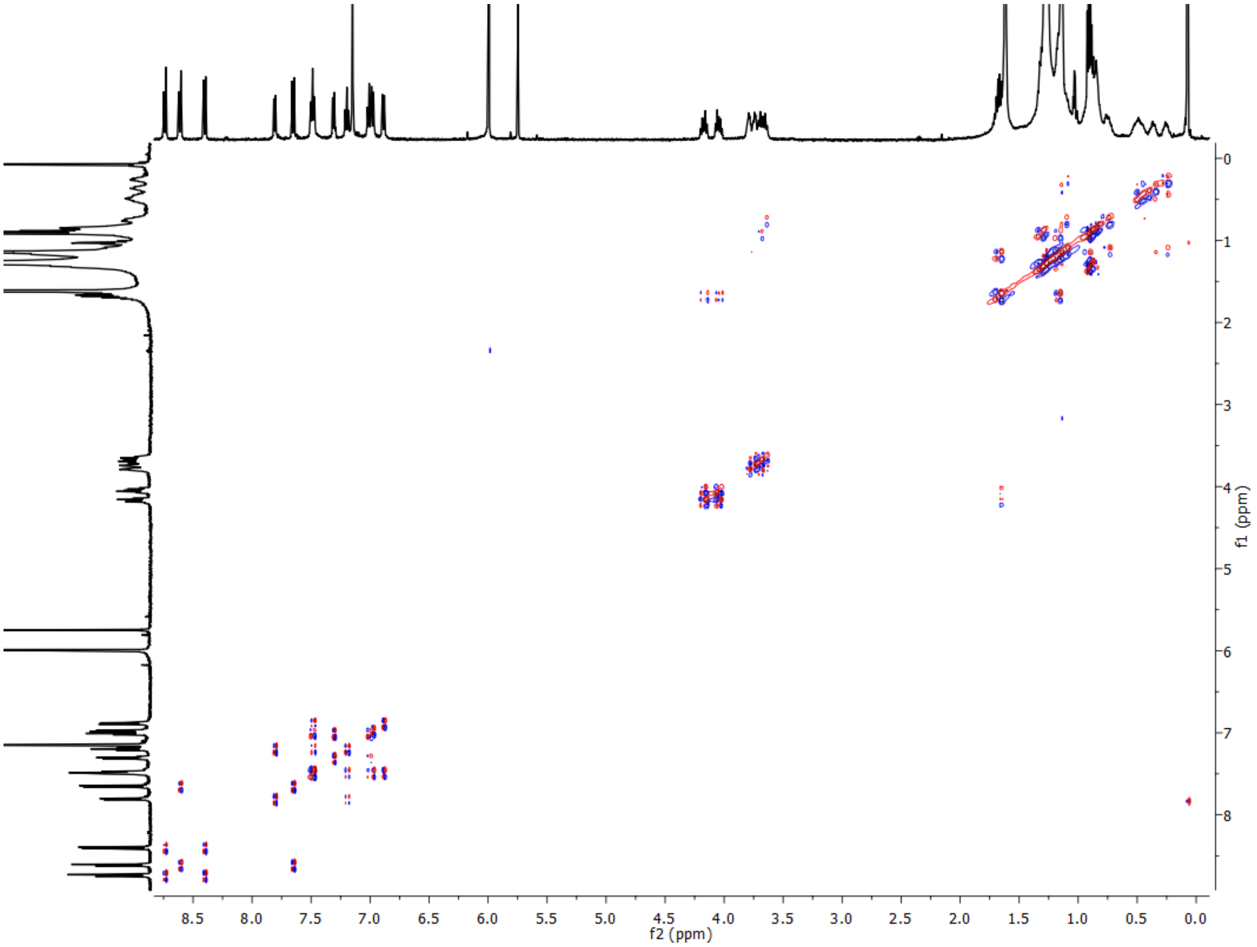

Supplementary Figure 173. COSY NMR (500 MHz) of *P*,*P*-**2-Ant-C6** in C_2D_2Cl4, measured at 298 K.

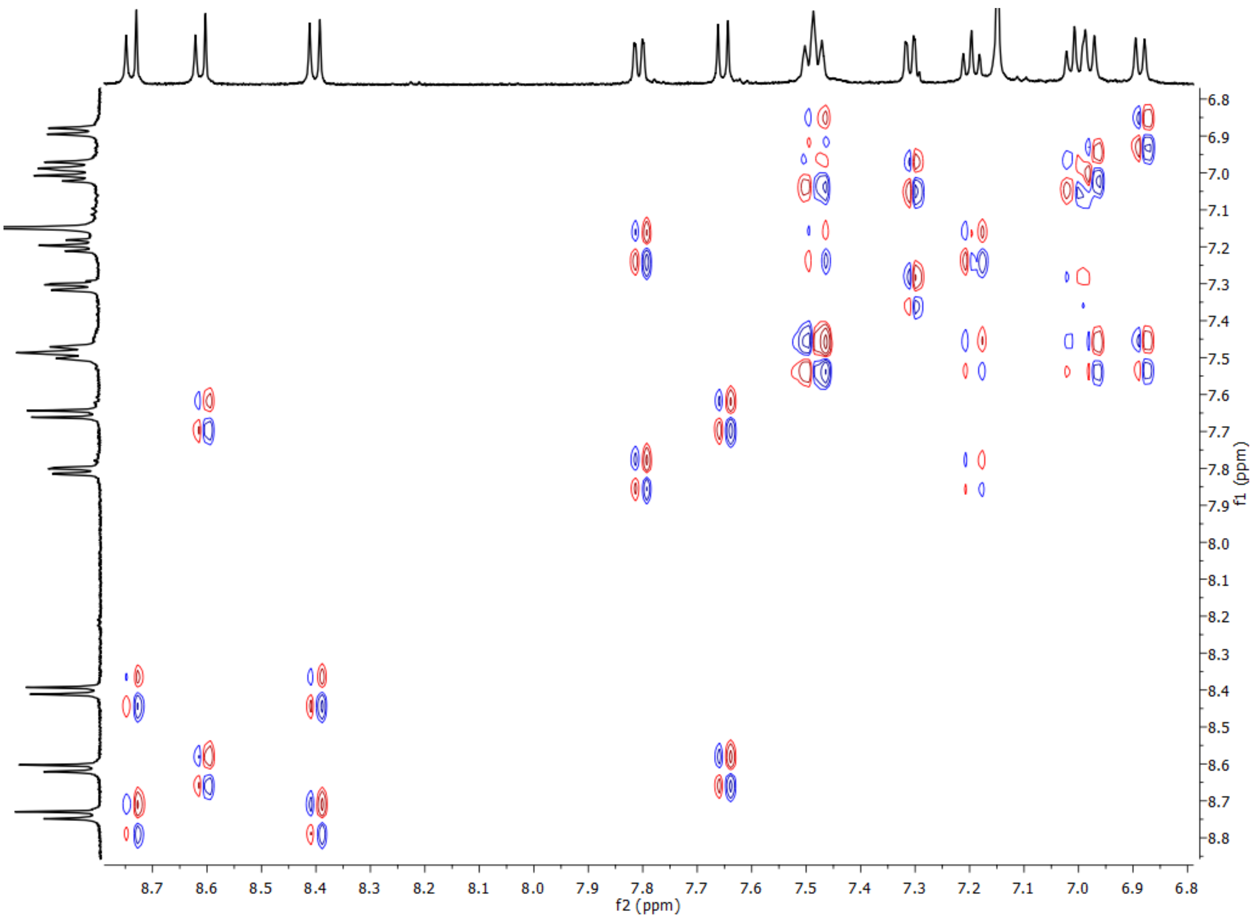

Supplementary Figure 174. COSY NMR (500 MHz) of ***P*,*P*-2-Ant-C6** in C_2D_2Cl4, measured at 298 K (expansion in aromatic region).

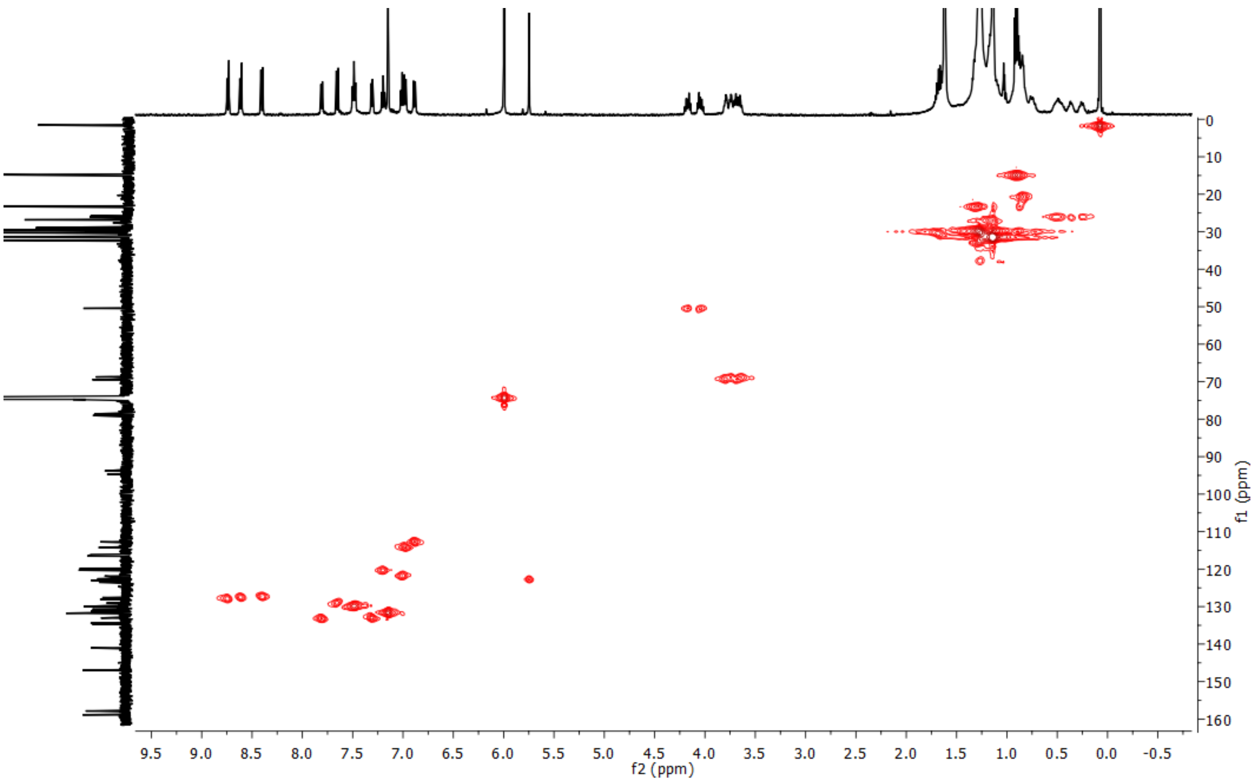

Supplementary Figure 175. HSQC NMR (500 MHz) of *P*,*P*-**2-Ant-C6** in C_2D_2Cl4, measured at 298 K.

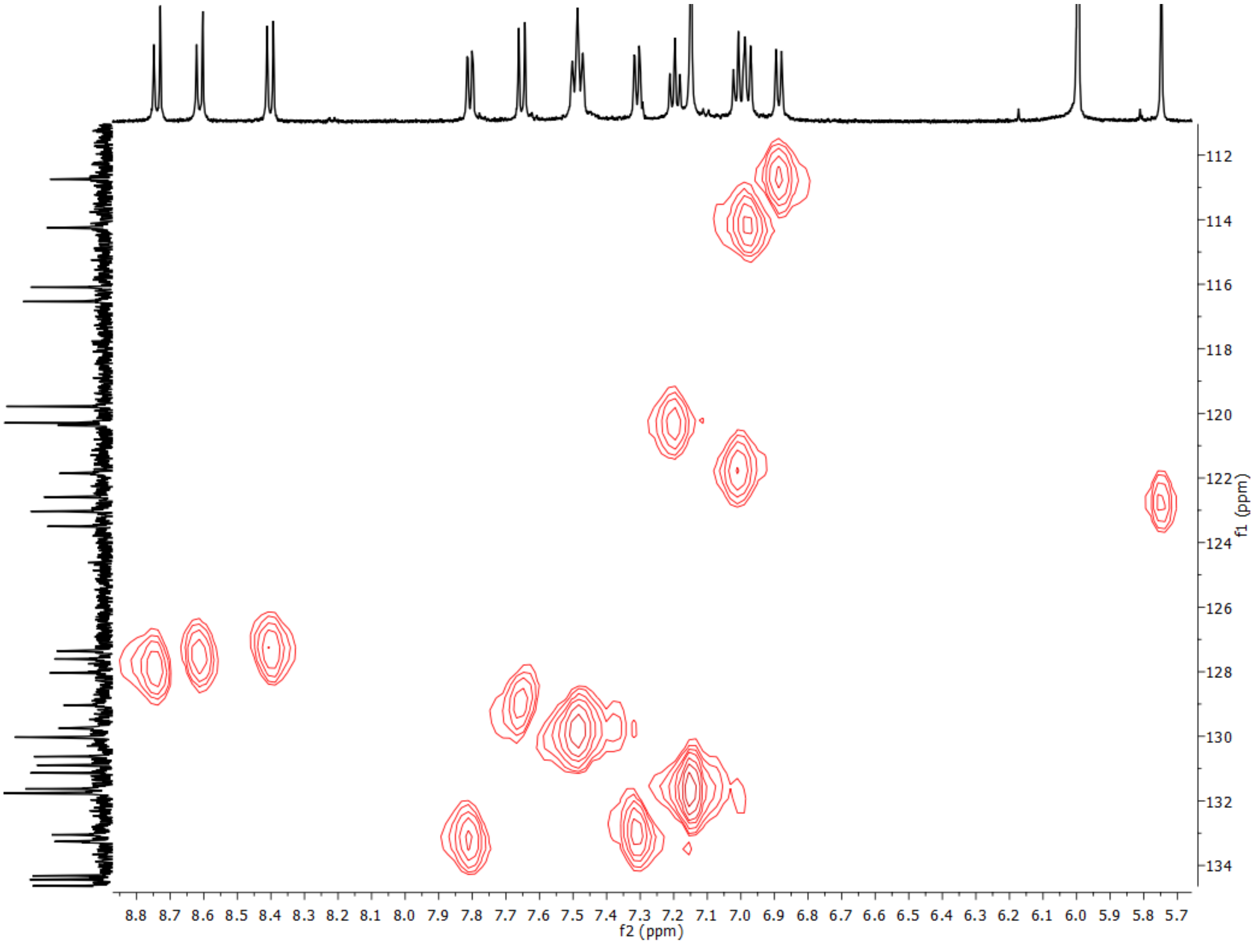

Supplementary Figure 176. HSQC NMR (500 MHz) *P*,*P*-**2-Ant-C6** in C_2D_2Cl4, measured at 298 K (expansion in aromatic region).

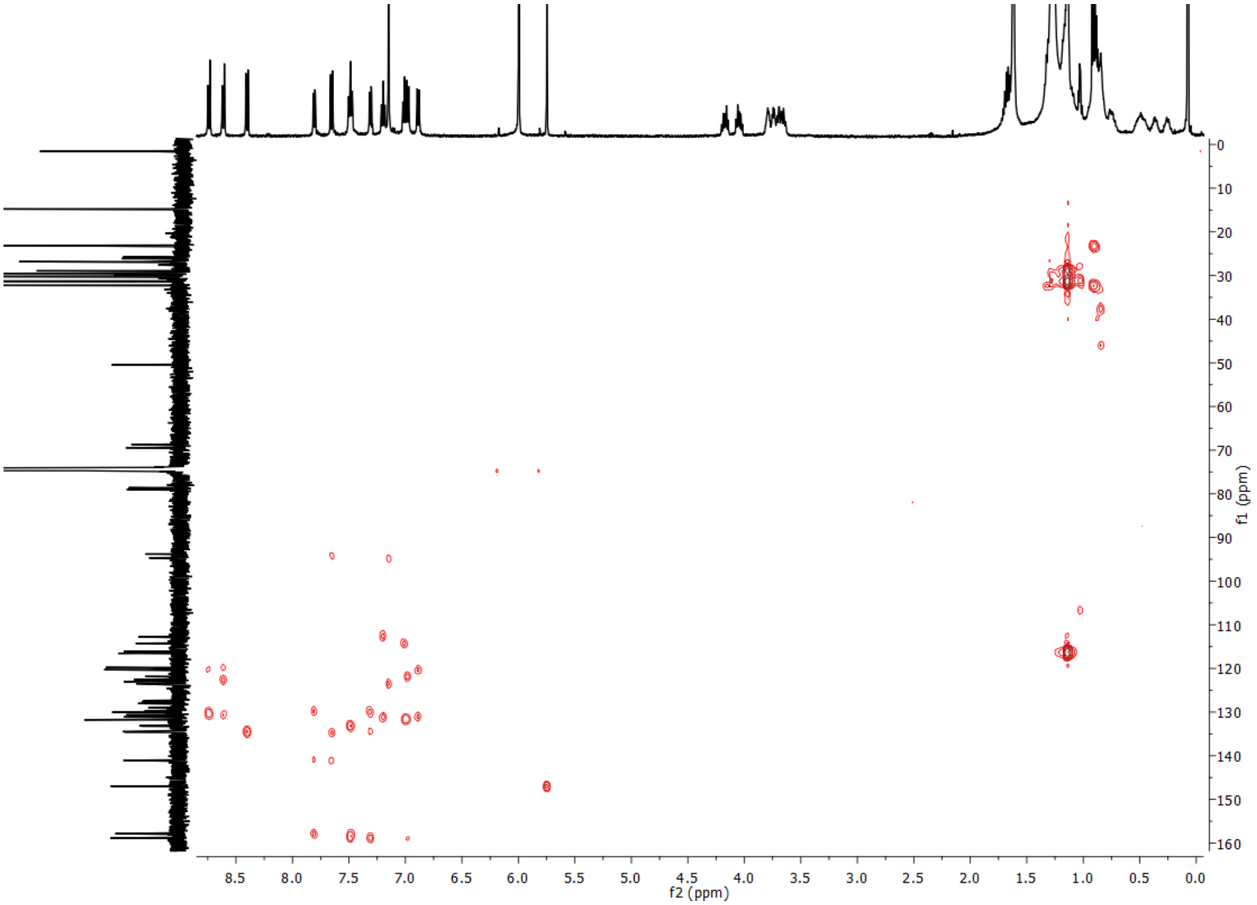

Supplementary Figure 177. HMBC NMR (500 MHz) of *P*,*P*-**2-Ant-C6** in C_2D_2Cl4, measured at 298 K.

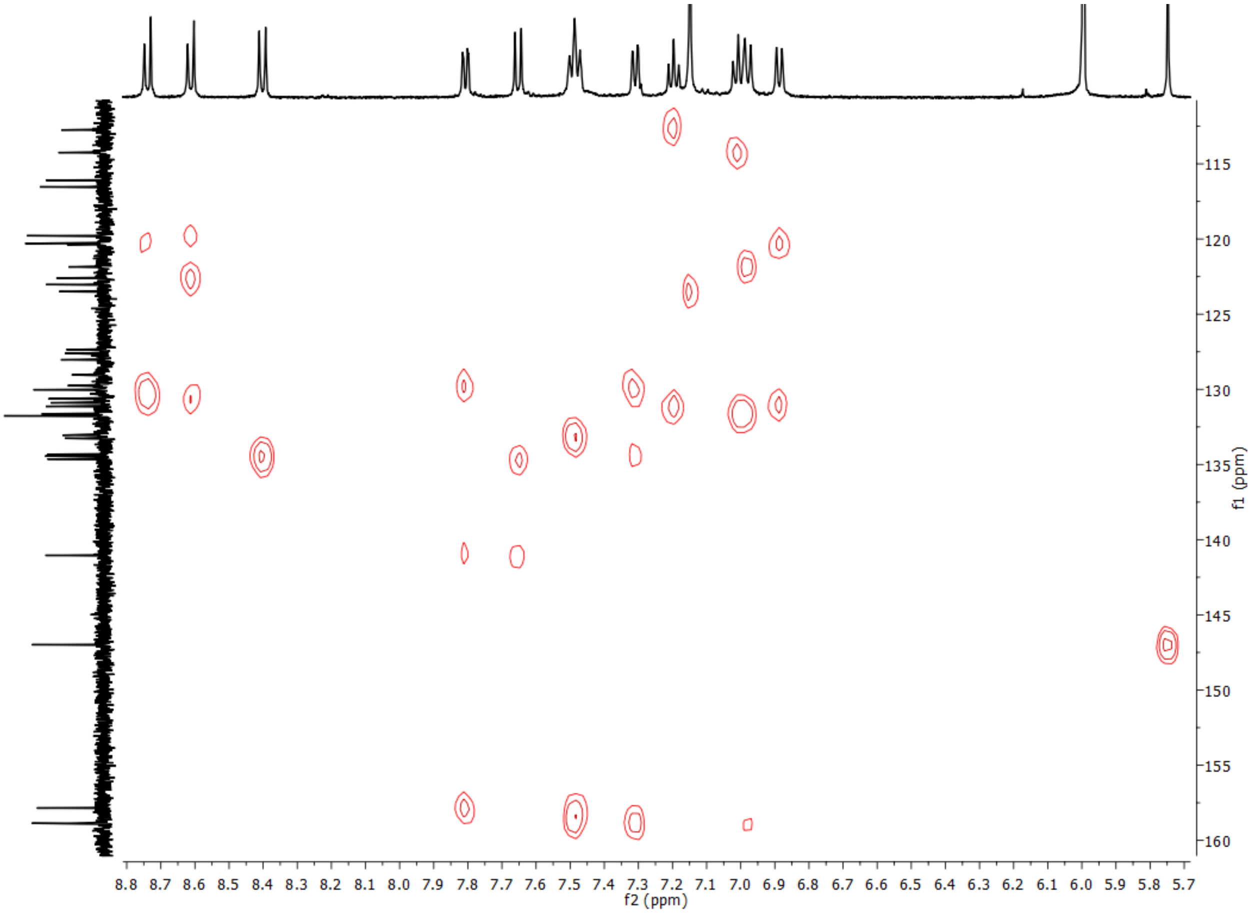

Supplementary Figure 178. HMBC NMR (500 MHz) of *P*,*P*-**2-Ant-C6** in C_2D_2Cl4, measured at 298 K (expansion in aromatic region).

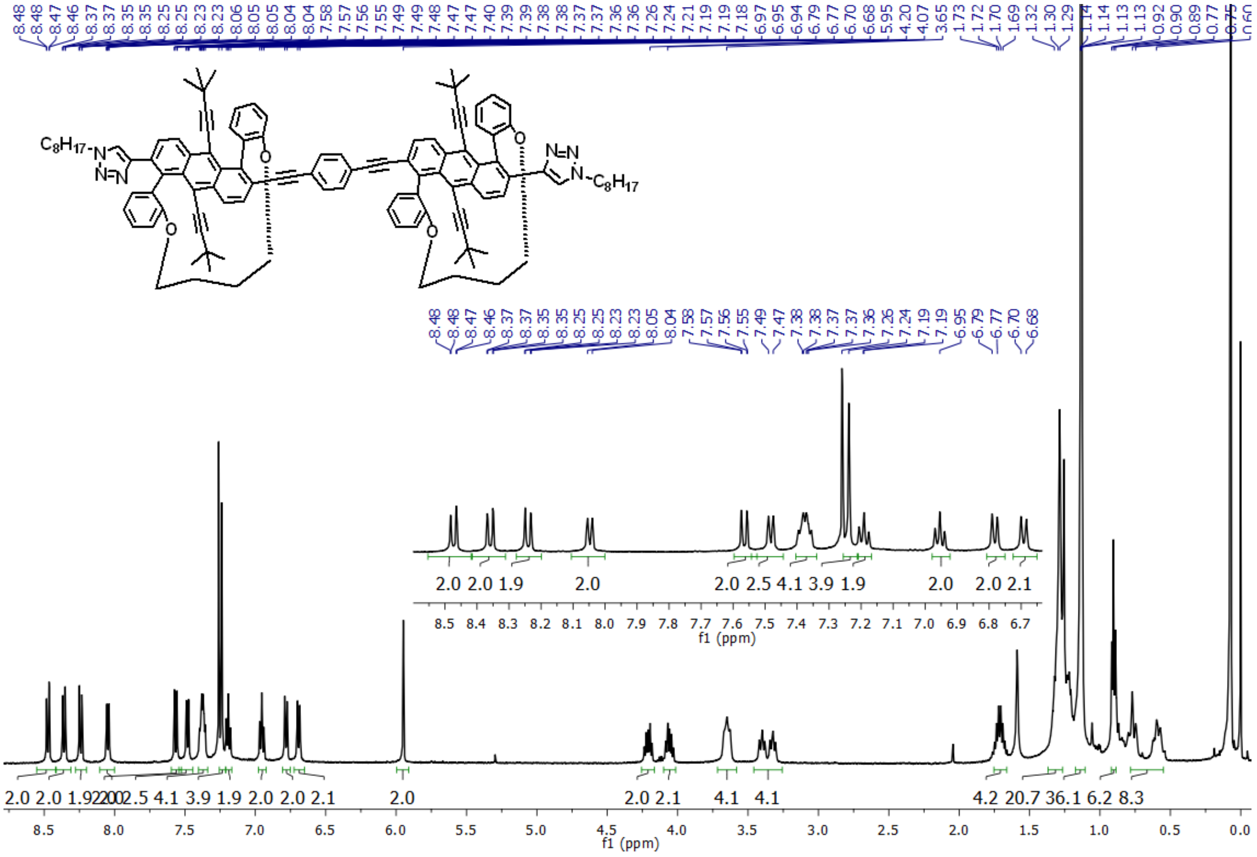

Supplementary Figure 179. ^{1}H NMR (500 MHz) of *P*,*P*-**2-Ant-C4** in $CDCl_3$, measured at 298 K.

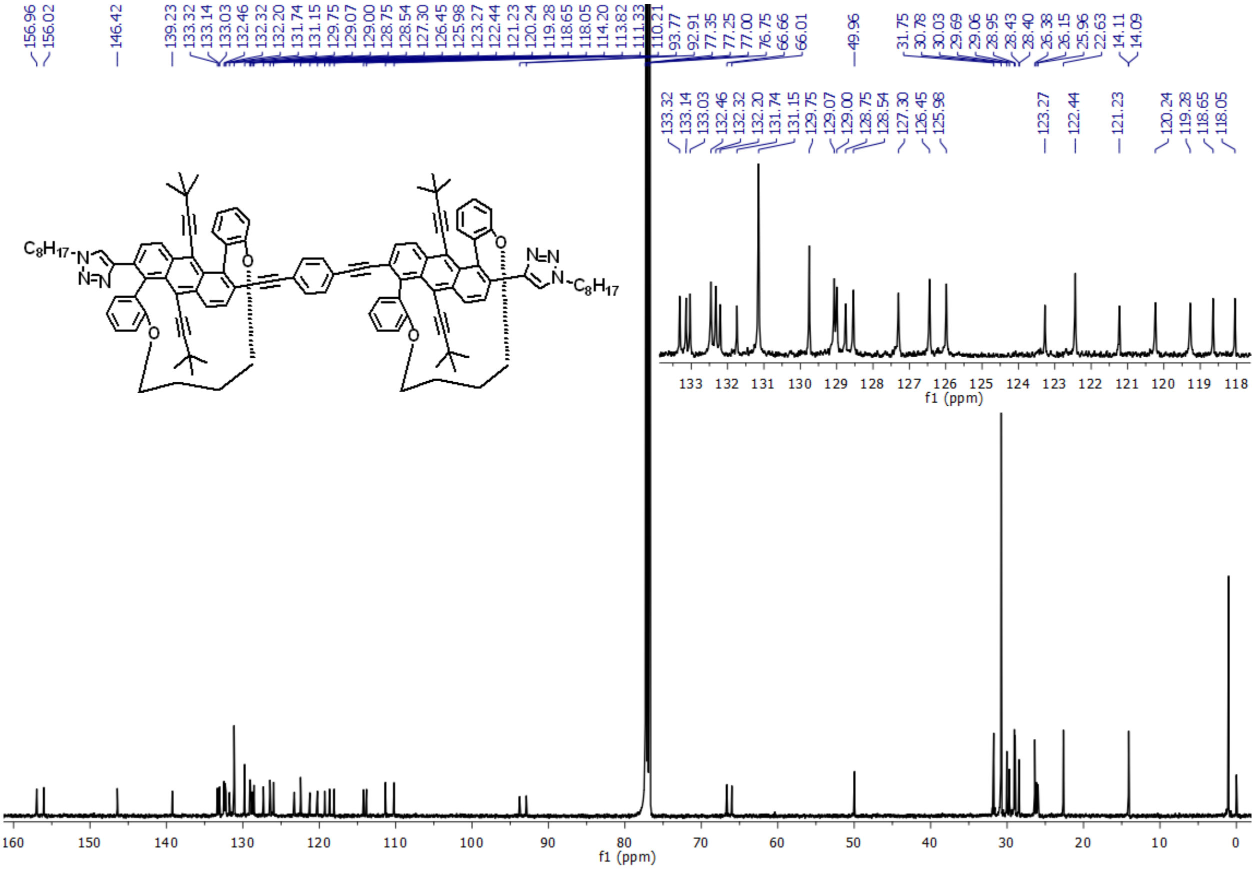

Supplementary Figure 180. ^{13}C NMR (126 MHz) of *P*,*P*-**2-Ant-C4** in $CDCl_3$, measured at 298 K.

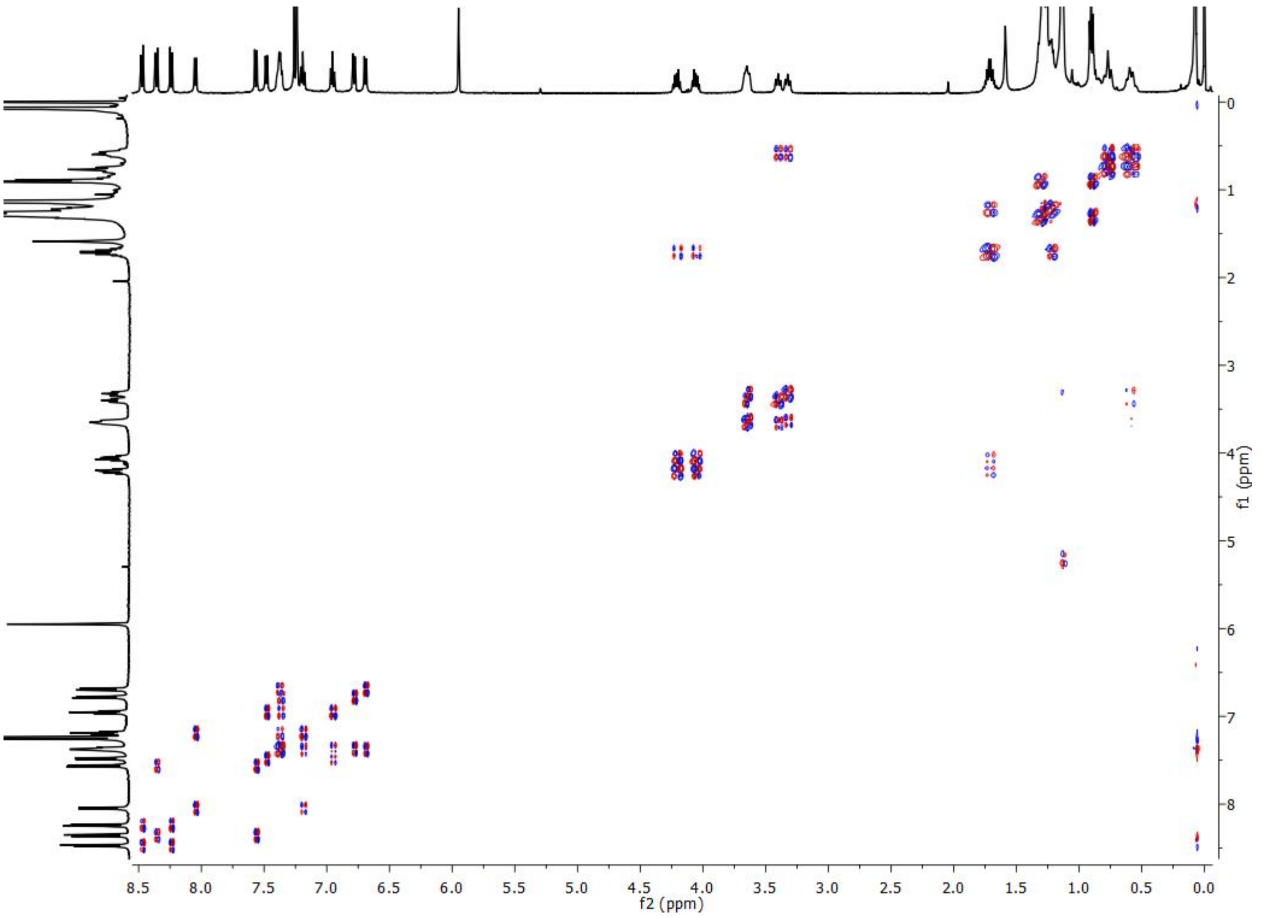

Supplementary Figure 181. COSY NMR (500 MHz) of *P*,*P*-**2-Ant-C4** in $CDCl_3$, measured at 298 K.

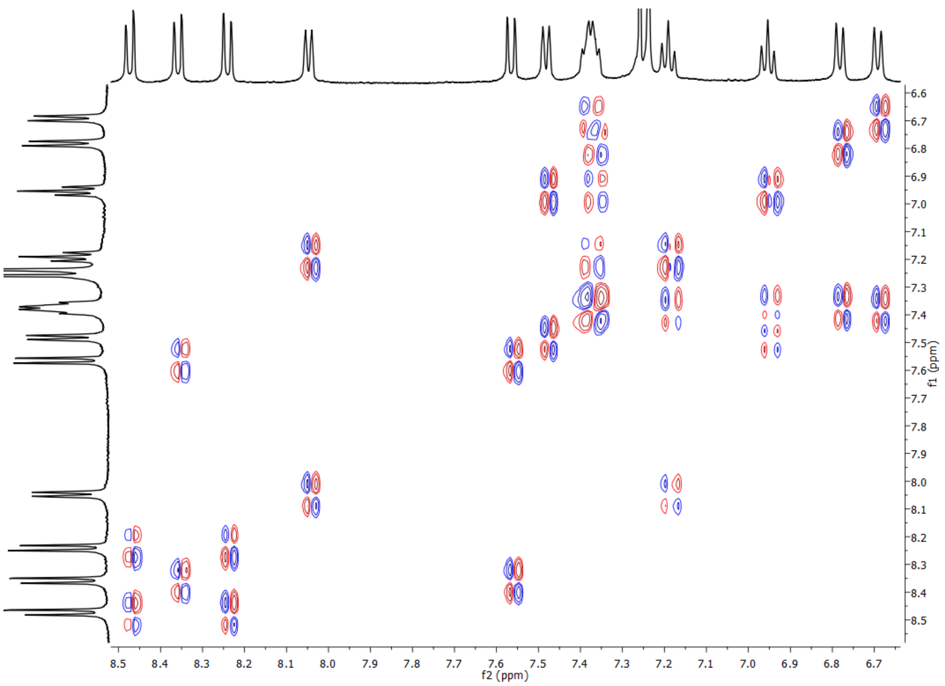

Supplementary Figure 182. COSY NMR (500 MHz) of *P*,*P*-**2-Ant-C4** in $CDCl_3$, measured at 298 K (expansion in aromatic region).

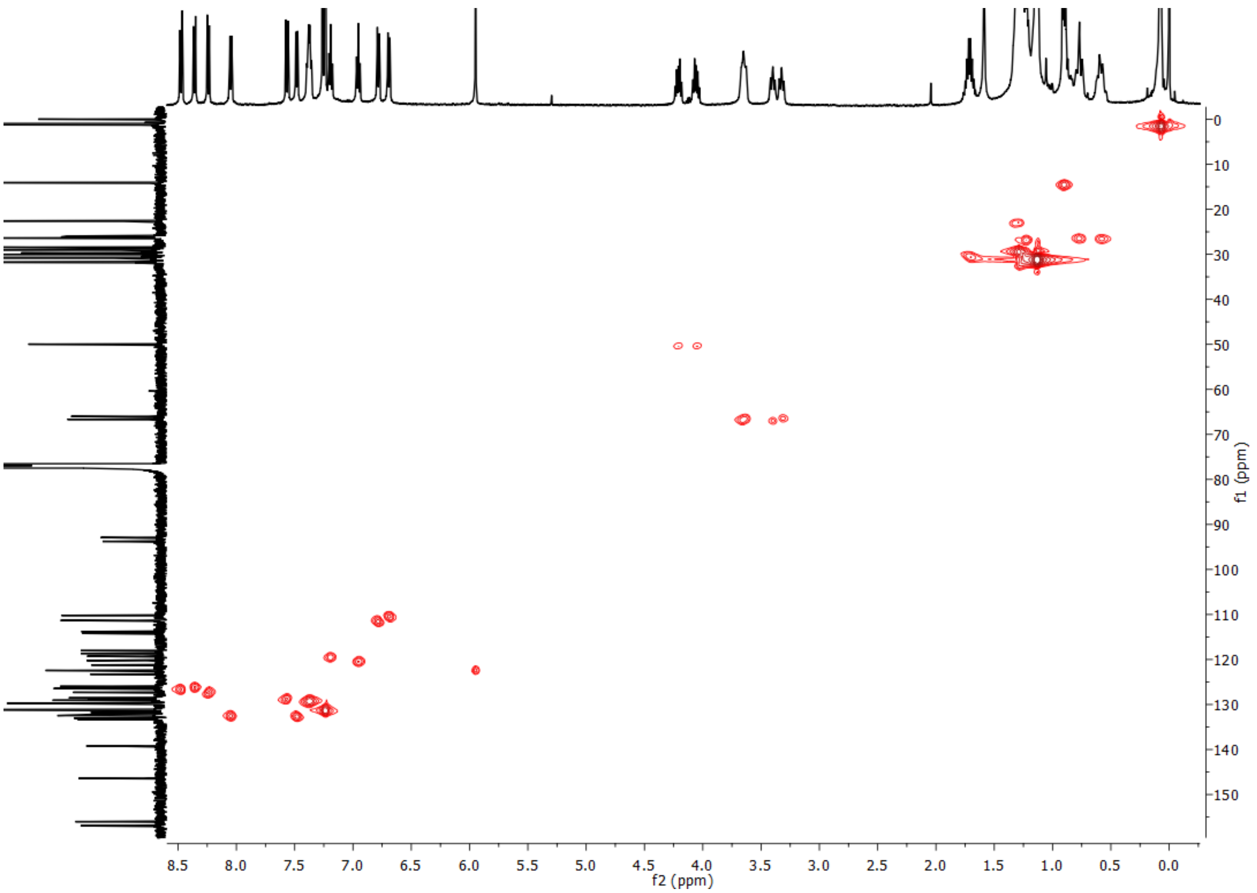

Supplementary Figure 183. HSQC NMR (500 MHz) of *P*,*P*-**2-Ant-C4** in $CDCl_3$, measured at 298 K.

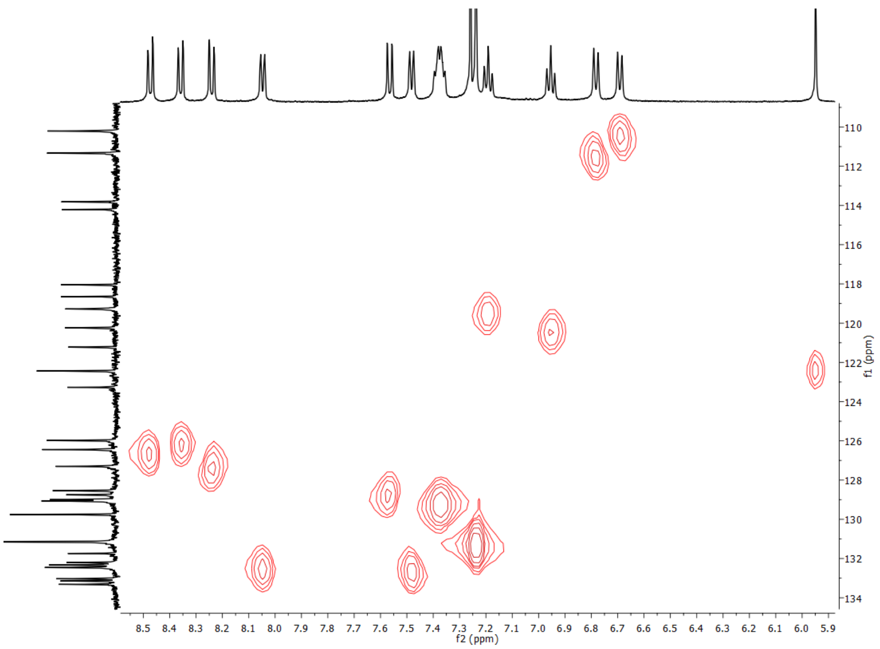

Supplementary Figure 184. HSQC NMR (500 MHz) of *P*,*P*-**2-Ant-C4** in $CDCl_3$, measured at 298 K (expansion in aromatic region).

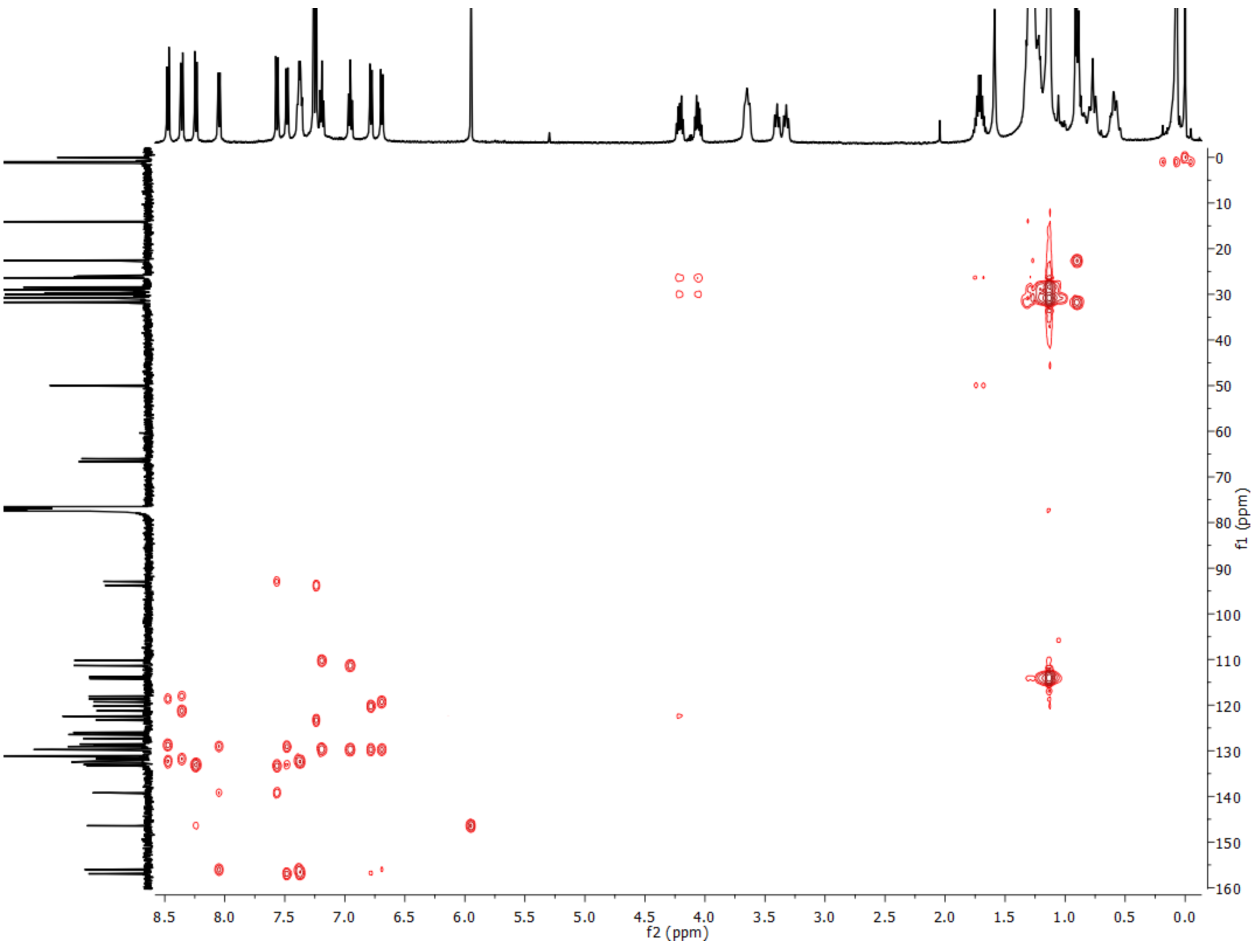

Supplementary Figure 185. HMBC NMR (500 MHz) of *P*,*P*-**2-Ant-C4** in $CDCl_3$, measured at 298 K.

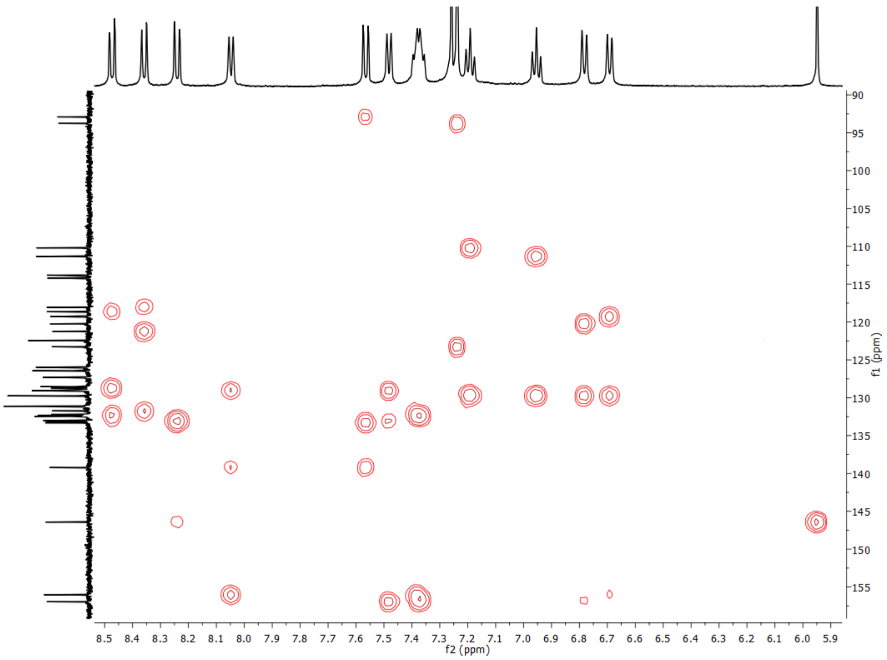

Supplementary Figure 186. HMBC NMR (500 MHz) of *P*,*P*-**2-Ant-C4** in $CDCl_3$, measured at 298 K (expansion in aromatic region).

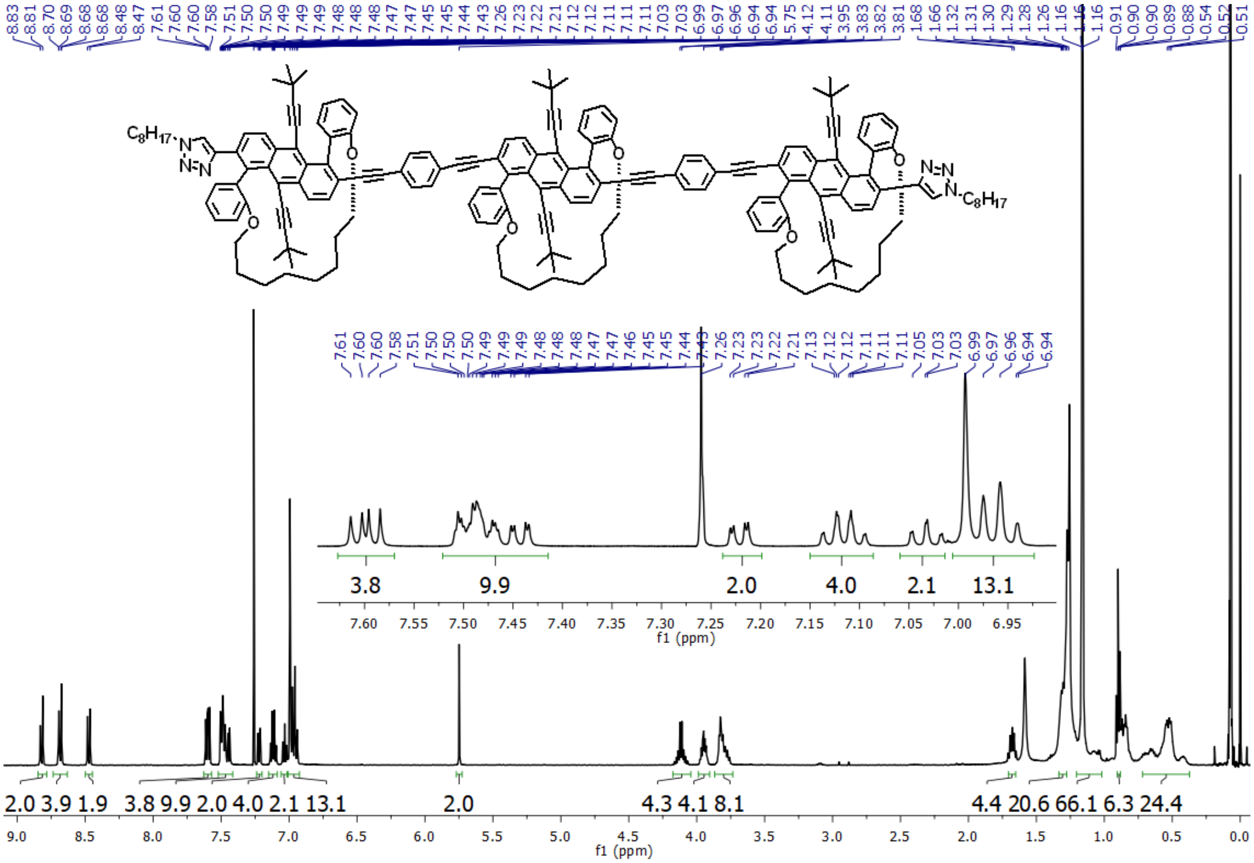

Supplementary Figure 187. ^{1}H NMR (500 MHz) of *P*,*P*,*P*-**3-Ant-C8** in $CDCl_3$, measured at 298 K.

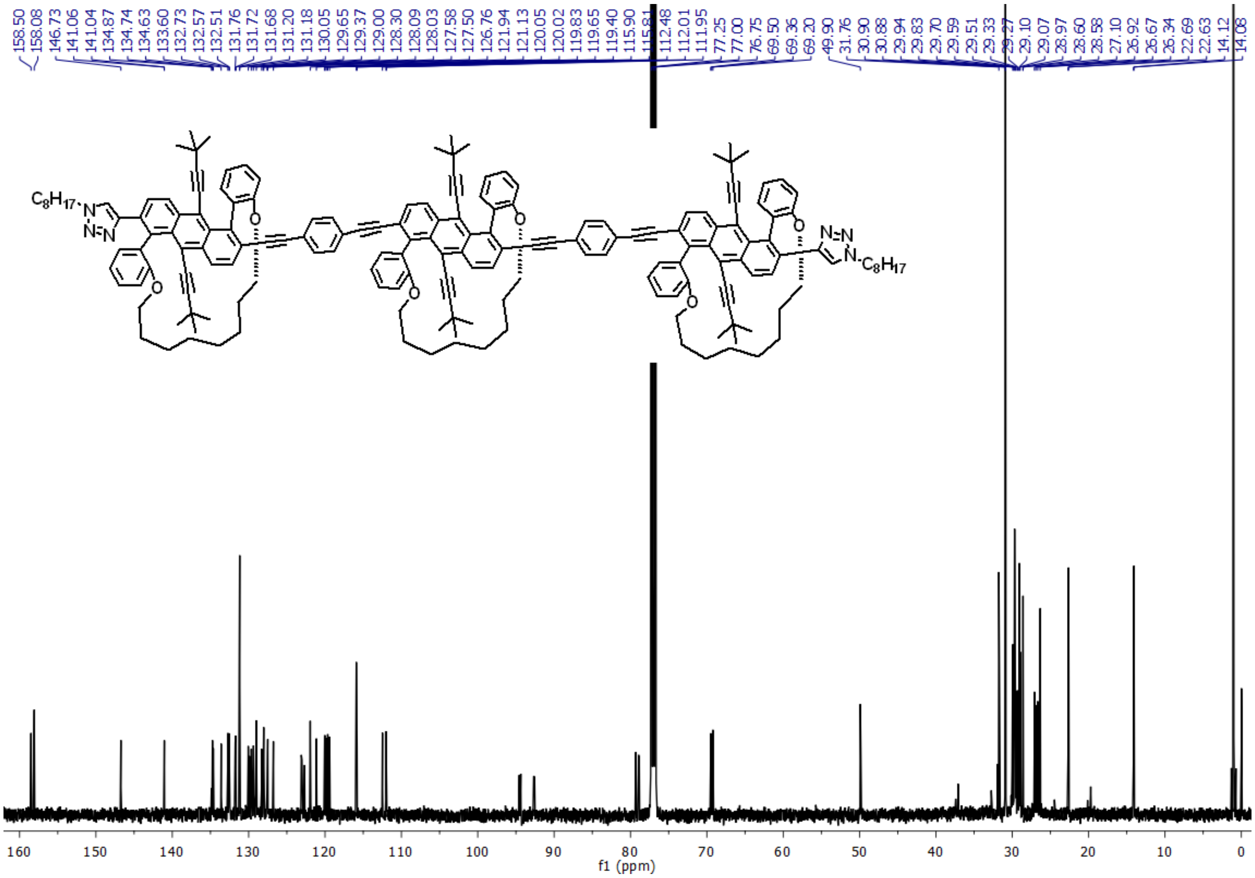

Supplementary Figure 188. ^{13}C NMR (126 MHz) of *P*,*P*,*P*-**3-Ant-C8** in $CDCl_3$, measured at 298 K

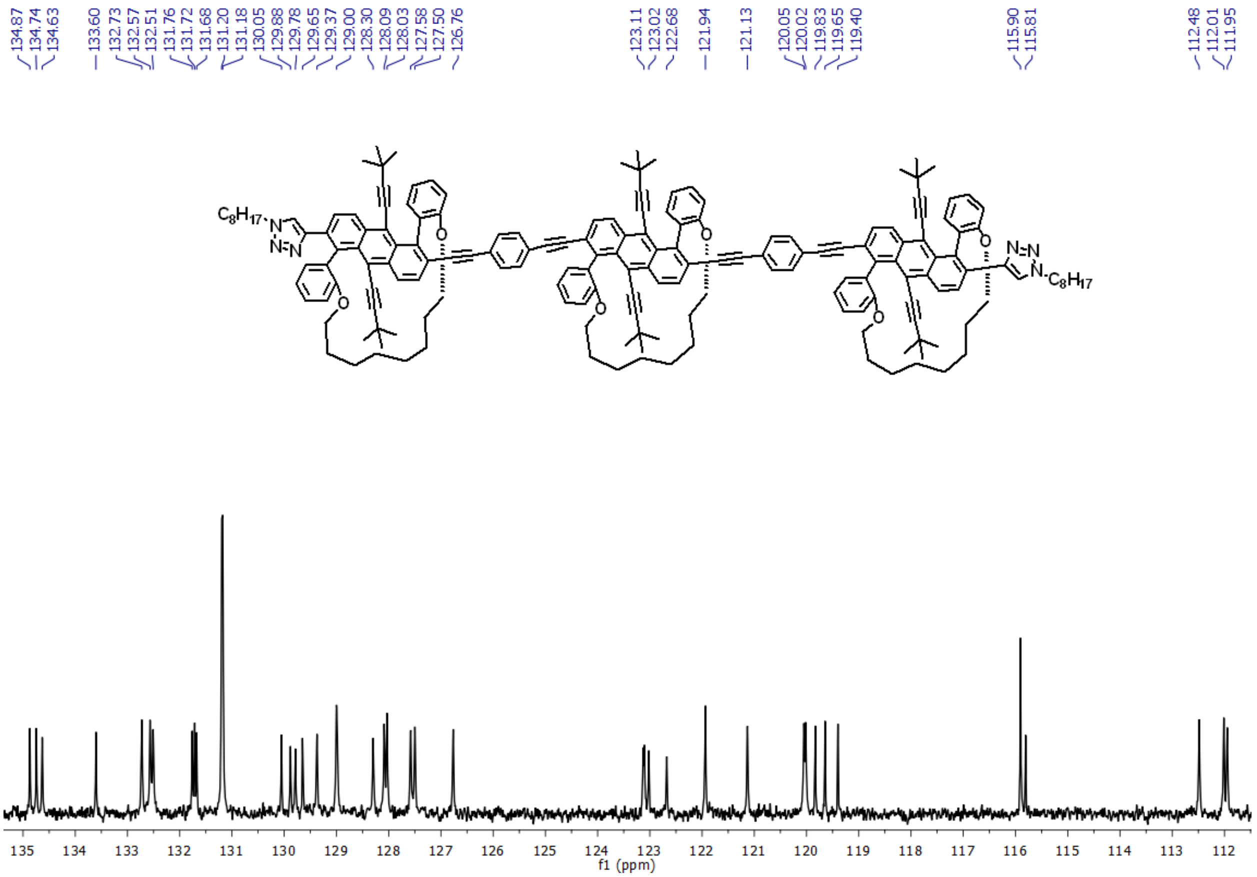

Supplementary Figure 189. ^{13}C NMR (126 MHz) of *P*,*P*,*P*-**3-Ant-C8** in $CDCl_3$, measured at 298 K (expansion in aromatic region).

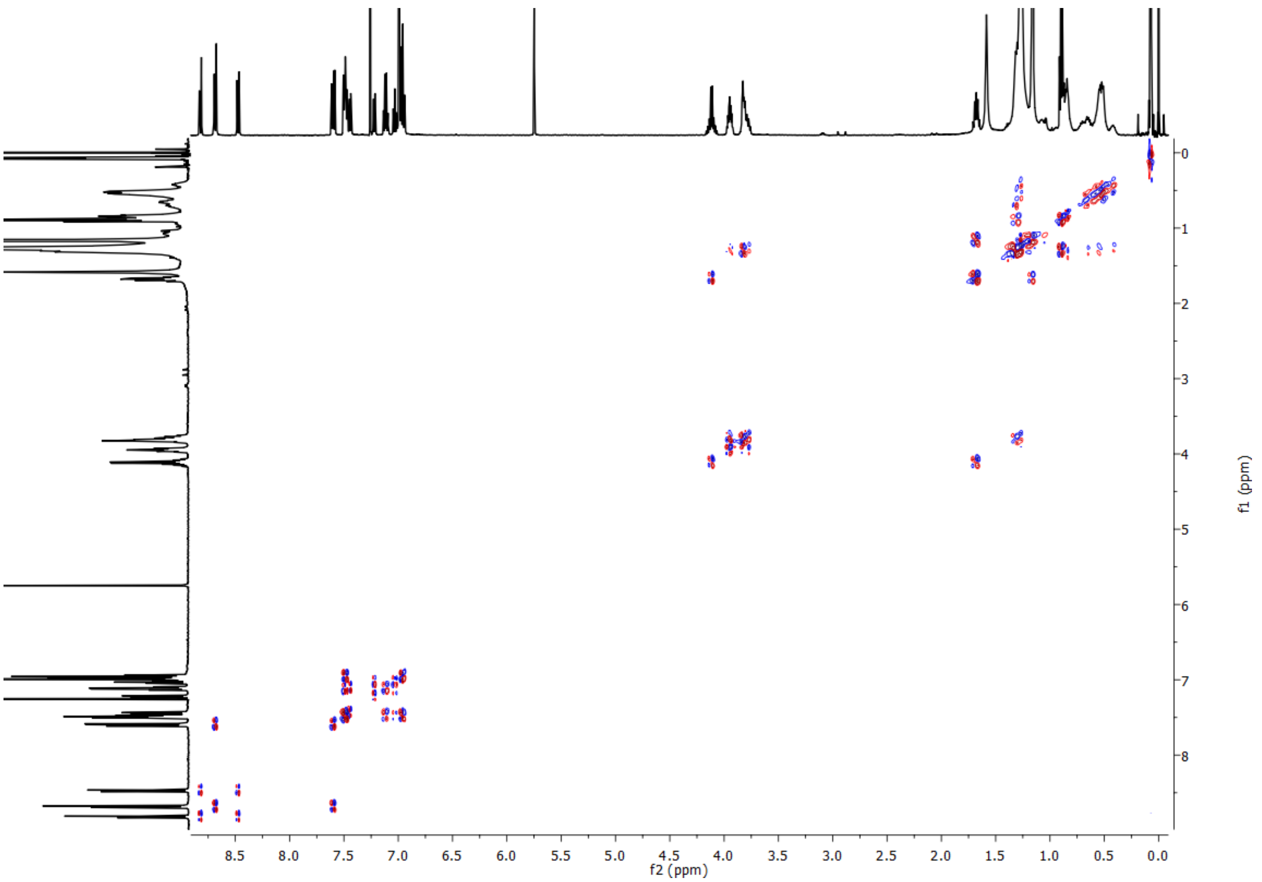

Supplementary Figure 190. COSY NMR (500 MHz) of *P*,*P*,*P*-**3-Ant-C8** in $CDCl_3$, measured at 298 K

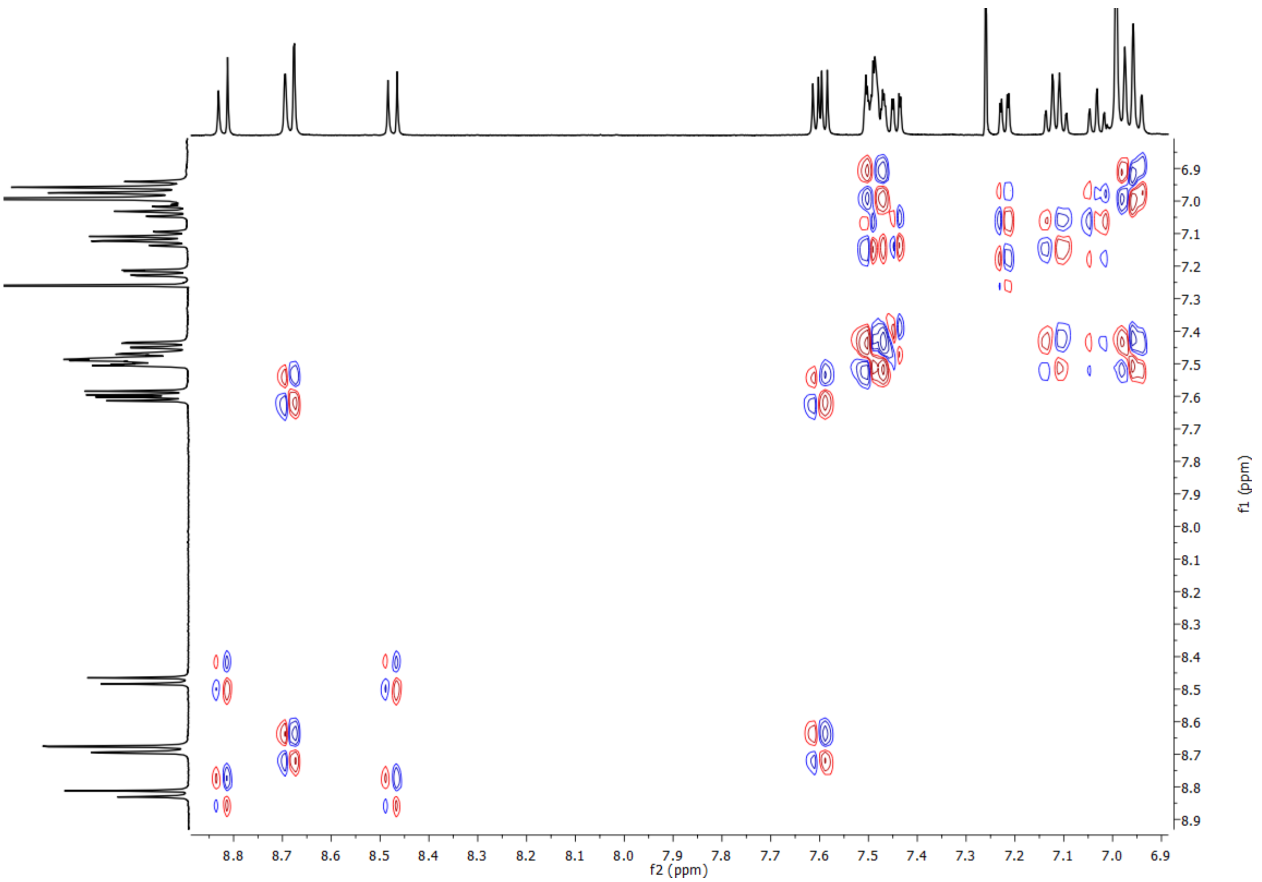

Supplementary Figure 191. COSY NMR (500 MHz) of *P*,*P*,*P*-**3-Ant-C8** in $CDCl_3$, measured at 298 K (expansion in aromatic region).

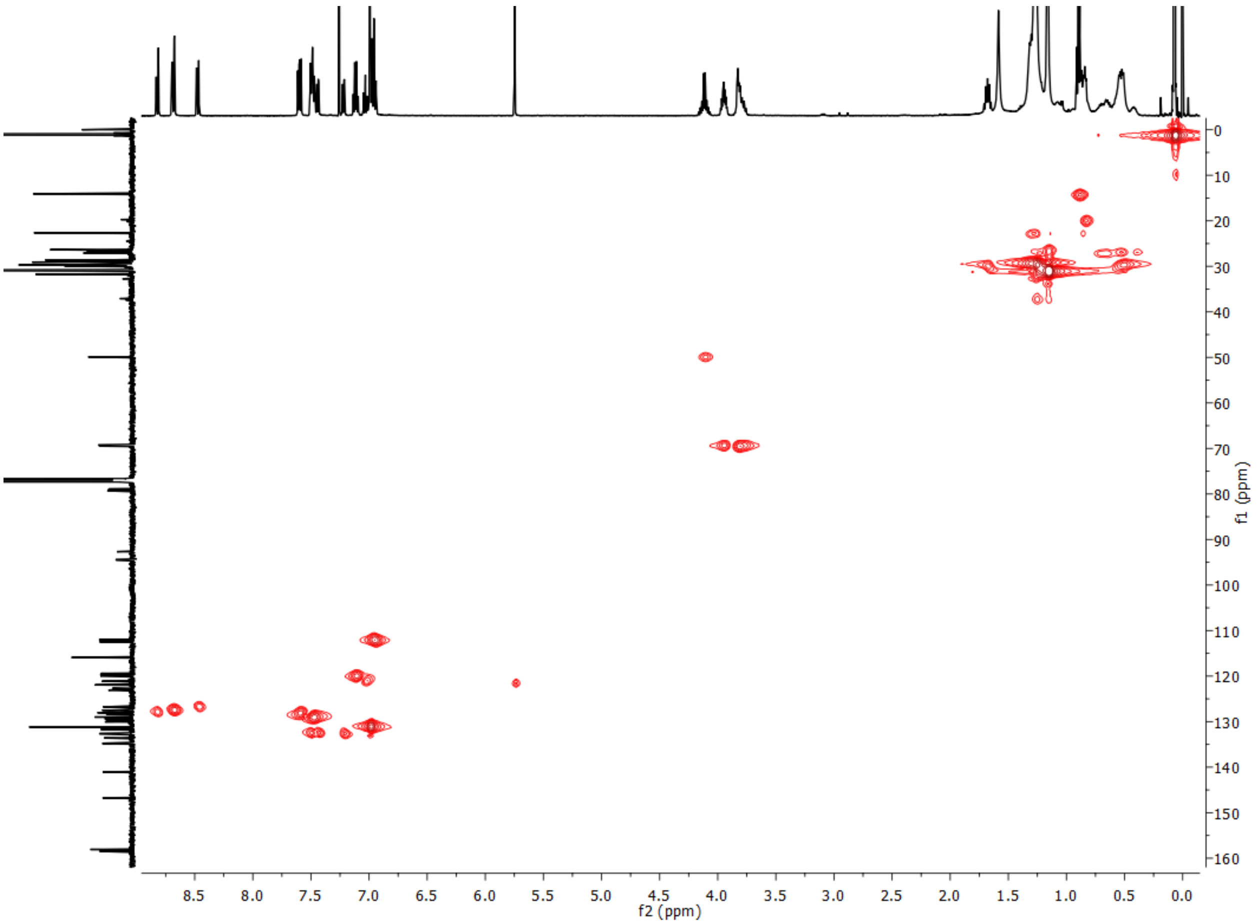

Supplementary Figure 192. HSQC NMR (500 MHz) of *P*,*P*,*P*-**3-Ant-C8** in $CDCl_3$, measured at 298 K

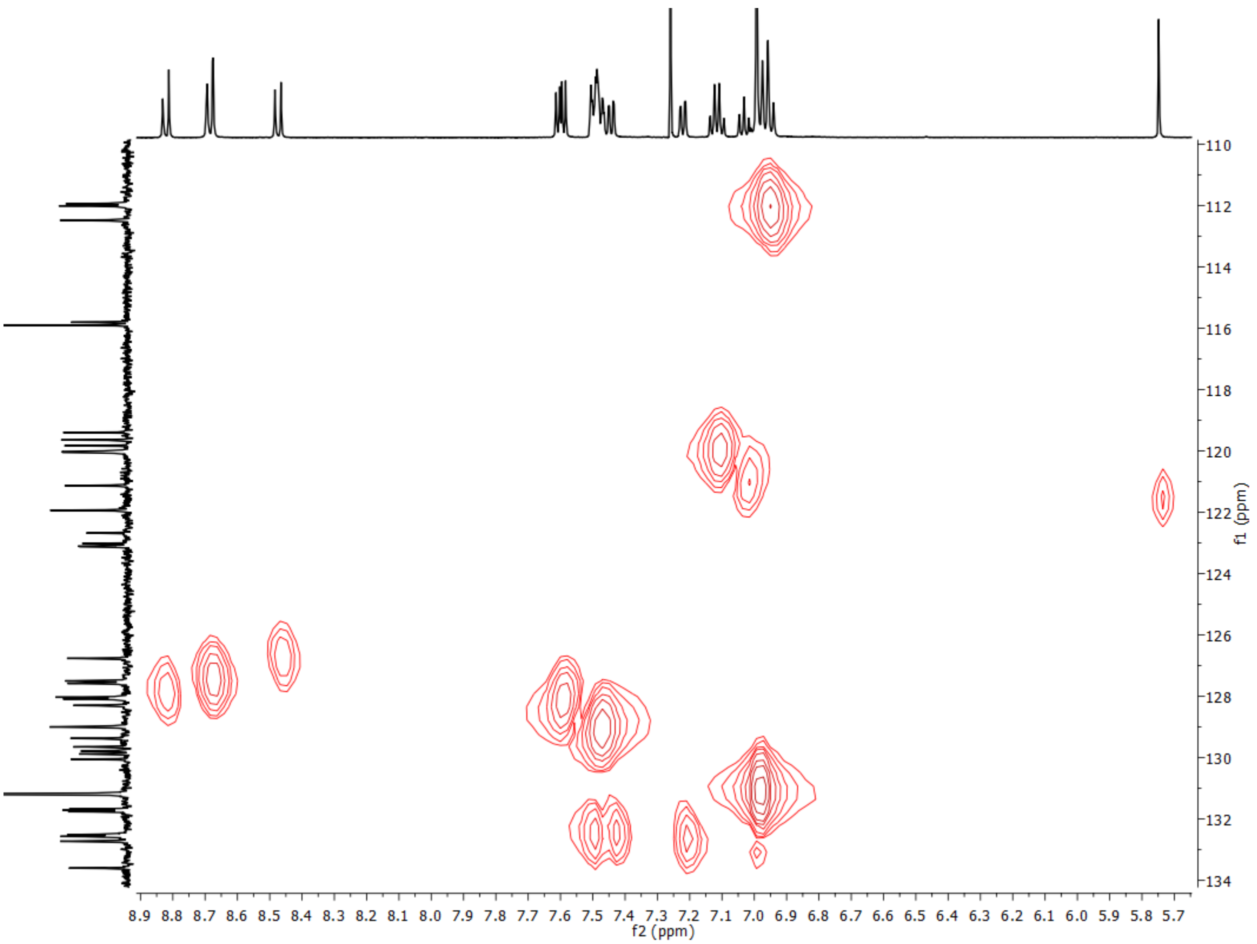

Supplementary Figure 193. HSQC NMR (500 MHz) of *P*,*P*,*P*-**3-Ant-C8** in $CDCl_3$, measured at 298 K (expansion in aromatic region).

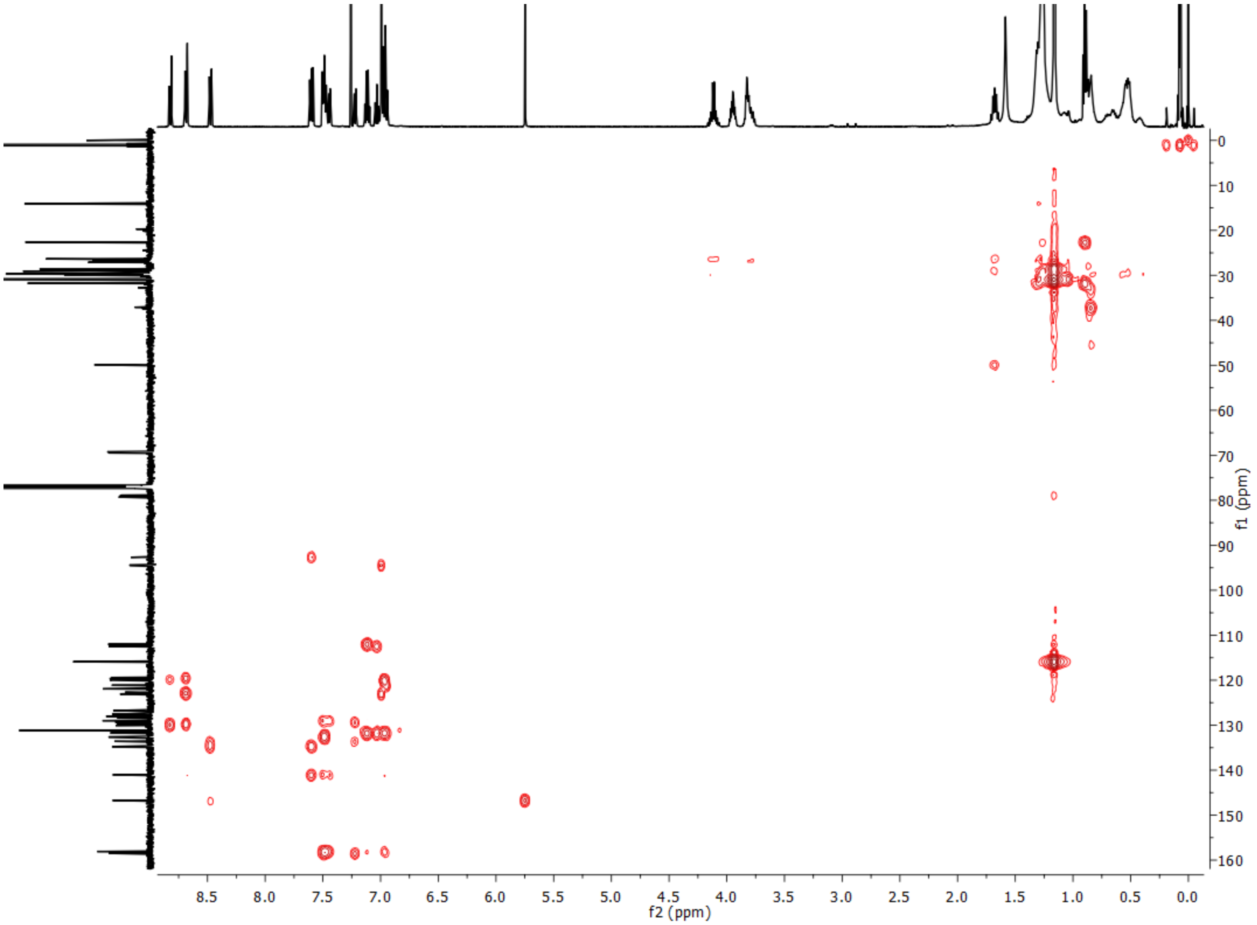

Supplementary Figure 194. HMBC NMR (500 MHz) of *P*,*P*,*P*-**3-Ant-C8** in $CDCl_3$, measured at 298 K.

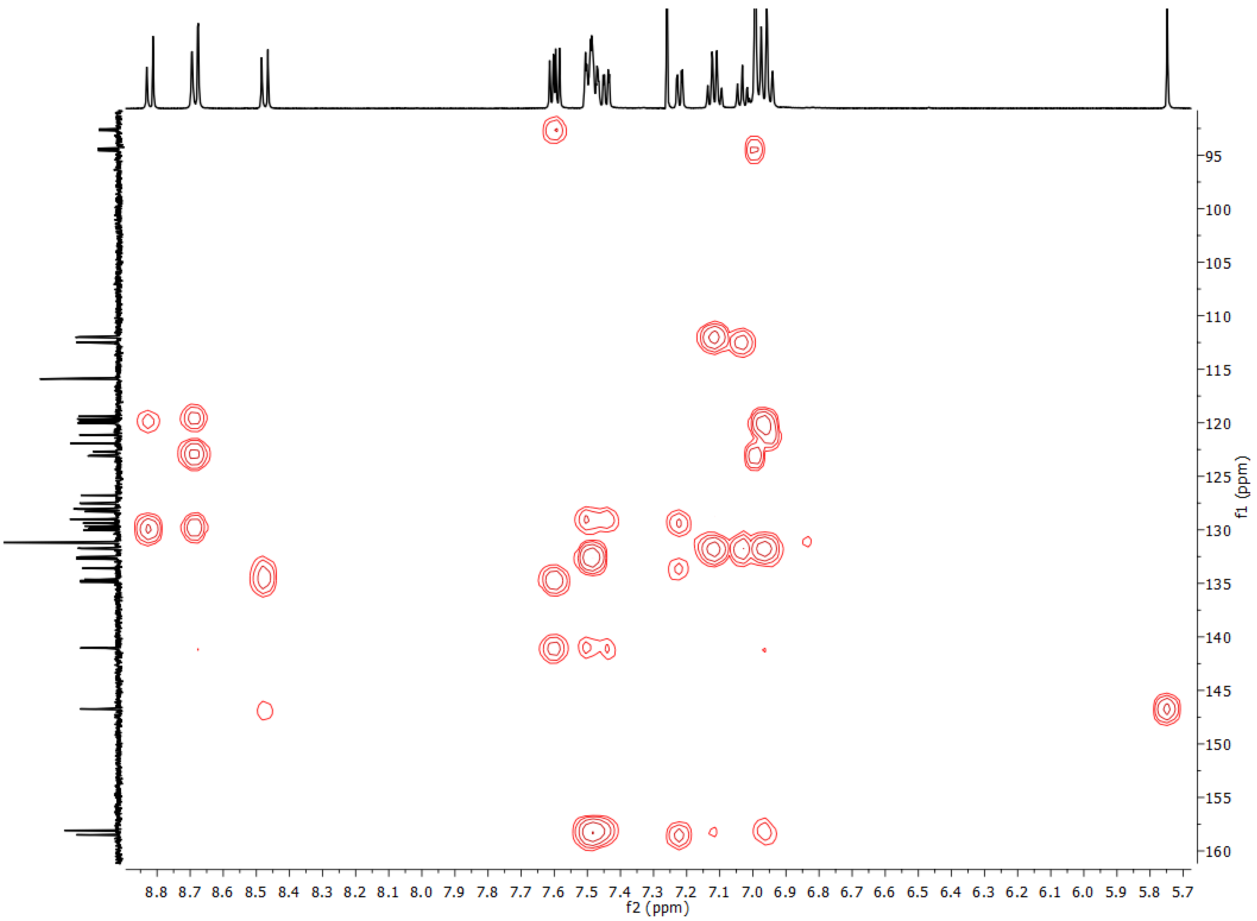

Supplementary Figure 195. HMBC NMR (500 MHz) of *P*,*P*,*P*-**3-Ant-C8** in $CDCl_3$, measured at 298 K (expansion in aromatic region).

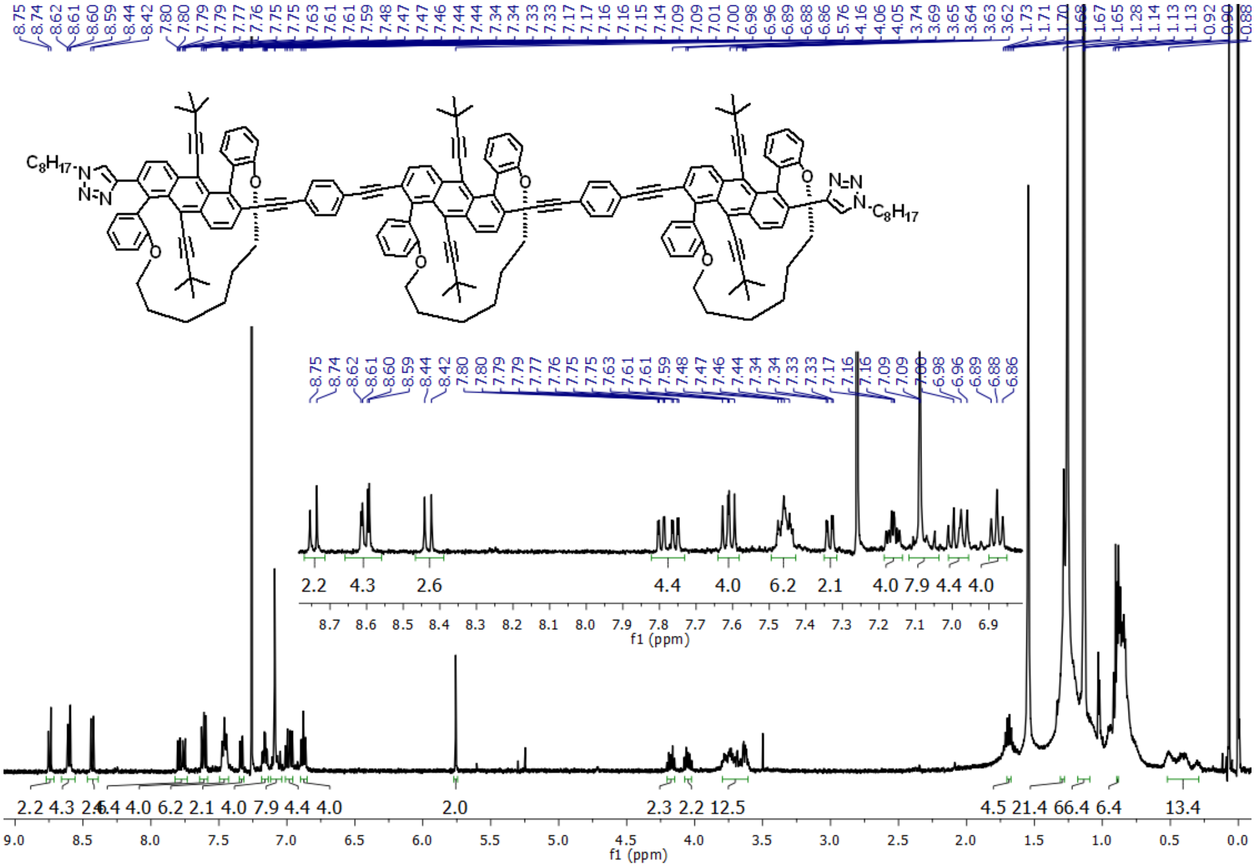

Supplementary Figure 196. ^{1}H NMR (500 MHz) of *P*,*P*,*P*-**3-Ant-C6** in $CDCl_3$, measured at 298 K.

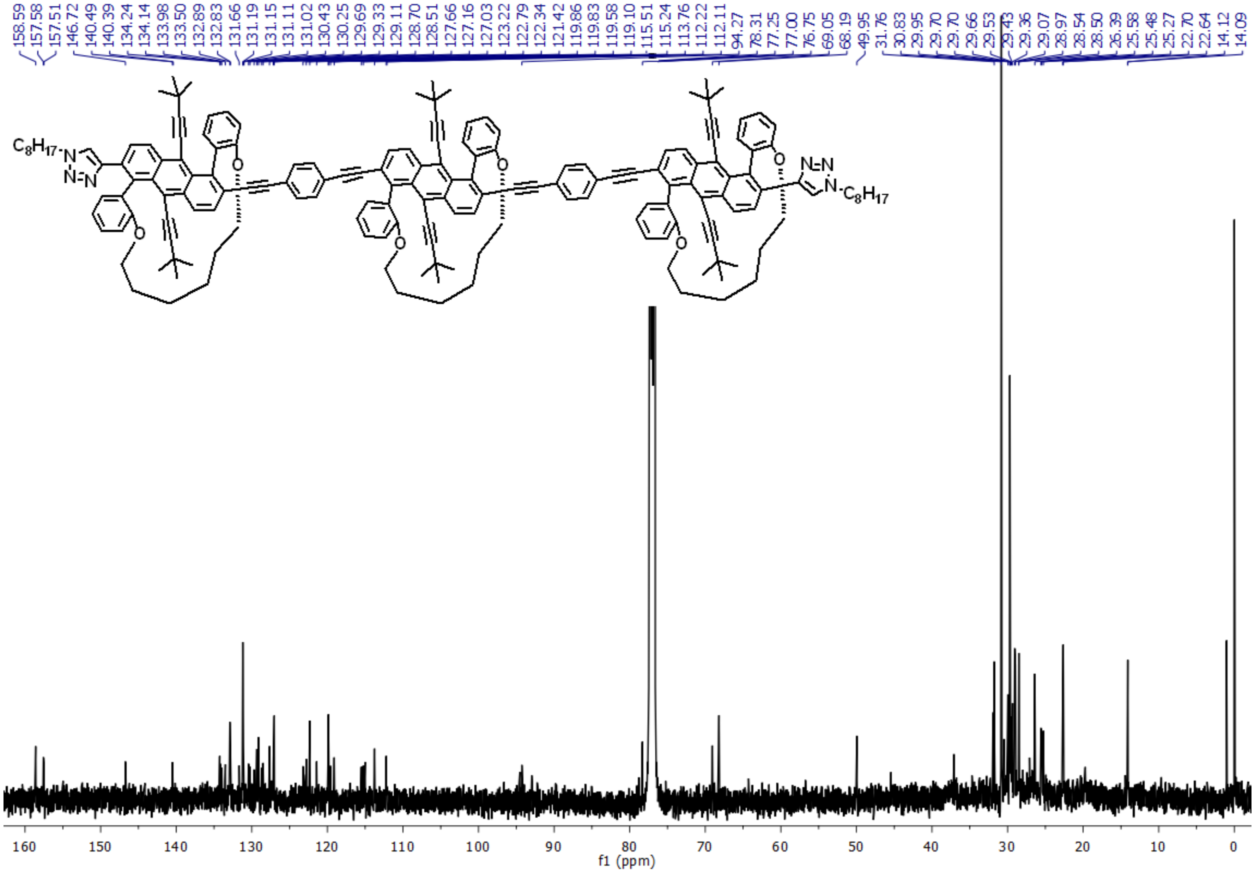

Supplementary Figure 197. ^{13}C NMR (126 MHz) of *P*,*P*,*P*-**3-Ant-C6** in $CDCl_3$, measured at 298 K.

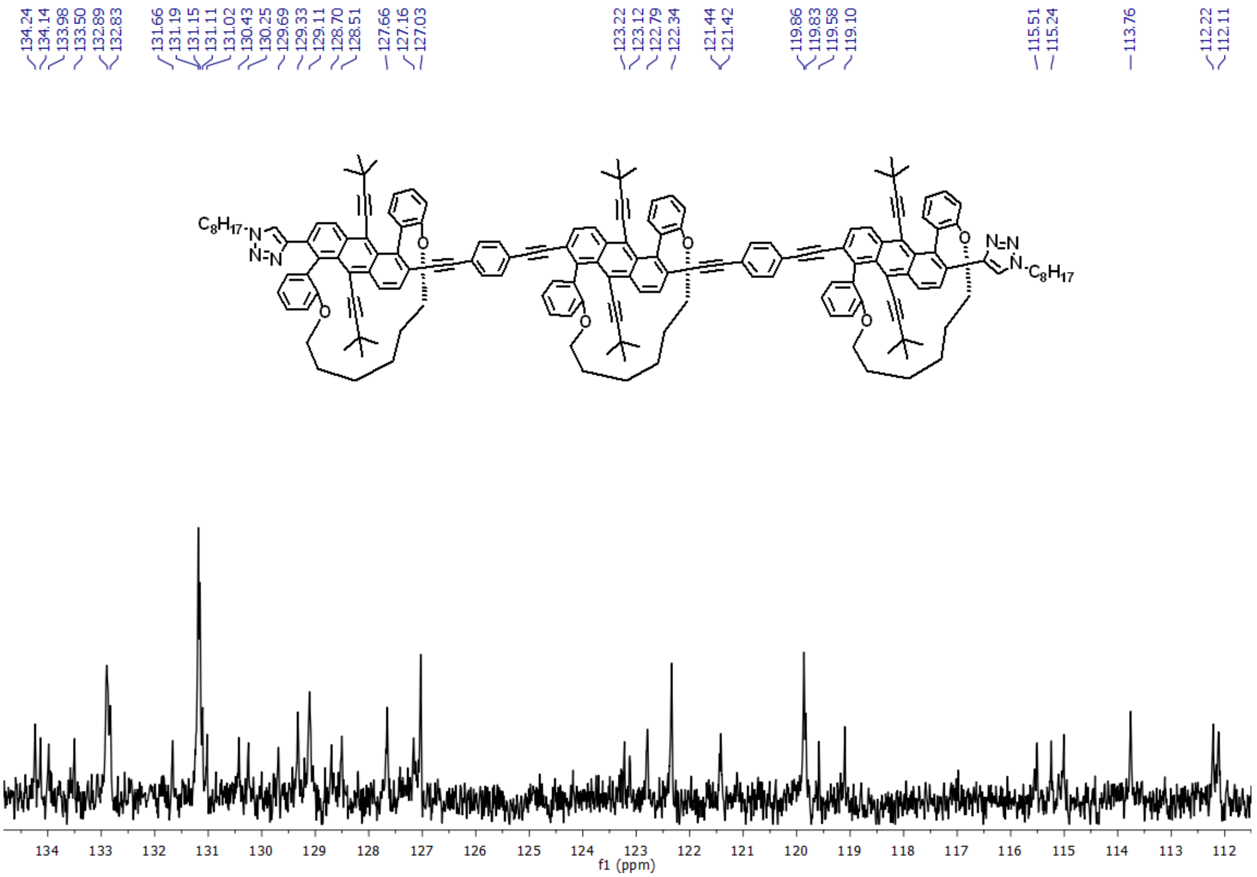

Supplementary Figure 198. ^{13}C NMR (126 MHz) of *P*,*P*,*P*-**3-Ant-C6** in $CDCl_3$, measured at 298 K (expansion in aromatic region).

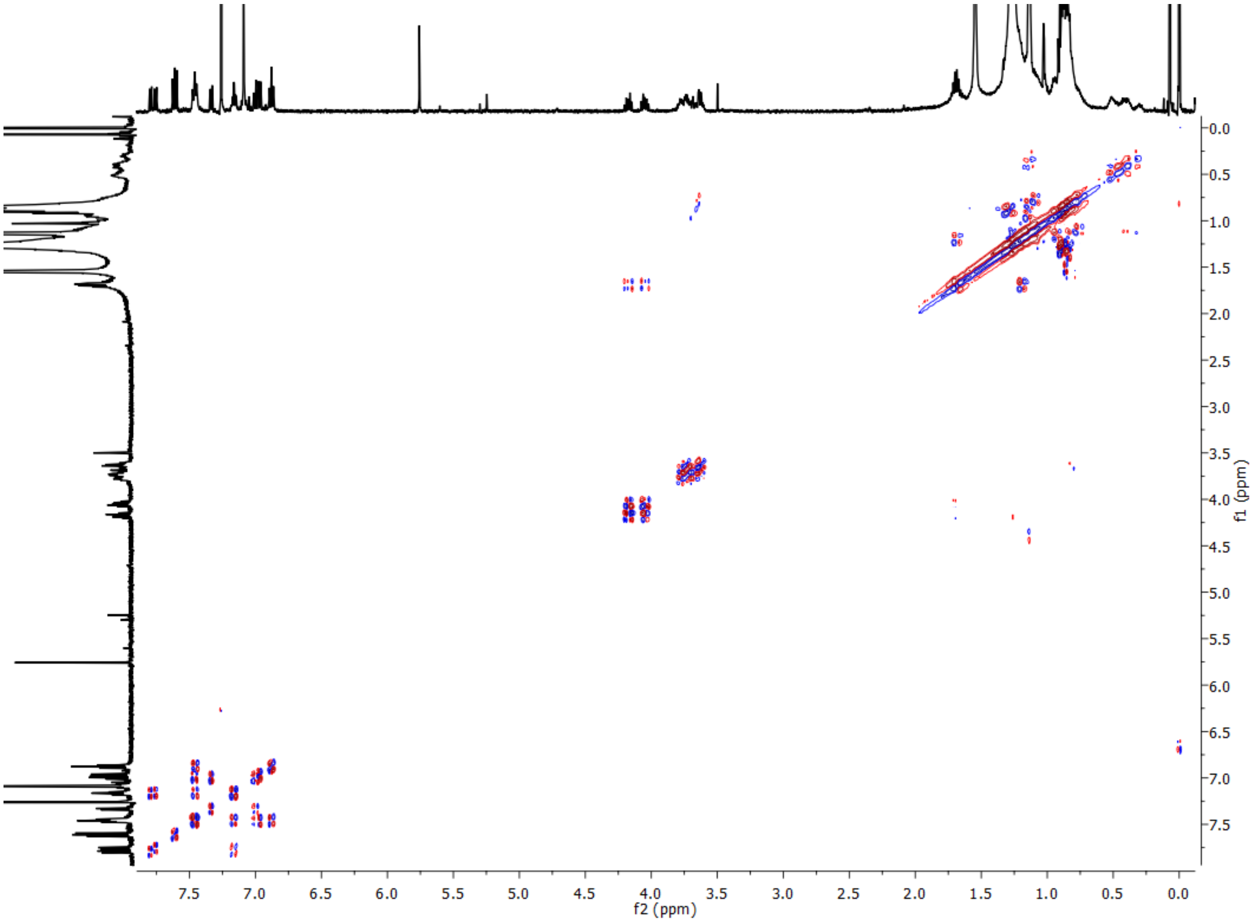

Supplementary Figure 199. COSY NMR (500 MHz) of *P*,*P*,*P*-**3-Ant-C6** in $CDCl_3$, measured at 298 K.

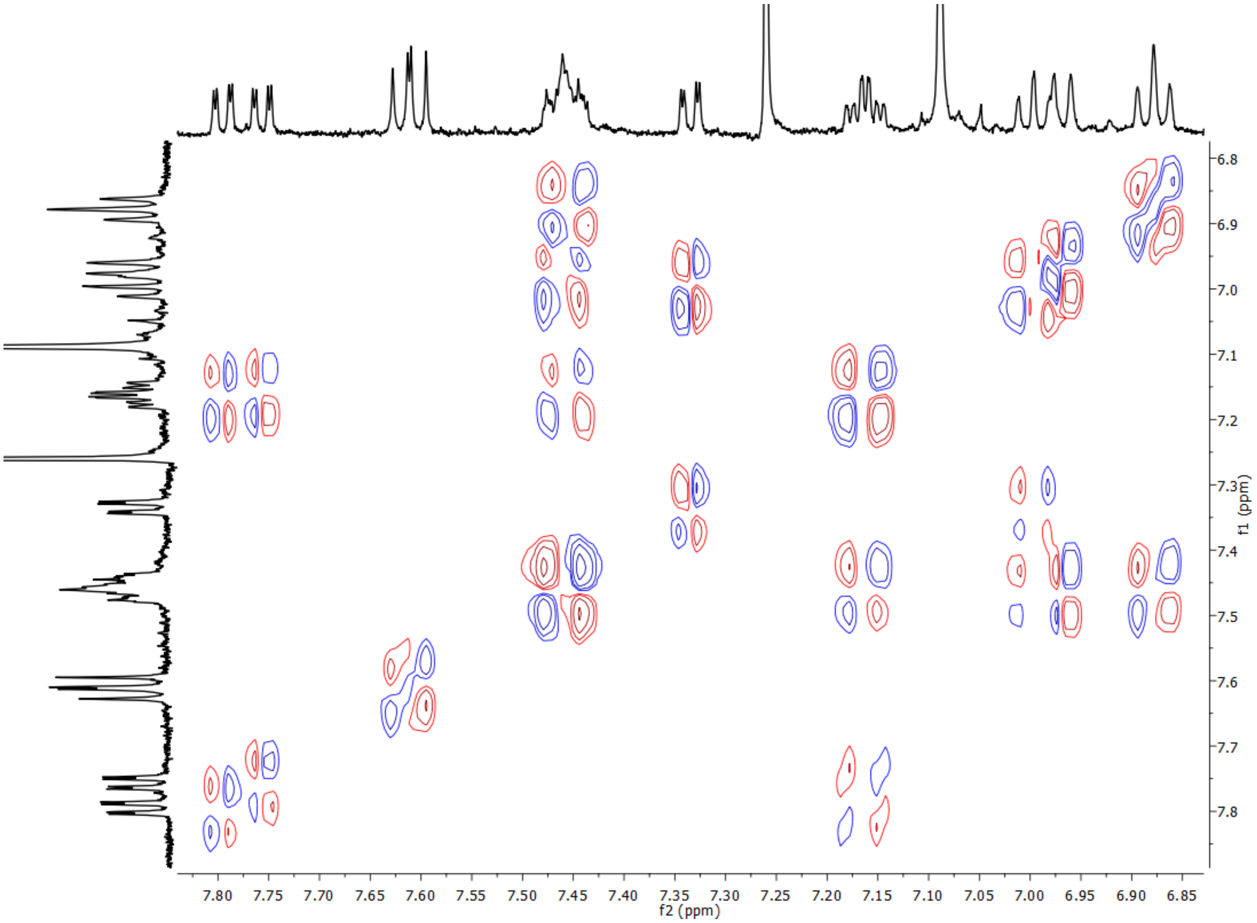

Supplementary Figure 200. COSY NMR (500 MHz) of *P*,*P*,*P*-**3-Ant-C6** in $CDCl_3$, measured at 298 K (expansion in aromatic region).

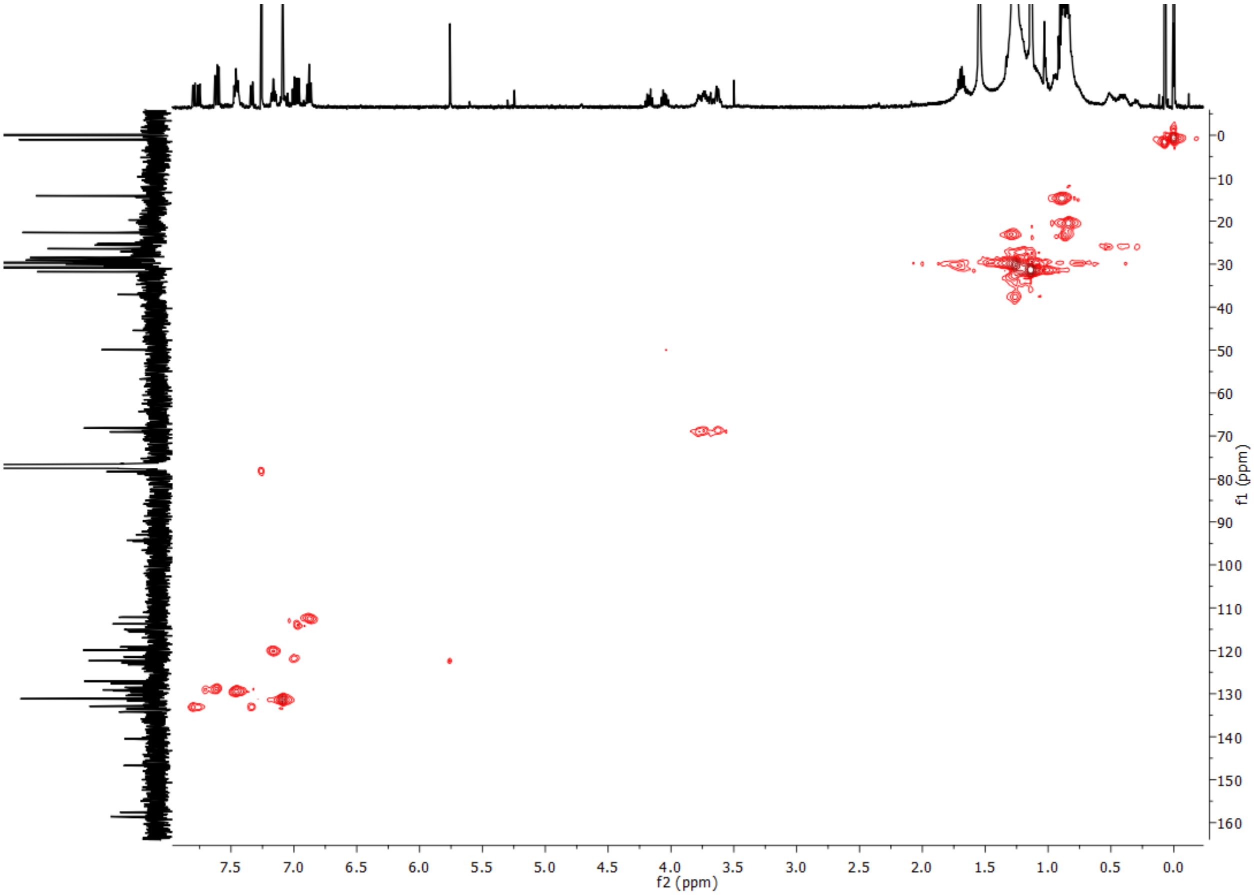

Supplementary Figure 201. HSQC NMR (500 MHz) of *P*,*P*,*P*-**3-Ant-C6** in $CDCl_3$, measured at 298 K.

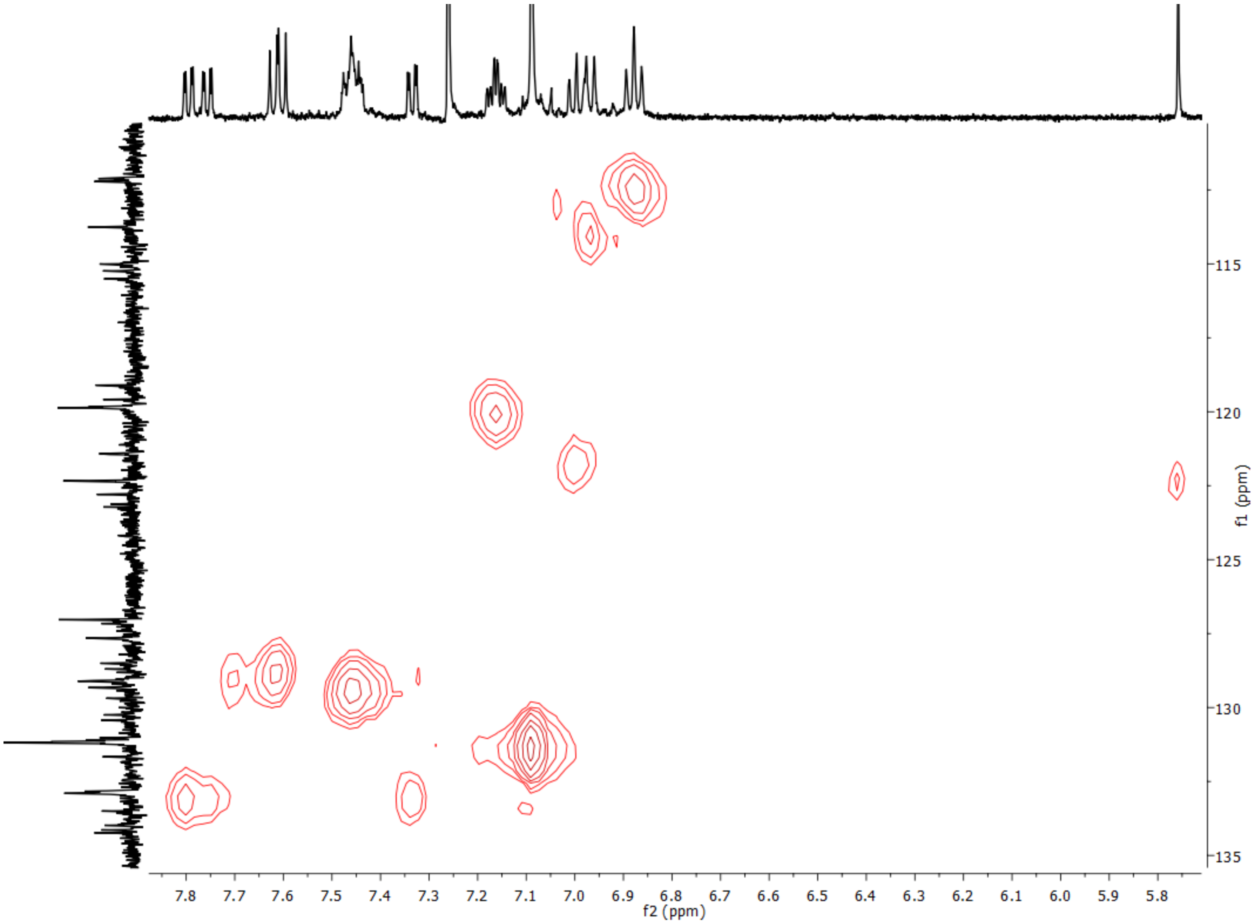

Supplementary Figure 202. HSQC NMR (500 MHz) of *P*,*P*,*P*-**3-Ant-C6** in $CDCl_3$, measured at 298 K (expansion in aromatic region).

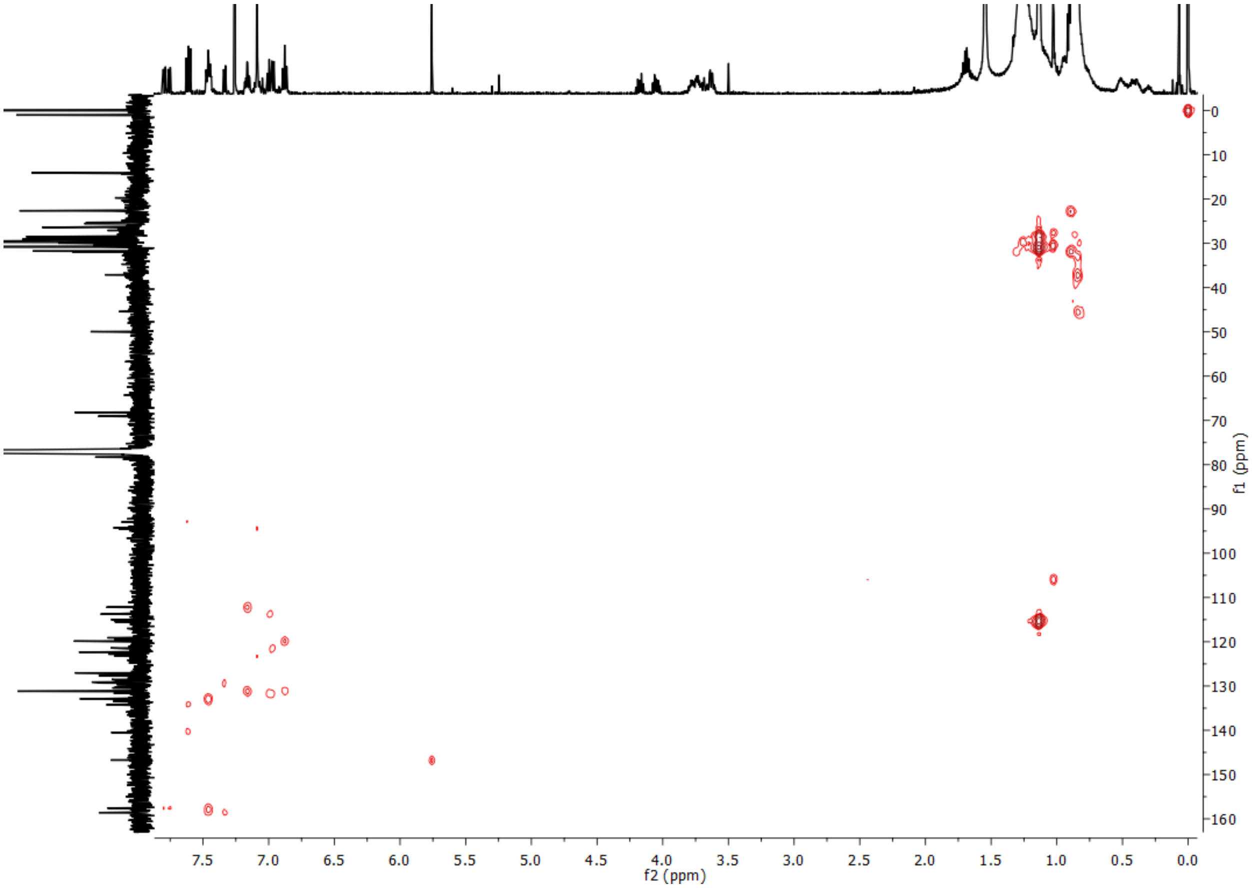

Supplementary Figure 203. HMBC NMR (500 MHz) of *P*,*P*,*P*-**3-Ant-C6** in $CDCl_3$, measured at 298 K.

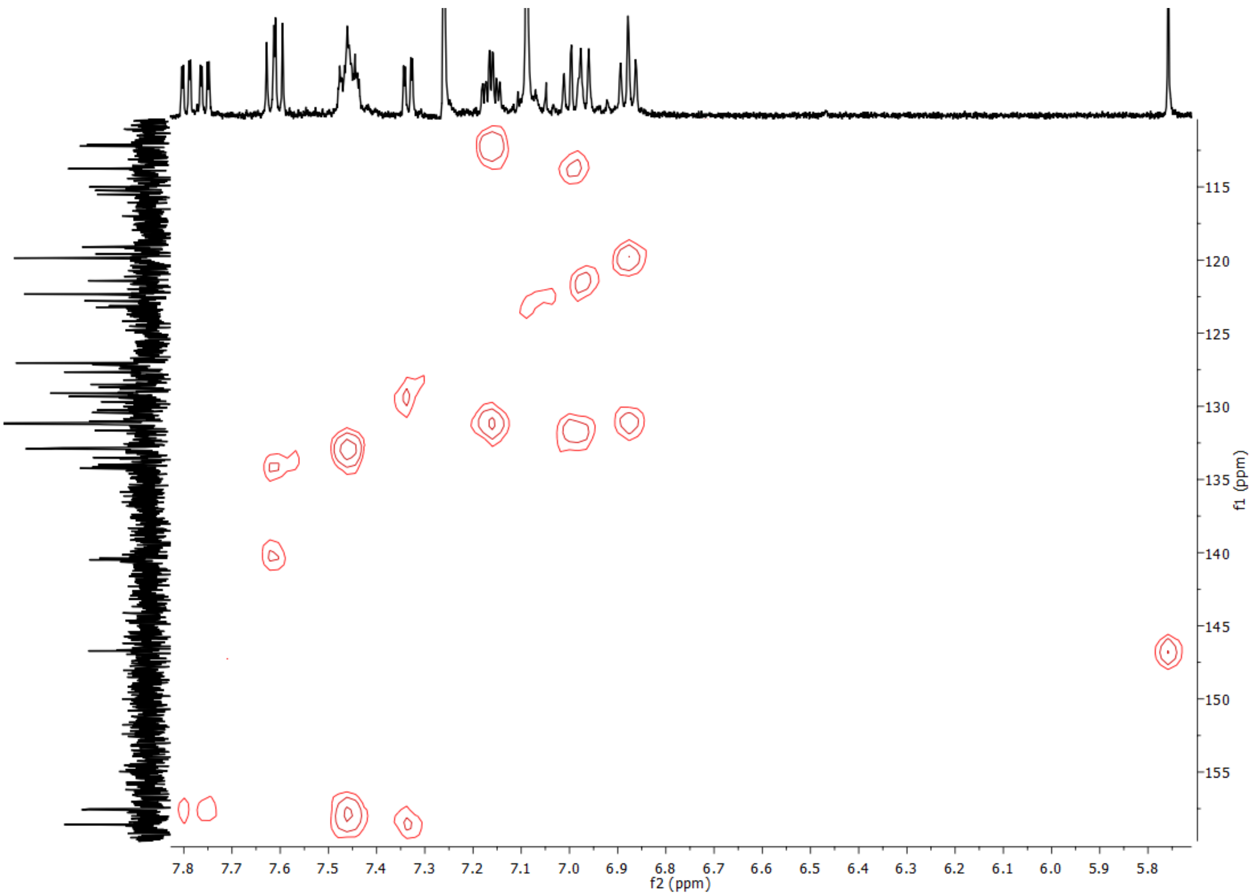

Supplementary Figure 204. HMBC NMR (500 MHz) of *P*,*P*,*P*-**3-Ant-C6** in $CDCl_3$, measured at 298 K (expansion in aromatic region).

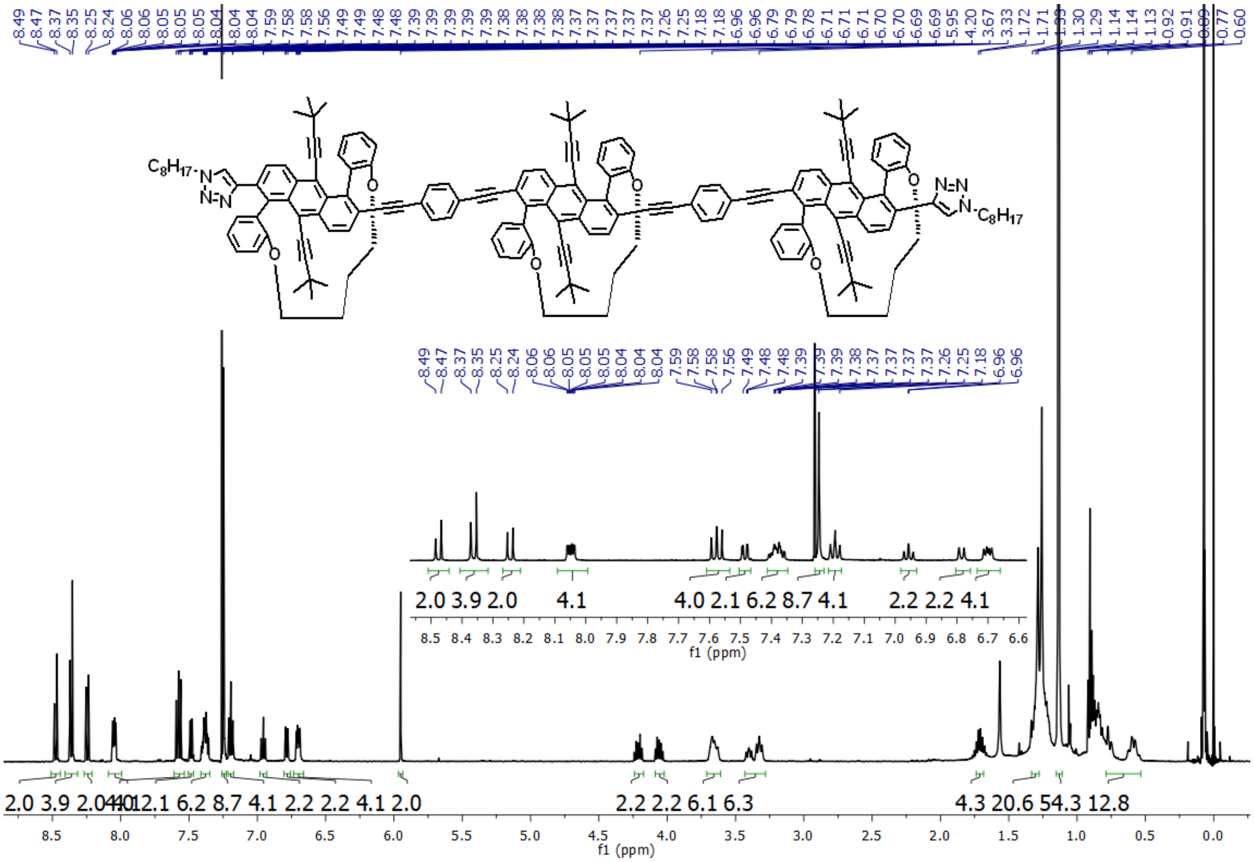

Supplementary Figure 205. ^{1}H NMR (500 MHz) of *P*,*P*,*P*-**3-Ant-C4** in $CDCl_3$, measured at 298 K.

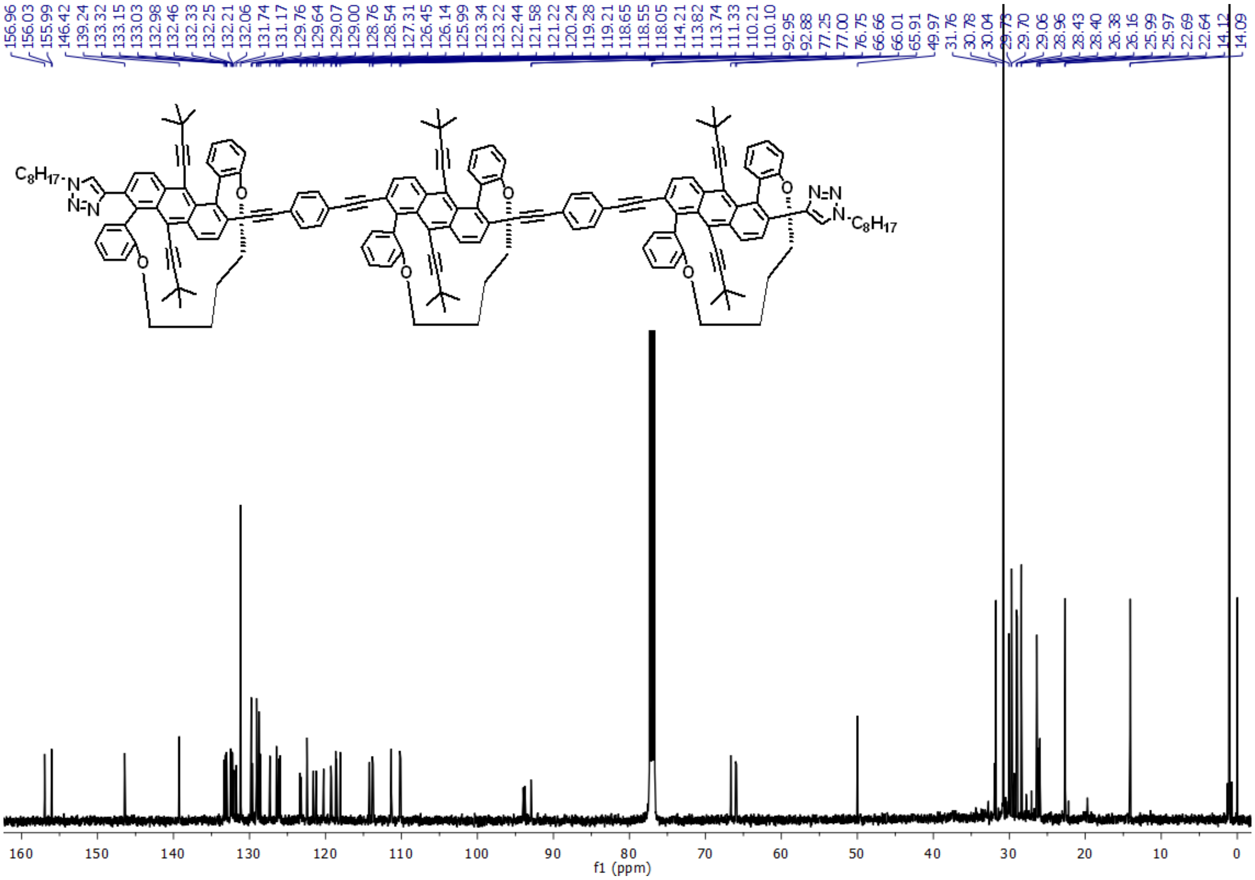

Supplementary Figure 206. ^{13}C NMR (126 MHz) of *P*,*P*,*P*-**3-Ant-C4** in $CDCl_3$, measured at 298 K.

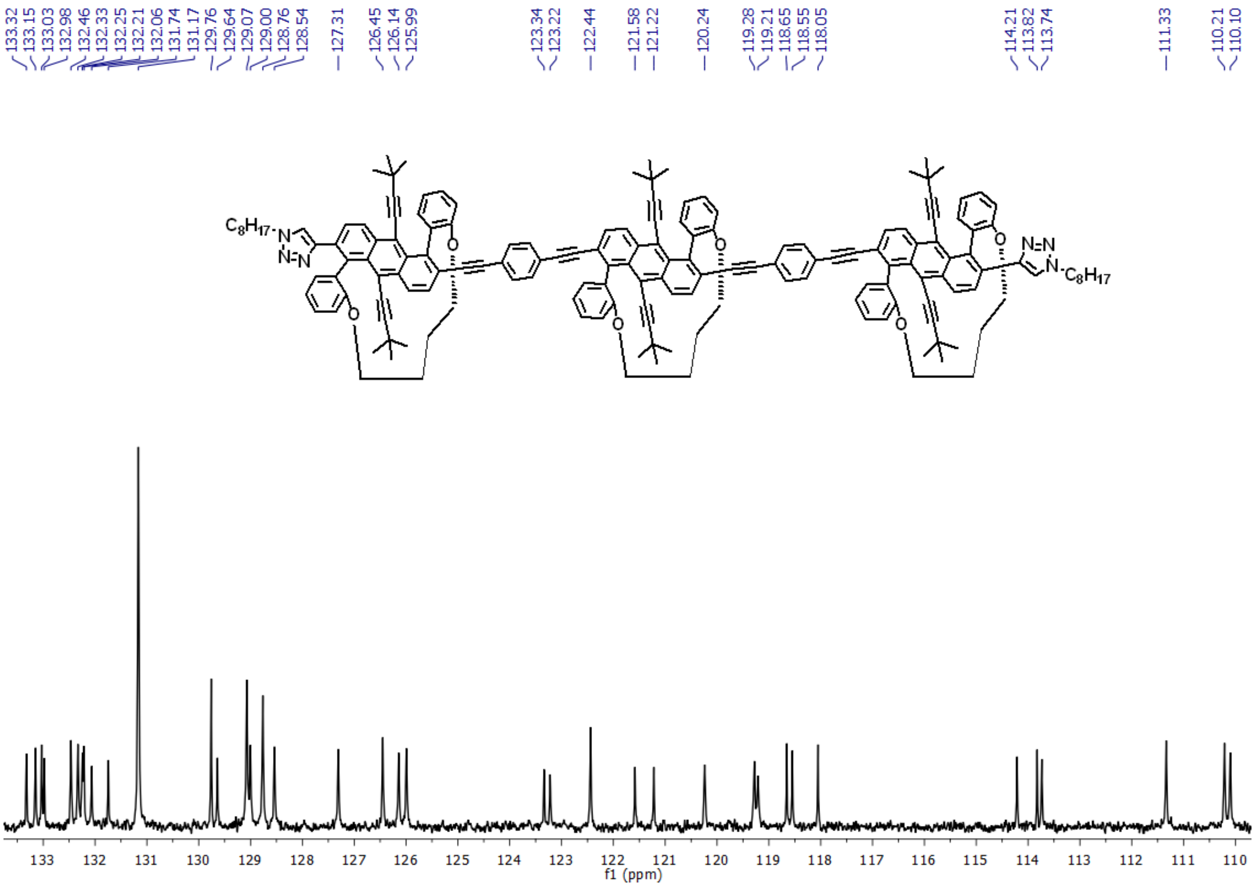

Supplementary Figure 207. ^{13}C NMR (126 MHz) of *P*,*P*,*P*-**3-Ant-C4** in $CDCl_3$, measured at 298 K (expansion in aromatic region).

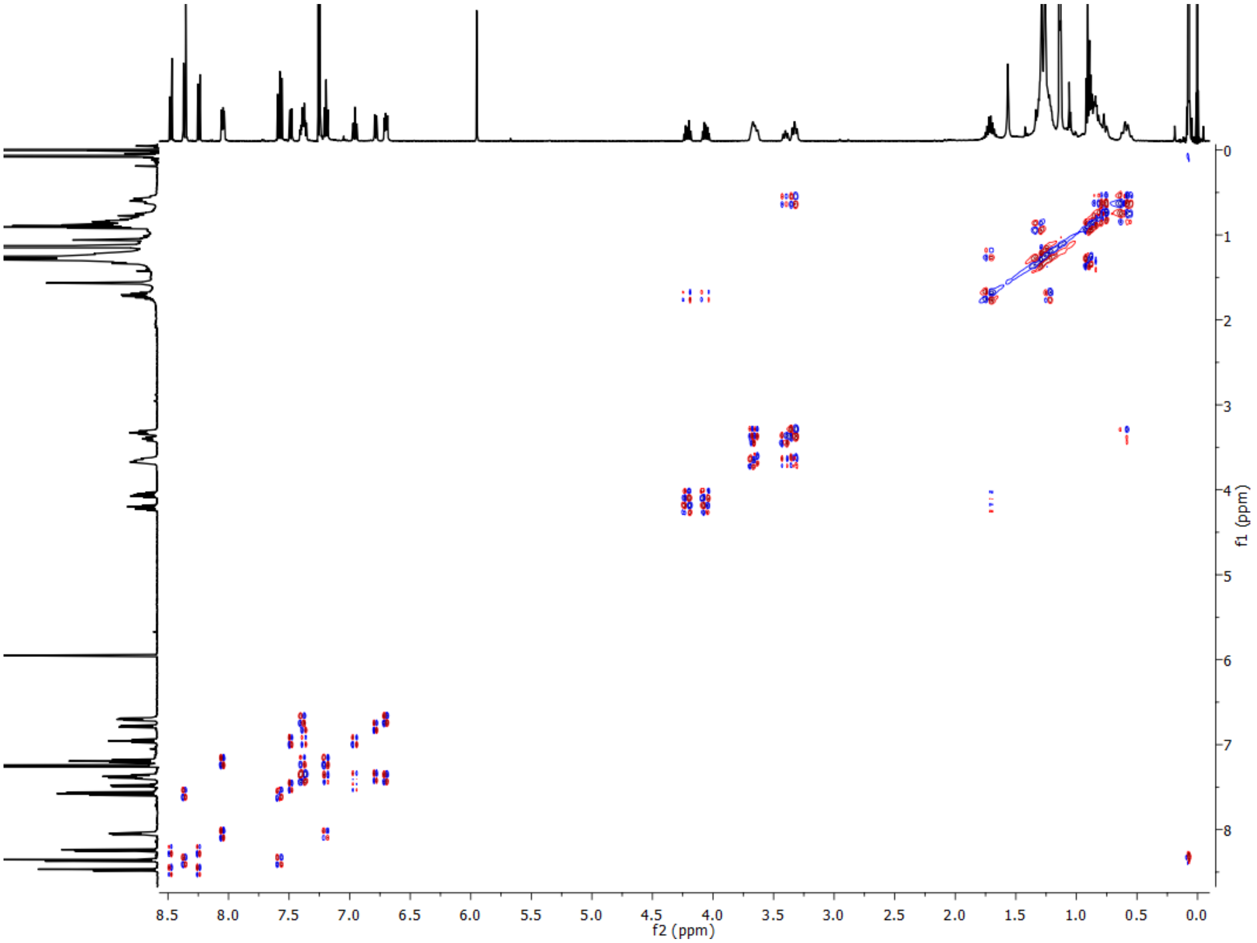

Supplementary Figure 208. COSY NMR (500 MHz) of *P*,*P*,*P*-**3-Ant-C4** in $CDCl_3$, measured at 298 K.

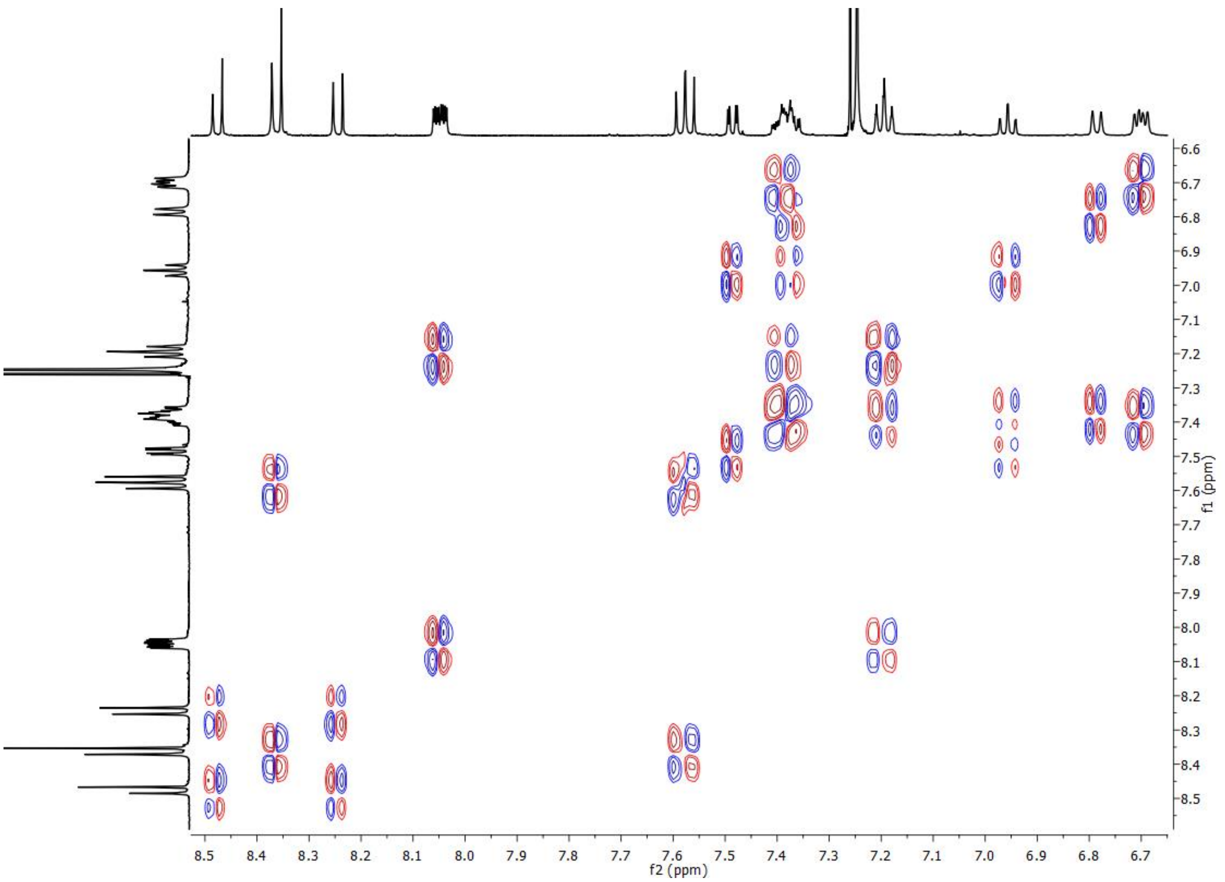

Supplementary Figure 209. COSY NMR (500 MHz) of *P*,*P*,*P*-**3-Ant-C4** in $CDCl_3$, measured at 298 K (expansion in aromatic region).

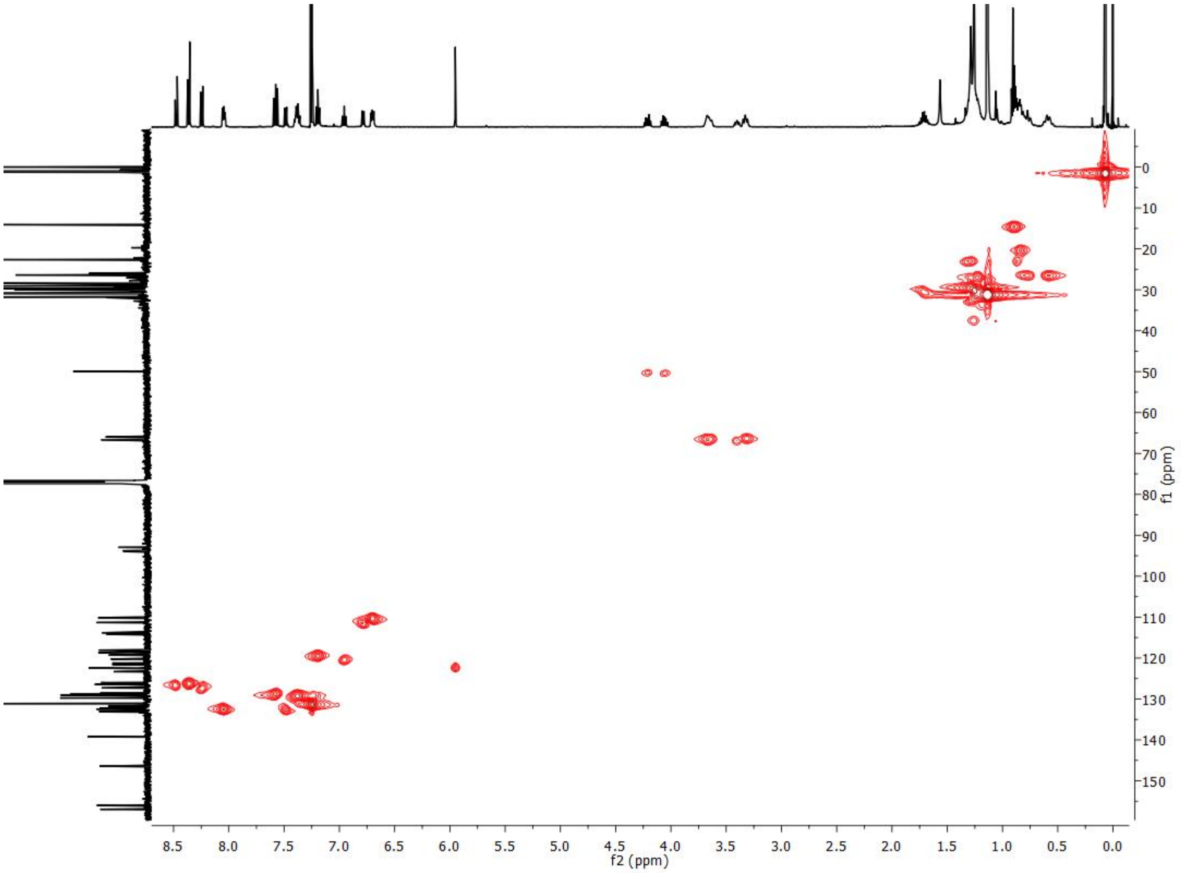

Supplementary Figure 210. HSQC NMR (500 MHz) of *P*,*P*,*P*-**3-Ant-C4** in $CDCl_3$, measured at 298 K.

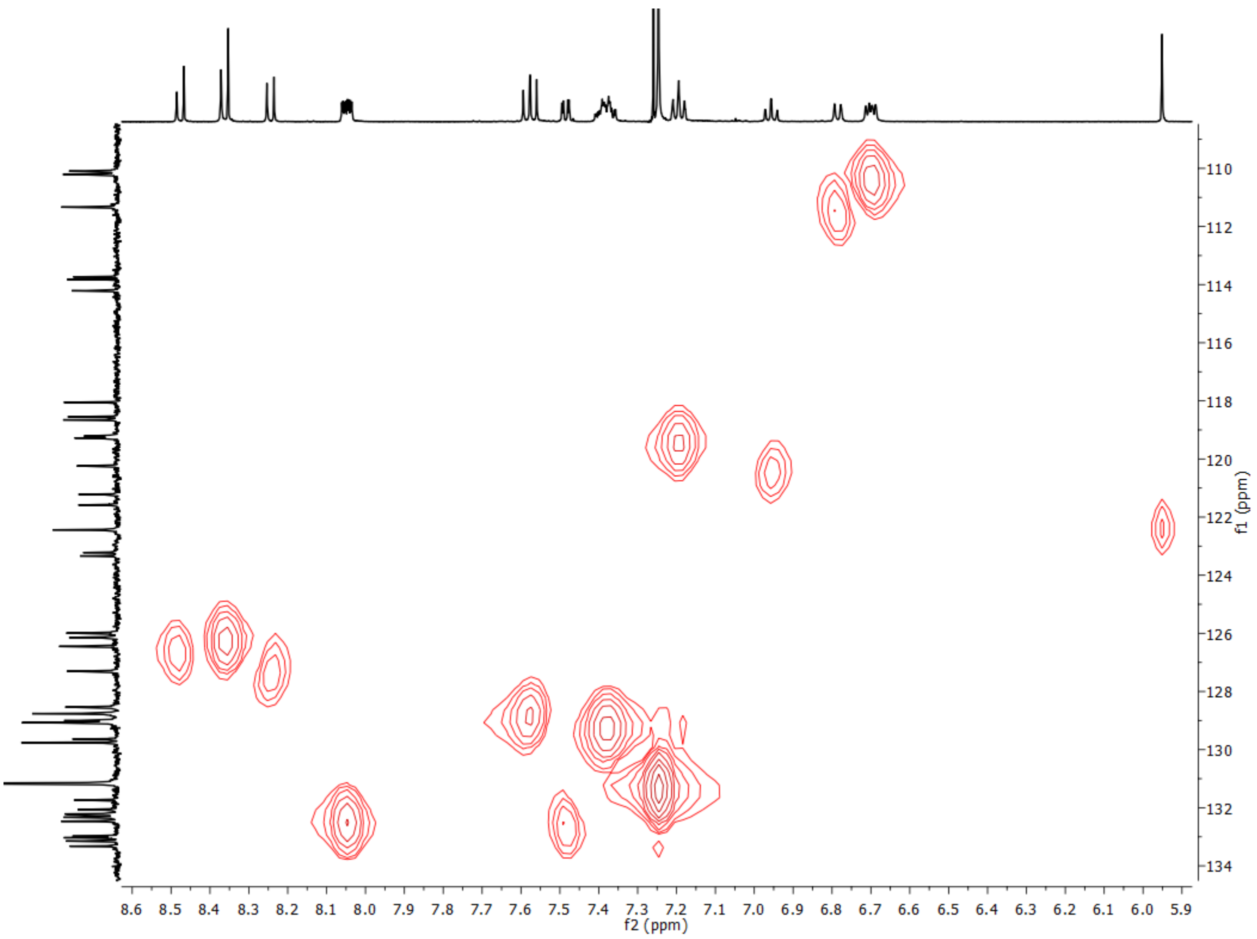

Supplementary Figure 211. HSQC NMR (500 MHz) of *P*,*P*,*P*-**3-Ant-C4** in $CDCl_3$, measured at 298 K (expansion in aromatic region).

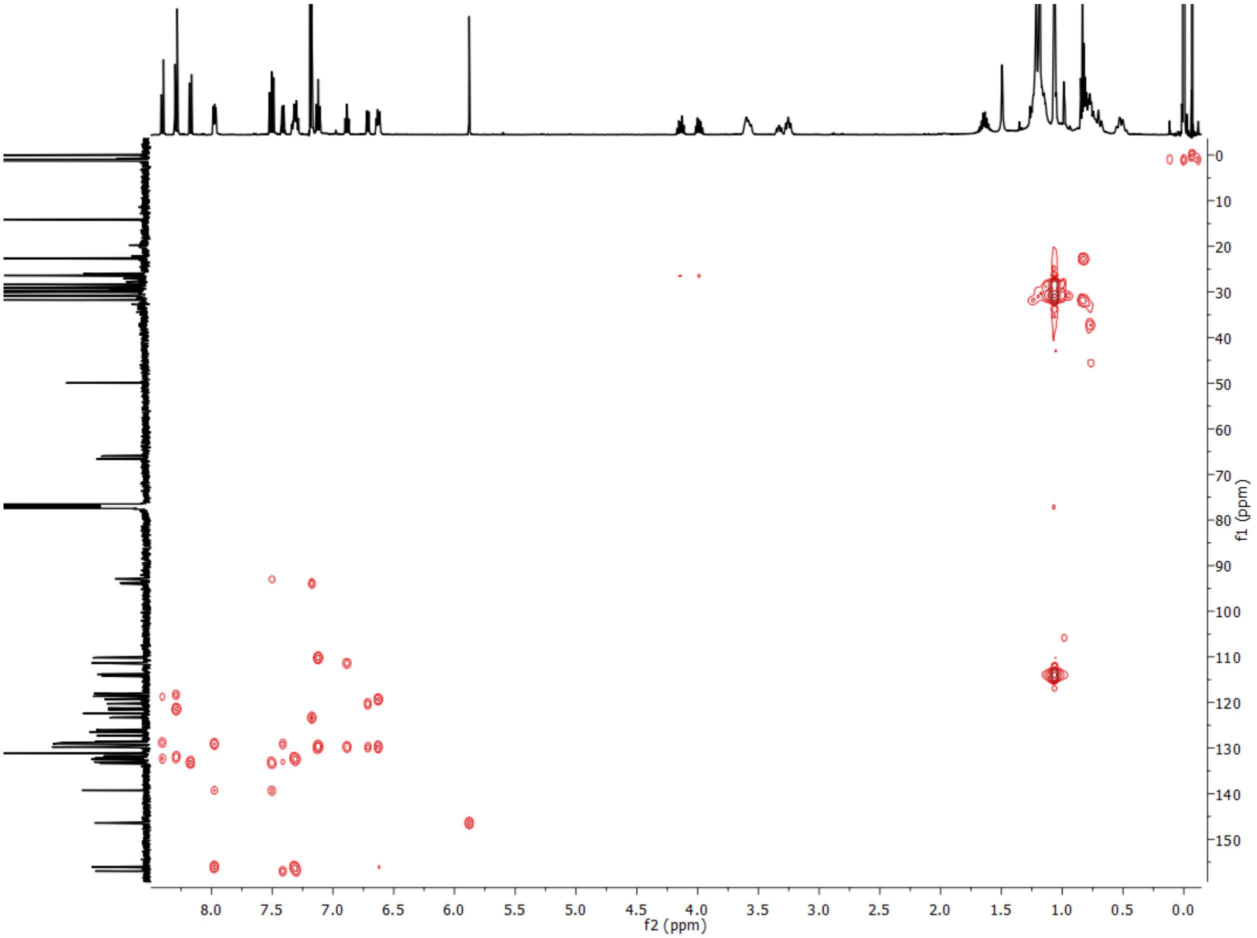

Supplementary Figure 212. HMBC NMR (500 MHz) of *P*,*P*,*P*-**3-Ant-C4** in $CDCl_3$, measured at 298 K.

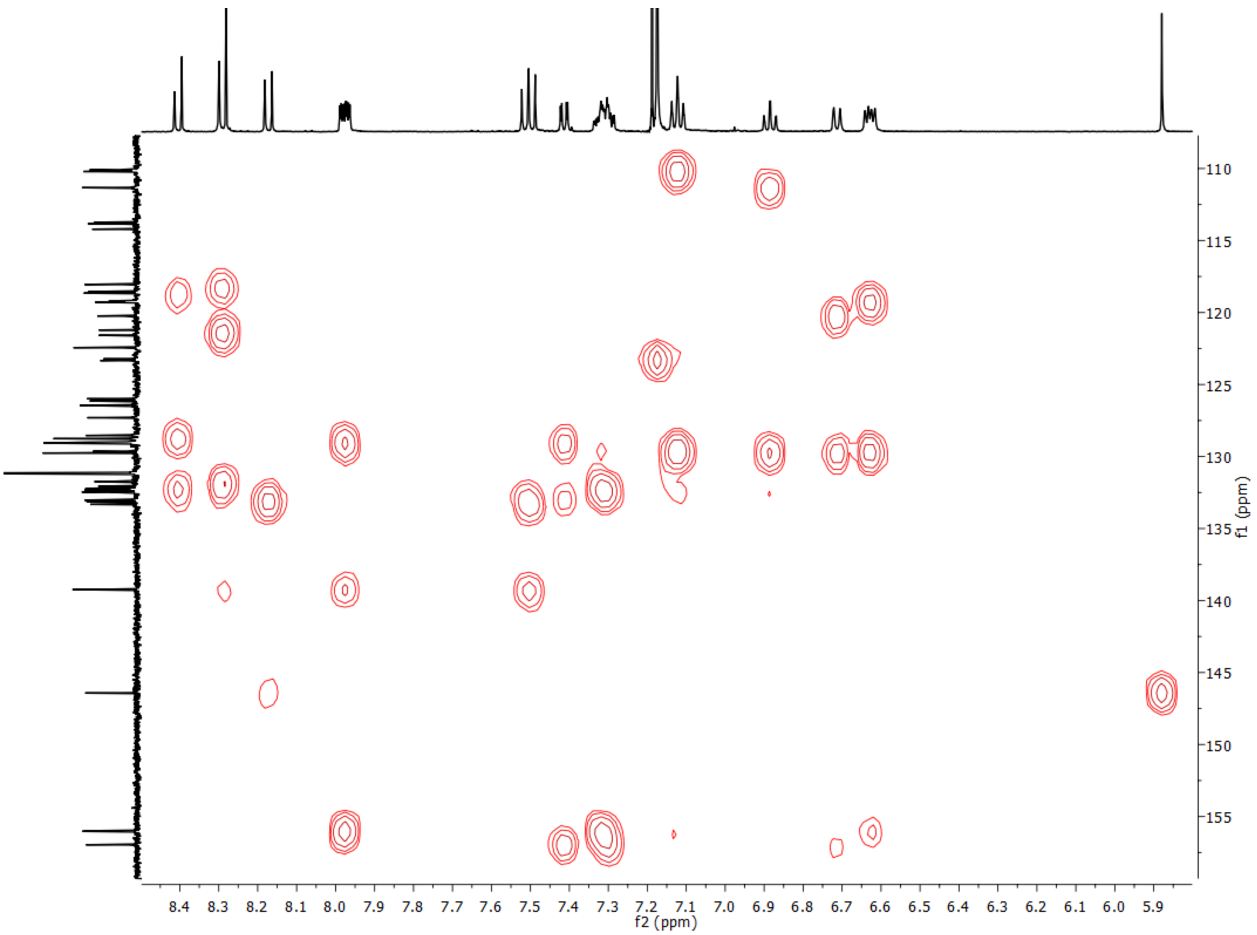

Supplementary Figure 213. HMBC NMR (500 MHz) of *P*,*P*,*P*-**3-Ant-C4** in $CDCl_3$, measured at 298 K (expansion in aromatic region).

S4 Photophysical properties

All photophysical studies were performed with dilute solutions of the compounds keeping the absorbance from the lowest energy band in the range of 0.05 to exclude self-absorption.

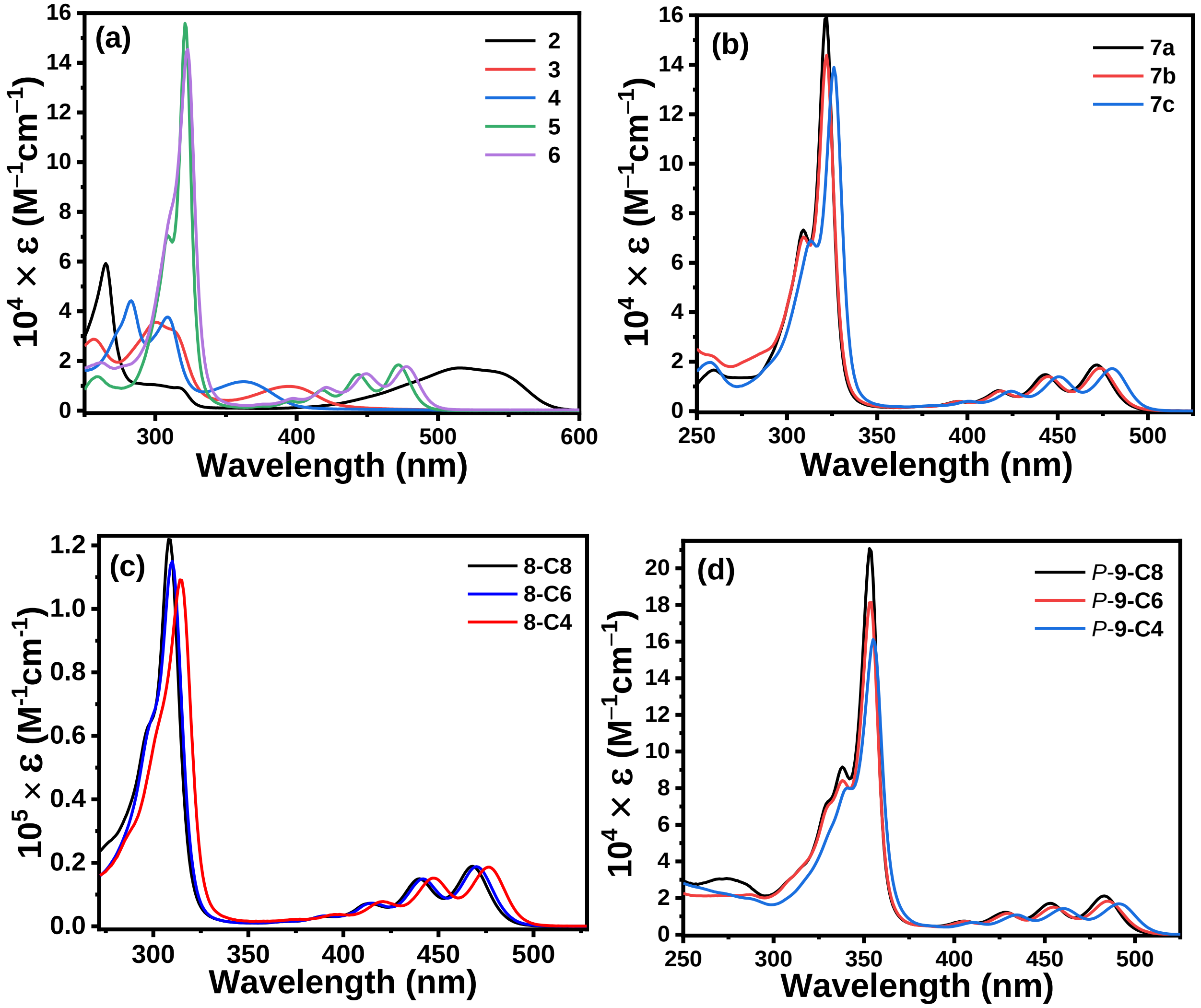

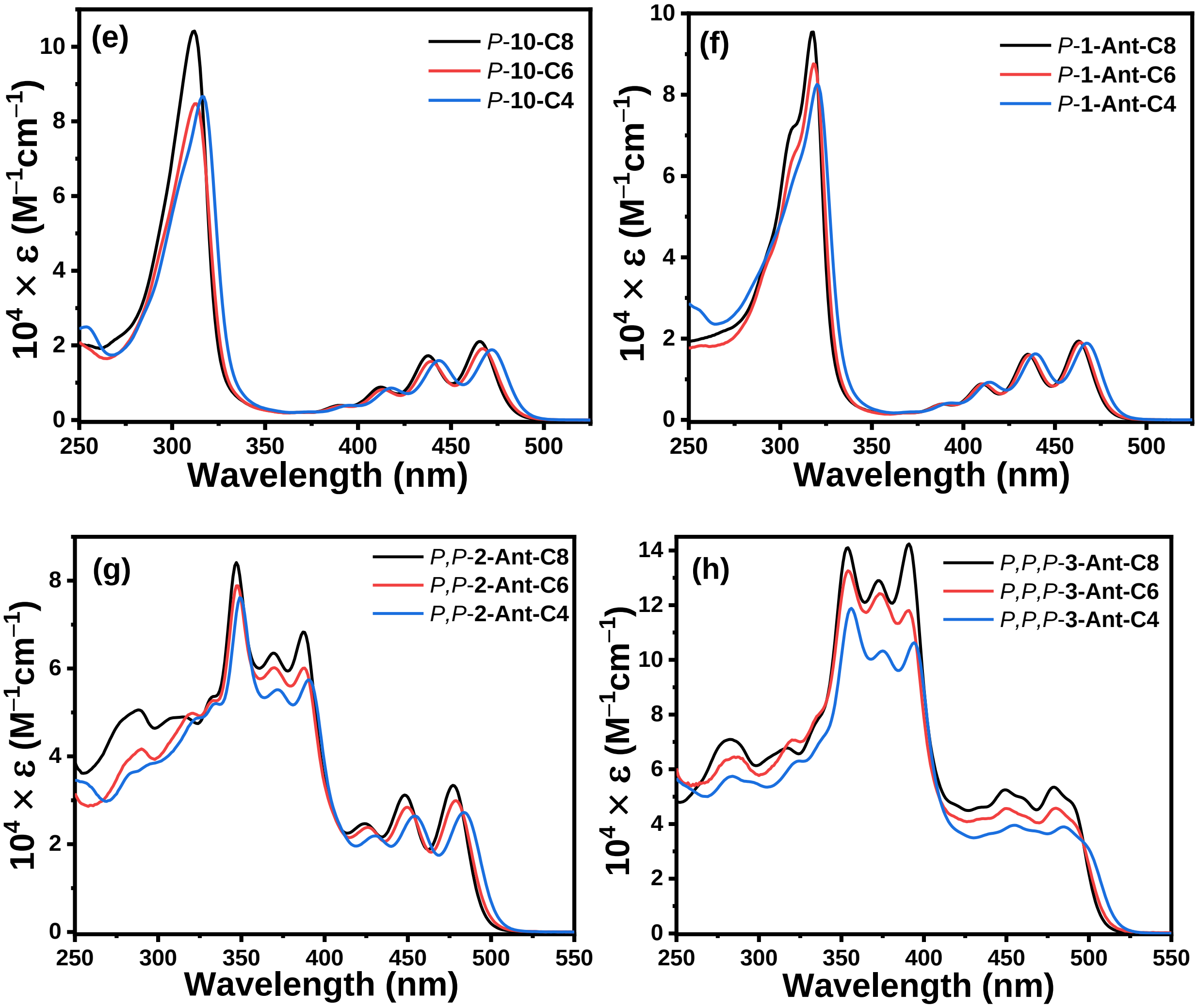

Supplementary Figure 214. UV-vis absorption spectra of compounds (a) **2–6**, (b) **7a–c** (c) **8-Cn**, (d) **9-Cn**, e) **10-Cn**, (f) *P*-**1-Ant-Cn**, (g) *P*,*P*-**2-Ant-Cn,** and (h) *P*,*P*,*P*-**3-Ant-Cn** derivatives in chloroform at 298 K.

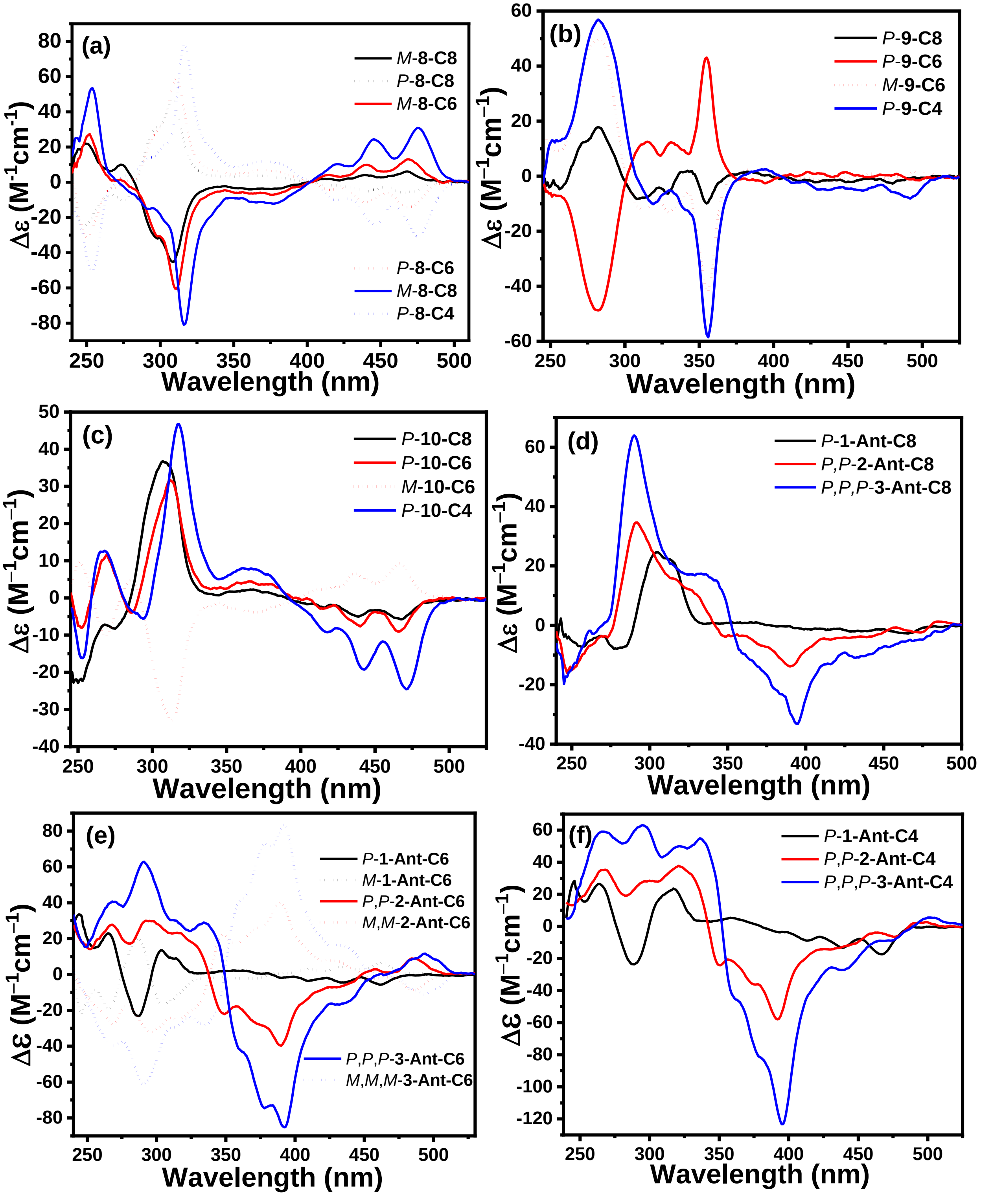

Supplementary Figure 215. CD spectra of the enantiomers of (a) **8-Cn**, (b) **9-Cn**, (c) **10-Cn**, (d) **Ant-C8** ,(e) **Ant-C6**, and (f) **Ant-C4** derivatives in chloroform at 298 K.

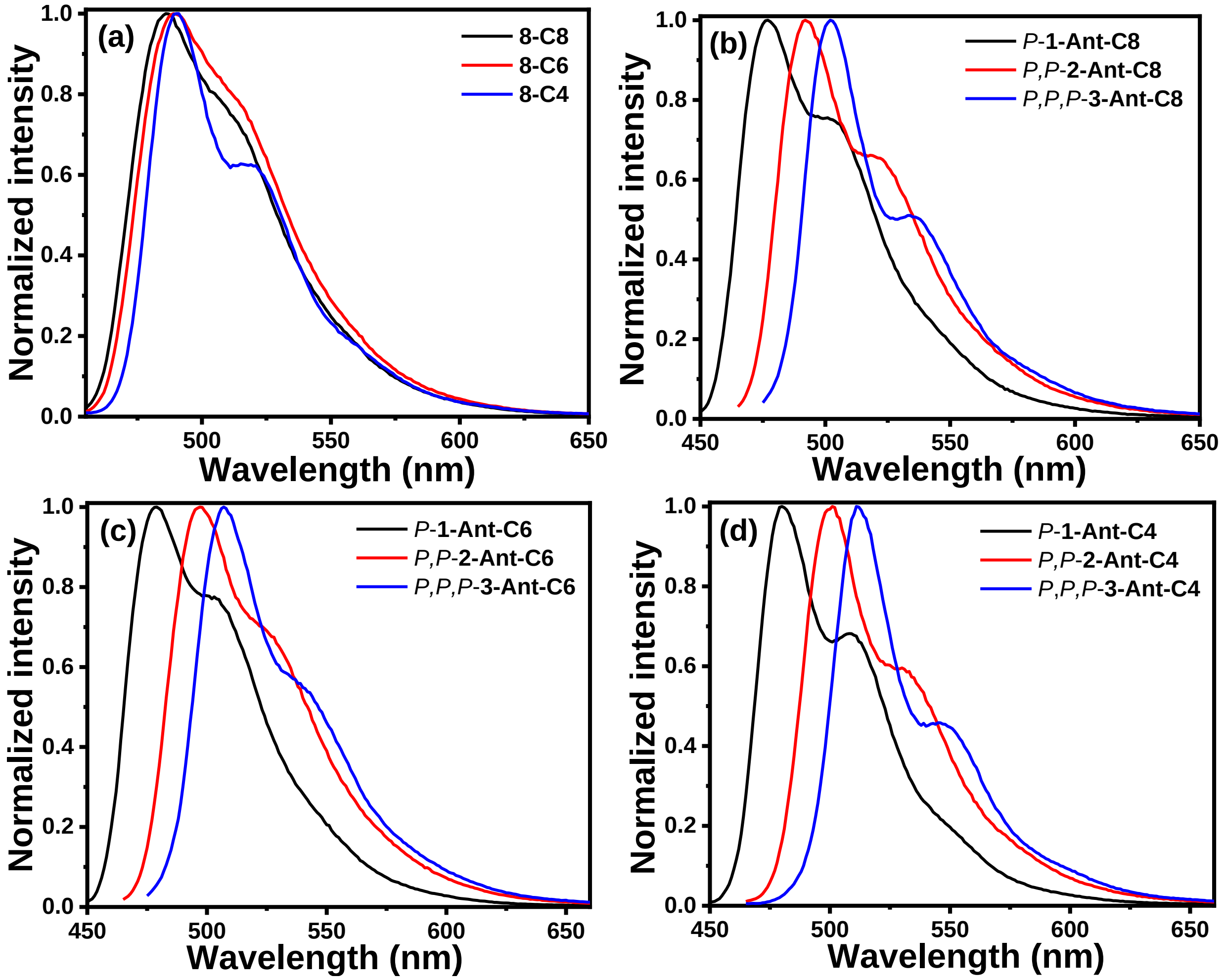

Supplementary Figure 216. Steady-state fluorescence spectra of (a), **8-Cn** (b) **Ant-C8**, (c) **Ant-C6**, and (d) **Ant-C4** derivatives in chloroform at 298 K.

S5 Single crystal X-ray diffraction crystallography (SCXRD)

Single crystals of **7-C8, 7-C4** were grown in hexane, whereas enatiopure *P*-**8-Cn** crystals were obtained from a mixture of EtOAc/hexane by the slow evaporation method. The enantiopure oligomer *P*,*P*-**2-Ant-C4** was crystallized by layering method from dichloromethane/hexane. A single crystal was attached to a 400/50 MicroMesh™ with NVH Oil,[2] and transferred to a Bruker SMART APEX CCD X-ray diffractometer equipped with a graphite-monochromator. Maintaining the crystals at the designated temperatures described below was achieved with a Bruker KRYOFLEX nitrogen cryostat (for the relevant materials). The system was controlled by a Pentium-based PC running the SMART software package.[3] Data were collected at room temperature using Mo-Kα radiation (λ=0.71073 Å). Cu-Kα radiation (λ=1.54184 Å) was used for *P*-1**-Ant-C4** and *P*,*P*-**2-Ant-C4.** Immediately after collection, the raw data frames were subjected to integration and reduction by the SAINT program package.[4] The structure was solved and refined by the SHELXTL software package.[5] Structures *P*-1**-Ant-C4** and *P*,*P*-**2-Ant-C4** were collected on a Rigaku XTALAB PRO sealed tube dual source with microfocus and Dectris Pilatus 200K, Cu-Kα radiation (λ=1.54184 Å). Data collection was performed in low temperature under LN. Data were processed with CrysAlis^{PRO} (Rigaku). Structures were solved using SHELXT. and refinement performed based on F^2 with SHELXL. and OLEX2. with full matrix least-squares. All non-hydrogen atoms were refined aniostropically. Hydrogens were placed at calculated positions and refined using a riding model. Disordered solvent contributions were treated by PLATON/SQUEEZE in structure *P*,*P*-**2-Ant-C4**.

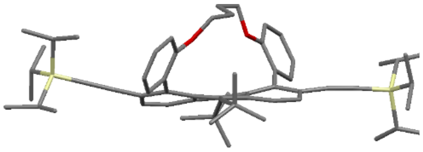

7-C4

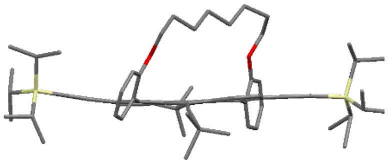

7-C8

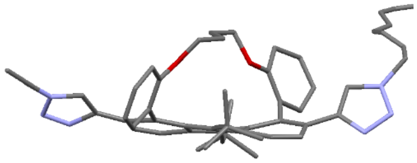

P-**1-Ant-C4**

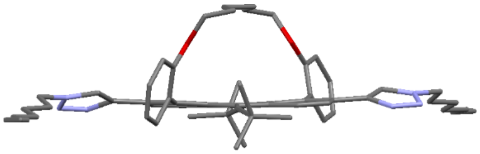

P-**1-Ant-C6**

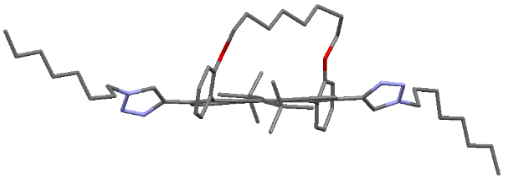

P-**1-Ant-C8**

Figure 217. Crystal structures of **7-C4**, **7-C8** and enantiopure *P*-**1-Ant-Cn**

Supplementary Table 1. Crystallographic refinement parameters of **7-C8**, **7-C4**

**Parameters**	**7-C8**	**7-C4**
Empirical formula	$C_{68}H_{66}O_2Si_2$	$C_{64}H_{80}O_2Si_2$
Formula weight	971.38	937.46
Temperature/K	293(1)	298.4(3)
Crystal system	triclinic	triclinic
Space group	P-1	P-1
a/Å	13.9224(4)	13.25610(10)
b/Å	15.4189(4)	14.04250(10)
c/Å	15.7715(6)	15.8457(2)
α/°	81.783(3)	79.5900(10)
β/°	72.655(3)	88.1030(10)
γ/°	84.518(2)	87.7660(10)
Volume/$Å^3$	3193.50(17)	2897.88(5)
Z	2	2
ρ_{calc}g/cm^3	1.010	1.074
μ/mm^{-1}	0.094	0.102
F(000)	1036.0	1016.0
Crystal size/mm^3	0.147 × 0.139 × 0.09	0.203 × 0.105 × 0.095
Radiation	Mo Kα (λ = 0.71073)	Mo Kα (λ = 0.71073)
2θ range for data collection/°	4.06 to 61.81	3.984 to 64.192
Index ranges	-18 ≤ h ≤ 19, -21 ≤ k ≤ 21, -21 ≤ l ≤ 20	-19 ≤ h ≤ 18, -20 ≤ k ≤ 19, -18 ≤ l ≤ 22
Reflections collected	45649	63814
Independent reflections	15637 [R_{int} = 0.0583, R_{sigma} = 0.0626]	17308 [R_{int} = 0.0239, R_{sigma} = 0.0265]
Data/restraints/parameters	15637/0/664	17308/0/631
Goodness-of-fit on F^2	0.991	1.044
Final R indexes [I>=2σ (I)]	R_1 = 0.0979, wR_2 = 0.2815	R_1 = 0.0596, wR_2 = 0.1687
Final R indexes [all data]	R_1 = 0.1668, wR_2 = 0.3230	R_1 = 0.0881, wR_2 = 0.1846
Largest diff. peak/hole / e $Å^{-3}$	0.54/-0.29	0.39/-0.20
CCDC number	2099695	2099696

Supplementary Table 2. Crystallographic refinement parameters of **8-Cn**

**Parameters**	**8-C8**	**8-C6**	**8-C4**
Empirical formula	$C_{50}H_{48}O_2$	$C_{48}H_{44}O_2$	$C_{46}H_{40}O_2$
Formula weight	680.88	652.83	624.78
Temperature/K	297.3(7)	297.1(7)	296.8(4)
Crystal system	triclinic	monoclinic	monoclinic
Space group	P-1	$P2_1/c$	$P2_1/c$
a/Å	12.2389(2)	17.0067(5)	14.5543(2)
b/Å	14.0885(2)	16.8726(5)	16.1578(3)
c/Å	14.5046(2)	14.6726(4)	15.7274(3)
α/°	112.6140(10)	90	90
β/°	114.548(2)	91.704(2)	100.110(2)
γ/°	95.1540(10)	90	90
Volume/$Å^3$	2005.12(6)	4208.4(2)	3641.11(11)
Z	2	4	4
$\rho_{calc}g/cm^3$	1.128	1.030	1.140
μ/mm^{-1}	0.067	0.061	0.068
F(000)	728.0	1392.0	1328.0
Crystal size/mm^3	0.251 × 0.161 × 0.046	0.152 × 0.103 × 0.051	0.283 × 0.201 × 0.098
Radiation	Mo Kα (λ = 0.71073)	Mo Kα (λ = 0.71073)	Mo Kα (λ = 0.71073)
2θ range for data collection/°	3.832 to 64.412	4.346 to 64.384	3.644 to 64.314
Index ranges	$-17 \leq h \leq 17$, $-20 \leq k \leq 20$, $-21 \leq l \leq 20$	$-22 \leq h \leq 22$, $-23 \leq k \leq 24$, $-21 \leq l \leq 19$	$-21 \leq h \leq 21$, $-23 \leq k \leq 23$, $-23 \leq l \leq 22$
Reflections collected	36691	54332	70483
Independent reflections	11824 [R_{int} = 0.0213, R_{sigma} = 0.0282]	12280 [R_{int} = 0.0498, R_{sigma} = 0.0560]	11285 [R_{int} = 0.0299, R_{sigma} = 0.0221]
Data/restraints/parameters	11824/0/475	12280/16/541	11285/88/470
Goodness-of-fit on F^2	1.072	1.013	1.057
Final R indexes [I>=2σ (I)]	R_1 = 0.0534, wR_2 = 0.1507	R_1 = 0.0780, wR_2 = 0.1640	R_1 = 0.0497, wR_2 = 0.1348
Final R indexes [all data]	R_1 = 0.0792, wR_2 = 0.1632	R_1 = 0.1506, wR_2 = 0.1915	R_1 = 0.0710, wR_2 = 0.1466
Largest diff. peak/hole / e $Å^{-3}$	0.29/-0.18	0.15/-0.14	0.30/-0.20
CCDC number	2099699	2099700	2099701

Supplementary Table 3. Crystallographic refinement parameters of *P*-**1-Ant-Cn and** *P*,*P*-**2-Ant-C4**

**Parameters**	*P*-**1-Ant-C8**	*P*-**1-Ant-C6**	*P*-**1-Ant-C4**	*P*,*P*-**2-Ant-C4**
Empirical formula	$C_{66}H_{82}N_6O_2$	$C_{64}H_{78}N_6O_2$	$C_{62}H_{74}N_6O_2$	$C_{162}H_{168}N_9O_6$
Formula weight	991.37	963.32	935.27	2337.04
Temperature/K	149.99(14)	288.00(8)	100.00(10)	100(2) K
Crystal system	monoclinic	monoclinic	Monoclinic	Triclinic
Space group	$P2_1$	I2	$P2_1$	P-1
a/Å	12.5873(2)	15.5279(6)	12.2328(6)	12.2018(7)
b/Å	8.47740(10)	7.7387(3)	7.7170 (4)	19.6729(11)
c/Å	26.8245(4)	24.4021(8)	28.199 (2)	22.6680(12)
α/°	90	90	90	75.799(5)
β/°	95.5310(10)	101.386(3)	89.953(5)	83.078(5)
γ/°	90	90	90	76.653(5)
Volume/$Å^3$	2849.05(7)	2874.59(19)	2662.0(3)	5120.8(5)
Z	2	2	2	2
$\rho_{calc}g/cm^3$	1.156	1.113	1.167	1.516
μ/mm^{-1}	0.070	0.067	0.546	0.705
F(000)	1072.0	1040.0	1008	2502
Crystal size/mm^3	0.456 × 0.252 × 0.092	0.178 × 0.047 × 0.04	0.327 × 0.041 × 0.019	0.041 × 0.040 × 0.034
Radiation	Mo Kα (λ = 0.71073)	Mo Kα (λ = 0.71073)	Cu Kα (1.54184)	Cu Kα (1.54184)
2θ range for data collection/°	3.722 to 64.29	5.278 to 61.586	3.134 to 47.238	2.369 to 47.238
Index ranges	-18 ≤ h ≤ 18, -12 ≤ k ≤ 11, -35 ≤ l ≤ 39	-22 ≤ h ≤ 19, -8 ≤ k ≤ 10, -34 ≤ l ≤ 31	-11<=h<=11, -7<=k<=7, -26<=l<=26	-9<=h<=11, -18<=k<=18, -21<=l<=21
Reflections collected	71877	18646	16551	30811
Independent reflections	16703 [R_{int} = 0.0239, R_{sigma} = 0.0242]	6557 [R_{int} = 0.0280, R_{sigma} = 0.0400]	4790 [R(int) = 0.0587, R_{sigma} = 0.0504]	9252 [[R(int) = 0.0674, R_{sigma} = 0.0843]
Data/restraints/parameters	16703/1/675	6557/1/329	4790 / 153 / 686	9252 / 441 / 1229
Goodness-of-fit on F^2	1.054	1.004	1.055	1.210
Final R indexes [I>=2σ (I)]	R_1 = 0.0419, wR_2 = 0.1131	R_1 = 0.0592, wR_2 = 0.1507	R1 = 0.0973, wR2 = 0.2646	R1 = 0.1084, wR2 = 0.2983
Final R indexes [all data]	R_1 = 0.0500, wR_2 = 0.1178	R_1 = 0.1099, wR_2 = 0.1732	R1 = 0.1074, wR2 = 0.2768	R1 = 0.1625, wR2 = 0.3331
Largest diff. peak/hole / e $Å^{-3}$	0.32/-0.24	0.15/-0.13	0.299/-0.328	0.848/-0.436
CCDC number	2099698	2099697	2099197	2099196

S6 Computational details

All calculations were carried out using the Gaussian 09 program applying density functional theory (DFT). All molecules were optimized using a hybrid density functional[6] and Becke's three-parameter exchange functional combined with the LYP correlation functional (B3LYP)[7] and with the 6-31G(d) basis set (B3LYP/6-31G(d)). To understand the UV-vis and CD spectral transition of the molecules, time dependent (TD)-DFT calculations were performed using the CAM-B3LYP functional.

S6.1 Calculated structures of the analogs of the synthesised molecules

Supplementary Table 4. Optimized (DFT-B3LYP-6-31G(d)) structures of the analogs of the synthesized molecules

Compounds	Absolute Energy (Hatree)
M-**8-C8**	-2084.443021
M-**8-C6**	-2005.818106
M-**8-C4**	-1927.195871
M-**1-Ant-C8**	-2492.852265
M-**1-Ant-C6**	-2414.228103
M-**1-Ant-C4**	-2335.603117
syn-M,M-**2-Ant-C8**	-4807.187670
anti-M,M-**2-Ant-C8**	-4807.187666
syn-M,M-**2-Ant-C6**	-4649.939387
anti-M,M-**2-Ant-C6**	-4649.939422
syn-M,M-**2-Ant-C4**	-4492.690799
anti-M,M-**2-Ant-C4**	-4492.690861
syn-M,M,M-**3-Ant-C8**	-7121.522652
anti-M,M,M-**3-Ant-C8**	-7121.522628
syn-M,M,M-**3-Ant-C6**	-6885.650715
anti-M,M,M-**3-Ant-C6**	-6885.650668
syn-M,M,M-**3-Ant-C4**	-6649.778431
anti-M,M,M-**3-Ant-C4**	-6649.778468

6.1.1 Optimized (DFT-B3LYP-6-31G(d)) geometries of 8-Cn

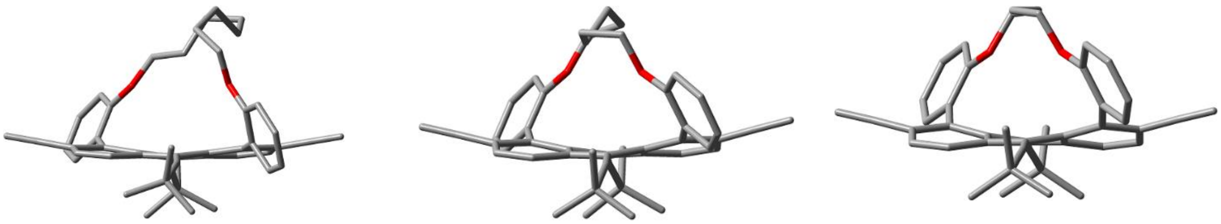

M-**8-C8** *M*-**8-C6** *M*-**8-C4**

6.1.2 Optimized (DFT-B3LYP-6-31G(d)) geometries of 1-Ant-Cn

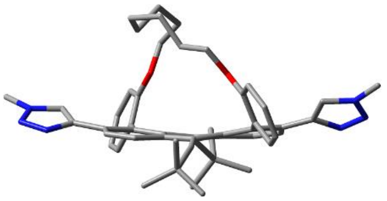

M-**1-Ant-C8**

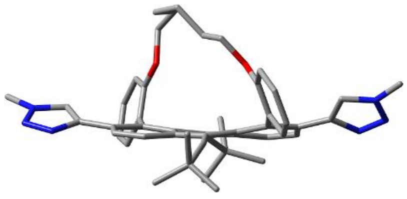

M-**1-Ant-C6**

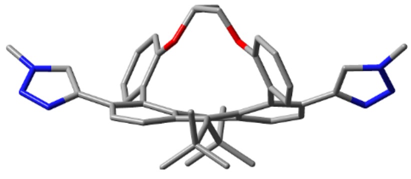

M-**1-Ant-C4**

6.1.3 Optimized (DFT-B3LYP-6-31G(d)) geometries of 2-Ant-Cn

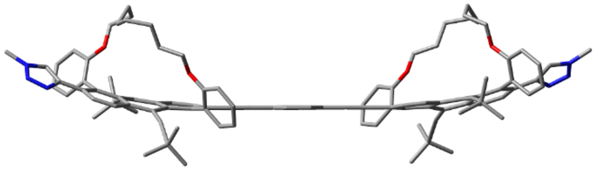

syn-*M*,*M*-**2-Ant-C8**

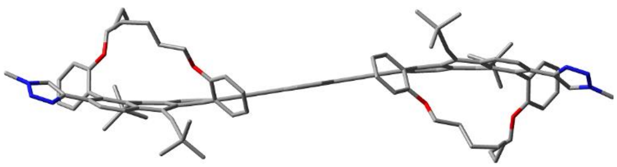

anti-*M*,*M*-**2-Ant-C8**

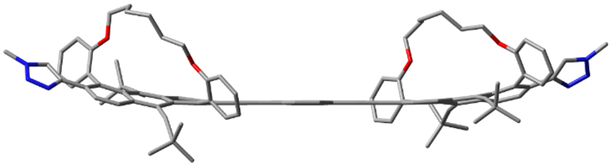

syn-*M*,*M*-**2-Ant-C6**

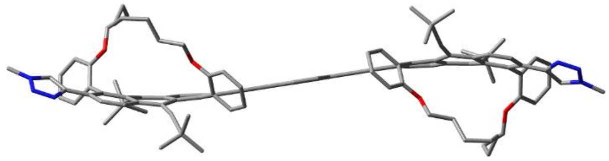

anti-*M*-**2-Ant-C6**

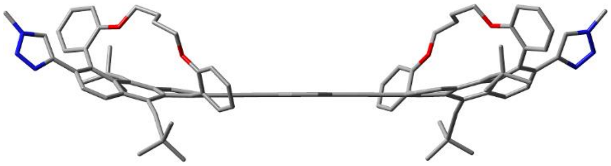

syn-*M*,*M*-**2-Ant-C4**

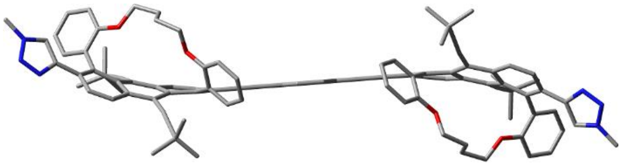

anti-*M*,*M*-**2-Ant-C4**

6.1.4 Optimized (DFT-B3LYP-6-31G(d)) geometries of 3-Ant-Cn

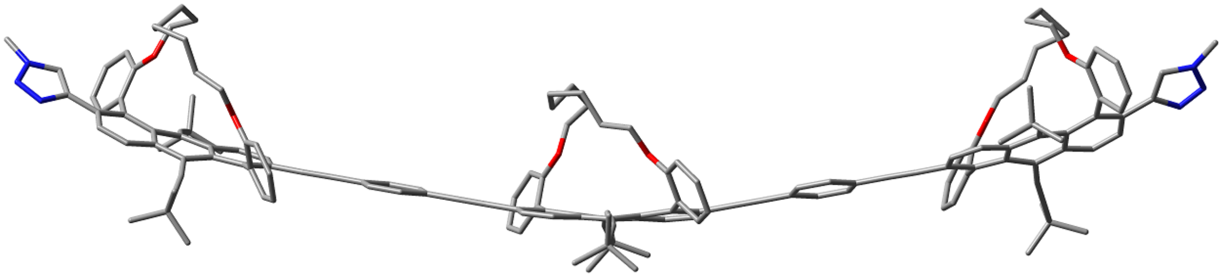

syn-*M*,*M*,*M*-**3-Ant-C8**

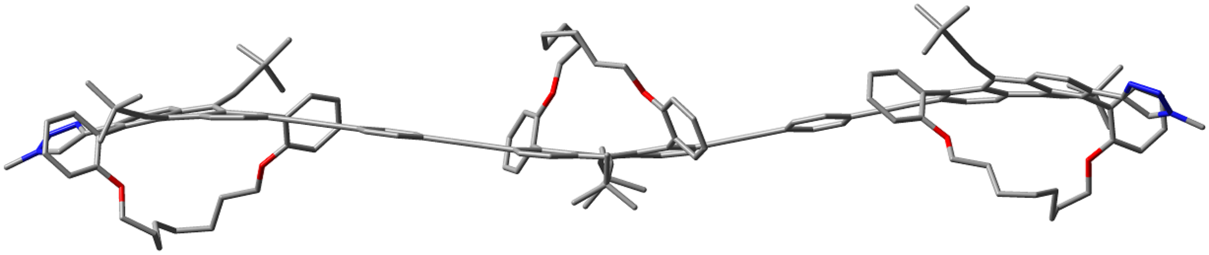

anti-*M*,*M*,*M*-**3-Ant-C8**

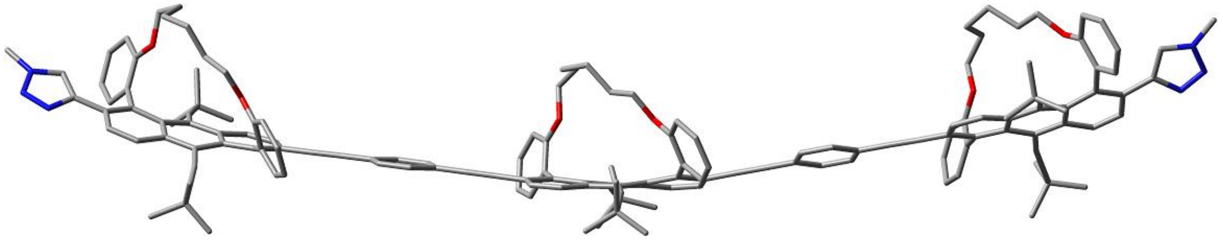

syn-*M*,*M*,*M*-**3-Ant-C6**

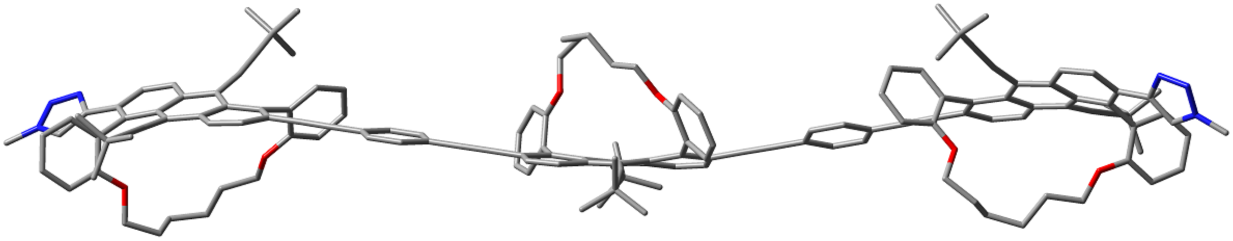

anti-*M*,*M*,*M*-**3-Ant-C6**

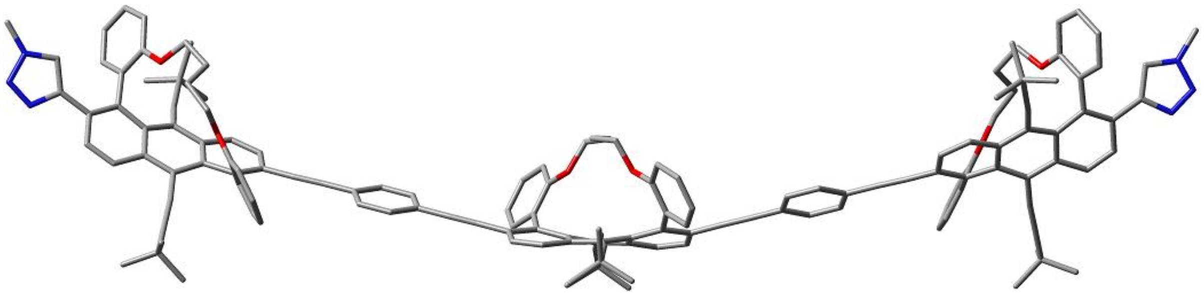

syn-*M*,*M*,*M*-**3-Ant-C4**

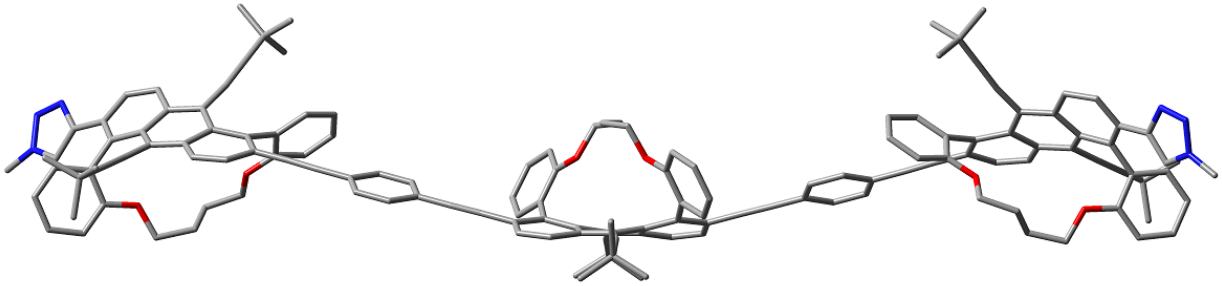

anti-*M*,*M*,*M*-**3-Ant-C4**

S6.2 Computational insights from the twisted parent anthracene oligomers

To compare the experimentally obtained optoelectronic properties of the twistacenes and their oligomers, the parent acene analogs were also examined using identical computation methods. To maintain consistency with the experimentally observed CD spectra for the synthesised molecules, which were explained by a *syn* conformation of the tethered twistacenes in oligomers, here we considered only parent oligomers in the *syn* conformation. The computational approach to twisting anthracene was as per our previously reported method.[8]

Supplementary Table 5. Optimized (DFT-B3LYP-6-31G(d)) structures of the *M*-Ant-Cn skeleton

Compounds	Absolute Energy (Hatree)
M-**Ant-10**	-539.529694
M-**Ant-20**	-539.527312
M-**Ant-30**	-539.523322
M-**Ant-40**	-539.517690
M,*M*-**2-Ant-10**	-1461.239610
M,*M*-**2-Ant-20**	-1461.234935
M,*M*-**2-Ant-30**	-1461.227112
M,*M*-**2-Ant-40**	-1461.216101
M,*M*,*M*-**3-Ant-10**	-2382.949512
M,*M*,*M*-**3-Ant-20**	-2382.942538
M,*M*,*M*-**3-Ant-30**	-2382.930884
M,*M*,*M*-**3-Ant-40**	-2382.914477

6.2.1 Optimized (DFT-B3LYP-6-31G(d)) geometries of the M-Ant-Cn skeleton

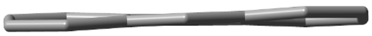

M-**Ant-10**

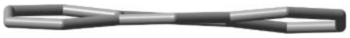

M-**Ant-20**

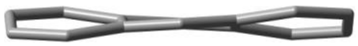

M-**Ant-30**

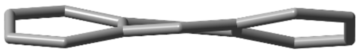

M-**Ant-40**

6.2.2 Optimized (DFT-B3LYP-6-31G(d)) geometries of the syn-M,M-2-Ant-Cn skeleton

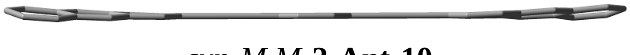

syn-*M*,*M*-**2-Ant-10**

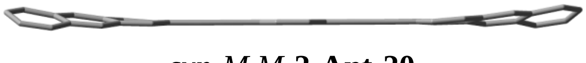

syn-*M*,*M*-**2-Ant-20**

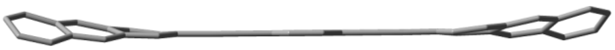

syn-M,M-**2-Ant-30**

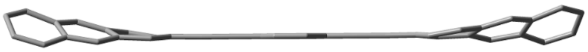

syn-M,M-**2-Ant-40**

6.2.3 Optimized (DFT-B3LYP-6-31G(d)) geometries of the M,M,M-3-Ant-Cn skeleton

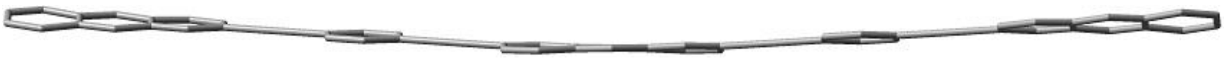

syn-M,M,M-**3-Ant-10**

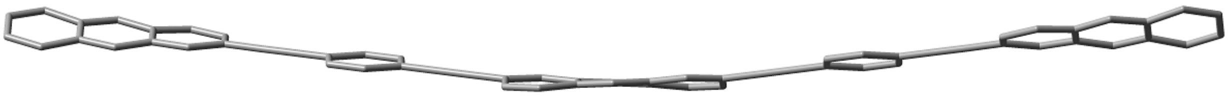

syn-M,M,M-**3-Ant-20**

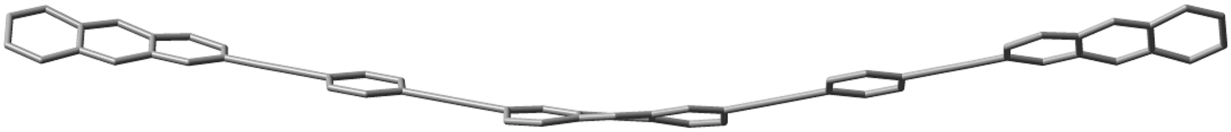

syn-M,M,M-**3-Ant-30**

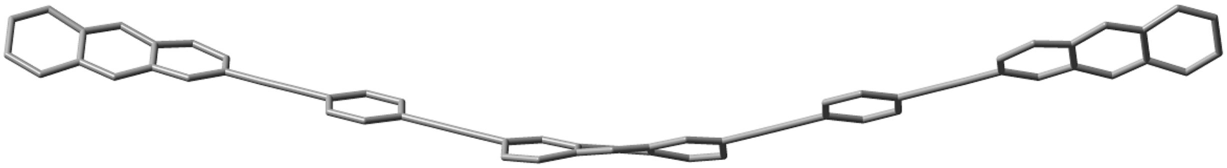

syn-M,M,M-**3-Ant-40**

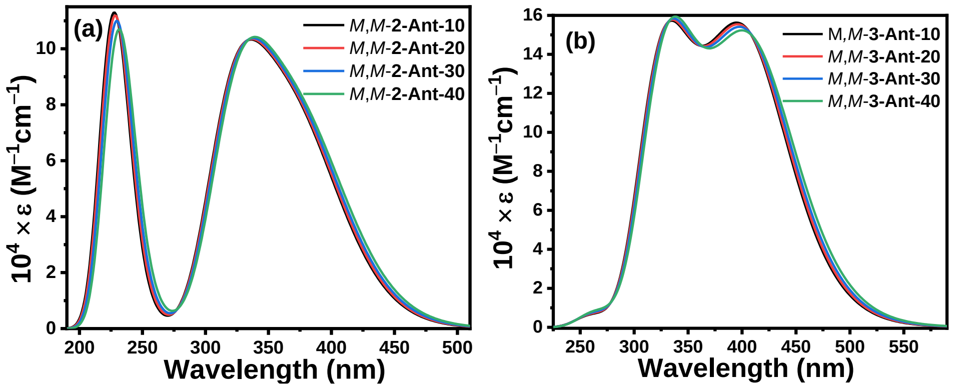

Supplementary Figure 218. Calculated (TD-DFT-6-31G(d)-CAMB3LYP) UV-vis absorption spectra of the skeletons of (a) *syn-M,M*-**2-Ant-Cn** skeleton and (b) *syn-M,M,M*-**3-Ant-Cn**.

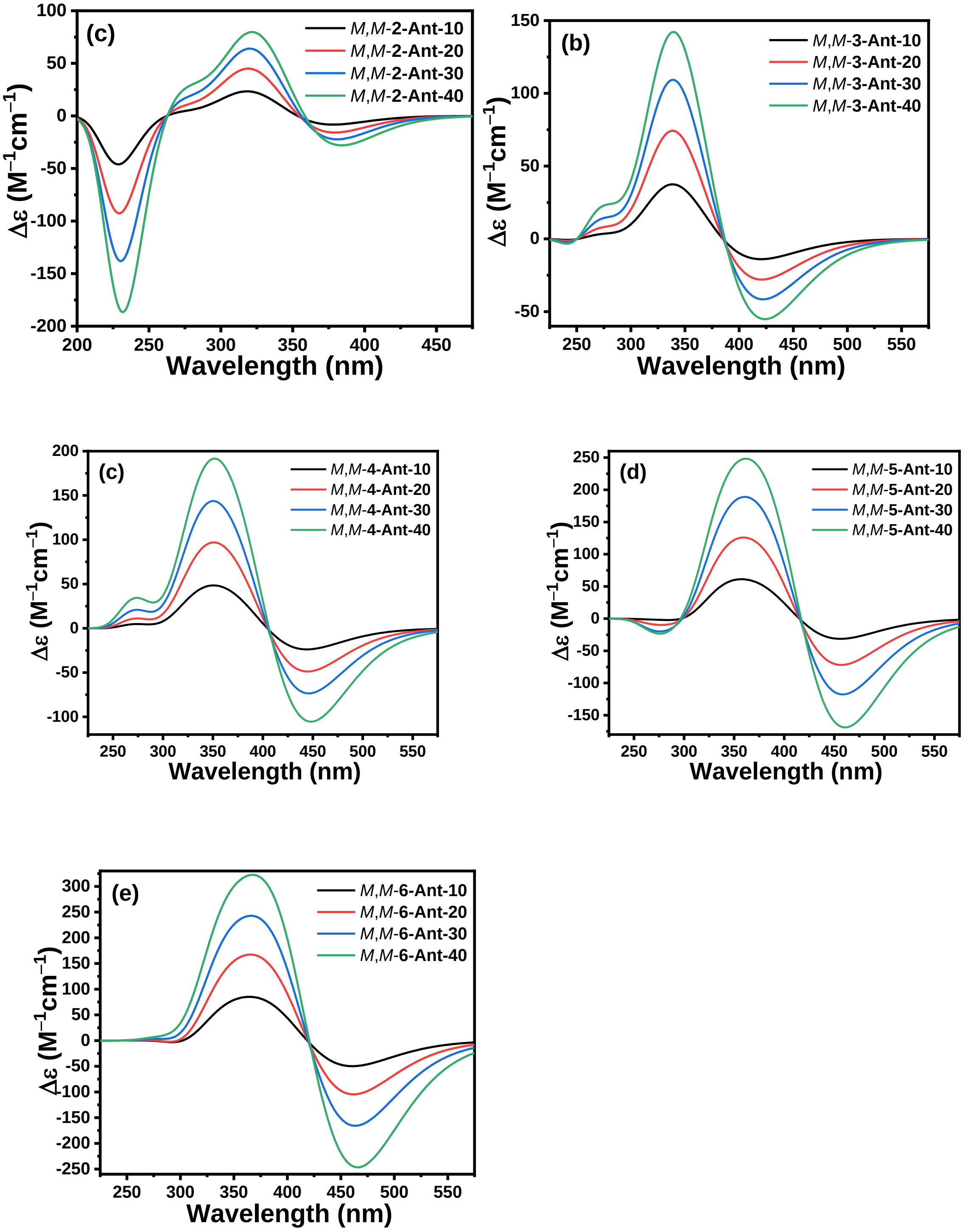

Supplementary Figure 219. Calculated (TD-DFT-6-31G(d)-CAMB3LYP) CD spectra of the skeletons of (a) *syn*-*M*,*M*-**2-Ant-Cn** skeleton (b) *syn*-*M*,*M*,*M*-**3-Ant-Cn** skeleton, (c) *syn*-*M*,*M*-**4-Ant-Cn** skeleton (d) *syn*-*M*,*M*,*M*-**5-Ant-Cn** skeleton, (e) *syn*-*M*,*M*,*M*-**6-Ant-Cn** skeleton

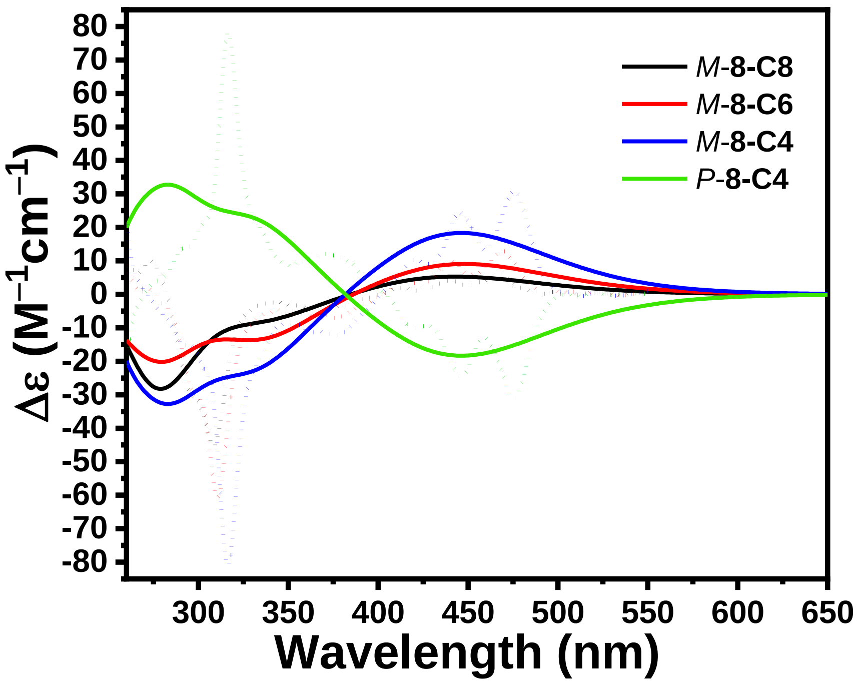

Supplementary Figure 220. Calculated – solid lines (TD-DFT-6-31G(d)-CAMB3LYP) and experimental (dotted line, measured in chloroform) CD spectra for *P* and *M* enantiomers of **8-Cn**.

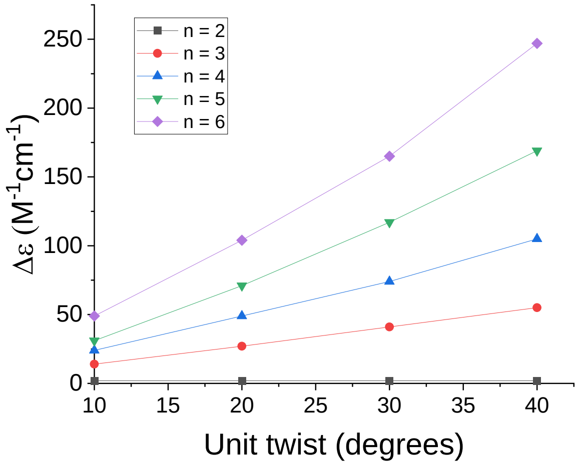

Supplementary Figure 221. Calculated (TD-DFT/CAM-B3LYP/6-31G(d)) maximal molar circular dichroism for the lowest energy transition for skeleton of **n-Ant-C**, with end-to-end twisting of 10° to 40° where n = number of repeat units.

To further verify that increase in Δε indeed stems from the helical structure, we normalized the difference in Δε between 10° to 40° (ΔΔε = (Δε(40°-10°)/n)) by the number of repeat units (ΔΔε = (Δε(40°-10°)/n) where n = number of repeat units). After normalization, the ΔΔε increases from 13.6 to the trimer up to 33 for the monomer. Our computational results are in agreement with overall amplification stemming from the secondary structure.

6.2.4 Transition dipole moment of the skeletons M,M-2-Ant-40 and M,M,M-3-Ant-40

(a)

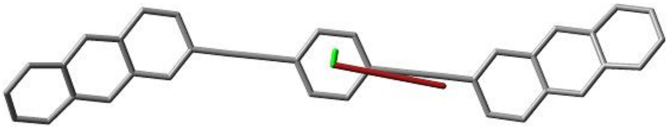

(b)

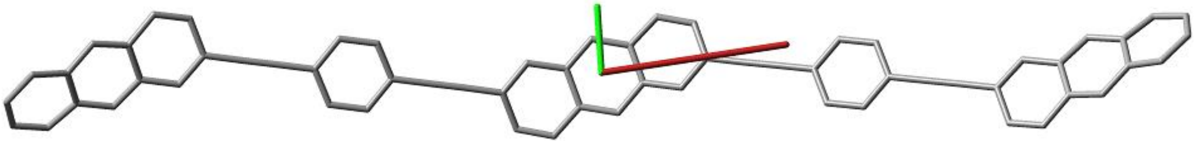

Supplementary Figure 222. Calculated (DFT-B3LYP-6-31G(d)) electric and magnetic transition dipole moments of the skeletons of (a) *M*,*M*-**2-Ant-40** and (b) *M*,*M*,*M*-**3-Ant-40**.

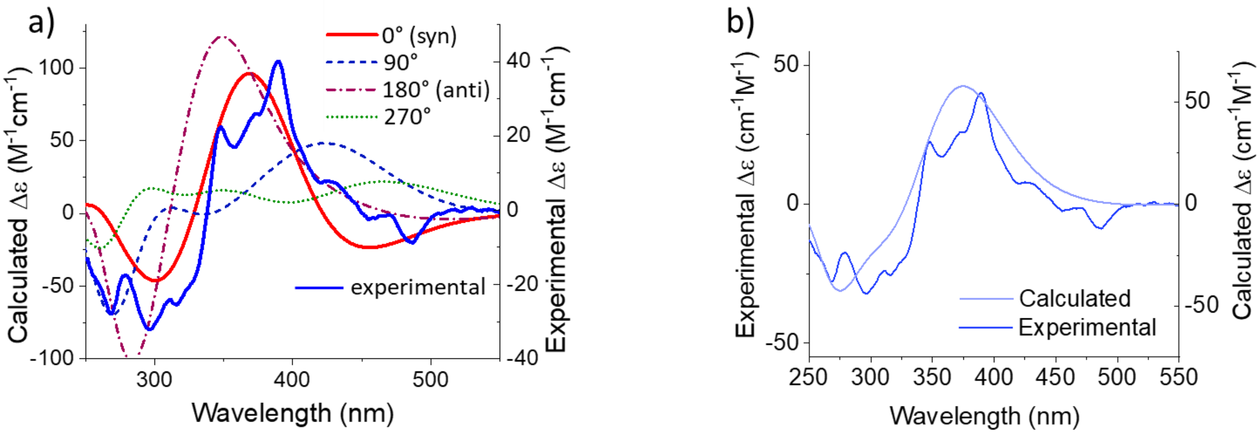

Supplementary Figure 222. Calculated (TD-DFT/B3LYP/6-31G(d)) ECD spectra for *P*,*P*-**2-Ant-C6** (a) in different conformations; (b) averaged calculated spectra of *syn* and *anti* conformers *vs*. experimental spectra.

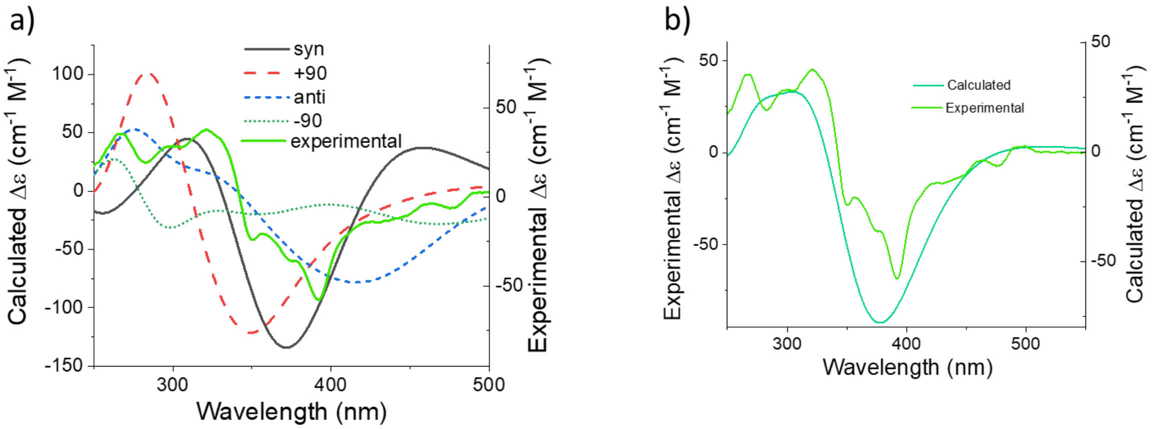

Supplementary Figure 223. Calculated (TD-DFT/B3LYP/6-31G(d)) ECD spectra for *M*,*M*-**2-Ant-C6** (a) in different conformations; (b) averaged calculated spectra of *syn* and *anti* conformers *vs*. experimental spectra.

Supplementary References

[1] Seidel, N.; Seichter, W.; Weber, E., 1,5-Diamino-2,6-dibromo-9,10-anthraquinone. *Acta Crystallogr Sect E Struct Rep Online* **2012,** *68* (Pt 3), o838-o838.

[2] MiTeGen, LLC P.O. Box 3867 Ithaca, NY 14852.

[3] SMART-NT V5.6, BRUKER AXS GMBH, D-76181 Karlsruhe, Germany, 2002.

[4] SAINT-NT V5.0, BRUKER AXS GMBH, D-76181 Karlsruhe, Germany, 2002.

[5] SHELXTL-NT V6.1, BRUKER AXS GMBH, D-76181 Karlsruhe, Germany, 2002.

[6] (a) Parr, R. G.; Yang, W. Density-functional Theory of Atoms and Molecules, Oxford University Press, New York, **1989**. (b) Koch, W.; Holthausen, M. C. A Chemist's Guide to Density Functional Theory, Wiley-VCH, New York, **2000**.

[7] Lee, C.; Yang, W.; Parr, R. G. Phys. Rev. B 1988, 37, 785. (b) Becke, A. D. J. Chem. Phys. **1993**, *98*, 5648.

[8] Bedi, A.; Gidron, O., *Chem. Eur. J.*, **2019**, *25*, 3279−3285.
